# Regulation of IL-17A Production Is Distinct from IL-17F in a Primary Human Cell Co-culture Model of T Cell-Mediated B Cell Activation

**DOI:** 10.1371/journal.pone.0058966

**Published:** 2013-03-07

**Authors:** Andrew C. Melton, Jennifer Melrose, Liisa Alajoki, Sylvie Privat, Hannah Cho, Naomi Brown, Ana Marija Plavec, Dat Nguyen, Elijah D. Johnston, Jian Yang, Mark A. Polokoff, Ivan Plavec, Ellen L. Berg, Alison O'Mahony

**Affiliations:** BioSeek, a division of DiscoveRx, Corp., South San Francisco, California, United States of America; McGill University, Canada

## Abstract

Improper regulation of B cell responses leads to excessive production of antibodies and contributes to the development of autoimmune disease. T helper 17 (Th17) cells also drive the development of autoimmune disease, but the role of B cells in shaping Th17 cell-mediated immune responses, as well as the reciprocal regulation of B cell responses by IL-17 family cytokines, remains unclear. The aim of this study was to characterize the regulation of IL-17A and IL-17F in a model of T cell-dependent B cell activation. Stimulation of primary human B cell and peripheral blood mononuclear cell (BT) co-cultures with α-IgM and a non-mitogenic concentration of superantigens for three days promoted a Th17 cell response as evidenced by increased expression of Th17-related gene transcripts, including *Il17f*, *Il21*, *Il22*, and *Il23r*, in CD4 T cells, as well as the secretion of IL-17A and IL-17F protein. We tested the ability of 144 pharmacologic modulators representing 91 different targets or pathways to regulate IL-17A and IL-17F production in these stimulated BT co-cultures. IL-17A production was found to be preferentially sensitive to inhibition of the PI3K/mTOR pathway, while prostaglandin EP receptor agonists, including PGE2, increased IL-17A concentrations. In contrast, the production of IL-17F was inhibited by PGE2, but selectively increased by TLR2 and TLR5 agonists. These results indicate that IL-17A regulation is distinct from IL-17F in stimulated BT co-cultures and that this co-culture approach can be used to identify pathway mechanisms and novel agents that selectively inhibit production of IL-17A or IL-17F.

## Introduction

Activation of T and B cells by cognate interactions is critical for adaptive immune responses, but improper regulation of this process can drive the development of autoimmune disease. B cell activation is triggered by BCR-mediated binding of antigen in secondary lymphoid structures, where B cells present antigen-derived peptides on HLA proteins and interact with cognate T cells [1]. This interaction, in conjunction with the engagement of CD40 on B cells by CD40L on T cells, results in B cell proliferation, class switching and somatic hypermutation of antibody genes, and formation of memory B cell populations. However, the mechanisms that regulate T cell-dependent B cell activation, and B cell-dependent T cell activation in the context of autoimmune disease are less well understood.

Th17 cells, a CD4 T helper subset characterized by the production of IL-17A, IL-17F, IL-21, and IL-22 cytokines, are implicated in the pathogenesis of many autoimmune diseases [2]. While this T cell lineage is beneficial for defense against certain extracellular bacterial and fungal infections, the secretion of IL-17A and IL-17F by Th17 cells may contribute to autoimmune disease pathogenesis by promoting the accumulation of neutrophils within tissues [3]. Th17 cells can also modulate B cell activation. For example, IL-21, a Th17 cell product, is required for the differentiation of B cells into antibody-secreting plasma cells, and IL-21-deficient mice show impaired persistence of germinal centers (GC) [4], [5]. Also, mice housed under germ-free conditions have diminished B cell responses and lack splenic GCs, which is associated with a loss of Th17 cells [6]. Moreover, Th17 cells can induce B cell proliferation and promote antibody isotype switching [7]. Although Th17 cells influence GC formation and B cell function, the role of B cells in influencing Th17 responses remains to be defined.

Recent clinical studies with B cell depleting antibodies support a role for B cells in the development and survival of Th17 cells in humans. Treatment of rheumatoid arthritis (RA), systemic lupus erythematosus (SLE), multiple sclerosis (MS), or Sjögren's syndrome (SS) patients with rituximab, a CD20 antibody that depletes B cells, also decreased the number of circulating Th17 cells and serum IL-17A levels [8], [9]. In addition, patients with primary B cell deficiencies show a severe reduction in circulating Th17 cells [10]. These studies suggest that, in addition to producing pathogenic antibodies, B cells may contribute to autoimmune disease by facilitating the development of pathogenic Th17 cells.

In this study we used a co-culture model of T cell-dependent B cell responses to study the B cell regulation of Th17 cells. IL-17F protein levels, and to a lesser extent IL-17A, were highly increased in co-cultures of peripheral blood mononuclear cells (PBMC) and B cells (BT co-cultures) after 3 days of stimulation with α-IgM and a low concentration of superantigens. In order to investigate the mechanisms that regulate production of IL-17A and IL-17F during B cell-dependent T cell activation, we screened 144 pharmacologic modulators that covered a broad spectrum of biologic mechanisms. We identified a number of pathways or targets that selectively impacted either IL-17A or IL-17F alone, or both together. Thus, the production of IL-17A and IL-17F by BT co-cultures is controlled through distinct pathways that can be independently regulated.

## Materials and Methods

### Human Cells

Frozen vials of positively selected primary normal human CD19+ B cells and negatively selected CD4+ T cells were obtained from AllCells (Emeryville, CA). PBMC were isolated from buffy coats (Stanford Blood Center, Stanford, CA) as previously described [11]. These studies follow the guidelines for human subjects research under United States HHS human subjects regulations (45 CFR Part 46).

### Stimulated BT Co-cultures

A co-culture system that models human B cell-dependent T cell responses (BT BioMAP® system) has been previously described [12]. PBMC, CD19+ B cells, and in some experiments CD4+ T cells, were prepared by thawing and washing once in medium containing DMEM/F12 (CellGro, Manassas, VA), 10% heat-inactivated FBS (Hyclone, Logan, UT), 2 mM L-Glutamine (CellGro), 500 IU/ml Penicillin and 500 μg/ml Streptomycin (CellGro), 1 mM Na-Pyruvate (Gibco, Grand Island, NY), 55 μM 2-mercaptoethanol (Gibco), and 12.5 nM Hydrocortisone (Sigma, St. Louis, MO). Pooled B cells (25,000 cells/well) and PBMC (25,000 cells/well) from 3–5 donors were then added to round-bottom 96-well plates (Costar, Corning, NY) and stimulated with 5 μg/ml goat (Fab')2 anti-human IgM low endotoxin/azide-free (α-IgM, Southern Biotech, Birmingham, AL) and a cocktail of superantigens (SAg), containing 0.02 ng/ml Staphylococcal enterotoxin B (SEB, Toxin Technologies, Sarasota, FL) and 0.02 ng/ml Toxic shock syndrome toxin-1 (TSST-1, Toxin Technologies) for the indicated times.

### Flow Cytometry

Intracellular cytokine staining and FACS analysis were performed as previously described [13]. Briefly, cells were stimulated with 50 ng/ml (PMA, Sigma), 1 μM ionomycin (Sigma), and 2 μM monensin (eBioscience, San Diego, CA) for 5 hours, washed, and stained with a dead cell marker (Live/Dead Fixable Near-IR, Life Technologies, Grand Island, NY). Cells were then blocked with Human FcR Blocking Reagent (Miltenyi Biotech, Auburn, CA) and stained at 4°C with fluorophore-conjugate antibodies (eBioscience or BD Pharmingen) to CD3 (clone OKT3), CD4 (clone OKT4), CD8 (clone SK1), CD19 (clone HIB19), and CD56 (clone CMSSB). After washing, cells were fixed, permeabilized, and stained with antibodies to IL-17A (clone N49–653), IL-17F (clone O33–782), or a mouse IgG1, ê isotype control antibody (clone X40). FACS analysis was performed on an LSRII (BD Biosciences) and post-analysis of flow cytometry data was performed with FlowJo software (Tree Star Inc.).

### Cytokine, IgG, Proliferation and Cytotoxicity Measurements

Cytokine concentrations in 72 hour-culture supernatants were measured by ELISA as previously described [14]. Mouse antibodies for the detection of human IL-2 and IL-6 were from R&D Systems (Minneapolis, MN), antibodies for IL-17F were from eBioscience, and antibodies for TNFα were from Invitrogen (Grand Island, NY). Mouse antibodies for IL-17A were from R&D Systems or eBioscience, and comparable results were obtained with antibodies from either source. Soluble human IgG was measured in 6 day-culture supernatants with a Human IgG ELISA kit from Bethyl Laboratories (Montgomery, TX). Proliferation was determined by quantitation of AlamarBlue (Invitrogen) reduction. AlamarBlue was added at a 1∶10 dilution to 72-hour cultures and 12 hours later absorbance was measured with a Victor^2^ plate reader (Perkin Elmer, Waltham, MA) set at 546 nm. Drug effects on cell viability (i.e., cytotoxicity) were measured in a similar fashion by adding AlamarBlue to 18–24 hour cultures. Drug-treated samples showing a decrease in absorbance > Log_10_ −0.2 compared to stimulated samples without drug were identified as overtly cytotoxic and were not included in analyses.

### Screen

Pharmacologic agents used for the screen of stimulated BT co-cultures were obtained from the sources listed in Table S3. Test agents were prepared in DMSO (0.1% final concentration) and added to co-cultures 1 hour before the addition of α-IgM and SAg. Dose ranges were selected based on published reports and our previous experience with these compounds [14]. Each assay plate included a number of controls, for which the measured values were used as criteria for inclusion of data into the screen, as previously reported [14], [15]. Briefly, for each plate the Log_10_ of the ratio of the average values measured for the positive control stimulation wells was divided by the calculated average values for the DMSO control non-stimulated wells (6 to 12 DMSO control samples from the same plate). Data were generated from multiple pools of donors and significance prediction envelopes (95%) were calculated for historical controls as previously described [11], [14]. Plates also contained control stimulation wells with colchicine (1.11 µM). Data from the agent screen were generated from individual experiments performed with a single well per readout parameter, per concentration tested.

### Gene Expression Studies

For microarray analysis, B cells and PBMC were cultured as above for three days with and without α-IgM and SAg stimulation, and then washed twice with PBS and lysed in RLT Buffer (Qiagen, Valencia, CA). Samples were then amplified and run on an Illumina HumanHT-12 v4 Expression BeadChip (Qiagen Genomics Services). Data was exported from GenomeStudio (Illumina, San Diego, CA) and normalized using quantile normalization in GeneSpring software (Agilent, Santa Clara, CA). A paired t Test was performed within the Multiple Array Viewer function of MultiExperiment Viewer software [16] and was used to calculate the fold change, absolute t-value, FDR, and *P*-value for each gene. Gene comparisons were between donor-matched stimulated and non-stimulated control B cell and PBMC co-cultures. Data were deposited in the National Center for Biotechnology Information Gene Expression Omnibus database under accession number GSE42567. For Th17-related gene analysis, B cells and PBMC were cultured as above and CD4 T cells and B cells were isolated by positive FACS sorting using a FACSAria II (BD Biosciences) at the Laboratory for Cell Analysis (UCSF, San Francisco, CA). Sorted B and CD4 T cells used for gene expression analysis were greater than 98% pure (data not shown). The following antibodies (from BD, San Jose, CA or eBioscience) were used for cell sorting: α-CD19 (clone HIB19), α-CD79b (clone CB3-1), α-CD4 (clone RPA-T4), α-CD3 (clone SK7). Purified cells were immediately lysed in RLT Buffer and stored at −80°C. RNA was isolated and qRT-PCR was performed using a Human Th17 for Autoimmunity & Inflammation RT^2^ Profiler PCR Array (Qiagen Genomics Services). Fold changes were calculated for each gene by dividing Illumina AU values or RT-PCR expression values (2^−Ct^) for stimulated samples by the respective values for non-stimulated samples.

### Statistical Analysis

Statistical analyses for gene expression studies are described above. For cytokine measurements, paired data were evaluated using a 2-tailed Student's *t* test. Differences with a *P* value less than 0.05 were considered statistically significant.

## Results

### Primary Human B Cells Contribute to the Polarization of CD4 T Cells to a Th17 Phenotype in a Model of T Cell-Dependent B Cell Activation

We have previously described a co-culture assay with primary human B cells and PBMC stimulated with α-IgM and a relatively low concentration (0.02 ng/ml) of the SEB and TSST-1 superantigens (SAg) that models T cell-dependent B cell activation (BT BioMAP system) [12]. The low concentration of SAg used in this model facilitates T cell-dependent B cell activation with minimal effects on T cell proliferation [17]. This concentration of SAg allows us to interrogate the mechanisms that regulate T cell cytokine production independently of T cell proliferation-dependent effects. SAg also masks any allogeneic reaction that may occur from mixing cells from multiple donors. In characterizing this model, we measured genome-wide mRNA expression levels by microarray in B cell and PBMC (BT) co-cultures after three days of stimulation with α-IgM and SAg. Interestingly, *Il17f* was the most strongly induced gene in co-cultures after three days of stimulation (Table 1 and Table S1). This finding suggests that activation conditions relevant for T cell-dependent B cell activation also contribute to B cell-dependent T cell responses, resulting in the production of IL-17 family cytokines by one or more cell types.

**Table 1:** IL-17F is the most strongly induced gene in BT co-cultures after three days of stimulation in a model of T cell-dependent B cell activation^a^.

Gene Symbol	Stim B/PBMC Mean	Stim B/PBMC Std. Dev.	Control B/PBMC Mean	Control B/PBMC Std. Dev.	Absolute t value	*P* value	FDR	Fold Change
IL17F	1907.4	615.1	121.1	12.2	4.9	0.039	0.446	15.75
HS.579631	2203.0	557.6	311.8	13.4	5.8	0.029	0.445	7.06
FASN	4308.0	1441.3	661.2	407.9	6.0	0.027	0.448	6.52
CD1C	690.7	197.0	127.4	3.0	5.0	0.038	0.447	5.42
KIAA0101	1622.1	708.8	311.5	208.7	4.4	0.048	0.450	5.21
TYMS	2824.1	986.7	543.2	427.0	6.9	0.020	0.447	5.20
UHRF1	3838.3	1639.2	807.9	748.1	4.9	0.039	0.444	4.75
AURKB	1327.2	477.4	289.3	197.8	6.4	0.024	0.453	4.59
TOP2A	1779.7	739.1	399.5	324.7	5.7	0.029	0.445	4.46
SCD	2975.5	1321.3	669.7	425.0	4.4	0.048	0.450	4.44

a The top ten induced genes as determined by microarray analysis in BT co-cultures stimulated with α-IgM and SAg for three days compared to the same cells co-cultured for three days without stimulation. Data are from 3 independent replicates with 3 different donor pools.

**Figure 1:**
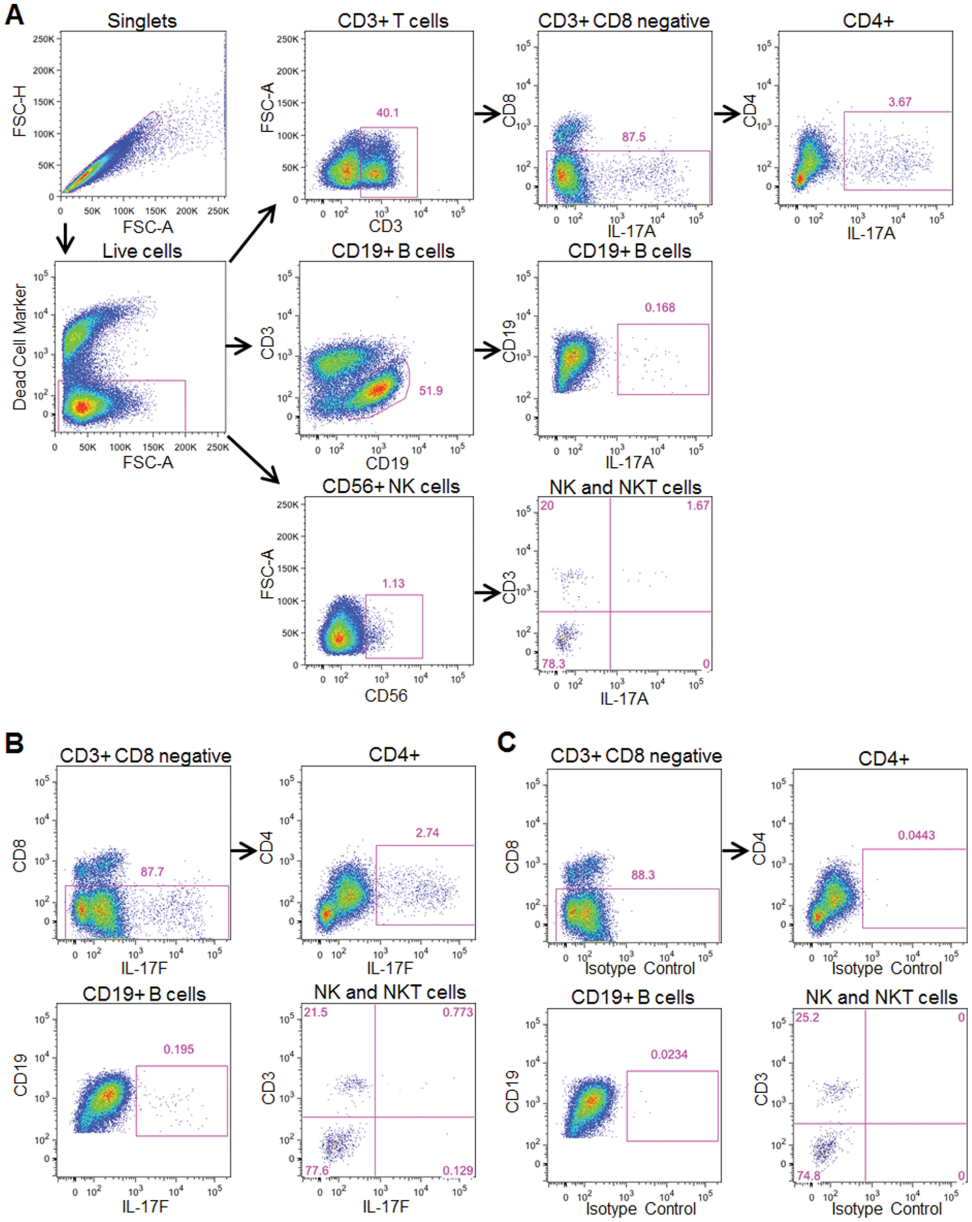
IL-17A and IL-17F are predominantly expressed by CD4 T cells in a BT co-culture model of human B cell-dependent T cell responses. FACS analysis gating strategy for cell types present in BT co-cultures after stimulation for three days with α-IgM and SAg (A). Gated cell populations are listed above the FACS plots and the percentage of cells present in each gate relative to the parent population is shown. To detect intracellular cytokine expression, BT co-cultures were treated for 5 hours with PMA, ionomycin, and monensin, and then stained with antibodies specific to IL-17A (A), IL-17F (B), or with an isotype control antibody common to the isotype of α-IL-17A and α-IL-17F (C).

To determine which cell types in BT co-cultures were producing IL-17 family cytokines, we performed intracellular flow cytometry for IL-17A and IL-17F with cell surface markers specific for CD4 T, CD8 T, B, NK, and NKT cells. Detection of IL-17A and IL-17F by intracellular flow cytometry requires secondary stimulation with phorbol 12-myristate 13-acetate (PMA) and ionomycin in combination with a protein transport inhibitor, such as monensin [18]. However, a limitation of this technique is that secondary stimulation causes decreased surface expression of CD4, which undermines the detection of CD4 T cells [18]. We therefore used the gating strategy shown in Figure 1, whereby CD4 T cells are detected after first gating on the total CD3+ cell population and then analyzing the cells that are negative for CD8 staining. Nearly all of the cells labeled with antibodies to IL-17A and IL-17F stained positive for CD4 (Figures 1A and 1B). Notably, a small percentage of B and NKT cells showed IL-17A and IL-17F expression (Figures 1A and IB). IL-17A or IL-17F antibodies were of the mouse IgG1, κ isotype and a mouse IgG1, κ isotype control antibody used in place of antibodies to IL-17A or IL-17F exhibited a minimal intracellular cytokine signal (Figure 1C). These data indicate that CD4 T cells are the predominant cell type that produces IL-17A and IL-17F in this model of T cell-dependent B cell responses.

We next investigated whether genes related to regulation of IL-17 family cytokines are similarly changed in CD4 T cells and B cells during BT co-culture stimulation. We performed quantitative RT-PCR with a panel of 84 probes for genes related to regulation of IL-17 cytokines on FACS-purified CD4 T cells and B cells isolated from BT co-cultures stimulated with or without α-IgM and SAg for three days. Stimulation of the BT co-cultures significantly increased levels of only 4 genes in CD4 T cells: *Il17f, Il22, Il23r,* and *Il21* (Table 2 and Table S2). Some genes specific for Th17 cells in the CD4 T cell compartment, such as *Il17a* and *Stat3* [19], were expressed but remained unchanged by stimulation. This stimulation did not increase *Cd40l* expression at 72 hours after stimulation, consistent with the transient induction in CD40L that returns to baseline levels within 24–48 hours [20]–[22]. Genes specific for other T cell subsets, including *Ifng* (Th1), *Il4* (Th2), *Il13* (Th2), and *Foxp3* (Treg), were either unchanged or significantly decreased compared to non-stimulated cells. Stimulation increased a larger number of genes in B cells, including *Ccl22, Csf2* (GM-CSF), *Il12rb, Stat4*, and *Icam1*. Although *Il17f* mRNA was elevated nearly 5-fold in B cells, consistent with the small percentage of B cells that expressed IL-17F by FACS (Figure 1B), the possibility that the detected mRNA may have originated from a small subset of contaminating T cells cannot be completely excluded. The full list of genes and expression levels is presented in Table S2. These data indicate that CD4 T cells express a Th17-like gene signature in this BT co-culture model when stimulated under conditions that elicit B cell-dependent T cell responses.

**Table 2:** CD4 T cells increase expression of several Th17-associated genes after three days of stimulation in a model of T cell-dependent B cell activation^a^.

CD4 T Cells				B Cells			
Gene Symbol	Avg. Fold Change	Std. Dev	*t*-test	Gene Symbol	Avg. Fold Change	Std. Dev.	*t*-test
IL17F	36.25	17.04	2.3E-02	CCL22	341.26	90.28	2.8E-03
IL22	28.41	2.88	7.9E-05	CSF2	124.42	63.11	2.8E-02
IL23R	3.60	1.42	3.4E-02	CCL1	105.51	83.19	9.5E-02
IL21	2.14	0.50	1.7E-02	CCL2	25.69	27.53	2.0E-01
IL5	1.45	0.74	3.6E-01	CX3CL1	15.23	4.44	5.2E-03
CCL20	1.29	1.35	7.3E-01	IL12RB2	12.89	6.15	2.9E-02
IL2	1.13	0.69	7.7E-01	STAT4	11.10	5.65	3.6E-02
IL17A	1.09	0.87	8.6E-01	ICAM1	5.63	1.22	2.8E-03
TLR4	1.00	1.71	1.0E+00	IL17F	5.10	4.26	1.7E-01
IL27	1.00	0.29	9.9E-01	CCL20	4.61	2.95	1.0E-01

a The top ten induced genes as determined by quantitative RT-PCR in CD4 T and B cell populations purified by FACS from BT co-cultures stimulated for three days with α-IgM and SAg stimulation. Gene expression values (2^−Ct^) were normalized to b-actin gene expression and presented as the fold change average ± standard deviation of cells sorted from 3 independent donor pools of BT co-cultures compared to cells isolated from non-stimulated BT co-cultures.

### IL-17A and IL-17F Proteins Are Secreted during B Cell-Dependent T Cell Activation

**Figure 2:**
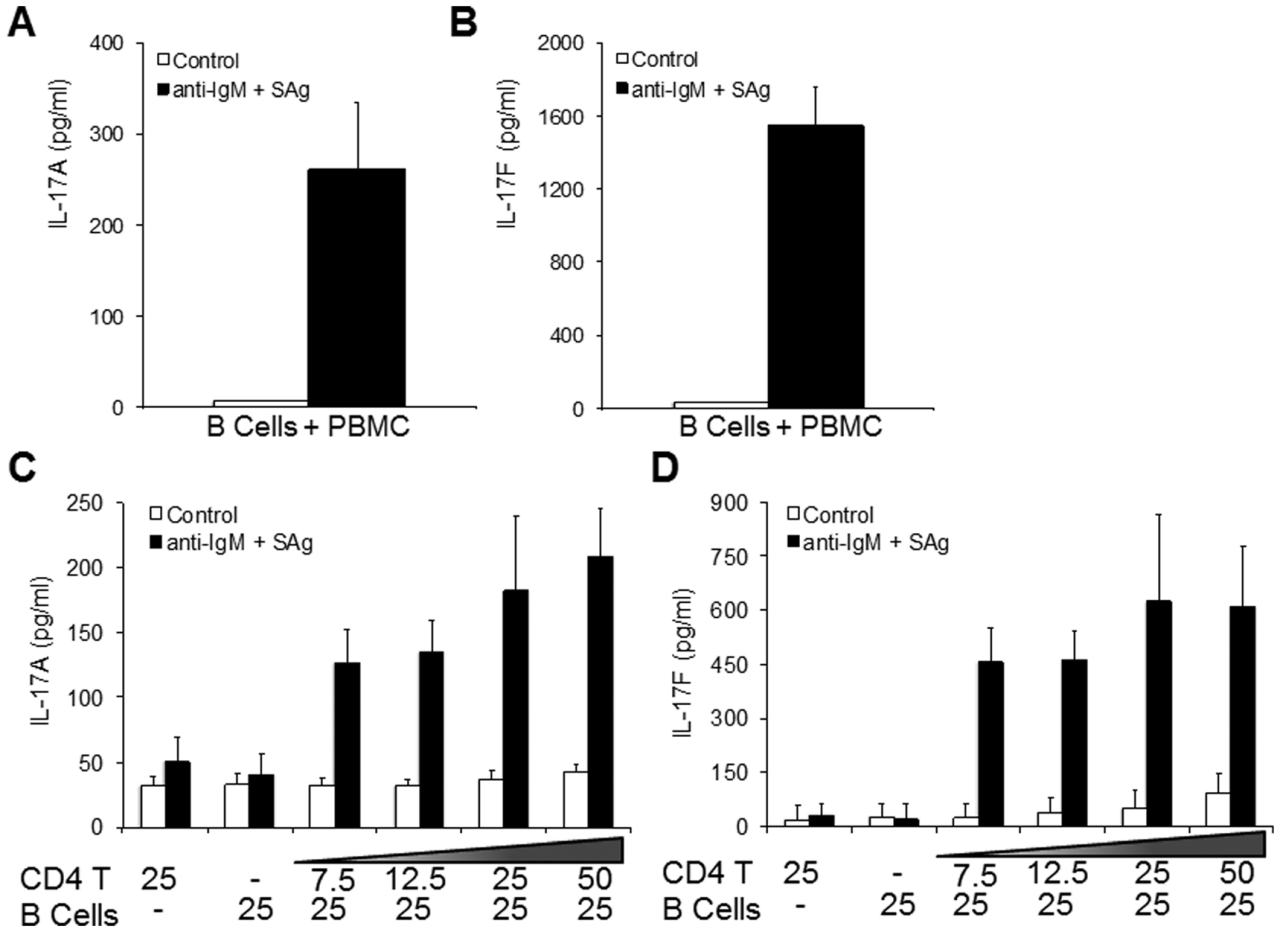
Production of IL-17A and IL-17F protein in BT co-cultures requires B cell and CD4 T cell interaction. Measurement of IL-17A (A) and IL-17F (B) by ELISA in supernatants from 25,000 B cells/well co-cultured with 25,000 PBMC/well and stimulated with α-IgM and SAg for three days. Measurement of IL-17A (C) and IL-17F (D) by ELISA in supernatants from 25,000 B cells/well and 25,000 CD4 T cells/well cultured alone or 25,000 B cells cultured with increasing numbers of CD4 T cells in the presence of α-IgM and SAg or carrier (Control) for three days. Data are the means ± standard deviation of three B cell and CD4 T cell donor pools and two independent experiments.

Although IL-17A and IL-17F were expressed in CD4 T cells by FACS analysis of stimulated BT co-cultures, IL-17F, but not IL-17A, transcripts were significantly increased at 3 days following B cell-dependent T cell activation. To determine if these proteins are secreted, we used ELISA to measure IL-17A and IL-17F in culture supernatants. BT co-culture supernatants contained substantial amounts of IL-17A (261±73 pg/ml) and IL-17F (1549±206 pg/ml) after stimulation with α-IgM and SAg for three days (Figures 2A and 2B). We then stimulated B cells and CD4 T cells, individually and in co-culture, with α-IgM and SAg to determine if production of these cytokines required the presence of both cell types. IL-17A and IL-17F production did not change when B cells or CD4 T cells were cultured alone for three days, even in the presence of stimulation (Figures 2C and 2D). However, when stimulated, B cells and CD4 T cells co-cultured at ratios from 3∶1 to 1∶2, produced between 126±26 pg/ml and 209±37 pg/ml IL-17A, and between 456±94 pg/ml and 610±170 pg/ml IL-17F, in culture supernatants (Figures 2C and 2D). IL-17A and IL-17F concentrations were modestly, although not significantly, elevated with increasing numbers of CD4 T cells. These data indicate that IL-17A and IL-17F are secreted in BT co-cultures upon stimulation with α-IgM and a low concentration of SAg.

### Regulation of IL-17A and IL-17F Production Occurs through Distinct Pathways

We next screened a panel of 144 pharmacologic modulators representing 91 different targets or pathways to determine their effects on IL-17A and IL-17F production in this co-culture system. Since small molecule pharmacologic inhibitors can often inhibit multiple targets, whenever possible multiple chemically unrelated compounds specific for the same target were used. Biologics, including cytokines and antibodies, were also used to test some mechanisms. Table S3 lists compounds used and reported mechanism(s) of action. Compounds and biologics were screened at 4 or more doses in BT co-cultures to identify those that stimulated or inhibited production of IL-17A and IL-17F, as well as IL-2, IL-6, IgG, and TNFα. T cells make IL-2, whereas B cells are the primary source for IgG and IL-6. Several cell types present in BT co-cultures produce TNFα.

These screening results suggest that a number of pathways or targets are involved in the regulation of IL-17A and IL-17F production (Table 3 and Table S4). Inhibitors of BTK (tested with PCI-32765), calcineurin (FK-506), MEK (AS703026, PD184352, UO126, and RO-5126766), p38 MAPK (AMG548 and BIRB-796), PKC (GF 109203X and AEB071), and RORγ (SR2211) reduced the levels of both IL-17A and IL-17F production, suggesting that these targets are positive regulators of both IL-17A and IL-17F. Other targets involved in general mechanisms, such as microtubule function, HDAC, HMG-CoA reductase, mitochondrial function, RNA polymerase, hsp90, and the proteasome, were also shown to be involved, as inhibitors of these targets all reduced the levels of IL-17A and IL-17F. Activators of glucocorticoid receptors (GR; tested with dexamethasone and prednisolone), RAR/RXR (trans-retinoic acid), and vitamin D receptor (calcitriol), inhibited IL-17A and IL-17F production, suggesting that these targets may function as negative regulators of IL-17A and IL-17F. Other mechanisms were identified with selective effects on IL-17A versus Il-17F. For example, mTOR (rapamycin, Torin-1, Torin-2, PP 242 and temsirolimus), PI3Kδ (CAL-101 and IC-87114), as well as IL-2R (IL-2) increased IL-17A production but did not affect IL-17F production. Modulating other targets, including JAK (CP-690,550 and INCB-018424) and PDGFR (PDGF-BB) increased IL-17F levels more than IL-17A levels, although these differences were more apparent at lower doses. These data suggest that distinct signaling pathways independently regulate IL-17A and IL17F.

**Table 3:** Summary of well-characterized compound targets that preferentially regulated production of both IL-17A and IL-17F, or had selectivity for IL-17A or IL-17F^a^.

*Inhibition of IL-17A and/or IL-17F*		
Class/Target	Number of Agents (Total in Screen)	Selectivity
BTK	1 (1)	IL-17A and IL-17F
Calcineurin	1 (1)	IL-17A and IL-17F
GR Agonist	2 (4)	IL-17A and IL-17F
JAK	2 (2)	IL-17F over IL-17A*
MEK	4 (5)	IL-17A and IL-17F
Microtubule	3 (4)	IL-17F over IL-17A*
MR Agonist	1 (1)	IL-17F over IL-17A
mTOR	5 (6)	IL-17A over IL-17F*
p38 MAPK	2 (4)	IL-17A and IL-17F
PI3Kγ	2 (2)	IL-17A over IL-17F*
PI3K/mTOR	1 (1)	IL-17A over IL-17F*
PKC	2 (3)	IL-17A and IL-17F
RORγ	1 (2)	IL-17A and IL-17F
Vitamin D Receptor Agonist	1 (1)	IL-17A and IL-17F

a Data from a screen of 144 pharmacologic modulators are summarized to show compound classes and targets that regulated production of both IL-17A and IL-17F or had selectivity for IL-17A or IL-17F in BT co-cultures. The number of agents with selectivity for a specific target is shown in parentheses next to the total number of agents in the screen for that target. Class/targets were only included if greater than 50% of agents specific for a class/target had effects on IL-17A and/or IL-17F. Agents that inhibited both IL-17A and IL-17F, or preferentially inhibited IL-17A or IL-17F had readout values less than log_10_ ratio −0.2 and were observed at 2 or more non-cytotoxic doses. *Agents against these targets selectively inhibited IL-17A or IL-17F at 2 or more relatively low doses and inhibited both IL-17A and IL-17F at 2 or more higher doses.

**Figure 3:**
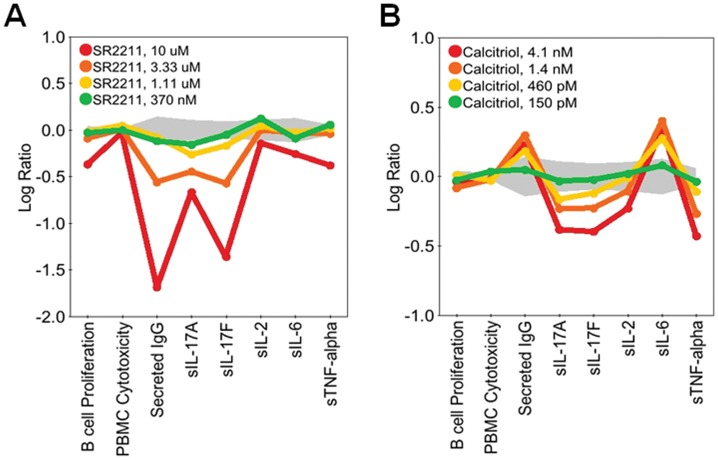
SR2211, an RORγ inverse agonist, and Calcitriol, a Vitamin D3 receptor agonist, block production of IL-17A and IL-17F in BT co-cultures. Profiles of SR2211 (0.370–10 μM; A) or Calcitriol (0.15–4.1 nM; B) added to BT co-cultures stimulated with α-IgM and SAg for three days. Parameters measured (B cell Proliferation, PBMC Cytotoxicity, Secreted IgG, sIL-17A, sIL-17F, sIL-2, sIL-6, and sTNF-alpha) are indicated along the x-axis. Data are presented as the Log_10_ ratio of drug-treated stimulated cells compared to control stimulated cells. The gray area above and below the y-axis origin indicates the 95% significance envelope for control samples based on historical data.

**Figure 4:**
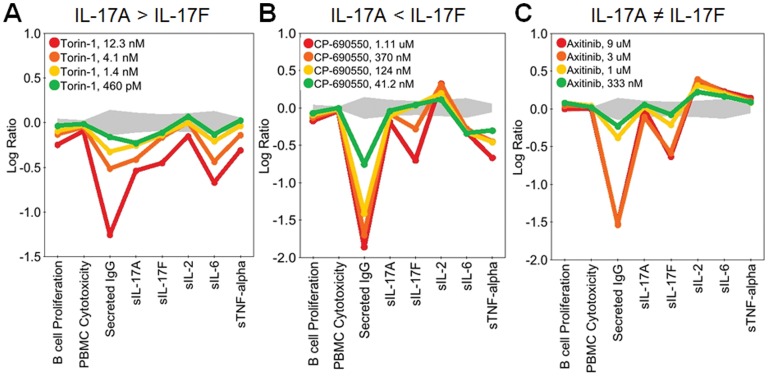
Torin-1, CP-690,550, and Axitinib are examples of compounds that regulated production of IL-17A, IL-17F or both IL-17A and IL-17F. Profiles of Torin-1 (0.460–12.3 nM; A), CP-690,550 (0.041–1.11 μM; B), or Axitinib (0.333–9 μM; C) added to BT co-cultures stimulated with α-IgM and SAg for three days. Parameters measured are indicated along the x-axis. Torin-1 inhibits IL-17A more potently than IL-17F (A), whereas CP-690,550 inhibits IL-17F more potently than IL-17A (B). Similarly, Axitinib blocks IL-17F production but does not affect IL-17A (C). Data are presented as the Log_10_ ratio of drug-treated stimulated cells compared to control stimulated cells. The gray area above and below the y-axis origin indicates the 95% significance envelope for control samples based on historical data.

**Figure 5:**
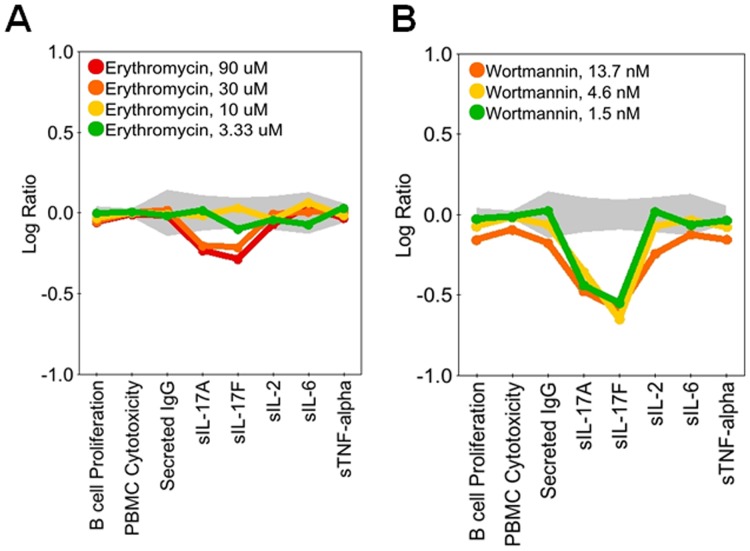
Erythromycin and Wortmannin at select doses inhibit IL-17A and IL-17F without modulating other parameters. Profiles of erythromycin (3.3–90 μM; A) or wortmannin (1.5–13.7 nM; B) added to BT co-cultures stimulated with α-IgM and SAg for three days. Erythromycin inhibited IL-17A and IL-17F production but did not affect B cell proliferation, secreted IgG, IL-2, IL-6, or TNFα, whereas wortmannin selectively inhibited IL-17A and IL-17F only at lower doses. Data are presented as the Log_10_ ratio of treated stimulated cells compared to control stimulated cells. The gray area above and below the y-axis origin indicates the 95% significance envelope for control samples based on historical data.

**Figure 6:**
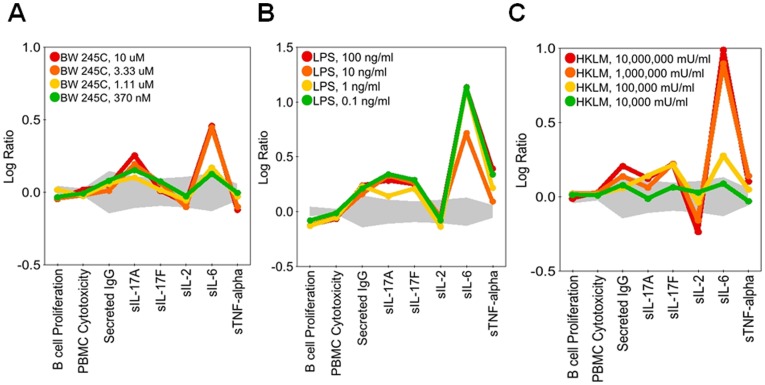
Examples of agents that potentiated production of IL-17A and IL-17F in BT co-cultures. Profiles of BW 245C (0.37–10 μM; A), LPS (0.1–100 ng/ml; B), or HKLM (10–10,000 U/ml; C) added to BT co-cultures stimulated with α-IgM and SAg for three days. BW245C and LPS enhance IL-17A and IL-6 production, while LPS also stimulates IL-17F, secreted IgG, IL-6, and TNFα. HKLM stimulates IL-17F more potently than IL-17A. Data are presented as the log_10_ ratio of treated stimulated cells compared to control stimulated cells. The gray area above and below the y-axis origin indicates the 95% significance envelope for control samples based on historical data.

**Figure 7:**
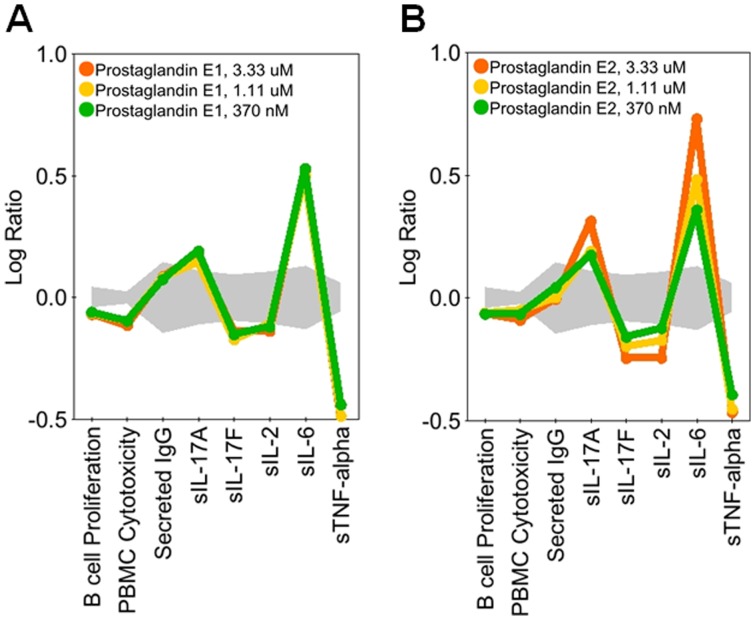
PGE1 and PGE2 stimulate production of IL-17A and impair production of IL-17F. Profiles of PGE1 (0.37–3.33 μM; A) and PGE2 (0.37–3.33 μM; B) added to BT co-cultures stimulated with α-IgM and SAg for three days. PGE1 and PGE2 increased production of IL-17A and IL-6, but inhibited IL-17F, IL-2, and TNFα production. Data are presented as the Log_10_ ratio of treated stimulated cells compared to control stimulated cells. The gray area above and below the y-axis origin indicates the 95% significance envelope for control samples based on historical data.

The diversity of patterns observed in the regulation of IL-17A and IL-17F are illustrated in Figures 3, 4, 5, 6, and 7. In Figure 3, SR2211, an RORγ-selective inverse agonist (0.37–10 μM), and 1,25-dihydroxyvitamin D_3_ (calcitriol), a vitamin D receptor agonist (0.15–4.1 nM), significantly decreased IL-17A and IL-17F production in the BT co-culture system, consistent with the reported effects of these agents in CD4 T cells [23], [24]. Calcitriol also increased expression of IL-6 and secretion of IgG, whereas SR2211 decreased production of IgG. Torin-1, an mTOR inhibitor, decreased IL-17A production to a greater extent than IL-17F at lower concentrations, whereas CP-690,550 (tofacitinib), a JAK inhibitor, preferentially blocked production of IL-17F over IL-17A (Figures 4A and 4B). Axitinib, a small-molecule tyrosine kinase inhibitor with selectivity for VEGFR, diminished production of IL-17F without affecting IL-17A production (Figure 4C). While this result with axitinib is intriguing, the effective doses are high (3 and 9 μM), raising the possibility that this activity may be unrelated to the primary targets of this compound. Both CP-690,550 and axitinib, which impacted IL-17F more than IL-17A, stimulated production of IL-2 at multiple concentrations. However, CP-690,550, but not axitinib, inhibited IL-6 and TNFα (Figure 4C). Interestingly, two compounds, erythromycin (3.3 μM–90 μM) and wortmannin (1.5 nM–13.7 nM), regulated IL-17A and IL-17F at concentrations that did not affect IgG, IL-2, IL-6 or TNFα (Figures 5A and 5B); higher levels of wortmannin decreased IgG, IL-2, IL-6, and TNFα production and inhibited B cell proliferation (Figure 5B).

**Figure 8:**
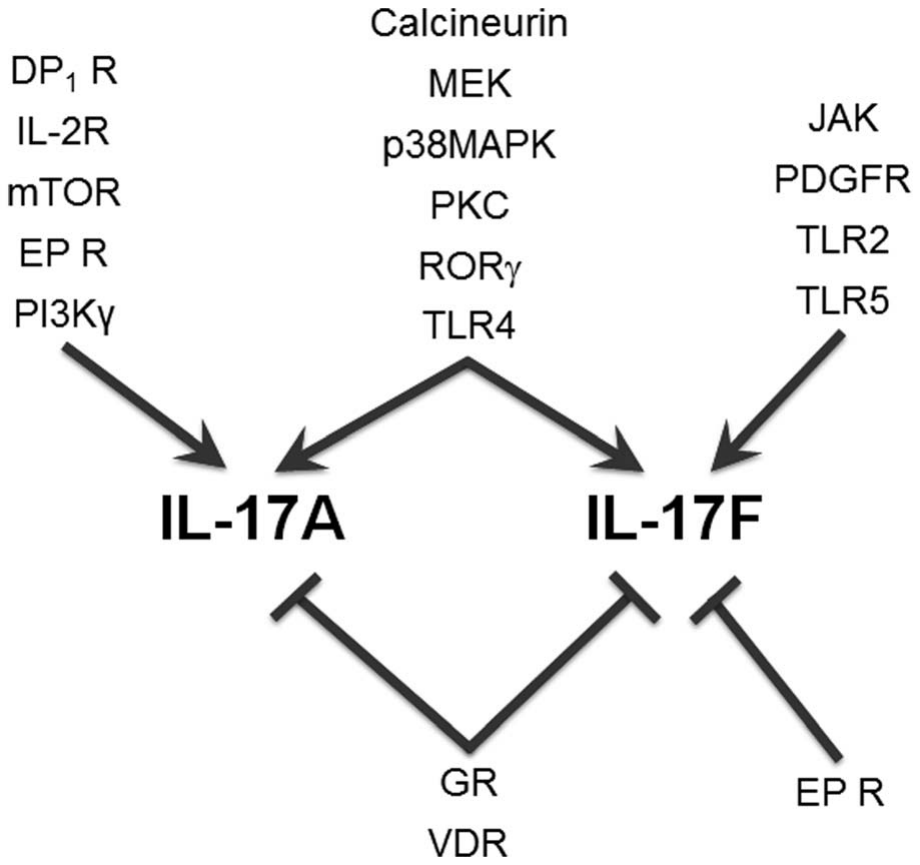
Summary of pathways and targets identified in a screen of pharmacologic modulators that positively or negatively regulate IL-17A and/or IL-17F in stimulated BT co-cultures. Pathways and targets are listed next to arrows that indicate selectivity for IL-17A, IL-17A and IL-17F, or IL-17F.

We identified 9 agents that stimulated IL-17A or IL-17F production at two or more doses (Table 4 and Table S4). LPS, a TLR4 ligand, increased IL-17A, IL-17F, IL-6, IgG, and TNFα, whereas BW 245C, a DP_1_ receptor agonist, selectively stimulated IL-17A and IL-6 production (Figures 6A and 6B). HKLM, a TLR2 ligand, more effectively enhanced IL-17F production than IL-17A (Figure 6C). FSL-1 and flagellin, TLR6/2 and TLR5 ligands, respectively, also increased IL-17F production at two doses, but other TLR ligands in the screen, including imiquimod, ODN2006, PAM3CSK4, Poly(I∶C), and ssRNA40 did not enhance production of IL-17A or IL-17F at more than one dose (Tables S3 and S4). IL-2 increased IL-17A at lower doses but not at higher doses (Tables S3 and S4). Interestingly, two agents, prostaglandin E_1_ (PGE_1_) and PGE_2_ (EP receptor ligands), stimulated IL-17A production but inhibited IL-17F (Figures 7A and 7B). Together these results show that although many pathways are involved in the regulation of IL-17A and IL-17F production in BT co-cultures, only a few pathways are involved in the differential regulation of IL-17A versus IL-17F (Figure 8).

**Table 4:** Summary of compound targets that stimulated production of IL-17A and/or IL-17F^a^.

*Stimulation of IL-17A and/or IL-17F*				
Class/Target	Agent(s)	Number of Agents (Total in Screen)	Selectivity	
DP Agonist	BW 245C	1 (1)	IL-17A	
EP Agonist	Iloprost, PGE1, PGE2	3 (3)	IL-17A	
IL-2R Ligand	IL-2	1 (1)	IL-17A and IL-17F	
TLR2	HKLM	1 (1)	IL-17F	
TLR2/6	FSL-1	1 (1)	IL-17A and IL-17F	
TLR4	LPS	1 (1)	IL-17A and IL-17F	
TLR5	Flagellin	1 (1)	IL-17F	

a Data from the screen summarized to show compound targets that increased production of both IL-17A or IL-17F, or had selectivity for IL-17A or IL-17F in BT co-cultures. Effects of agents on IL-17A and/or IL-17F had readout values greater than log_10_ ratio 0.15 and were observed at 2 or more non-cytotoxic doses. The number of agents with selectivity for a specific target is shown in parentheses next to the total number of agents in the screen for that target.

## Discussion

In this study, we report that regulation of IL-17A occurs through pathways distinct from those that regulate IL-17F in a primary human cell-based model of T cell-dependent B cell activation (BioMAP® BT system). We show that significant amounts of IL-17A and IL-17F are produced and secreted by CD4 T cells in these stimulated BT co-cultures and that both B and T cells are required for optimal production of IL-17 cytokines. Previous studies examining the role of Th17 cells in B cell activation focused on either the activity of Th17 cells or IL-17A and IL-17F cytokines on B cell function [4], [7]. Here, we evaluate the reciprocal situation by investigating the effects of B cell activation on T cell function and the production of IL-17A and IL-17F. We found that B cells activated by α-IgM in the presence of peripheral blood CD4 T cells, primed with non-mitogenic concentrations of superantigens to induce TCR stimulation, contribute to the polarization of CD4 T cells to a Th17-like phenotype. These findings suggest that B cell regulation of the production of IL-17A and IL-17F by CD4 T cells during B cell-dependent T cell activation may be an important event for biological processes where IL-17 and Th17 cells play a role, such as in autoimmune diseases and GC formation.

Several studies in transgenic mice have examined the role of IL-17 family cytokines in B cell activation and subsequent formation of GC in vivo. IL-17A-deficient mice show impaired antigen-specific Ig production upon immunization compared to normal control mice, although the deficient mice still form normal GCs [25], [26]. In contrast, mice deficient in IL-17 receptor A (IL-17RA) have both decreased Ig production and fewer GCs. Because IL-17A and IL-17F signal through the IL-17RA and IL-17RC heterodimer [27], and IL-25 signals through a IL-17RA and IL-17RB heterodimer, together these studies suggest that IL-17F and/or IL-25 may be important for the creation of GCs. In addition, autoimmune mice on the BXD2 background express elevated levels of IL-17A and spontaneously develop GCs before the formation of pathogenic autoantibodies, further supporting a role for IL-17A in B cell responses [28]. Our findings here in primary human cells also support a model where human B cells polarize CD4 T cells to produce IL-17A and IL-17F during B-T cell activation.

Our data and the data of others indicate that CD4 T cells are the predominant source of IL-17A and IL-17F in the BT co-cultures, but other cell types may have also contributed to amounts measured in culture supernatants [29], [30]. We observed that increased amounts of IL-17A and IL-17F were produced in B cell and PBMC (BT) co-cultures compared to B cell and purified CD4 T cell co-cultures. This difference may be explained by the production of these cytokines by additional cell types within the PBMC fraction or by the presence of additional APC that enhanced IL-17A and IL-17F production by CD4 T cells. B and NKT cells expressed IL-17A and IL-17F as measured by intracellular FACS, and although these cytokines were only expressed by a small percentage of these cells, their production of IL-17A could be sufficient to explain the discrepancy observed between amounts measured in supernatants and the lack of significantly increased *Il17a* mRNA in purified CD4 T cells. Importantly, this finding is in line with previous work showing that human B cells are capable of producing IL-17A and IL-17F [31]. While α-IgM + SAg stimulation slightly decreased high expression levels of *Rorc* mRNA in CD4 T cells, this finding is consistent with reports that IL-17A and IL-17F can be independently regulated from *Rorc* [32], [33]. We also cannot exclude the possibility that *Rorc* or *Il17a* were regulated on a time course not examined in this study. CD4-CD8- T cells, which are known to produce IL-17A under some conditions, may have also contributed to the production of IL-17A or IL-17F measured in culture supernatants [34]. Future work should focus on further characterizing the factors produced by cell types stimulated in the context of BT co-culture to elucidate how B cells induce the polarization of CD4 T cells to a Th17-like phenotype.

A surprising number of genes related to Th17 biology were up regulated in B cells after BT co-culture and stimulation with α-IgM + SAg. To our knowledge, these B cell genes have not been previously implicated in B cell regulation of Th17 differentiation. For example, *Ccl22*, the most highly induced gene in B cells isolated from BT co-cultures, is typically produced by macrophages and dendritic cells and is found at elevated concentrations in the CNS of multiple sclerosis (MS) patients [35]. The prominent role of Th17 cells in MS suggests further work should explore the role of B cell-produced CCL22 in Th17 development and autoimmune disease. *CSF2* (GM-CSF), the second most highly induced Th17-related gene, was recently reported to mark a novel B cell subset critical for innate immune responses [36]. In light of our findings here, certain B cell populations may also prove to be a significant source of GM-CSF in the pathogenesis of autoimmune disease.

As an initial step to identify pathways important in the B cell regulation of IL-17A and IL-17F production by T cells, we screened a broad panel of diverse pharmacologic agents. Regulation of IL-17A and IL-17F production by CD4 T cells has been both expected and observed to occur predominantly through shared pathways due to the proximity of the IL-17A and IL-17F genes on chromosome 6 and parallel H3 histone hyperacetylation at multiple conserved noncoding sequence sites within the IL-17A-IL-17F locus [37]. Previous reports in mice suggest some differences, as IL-17A production by CD4 T cells was shown to require maximal TCR stimulation, whereas IL-17F was found to be independent of Itk and PLCγ activation [32]. Another study demonstrated that certain CD4 T cell populations produce IL-17F independent of IL-17A [38], perhaps reflecting temporal differences in the synthesis of IL-17F and IL-17A in developing Th17 cells [39]. Moreover, increased CREMα expression in T cells isolated from SLE patients results in decreased IL-17F expression but not IL-17A [40].

CP-690,550, the same JAK inhibitor used in our screen, has been shown to block IL-17A and IL-17F production when Th17 cells are differentiated in the presence of IL-6 and IL-23, but to enhance IL-17A and have no effect on IL-17F when TGFβ is added to the differentiation media [41]. In our co-culture system, CP-690,550 preferentially inhibits IL-17F over IL-17A, suggesting that TGFβ is not a regulator of the production of IL-17A or IL-17F in our conditions. Indeed, LY2157299, a TGFβ Receptor I inhibitor [42], had no effect on production of IL-17A or IL-17F in our system. Thus, in contrast to in vitro models of Th17 differentiation that use a combination of IL-1β, IL-6, IL-23 or TGFβ to drive differentiation [13], [19], CD4 T cells in our BT co-cultures, polarize to a Th17-like phenotype without the addition of exogenous cytokines. This difference highlights the physiological relevance of our approach.

We here identify novel pharmacologic agents that regulate IL-17A or IL-17F production. Several microtubule inhibitors, including paclitaxel, epothilone B, and picropodophyllin, all preferentially inhibited IL-17F over IL-17A, but did not impair production of IL-2 or have significant cytotoxic effects at the doses tested. Interestingly, we found that erythromycin, a macrolide antibiotic, blocked IL-17A and IL-17F without affecting B cell proliferation or production of IgG, IL-2, IL-6, or TNFα in stimulated BT co-cultures. Erythromycin inhibits NF-κB signaling in T cells, which may account for the effect of this antibiotic on IL-17A and IL-17F [43]. Although these previously unidentified pathways regulate IL-17A and IL-17F production in stimulated BT co-cultures, it remains to be seen how these pathways contribute to CD4 T cell production of IL-17A or IL-17F when stimulated through activation by dendritic cells or other cell types.

In this study we present data that indicates primary human B cells contribute to the polarization of CD4 T cells toward a Th17 phenotype in a model of T cell-dependent B cell activation. This finding adds to a growing body of evidence implicating IL-17A and/or IL-17F production by CD4 T cells in the development of antibody-mediated immune responses and the formation of GCs. Understanding the cellular interactions and signaling processes that govern how B cell responses are regulated and GCs are formed should benefit the development of therapeutics for autoimmune diseases. The approach described here utilizing a T cell-dependent model of B cell activation that also reflects B cell-dependent differentiation of Th17 cells provides a useful screening system for the identification of targets, pathways, and small molecule inhibitors that selectively act on IL-17A or IL-17F production. The importance of selectively targeting IL-17A or IL-17F *in vivo* is not well understood, but the compounds and agents identified here with activity on IL-17A versus IL-17F should help address this question.

## Supporting Information

Table S1 **Microarray data set for genes significantly increased or decreased (** ***P*** **≤0.05) in stimulated BT co-cultures.** Microarray analysis in BT co-cultures stimulated with α-IgM and SAg for three days compared to the same cells co-cultured for three days without stimulation. Means and standard deviations are from 3 independent replicates with 3 different donor pools.
(DOC)

Table S2 **Quantitative RT-PCR Th17 Array data set for purified CD4 T cells and B cells isolated from stimulated and non-stimulated BT co-cultures.** Quantitative RT-PCR gene expression data from CD4 T and B cell populations purified by FACS after three days in co-culture with α-IgM and SAg stimulation. Data normalized to b-actin expression levels and presented as fold change (above) and raw Ct values (below) are shown. Gene expression data are from 3 independent donor pools of stimulated BT co-cultures compared to cells isolated from non-stimulated BT co-cultures.
(DOC)

Table S3 **Screen of agents in stimulated BT co-cultures.** Agents screened are listed with the concentrations tested, supplier, and putative mechanism of action. Data for each agent screened on B cell proliferation, PBMC cytotoxicity, IgG, IL-17A, IL-17F, IL-2, IL-6 and TNFα. Data are presented as the Log_10_ ratio of values from agent-treated stimulated BT co-cultures to values from control stimulated BT co-cultures.
(DOC)

Table S4 **Lists of agents and their concentrations that regulated IL-17A and/or IL-17F in stimulated in BT co-cultures.** Agents are listed that decreased both IL-17A and IL-17F, decreased IL-17A but not IL-17F, decreased IL-17F but not IL-17A, and increased either IL-17A or IL-17F. Agents were only included if they inhibited readouts greater than Log_10_ ratio −0.2 or increased readouts greater than Log_10_ ratio −0.15 at 2 or more non-cytotoxic concentrations.
(DOC)
