# Supplementary material for: Regulation of IL-17A Production Is Distinct from IL-17F in a Primary Human Cell Co-culture Model of T Cell-Mediated B Cell Activation
Source: PLoS One. 2013 Mar 7;8(3):e58966. doi: 10.1371/journal.pone.0058966 (PMC3591360; doi:10.1371/journal.pone.0058966)
Supplement: Table S1 — Microarray data set for genes significantly increased or decreased ( P ≤0.05) in stimulated BT co-cultures. Microarray analysis in BT co-cultures stimulated with α-IgM and SAg for three days compared to the same cells co-cultured for three days without stimulation. Means and standard deviations are from 3 independent replicates with 3 different donor pools. (DOC) [file pone.0058966.s001.doc]

**Table S1. Microarray data set for genes significantly increased or decreased (*P* ≤ 0.05) in stimulated BT co-cultures.** Microarray analysis in BT co-cultures stimulated with α-IgM and SAg for three days compared to the same cells co-cultured for three days without stimulation. Means and standard deviations are from 3 independent replicates with 3 different donor pools.

| Gene Symbol | Stim B/PBMC Mean | Stim B/PBMC Std. Dev. | Control B/PBMC Mean | Control B/PBMC Std. Dev. | Absolute t value | *P* value | FDR | Fold Change |
| --- | --- | --- | --- | --- | --- | --- | --- | --- |
| IL17F | 1907.42 | 615.09 | 121.08 | 12.25 | 4.94 | 0.039 | 0.446 | 15.75 |
| HS.579631 | 2203.00 | 557.56 | 311.83 | 13.39 | 5.75 | 0.029 | 0.445 | 7.06 |
| FASN | 4307.95 | 1441.34 | 661.21 | 407.94 | 6.02 | 0.027 | 0.448 | 6.52 |
| CD1C | 690.70 | 196.96 | 127.35 | 2.98 | 4.98 | 0.038 | 0.447 | 5.42 |
| KIAA0101 | 1622.11 | 708.82 | 311.46 | 208.69 | 4.42 | 0.048 | 0.450 | 5.21 |
| TYMS | 2824.15 | 986.70 | 543.22 | 426.99 | 6.93 | 0.020 | 0.447 | 5.20 |
| UHRF1 | 3838.29 | 1639.18 | 807.90 | 748.14 | 4.90 | 0.039 | 0.444 | 4.75 |
| AURKB | 1327.24 | 477.43 | 289.34 | 197.83 | 6.38 | 0.024 | 0.453 | 4.59 |
| TOP2A | 1779.72 | 739.06 | 399.46 | 324.73 | 5.74 | 0.029 | 0.445 | 4.46 |
| SCD | 2975.46 | 1321.33 | 669.67 | 424.97 | 4.42 | 0.048 | 0.450 | 4.44 |
| TMEM97 | 2374.47 | 1031.99 | 536.84 | 414.81 | 5.16 | 0.036 | 0.446 | 4.42 |
| GINS2 | 1801.07 | 654.78 | 408.66 | 288.39 | 6.55 | 0.023 | 0.453 | 4.41 |
| CKS2 | 4601.83 | 1768.30 | 1063.73 | 845.74 | 6.63 | 0.022 | 0.453 | 4.33 |
| MELK | 1486.75 | 603.77 | 347.32 | 272.29 | 5.90 | 0.028 | 0.447 | 4.28 |
| UBE2C | 2130.76 | 1011.08 | 503.73 | 491.55 | 5.01 | 0.038 | 0.447 | 4.23 |
| CKS2 | 2179.62 | 1010.11 | 516.51 | 392.92 | 4.44 | 0.047 | 0.450 | 4.22 |
| CD70 | 1168.04 | 275.32 | 281.51 | 99.39 | 7.22 | 0.019 | 0.450 | 4.15 |
| MCM4 | 1612.83 | 693.69 | 393.89 | 284.33 | 5.01 | 0.038 | 0.446 | 4.09 |
| EXO1 | 806.92 | 241.28 | 198.37 | 110.80 | 7.80 | 0.016 | 0.449 | 4.07 |
| RPS24 | 724.23 | 279.88 | 185.70 | 79.37 | 4.65 | 0.043 | 0.447 | 3.90 |
| CDCA8 | 792.77 | 295.91 | 206.10 | 77.09 | 4.61 | 0.044 | 0.447 | 3.85 |
| LOC643300 | 928.50 | 312.40 | 243.71 | 116.17 | 5.46 | 0.032 | 0.445 | 3.81 |
| NPW | 707.97 | 160.42 | 185.83 | 52.94 | 8.35 | 0.014 | 0.452 | 3.81 |
| TPX2 | 733.12 | 202.49 | 192.68 | 64.02 | 6.59 | 0.022 | 0.454 | 3.80 |
| SQLE | 3447.85 | 1338.17 | 910.39 | 564.78 | 4.98 | 0.038 | 0.446 | 3.79 |
| EZH2 | 701.32 | 252.27 | 187.30 | 54.10 | 4.47 | 0.047 | 0.449 | 3.74 |
| HMMR | 1702.09 | 818.10 | 458.21 | 446.02 | 5.13 | 0.036 | 0.447 | 3.71 |
| CTPS | 1663.68 | 416.42 | 448.60 | 254.45 | 12.40 | 0.006 | 0.444 | 3.71 |
| CENPM | 1007.74 | 318.68 | 273.94 | 174.28 | 8.60 | 0.013 | 0.450 | 3.68 |
| HJURP | 609.35 | 208.59 | 166.90 | 54.95 | 4.99 | 0.038 | 0.447 | 3.65 |
| MCM2 | 1695.90 | 747.71 | 476.55 | 382.33 | 5.16 | 0.036 | 0.447 | 3.56 |
| FEN1 | 3340.77 | 1142.98 | 941.58 | 668.19 | 7.29 | 0.018 | 0.450 | 3.55 |
| LOC387882 | 1145.07 | 432.02 | 323.37 | 213.70 | 5.58 | 0.031 | 0.445 | 3.54 |
| C16ORF33 | 2114.67 | 498.24 | 603.18 | 345.42 | 15.45 | 0.004 | 0.451 | 3.51 |
| MCM10 | 946.49 | 405.80 | 270.61 | 188.90 | 5.30 | 0.034 | 0.447 | 3.50 |
| MGC40489 | 1042.77 | 314.92 | 299.23 | 99.43 | 5.84 | 0.028 | 0.446 | 3.48 |
| CDC25A | 646.75 | 252.43 | 185.89 | 104.33 | 4.93 | 0.039 | 0.446 | 3.48 |
| NOLC1 | 1093.31 | 320.06 | 316.05 | 178.07 | 9.38 | 0.011 | 0.462 | 3.46 |
| CCNF | 976.39 | 377.50 | 282.86 | 182.47 | 6.11 | 0.026 | 0.450 | 3.45 |
| PAICS | 5202.45 | 1891.04 | 1515.78 | 1093.15 | 7.86 | 0.016 | 0.451 | 3.43 |
| PBK | 554.09 | 219.73 | 161.82 | 76.24 | 4.72 | 0.042 | 0.445 | 3.42 |
| PPP1R14A | 558.84 | 137.89 | 164.27 | 28.60 | 6.25 | 0.025 | 0.449 | 3.40 |
| SQLE | 845.79 | 286.85 | 248.86 | 49.57 | 4.35 | 0.049 | 0.452 | 3.40 |
| KPNA2 | 1163.47 | 408.81 | 343.78 | 193.51 | 5.71 | 0.029 | 0.445 | 3.38 |
| TFDP1 | 2281.45 | 649.03 | 677.12 | 350.43 | 8.93 | 0.012 | 0.455 | 3.37 |
| PSAT1 | 859.80 | 271.54 | 256.56 | 100.95 | 6.02 | 0.027 | 0.448 | 3.35 |
| DDIT4 | 1771.15 | 915.03 | 533.21 | 432.94 | 4.44 | 0.047 | 0.450 | 3.32 |
| MCM6 | 5854.16 | 2483.96 | 1776.93 | 1478.82 | 6.08 | 0.026 | 0.449 | 3.29 |
| NPM3 | 1480.79 | 485.43 | 450.72 | 260.15 | 7.92 | 0.016 | 0.452 | 3.29 |
| LOC100128266 | 3954.42 | 610.23 | 1205.65 | 727.40 | 6.98 | 0.020 | 0.448 | 3.28 |
| HIST1H4C | 14326.73 | 967.53 | 4385.68 | 160.22 | 19.24 | 0.003 | 0.452 | 3.27 |
| CDKN3 | 629.84 | 239.74 | 193.20 | 88.51 | 4.95 | 0.038 | 0.446 | 3.26 |
| GAR1 | 3567.59 | 1405.62 | 1095.22 | 642.57 | 5.42 | 0.032 | 0.445 | 3.26 |
| TNFSF4 | 561.12 | 148.43 | 173.40 | 34.24 | 5.32 | 0.034 | 0.447 | 3.24 |
| TMEM106C | 1386.57 | 455.73 | 428.69 | 235.74 | 7.23 | 0.019 | 0.450 | 3.23 |
| LOC642956 | 2266.20 | 718.22 | 704.63 | 512.28 | 5.97 | 0.027 | 0.447 | 3.22 |
| CCT2 | 3335.03 | 1056.05 | 1041.26 | 461.95 | 6.66 | 0.022 | 0.452 | 3.20 |
| LOC729816 | 1668.20 | 594.11 | 523.18 | 376.31 | 7.28 | 0.018 | 0.449 | 3.19 |
| PSMG1 | 2379.32 | 988.31 | 747.23 | 402.44 | 4.78 | 0.041 | 0.444 | 3.18 |
| CCDC34 | 881.47 | 360.98 | 277.33 | 138.20 | 4.68 | 0.043 | 0.447 | 3.18 |
| ZWILCH | 1320.76 | 543.11 | 417.62 | 207.35 | 4.61 | 0.044 | 0.447 | 3.16 |
| LOC731049 | 3187.71 | 1044.50 | 1012.82 | 450.20 | 6.08 | 0.026 | 0.449 | 3.15 |
| KIF23 | 595.35 | 219.40 | 189.60 | 57.92 | 4.33 | 0.049 | 0.452 | 3.14 |
| UCK2 | 696.50 | 99.74 | 221.84 | 74.01 | 30.64 | 0.001 | 0.453 | 3.14 |
| CCND2 | 5780.40 | 1017.99 | 1843.47 | 1443.75 | 9.39 | 0.011 | 0.462 | 3.14 |
| WDR12 | 1346.87 | 587.85 | 432.77 | 253.56 | 4.66 | 0.043 | 0.448 | 3.11 |
| UBE2T | 1058.93 | 442.38 | 340.78 | 188.20 | 4.89 | 0.039 | 0.444 | 3.11 |
| SLC7A5 | 1730.87 | 786.91 | 558.68 | 329.24 | 4.40 | 0.048 | 0.450 | 3.10 |
| DCUN1D5 | 2264.12 | 953.72 | 732.95 | 353.63 | 4.40 | 0.048 | 0.450 | 3.09 |
| LTA | 11886.67 | 4258.68 | 3851.02 | 3303.59 | 5.90 | 0.028 | 0.447 | 3.09 |
| PFKM | 785.04 | 181.89 | 254.37 | 67.76 | 7.16 | 0.019 | 0.451 | 3.09 |
| CCDC34 | 601.75 | 200.26 | 195.37 | 121.14 | 8.85 | 0.013 | 0.457 | 3.08 |
| SLBP | 1844.31 | 590.19 | 599.93 | 245.35 | 5.93 | 0.027 | 0.447 | 3.07 |
| GMPS | 1110.79 | 409.04 | 364.00 | 133.33 | 4.69 | 0.043 | 0.447 | 3.05 |
| EZH2 | 580.47 | 237.87 | 190.81 | 86.91 | 4.37 | 0.049 | 0.451 | 3.04 |
| LOC100128266 | 5408.97 | 2453.09 | 1784.84 | 1144.24 | 4.72 | 0.042 | 0.445 | 3.03 |
| C1QBP | 4844.69 | 1715.56 | 1610.17 | 835.74 | 5.03 | 0.037 | 0.447 | 3.01 |
| RCAN1 | 1457.99 | 593.32 | 485.92 | 205.42 | 4.34 | 0.049 | 0.452 | 3.00 |
| HOMER2 | 518.96 | 71.41 | 173.16 | 41.05 | 15.87 | 0.004 | 0.453 | 3.00 |
| EEF1E1 | 2440.12 | 1048.48 | 815.89 | 519.83 | 5.14 | 0.036 | 0.447 | 2.99 |
| CCL17 | 1515.19 | 116.62 | 507.23 | 499.10 | 4.32 | 0.050 | 0.452 | 2.99 |
| RAN | 9716.93 | 2172.09 | 3255.81 | 1264.66 | 12.28 | 0.007 | 0.440 | 2.98 |
| CDT1 | 596.51 | 228.11 | 200.05 | 118.88 | 5.57 | 0.031 | 0.445 | 2.98 |
| UCHL5 | 1596.83 | 665.04 | 536.08 | 298.31 | 5.01 | 0.038 | 0.447 | 2.98 |
| NP | 2461.48 | 857.28 | 826.79 | 488.94 | 7.36 | 0.018 | 0.449 | 2.98 |
| C17ORF53 | 575.26 | 194.76 | 193.33 | 60.61 | 4.81 | 0.041 | 0.444 | 2.98 |
| CCDC86 | 1262.54 | 468.57 | 424.46 | 154.24 | 4.44 | 0.047 | 0.450 | 2.97 |
| MCM7 | 5408.92 | 2069.93 | 1837.63 | 1298.02 | 7.18 | 0.019 | 0.450 | 2.94 |
| THOC4 | 1297.67 | 483.00 | 441.12 | 166.65 | 4.65 | 0.043 | 0.447 | 2.94 |
| DUT | 960.19 | 405.00 | 326.57 | 225.67 | 5.90 | 0.028 | 0.448 | 2.94 |
| HSPC111 | 1094.13 | 301.29 | 372.63 | 167.36 | 9.23 | 0.012 | 0.456 | 2.94 |
| LOC728564 | 2079.04 | 854.04 | 710.76 | 386.21 | 4.89 | 0.039 | 0.444 | 2.93 |
| BATF | 1047.03 | 457.35 | 358.62 | 218.79 | 4.99 | 0.038 | 0.447 | 2.92 |
| LOC729423 | 1405.91 | 178.20 | 483.09 | 85.07 | 16.49 | 0.004 | 0.457 | 2.91 |
| LOC731314 | 1513.85 | 365.24 | 520.64 | 273.62 | 18.30 | 0.003 | 0.455 | 2.91 |
| SMS | 2969.99 | 800.62 | 1021.58 | 362.36 | 7.59 | 0.017 | 0.444 | 2.91 |
| KIF2C | 439.41 | 162.91 | 152.14 | 48.39 | 4.34 | 0.049 | 0.452 | 2.89 |
| PTGER4 | 2061.92 | 678.23 | 714.79 | 328.04 | 6.46 | 0.023 | 0.453 | 2.88 |
| DDX21 | 4504.36 | 1714.49 | 1572.85 | 833.04 | 5.73 | 0.029 | 0.446 | 2.86 |
| DCTPP1 | 2372.17 | 959.87 | 829.39 | 443.83 | 4.46 | 0.047 | 0.449 | 2.86 |
| LOC651816 | 779.59 | 125.45 | 273.21 | 28.59 | 9.05 | 0.012 | 0.455 | 2.85 |
| ABCB10 | 1139.63 | 459.53 | 399.73 | 164.45 | 4.32 | 0.050 | 0.452 | 2.85 |
| PGAM1 | 2147.43 | 469.04 | 753.29 | 201.86 | 9.01 | 0.012 | 0.454 | 2.85 |
| CCT3 | 2410.46 | 808.72 | 849.03 | 322.79 | 5.47 | 0.032 | 0.445 | 2.84 |
| SLC35F2 | 814.56 | 259.48 | 287.28 | 132.43 | 7.05 | 0.020 | 0.449 | 2.84 |
| TFAM | 1963.66 | 797.96 | 692.62 | 296.92 | 4.38 | 0.048 | 0.451 | 2.84 |
| MRPS17 | 1150.60 | 405.23 | 408.51 | 224.57 | 7.11 | 0.019 | 0.449 | 2.82 |
| HMGCS1 | 2058.20 | 755.79 | 731.22 | 390.79 | 6.02 | 0.027 | 0.449 | 2.81 |
| IARS | 2065.71 | 857.65 | 743.47 | 335.99 | 4.38 | 0.048 | 0.451 | 2.78 |
| LOC649553 | 4719.32 | 1621.31 | 1702.57 | 802.84 | 6.19 | 0.025 | 0.449 | 2.77 |
| GLO1 | 2113.48 | 729.74 | 763.77 | 217.75 | 4.55 | 0.045 | 0.448 | 2.77 |
| LOC399804 | 8932.91 | 3338.01 | 3231.85 | 1811.89 | 6.32 | 0.024 | 0.451 | 2.76 |
| PDCD2L | 691.78 | 194.17 | 250.71 | 74.63 | 6.36 | 0.024 | 0.452 | 2.76 |
| LOC400013 | 2844.21 | 1083.33 | 1030.95 | 478.70 | 5.16 | 0.036 | 0.447 | 2.76 |
| NCL | 1925.85 | 629.01 | 699.51 | 268.33 | 5.65 | 0.030 | 0.446 | 2.75 |
| CXORF64 | 487.80 | 87.75 | 177.55 | 26.01 | 7.72 | 0.016 | 0.449 | 2.75 |
| PFAS | 1451.80 | 601.82 | 529.20 | 339.94 | 5.61 | 0.030 | 0.445 | 2.74 |
| RRM1 | 3717.17 | 1694.97 | 1356.77 | 949.48 | 4.61 | 0.044 | 0.448 | 2.74 |
| RRAS2 | 787.67 | 226.36 | 287.91 | 70.67 | 5.39 | 0.033 | 0.445 | 2.74 |
| TUBB | 2539.20 | 321.33 | 928.39 | 267.61 | 12.89 | 0.006 | 0.438 | 2.74 |
| RPLP0 | 966.98 | 264.07 | 354.40 | 73.16 | 5.55 | 0.031 | 0.445 | 2.73 |
| CKS1B | 1191.77 | 528.48 | 436.84 | 255.65 | 4.56 | 0.045 | 0.448 | 2.73 |
| FABP5 | 4430.53 | 1203.62 | 1625.41 | 1228.37 | 5.81 | 0.028 | 0.444 | 2.73 |
| MCM3 | 1949.03 | 484.64 | 716.99 | 437.27 | 31.37 | 0.001 | 0.475 | 2.72 |
| CHAF1B | 524.71 | 203.50 | 193.12 | 78.47 | 4.43 | 0.047 | 0.449 | 2.72 |
| PA2G4 | 2397.05 | 321.24 | 884.02 | 344.72 | 5.50 | 0.031 | 0.446 | 2.71 |
| DNAJC9 | 2704.12 | 855.18 | 1000.00 | 577.03 | 10.61 | 0.009 | 0.453 | 2.70 |
| LOC653874 | 738.50 | 306.69 | 273.44 | 156.05 | 4.66 | 0.043 | 0.448 | 2.70 |
| HNRNPAB | 1336.74 | 499.84 | 496.85 | 270.58 | 5.27 | 0.034 | 0.448 | 2.69 |
| LOC648210 | 5596.64 | 1714.55 | 2085.02 | 624.91 | 4.48 | 0.046 | 0.448 | 2.68 |
| RPP40 | 1067.62 | 403.16 | 398.06 | 167.83 | 4.30 | 0.050 | 0.452 | 2.68 |
| HNRNPAB | 3099.60 | 920.53 | 1155.96 | 725.68 | 11.11 | 0.008 | 0.457 | 2.68 |
| LDHA | 16974.01 | 4186.49 | 6374.29 | 3588.62 | 30.69 | 0.001 | 0.456 | 2.66 |
| IMPDH2 | 5121.72 | 1710.53 | 1930.28 | 982.19 | 7.14 | 0.019 | 0.449 | 2.65 |
| DCUN1D5 | 1544.82 | 702.24 | 582.40 | 390.74 | 5.35 | 0.033 | 0.446 | 2.65 |
| TIMM23 | 1452.99 | 592.74 | 547.79 | 271.41 | 4.88 | 0.040 | 0.444 | 2.65 |
| SRM | 1364.70 | 78.15 | 515.08 | 204.46 | 8.43 | 0.014 | 0.451 | 2.65 |
| RPL34 | 1307.55 | 378.46 | 493.61 | 196.02 | 7.40 | 0.018 | 0.448 | 2.65 |
| ODC1 | 3709.90 | 925.03 | 1405.05 | 632.58 | 10.30 | 0.009 | 0.452 | 2.64 |
| NFIL3 | 783.61 | 296.55 | 297.19 | 169.40 | 6.50 | 0.023 | 0.452 | 2.64 |
| ADSL | 3775.45 | 816.18 | 1435.35 | 372.80 | 8.34 | 0.014 | 0.452 | 2.63 |
| RUVBL1 | 569.39 | 130.45 | 216.73 | 41.15 | 6.82 | 0.021 | 0.448 | 2.63 |
| EBPL | 545.38 | 136.98 | 207.64 | 25.91 | 5.22 | 0.035 | 0.448 | 2.63 |
| PRKCDBP | 522.82 | 103.84 | 199.27 | 44.32 | 5.51 | 0.031 | 0.446 | 2.62 |
| GPATCH4 | 935.41 | 286.59 | 357.84 | 96.89 | 5.25 | 0.034 | 0.447 | 2.61 |
| MSH6 | 3071.31 | 1005.47 | 1176.47 | 597.98 | 8.05 | 0.015 | 0.454 | 2.61 |
| WDR75 | 2030.68 | 608.67 | 781.53 | 418.42 | 11.17 | 0.008 | 0.455 | 2.60 |
| HPRT1 | 3044.63 | 1202.28 | 1176.06 | 577.71 | 5.18 | 0.035 | 0.447 | 2.59 |
| PRMT1 | 908.31 | 130.35 | 351.03 | 111.03 | 22.78 | 0.002 | 0.454 | 2.59 |
| C15ORF23 | 439.30 | 120.36 | 170.04 | 17.88 | 4.52 | 0.046 | 0.448 | 2.58 |
| DUT | 456.81 | 170.99 | 177.05 | 69.83 | 4.63 | 0.044 | 0.447 | 2.58 |
| MTHFD2 | 3025.59 | 1249.86 | 1174.07 | 602.12 | 4.86 | 0.040 | 0.445 | 2.58 |
| POLD2 | 924.45 | 228.58 | 359.62 | 158.32 | 9.13 | 0.012 | 0.457 | 2.57 |
| HNRNPAB | 7589.67 | 2121.35 | 2955.65 | 1610.09 | 7.75 | 0.016 | 0.449 | 2.57 |
| NDUFAF2 | 831.14 | 216.18 | 323.97 | 79.62 | 5.89 | 0.028 | 0.446 | 2.57 |
| RBBP8 | 1378.62 | 434.67 | 537.86 | 231.26 | 6.49 | 0.023 | 0.452 | 2.56 |
| CD83 | 5780.75 | 1899.67 | 2256.08 | 1177.35 | 5.04 | 0.037 | 0.447 | 2.56 |
| SKP2 | 1224.29 | 342.11 | 479.01 | 188.71 | 6.09 | 0.026 | 0.450 | 2.56 |
| DHCR7 | 491.91 | 74.67 | 192.62 | 41.37 | 10.62 | 0.009 | 0.454 | 2.55 |
| LDHA | 15856.26 | 5310.18 | 6209.20 | 4546.43 | 12.12 | 0.007 | 0.443 | 2.55 |
| MCM5 | 1226.31 | 410.85 | 480.24 | 189.22 | 5.02 | 0.037 | 0.446 | 2.55 |
| NOP56 | 5422.53 | 1460.27 | 2135.21 | 1180.39 | 20.10 | 0.002 | 0.448 | 2.54 |
| MIR1978 | 3171.29 | 1067.52 | 1250.48 | 378.76 | 4.58 | 0.045 | 0.448 | 2.54 |
| LOC391811 | 4371.13 | 1267.72 | 1725.01 | 933.34 | 13.71 | 0.005 | 0.445 | 2.53 |
| MRPL39 | 1550.36 | 537.78 | 612.91 | 302.98 | 6.61 | 0.022 | 0.453 | 2.53 |
| NHP2 | 1260.17 | 313.73 | 498.68 | 196.55 | 11.25 | 0.008 | 0.456 | 2.53 |
| TRAP1 | 1085.36 | 365.57 | 429.66 | 165.60 | 5.02 | 0.038 | 0.446 | 2.53 |
| TACC3 | 1290.80 | 335.66 | 511.22 | 212.03 | 9.25 | 0.011 | 0.457 | 2.52 |
| HNRPA1P4 | 5381.58 | 1744.28 | 2132.64 | 812.38 | 5.61 | 0.030 | 0.445 | 2.52 |
| RSL1D1 | 2040.21 | 533.42 | 808.52 | 222.06 | 6.07 | 0.026 | 0.448 | 2.52 |
| MRPL1 | 1377.43 | 426.69 | 546.31 | 380.64 | 12.40 | 0.006 | 0.443 | 2.52 |
| PDXP | 1042.05 | 378.44 | 413.49 | 196.00 | 5.62 | 0.030 | 0.446 | 2.52 |
| SNHG1 | 1846.62 | 755.74 | 733.28 | 331.05 | 4.45 | 0.047 | 0.450 | 2.52 |
| PGAM4 | 1094.48 | 237.25 | 434.88 | 116.08 | 8.50 | 0.014 | 0.452 | 2.52 |
| RPA3 | 3328.65 | 935.28 | 1323.97 | 712.38 | 15.54 | 0.004 | 0.451 | 2.51 |
| CCND2 | 9149.25 | 1908.60 | 3642.79 | 2590.77 | 8.31 | 0.014 | 0.452 | 2.51 |
| PSMD12 | 1686.10 | 603.91 | 672.08 | 316.67 | 6.11 | 0.026 | 0.450 | 2.51 |
| PPA1 | 6462.44 | 2156.42 | 2576.90 | 1189.12 | 6.92 | 0.020 | 0.446 | 2.51 |
| FABP5L2 | 3775.06 | 1210.28 | 1506.90 | 1181.49 | 6.57 | 0.022 | 0.454 | 2.51 |
| HSPD1 | 17933.87 | 5250.53 | 7175.65 | 4330.46 | 17.09 | 0.003 | 0.455 | 2.50 |
| BCAR3 | 928.73 | 342.65 | 373.30 | 219.79 | 7.82 | 0.016 | 0.449 | 2.49 |
| GOT2 | 2702.23 | 1179.60 | 1087.77 | 616.64 | 4.78 | 0.041 | 0.444 | 2.48 |
| NCAPG2 | 441.78 | 134.77 | 177.92 | 34.07 | 4.47 | 0.046 | 0.449 | 2.48 |
| KIAA0020 | 1861.39 | 730.91 | 749.65 | 405.74 | 5.76 | 0.029 | 0.444 | 2.48 |
| ADCY3 | 2055.76 | 838.20 | 828.13 | 378.54 | 4.62 | 0.044 | 0.447 | 2.48 |
| NHP2 | 990.36 | 158.53 | 400.04 | 115.94 | 15.60 | 0.004 | 0.451 | 2.48 |
| LRP8 | 694.01 | 254.23 | 281.62 | 109.20 | 4.78 | 0.041 | 0.444 | 2.46 |
| DKC1 | 5232.82 | 1646.11 | 2123.57 | 1146.11 | 10.45 | 0.009 | 0.456 | 2.46 |
| ATIC | 4761.78 | 1601.81 | 1937.64 | 792.01 | 5.98 | 0.027 | 0.448 | 2.46 |
| SYNCRIP | 2715.10 | 816.90 | 1105.62 | 571.94 | 9.20 | 0.012 | 0.456 | 2.46 |
| GEMIN6 | 921.77 | 240.04 | 375.65 | 118.02 | 7.22 | 0.019 | 0.450 | 2.45 |
| ACLY | 1462.04 | 275.39 | 596.58 | 200.59 | 20.03 | 0.002 | 0.450 | 2.45 |
| E2F2 | 662.26 | 223.66 | 270.56 | 135.91 | 7.69 | 0.016 | 0.448 | 2.45 |
| HPRT1 | 1430.81 | 408.96 | 584.99 | 214.58 | 7.51 | 0.017 | 0.448 | 2.45 |
| UTP11L | 1275.67 | 427.61 | 521.92 | 218.47 | 5.97 | 0.027 | 0.447 | 2.44 |
| FOXM1 | 376.03 | 101.65 | 153.85 | 44.15 | 5.73 | 0.029 | 0.446 | 2.44 |
| SPRY1 | 464.09 | 129.80 | 189.94 | 21.47 | 4.37 | 0.049 | 0.451 | 2.44 |
| SERPINB1 | 1263.65 | 444.38 | 518.11 | 250.43 | 5.07 | 0.037 | 0.447 | 2.44 |
| RRS1 | 889.81 | 303.61 | 365.01 | 171.11 | 6.80 | 0.021 | 0.448 | 2.44 |
| KHSRP | 1474.94 | 335.51 | 606.03 | 248.77 | 5.34 | 0.033 | 0.446 | 2.43 |
| PDCD5 | 2291.88 | 714.49 | 942.51 | 488.63 | 10.32 | 0.009 | 0.453 | 2.43 |
| GFI1 | 648.97 | 252.17 | 267.18 | 111.23 | 4.69 | 0.043 | 0.446 | 2.43 |
| CENPN | 628.79 | 154.24 | 260.07 | 75.18 | 7.01 | 0.020 | 0.449 | 2.42 |
| MYL6B | 919.18 | 324.96 | 380.93 | 125.59 | 4.61 | 0.044 | 0.447 | 2.41 |
| PTRH2 | 989.34 | 280.12 | 410.11 | 157.50 | 7.63 | 0.017 | 0.446 | 2.41 |
| DHCR7 | 446.19 | 96.31 | 185.00 | 56.04 | 10.08 | 0.010 | 0.454 | 2.41 |
| LSM2 | 3023.23 | 724.83 | 1254.29 | 535.87 | 16.11 | 0.004 | 0.455 | 2.41 |
| MCM3 | 6959.53 | 2217.72 | 2895.78 | 1571.45 | 10.84 | 0.008 | 0.455 | 2.40 |
| GTPBP4 | 4746.55 | 2025.44 | 1978.79 | 1136.93 | 5.22 | 0.035 | 0.447 | 2.40 |
| GAPDH | 5825.90 | 363.92 | 2430.51 | 505.83 | 6.78 | 0.021 | 0.448 | 2.40 |
| SNRPB | 4755.47 | 1234.72 | 1984.11 | 798.47 | 11.00 | 0.008 | 0.456 | 2.40 |
| FABP5L2 | 12252.54 | 2930.04 | 5115.01 | 4175.49 | 9.78 | 0.010 | 0.457 | 2.40 |
| LOC732007 | 1281.16 | 281.84 | 534.94 | 198.58 | 12.86 | 0.006 | 0.437 | 2.39 |
| CARM1 | 2388.74 | 732.76 | 1000.06 | 332.39 | 5.96 | 0.027 | 0.448 | 2.39 |
| BYSL | 732.65 | 218.05 | 308.08 | 132.28 | 7.72 | 0.016 | 0.449 | 2.38 |
| SNRPF | 3848.68 | 1599.99 | 1619.28 | 904.85 | 5.42 | 0.032 | 0.445 | 2.38 |
| SNRPF | 4766.64 | 1685.26 | 2005.95 | 1037.13 | 7.36 | 0.018 | 0.449 | 2.38 |
| H2AFZ | 11362.89 | 5165.45 | 4785.94 | 2644.54 | 4.51 | 0.046 | 0.449 | 2.37 |
| GNL3 | 2332.93 | 834.70 | 983.38 | 423.59 | 5.57 | 0.031 | 0.445 | 2.37 |
| EXOSC8 | 1082.19 | 356.46 | 456.49 | 216.04 | 6.79 | 0.021 | 0.448 | 2.37 |
| C14ORF156 | 5072.46 | 2179.76 | 2146.09 | 1097.05 | 4.46 | 0.047 | 0.450 | 2.36 |
| IL4I1 | 930.07 | 226.48 | 393.80 | 92.66 | 6.02 | 0.027 | 0.448 | 2.36 |
| LOC647000 | 3421.90 | 1033.51 | 1451.87 | 884.61 | 6.93 | 0.020 | 0.447 | 2.36 |
| STOML2 | 3760.04 | 849.17 | 1598.46 | 625.06 | 13.75 | 0.005 | 0.448 | 2.35 |
| SLC25A19 | 1376.39 | 357.98 | 585.25 | 220.18 | 9.51 | 0.011 | 0.464 | 2.35 |
| PARP1 | 6029.68 | 2205.36 | 2566.24 | 1112.13 | 5.26 | 0.034 | 0.448 | 2.35 |
| AGK | 766.94 | 270.12 | 326.42 | 141.11 | 4.43 | 0.047 | 0.450 | 2.35 |
| LOC653147 | 1344.98 | 398.73 | 572.68 | 293.52 | 11.05 | 0.008 | 0.458 | 2.35 |
| RRAS2 | 850.43 | 307.11 | 362.12 | 159.42 | 5.51 | 0.031 | 0.446 | 2.35 |
| BCL2L12 | 1001.14 | 361.36 | 426.47 | 202.81 | 5.85 | 0.028 | 0.446 | 2.35 |
| LSM5 | 3013.03 | 1056.92 | 1284.00 | 544.84 | 5.80 | 0.028 | 0.444 | 2.35 |
| LOC653505 | 2158.27 | 783.91 | 920.04 | 338.15 | 4.61 | 0.044 | 0.447 | 2.35 |
| RGS16 | 477.33 | 109.78 | 204.15 | 98.45 | 8.23 | 0.014 | 0.451 | 2.34 |
| DSCC1 | 471.83 | 150.28 | 201.83 | 71.19 | 5.64 | 0.030 | 0.447 | 2.34 |
| MIF | 6134.34 | 1417.45 | 2624.26 | 1579.32 | 20.86 | 0.002 | 0.438 | 2.34 |
| HS.25892 | 1349.46 | 484.67 | 577.32 | 281.50 | 5.81 | 0.028 | 0.444 | 2.34 |
| MTERFD1 | 1189.87 | 272.65 | 510.52 | 194.68 | 6.08 | 0.026 | 0.449 | 2.33 |
| COQ2 | 1100.39 | 424.06 | 472.76 | 198.99 | 4.53 | 0.045 | 0.449 | 2.33 |
| AIMP2 | 2475.62 | 812.03 | 1065.03 | 541.50 | 8.64 | 0.013 | 0.451 | 2.32 |
| GPI | 1836.40 | 580.19 | 790.60 | 325.56 | 6.29 | 0.024 | 0.449 | 2.32 |
| BHLHB2 | 7009.63 | 2639.48 | 3020.19 | 2200.28 | 6.30 | 0.024 | 0.450 | 2.32 |
| LOC92755 | 1461.97 | 8.33 | 629.99 | 72.68 | 17.91 | 0.003 | 0.445 | 2.32 |
| MYC | 2700.65 | 1218.59 | 1167.60 | 753.00 | 4.31 | 0.050 | 0.452 | 2.31 |
| RNMTL1 | 1168.77 | 402.50 | 506.17 | 208.18 | 5.80 | 0.029 | 0.444 | 2.31 |
| TUBB2C | 2210.81 | 587.05 | 958.06 | 246.63 | 6.03 | 0.026 | 0.449 | 2.31 |
| ABCE1 | 1789.11 | 739.00 | 776.11 | 376.07 | 4.55 | 0.045 | 0.448 | 2.31 |
| NOP2 | 3078.57 | 728.39 | 1336.31 | 619.61 | 12.83 | 0.006 | 0.438 | 2.30 |
| RACGAP1 | 539.79 | 197.95 | 234.57 | 109.57 | 5.46 | 0.032 | 0.445 | 2.30 |
| MGC4677 | 2627.79 | 813.59 | 1142.12 | 668.74 | 13.20 | 0.006 | 0.443 | 2.30 |
| POLA2 | 989.36 | 382.86 | 430.25 | 206.41 | 5.47 | 0.032 | 0.445 | 2.30 |
| PLD6 | 910.95 | 255.06 | 397.66 | 182.72 | 11.37 | 0.008 | 0.452 | 2.29 |
| HSPA14 | 819.85 | 262.55 | 358.18 | 125.71 | 5.10 | 0.036 | 0.447 | 2.29 |
| PRIM1 | 947.11 | 375.48 | 414.06 | 193.22 | 4.93 | 0.039 | 0.445 | 2.29 |
| GPN3 | 996.84 | 343.18 | 436.07 | 199.42 | 5.45 | 0.032 | 0.445 | 2.29 |
| RPL26L1 | 1414.94 | 472.16 | 619.16 | 233.43 | 5.65 | 0.030 | 0.447 | 2.29 |
| PGAM4 | 3524.91 | 855.85 | 1542.80 | 722.35 | 25.68 | 0.002 | 0.439 | 2.28 |
| CUGBP2 | 705.68 | 197.08 | 309.10 | 91.52 | 6.48 | 0.023 | 0.452 | 2.28 |
| PYCR1 | 365.20 | 38.26 | 160.12 | 31.56 | 15.66 | 0.004 | 0.453 | 2.28 |
| MKI67IP | 1834.45 | 587.76 | 805.19 | 390.79 | 7.68 | 0.017 | 0.448 | 2.28 |
| SLC7A1 | 1831.22 | 454.55 | 803.82 | 424.74 | 50.50 | 0.000 | 0.639 | 2.28 |
| CCNE1 | 446.18 | 140.70 | 195.89 | 80.24 | 6.00 | 0.027 | 0.447 | 2.28 |
| LOC388275 | 2161.05 | 322.54 | 952.90 | 407.64 | 19.18 | 0.003 | 0.452 | 2.27 |
| MRPS12 | 788.92 | 115.04 | 348.66 | 71.80 | 11.37 | 0.008 | 0.452 | 2.26 |
| VARS2 | 719.76 | 234.27 | 318.12 | 107.26 | 5.38 | 0.033 | 0.445 | 2.26 |
| POLR1C | 1318.23 | 463.79 | 582.64 | 232.18 | 5.47 | 0.032 | 0.445 | 2.26 |
| MRPL4 | 439.54 | 26.36 | 194.50 | 8.80 | 22.94 | 0.002 | 0.452 | 2.26 |
| YARS2 | 1041.79 | 321.25 | 461.17 | 153.12 | 5.70 | 0.029 | 0.445 | 2.26 |
| PRMT1 | 2729.32 | 1016.49 | 1209.52 | 525.94 | 4.77 | 0.041 | 0.444 | 2.26 |
| CARHSP1 | 1500.01 | 480.56 | 664.79 | 250.92 | 6.05 | 0.026 | 0.448 | 2.26 |
| CDK4 | 2949.27 | 1117.93 | 1308.30 | 735.52 | 5.83 | 0.028 | 0.445 | 2.25 |
| WDR18 | 2520.32 | 956.43 | 1120.02 | 606.56 | 6.66 | 0.022 | 0.452 | 2.25 |
| PCNA | 4916.52 | 2221.82 | 2188.32 | 1647.84 | 4.92 | 0.039 | 0.445 | 2.25 |
| LOC100133328 | 748.47 | 151.47 | 333.53 | 97.65 | 10.87 | 0.008 | 0.455 | 2.24 |
| LOC648249 | 4586.39 | 1709.96 | 2046.39 | 754.24 | 4.58 | 0.045 | 0.448 | 2.24 |
| C17ORF58 | 1332.52 | 465.03 | 595.91 | 322.08 | 5.66 | 0.030 | 0.446 | 2.24 |
| MRPS30 | 1785.80 | 602.36 | 799.10 | 322.38 | 6.09 | 0.026 | 0.450 | 2.23 |
| COMTD1 | 585.49 | 65.90 | 262.34 | 54.20 | 47.74 | 0.000 | 0.610 | 2.23 |
| C17ORF45 | 8496.78 | 2238.29 | 3807.17 | 1634.40 | 6.43 | 0.023 | 0.453 | 2.23 |
| LRMP | 1382.26 | 349.75 | 619.71 | 140.13 | 6.01 | 0.027 | 0.448 | 2.23 |
| LOC648210 | 6049.94 | 2840.99 | 2716.42 | 1554.57 | 4.49 | 0.046 | 0.448 | 2.23 |
| IPO4 | 579.50 | 44.60 | 260.24 | 33.73 | 23.13 | 0.002 | 0.452 | 2.23 |
| NIP7 | 1424.42 | 390.15 | 640.09 | 333.80 | 18.03 | 0.003 | 0.451 | 2.23 |
| BRI3BP | 405.24 | 131.28 | 182.20 | 46.81 | 4.53 | 0.045 | 0.449 | 2.22 |
| LOC728069 | 1428.14 | 331.61 | 642.15 | 315.77 | 43.10 | 0.001 | 0.636 | 2.22 |
| EXOSC7 | 1122.82 | 422.94 | 505.43 | 204.93 | 4.67 | 0.043 | 0.448 | 2.22 |
| MRPL13 | 1737.42 | 449.79 | 783.17 | 351.14 | 16.42 | 0.004 | 0.454 | 2.22 |
| KIF22 | 1084.38 | 346.54 | 489.08 | 191.87 | 6.15 | 0.025 | 0.448 | 2.22 |
| FAM136A | 1150.80 | 506.47 | 519.12 | 263.63 | 4.44 | 0.047 | 0.449 | 2.22 |
| LRPPRC | 785.47 | 241.77 | 354.36 | 84.93 | 4.50 | 0.046 | 0.449 | 2.22 |
| METAP2 | 5010.77 | 1431.38 | 2260.83 | 1244.14 | 25.41 | 0.002 | 0.435 | 2.22 |
| SHMT2 | 4188.07 | 1472.96 | 1890.37 | 1036.11 | 7.88 | 0.016 | 0.450 | 2.22 |
| LOC642817 | 2094.19 | 748.11 | 945.40 | 294.56 | 4.31 | 0.050 | 0.452 | 2.22 |
| RGS1 | 1936.14 | 734.13 | 876.15 | 434.96 | 5.48 | 0.032 | 0.445 | 2.21 |
| LOC388707 | 2773.66 | 931.48 | 1256.25 | 494.44 | 5.62 | 0.030 | 0.446 | 2.21 |
| REXO2 | 2000.53 | 681.72 | 906.11 | 364.16 | 5.88 | 0.028 | 0.446 | 2.21 |
| POLR2D | 650.09 | 204.73 | 294.70 | 79.24 | 4.67 | 0.043 | 0.447 | 2.21 |
| IPO11 | 893.11 | 359.79 | 405.10 | 167.89 | 4.40 | 0.048 | 0.450 | 2.20 |
| PHB2 | 2329.97 | 389.98 | 1058.11 | 371.51 | 23.03 | 0.002 | 0.453 | 2.20 |
| SIVA | 575.50 | 156.73 | 261.49 | 50.87 | 5.13 | 0.036 | 0.447 | 2.20 |
| RRP1B | 499.10 | 92.12 | 226.84 | 39.97 | 6.44 | 0.023 | 0.453 | 2.20 |
| SKP2 | 322.02 | 63.70 | 146.57 | 10.15 | 5.15 | 0.036 | 0.446 | 2.20 |
| NAPSB | 2234.68 | 903.22 | 1019.38 | 468.69 | 4.81 | 0.041 | 0.444 | 2.19 |
| MRPL16 | 1550.32 | 492.55 | 708.07 | 274.64 | 5.62 | 0.030 | 0.445 | 2.19 |
| MRPS12 | 3978.62 | 1053.23 | 1817.18 | 746.98 | 11.48 | 0.007 | 0.450 | 2.19 |
| NME1 | 497.60 | 134.31 | 227.69 | 101.98 | 8.74 | 0.013 | 0.456 | 2.19 |
| CRIP1 | 5086.04 | 1350.90 | 2329.27 | 1469.17 | 4.46 | 0.047 | 0.450 | 2.18 |
| TFB2M | 1243.62 | 382.72 | 569.77 | 250.83 | 8.70 | 0.013 | 0.453 | 2.18 |
| MRPL36 | 1784.92 | 516.23 | 818.20 | 333.54 | 8.35 | 0.014 | 0.452 | 2.18 |
| CCT8 | 7708.55 | 2793.07 | 3534.53 | 1895.31 | 6.94 | 0.020 | 0.447 | 2.18 |
| HMGA1 | 3562.37 | 543.94 | 1634.62 | 914.82 | 8.77 | 0.013 | 0.456 | 2.18 |
| THOC4 | 277.84 | 69.35 | 127.68 | 18.82 | 4.99 | 0.038 | 0.447 | 2.18 |
| VARS | 1723.41 | 538.72 | 792.28 | 455.03 | 12.06 | 0.007 | 0.443 | 2.18 |
| C3ORF26 | 518.69 | 128.86 | 238.52 | 47.91 | 5.62 | 0.030 | 0.445 | 2.17 |
| DUSP10 | 592.11 | 81.01 | 272.42 | 47.68 | 15.82 | 0.004 | 0.449 | 2.17 |
| TUBG1 | 1187.40 | 378.27 | 546.41 | 163.56 | 5.15 | 0.036 | 0.446 | 2.17 |
| ITGB3BP | 500.91 | 173.76 | 231.15 | 112.09 | 7.53 | 0.017 | 0.446 | 2.17 |
| ASNS | 1237.06 | 405.29 | 571.28 | 190.24 | 5.31 | 0.034 | 0.447 | 2.17 |
| NAT10 | 1508.15 | 510.69 | 696.60 | 239.55 | 5.18 | 0.035 | 0.447 | 2.17 |
| C16ORF59 | 285.44 | 72.36 | 131.90 | 29.18 | 5.87 | 0.028 | 0.445 | 2.16 |
| CCT7 | 5137.06 | 1666.83 | 2382.86 | 1151.51 | 9.06 | 0.012 | 0.456 | 2.16 |
| SP140 | 1789.55 | 627.55 | 831.50 | 363.62 | 6.15 | 0.025 | 0.448 | 2.15 |
| HIBCH | 755.91 | 276.50 | 351.61 | 169.99 | 6.53 | 0.023 | 0.453 | 2.15 |
| RAB11FIP1 | 1907.07 | 709.30 | 887.52 | 386.44 | 5.43 | 0.032 | 0.444 | 2.15 |
| NHP2 | 1092.57 | 367.39 | 508.61 | 216.41 | 5.87 | 0.028 | 0.445 | 2.15 |
| PPM1G | 5445.50 | 1641.81 | 2535.74 | 1163.94 | 10.52 | 0.009 | 0.454 | 2.15 |
| BOLA3 | 2683.21 | 809.10 | 1249.97 | 624.30 | 6.55 | 0.023 | 0.454 | 2.15 |
| SIGMAR1 | 828.20 | 251.29 | 385.84 | 135.06 | 6.26 | 0.025 | 0.450 | 2.15 |
| LOC100128410 | 1066.41 | 405.99 | 496.88 | 316.63 | 4.37 | 0.049 | 0.451 | 2.15 |
| PLAGL2 | 797.64 | 226.23 | 373.40 | 100.51 | 5.16 | 0.036 | 0.446 | 2.14 |
| GAPDH | 9287.12 | 295.87 | 4353.43 | 1721.70 | 5.22 | 0.035 | 0.446 | 2.13 |
| LOC100131940 | 2145.53 | 921.03 | 1006.15 | 477.27 | 4.43 | 0.047 | 0.449 | 2.13 |
| FOXRED1 | 647.89 | 243.53 | 303.87 | 114.31 | 4.53 | 0.045 | 0.449 | 2.13 |
| C13ORF27 | 993.80 | 318.44 | 466.41 | 228.46 | 6.12 | 0.026 | 0.450 | 2.13 |
| PRICKLE4 | 3458.27 | 589.07 | 1626.51 | 698.99 | 13.52 | 0.005 | 0.446 | 2.13 |
| PRDX3 | 2834.58 | 1093.89 | 1333.90 | 559.04 | 4.84 | 0.040 | 0.444 | 2.13 |
| DDX1 | 3134.09 | 1094.60 | 1475.13 | 597.94 | 5.75 | 0.029 | 0.445 | 2.12 |
| RQCD1 | 563.56 | 216.36 | 265.29 | 107.15 | 4.34 | 0.049 | 0.452 | 2.12 |
| VBP1 | 1559.91 | 609.78 | 735.29 | 311.84 | 4.64 | 0.043 | 0.447 | 2.12 |
| C12ORF10 | 1693.74 | 498.42 | 798.87 | 322.09 | 8.53 | 0.013 | 0.452 | 2.12 |
| TRNT1 | 664.84 | 267.17 | 313.79 | 131.50 | 4.44 | 0.047 | 0.450 | 2.12 |
| CHAC2 | 334.09 | 127.94 | 157.77 | 66.55 | 4.44 | 0.047 | 0.450 | 2.12 |
| SNRNP40 | 2060.60 | 752.99 | 974.72 | 405.85 | 5.40 | 0.033 | 0.445 | 2.11 |
| OBFC2B | 602.83 | 122.46 | 285.83 | 70.11 | 10.13 | 0.010 | 0.454 | 2.11 |
| MRPL11 | 1582.98 | 455.50 | 751.03 | 250.15 | 6.71 | 0.021 | 0.450 | 2.11 |
| PDSS1 | 811.36 | 304.52 | 385.05 | 137.42 | 4.41 | 0.048 | 0.450 | 2.11 |
| C1ORF131 | 1243.83 | 352.95 | 590.94 | 201.34 | 7.41 | 0.018 | 0.448 | 2.10 |
| ABCE1 | 2240.95 | 890.90 | 1065.13 | 430.80 | 4.43 | 0.047 | 0.449 | 2.10 |
| RPL9 | 948.23 | 293.26 | 451.01 | 120.58 | 4.81 | 0.041 | 0.444 | 2.10 |
| STRA13 | 1980.34 | 418.69 | 942.53 | 368.85 | 34.83 | 0.001 | 0.526 | 2.10 |
| C16ORF35 | 1345.82 | 440.34 | 640.87 | 355.03 | 10.46 | 0.009 | 0.455 | 2.10 |
| SLC39A8 | 587.87 | 147.05 | 280.04 | 211.45 | 7.84 | 0.016 | 0.450 | 2.10 |
| RILPL2 | 1133.45 | 359.57 | 541.16 | 219.33 | 7.24 | 0.019 | 0.450 | 2.09 |
| KPNA3 | 1389.35 | 560.88 | 663.54 | 369.88 | 6.58 | 0.022 | 0.454 | 2.09 |
| DHCR24 | 493.34 | 192.44 | 235.79 | 115.55 | 5.49 | 0.032 | 0.446 | 2.09 |
| BOLA3 | 2845.63 | 664.74 | 1361.25 | 697.92 | 16.51 | 0.004 | 0.457 | 2.09 |
| C1ORF135 | 260.10 | 84.60 | 124.58 | 37.07 | 4.45 | 0.047 | 0.450 | 2.09 |
| UPF3B | 893.28 | 333.04 | 427.86 | 170.76 | 4.84 | 0.040 | 0.444 | 2.09 |
| TMEM14A | 364.03 | 92.43 | 174.69 | 42.98 | 6.38 | 0.024 | 0.453 | 2.08 |
| MRPL12 | 590.44 | 66.96 | 283.41 | 76.46 | 55.49 | 0.000 | 0.667 | 2.08 |
| APEX1 | 3994.21 | 879.21 | 1917.71 | 661.92 | 15.83 | 0.004 | 0.451 | 2.08 |
| CCT6A | 2055.36 | 759.80 | 987.24 | 657.76 | 8.25 | 0.014 | 0.452 | 2.08 |
| RG9MTD1 | 1085.92 | 387.77 | 521.80 | 306.79 | 6.09 | 0.026 | 0.449 | 2.08 |
| RANGAP1 | 2199.33 | 643.36 | 1057.16 | 552.73 | 10.36 | 0.009 | 0.451 | 2.08 |
| LOC401537 | 6032.61 | 1675.54 | 2905.82 | 747.65 | 4.50 | 0.046 | 0.449 | 2.08 |
| RAE1 | 764.26 | 255.77 | 368.46 | 122.68 | 4.91 | 0.039 | 0.444 | 2.07 |
| NIPA2 | 2687.61 | 755.36 | 1296.19 | 474.42 | 6.24 | 0.025 | 0.449 | 2.07 |
| MTHFD1 | 396.35 | 50.40 | 191.20 | 57.98 | 11.85 | 0.007 | 0.445 | 2.07 |
| LOC100130511 | 1531.62 | 602.67 | 739.29 | 403.06 | 5.25 | 0.034 | 0.448 | 2.07 |
| EXOSC9 | 1135.12 | 522.77 | 548.46 | 347.81 | 5.71 | 0.029 | 0.445 | 2.07 |
| SIVA1 | 566.18 | 98.32 | 273.87 | 60.71 | 6.99 | 0.020 | 0.449 | 2.07 |
| FDPS | 822.43 | 241.48 | 397.86 | 202.52 | 6.73 | 0.021 | 0.449 | 2.07 |
| MRPS27 | 1211.50 | 347.32 | 586.47 | 154.25 | 5.42 | 0.032 | 0.445 | 2.07 |
| LOC100132299 | 621.78 | 131.24 | 301.83 | 50.26 | 6.26 | 0.025 | 0.449 | 2.06 |
| NUP93 | 1631.03 | 294.61 | 791.88 | 281.20 | 56.94 | 0.000 | 0.729 | 2.06 |
| SLAMF1 | 1471.33 | 470.59 | 715.48 | 305.66 | 6.35 | 0.024 | 0.452 | 2.06 |
| SNRPC | 2093.07 | 717.27 | 1018.07 | 365.85 | 5.28 | 0.034 | 0.447 | 2.06 |
| LOC644774 | 1595.46 | 438.03 | 776.10 | 269.44 | 8.05 | 0.015 | 0.454 | 2.06 |
| HSPH1 | 5132.91 | 1985.47 | 2497.08 | 1363.74 | 6.65 | 0.022 | 0.453 | 2.06 |
| LOC100129267 | 505.60 | 139.66 | 246.07 | 91.27 | 9.29 | 0.011 | 0.460 | 2.05 |
| TPI1 | 3667.09 | 1196.92 | 1785.07 | 497.62 | 4.36 | 0.049 | 0.451 | 2.05 |
| LOC341457 | 13197.97 | 2155.82 | 6427.02 | 2417.45 | 31.93 | 0.001 | 0.487 | 2.05 |
| LOC389168 | 10820.58 | 3881.06 | 5296.21 | 1910.33 | 4.75 | 0.042 | 0.445 | 2.04 |
| EXO1 | 217.35 | 24.85 | 106.42 | 4.67 | 7.74 | 0.016 | 0.450 | 2.04 |
| BCAT2 | 612.66 | 121.31 | 300.37 | 95.23 | 18.09 | 0.003 | 0.453 | 2.04 |
| KIF4A | 244.39 | 54.99 | 119.93 | 27.05 | 7.71 | 0.016 | 0.448 | 2.04 |
| PDCL3 | 927.79 | 358.41 | 455.47 | 181.01 | 4.55 | 0.045 | 0.449 | 2.04 |
| DHRS3 | 580.69 | 185.93 | 285.08 | 87.39 | 4.55 | 0.045 | 0.448 | 2.04 |
| FKBP4 | 791.51 | 250.88 | 388.72 | 109.45 | 4.71 | 0.042 | 0.446 | 2.04 |
| LDHB | 6275.04 | 2320.22 | 3083.99 | 1671.72 | 8.52 | 0.013 | 0.452 | 2.03 |
| VKORC1L1 | 550.61 | 201.02 | 270.71 | 116.66 | 5.64 | 0.030 | 0.447 | 2.03 |
| TUBG1 | 710.30 | 232.44 | 349.38 | 151.73 | 5.08 | 0.037 | 0.447 | 2.03 |
| UTP14A | 471.38 | 93.08 | 231.94 | 33.76 | 6.25 | 0.025 | 0.449 | 2.03 |
| LOC606724 | 3964.83 | 1101.08 | 1952.78 | 629.96 | 6.57 | 0.022 | 0.454 | 2.03 |
| EIF5A | 1894.56 | 503.42 | 933.90 | 378.87 | 11.60 | 0.007 | 0.450 | 2.03 |
| AFG3L2 | 1700.52 | 469.50 | 839.05 | 273.48 | 7.61 | 0.017 | 0.445 | 2.03 |
| THOP1 | 695.43 | 163.17 | 343.59 | 134.77 | 21.36 | 0.002 | 0.451 | 2.02 |
| HSPE1 | 2024.90 | 955.70 | 1000.61 | 623.16 | 4.90 | 0.039 | 0.444 | 2.02 |
| TOMM40 | 3435.31 | 1211.00 | 1699.45 | 853.03 | 5.46 | 0.032 | 0.445 | 2.02 |
| SSBP1 | 3339.72 | 1174.31 | 1653.51 | 827.32 | 8.31 | 0.014 | 0.452 | 2.02 |
| OGFOD1 | 466.05 | 129.75 | 230.92 | 67.28 | 4.64 | 0.043 | 0.447 | 2.02 |
| SUMO2 | 2836.25 | 909.09 | 1406.40 | 593.07 | 7.35 | 0.018 | 0.450 | 2.02 |
| LSM4 | 2128.79 | 594.96 | 1055.92 | 395.34 | 9.28 | 0.011 | 0.460 | 2.02 |
| IDH3A | 853.81 | 217.07 | 423.80 | 142.12 | 8.78 | 0.013 | 0.457 | 2.01 |
| LOC728324 | 742.08 | 198.27 | 368.42 | 57.12 | 4.43 | 0.047 | 0.449 | 2.01 |
| NUDT5 | 1670.93 | 615.23 | 829.76 | 374.07 | 5.64 | 0.030 | 0.447 | 2.01 |
| RAD51C | 606.49 | 174.45 | 301.59 | 144.05 | 14.93 | 0.004 | 0.452 | 2.01 |
| MRPL37 | 2570.92 | 1011.79 | 1281.05 | 701.51 | 6.21 | 0.025 | 0.449 | 2.01 |
| TUBB | 7516.64 | 2363.97 | 3747.49 | 2234.41 | 5.55 | 0.031 | 0.444 | 2.01 |
| CBX5 | 1098.42 | 430.68 | 548.65 | 343.88 | 10.97 | 0.008 | 0.455 | 2.00 |
| SAE1 | 4531.98 | 1660.43 | 2264.94 | 960.64 | 5.58 | 0.031 | 0.445 | 2.00 |
| SLC25A22 | 560.96 | 200.64 | 280.36 | 128.30 | 5.82 | 0.028 | 0.444 | 2.00 |
| PHF19 | 1128.22 | 309.91 | 563.94 | 206.77 | 9.06 | 0.012 | 0.456 | 2.00 |
| SIAH2 | 489.29 | 86.54 | 244.88 | 29.67 | 6.18 | 0.025 | 0.448 | 2.00 |
| GNL2 | 2256.45 | 758.77 | 1131.66 | 401.25 | 5.44 | 0.032 | 0.445 | 1.99 |
| SFRS1 | 5358.60 | 2183.97 | 2688.80 | 1459.20 | 6.26 | 0.025 | 0.449 | 1.99 |
| MRPL35 | 1800.12 | 556.18 | 903.65 | 434.71 | 8.14 | 0.015 | 0.451 | 1.99 |
| IMP4 | 2953.27 | 629.82 | 1483.43 | 608.64 | 34.14 | 0.001 | 0.494 | 1.99 |
| GAR1 | 1313.46 | 594.63 | 660.17 | 509.08 | 6.66 | 0.022 | 0.452 | 1.99 |
| POLR1C | 741.49 | 241.04 | 372.88 | 134.05 | 4.60 | 0.044 | 0.447 | 1.99 |
| LOC400455 | 2012.68 | 695.72 | 1012.34 | 427.26 | 5.95 | 0.027 | 0.447 | 1.99 |
| SIGMAR1 | 569.91 | 60.41 | 286.68 | 89.20 | 12.61 | 0.006 | 0.440 | 1.99 |
| EIF3B | 6948.57 | 2007.16 | 3505.51 | 1574.09 | 8.33 | 0.014 | 0.452 | 1.98 |
| ACOT7 | 1495.25 | 577.28 | 757.48 | 557.42 | 7.06 | 0.020 | 0.449 | 1.97 |
| VDAC1 | 6925.99 | 2295.64 | 3509.01 | 1778.74 | 8.62 | 0.013 | 0.451 | 1.97 |
| SFXN4 | 679.09 | 263.41 | 344.14 | 137.32 | 4.57 | 0.045 | 0.448 | 1.97 |
| POLR2H | 3279.06 | 1216.11 | 1663.74 | 812.25 | 6.55 | 0.023 | 0.454 | 1.97 |
| PKMYT1 | 245.18 | 36.48 | 124.76 | 9.44 | 5.76 | 0.029 | 0.444 | 1.97 |
| LOC644877 | 475.46 | 150.35 | 242.02 | 63.11 | 4.45 | 0.047 | 0.450 | 1.96 |
| ETFA | 2411.36 | 715.89 | 1227.70 | 483.19 | 8.33 | 0.014 | 0.452 | 1.96 |
| MRPL42 | 598.36 | 172.74 | 304.81 | 113.30 | 6.98 | 0.020 | 0.448 | 1.96 |
| CDCA4 | 557.74 | 148.63 | 284.41 | 99.25 | 4.67 | 0.043 | 0.447 | 1.96 |
| POLR2F | 2165.13 | 555.70 | 1104.15 | 332.81 | 8.22 | 0.014 | 0.452 | 1.96 |
| HNRNPC | 1264.37 | 472.89 | 644.90 | 239.85 | 4.60 | 0.044 | 0.447 | 1.96 |
| UTP14A | 867.24 | 208.36 | 442.52 | 138.00 | 7.41 | 0.018 | 0.449 | 1.96 |
| MRPL17 | 1645.62 | 402.88 | 840.09 | 305.07 | 10.78 | 0.009 | 0.454 | 1.96 |
| SLC39A14 | 379.58 | 111.31 | 193.96 | 68.76 | 5.22 | 0.035 | 0.448 | 1.96 |
| MRPL22 | 2316.00 | 916.27 | 1184.65 | 567.82 | 5.49 | 0.032 | 0.445 | 1.96 |
| RBM18 | 737.18 | 255.27 | 377.48 | 142.53 | 5.52 | 0.031 | 0.446 | 1.95 |
| CCNE1 | 363.01 | 116.24 | 185.88 | 53.27 | 4.63 | 0.044 | 0.447 | 1.95 |
| LOC642197 | 740.13 | 278.10 | 379.17 | 142.24 | 4.49 | 0.046 | 0.448 | 1.95 |
| XRCC6 | 3444.31 | 1011.63 | 1765.90 | 437.04 | 4.35 | 0.049 | 0.452 | 1.95 |
| LOC729317 | 2409.44 | 380.55 | 1236.31 | 318.99 | 21.27 | 0.002 | 0.449 | 1.95 |
| C6ORF153 | 1623.05 | 601.88 | 833.32 | 390.36 | 6.09 | 0.026 | 0.450 | 1.95 |
| ACOT7 | 480.34 | 153.02 | 246.64 | 96.17 | 6.17 | 0.025 | 0.448 | 1.95 |
| PDCD2 | 1333.80 | 353.07 | 685.59 | 174.56 | 5.35 | 0.033 | 0.446 | 1.95 |
| ZNF593 | 1147.11 | 305.93 | 589.80 | 237.04 | 8.57 | 0.013 | 0.451 | 1.94 |
| GRAMD1A | 983.35 | 351.99 | 506.58 | 191.93 | 5.15 | 0.036 | 0.446 | 1.94 |
| POP7 | 440.46 | 109.06 | 226.91 | 66.99 | 8.60 | 0.013 | 0.450 | 1.94 |
| COPS3 | 2665.47 | 944.05 | 1373.19 | 568.29 | 5.55 | 0.031 | 0.444 | 1.94 |
| HSD17B12 | 1072.48 | 469.97 | 552.86 | 274.03 | 4.55 | 0.045 | 0.448 | 1.94 |
| CYB5B | 4652.21 | 1737.98 | 2398.22 | 1323.94 | 8.45 | 0.014 | 0.452 | 1.94 |
| NCL | 10107.03 | 4765.62 | 5218.96 | 3092.79 | 4.83 | 0.040 | 0.444 | 1.94 |
| MRPL20 | 697.22 | 209.01 | 360.07 | 97.57 | 5.14 | 0.036 | 0.446 | 1.94 |
| SNAPC4 | 1015.29 | 379.66 | 524.87 | 225.11 | 5.49 | 0.032 | 0.446 | 1.93 |
| ESD | 3186.51 | 507.70 | 1647.63 | 508.39 | 20.69 | 0.002 | 0.440 | 1.93 |
| LOC100130233 | 398.82 | 38.53 | 206.22 | 16.85 | 12.76 | 0.006 | 0.440 | 1.93 |
| TIAL1 | 941.11 | 365.09 | 487.17 | 186.88 | 4.38 | 0.048 | 0.451 | 1.93 |
| SNRPB | 3561.22 | 562.61 | 1843.57 | 457.62 | 9.53 | 0.011 | 0.464 | 1.93 |
| DIAPH3 | 214.32 | 54.98 | 111.05 | 22.40 | 5.20 | 0.035 | 0.447 | 1.93 |
| HAT1 | 491.23 | 150.62 | 254.57 | 75.07 | 5.19 | 0.035 | 0.447 | 1.93 |
| CUTC | 755.39 | 250.01 | 391.71 | 128.53 | 4.43 | 0.047 | 0.449 | 1.93 |
| LOC100131735 | 1232.91 | 265.59 | 639.50 | 224.01 | 24.50 | 0.002 | 0.437 | 1.93 |
| FABP5 | 331.07 | 71.56 | 171.73 | 42.32 | 5.07 | 0.037 | 0.446 | 1.93 |
| SNRPB2 | 2813.14 | 908.87 | 1459.51 | 502.87 | 4.64 | 0.043 | 0.447 | 1.93 |
| BMS1 | 1758.83 | 454.42 | 912.57 | 307.53 | 9.83 | 0.010 | 0.457 | 1.93 |
| SREBF1 | 419.77 | 123.98 | 217.89 | 73.96 | 6.28 | 0.024 | 0.449 | 1.93 |
| TRAPPC4 | 1487.28 | 482.47 | 772.41 | 375.25 | 6.76 | 0.021 | 0.448 | 1.93 |
| NSUN2 | 3635.04 | 1260.68 | 1888.84 | 829.25 | 6.97 | 0.020 | 0.448 | 1.92 |
| SLC29A1 | 404.55 | 103.22 | 210.38 | 87.98 | 7.32 | 0.018 | 0.450 | 1.92 |
| PPP1R14B | 1449.42 | 262.91 | 754.16 | 281.52 | 8.81 | 0.013 | 0.457 | 1.92 |
| YWHAH | 7100.72 | 2535.21 | 3695.24 | 2060.40 | 11.99 | 0.007 | 0.444 | 1.92 |
| RASSF2 | 1379.62 | 332.33 | 718.07 | 165.40 | 6.79 | 0.021 | 0.448 | 1.92 |
| DUSP5 | 2613.36 | 850.27 | 1360.78 | 787.58 | 8.07 | 0.015 | 0.453 | 1.92 |
| RUVBL1 | 277.57 | 34.15 | 144.57 | 15.33 | 10.56 | 0.009 | 0.454 | 1.92 |
| DTD1 | 557.34 | 127.16 | 290.42 | 101.56 | 17.58 | 0.003 | 0.448 | 1.92 |
| PRPSAP2 | 1221.72 | 496.36 | 636.81 | 301.14 | 4.55 | 0.045 | 0.448 | 1.92 |
| DBI | 7131.16 | 2279.43 | 3717.34 | 1581.78 | 8.32 | 0.014 | 0.452 | 1.92 |
| MRPS11 | 975.70 | 333.53 | 508.82 | 162.38 | 4.66 | 0.043 | 0.448 | 1.92 |
| SETMAR | 572.42 | 152.69 | 298.53 | 76.01 | 6.00 | 0.027 | 0.447 | 1.92 |
| SLC25A3 | 5879.80 | 1986.75 | 3066.67 | 1265.49 | 5.82 | 0.028 | 0.445 | 1.92 |
| SLC29A1 | 371.12 | 78.68 | 193.64 | 57.29 | 6.43 | 0.023 | 0.452 | 1.92 |
| TTLL12 | 736.55 | 182.38 | 384.73 | 192.61 | 33.23 | 0.001 | 0.491 | 1.91 |
| LOC644131 | 1726.83 | 741.80 | 902.47 | 591.45 | 7.11 | 0.019 | 0.449 | 1.91 |
| STAMBPL1 | 1860.04 | 657.38 | 973.19 | 576.03 | 5.64 | 0.030 | 0.447 | 1.91 |
| SKA2 | 709.62 | 267.45 | 371.41 | 199.75 | 7.11 | 0.019 | 0.450 | 1.91 |
| UBE2M | 2894.11 | 674.85 | 1515.04 | 740.80 | 36.22 | 0.001 | 0.522 | 1.91 |
| KIF18A | 255.02 | 41.66 | 133.76 | 18.62 | 8.19 | 0.015 | 0.453 | 1.91 |
| LOC730534 | 7049.38 | 1575.63 | 3700.57 | 1370.79 | 6.36 | 0.024 | 0.452 | 1.90 |
| CS | 3839.66 | 1098.73 | 2017.54 | 671.67 | 7.39 | 0.018 | 0.449 | 1.90 |
| GPN1 | 1426.61 | 414.35 | 749.70 | 197.47 | 4.91 | 0.039 | 0.445 | 1.90 |
| APOBEC3G | 818.14 | 172.85 | 429.97 | 105.96 | 8.02 | 0.015 | 0.452 | 1.90 |
| PRDX6 | 2180.51 | 617.01 | 1146.81 | 390.01 | 5.75 | 0.029 | 0.445 | 1.90 |
| GLRX5 | 4237.18 | 1667.17 | 2229.35 | 1140.89 | 6.60 | 0.022 | 0.453 | 1.90 |
| HIGD1A | 2418.09 | 1018.66 | 1272.33 | 674.79 | 5.71 | 0.029 | 0.445 | 1.90 |
| C21ORF70 | 400.96 | 118.63 | 211.07 | 62.59 | 5.80 | 0.028 | 0.444 | 1.90 |
| TIMM23 | 5707.55 | 1852.44 | 3008.51 | 1510.15 | 10.16 | 0.010 | 0.453 | 1.90 |
| TUBA1C | 10119.75 | 1388.15 | 5337.58 | 1125.54 | 14.90 | 0.004 | 0.451 | 1.90 |
| LOC100129673 | 447.00 | 127.49 | 236.00 | 47.87 | 4.39 | 0.048 | 0.451 | 1.89 |
| C10ORF2 | 415.59 | 85.19 | 219.43 | 51.74 | 6.90 | 0.020 | 0.446 | 1.89 |
| SCARB1 | 362.81 | 78.39 | 191.60 | 31.59 | 5.79 | 0.029 | 0.444 | 1.89 |
| LOC100130178 | 1497.40 | 444.79 | 791.32 | 335.03 | 9.15 | 0.012 | 0.457 | 1.89 |
| SLC5A6 | 800.01 | 260.63 | 422.89 | 151.20 | 4.65 | 0.043 | 0.447 | 1.89 |
| CCNC | 748.86 | 279.65 | 396.19 | 171.61 | 5.51 | 0.031 | 0.446 | 1.89 |
| HS.213061 | 3367.95 | 1326.41 | 1782.85 | 815.23 | 5.37 | 0.033 | 0.445 | 1.89 |
| POLE | 353.82 | 82.92 | 187.35 | 35.64 | 5.40 | 0.033 | 0.445 | 1.89 |
| MRPL46 | 739.59 | 234.89 | 391.84 | 115.46 | 5.00 | 0.038 | 0.447 | 1.89 |
| GBE1 | 558.32 | 144.56 | 296.16 | 74.58 | 6.47 | 0.023 | 0.453 | 1.89 |
| PRDX1 | 9131.35 | 1719.02 | 4849.51 | 1787.77 | 29.28 | 0.001 | 0.451 | 1.88 |
| KIAA0114 | 581.36 | 169.73 | 309.09 | 113.63 | 8.21 | 0.015 | 0.452 | 1.88 |
| LSM3 | 1354.82 | 460.17 | 720.42 | 275.10 | 5.85 | 0.028 | 0.446 | 1.88 |
| FBL | 2914.66 | 1045.49 | 1550.02 | 818.15 | 6.33 | 0.024 | 0.451 | 1.88 |
| FAM113B | 1581.36 | 544.76 | 841.49 | 284.15 | 4.63 | 0.044 | 0.447 | 1.88 |
| THOC7 | 2843.21 | 999.35 | 1513.74 | 604.37 | 5.55 | 0.031 | 0.445 | 1.88 |
| HDDC2 | 2960.09 | 994.54 | 1576.78 | 615.42 | 6.20 | 0.025 | 0.449 | 1.88 |
| CDC23 | 1426.41 | 645.78 | 760.03 | 388.40 | 4.43 | 0.047 | 0.450 | 1.88 |
| PSMB2 | 3190.28 | 819.14 | 1700.45 | 832.39 | 39.18 | 0.001 | 0.540 | 1.88 |
| ESPL1 | 327.97 | 74.10 | 174.91 | 47.10 | 4.54 | 0.045 | 0.449 | 1.88 |
| ZWILCH | 346.98 | 112.60 | 185.10 | 53.06 | 4.67 | 0.043 | 0.448 | 1.87 |
| RARS | 3472.19 | 1388.29 | 1855.62 | 863.25 | 5.30 | 0.034 | 0.447 | 1.87 |
| MTERFD1 | 778.68 | 214.45 | 416.33 | 113.41 | 4.94 | 0.039 | 0.445 | 1.87 |
| PIP5K1B | 258.38 | 23.19 | 138.20 | 4.52 | 9.82 | 0.010 | 0.457 | 1.87 |
| DDX39 | 4942.61 | 1583.79 | 2647.34 | 1158.89 | 7.98 | 0.015 | 0.452 | 1.87 |
| LOC440589 | 14074.64 | 4063.14 | 7550.76 | 1871.24 | 5.14 | 0.036 | 0.446 | 1.86 |
| CLPP | 567.63 | 172.38 | 304.74 | 84.26 | 5.14 | 0.036 | 0.446 | 1.86 |
| NXT1 | 1842.72 | 775.12 | 989.96 | 475.80 | 4.93 | 0.039 | 0.446 | 1.86 |
| TOMM22 | 1570.20 | 382.31 | 844.03 | 227.90 | 7.31 | 0.018 | 0.450 | 1.86 |
| RUVBL2 | 560.17 | 122.88 | 301.65 | 62.77 | 6.13 | 0.026 | 0.449 | 1.86 |
| PRDX4 | 1374.69 | 456.65 | 740.79 | 297.65 | 6.77 | 0.021 | 0.448 | 1.86 |
| EIF3B | 1748.49 | 502.14 | 942.51 | 324.08 | 7.21 | 0.019 | 0.450 | 1.86 |
| SNRNP25 | 318.91 | 46.76 | 171.95 | 30.07 | 15.25 | 0.004 | 0.449 | 1.85 |
| POLR3B | 636.01 | 229.07 | 343.04 | 140.65 | 4.75 | 0.042 | 0.444 | 1.85 |
| NME1-NME2 | 3758.61 | 866.00 | 2028.26 | 799.12 | 7.11 | 0.019 | 0.450 | 1.85 |
| TMEM5 | 868.83 | 254.51 | 469.10 | 140.94 | 5.10 | 0.036 | 0.446 | 1.85 |
| LOC730029 | 7821.67 | 2371.70 | 4223.55 | 1708.49 | 9.15 | 0.012 | 0.457 | 1.85 |
| LOC729535 | 379.24 | 66.09 | 204.85 | 48.26 | 16.47 | 0.004 | 0.457 | 1.85 |
| SCARNA10 | 353.69 | 48.56 | 191.07 | 10.63 | 5.20 | 0.035 | 0.447 | 1.85 |
| NOP14 | 646.27 | 169.65 | 349.17 | 177.38 | 5.18 | 0.035 | 0.447 | 1.85 |
| YEATS4 | 892.47 | 374.96 | 482.65 | 216.46 | 4.33 | 0.049 | 0.452 | 1.85 |
| N6AMT2 | 415.66 | 137.40 | 224.83 | 74.48 | 5.11 | 0.036 | 0.447 | 1.85 |
| PMPCA | 1116.86 | 358.41 | 604.15 | 212.98 | 5.46 | 0.032 | 0.445 | 1.85 |
| TPI1 | 3466.24 | 905.83 | 1875.19 | 786.60 | 12.18 | 0.007 | 0.441 | 1.85 |
| LOC728188 | 337.00 | 56.10 | 182.38 | 24.92 | 6.23 | 0.025 | 0.449 | 1.85 |
| FAM86A | 406.20 | 130.36 | 219.88 | 89.61 | 7.12 | 0.019 | 0.450 | 1.85 |
| HNRNPM | 3481.16 | 965.01 | 1885.91 | 682.72 | 6.80 | 0.021 | 0.448 | 1.85 |
| LOC440043 | 11731.09 | 4721.20 | 6356.65 | 4072.62 | 5.63 | 0.030 | 0.446 | 1.85 |
| RNASEH2A | 439.42 | 86.96 | 238.16 | 77.74 | 18.01 | 0.003 | 0.449 | 1.85 |
| EPRS | 2722.98 | 888.13 | 1476.84 | 630.11 | 8.30 | 0.014 | 0.452 | 1.84 |
| SPRY1 | 293.65 | 30.50 | 159.45 | 13.04 | 8.67 | 0.013 | 0.452 | 1.84 |
| APEX2 | 802.38 | 252.57 | 436.74 | 124.70 | 4.92 | 0.039 | 0.445 | 1.84 |
| PPAN | 341.74 | 58.57 | 186.04 | 33.97 | 10.93 | 0.008 | 0.455 | 1.84 |
| PHF19 | 313.60 | 84.08 | 170.73 | 40.63 | 5.54 | 0.031 | 0.444 | 1.84 |
| SNRPA | 847.37 | 180.73 | 461.48 | 152.05 | 7.34 | 0.018 | 0.450 | 1.84 |
| SFRS3 | 981.28 | 350.35 | 534.53 | 209.72 | 5.47 | 0.032 | 0.445 | 1.84 |
| RPL29 | 246.32 | 57.59 | 134.49 | 15.81 | 4.62 | 0.044 | 0.447 | 1.83 |
| KIAA0391 | 863.08 | 283.83 | 471.44 | 151.07 | 4.73 | 0.042 | 0.445 | 1.83 |
| C7ORF68 | 266.89 | 60.62 | 146.25 | 15.69 | 4.59 | 0.044 | 0.447 | 1.82 |
| SF3B4 | 2878.71 | 788.73 | 1578.21 | 622.15 | 12.47 | 0.006 | 0.444 | 1.82 |
| MRPS28 | 969.10 | 391.03 | 531.34 | 254.85 | 4.55 | 0.045 | 0.448 | 1.82 |
| CDK7 | 976.48 | 314.86 | 535.68 | 174.74 | 5.45 | 0.032 | 0.445 | 1.82 |
| LOC100130003 | 8344.25 | 1742.84 | 4578.79 | 1996.64 | 15.34 | 0.004 | 0.451 | 1.82 |
| RRP7A | 1670.60 | 472.68 | 917.89 | 489.99 | 72.70 | 0.000 | 0.745 | 1.82 |
| SIVA | 1016.43 | 309.54 | 558.48 | 210.66 | 6.75 | 0.021 | 0.449 | 1.82 |
| PTPN7 | 574.01 | 116.41 | 315.60 | 99.46 | 24.28 | 0.002 | 0.435 | 1.82 |
| C1ORF57 | 554.70 | 125.82 | 305.31 | 89.24 | 5.83 | 0.028 | 0.445 | 1.82 |
| PRMT6 | 976.67 | 397.35 | 537.87 | 237.67 | 4.76 | 0.041 | 0.444 | 1.82 |
| MPP6 | 283.69 | 74.81 | 156.27 | 46.13 | 6.53 | 0.023 | 0.453 | 1.82 |
| SAP30 | 758.51 | 281.93 | 417.84 | 222.43 | 7.41 | 0.018 | 0.449 | 1.82 |
| LOC100129585 | 875.60 | 261.61 | 482.43 | 202.75 | 7.82 | 0.016 | 0.449 | 1.81 |
| C16ORF53 | 1262.94 | 625.08 | 696.25 | 399.54 | 4.32 | 0.050 | 0.452 | 1.81 |
| XRCC6 | 1888.02 | 457.61 | 1041.01 | 363.36 | 13.71 | 0.005 | 0.446 | 1.81 |
| LOC644315 | 3192.11 | 1272.50 | 1760.26 | 722.55 | 4.44 | 0.047 | 0.449 | 1.81 |
| PELO | 1268.09 | 507.57 | 699.68 | 349.52 | 6.16 | 0.025 | 0.448 | 1.81 |
| APTX | 883.81 | 271.82 | 487.70 | 190.92 | 8.05 | 0.015 | 0.453 | 1.81 |
| PELP1 | 998.19 | 265.38 | 551.11 | 138.15 | 5.71 | 0.029 | 0.445 | 1.81 |
| C3ORF31 | 620.00 | 203.25 | 342.34 | 117.32 | 5.19 | 0.035 | 0.447 | 1.81 |
| ORC1L | 266.67 | 46.70 | 147.41 | 15.74 | 6.09 | 0.026 | 0.450 | 1.81 |
| MRPL2 | 543.36 | 44.02 | 300.41 | 28.85 | 27.07 | 0.001 | 0.444 | 1.81 |
| ATP5G1 | 2078.48 | 600.30 | 1149.58 | 570.98 | 30.93 | 0.001 | 0.470 | 1.81 |
| PHACTR1 | 291.15 | 25.85 | 161.09 | 17.54 | 5.59 | 0.031 | 0.445 | 1.81 |
| NOP2 | 447.65 | 80.90 | 247.77 | 54.04 | 6.91 | 0.020 | 0.446 | 1.81 |
| KBTBD8 | 986.77 | 382.73 | 546.42 | 362.32 | 6.86 | 0.021 | 0.447 | 1.81 |
| LOC100129067 | 396.13 | 32.47 | 219.38 | 68.70 | 6.32 | 0.024 | 0.451 | 1.81 |
| ENO1 | 18387.06 | 4518.98 | 10184.79 | 5275.24 | 18.76 | 0.003 | 0.458 | 1.81 |
| PSMA1 | 4365.66 | 1359.55 | 2421.31 | 1055.66 | 7.81 | 0.016 | 0.449 | 1.80 |
| SHFM1 | 2508.86 | 688.74 | 1391.67 | 512.32 | 8.62 | 0.013 | 0.450 | 1.80 |
| LOC642197 | 666.80 | 154.84 | 370.12 | 186.10 | 9.33 | 0.011 | 0.463 | 1.80 |
| PDHX | 606.48 | 191.82 | 337.10 | 136.70 | 8.37 | 0.014 | 0.452 | 1.80 |
| EIF6 | 1295.66 | 327.95 | 720.84 | 198.60 | 5.80 | 0.028 | 0.444 | 1.80 |
| BTF3 | 10942.42 | 3085.58 | 6087.96 | 2404.57 | 8.34 | 0.014 | 0.452 | 1.80 |
| ZCCHC9 | 1030.98 | 340.32 | 574.23 | 196.60 | 5.28 | 0.034 | 0.447 | 1.80 |
| NUP85 | 1261.96 | 317.94 | 703.04 | 217.65 | 7.99 | 0.015 | 0.452 | 1.80 |
| U2AF1 | 982.60 | 394.87 | 548.30 | 256.21 | 5.42 | 0.032 | 0.445 | 1.79 |
| GPN2 | 1143.12 | 346.82 | 638.38 | 313.47 | 19.34 | 0.003 | 0.454 | 1.79 |
| HNRNPK | 4093.24 | 1188.37 | 2285.97 | 565.33 | 4.64 | 0.044 | 0.447 | 1.79 |
| PRMT6 | 628.19 | 175.49 | 351.11 | 69.52 | 4.49 | 0.046 | 0.448 | 1.79 |
| PRPS2 | 824.03 | 148.73 | 460.91 | 178.02 | 11.23 | 0.008 | 0.456 | 1.79 |
| MRPS2 | 447.14 | 65.86 | 250.11 | 66.37 | 40.84 | 0.001 | 0.566 | 1.79 |
| LOC390557 | 12155.33 | 2568.79 | 6803.28 | 2729.79 | 8.39 | 0.014 | 0.452 | 1.79 |
| DDX56 | 949.34 | 276.30 | 531.61 | 187.20 | 8.12 | 0.015 | 0.452 | 1.79 |
| C17ORF79 | 1042.61 | 372.10 | 583.99 | 292.40 | 7.98 | 0.015 | 0.452 | 1.79 |
| COPS4 | 916.52 | 301.02 | 513.59 | 206.71 | 6.86 | 0.021 | 0.446 | 1.78 |
| CCT4 | 370.05 | 103.06 | 207.62 | 66.17 | 7.62 | 0.017 | 0.446 | 1.78 |
| HSPA4 | 3468.00 | 1317.48 | 1946.08 | 1106.05 | 9.26 | 0.011 | 0.458 | 1.78 |
| RIOK1 | 525.15 | 156.05 | 294.71 | 117.76 | 10.01 | 0.010 | 0.454 | 1.78 |
| TUBA1C | 6874.67 | 2513.15 | 3858.99 | 1445.23 | 4.81 | 0.041 | 0.444 | 1.78 |
| PPP1R16B | 1688.63 | 677.36 | 948.09 | 442.75 | 5.21 | 0.035 | 0.447 | 1.78 |
| UBE2I | 3583.16 | 1129.54 | 2012.60 | 828.77 | 7.59 | 0.017 | 0.445 | 1.78 |
| PBRM1 | 268.07 | 18.90 | 150.63 | 23.15 | 15.99 | 0.004 | 0.454 | 1.78 |
| DKFZP761P0423 | 762.29 | 41.87 | 428.49 | 83.81 | 8.01 | 0.015 | 0.452 | 1.78 |
| NOB1 | 466.88 | 79.59 | 262.45 | 74.40 | 15.02 | 0.004 | 0.451 | 1.78 |
| PRELID1 | 1467.19 | 417.81 | 825.99 | 250.80 | 5.50 | 0.032 | 0.446 | 1.78 |
| TOPBP1 | 1102.56 | 323.78 | 620.84 | 215.15 | 7.32 | 0.018 | 0.450 | 1.78 |
| RASSF2 | 2378.54 | 627.20 | 1339.97 | 624.48 | 38.79 | 0.001 | 0.523 | 1.78 |
| RNPS1 | 1902.11 | 504.50 | 1071.81 | 264.83 | 6.00 | 0.027 | 0.447 | 1.77 |
| PRDX1 | 11114.54 | 2233.38 | 6265.36 | 2365.06 | 11.08 | 0.008 | 0.457 | 1.77 |
| SAPS1 | 610.76 | 97.09 | 344.29 | 71.74 | 5.55 | 0.031 | 0.445 | 1.77 |
| HNRPA1L-2 | 2377.20 | 654.38 | 1340.83 | 631.82 | 7.73 | 0.016 | 0.449 | 1.77 |
| SOD1 | 6712.66 | 1579.44 | 3787.66 | 1536.13 | 14.19 | 0.005 | 0.448 | 1.77 |
| RECQL4 | 242.44 | 35.58 | 136.81 | 13.67 | 5.77 | 0.029 | 0.444 | 1.77 |
| PTPLAD1 | 2108.29 | 630.04 | 1189.83 | 577.80 | 26.70 | 0.001 | 0.450 | 1.77 |
| POLR2I | 986.80 | 294.75 | 556.91 | 168.47 | 5.86 | 0.028 | 0.445 | 1.77 |
| IMPA1 | 866.02 | 401.75 | 489.16 | 301.81 | 6.32 | 0.024 | 0.451 | 1.77 |
| CIP29 | 2220.03 | 758.45 | 1254.45 | 572.44 | 8.98 | 0.012 | 0.454 | 1.77 |
| EIF2B3 | 531.25 | 194.13 | 300.42 | 140.72 | 6.18 | 0.025 | 0.449 | 1.77 |
| C14ORF2 | 626.20 | 204.46 | 354.12 | 120.64 | 5.62 | 0.030 | 0.445 | 1.77 |
| H2AFX | 327.02 | 32.65 | 184.94 | 18.69 | 16.82 | 0.004 | 0.453 | 1.77 |
| BTG3 | 1034.93 | 340.13 | 585.51 | 207.35 | 4.80 | 0.041 | 0.444 | 1.77 |
| CSDA | 4272.59 | 1360.35 | 2419.03 | 825.79 | 5.80 | 0.028 | 0.444 | 1.77 |
| PARK7 | 4812.56 | 1544.26 | 2724.88 | 1043.87 | 6.80 | 0.021 | 0.448 | 1.77 |
| HMGCR | 1192.18 | 460.99 | 675.28 | 307.32 | 5.80 | 0.028 | 0.444 | 1.77 |
| DTX1 | 240.76 | 24.38 | 136.44 | 9.17 | 10.04 | 0.010 | 0.453 | 1.76 |
| LOC728139 | 3358.17 | 563.44 | 1903.34 | 620.13 | 10.61 | 0.009 | 0.453 | 1.76 |
| ABCF2 | 313.10 | 7.55 | 177.52 | 33.30 | 6.04 | 0.026 | 0.449 | 1.76 |
| SAMM50 | 1330.09 | 477.82 | 754.21 | 257.75 | 4.40 | 0.048 | 0.450 | 1.76 |
| CD320 | 345.21 | 91.77 | 195.97 | 67.62 | 10.10 | 0.010 | 0.454 | 1.76 |
| HSD17B12 | 2740.04 | 901.25 | 1556.31 | 962.35 | 13.04 | 0.006 | 0.444 | 1.76 |
| NFATC1 | 530.42 | 114.86 | 301.40 | 126.82 | 5.89 | 0.028 | 0.446 | 1.76 |
| NAP1L5 | 233.43 | 40.44 | 132.81 | 13.20 | 5.85 | 0.028 | 0.446 | 1.76 |
| LOC652545 | 405.88 | 77.11 | 231.17 | 35.74 | 7.12 | 0.019 | 0.449 | 1.76 |
| PUF60 | 2430.73 | 542.15 | 1385.33 | 487.25 | 8.12 | 0.015 | 0.452 | 1.75 |
| DDX10 | 795.23 | 267.63 | 453.32 | 153.75 | 5.08 | 0.037 | 0.446 | 1.75 |
| NSA2 | 2512.64 | 792.92 | 1433.26 | 435.61 | 4.96 | 0.038 | 0.445 | 1.75 |
| C14ORF112 | 1106.63 | 363.95 | 632.05 | 203.52 | 4.77 | 0.041 | 0.444 | 1.75 |
| VDAC3 | 5669.06 | 2071.45 | 3238.11 | 1293.91 | 5.35 | 0.033 | 0.446 | 1.75 |
| HERPUD1 | 1871.10 | 651.56 | 1069.83 | 457.23 | 6.61 | 0.022 | 0.454 | 1.75 |
| FASTKD3 | 367.21 | 67.10 | 209.97 | 35.47 | 6.56 | 0.022 | 0.454 | 1.75 |
| SSRP1 | 488.20 | 104.21 | 279.19 | 48.06 | 5.22 | 0.035 | 0.447 | 1.75 |
| WBSCR22 | 1055.53 | 309.30 | 603.77 | 252.52 | 11.18 | 0.008 | 0.455 | 1.75 |
| GRHPR | 602.63 | 143.70 | 344.81 | 98.66 | 6.35 | 0.024 | 0.451 | 1.75 |
| MRPS24 | 1872.94 | 722.13 | 1072.07 | 422.14 | 4.62 | 0.044 | 0.447 | 1.75 |
| FIBP | 1342.65 | 323.38 | 768.67 | 162.20 | 5.88 | 0.028 | 0.446 | 1.75 |
| RBBP7 | 1557.43 | 678.29 | 891.76 | 455.73 | 5.03 | 0.037 | 0.446 | 1.75 |
| RPL14 | 2138.23 | 1097.59 | 1224.67 | 824.28 | 5.26 | 0.034 | 0.448 | 1.75 |
| PSMC3IP | 213.87 | 31.25 | 122.58 | 11.38 | 5.80 | 0.028 | 0.444 | 1.74 |
| IL24 | 226.00 | 36.54 | 129.54 | 16.38 | 5.70 | 0.029 | 0.445 | 1.74 |
| ENY2 | 2372.85 | 841.63 | 1361.06 | 499.53 | 4.76 | 0.041 | 0.444 | 1.74 |
| SHPK | 893.01 | 307.66 | 512.24 | 278.87 | 10.32 | 0.009 | 0.453 | 1.74 |
| EIF3B | 426.68 | 75.15 | 244.80 | 34.14 | 5.34 | 0.033 | 0.446 | 1.74 |
| NUDCD2 | 602.53 | 249.20 | 346.05 | 157.19 | 4.74 | 0.042 | 0.445 | 1.74 |
| LOC728666 | 605.82 | 77.31 | 347.99 | 76.17 | 6.96 | 0.020 | 0.447 | 1.74 |
| SNX8 | 947.41 | 154.74 | 544.44 | 78.78 | 9.06 | 0.012 | 0.456 | 1.74 |
| PSMC3 | 2189.81 | 835.43 | 1259.06 | 524.28 | 4.58 | 0.044 | 0.448 | 1.74 |
| HIST1H2BH | 238.05 | 43.44 | 136.90 | 14.21 | 5.01 | 0.038 | 0.447 | 1.74 |
| PMM2 | 542.01 | 116.11 | 311.74 | 42.95 | 5.18 | 0.035 | 0.447 | 1.74 |
| DLAT | 741.76 | 213.12 | 426.71 | 220.32 | 10.41 | 0.009 | 0.455 | 1.74 |
| AK2 | 1541.70 | 276.95 | 887.30 | 301.86 | 45.36 | 0.000 | 0.620 | 1.74 |
| AARS | 3995.23 | 1485.07 | 2299.75 | 906.47 | 5.04 | 0.037 | 0.447 | 1.74 |
| SIGMAR1 | 292.90 | 19.11 | 168.64 | 44.17 | 4.75 | 0.042 | 0.444 | 1.74 |
| ARF4 | 445.45 | 38.86 | 256.48 | 27.07 | 13.42 | 0.006 | 0.442 | 1.74 |
| KIAA1524 | 230.21 | 58.61 | 132.56 | 29.30 | 5.74 | 0.029 | 0.445 | 1.74 |
| MRPL14 | 1391.85 | 401.80 | 801.82 | 316.72 | 10.00 | 0.010 | 0.453 | 1.74 |
| APEH | 1855.67 | 660.18 | 1069.63 | 414.27 | 5.49 | 0.032 | 0.446 | 1.73 |
| HARS | 1488.51 | 228.54 | 858.34 | 194.83 | 28.21 | 0.001 | 0.456 | 1.73 |
| ACY1 | 355.55 | 56.31 | 205.35 | 64.42 | 11.45 | 0.008 | 0.453 | 1.73 |
| EIF4H | 2031.94 | 540.39 | 1173.63 | 274.99 | 5.58 | 0.031 | 0.445 | 1.73 |
| CENPM | 261.02 | 7.23 | 150.83 | 18.56 | 13.31 | 0.006 | 0.446 | 1.73 |
| HS.555181 | 739.89 | 241.97 | 427.63 | 217.77 | 5.24 | 0.035 | 0.447 | 1.73 |
| LOC441246 | 8961.19 | 2741.50 | 5185.41 | 1535.25 | 4.32 | 0.050 | 0.452 | 1.73 |
| RSL24D1 | 3695.09 | 1342.24 | 2138.34 | 824.18 | 4.77 | 0.041 | 0.444 | 1.73 |
| SEC13 | 837.14 | 171.41 | 484.55 | 151.69 | 20.98 | 0.002 | 0.444 | 1.73 |
| LOC728312 | 239.04 | 13.31 | 138.43 | 13.16 | 34.57 | 0.001 | 0.506 | 1.73 |
| SNRPG | 10160.94 | 3125.86 | 5884.39 | 1986.04 | 5.95 | 0.027 | 0.447 | 1.73 |
| SURF6 | 891.96 | 224.64 | 516.69 | 155.38 | 5.86 | 0.028 | 0.445 | 1.73 |
| EIF4G1 | 1612.64 | 314.97 | 934.29 | 370.68 | 21.07 | 0.002 | 0.444 | 1.73 |
| LOC648740 | 550.64 | 48.52 | 319.12 | 67.81 | 5.90 | 0.028 | 0.447 | 1.73 |
| SLC25A5 | 15415.62 | 5101.95 | 8936.13 | 3891.20 | 9.25 | 0.011 | 0.457 | 1.73 |
| ARL5A | 330.25 | 74.73 | 191.49 | 20.79 | 4.46 | 0.047 | 0.450 | 1.72 |
| HNRNPD | 8734.51 | 3100.64 | 5071.65 | 1999.58 | 5.59 | 0.031 | 0.445 | 1.72 |
| CPSF4 | 2321.90 | 627.34 | 1348.38 | 446.07 | 9.30 | 0.011 | 0.461 | 1.72 |
| FIP1L1 | 636.57 | 166.03 | 369.90 | 74.95 | 4.80 | 0.041 | 0.444 | 1.72 |
| WDYHV1 | 298.30 | 85.29 | 173.41 | 47.45 | 5.71 | 0.029 | 0.445 | 1.72 |
| MRRF | 390.15 | 115.20 | 226.91 | 71.16 | 5.50 | 0.032 | 0.446 | 1.72 |
| UBAP2 | 306.37 | 66.56 | 178.26 | 25.12 | 5.29 | 0.034 | 0.447 | 1.72 |
| EIF4ENIF1 | 614.44 | 240.51 | 357.59 | 159.83 | 5.26 | 0.034 | 0.448 | 1.72 |
| NFKB1 | 5529.78 | 1928.19 | 3222.53 | 1182.78 | 5.29 | 0.034 | 0.447 | 1.72 |
| CENPM | 174.35 | 33.51 | 101.63 | 9.17 | 4.66 | 0.043 | 0.448 | 1.72 |
| LOC728037 | 333.07 | 74.98 | 194.21 | 39.21 | 6.31 | 0.024 | 0.451 | 1.71 |
| ADPRHL2 | 765.40 | 240.32 | 446.31 | 143.76 | 5.38 | 0.033 | 0.445 | 1.71 |
| NUP153 | 903.81 | 214.01 | 527.07 | 113.57 | 5.73 | 0.029 | 0.446 | 1.71 |
| CANX | 1795.72 | 481.19 | 1047.30 | 377.15 | 12.45 | 0.006 | 0.444 | 1.71 |
| BDH1 | 213.10 | 40.53 | 124.33 | 18.07 | 5.02 | 0.037 | 0.446 | 1.71 |
| PHB | 1065.36 | 395.38 | 621.71 | 248.83 | 5.03 | 0.037 | 0.447 | 1.71 |
| DAP3 | 1439.72 | 493.35 | 840.54 | 313.30 | 5.72 | 0.029 | 0.446 | 1.71 |
| CUL2 | 1431.50 | 543.01 | 836.36 | 360.75 | 5.64 | 0.030 | 0.447 | 1.71 |
| GRPEL1 | 997.36 | 317.20 | 582.91 | 297.37 | 8.65 | 0.013 | 0.451 | 1.71 |
| ZDHHC16 | 1874.08 | 585.17 | 1096.63 | 391.82 | 6.36 | 0.024 | 0.452 | 1.71 |
| OGFOD1 | 654.13 | 212.80 | 382.96 | 176.98 | 6.01 | 0.027 | 0.448 | 1.71 |
| SNHG4 | 200.45 | 33.15 | 117.36 | 13.13 | 7.01 | 0.020 | 0.448 | 1.71 |
| ARID3A | 1171.79 | 342.36 | 686.10 | 306.15 | 19.49 | 0.003 | 0.455 | 1.71 |
| OAT | 556.88 | 78.09 | 326.08 | 79.98 | 20.18 | 0.002 | 0.452 | 1.71 |
| RPS2 | 6869.03 | 709.97 | 4022.19 | 748.81 | 9.02 | 0.012 | 0.455 | 1.71 |
| RFWD3 | 500.45 | 154.65 | 293.08 | 100.30 | 5.72 | 0.029 | 0.446 | 1.71 |
| GMDS | 672.92 | 233.25 | 394.28 | 148.19 | 5.67 | 0.030 | 0.446 | 1.71 |
| NCBP1 | 1302.55 | 556.29 | 763.36 | 365.97 | 4.63 | 0.044 | 0.447 | 1.71 |
| TBCE | 892.82 | 286.17 | 523.47 | 187.38 | 6.23 | 0.025 | 0.448 | 1.71 |
| EIF4A3 | 4011.43 | 1337.15 | 2355.08 | 1282.53 | 25.68 | 0.002 | 0.436 | 1.70 |
| NTHL1 | 358.07 | 69.42 | 210.24 | 51.67 | 14.20 | 0.005 | 0.449 | 1.70 |
| HNRNPM | 5103.38 | 1459.34 | 2997.06 | 944.71 | 4.85 | 0.040 | 0.444 | 1.70 |
| NIT2 | 556.16 | 142.07 | 326.76 | 106.85 | 10.71 | 0.009 | 0.453 | 1.70 |
| ILF2 | 4524.45 | 1782.68 | 2658.41 | 1399.05 | 4.73 | 0.042 | 0.445 | 1.70 |
| EIF5B | 544.05 | 134.46 | 319.67 | 124.54 | 7.30 | 0.018 | 0.450 | 1.70 |
| RBMX | 1421.33 | 432.60 | 835.22 | 308.40 | 4.88 | 0.040 | 0.444 | 1.70 |
| PPP1R12B | 269.06 | 54.42 | 158.11 | 38.03 | 6.65 | 0.022 | 0.453 | 1.70 |
| RPUSD4 | 1935.89 | 615.20 | 1138.31 | 465.30 | 9.18 | 0.012 | 0.455 | 1.70 |
| LOC389168 | 7927.39 | 3192.86 | 4662.67 | 2162.47 | 5.39 | 0.033 | 0.445 | 1.70 |
| LOC100132457 | 3828.14 | 1433.24 | 2251.92 | 1130.40 | 6.06 | 0.026 | 0.448 | 1.70 |
| SLC39A3 | 1152.19 | 348.67 | 677.80 | 217.49 | 5.83 | 0.028 | 0.445 | 1.70 |
| LONP1 | 810.50 | 236.21 | 476.87 | 182.99 | 7.16 | 0.019 | 0.451 | 1.70 |
| ENDOG | 278.54 | 28.16 | 163.92 | 23.67 | 7.77 | 0.016 | 0.449 | 1.70 |
| ELAC2 | 361.44 | 92.01 | 212.72 | 44.51 | 5.30 | 0.034 | 0.447 | 1.70 |
| LOC728698 | 2160.63 | 738.02 | 1272.41 | 686.08 | 6.14 | 0.025 | 0.449 | 1.70 |
| NT5DC2 | 250.41 | 33.40 | 147.56 | 13.72 | 5.47 | 0.032 | 0.445 | 1.70 |
| IARS2 | 1890.58 | 548.95 | 1114.14 | 473.64 | 5.18 | 0.035 | 0.447 | 1.70 |
| TBC1D13 | 345.97 | 61.71 | 204.20 | 41.54 | 9.95 | 0.010 | 0.456 | 1.69 |
| ZNF207 | 1951.37 | 629.36 | 1152.54 | 605.90 | 16.39 | 0.004 | 0.452 | 1.69 |
| SLC25A3 | 4650.85 | 1242.13 | 2747.58 | 825.68 | 5.48 | 0.032 | 0.445 | 1.69 |
| NUDT3 | 868.49 | 175.08 | 513.24 | 129.70 | 10.12 | 0.010 | 0.454 | 1.69 |
| LOC100128805 | 539.22 | 168.49 | 318.77 | 94.84 | 5.09 | 0.037 | 0.446 | 1.69 |
| ABCF1 | 1117.63 | 353.28 | 661.57 | 196.39 | 5.03 | 0.037 | 0.447 | 1.69 |
| ALG13 | 695.73 | 210.62 | 412.33 | 154.53 | 5.44 | 0.032 | 0.445 | 1.69 |
| FOXM1 | 250.34 | 32.08 | 148.43 | 15.08 | 10.36 | 0.009 | 0.453 | 1.69 |
| TMEM177 | 307.19 | 51.66 | 182.15 | 28.01 | 9.13 | 0.012 | 0.457 | 1.69 |
| CYC1 | 2634.76 | 694.03 | 1563.74 | 659.57 | 10.40 | 0.009 | 0.454 | 1.68 |
| SPIB | 720.50 | 135.22 | 427.70 | 193.81 | 8.55 | 0.013 | 0.451 | 1.68 |
| QARS | 3566.76 | 1186.09 | 2117.61 | 752.56 | 5.59 | 0.031 | 0.445 | 1.68 |
| C16ORF75 | 773.01 | 191.06 | 459.13 | 202.46 | 15.34 | 0.004 | 0.450 | 1.68 |
| C6ORF66 | 388.31 | 112.67 | 231.03 | 68.33 | 4.90 | 0.039 | 0.444 | 1.68 |
| THOC5 | 478.78 | 123.05 | 284.99 | 67.36 | 4.86 | 0.040 | 0.445 | 1.68 |
| ARPP19 | 485.03 | 24.86 | 289.01 | 21.69 | 37.96 | 0.001 | 0.504 | 1.68 |
| COPS5 | 1745.59 | 466.11 | 1041.57 | 323.92 | 5.84 | 0.028 | 0.446 | 1.68 |
| NEFH | 265.56 | 29.91 | 158.47 | 24.34 | 19.88 | 0.003 | 0.450 | 1.68 |
| PSMD1 | 1142.02 | 303.46 | 682.09 | 226.46 | 10.20 | 0.009 | 0.452 | 1.67 |
| SFRS10 | 2587.50 | 971.17 | 1546.20 | 666.73 | 5.25 | 0.034 | 0.447 | 1.67 |
| RRP8 | 635.71 | 198.26 | 379.95 | 149.62 | 8.78 | 0.013 | 0.457 | 1.67 |
| LOC440145 | 334.07 | 111.88 | 199.71 | 64.49 | 4.68 | 0.043 | 0.447 | 1.67 |
| HAT1 | 1778.90 | 844.97 | 1064.43 | 720.69 | 4.78 | 0.041 | 0.444 | 1.67 |
| STRADB | 465.06 | 129.81 | 278.96 | 93.65 | 8.72 | 0.013 | 0.454 | 1.67 |
| LOC646630 | 8504.02 | 2918.61 | 5102.01 | 2193.64 | 7.84 | 0.016 | 0.450 | 1.67 |
| ITGB4BP | 1940.20 | 334.05 | 1164.39 | 391.97 | 9.20 | 0.012 | 0.457 | 1.67 |
| SRP72 | 1014.60 | 285.42 | 609.01 | 248.98 | 13.02 | 0.006 | 0.443 | 1.67 |
| TEX10 | 418.25 | 117.62 | 251.25 | 77.07 | 6.71 | 0.021 | 0.450 | 1.66 |
| DDX19B | 1057.76 | 283.12 | 635.58 | 171.08 | 4.56 | 0.045 | 0.449 | 1.66 |
| PRAGMIN | 1720.65 | 688.63 | 1034.02 | 438.67 | 4.73 | 0.042 | 0.445 | 1.66 |
| HSPA4 | 1350.48 | 607.50 | 811.75 | 572.94 | 4.93 | 0.039 | 0.446 | 1.66 |
| ZNF207 | 2836.21 | 1277.60 | 1705.07 | 835.81 | 4.43 | 0.047 | 0.450 | 1.66 |
| ATP5J | 3608.33 | 950.75 | 2169.25 | 842.83 | 20.68 | 0.002 | 0.437 | 1.66 |
| DNM1L | 507.62 | 167.49 | 305.24 | 123.13 | 6.26 | 0.025 | 0.449 | 1.66 |
| CHCHD2 | 8667.67 | 2364.68 | 5215.82 | 1866.82 | 12.00 | 0.007 | 0.445 | 1.66 |
| C16ORF91 | 430.46 | 100.22 | 259.18 | 68.02 | 9.10 | 0.012 | 0.457 | 1.66 |
| SKIV2L2 | 1050.45 | 422.03 | 632.60 | 277.74 | 4.70 | 0.042 | 0.447 | 1.66 |
| HSPA8 | 14155.66 | 4344.28 | 8526.61 | 3533.22 | 4.54 | 0.045 | 0.449 | 1.66 |
| DHX30 | 388.14 | 108.54 | 233.84 | 59.19 | 5.23 | 0.035 | 0.448 | 1.66 |
| NAP1L1 | 2385.56 | 888.01 | 1438.49 | 720.40 | 6.18 | 0.025 | 0.448 | 1.66 |
| POLR2G | 1880.52 | 545.42 | 1134.36 | 400.59 | 8.74 | 0.013 | 0.456 | 1.66 |
| LOC100129828 | 388.04 | 83.19 | 234.39 | 70.43 | 4.72 | 0.042 | 0.445 | 1.66 |
| TBRG4 | 831.48 | 279.09 | 502.58 | 190.19 | 6.38 | 0.024 | 0.453 | 1.65 |
| MED10 | 1213.39 | 365.57 | 733.73 | 297.79 | 7.79 | 0.016 | 0.449 | 1.65 |
| XRCC3 | 285.37 | 32.46 | 172.57 | 16.87 | 11.56 | 0.007 | 0.448 | 1.65 |
| RPSA | 13227.92 | 4422.66 | 8004.05 | 2949.00 | 4.40 | 0.048 | 0.451 | 1.65 |
| TIAL1 | 1732.83 | 541.74 | 1049.14 | 356.59 | 6.34 | 0.024 | 0.451 | 1.65 |
| ANP32B | 10710.99 | 3584.44 | 6485.40 | 2360.34 | 5.20 | 0.035 | 0.447 | 1.65 |
| EXOSC4 | 307.35 | 41.31 | 186.28 | 25.50 | 7.67 | 0.017 | 0.447 | 1.65 |
| DCPS | 378.17 | 46.94 | 229.23 | 45.23 | 6.82 | 0.021 | 0.448 | 1.65 |
| FAM158A | 278.32 | 31.63 | 168.71 | 25.38 | 13.90 | 0.005 | 0.451 | 1.65 |
| C6ORF115 | 2059.71 | 841.70 | 1249.51 | 638.34 | 5.66 | 0.030 | 0.446 | 1.65 |
| LOC646483 | 4007.09 | 1000.47 | 2434.32 | 941.36 | 11.14 | 0.008 | 0.456 | 1.65 |
| TARS | 2108.28 | 1014.35 | 1281.10 | 733.62 | 4.36 | 0.049 | 0.452 | 1.65 |
| LOC151579 | 2499.84 | 1007.87 | 1519.96 | 813.42 | 5.78 | 0.029 | 0.444 | 1.64 |
| TUFM | 1861.23 | 618.04 | 1132.46 | 490.40 | 5.04 | 0.037 | 0.447 | 1.64 |
| CEBPZ | 2342.18 | 721.35 | 1425.35 | 593.11 | 5.65 | 0.030 | 0.447 | 1.64 |
| LOC644879 | 346.80 | 11.60 | 211.24 | 55.40 | 4.81 | 0.041 | 0.445 | 1.64 |
| SNRPD2 | 12861.40 | 3826.55 | 7836.18 | 2661.01 | 5.93 | 0.027 | 0.448 | 1.64 |
| BCLAF1 | 780.07 | 257.75 | 475.48 | 179.43 | 6.67 | 0.022 | 0.452 | 1.64 |
| WDR4 | 251.54 | 20.13 | 153.65 | 30.83 | 14.38 | 0.005 | 0.445 | 1.64 |
| SNHG5 | 1762.75 | 660.72 | 1076.76 | 429.76 | 5.04 | 0.037 | 0.447 | 1.64 |
| BOLA2 | 4331.40 | 723.33 | 2647.21 | 589.74 | 21.78 | 0.002 | 0.452 | 1.64 |
| ZNF828 | 1195.18 | 345.84 | 730.66 | 223.32 | 4.86 | 0.040 | 0.444 | 1.64 |
| LOC728128 | 11766.40 | 4047.61 | 7195.56 | 2584.17 | 5.39 | 0.033 | 0.445 | 1.64 |
| MRPS11 | 468.02 | 114.03 | 286.23 | 84.30 | 9.24 | 0.012 | 0.456 | 1.64 |
| LOC730246 | 14682.82 | 3480.70 | 8985.91 | 3675.77 | 24.67 | 0.002 | 0.433 | 1.63 |
| ATP5F1 | 5736.02 | 2026.81 | 3513.49 | 1149.26 | 4.38 | 0.048 | 0.451 | 1.63 |
| WDR74 | 750.71 | 207.44 | 460.00 | 136.67 | 7.08 | 0.019 | 0.450 | 1.63 |
| BNIP1 | 227.78 | 66.16 | 139.80 | 35.39 | 4.59 | 0.044 | 0.448 | 1.63 |
| EIF4G1 | 924.59 | 197.47 | 567.85 | 176.71 | 27.16 | 0.001 | 0.447 | 1.63 |
| PSME3 | 882.97 | 272.83 | 542.44 | 227.72 | 7.55 | 0.017 | 0.446 | 1.63 |
| LOC439953 | 12913.18 | 3940.83 | 7936.70 | 3365.53 | 8.19 | 0.015 | 0.452 | 1.63 |
| TOE1 | 282.44 | 78.17 | 173.73 | 57.23 | 8.99 | 0.012 | 0.454 | 1.63 |
| EEF2 | 10579.08 | 3034.18 | 6511.93 | 2093.68 | 6.06 | 0.026 | 0.448 | 1.62 |
| CAD | 403.07 | 86.50 | 248.12 | 58.61 | 7.25 | 0.019 | 0.450 | 1.62 |
| PGK1 | 2101.57 | 824.39 | 1294.07 | 662.22 | 7.95 | 0.015 | 0.453 | 1.62 |
| IFRD2 | 293.49 | 58.98 | 180.73 | 22.44 | 5.31 | 0.034 | 0.447 | 1.62 |
| CMC1 | 569.48 | 193.67 | 350.70 | 116.72 | 4.84 | 0.040 | 0.444 | 1.62 |
| C19ORF2 | 3089.14 | 983.95 | 1902.99 | 670.40 | 4.90 | 0.039 | 0.444 | 1.62 |
| LOC286444 | 8528.69 | 501.23 | 5258.06 | 1503.22 | 5.33 | 0.033 | 0.447 | 1.62 |
| APRT | 694.22 | 27.62 | 428.05 | 55.15 | 12.54 | 0.006 | 0.442 | 1.62 |
| LOC728031 | 5413.27 | 1790.61 | 3339.41 | 1240.28 | 6.19 | 0.025 | 0.449 | 1.62 |
| LOC644877 | 1045.70 | 485.59 | 645.21 | 423.24 | 5.16 | 0.036 | 0.447 | 1.62 |
| GTF3C2 | 578.01 | 183.69 | 356.72 | 113.84 | 5.35 | 0.033 | 0.446 | 1.62 |
| GEMIN5 | 294.25 | 29.73 | 181.63 | 21.20 | 16.72 | 0.004 | 0.455 | 1.62 |
| TTC1 | 542.61 | 83.02 | 335.09 | 108.36 | 12.90 | 0.006 | 0.440 | 1.62 |
| ENSA | 318.97 | 62.82 | 197.00 | 32.55 | 5.76 | 0.029 | 0.445 | 1.62 |
| CD19 | 2928.22 | 1119.33 | 1810.65 | 852.06 | 4.34 | 0.049 | 0.452 | 1.62 |
| TMEM118 | 271.12 | 67.27 | 167.72 | 39.14 | 5.46 | 0.032 | 0.445 | 1.62 |
| DPP3 | 470.36 | 105.04 | 291.03 | 101.98 | 13.54 | 0.005 | 0.446 | 1.62 |
| ACN9 | 316.74 | 80.93 | 196.00 | 43.91 | 4.41 | 0.048 | 0.450 | 1.62 |
| JOSD1 | 902.27 | 356.64 | 558.66 | 253.82 | 5.60 | 0.030 | 0.445 | 1.62 |
| WDR33 | 1250.80 | 345.98 | 774.53 | 353.26 | 77.30 | 0.000 | 0.791 | 1.61 |
| NOL7 | 4282.66 | 1630.86 | 2654.82 | 1090.10 | 5.21 | 0.035 | 0.447 | 1.61 |
| CUL4A | 1027.97 | 363.74 | 638.10 | 235.60 | 4.90 | 0.039 | 0.444 | 1.61 |
| TDG | 857.53 | 297.72 | 532.66 | 210.21 | 6.43 | 0.023 | 0.453 | 1.61 |
| LOC646531 | 8875.58 | 1572.41 | 5519.32 | 1064.46 | 6.55 | 0.023 | 0.454 | 1.61 |
| BTLA | 519.31 | 184.50 | 323.08 | 113.25 | 4.68 | 0.043 | 0.447 | 1.61 |
| KCNG1 | 227.82 | 38.38 | 141.76 | 10.64 | 4.80 | 0.041 | 0.444 | 1.61 |
| ZNF142 | 760.76 | 204.97 | 473.41 | 123.86 | 4.70 | 0.042 | 0.447 | 1.61 |
| LOC729342 | 262.62 | 25.50 | 163.50 | 26.45 | 21.81 | 0.002 | 0.455 | 1.61 |
| C8ORF33 | 573.57 | 118.29 | 357.61 | 78.65 | 7.07 | 0.019 | 0.449 | 1.60 |
| EWSR1 | 792.05 | 323.92 | 494.34 | 227.65 | 4.95 | 0.039 | 0.446 | 1.60 |
| FARSA | 497.10 | 78.92 | 310.26 | 71.71 | 6.61 | 0.022 | 0.453 | 1.60 |
| TFB1M | 480.99 | 97.39 | 300.32 | 109.60 | 12.90 | 0.006 | 0.439 | 1.60 |
| MRPS7 | 1043.54 | 374.53 | 651.66 | 300.35 | 4.58 | 0.045 | 0.448 | 1.60 |
| DHX29 | 1057.04 | 324.27 | 660.43 | 201.69 | 4.48 | 0.046 | 0.449 | 1.60 |
| MAT2A | 4947.49 | 1913.51 | 3091.90 | 1345.68 | 5.03 | 0.037 | 0.447 | 1.60 |
| SMARCA4 | 1108.91 | 310.07 | 693.36 | 233.25 | 4.34 | 0.049 | 0.452 | 1.60 |
| BOLA2 | 2114.03 | 679.05 | 1323.84 | 388.21 | 4.35 | 0.049 | 0.452 | 1.60 |
| LOC389141 | 13530.94 | 4766.61 | 8476.00 | 3808.82 | 5.55 | 0.031 | 0.445 | 1.60 |
| TXLNA | 2240.30 | 970.43 | 1403.60 | 636.58 | 4.34 | 0.049 | 0.452 | 1.60 |
| PPP1CC | 6575.89 | 2740.47 | 4122.90 | 2182.17 | 6.31 | 0.024 | 0.451 | 1.59 |
| RHOG | 2668.55 | 74.73 | 1674.39 | 341.79 | 6.20 | 0.025 | 0.449 | 1.59 |
| GTF2H3 | 406.18 | 101.57 | 254.95 | 69.72 | 5.29 | 0.034 | 0.447 | 1.59 |
| RPS17 | 486.47 | 89.41 | 305.41 | 93.69 | 61.24 | 0.000 | 0.788 | 1.59 |
| CHPT1 | 759.22 | 179.36 | 476.70 | 110.83 | 5.32 | 0.034 | 0.447 | 1.59 |
| FTSJ3 | 335.27 | 67.92 | 210.62 | 36.14 | 5.48 | 0.032 | 0.445 | 1.59 |
| RNF4 | 562.81 | 152.25 | 353.65 | 87.42 | 4.59 | 0.044 | 0.447 | 1.59 |
| DDX19A | 447.17 | 109.01 | 281.01 | 66.43 | 6.48 | 0.023 | 0.452 | 1.59 |
| APRT | 1388.36 | 218.12 | 872.75 | 218.79 | 13.33 | 0.006 | 0.446 | 1.59 |
| LOC729774 | 240.52 | 33.37 | 151.32 | 17.51 | 8.03 | 0.015 | 0.452 | 1.59 |
| SMARCD1 | 1922.81 | 636.18 | 1209.75 | 542.48 | 6.90 | 0.020 | 0.446 | 1.59 |
| PSD4 | 700.82 | 165.79 | 441.06 | 105.24 | 5.80 | 0.028 | 0.444 | 1.59 |
| SCNM1 | 314.08 | 66.21 | 197.72 | 28.66 | 4.73 | 0.042 | 0.445 | 1.59 |
| SFRS2 | 6248.27 | 2322.88 | 3934.32 | 1653.63 | 5.04 | 0.037 | 0.447 | 1.59 |
| ATP5C1 | 1869.31 | 670.21 | 1177.09 | 588.25 | 9.85 | 0.010 | 0.458 | 1.59 |
| DDB2 | 315.29 | 77.01 | 198.85 | 42.07 | 5.59 | 0.031 | 0.445 | 1.59 |
| LOC389322 | 1378.54 | 346.55 | 869.57 | 415.07 | 10.75 | 0.009 | 0.454 | 1.59 |
| CLEC2D | 20583.31 | 8193.46 | 12991.05 | 6152.96 | 6.18 | 0.025 | 0.448 | 1.58 |
| GNL3L | 994.20 | 200.15 | 627.69 | 116.82 | 7.19 | 0.019 | 0.450 | 1.58 |
| C17ORF96 | 713.41 | 152.28 | 450.71 | 179.10 | 4.58 | 0.044 | 0.448 | 1.58 |
| HK1 | 5326.13 | 1817.05 | 3365.75 | 1435.15 | 7.25 | 0.019 | 0.450 | 1.58 |
| LOC389137 | 395.37 | 89.62 | 250.01 | 43.76 | 5.22 | 0.035 | 0.447 | 1.58 |
| LOC100130980 | 21134.34 | 4302.78 | 13367.02 | 3181.84 | 5.48 | 0.032 | 0.445 | 1.58 |
| CS | 444.98 | 25.03 | 281.69 | 37.06 | 9.81 | 0.010 | 0.457 | 1.58 |
| MRPS22 | 1360.21 | 438.06 | 861.19 | 376.19 | 13.78 | 0.005 | 0.449 | 1.58 |
| ZNF259 | 428.39 | 111.99 | 271.25 | 91.13 | 7.84 | 0.016 | 0.450 | 1.58 |
| NONO | 328.61 | 8.86 | 208.25 | 15.91 | 29.45 | 0.001 | 0.457 | 1.58 |
| RPL12 | 4340.63 | 944.19 | 2751.41 | 729.70 | 10.68 | 0.009 | 0.454 | 1.58 |
| MRPL24 | 507.68 | 130.81 | 321.86 | 71.50 | 4.52 | 0.046 | 0.448 | 1.58 |
| DSN1 | 362.80 | 114.71 | 230.06 | 80.12 | 6.63 | 0.022 | 0.453 | 1.58 |
| IDI1 | 960.97 | 435.95 | 609.46 | 373.93 | 5.43 | 0.032 | 0.445 | 1.58 |
| UBIAD1 | 717.19 | 245.72 | 454.89 | 156.57 | 4.70 | 0.042 | 0.447 | 1.58 |
| PPIAL4A | 12642.40 | 4788.32 | 8021.78 | 3518.35 | 5.01 | 0.038 | 0.447 | 1.58 |
| RDBP | 439.60 | 76.07 | 279.24 | 49.37 | 6.31 | 0.024 | 0.451 | 1.57 |
| C16ORF53 | 338.71 | 52.38 | 215.15 | 34.20 | 11.04 | 0.008 | 0.457 | 1.57 |
| RAB8A | 3505.71 | 1026.33 | 2228.67 | 1104.17 | 25.26 | 0.002 | 0.435 | 1.57 |
| NME1-NME2 | 209.68 | 23.47 | 133.43 | 1.61 | 6.04 | 0.026 | 0.448 | 1.57 |
| NAP1L1 | 948.91 | 313.72 | 603.97 | 299.28 | 5.94 | 0.027 | 0.447 | 1.57 |
| PUS1 | 362.44 | 37.97 | 230.86 | 65.34 | 6.45 | 0.023 | 0.453 | 1.57 |
| GATAD2A | 2395.01 | 740.22 | 1526.01 | 651.62 | 13.54 | 0.005 | 0.447 | 1.57 |
| HMGN2 | 5865.06 | 1925.82 | 3738.33 | 1687.16 | 12.43 | 0.006 | 0.445 | 1.57 |
| LOC388524 | 21648.96 | 4327.68 | 13799.77 | 3437.86 | 4.69 | 0.043 | 0.447 | 1.57 |
| DDX47 | 3010.56 | 1145.27 | 1919.59 | 761.26 | 4.90 | 0.039 | 0.444 | 1.57 |
| ARHGDIA | 2207.09 | 85.19 | 1407.31 | 173.83 | 7.08 | 0.019 | 0.449 | 1.57 |
| RAP1GDS1 | 498.18 | 123.51 | 317.79 | 150.09 | 11.75 | 0.007 | 0.448 | 1.57 |
| DARS2 | 582.17 | 230.24 | 371.38 | 180.20 | 7.28 | 0.018 | 0.449 | 1.57 |
| TAGLN2 | 2022.42 | 172.58 | 1290.56 | 211.60 | 23.18 | 0.002 | 0.452 | 1.57 |
| NUP62 | 3957.57 | 1352.00 | 2526.80 | 917.42 | 4.49 | 0.046 | 0.449 | 1.57 |
| EIF3E | 9150.01 | 3381.51 | 5842.40 | 2220.37 | 4.93 | 0.039 | 0.445 | 1.57 |
| DHFR | 198.75 | 8.97 | 126.97 | 13.15 | 9.12 | 0.012 | 0.456 | 1.57 |
| EHBP1 | 396.35 | 112.43 | 253.22 | 80.51 | 5.16 | 0.036 | 0.447 | 1.57 |
| LOC729742 | 2583.39 | 805.43 | 1650.50 | 538.42 | 6.02 | 0.026 | 0.449 | 1.57 |
| RPL31 | 2687.44 | 894.92 | 1719.27 | 710.89 | 5.25 | 0.034 | 0.447 | 1.56 |
| LOC729769 | 1550.44 | 434.70 | 992.38 | 332.65 | 8.83 | 0.013 | 0.456 | 1.56 |
| GMPPB | 501.35 | 118.64 | 320.98 | 76.65 | 7.09 | 0.019 | 0.450 | 1.56 |
| API5 | 2059.33 | 584.40 | 1318.63 | 502.04 | 8.40 | 0.014 | 0.452 | 1.56 |
| FAM136B | 240.95 | 38.61 | 154.49 | 11.91 | 4.91 | 0.039 | 0.445 | 1.56 |
| UQCC | 284.29 | 44.50 | 182.36 | 16.45 | 6.29 | 0.024 | 0.450 | 1.56 |
| LOC728368 | 20387.15 | 7044.98 | 13084.48 | 4955.49 | 4.95 | 0.039 | 0.446 | 1.56 |
| DEXI | 687.77 | 251.83 | 441.56 | 162.47 | 4.73 | 0.042 | 0.445 | 1.56 |
| CTNNAL1 | 456.12 | 172.25 | 292.91 | 165.56 | 11.36 | 0.008 | 0.451 | 1.56 |
| C11ORF73 | 438.77 | 113.21 | 281.78 | 66.77 | 5.81 | 0.028 | 0.444 | 1.56 |
| CYB5A | 960.19 | 293.31 | 616.79 | 302.48 | 5.43 | 0.032 | 0.444 | 1.56 |
| LOC646294 | 6623.92 | 1821.32 | 4256.69 | 1119.73 | 4.61 | 0.044 | 0.448 | 1.56 |
| RABGGTB | 916.37 | 297.09 | 589.70 | 272.67 | 15.83 | 0.004 | 0.452 | 1.55 |
| MRPL44 | 769.63 | 337.42 | 495.42 | 256.48 | 5.12 | 0.036 | 0.447 | 1.55 |
| PSMA5 | 7020.51 | 2127.10 | 4520.56 | 1906.86 | 14.75 | 0.005 | 0.451 | 1.55 |
| POLD1 | 520.12 | 165.29 | 334.95 | 163.24 | 78.07 | 0.000 | 0.862 | 1.55 |
| PSMD4 | 891.44 | 303.80 | 574.10 | 196.38 | 5.11 | 0.036 | 0.447 | 1.55 |
| MAGMAS | 794.91 | 260.56 | 512.02 | 172.80 | 5.10 | 0.036 | 0.447 | 1.55 |
| INTS6 | 1190.08 | 440.18 | 766.91 | 381.35 | 12.34 | 0.007 | 0.441 | 1.55 |
| LETM1 | 290.56 | 49.19 | 187.50 | 19.73 | 5.94 | 0.027 | 0.447 | 1.55 |
| ICT1 | 625.84 | 147.22 | 404.06 | 127.95 | 15.30 | 0.004 | 0.450 | 1.55 |
| TMEM93 | 1737.67 | 492.05 | 1122.47 | 480.30 | 21.50 | 0.002 | 0.451 | 1.55 |
| KHDRBS1 | 4245.57 | 1207.23 | 2743.02 | 1160.05 | 18.74 | 0.003 | 0.454 | 1.55 |
| LOC729926 | 2251.31 | 834.43 | 1457.48 | 531.02 | 4.53 | 0.045 | 0.449 | 1.54 |
| LOC731878 | 645.99 | 145.94 | 418.56 | 103.84 | 8.04 | 0.015 | 0.453 | 1.54 |
| MID1IP1 | 499.03 | 33.39 | 323.41 | 44.97 | 20.13 | 0.002 | 0.452 | 1.54 |
| MYO19 | 205.85 | 22.35 | 133.45 | 11.26 | 7.68 | 0.017 | 0.448 | 1.54 |
| ABI2 | 356.98 | 98.74 | 231.47 | 66.37 | 6.29 | 0.024 | 0.449 | 1.54 |
| HTRA2 | 341.13 | 38.60 | 221.30 | 29.51 | 8.56 | 0.013 | 0.451 | 1.54 |
| YIF1B | 285.75 | 40.39 | 185.42 | 25.06 | 4.51 | 0.046 | 0.448 | 1.54 |
| FAF1 | 604.32 | 197.61 | 392.33 | 115.77 | 4.46 | 0.047 | 0.449 | 1.54 |
| SLC29A2 | 282.59 | 71.03 | 183.52 | 36.36 | 4.49 | 0.046 | 0.449 | 1.54 |
| EIF1AX | 1096.01 | 471.26 | 711.85 | 438.09 | 7.09 | 0.019 | 0.450 | 1.54 |
| ATPAF1 | 741.76 | 162.71 | 482.09 | 146.77 | 12.33 | 0.007 | 0.440 | 1.54 |
| TRNT1 | 226.58 | 26.79 | 147.29 | 20.26 | 6.97 | 0.020 | 0.447 | 1.54 |
| BANF1 | 2030.28 | 142.81 | 1320.49 | 89.73 | 15.94 | 0.004 | 0.453 | 1.54 |
| MDH2 | 9950.62 | 3632.24 | 6474.52 | 2270.43 | 4.42 | 0.048 | 0.450 | 1.54 |
| TBCA | 4255.20 | 1595.59 | 2769.13 | 1029.32 | 4.43 | 0.047 | 0.449 | 1.54 |
| CSNK2A2 | 479.14 | 147.43 | 311.93 | 97.36 | 5.58 | 0.031 | 0.445 | 1.54 |
| WDYHV1 | 260.73 | 51.06 | 169.87 | 24.42 | 5.34 | 0.033 | 0.446 | 1.53 |
| TSTA3 | 291.63 | 33.38 | 190.04 | 23.07 | 6.85 | 0.021 | 0.447 | 1.53 |
| ORC3L | 540.73 | 137.34 | 352.52 | 84.88 | 5.39 | 0.033 | 0.445 | 1.53 |
| SELK | 581.11 | 128.60 | 379.04 | 99.54 | 6.47 | 0.023 | 0.453 | 1.53 |
| PPP2CA | 1271.80 | 480.63 | 829.58 | 358.33 | 6.17 | 0.025 | 0.448 | 1.53 |
| EXOSC5 | 258.93 | 15.18 | 168.90 | 23.27 | 17.99 | 0.003 | 0.448 | 1.53 |
| NDUFB6 | 680.43 | 200.45 | 443.87 | 151.64 | 8.36 | 0.014 | 0.452 | 1.53 |
| TXNDC5 | 1500.82 | 511.32 | 979.06 | 434.80 | 5.03 | 0.037 | 0.446 | 1.53 |
| PTBP1 | 6950.62 | 1958.80 | 4534.47 | 1855.37 | 9.27 | 0.011 | 0.459 | 1.53 |
| CORO1A | 2954.73 | 708.49 | 1927.69 | 747.42 | 13.75 | 0.005 | 0.447 | 1.53 |
| ATP5J | 335.07 | 91.28 | 218.61 | 55.91 | 4.38 | 0.048 | 0.451 | 1.53 |
| WDR21A | 250.45 | 38.50 | 163.42 | 36.89 | 14.69 | 0.005 | 0.446 | 1.53 |
| MSH3 | 298.65 | 87.14 | 194.96 | 50.90 | 4.94 | 0.039 | 0.445 | 1.53 |
| RPA2 | 2779.17 | 1087.24 | 1814.59 | 807.50 | 4.98 | 0.038 | 0.447 | 1.53 |
| TCF4 | 733.05 | 197.74 | 478.86 | 199.24 | 10.21 | 0.009 | 0.452 | 1.53 |
| C19ORF48 | 188.24 | 8.33 | 122.98 | 9.76 | 8.06 | 0.015 | 0.453 | 1.53 |
| C9ORF46 | 1166.86 | 227.91 | 762.64 | 260.73 | 9.59 | 0.011 | 0.464 | 1.53 |
| C2ORF49 | 305.88 | 70.69 | 199.95 | 55.02 | 11.71 | 0.007 | 0.450 | 1.53 |
| HSP90AA1 | 19064.76 | 6285.06 | 12462.92 | 5058.27 | 8.17 | 0.015 | 0.451 | 1.53 |
| CYB5A | 426.49 | 126.60 | 279.02 | 69.88 | 4.50 | 0.046 | 0.449 | 1.53 |
| SLC35A4 | 487.23 | 49.63 | 319.03 | 47.08 | 66.55 | 0.000 | 0.762 | 1.53 |
| ELOF1 | 412.75 | 51.05 | 270.38 | 50.72 | 19.93 | 0.003 | 0.449 | 1.53 |
| LOC100128086 | 511.94 | 137.25 | 335.42 | 94.10 | 5.57 | 0.031 | 0.445 | 1.53 |
| FAHD2B | 335.48 | 71.56 | 219.84 | 50.84 | 8.18 | 0.015 | 0.452 | 1.53 |
| SLC35A2 | 546.32 | 161.74 | 358.03 | 127.57 | 8.46 | 0.014 | 0.454 | 1.53 |
| BUB3 | 3823.51 | 1200.28 | 2507.46 | 702.29 | 4.41 | 0.048 | 0.450 | 1.52 |
| NDUFV1 | 1325.55 | 388.81 | 869.51 | 318.32 | 5.60 | 0.030 | 0.445 | 1.52 |
| LOC642197 | 654.42 | 274.72 | 429.52 | 222.55 | 5.22 | 0.035 | 0.447 | 1.52 |
| ERI3 | 370.48 | 91.26 | 243.27 | 40.27 | 4.32 | 0.050 | 0.452 | 1.52 |
| ROMO1 | 2276.31 | 316.28 | 1495.33 | 228.70 | 14.73 | 0.005 | 0.450 | 1.52 |
| VPS24 | 211.61 | 50.36 | 139.01 | 25.04 | 4.53 | 0.045 | 0.449 | 1.52 |
| AARSD1 | 375.69 | 81.00 | 246.88 | 54.53 | 7.91 | 0.016 | 0.451 | 1.52 |
| TACO1 | 630.44 | 175.32 | 414.73 | 133.91 | 7.81 | 0.016 | 0.449 | 1.52 |
| HYAL3 | 213.77 | 35.86 | 140.68 | 15.00 | 5.02 | 0.037 | 0.446 | 1.52 |
| MIR586 | 213.15 | 24.07 | 140.30 | 18.03 | 19.62 | 0.003 | 0.456 | 1.52 |
| RPL4 | 6928.40 | 2144.34 | 4563.17 | 1460.67 | 4.39 | 0.048 | 0.451 | 1.52 |
| ATP5L | 3925.51 | 862.15 | 2585.57 | 808.17 | 42.59 | 0.001 | 0.635 | 1.52 |
| WDR74 | 464.67 | 103.06 | 306.11 | 68.95 | 7.10 | 0.019 | 0.449 | 1.52 |
| APBB1IP | 1902.67 | 287.17 | 1253.57 | 334.83 | 6.27 | 0.025 | 0.449 | 1.52 |
| EIF2B5 | 621.82 | 164.69 | 409.81 | 104.80 | 4.96 | 0.038 | 0.446 | 1.52 |
| RPS9 | 4557.37 | 746.60 | 3003.67 | 743.58 | 11.64 | 0.007 | 0.449 | 1.52 |
| MTMR14 | 1983.95 | 563.02 | 1307.63 | 362.98 | 5.07 | 0.037 | 0.447 | 1.52 |
| CHCHD10 | 2905.93 | 689.49 | 1915.42 | 616.03 | 4.64 | 0.043 | 0.447 | 1.52 |
| LOC100132051 | 171.53 | 19.58 | 113.10 | 3.56 | 5.87 | 0.028 | 0.445 | 1.52 |
| PCGF6 | 316.90 | 133.48 | 208.96 | 109.26 | 5.66 | 0.030 | 0.446 | 1.52 |
| SNX11 | 681.19 | 186.03 | 449.24 | 198.53 | 25.67 | 0.002 | 0.434 | 1.52 |
| NDUFB7 | 1217.17 | 151.82 | 802.77 | 216.05 | 8.77 | 0.013 | 0.457 | 1.52 |
| SLC41A1 | 257.61 | 67.51 | 169.92 | 45.49 | 6.82 | 0.021 | 0.448 | 1.52 |
| PKMYT1 | 173.47 | 8.67 | 114.45 | 5.31 | 7.33 | 0.018 | 0.450 | 1.52 |
| MMADHC | 1594.57 | 713.94 | 1052.40 | 554.23 | 4.95 | 0.039 | 0.446 | 1.52 |
| NFATC1 | 248.59 | 13.86 | 164.16 | 8.51 | 22.02 | 0.002 | 0.450 | 1.51 |
| NDUFS3 | 2863.73 | 587.49 | 1891.28 | 711.56 | 10.53 | 0.009 | 0.455 | 1.51 |
| SAR1B | 1666.46 | 614.08 | 1100.60 | 491.20 | 5.46 | 0.032 | 0.445 | 1.51 |
| LOC653162 | 20256.74 | 5594.03 | 13391.44 | 4300.60 | 9.19 | 0.012 | 0.456 | 1.51 |
| HAUS8 | 272.29 | 73.80 | 180.02 | 41.83 | 4.96 | 0.038 | 0.446 | 1.51 |
| STAG1 | 630.63 | 212.57 | 416.98 | 159.82 | 5.14 | 0.036 | 0.446 | 1.51 |
| PCK2 | 1084.15 | 273.50 | 716.91 | 277.50 | 5.17 | 0.035 | 0.447 | 1.51 |
| GTF2E2 | 2181.43 | 879.96 | 1443.11 | 620.73 | 4.93 | 0.039 | 0.445 | 1.51 |
| C1ORF52 | 723.23 | 206.11 | 478.52 | 185.82 | 20.87 | 0.002 | 0.440 | 1.51 |
| PDIA5 | 247.61 | 30.04 | 163.96 | 23.54 | 20.88 | 0.002 | 0.443 | 1.51 |
| STAU1 | 1148.61 | 364.03 | 761.71 | 321.01 | 9.21 | 0.012 | 0.457 | 1.51 |
| YBX1 | 9608.18 | 2868.84 | 6382.10 | 2824.02 | 34.34 | 0.001 | 0.494 | 1.51 |
| ACADM | 2744.43 | 899.34 | 1823.12 | 1074.29 | 8.42 | 0.014 | 0.452 | 1.51 |
| RPL14L | 7853.30 | 3035.64 | 5218.50 | 2215.68 | 4.32 | 0.050 | 0.452 | 1.50 |
| CAMK2D | 213.43 | 47.59 | 141.90 | 22.43 | 4.76 | 0.041 | 0.444 | 1.50 |
| EFTUD2 | 1612.45 | 331.48 | 1072.07 | 336.81 | 79.11 | 0.000 | 0.944 | 1.50 |
| PRKDC | 214.75 | 11.77 | 142.89 | 9.27 | 5.92 | 0.027 | 0.447 | 1.50 |
| TUBA1A | 21489.05 | 6322.13 | 14308.77 | 5504.78 | 8.67 | 0.013 | 0.451 | 1.50 |
| LOC440737 | 3706.93 | 1124.04 | 2471.23 | 952.85 | 11.41 | 0.008 | 0.452 | 1.50 |
| MEMO1 | 477.75 | 106.75 | 318.57 | 91.18 | 14.52 | 0.005 | 0.443 | 1.50 |
| FGFR1OP | 209.03 | 57.93 | 139.39 | 38.25 | 6.02 | 0.026 | 0.449 | 1.50 |
| NSMCE1 | 478.66 | 88.39 | 319.26 | 61.95 | 10.11 | 0.010 | 0.454 | 1.50 |
| AYP1P1 | 460.77 | 23.63 | 307.54 | 62.11 | 6.63 | 0.022 | 0.453 | 1.50 |
| ZNF280D | 207.88 | 51.16 | 138.75 | 35.14 | 7.35 | 0.018 | 0.450 | 1.50 |
| LOC100133185 | 980.21 | 305.71 | 654.25 | 195.75 | 4.69 | 0.043 | 0.447 | 1.50 |
| LOC645387 | 7312.39 | 2290.11 | 4881.49 | 1771.49 | 4.84 | 0.040 | 0.445 | 1.50 |
| UPF2 | 845.62 | 308.16 | 564.52 | 204.65 | 4.65 | 0.043 | 0.447 | 1.50 |
| NUP188 | 448.71 | 95.99 | 299.62 | 77.95 | 5.76 | 0.029 | 0.445 | 1.50 |
| RAB27A | 321.73 | 65.64 | 214.87 | 32.31 | 4.72 | 0.042 | 0.445 | 1.50 |
| CHD4 | 1229.69 | 204.27 | 821.29 | 269.31 | 10.84 | 0.008 | 0.455 | 1.50 |
| NONO | 5917.12 | 1884.63 | 3954.21 | 1735.95 | 11.02 | 0.008 | 0.457 | 1.50 |
| LDLR | 1172.98 | 448.03 | 784.50 | 360.50 | 7.37 | 0.018 | 0.449 | 1.50 |
| RINL | 481.99 | 152.55 | 322.41 | 112.93 | 6.38 | 0.024 | 0.452 | 1.49 |
| LOC730316 | 1811.15 | 391.12 | 1212.51 | 405.30 | 4.49 | 0.046 | 0.448 | 1.49 |
| C6ORF160 | 4659.20 | 1932.86 | 3121.46 | 1752.86 | 12.30 | 0.007 | 0.440 | 1.49 |
| LOC91561 | 1488.51 | 437.15 | 997.94 | 351.37 | 7.22 | 0.019 | 0.450 | 1.49 |
| RPL10A | 17509.82 | 4205.68 | 11743.36 | 3108.67 | 9.05 | 0.012 | 0.456 | 1.49 |
| EI24 | 498.70 | 166.36 | 334.56 | 121.71 | 6.08 | 0.026 | 0.449 | 1.49 |
| RANBP3 | 681.94 | 170.28 | 457.64 | 127.01 | 7.86 | 0.016 | 0.450 | 1.49 |
| AK2 | 1139.33 | 261.97 | 764.70 | 285.99 | 10.55 | 0.009 | 0.454 | 1.49 |
| RPL13A | 7591.44 | 2087.57 | 5097.82 | 1303.07 | 5.38 | 0.033 | 0.445 | 1.49 |
| TCP1 | 296.43 | 44.08 | 199.06 | 29.72 | 7.32 | 0.018 | 0.450 | 1.49 |
| LOC731365 | 11985.40 | 3884.07 | 8049.70 | 2753.02 | 5.53 | 0.031 | 0.445 | 1.49 |
| DLST | 308.60 | 75.47 | 207.35 | 56.07 | 8.93 | 0.012 | 0.455 | 1.49 |
| EIF4H | 4525.66 | 1219.81 | 3041.08 | 786.73 | 5.84 | 0.028 | 0.446 | 1.49 |
| DLEU1 | 184.78 | 42.34 | 124.25 | 20.51 | 4.80 | 0.041 | 0.444 | 1.49 |
| PRKDC | 409.01 | 118.52 | 275.11 | 64.83 | 4.31 | 0.050 | 0.452 | 1.49 |
| METTL13 | 399.80 | 119.14 | 268.99 | 78.53 | 4.86 | 0.040 | 0.445 | 1.49 |
| MAPKAPK5 | 1334.45 | 434.54 | 897.94 | 381.54 | 12.91 | 0.006 | 0.442 | 1.49 |
| ATP5J2 | 2476.98 | 638.76 | 1666.85 | 655.22 | 22.98 | 0.002 | 0.453 | 1.49 |
| MARS | 1357.57 | 517.70 | 913.57 | 348.08 | 4.38 | 0.048 | 0.451 | 1.49 |
| SLC1A5 | 799.13 | 91.57 | 537.90 | 181.38 | 4.90 | 0.039 | 0.444 | 1.49 |
| MAP4K1 | 482.46 | 84.54 | 324.78 | 62.46 | 6.90 | 0.020 | 0.446 | 1.49 |
| TIGD5 | 484.33 | 99.48 | 326.08 | 97.06 | 15.63 | 0.004 | 0.453 | 1.49 |
| ACOX3 | 408.51 | 56.56 | 275.17 | 79.44 | 4.32 | 0.050 | 0.452 | 1.48 |
| TMEM183B | 2347.88 | 988.88 | 1581.73 | 749.82 | 5.55 | 0.031 | 0.445 | 1.48 |
| IMMP2L | 214.43 | 37.49 | 144.60 | 38.37 | 7.65 | 0.017 | 0.446 | 1.48 |
| HOMER2 | 196.40 | 10.58 | 132.47 | 16.29 | 6.76 | 0.021 | 0.448 | 1.48 |
| C17ORF49 | 1190.60 | 221.92 | 803.04 | 238.66 | 10.98 | 0.008 | 0.455 | 1.48 |
| CWC15 | 1063.62 | 342.98 | 717.91 | 236.99 | 5.10 | 0.036 | 0.447 | 1.48 |
| COASY | 1733.77 | 651.54 | 1170.45 | 497.76 | 5.80 | 0.028 | 0.444 | 1.48 |
| ARHGAP21 | 780.98 | 268.22 | 527.31 | 199.88 | 6.18 | 0.025 | 0.448 | 1.48 |
| HNRNPF | 321.76 | 91.76 | 217.27 | 66.34 | 4.83 | 0.040 | 0.444 | 1.48 |
| UBR7 | 1292.18 | 501.57 | 872.72 | 368.66 | 4.81 | 0.041 | 0.444 | 1.48 |
| CSTF3 | 315.44 | 94.39 | 213.28 | 71.83 | 7.67 | 0.017 | 0.448 | 1.48 |
| HN1 | 290.79 | 76.94 | 196.66 | 62.81 | 11.42 | 0.008 | 0.453 | 1.48 |
| DUS2L | 407.01 | 133.27 | 275.52 | 104.20 | 7.74 | 0.016 | 0.450 | 1.48 |
| SCOC | 733.56 | 262.66 | 496.70 | 220.50 | 6.61 | 0.022 | 0.454 | 1.48 |
| RAD23B | 485.27 | 126.97 | 329.01 | 100.59 | 9.15 | 0.012 | 0.457 | 1.47 |
| ACTG1 | 26659.71 | 6681.65 | 18076.81 | 4215.98 | 5.33 | 0.033 | 0.447 | 1.47 |
| MRRF | 514.47 | 166.69 | 348.91 | 109.58 | 4.66 | 0.043 | 0.448 | 1.47 |
| HSPA8 | 13835.89 | 4039.55 | 9384.18 | 3171.04 | 8.83 | 0.013 | 0.457 | 1.47 |
| LOC401127 | 215.13 | 17.54 | 145.94 | 11.00 | 8.55 | 0.013 | 0.451 | 1.47 |
| LSM1 | 3602.80 | 1049.82 | 2444.95 | 833.13 | 7.19 | 0.019 | 0.450 | 1.47 |
| C5ORF35 | 318.86 | 103.17 | 216.40 | 68.30 | 5.05 | 0.037 | 0.447 | 1.47 |
| DBNL | 3435.75 | 1120.24 | 2333.81 | 760.66 | 5.23 | 0.035 | 0.448 | 1.47 |
| NOP10 | 5689.09 | 1983.72 | 3864.72 | 1932.41 | 6.04 | 0.026 | 0.448 | 1.47 |
| PFN1 | 8462.50 | 272.85 | 5756.92 | 1120.25 | 5.52 | 0.031 | 0.445 | 1.47 |
| SLC2A1 | 746.53 | 278.23 | 508.30 | 231.81 | 8.69 | 0.013 | 0.452 | 1.47 |
| LOC441013 | 10896.71 | 2444.18 | 7421.03 | 2114.25 | 11.19 | 0.008 | 0.456 | 1.47 |
| IQCC | 230.74 | 47.72 | 157.20 | 22.36 | 5.01 | 0.038 | 0.446 | 1.47 |
| GPATCH4 | 193.51 | 38.65 | 131.85 | 16.61 | 4.70 | 0.042 | 0.446 | 1.47 |
| C16ORF61 | 3100.51 | 1153.37 | 2113.11 | 1031.01 | 6.27 | 0.024 | 0.449 | 1.47 |
| COASY | 374.94 | 69.81 | 255.75 | 49.03 | 4.73 | 0.042 | 0.445 | 1.47 |
| PITPNB | 366.79 | 65.24 | 250.20 | 36.29 | 6.37 | 0.024 | 0.453 | 1.47 |
| AP1B1 | 1008.46 | 332.05 | 688.24 | 325.03 | 18.02 | 0.003 | 0.450 | 1.47 |
| PSMB7 | 2777.91 | 1020.93 | 1896.25 | 715.63 | 4.96 | 0.038 | 0.446 | 1.46 |
| LOC100129139 | 207.72 | 6.92 | 141.80 | 5.92 | 8.90 | 0.012 | 0.456 | 1.46 |
| TMSB4X | 417.30 | 13.90 | 284.98 | 18.47 | 16.69 | 0.004 | 0.455 | 1.46 |
| MAD2L2 | 900.85 | 243.08 | 615.32 | 185.98 | 6.95 | 0.020 | 0.447 | 1.46 |
| MRPL49 | 501.30 | 168.72 | 342.51 | 120.27 | 4.59 | 0.044 | 0.448 | 1.46 |
| FANCE | 580.88 | 205.09 | 397.46 | 151.43 | 5.40 | 0.033 | 0.445 | 1.46 |
| U2AF2 | 2655.77 | 864.77 | 1818.15 | 801.87 | 21.39 | 0.002 | 0.452 | 1.46 |
| UBQLN4 | 1005.87 | 305.13 | 688.75 | 288.17 | 14.03 | 0.005 | 0.450 | 1.46 |
| LOC649076 | 22221.37 | 5957.67 | 15222.49 | 4722.29 | 7.20 | 0.019 | 0.450 | 1.46 |
| CBX6 | 2068.46 | 761.75 | 1417.12 | 791.82 | 5.55 | 0.031 | 0.445 | 1.46 |
| TRIAP1 | 632.25 | 238.82 | 433.75 | 171.34 | 4.70 | 0.042 | 0.447 | 1.46 |
| PKN3 | 165.63 | 17.51 | 113.71 | 4.60 | 4.78 | 0.041 | 0.444 | 1.46 |
| DHX36 | 477.82 | 105.84 | 328.15 | 99.14 | 18.22 | 0.003 | 0.455 | 1.46 |
| LOC728553 | 2529.23 | 894.00 | 1737.42 | 605.21 | 4.72 | 0.042 | 0.445 | 1.46 |
| RPL36AL | 3877.84 | 965.65 | 2664.33 | 1073.42 | 17.32 | 0.003 | 0.454 | 1.46 |
| ARMC1 | 675.31 | 258.42 | 464.24 | 179.10 | 4.61 | 0.044 | 0.447 | 1.45 |
| EIF3H | 2490.08 | 841.38 | 1712.24 | 583.84 | 4.68 | 0.043 | 0.447 | 1.45 |
| NME2 | 263.73 | 41.05 | 181.49 | 26.19 | 9.57 | 0.011 | 0.462 | 1.45 |
| SLC27A5 | 179.13 | 18.99 | 123.27 | 10.50 | 6.85 | 0.021 | 0.447 | 1.45 |
| URM1 | 1521.73 | 531.89 | 1048.21 | 371.49 | 5.10 | 0.036 | 0.447 | 1.45 |
| RPL7A | 4653.24 | 1091.99 | 3206.18 | 882.49 | 10.38 | 0.009 | 0.453 | 1.45 |
| CHD4 | 472.04 | 75.45 | 325.48 | 39.13 | 6.93 | 0.020 | 0.447 | 1.45 |
| RPS6P1 | 5867.06 | 1711.64 | 4046.73 | 1627.64 | 11.03 | 0.008 | 0.457 | 1.45 |
| INCENP | 206.35 | 52.27 | 142.34 | 32.63 | 5.56 | 0.031 | 0.444 | 1.45 |
| SFRS4 | 2843.42 | 883.09 | 1962.86 | 674.22 | 4.43 | 0.047 | 0.450 | 1.45 |
| IDH3B | 1246.75 | 332.63 | 860.92 | 234.85 | 5.25 | 0.034 | 0.447 | 1.45 |
| PMS2L4 | 453.38 | 116.56 | 313.34 | 83.89 | 5.85 | 0.028 | 0.446 | 1.45 |
| LOC644816 | 243.78 | 3.82 | 168.58 | 22.03 | 5.06 | 0.037 | 0.446 | 1.45 |
| SEC61G | 4007.42 | 1591.24 | 2772.09 | 1186.93 | 4.96 | 0.038 | 0.446 | 1.45 |
| DDB1 | 3763.22 | 1333.50 | 2604.41 | 1180.80 | 6.18 | 0.025 | 0.449 | 1.44 |
| MED27 | 228.58 | 40.40 | 158.28 | 15.48 | 4.82 | 0.041 | 0.445 | 1.44 |
| DNCL1 | 6490.48 | 1535.22 | 4499.74 | 1669.59 | 20.11 | 0.002 | 0.451 | 1.44 |
| SPAG5 | 188.61 | 25.70 | 130.79 | 15.65 | 6.40 | 0.024 | 0.454 | 1.44 |
| RPL23AP7 | 338.75 | 70.70 | 234.96 | 64.94 | 14.65 | 0.005 | 0.444 | 1.44 |
| ARL2 | 1410.84 | 523.54 | 978.61 | 388.62 | 5.41 | 0.032 | 0.445 | 1.44 |
| SBDSP | 327.90 | 98.93 | 227.47 | 67.63 | 4.65 | 0.043 | 0.447 | 1.44 |
| AATF | 600.59 | 182.61 | 416.65 | 130.26 | 5.90 | 0.028 | 0.447 | 1.44 |
| GCN1L1 | 1823.31 | 695.38 | 1264.97 | 566.78 | 5.82 | 0.028 | 0.445 | 1.44 |
| SGTA | 281.33 | 37.14 | 195.33 | 9.36 | 5.17 | 0.035 | 0.447 | 1.44 |
| MCAT | 169.68 | 16.21 | 117.91 | 7.75 | 7.95 | 0.015 | 0.453 | 1.44 |
| PLSCR3 | 1659.53 | 612.69 | 1154.13 | 498.46 | 7.24 | 0.019 | 0.450 | 1.44 |
| HSD17B8 | 261.09 | 9.77 | 181.65 | 11.47 | 21.21 | 0.002 | 0.446 | 1.44 |
| ABCB6 | 377.15 | 60.18 | 262.81 | 42.47 | 10.75 | 0.009 | 0.453 | 1.44 |
| C19ORF53 | 1430.85 | 244.28 | 997.86 | 357.33 | 5.55 | 0.031 | 0.445 | 1.43 |
| C6ORF108 | 1069.51 | 290.90 | 745.96 | 272.33 | 4.99 | 0.038 | 0.447 | 1.43 |
| PTGIR | 154.41 | 14.10 | 107.74 | 2.71 | 5.71 | 0.029 | 0.446 | 1.43 |
| KDELR2 | 1096.03 | 313.83 | 764.77 | 282.79 | 14.09 | 0.005 | 0.450 | 1.43 |
| LOC127295 | 2125.42 | 639.04 | 1483.07 | 454.82 | 6.04 | 0.026 | 0.449 | 1.43 |
| TRIM37 | 237.05 | 61.64 | 165.46 | 34.57 | 4.58 | 0.045 | 0.448 | 1.43 |
| COPS8 | 270.76 | 58.25 | 189.02 | 30.76 | 4.97 | 0.038 | 0.446 | 1.43 |
| C17ORF42 | 380.44 | 60.57 | 265.61 | 59.49 | 14.06 | 0.005 | 0.451 | 1.43 |
| NME2 | 216.74 | 21.55 | 151.39 | 11.63 | 4.44 | 0.047 | 0.450 | 1.43 |
| ABCC4 | 248.65 | 75.41 | 173.71 | 56.32 | 5.10 | 0.036 | 0.446 | 1.43 |
| SRPR | 453.79 | 48.41 | 317.50 | 17.57 | 7.26 | 0.018 | 0.450 | 1.43 |
| RBM42 | 1060.15 | 138.82 | 742.17 | 125.78 | 6.96 | 0.020 | 0.448 | 1.43 |
| WDR62 | 144.31 | 4.24 | 101.09 | 11.00 | 4.91 | 0.039 | 0.445 | 1.43 |
| SF3A2 | 1397.87 | 270.52 | 980.28 | 161.55 | 6.47 | 0.023 | 0.453 | 1.43 |
| NIF3L1 | 1497.21 | 537.96 | 1050.29 | 404.27 | 4.65 | 0.043 | 0.447 | 1.43 |
| RBBP9 | 265.63 | 78.42 | 186.37 | 60.91 | 7.02 | 0.020 | 0.449 | 1.43 |
| LOC645058 | 2214.27 | 756.08 | 1554.13 | 580.67 | 5.20 | 0.035 | 0.447 | 1.42 |
| RGS10 | 2789.77 | 926.41 | 1959.39 | 856.55 | 8.30 | 0.014 | 0.452 | 1.42 |
| RNF126 | 451.43 | 147.40 | 317.06 | 127.72 | 9.87 | 0.010 | 0.458 | 1.42 |
| SFPQ | 371.35 | 92.01 | 260.91 | 86.39 | 6.93 | 0.020 | 0.447 | 1.42 |
| CCBL2 | 788.56 | 270.98 | 554.12 | 210.83 | 5.71 | 0.029 | 0.446 | 1.42 |
| ATP5G3 | 217.95 | 42.57 | 153.17 | 29.34 | 7.64 | 0.017 | 0.446 | 1.42 |
| GTF3C5 | 473.35 | 35.58 | 333.09 | 72.71 | 6.54 | 0.023 | 0.454 | 1.42 |
| LOC648771 | 7027.56 | 1461.94 | 4945.64 | 1760.71 | 11.07 | 0.008 | 0.458 | 1.42 |
| DDX41 | 495.71 | 106.56 | 348.98 | 72.51 | 6.44 | 0.023 | 0.453 | 1.42 |
| CRKRS | 519.05 | 159.64 | 365.52 | 147.71 | 15.85 | 0.004 | 0.453 | 1.42 |
| RPL8 | 161.03 | 25.73 | 113.40 | 12.67 | 5.96 | 0.027 | 0.448 | 1.42 |
| MTA2 | 509.32 | 80.73 | 358.79 | 72.45 | 26.30 | 0.001 | 0.449 | 1.42 |
| PLD6 | 204.66 | 34.06 | 144.19 | 21.80 | 5.35 | 0.033 | 0.446 | 1.42 |
| C19ORF24 | 328.19 | 39.85 | 231.34 | 11.33 | 4.96 | 0.038 | 0.446 | 1.42 |
| SLC35B1 | 1279.27 | 384.21 | 902.56 | 283.82 | 5.94 | 0.027 | 0.447 | 1.42 |
| PABPN1 | 1706.10 | 518.77 | 1204.57 | 559.65 | 7.14 | 0.019 | 0.450 | 1.42 |
| DGUOK | 1993.46 | 659.73 | 1407.69 | 482.39 | 4.92 | 0.039 | 0.445 | 1.42 |
| CINP | 176.12 | 18.04 | 124.38 | 9.66 | 10.64 | 0.009 | 0.453 | 1.42 |
| LOC390578 | 250.01 | 41.82 | 176.59 | 16.43 | 4.99 | 0.038 | 0.447 | 1.42 |
| TMEM126B | 2827.13 | 967.52 | 1996.93 | 854.18 | 11.00 | 0.008 | 0.456 | 1.42 |
| DPP3 | 378.15 | 101.97 | 267.14 | 67.69 | 5.31 | 0.034 | 0.447 | 1.42 |
| HAUS4 | 569.69 | 144.41 | 402.67 | 146.40 | 4.46 | 0.047 | 0.450 | 1.41 |
| CHCHD5 | 308.41 | 31.44 | 218.03 | 45.79 | 10.69 | 0.009 | 0.453 | 1.41 |
| CLTA | 1677.86 | 450.77 | 1187.12 | 471.97 | 38.06 | 0.001 | 0.509 | 1.41 |
| LOC389599 | 394.42 | 94.69 | 279.27 | 72.49 | 8.46 | 0.014 | 0.452 | 1.41 |
| RAVER1 | 411.08 | 16.65 | 291.13 | 4.92 | 13.46 | 0.005 | 0.443 | 1.41 |
| ZNF581 | 918.22 | 103.95 | 650.65 | 104.65 | 7.55 | 0.017 | 0.446 | 1.41 |
| VPS33A | 414.46 | 98.11 | 293.97 | 93.58 | 6.77 | 0.021 | 0.448 | 1.41 |
| C1ORF86 | 291.43 | 61.33 | 206.77 | 32.70 | 4.63 | 0.044 | 0.447 | 1.41 |
| LOC728729 | 177.97 | 11.17 | 126.28 | 5.42 | 15.46 | 0.004 | 0.452 | 1.41 |
| FAM89A | 449.16 | 78.39 | 318.87 | 90.63 | 8.09 | 0.015 | 0.452 | 1.41 |
| PHF6 | 188.13 | 15.99 | 133.58 | 18.58 | 7.17 | 0.019 | 0.451 | 1.41 |
| PTP4A3 | 155.51 | 7.88 | 110.43 | 10.29 | 4.74 | 0.042 | 0.445 | 1.41 |
| NDUFA8 | 1850.35 | 667.12 | 1314.07 | 535.94 | 6.82 | 0.021 | 0.448 | 1.41 |
| DYNC1I2 | 943.42 | 256.81 | 670.25 | 235.39 | 6.51 | 0.023 | 0.452 | 1.41 |
| NGDN | 1127.74 | 357.73 | 801.95 | 299.34 | 9.66 | 0.011 | 0.462 | 1.41 |
| FKBP3 | 920.96 | 384.58 | 655.28 | 313.24 | 5.72 | 0.029 | 0.446 | 1.41 |
| LOC100129650 | 1346.44 | 342.82 | 958.64 | 357.49 | 5.18 | 0.035 | 0.447 | 1.40 |
| TRMT112 | 2507.94 | 609.17 | 1786.58 | 635.72 | 6.10 | 0.026 | 0.450 | 1.40 |
| STAT3 | 571.15 | 84.47 | 407.03 | 40.79 | 6.01 | 0.027 | 0.448 | 1.40 |
| ZNF165 | 224.67 | 35.80 | 160.20 | 36.95 | 7.44 | 0.018 | 0.448 | 1.40 |
| CHERP | 277.29 | 50.60 | 197.72 | 29.33 | 5.49 | 0.032 | 0.445 | 1.40 |
| DHDDS | 364.88 | 65.43 | 260.26 | 27.61 | 4.79 | 0.041 | 0.444 | 1.40 |
| TYSND1 | 684.90 | 197.33 | 488.72 | 159.18 | 5.86 | 0.028 | 0.445 | 1.40 |
| LOC644464 | 16835.96 | 5663.08 | 12017.57 | 4140.75 | 5.35 | 0.033 | 0.446 | 1.40 |
| MIR599 | 186.60 | 30.94 | 133.38 | 12.34 | 4.54 | 0.045 | 0.449 | 1.40 |
| PDE12 | 388.34 | 102.72 | 277.65 | 60.26 | 4.49 | 0.046 | 0.448 | 1.40 |
| LOC285900 | 6618.69 | 2095.03 | 4732.16 | 2033.16 | 13.75 | 0.005 | 0.446 | 1.40 |
| SUMO3 | 3175.43 | 1199.20 | 2272.07 | 853.08 | 4.52 | 0.046 | 0.449 | 1.40 |
| RAG1AP1 | 708.75 | 201.48 | 507.22 | 213.97 | 27.66 | 0.001 | 0.453 | 1.40 |
| GPS1 | 1389.48 | 273.95 | 995.04 | 344.40 | 4.79 | 0.041 | 0.444 | 1.40 |
| SUCLG1 | 1325.34 | 621.10 | 949.56 | 501.47 | 4.44 | 0.047 | 0.450 | 1.40 |
| DUS1L | 315.83 | 45.12 | 226.28 | 45.67 | 50.06 | 0.000 | 0.589 | 1.40 |
| RGS10 | 618.47 | 150.45 | 443.12 | 123.85 | 4.93 | 0.039 | 0.445 | 1.40 |
| LOC284167 | 425.26 | 90.31 | 304.75 | 51.49 | 5.29 | 0.034 | 0.447 | 1.40 |
| U2AF1L4 | 314.75 | 78.55 | 225.79 | 53.63 | 6.18 | 0.025 | 0.448 | 1.39 |
| PARP3 | 640.07 | 127.53 | 459.27 | 175.38 | 5.01 | 0.038 | 0.447 | 1.39 |
| SLC38A5 | 198.34 | 5.96 | 142.35 | 3.18 | 18.28 | 0.003 | 0.453 | 1.39 |
| LOC148430 | 19797.38 | 4862.59 | 14215.85 | 4530.43 | 4.67 | 0.043 | 0.447 | 1.39 |
| CRELD2 | 1221.50 | 359.79 | 877.69 | 314.98 | 12.42 | 0.006 | 0.444 | 1.39 |
| AP1M1 | 903.08 | 220.42 | 648.96 | 189.80 | 14.35 | 0.005 | 0.445 | 1.39 |
| SLCO4A1 | 299.43 | 77.65 | 215.21 | 66.25 | 4.65 | 0.043 | 0.447 | 1.39 |
| C11ORF57 | 367.92 | 52.81 | 264.55 | 50.31 | 6.27 | 0.025 | 0.449 | 1.39 |
| SNW1 | 576.15 | 141.79 | 415.13 | 152.15 | 22.63 | 0.002 | 0.451 | 1.39 |
| PTBP1 | 9686.52 | 3925.27 | 6982.89 | 3014.50 | 4.98 | 0.038 | 0.447 | 1.39 |
| PEMT | 380.74 | 65.93 | 274.50 | 60.94 | 7.25 | 0.019 | 0.450 | 1.39 |
| ATP5J2 | 2511.51 | 504.42 | 1810.87 | 674.61 | 5.49 | 0.032 | 0.446 | 1.39 |
| NEIL2 | 260.15 | 65.57 | 187.59 | 53.52 | 7.59 | 0.017 | 0.444 | 1.39 |
| TUBG1 | 241.87 | 21.48 | 174.42 | 7.62 | 6.74 | 0.021 | 0.448 | 1.39 |
| FAM86A | 189.40 | 7.46 | 136.60 | 7.06 | 18.67 | 0.003 | 0.453 | 1.39 |
| LOC729760 | 278.27 | 42.53 | 200.70 | 25.73 | 6.65 | 0.022 | 0.452 | 1.39 |
| LOC729366 | 205.30 | 28.27 | 148.14 | 7.18 | 4.35 | 0.049 | 0.452 | 1.39 |
| RIF1 | 200.91 | 16.19 | 144.98 | 5.33 | 8.83 | 0.013 | 0.456 | 1.39 |
| BUD31 | 1256.13 | 334.06 | 906.46 | 301.34 | 14.70 | 0.005 | 0.448 | 1.39 |
| RASAL1 | 206.12 | 54.97 | 148.80 | 35.22 | 4.95 | 0.038 | 0.446 | 1.39 |
| DPH3 | 588.77 | 181.68 | 425.06 | 191.04 | 4.49 | 0.046 | 0.448 | 1.39 |
| SLC19A1 | 189.89 | 12.86 | 137.14 | 4.48 | 8.79 | 0.013 | 0.457 | 1.38 |
| PCGF5 | 283.13 | 34.51 | 204.52 | 42.75 | 7.15 | 0.019 | 0.450 | 1.38 |
| TCTEX1D2 | 230.63 | 30.55 | 166.67 | 14.51 | 6.21 | 0.025 | 0.450 | 1.38 |
| TRAPPC2L | 1279.60 | 424.27 | 924.79 | 295.98 | 4.57 | 0.045 | 0.448 | 1.38 |
| DDA1 | 1691.38 | 665.18 | 1222.57 | 613.38 | 11.16 | 0.008 | 0.455 | 1.38 |
| PRR3 | 228.19 | 45.69 | 164.95 | 32.59 | 7.52 | 0.017 | 0.447 | 1.38 |
| TARBP1 | 263.77 | 75.39 | 190.75 | 56.92 | 5.13 | 0.036 | 0.446 | 1.38 |
| AGFG2 | 218.93 | 7.67 | 158.34 | 2.69 | 11.48 | 0.007 | 0.451 | 1.38 |
| SUPT5H | 826.51 | 151.58 | 598.16 | 85.28 | 4.39 | 0.048 | 0.451 | 1.38 |
| LOC100133055 | 194.66 | 45.39 | 141.01 | 26.44 | 4.79 | 0.041 | 0.444 | 1.38 |
| LOC647597 | 197.81 | 20.97 | 143.30 | 3.90 | 5.35 | 0.033 | 0.447 | 1.38 |
| HNRNPD | 11023.53 | 4269.09 | 7988.62 | 3283.95 | 5.24 | 0.035 | 0.447 | 1.38 |
| BAZ1A | 382.41 | 88.36 | 277.21 | 85.19 | 15.06 | 0.004 | 0.450 | 1.38 |
| LOC730323 | 279.52 | 64.54 | 202.69 | 41.58 | 4.88 | 0.040 | 0.444 | 1.38 |
| LOC400963 | 22378.56 | 4594.15 | 16234.53 | 4980.14 | 5.47 | 0.032 | 0.445 | 1.38 |
| RPS9 | 5747.35 | 907.26 | 4170.89 | 1088.17 | 4.62 | 0.044 | 0.447 | 1.38 |
| TGIF2 | 453.84 | 157.60 | 329.46 | 129.49 | 7.66 | 0.017 | 0.447 | 1.38 |
| L3MBTL2 | 439.46 | 119.89 | 319.02 | 85.81 | 5.18 | 0.035 | 0.447 | 1.38 |
| CNOT7 | 1138.03 | 383.16 | 826.25 | 338.32 | 9.48 | 0.011 | 0.464 | 1.38 |
| LOC100131859 | 401.66 | 188.50 | 291.63 | 176.62 | 4.95 | 0.038 | 0.445 | 1.38 |
| HSPD1 | 186.79 | 22.70 | 135.74 | 11.34 | 6.50 | 0.023 | 0.452 | 1.38 |
| GTPBP3 | 226.92 | 30.79 | 164.90 | 31.84 | 72.90 | 0.000 | 0.808 | 1.38 |
| LOC653375 | 263.71 | 42.56 | 191.69 | 46.71 | 14.10 | 0.005 | 0.451 | 1.38 |
| NOP2 | 395.78 | 80.43 | 287.74 | 104.64 | 5.25 | 0.034 | 0.447 | 1.38 |
| LY9 | 387.83 | 106.88 | 281.98 | 84.77 | 4.78 | 0.041 | 0.444 | 1.38 |
| WDR79 | 230.81 | 32.54 | 167.94 | 39.92 | 7.59 | 0.017 | 0.445 | 1.37 |
| RCC2 | 2675.08 | 953.28 | 1947.00 | 830.56 | 5.04 | 0.037 | 0.447 | 1.37 |
| PSMC5 | 3840.16 | 1255.16 | 2795.25 | 1383.59 | 12.34 | 0.007 | 0.441 | 1.37 |
| SNAP47 | 311.88 | 80.54 | 227.17 | 55.74 | 5.15 | 0.036 | 0.446 | 1.37 |
| GIYD1 | 342.75 | 46.72 | 249.86 | 39.80 | 10.72 | 0.009 | 0.453 | 1.37 |
| TMEM41A | 176.34 | 27.77 | 128.59 | 17.97 | 5.48 | 0.032 | 0.445 | 1.37 |
| SLC25A10 | 213.68 | 27.00 | 155.90 | 7.08 | 4.97 | 0.038 | 0.446 | 1.37 |
| C11ORF60 | 199.95 | 42.29 | 145.89 | 29.96 | 7.33 | 0.018 | 0.450 | 1.37 |
| ARL17B | 194.28 | 8.13 | 141.81 | 8.38 | 29.89 | 0.001 | 0.451 | 1.37 |
| HIF1A | 480.16 | 138.61 | 350.54 | 95.21 | 4.86 | 0.040 | 0.445 | 1.37 |
| NFKB2 | 327.25 | 43.82 | 239.02 | 11.97 | 4.71 | 0.042 | 0.446 | 1.37 |
| MRS2 | 270.38 | 61.60 | 197.48 | 54.70 | 16.46 | 0.004 | 0.457 | 1.37 |
| LOC645693 | 428.33 | 57.33 | 313.13 | 82.12 | 6.31 | 0.024 | 0.451 | 1.37 |
| RPS5 | 7914.13 | 2282.66 | 5789.84 | 2186.47 | 15.23 | 0.004 | 0.447 | 1.37 |
| LOC441896 | 327.57 | 79.93 | 239.81 | 45.99 | 4.33 | 0.049 | 0.452 | 1.37 |
| TXN2 | 186.05 | 9.23 | 136.31 | 26.54 | 4.40 | 0.048 | 0.450 | 1.36 |
| GSTP1 | 3751.01 | 1131.05 | 2748.85 | 808.16 | 4.93 | 0.039 | 0.445 | 1.36 |
| NUP62 | 463.18 | 105.72 | 339.61 | 90.64 | 4.50 | 0.046 | 0.448 | 1.36 |
| FAM96A | 4183.34 | 1672.06 | 3070.52 | 1239.88 | 4.31 | 0.050 | 0.452 | 1.36 |
| TMEM141 | 506.36 | 41.92 | 371.77 | 76.28 | 5.57 | 0.031 | 0.445 | 1.36 |
| AEN | 302.91 | 56.53 | 222.45 | 41.84 | 4.50 | 0.046 | 0.449 | 1.36 |
| ABCB7 | 568.40 | 216.11 | 417.42 | 210.83 | 10.25 | 0.009 | 0.452 | 1.36 |
| NDUFS5 | 2630.56 | 932.31 | 1931.87 | 851.65 | 9.83 | 0.010 | 0.457 | 1.36 |
| TOP1 | 284.85 | 55.41 | 209.27 | 34.77 | 6.25 | 0.025 | 0.449 | 1.36 |
| PPP1R8 | 296.12 | 61.51 | 217.90 | 46.67 | 8.28 | 0.014 | 0.452 | 1.36 |
| STK4 | 5000.53 | 1315.70 | 3679.89 | 1098.98 | 10.44 | 0.009 | 0.455 | 1.36 |
| POLR2J | 229.41 | 18.36 | 168.90 | 13.68 | 20.92 | 0.002 | 0.443 | 1.36 |
| LOC651745 | 183.40 | 16.18 | 135.14 | 7.27 | 7.66 | 0.017 | 0.447 | 1.36 |
| MDH1 | 3596.07 | 1242.43 | 2651.21 | 1220.93 | 7.42 | 0.018 | 0.449 | 1.36 |
| PIH1D1 | 372.55 | 90.33 | 274.76 | 65.48 | 4.46 | 0.047 | 0.449 | 1.36 |
| BEND4 | 187.77 | 18.37 | 138.53 | 14.26 | 4.77 | 0.041 | 0.444 | 1.36 |
| MRPS34 | 211.10 | 3.95 | 155.80 | 5.34 | 22.68 | 0.002 | 0.456 | 1.35 |
| CCDC85B | 240.02 | 20.87 | 177.17 | 10.22 | 4.91 | 0.039 | 0.445 | 1.35 |
| CSNK2A1 | 282.63 | 28.79 | 208.80 | 14.62 | 7.22 | 0.019 | 0.450 | 1.35 |
| DPH3 | 597.13 | 178.54 | 441.43 | 209.91 | 6.93 | 0.020 | 0.447 | 1.35 |
| VCP | 684.00 | 210.76 | 506.22 | 205.02 | 12.22 | 0.007 | 0.440 | 1.35 |
| ALDH4A1 | 162.01 | 28.60 | 119.92 | 16.71 | 6.01 | 0.027 | 0.448 | 1.35 |
| HEBP2 | 973.90 | 323.15 | 720.89 | 289.02 | 10.77 | 0.009 | 0.454 | 1.35 |
| TPM3 | 1121.62 | 368.84 | 830.38 | 431.98 | 4.84 | 0.040 | 0.444 | 1.35 |
| ESD | 307.39 | 91.02 | 227.76 | 89.54 | 53.46 | 0.000 | 0.636 | 1.35 |
| GLE1 | 1637.98 | 487.92 | 1213.93 | 435.89 | 9.20 | 0.012 | 0.456 | 1.35 |
| BAZ1A | 2046.08 | 879.29 | 1516.95 | 726.62 | 4.52 | 0.046 | 0.448 | 1.35 |
| LOC645018 | 929.57 | 155.01 | 689.42 | 210.09 | 4.98 | 0.038 | 0.447 | 1.35 |
| NUP210 | 683.64 | 160.01 | 507.11 | 207.94 | 5.14 | 0.036 | 0.446 | 1.35 |
| NDUFA11 | 700.30 | 135.18 | 519.56 | 97.66 | 7.08 | 0.019 | 0.450 | 1.35 |
| MAPRE2 | 949.65 | 186.03 | 704.57 | 233.36 | 4.72 | 0.042 | 0.445 | 1.35 |
| MRPL55 | 557.15 | 197.24 | 413.52 | 151.27 | 4.68 | 0.043 | 0.447 | 1.35 |
| LOC643779 | 456.78 | 27.77 | 339.10 | 25.79 | 31.39 | 0.001 | 0.479 | 1.35 |
| SDF2L1 | 2115.83 | 600.70 | 1571.16 | 453.42 | 5.90 | 0.028 | 0.447 | 1.35 |
| NFKBIB | 316.02 | 45.32 | 234.67 | 65.21 | 6.30 | 0.024 | 0.450 | 1.35 |
| TIMM44 | 245.42 | 57.39 | 182.27 | 35.62 | 4.41 | 0.048 | 0.450 | 1.35 |
| SCRIB | 611.77 | 244.65 | 454.69 | 209.31 | 4.64 | 0.043 | 0.447 | 1.35 |
| CD2BP2 | 1757.48 | 473.97 | 1306.32 | 509.13 | 7.25 | 0.018 | 0.450 | 1.35 |
| BIRC5 | 136.84 | 12.16 | 101.72 | 6.82 | 9.30 | 0.011 | 0.460 | 1.35 |
| PRKRIR | 2094.91 | 572.44 | 1557.41 | 635.30 | 14.74 | 0.005 | 0.451 | 1.35 |
| ZNF364 | 933.02 | 77.73 | 693.80 | 78.16 | 31.27 | 0.001 | 0.473 | 1.34 |
| LOC402057 | 282.98 | 41.39 | 210.73 | 18.06 | 4.37 | 0.049 | 0.451 | 1.34 |
| AK3L1 | 346.11 | 116.66 | 257.92 | 118.20 | 34.10 | 0.001 | 0.489 | 1.34 |
| LOC730324 | 567.93 | 185.28 | 423.30 | 160.70 | 9.45 | 0.011 | 0.465 | 1.34 |
| TMED2 | 1970.49 | 888.61 | 1469.15 | 712.53 | 4.42 | 0.048 | 0.450 | 1.34 |
| ACBD6 | 223.02 | 29.07 | 166.40 | 20.67 | 10.53 | 0.009 | 0.455 | 1.34 |
| TBCE | 239.17 | 56.59 | 178.59 | 37.50 | 4.74 | 0.042 | 0.445 | 1.34 |
| RNF126 | 188.64 | 22.17 | 140.93 | 15.67 | 6.22 | 0.025 | 0.449 | 1.34 |
| ARMCX6 | 291.64 | 62.65 | 217.95 | 50.54 | 4.72 | 0.042 | 0.445 | 1.34 |
| GOSR2 | 316.00 | 63.35 | 236.71 | 60.75 | 8.74 | 0.013 | 0.455 | 1.33 |
| MTX1 | 435.00 | 92.84 | 325.88 | 111.81 | 5.29 | 0.034 | 0.447 | 1.33 |
| MTP18 | 183.00 | 20.67 | 137.10 | 10.35 | 4.61 | 0.044 | 0.447 | 1.33 |
| LOC644363 | 1456.33 | 475.81 | 1091.38 | 459.86 | 11.05 | 0.008 | 0.457 | 1.33 |
| GGH | 259.83 | 88.05 | 194.77 | 82.85 | 9.07 | 0.012 | 0.456 | 1.33 |
| MAPKAP1 | 217.72 | 15.17 | 163.20 | 9.18 | 6.24 | 0.025 | 0.449 | 1.33 |
| TRPM4 | 162.13 | 21.57 | 121.54 | 14.16 | 4.93 | 0.039 | 0.445 | 1.33 |
| SCYL1 | 499.40 | 118.42 | 374.47 | 127.49 | 4.52 | 0.046 | 0.448 | 1.33 |
| C17ORF70 | 1243.73 | 446.78 | 932.62 | 362.42 | 6.15 | 0.025 | 0.448 | 1.33 |
| C18ORF45 | 183.70 | 37.08 | 137.81 | 29.52 | 6.70 | 0.022 | 0.450 | 1.33 |
| VPS29 | 2368.68 | 809.63 | 1777.00 | 748.43 | 10.97 | 0.008 | 0.454 | 1.33 |
| CKLF | 1322.18 | 362.79 | 991.92 | 311.73 | 10.47 | 0.009 | 0.455 | 1.33 |
| RINT1 | 314.73 | 58.20 | 236.41 | 44.02 | 6.30 | 0.024 | 0.450 | 1.33 |
| ISOC2 | 424.78 | 117.08 | 319.11 | 95.22 | 8.15 | 0.015 | 0.451 | 1.33 |
| DDX11 | 229.93 | 19.19 | 172.76 | 21.61 | 7.14 | 0.019 | 0.450 | 1.33 |
| CKB | 147.78 | 1.11 | 111.04 | 10.30 | 5.60 | 0.030 | 0.445 | 1.33 |
| MAD2L1BP | 401.48 | 133.18 | 301.70 | 93.51 | 4.36 | 0.049 | 0.452 | 1.33 |
| LOC100127918 | 451.10 | 61.18 | 339.05 | 102.67 | 4.58 | 0.044 | 0.448 | 1.33 |
| FAM86A | 257.17 | 46.81 | 193.33 | 27.47 | 5.31 | 0.034 | 0.447 | 1.33 |
| RPL5 | 7653.24 | 1628.78 | 5756.78 | 2023.04 | 7.91 | 0.016 | 0.451 | 1.33 |
| MAPKAP1 | 318.30 | 51.38 | 239.45 | 43.06 | 10.49 | 0.009 | 0.456 | 1.33 |
| C8ORF30B | 192.91 | 11.02 | 145.13 | 19.72 | 8.25 | 0.014 | 0.452 | 1.33 |
| STAMBP | 1125.70 | 396.54 | 847.60 | 308.95 | 4.91 | 0.039 | 0.445 | 1.33 |
| EME1 | 191.54 | 20.58 | 144.27 | 13.53 | 9.58 | 0.011 | 0.463 | 1.33 |
| HDGF | 493.62 | 85.80 | 371.80 | 48.29 | 4.34 | 0.049 | 0.452 | 1.33 |
| ST7 | 205.94 | 31.99 | 155.12 | 17.38 | 5.62 | 0.030 | 0.445 | 1.33 |
| LOC286157 | 1414.52 | 314.74 | 1066.15 | 257.21 | 7.86 | 0.016 | 0.451 | 1.33 |
| RPL14L | 4413.64 | 929.77 | 3326.92 | 1115.73 | 6.63 | 0.022 | 0.453 | 1.33 |
| TMEM201 | 147.13 | 23.46 | 110.96 | 12.15 | 5.37 | 0.033 | 0.446 | 1.33 |
| MVD | 244.18 | 24.47 | 184.24 | 20.52 | 10.29 | 0.009 | 0.453 | 1.33 |
| FAM86C | 161.06 | 12.59 | 121.53 | 2.15 | 6.39 | 0.024 | 0.453 | 1.33 |
| NUP43 | 1159.33 | 356.07 | 874.82 | 377.97 | 13.25 | 0.006 | 0.445 | 1.33 |
| PPP1R8 | 245.54 | 50.41 | 185.89 | 42.43 | 12.91 | 0.006 | 0.440 | 1.32 |
| LOC723972 | 281.88 | 35.16 | 213.42 | 41.50 | 9.53 | 0.011 | 0.463 | 1.32 |
| ARHGAP11A | 141.78 | 8.61 | 107.40 | 2.12 | 8.68 | 0.013 | 0.452 | 1.32 |
| MED7 | 368.54 | 105.55 | 279.21 | 76.03 | 5.23 | 0.035 | 0.447 | 1.32 |
| NDUFB8 | 5140.28 | 1554.06 | 3895.95 | 1325.02 | 4.45 | 0.047 | 0.450 | 1.32 |
| FAM64A | 149.88 | 18.91 | 113.62 | 15.32 | 11.27 | 0.008 | 0.455 | 1.32 |
| SNRNP27 | 955.62 | 388.46 | 724.60 | 318.55 | 5.15 | 0.036 | 0.447 | 1.32 |
| INVS | 268.78 | 59.02 | 203.94 | 42.64 | 6.86 | 0.021 | 0.447 | 1.32 |
| FAM96B | 1176.07 | 289.96 | 892.58 | 313.33 | 5.15 | 0.036 | 0.447 | 1.32 |
| ATP5O | 3568.07 | 1079.32 | 2709.34 | 889.93 | 4.61 | 0.044 | 0.447 | 1.32 |
| SNUPN | 400.73 | 35.79 | 304.37 | 54.96 | 5.48 | 0.032 | 0.445 | 1.32 |
| NAT5 | 3898.44 | 1258.21 | 2962.45 | 1024.63 | 6.84 | 0.021 | 0.448 | 1.32 |
| C12ORF29 | 326.82 | 90.28 | 248.43 | 66.40 | 5.63 | 0.030 | 0.446 | 1.32 |
| FAM119A | 252.59 | 23.90 | 192.18 | 17.13 | 5.48 | 0.032 | 0.445 | 1.31 |
| DDX23 | 712.18 | 208.11 | 541.87 | 166.75 | 4.42 | 0.048 | 0.450 | 1.31 |
| ARHGEF7 | 176.35 | 27.98 | 134.26 | 20.62 | 6.37 | 0.024 | 0.453 | 1.31 |
| CAMK2D | 151.56 | 15.77 | 115.48 | 6.14 | 5.95 | 0.027 | 0.447 | 1.31 |
| LOC644191 | 4244.28 | 1621.44 | 3235.52 | 1495.29 | 13.72 | 0.005 | 0.446 | 1.31 |
| LOC203547 | 909.21 | 318.54 | 693.55 | 235.36 | 4.46 | 0.047 | 0.449 | 1.31 |
| RPS14 | 8129.16 | 1415.19 | 6209.97 | 1640.57 | 11.07 | 0.008 | 0.458 | 1.31 |
| UBE2D3 | 2990.05 | 1011.55 | 2284.93 | 863.24 | 5.04 | 0.037 | 0.447 | 1.31 |
| ITGB1BP1 | 1306.69 | 595.07 | 999.31 | 515.31 | 4.93 | 0.039 | 0.446 | 1.31 |
| PCMT1 | 2594.16 | 842.32 | 1985.58 | 773.14 | 5.66 | 0.030 | 0.446 | 1.31 |
| DUSP4 | 124.35 | 8.63 | 95.21 | 2.18 | 5.02 | 0.037 | 0.447 | 1.31 |
| PSMG3 | 199.46 | 41.59 | 152.78 | 28.28 | 5.05 | 0.037 | 0.447 | 1.31 |
| LOC100130892 | 2350.29 | 960.93 | 1803.04 | 1003.76 | 6.21 | 0.025 | 0.449 | 1.30 |
| VPS72 | 271.22 | 47.01 | 208.09 | 33.26 | 7.94 | 0.015 | 0.452 | 1.30 |
| LOC647276 | 14174.02 | 3305.87 | 10877.36 | 3223.59 | 4.52 | 0.046 | 0.449 | 1.30 |
| C16ORF42 | 363.14 | 61.18 | 278.81 | 64.68 | 5.08 | 0.037 | 0.447 | 1.30 |
| EI24 | 312.88 | 60.32 | 240.26 | 69.38 | 8.19 | 0.015 | 0.453 | 1.30 |
| SNORA64 | 183.09 | 13.20 | 140.66 | 4.70 | 4.82 | 0.040 | 0.445 | 1.30 |
| LOC645381 | 200.53 | 39.82 | 154.10 | 33.68 | 9.86 | 0.010 | 0.458 | 1.30 |
| NCDN | 190.58 | 12.42 | 146.55 | 4.07 | 9.12 | 0.012 | 0.456 | 1.30 |
| HSPCAL3 | 314.60 | 30.62 | 242.09 | 44.58 | 6.61 | 0.022 | 0.453 | 1.30 |
| XPR1 | 725.94 | 265.39 | 558.65 | 229.65 | 6.91 | 0.020 | 0.447 | 1.30 |
| LOC401019 | 16187.68 | 2571.30 | 12462.70 | 2705.04 | 9.61 | 0.011 | 0.463 | 1.30 |
| EIF4G2 | 6999.33 | 2456.33 | 5389.02 | 2080.90 | 7.28 | 0.018 | 0.450 | 1.30 |
| UBE2NL | 172.04 | 18.43 | 132.55 | 3.43 | 4.47 | 0.047 | 0.449 | 1.30 |
| C7ORF20 | 192.79 | 21.80 | 148.55 | 15.71 | 11.35 | 0.008 | 0.451 | 1.30 |
| TAF1D | 301.76 | 104.24 | 232.69 | 87.89 | 5.18 | 0.035 | 0.447 | 1.30 |
| CISH | 354.69 | 62.12 | 273.53 | 61.39 | 6.19 | 0.025 | 0.449 | 1.30 |
| C12ORF43 | 291.95 | 75.11 | 225.20 | 61.22 | 8.18 | 0.015 | 0.452 | 1.30 |
| LOC728689 | 264.47 | 59.71 | 204.02 | 58.41 | 6.22 | 0.025 | 0.449 | 1.30 |
| AZI1 | 340.38 | 103.51 | 262.58 | 82.62 | 6.41 | 0.023 | 0.453 | 1.30 |
| MEF2C | 417.93 | 97.19 | 322.41 | 89.21 | 6.03 | 0.026 | 0.449 | 1.30 |
| LOC441034 | 26794.90 | 6827.81 | 20694.96 | 6043.29 | 4.82 | 0.041 | 0.445 | 1.29 |
| GNL1 | 469.56 | 49.71 | 362.93 | 69.58 | 5.37 | 0.033 | 0.445 | 1.29 |
| NDUFB2 | 3163.43 | 588.70 | 2445.45 | 562.89 | 6.82 | 0.021 | 0.448 | 1.29 |
| RNGTT | 943.32 | 328.77 | 729.32 | 294.77 | 4.45 | 0.047 | 0.450 | 1.29 |
| ZNF622 | 1424.26 | 562.14 | 1102.25 | 502.25 | 9.29 | 0.011 | 0.460 | 1.29 |
| IMPDH1 | 197.21 | 21.10 | 152.65 | 9.59 | 4.62 | 0.044 | 0.447 | 1.29 |
| KIAA0922 | 674.92 | 115.20 | 522.66 | 88.86 | 9.66 | 0.011 | 0.461 | 1.29 |
| SLC25A3 | 140.75 | 7.00 | 109.01 | 9.76 | 6.08 | 0.026 | 0.449 | 1.29 |
| FANCD2 | 149.25 | 20.48 | 115.61 | 7.77 | 4.54 | 0.045 | 0.449 | 1.29 |
| RNF7 | 2297.16 | 858.00 | 1779.72 | 685.06 | 4.71 | 0.042 | 0.446 | 1.29 |
| BAT1 | 1859.91 | 290.32 | 1442.19 | 310.53 | 10.26 | 0.009 | 0.452 | 1.29 |
| SRFBP1 | 321.75 | 75.46 | 249.55 | 89.33 | 8.23 | 0.014 | 0.452 | 1.29 |
| NKIRAS1 | 242.76 | 34.85 | 188.33 | 19.52 | 4.75 | 0.042 | 0.444 | 1.29 |
| DNAJC18 | 166.19 | 18.68 | 128.95 | 24.48 | 10.15 | 0.010 | 0.453 | 1.29 |
| RBM10 | 2705.64 | 1082.35 | 2100.28 | 876.60 | 5.08 | 0.037 | 0.447 | 1.29 |
| PHEX | 144.99 | 10.06 | 112.56 | 12.54 | 19.96 | 0.002 | 0.449 | 1.29 |
| LOC645296 | 10080.40 | 2289.71 | 7827.14 | 2494.26 | 10.40 | 0.009 | 0.455 | 1.29 |
| LOC644517 | 174.98 | 13.90 | 135.87 | 2.63 | 5.04 | 0.037 | 0.447 | 1.29 |
| DNAJC11 | 140.70 | 17.42 | 109.30 | 7.68 | 4.44 | 0.047 | 0.449 | 1.29 |
| GTF3A | 4172.01 | 1576.31 | 3241.79 | 1299.85 | 5.40 | 0.033 | 0.445 | 1.29 |
| ATP5I | 5474.66 | 1228.49 | 4254.22 | 1257.75 | 30.79 | 0.001 | 0.465 | 1.29 |
| MTMR14 | 642.59 | 122.84 | 500.24 | 95.39 | 6.65 | 0.022 | 0.453 | 1.28 |
| ORC5L | 186.15 | 52.28 | 144.93 | 38.22 | 5.07 | 0.037 | 0.446 | 1.28 |
| LMNA | 671.76 | 187.46 | 523.18 | 156.32 | 8.03 | 0.015 | 0.452 | 1.28 |
| NCAPH2 | 309.80 | 37.19 | 241.40 | 39.29 | 7.64 | 0.017 | 0.446 | 1.28 |
| ZDHHC9 | 223.63 | 35.64 | 174.28 | 37.14 | 30.83 | 0.001 | 0.468 | 1.28 |
| SRRM1L | 318.40 | 50.19 | 248.38 | 58.97 | 11.88 | 0.007 | 0.444 | 1.28 |
| TLR9 | 186.09 | 30.02 | 145.18 | 20.21 | 4.97 | 0.038 | 0.446 | 1.28 |
| HS.567759 | 224.24 | 22.76 | 175.00 | 11.58 | 7.63 | 0.017 | 0.446 | 1.28 |
| SPAG7 | 387.29 | 76.24 | 302.29 | 77.57 | 5.56 | 0.031 | 0.445 | 1.28 |
| MGC16169 | 311.54 | 69.51 | 243.26 | 65.39 | 21.75 | 0.002 | 0.449 | 1.28 |
| ECE2 | 138.37 | 9.41 | 108.21 | 2.61 | 5.21 | 0.035 | 0.447 | 1.28 |
| LOC100130092 | 804.47 | 292.09 | 629.17 | 244.49 | 4.59 | 0.044 | 0.448 | 1.28 |
| IFI27L2 | 820.52 | 238.21 | 641.99 | 216.68 | 8.96 | 0.012 | 0.454 | 1.28 |
| PSPH | 143.26 | 19.98 | 112.13 | 13.97 | 8.28 | 0.014 | 0.452 | 1.28 |
| LOC100130233 | 143.78 | 3.54 | 112.58 | 9.44 | 9.02 | 0.012 | 0.455 | 1.28 |
| LOC100130131 | 176.26 | 9.92 | 138.07 | 3.41 | 8.76 | 0.013 | 0.456 | 1.28 |
| LOC100132795 | 7439.95 | 2322.91 | 5828.27 | 2382.56 | 10.79 | 0.008 | 0.455 | 1.28 |
| LOC401717 | 548.57 | 62.26 | 429.81 | 66.83 | 7.91 | 0.016 | 0.452 | 1.28 |
| DCTD | 331.71 | 70.70 | 259.99 | 61.28 | 9.90 | 0.010 | 0.457 | 1.28 |
| C12ORF5 | 442.69 | 105.09 | 347.18 | 118.62 | 7.33 | 0.018 | 0.450 | 1.28 |
| PSMB1 | 5585.98 | 1993.65 | 4390.40 | 2332.90 | 5.19 | 0.035 | 0.447 | 1.27 |
| PPIL5 | 149.90 | 30.81 | 117.86 | 24.27 | 5.93 | 0.027 | 0.447 | 1.27 |
| SFMBT1 | 217.23 | 32.28 | 170.83 | 25.42 | 7.44 | 0.018 | 0.448 | 1.27 |
| LOC647276 | 21225.30 | 3783.94 | 16707.70 | 5127.09 | 5.64 | 0.030 | 0.447 | 1.27 |
| AACS | 161.68 | 15.56 | 127.32 | 11.92 | 4.67 | 0.043 | 0.447 | 1.27 |
| ATP5G2 | 2132.93 | 462.07 | 1680.90 | 470.42 | 9.56 | 0.011 | 0.462 | 1.27 |
| SNORA70 | 295.67 | 59.75 | 233.30 | 43.73 | 6.29 | 0.024 | 0.450 | 1.27 |
| LOC647070 | 131.63 | 3.19 | 103.91 | 6.61 | 4.90 | 0.039 | 0.444 | 1.27 |
| INTS7 | 234.54 | 65.82 | 185.39 | 50.10 | 4.49 | 0.046 | 0.448 | 1.27 |
| FILIP1L | 163.88 | 24.34 | 129.57 | 14.00 | 5.60 | 0.030 | 0.445 | 1.26 |
| METTL6 | 160.89 | 32.54 | 127.25 | 22.50 | 4.87 | 0.040 | 0.445 | 1.26 |
| ADRM1 | 712.93 | 156.21 | 564.29 | 163.40 | 5.83 | 0.028 | 0.445 | 1.26 |
| MPV17 | 753.17 | 143.74 | 596.27 | 92.17 | 5.24 | 0.034 | 0.447 | 1.26 |
| SULT1A3 | 249.53 | 23.66 | 197.63 | 24.02 | 8.88 | 0.012 | 0.456 | 1.26 |
| RBM41 | 200.13 | 20.37 | 158.51 | 22.00 | 35.44 | 0.001 | 0.530 | 1.26 |
| NDUFB10 | 1729.95 | 708.69 | 1370.67 | 674.55 | 4.66 | 0.043 | 0.448 | 1.26 |
| RPS29 | 406.61 | 49.98 | 322.22 | 32.60 | 6.90 | 0.020 | 0.446 | 1.26 |
| ZZZ3 | 763.17 | 222.84 | 605.16 | 224.45 | 17.17 | 0.003 | 0.456 | 1.26 |
| GLYATL2 | 114.81 | 2.09 | 91.06 | 9.07 | 4.91 | 0.039 | 0.445 | 1.26 |
| NDUFA6 | 546.51 | 150.68 | 433.51 | 128.01 | 7.85 | 0.016 | 0.450 | 1.26 |
| NCRNA00158 | 145.88 | 6.96 | 115.74 | 9.24 | 22.83 | 0.002 | 0.454 | 1.26 |
| LOC648099 | 264.67 | 41.07 | 210.30 | 47.71 | 14.15 | 0.005 | 0.448 | 1.26 |
| MRPL53 | 495.48 | 118.88 | 393.86 | 131.67 | 13.36 | 0.006 | 0.445 | 1.26 |
| LOC440589 | 15233.25 | 5299.64 | 12112.06 | 5301.57 | 8.46 | 0.014 | 0.454 | 1.26 |
| RAD1 | 218.82 | 45.50 | 173.99 | 34.63 | 4.88 | 0.040 | 0.444 | 1.26 |
| C5ORF51 | 1105.23 | 395.93 | 878.86 | 361.36 | 11.31 | 0.008 | 0.454 | 1.26 |
| SLC1A4 | 209.82 | 35.31 | 167.11 | 45.84 | 5.40 | 0.033 | 0.445 | 1.26 |
| ZNF35 | 239.41 | 60.59 | 190.77 | 42.59 | 4.67 | 0.043 | 0.447 | 1.25 |
| ZNRD1 | 257.79 | 49.92 | 205.63 | 47.85 | 5.03 | 0.037 | 0.447 | 1.25 |
| DCI | 328.73 | 68.05 | 262.38 | 81.30 | 7.72 | 0.016 | 0.449 | 1.25 |
| SNORA41 | 179.64 | 25.14 | 143.42 | 16.68 | 5.99 | 0.027 | 0.448 | 1.25 |
| MAGED2 | 148.43 | 3.24 | 118.51 | 13.13 | 5.07 | 0.037 | 0.446 | 1.25 |
| TM2D3 | 991.09 | 297.92 | 791.57 | 291.85 | 5.94 | 0.027 | 0.447 | 1.25 |
| LOC646791 | 163.01 | 7.71 | 130.20 | 4.07 | 4.97 | 0.038 | 0.445 | 1.25 |
| ZC3H18 | 248.14 | 61.17 | 198.36 | 43.02 | 4.61 | 0.044 | 0.448 | 1.25 |
| TBL2 | 220.77 | 47.12 | 176.67 | 56.52 | 4.86 | 0.040 | 0.445 | 1.25 |
| PEF1 | 478.76 | 131.96 | 383.58 | 132.19 | 6.11 | 0.026 | 0.450 | 1.25 |
| GLS | 383.45 | 92.94 | 307.44 | 81.23 | 5.89 | 0.028 | 0.446 | 1.25 |
| LOC389672 | 230.43 | 34.40 | 184.83 | 24.13 | 4.60 | 0.044 | 0.447 | 1.25 |
| WBSCR16 | 216.64 | 17.05 | 173.83 | 23.73 | 6.68 | 0.022 | 0.452 | 1.25 |
| RARS2 | 346.27 | 76.53 | 278.16 | 64.96 | 4.69 | 0.043 | 0.447 | 1.24 |
| LOC100129297 | 181.92 | 1.92 | 146.18 | 2.06 | 18.45 | 0.003 | 0.453 | 1.24 |
| MAP4K5 | 481.27 | 128.72 | 386.79 | 97.25 | 5.08 | 0.037 | 0.446 | 1.24 |
| RAMP1 | 120.64 | 10.78 | 96.98 | 6.28 | 7.59 | 0.017 | 0.445 | 1.24 |
| DCTD | 1114.01 | 418.22 | 895.61 | 363.61 | 5.16 | 0.036 | 0.447 | 1.24 |
| MIPEP | 219.50 | 40.82 | 176.55 | 30.12 | 5.96 | 0.027 | 0.448 | 1.24 |
| NSUN5 | 212.71 | 16.57 | 171.12 | 22.86 | 9.27 | 0.011 | 0.459 | 1.24 |
| RPS26L | 9314.66 | 3393.17 | 7496.57 | 3045.54 | 5.78 | 0.029 | 0.444 | 1.24 |
| LOC100128425 | 182.46 | 16.25 | 146.88 | 12.97 | 5.72 | 0.029 | 0.446 | 1.24 |
| APIP | 323.90 | 95.17 | 260.85 | 108.33 | 8.18 | 0.015 | 0.452 | 1.24 |
| C11ORF59 | 1310.63 | 282.59 | 1055.66 | 304.80 | 10.28 | 0.009 | 0.452 | 1.24 |
| SFXN2 | 143.86 | 15.22 | 115.95 | 10.97 | 5.98 | 0.027 | 0.448 | 1.24 |
| LOC100134101 | 131.45 | 2.68 | 105.95 | 4.07 | 6.57 | 0.022 | 0.454 | 1.24 |
| BPNT1 | 172.42 | 27.60 | 138.99 | 30.34 | 16.14 | 0.004 | 0.457 | 1.24 |
| SFRS15 | 522.01 | 156.66 | 421.00 | 123.28 | 5.24 | 0.035 | 0.447 | 1.24 |
| LOC643387 | 757.80 | 308.12 | 611.26 | 296.45 | 5.37 | 0.033 | 0.445 | 1.24 |
| CKLF | 1707.14 | 365.00 | 1377.57 | 459.27 | 6.02 | 0.026 | 0.449 | 1.24 |
| SYNGR2 | 123.86 | 9.18 | 100.03 | 7.11 | 12.63 | 0.006 | 0.440 | 1.24 |
| FBXO31 | 577.93 | 131.90 | 466.94 | 145.92 | 5.63 | 0.030 | 0.446 | 1.24 |
| LOC440396 | 253.14 | 56.08 | 204.58 | 46.05 | 4.80 | 0.041 | 0.444 | 1.24 |
| GPRIN1 | 132.12 | 6.19 | 106.78 | 2.93 | 8.27 | 0.014 | 0.452 | 1.24 |
| CLSPN | 128.40 | 14.95 | 103.80 | 11.22 | 11.37 | 0.008 | 0.451 | 1.24 |
| CTDP1 | 257.88 | 15.50 | 208.68 | 25.62 | 5.81 | 0.028 | 0.444 | 1.24 |
| PSMA6 | 1754.60 | 643.11 | 1420.34 | 581.27 | 4.92 | 0.039 | 0.445 | 1.24 |
| NARG2 | 150.15 | 21.97 | 121.58 | 11.16 | 4.49 | 0.046 | 0.448 | 1.23 |
| PRCC | 218.45 | 3.99 | 176.91 | 10.01 | 5.26 | 0.034 | 0.448 | 1.23 |
| TOB2 | 202.04 | 10.54 | 163.67 | 2.99 | 8.48 | 0.014 | 0.453 | 1.23 |
| MPV17L2 | 147.28 | 4.85 | 119.36 | 8.88 | 11.15 | 0.008 | 0.455 | 1.23 |
| DCXR | 601.66 | 66.75 | 487.59 | 106.47 | 4.97 | 0.038 | 0.446 | 1.23 |
| YIF1A | 590.61 | 173.31 | 478.73 | 184.43 | 13.04 | 0.006 | 0.444 | 1.23 |
| ARRDC2 | 215.01 | 48.60 | 174.28 | 34.61 | 5.04 | 0.037 | 0.447 | 1.23 |
| UBE2CBP | 176.70 | 26.57 | 143.33 | 16.02 | 4.39 | 0.048 | 0.451 | 1.23 |
| FBXL6 | 272.23 | 47.04 | 220.86 | 33.13 | 6.19 | 0.025 | 0.449 | 1.23 |
| ATF6 | 941.26 | 364.89 | 763.69 | 337.26 | 5.43 | 0.032 | 0.444 | 1.23 |
| GCDH | 190.81 | 6.55 | 154.90 | 11.42 | 10.15 | 0.010 | 0.454 | 1.23 |
| LOC649330 | 247.49 | 48.44 | 200.95 | 62.20 | 4.97 | 0.038 | 0.446 | 1.23 |
| HSPC159 | 141.15 | 12.66 | 114.61 | 13.57 | 4.35 | 0.049 | 0.452 | 1.23 |
| LOC402057 | 15700.53 | 4364.12 | 12751.70 | 4657.45 | 9.58 | 0.011 | 0.462 | 1.23 |
| R3HCC1 | 428.84 | 79.86 | 348.31 | 93.92 | 6.54 | 0.023 | 0.454 | 1.23 |
| LOC100131866 | 514.09 | 139.40 | 417.99 | 137.26 | 12.68 | 0.006 | 0.440 | 1.23 |
| GSTM1 | 610.15 | 97.49 | 496.22 | 78.57 | 5.39 | 0.033 | 0.445 | 1.23 |
| HCFC1R1 | 885.64 | 331.13 | 720.28 | 278.85 | 4.34 | 0.049 | 0.452 | 1.23 |
| IFFO2 | 182.41 | 16.08 | 148.36 | 8.98 | 8.25 | 0.014 | 0.452 | 1.23 |
| TNFRSF18 | 144.34 | 3.72 | 117.46 | 10.50 | 4.78 | 0.041 | 0.444 | 1.23 |
| DNMT3A | 157.12 | 8.02 | 128.18 | 14.66 | 6.88 | 0.020 | 0.445 | 1.23 |
| ZNF232 | 211.75 | 37.22 | 172.89 | 26.18 | 4.75 | 0.042 | 0.444 | 1.22 |
| NUPL1 | 143.13 | 15.05 | 116.88 | 12.81 | 12.31 | 0.007 | 0.439 | 1.22 |
| ATP5S | 196.55 | 23.35 | 160.54 | 24.72 | 10.36 | 0.009 | 0.452 | 1.22 |
| RPS8 | 10001.05 | 2731.66 | 8172.64 | 3371.05 | 4.39 | 0.048 | 0.451 | 1.22 |
| RPL6 | 26023.27 | 5856.06 | 21265.97 | 7107.42 | 6.28 | 0.024 | 0.449 | 1.22 |
| RAB4A | 163.01 | 22.51 | 133.27 | 26.77 | 9.58 | 0.011 | 0.462 | 1.22 |
| HIST1H3I | 127.08 | 2.71 | 103.95 | 7.82 | 7.41 | 0.018 | 0.448 | 1.22 |
| LOC388564 | 294.81 | 34.67 | 241.55 | 14.68 | 4.55 | 0.045 | 0.449 | 1.22 |
| XBP1 | 2380.69 | 719.83 | 1952.33 | 796.89 | 6.80 | 0.021 | 0.448 | 1.22 |
| LOC100132728 | 140.54 | 9.43 | 115.26 | 5.98 | 4.89 | 0.039 | 0.444 | 1.22 |
| LOC731096 | 10247.42 | 2759.40 | 8407.49 | 2617.30 | 5.40 | 0.033 | 0.445 | 1.22 |
| L1TD1 | 147.51 | 13.00 | 121.06 | 7.36 | 6.68 | 0.022 | 0.452 | 1.22 |
| RPL36AL | 4412.21 | 1681.76 | 3621.19 | 1406.58 | 4.95 | 0.038 | 0.445 | 1.22 |
| RPL35 | 9381.91 | 2339.77 | 7709.89 | 2581.04 | 11.20 | 0.008 | 0.456 | 1.22 |
| FAM86B1 | 154.50 | 14.13 | 127.02 | 14.90 | 42.50 | 0.001 | 0.608 | 1.22 |
| INO80B | 212.95 | 15.80 | 175.13 | 11.99 | 5.51 | 0.031 | 0.446 | 1.22 |
| LOC100130179 | 142.62 | 6.55 | 117.42 | 8.70 | 7.81 | 0.016 | 0.449 | 1.21 |
| TIAM1 | 175.08 | 20.56 | 144.18 | 26.44 | 7.62 | 0.017 | 0.446 | 1.21 |
| DDX49 | 183.35 | 13.34 | 151.20 | 8.82 | 4.34 | 0.049 | 0.452 | 1.21 |
| UCRC | 265.27 | 58.29 | 219.28 | 40.62 | 4.51 | 0.046 | 0.449 | 1.21 |
| LOC650185 | 126.86 | 19.01 | 104.87 | 13.93 | 6.17 | 0.025 | 0.448 | 1.21 |
| RSRC2 | 1905.05 | 797.98 | 1576.27 | 756.77 | 5.81 | 0.028 | 0.444 | 1.21 |
| LOC644934 | 3592.01 | 1406.36 | 2972.49 | 1295.25 | 6.07 | 0.026 | 0.449 | 1.21 |
| TNFRSF1B | 1804.56 | 601.65 | 1494.42 | 620.14 | 4.56 | 0.045 | 0.449 | 1.21 |
| HS.443490 | 262.91 | 77.38 | 217.79 | 76.57 | 10.06 | 0.010 | 0.454 | 1.21 |
| LOC649214 | 150.51 | 12.62 | 124.69 | 8.86 | 4.53 | 0.046 | 0.449 | 1.21 |
| DUX4 | 139.95 | 12.74 | 115.94 | 15.85 | 13.30 | 0.006 | 0.445 | 1.21 |
| SLC16A9 | 139.12 | 7.01 | 115.25 | 12.08 | 7.87 | 0.016 | 0.450 | 1.21 |
| C11ORF51 | 208.04 | 22.82 | 172.44 | 19.71 | 4.46 | 0.047 | 0.450 | 1.21 |
| TARBP2 | 180.37 | 10.85 | 149.58 | 13.76 | 6.35 | 0.024 | 0.452 | 1.21 |
| SLMAP | 715.98 | 252.65 | 594.28 | 225.89 | 5.81 | 0.028 | 0.444 | 1.20 |
| SH2D3A | 151.01 | 6.69 | 125.38 | 14.64 | 4.30 | 0.050 | 0.452 | 1.20 |
| WDR89 | 123.01 | 4.31 | 102.14 | 3.84 | 5.10 | 0.036 | 0.446 | 1.20 |
| UNG | 117.23 | 3.78 | 97.38 | 4.18 | 5.77 | 0.029 | 0.444 | 1.20 |
| CYP11B1 | 126.73 | 19.94 | 105.32 | 14.27 | 5.92 | 0.027 | 0.448 | 1.20 |
| GAK | 122.36 | 7.34 | 101.70 | 12.30 | 4.81 | 0.041 | 0.444 | 1.20 |
| ISCA1L | 335.62 | 70.21 | 279.26 | 55.54 | 6.40 | 0.024 | 0.453 | 1.20 |
| FAM40B | 124.03 | 8.69 | 103.27 | 4.11 | 6.41 | 0.023 | 0.454 | 1.20 |
| ALG1 | 129.68 | 12.13 | 108.05 | 5.49 | 5.08 | 0.037 | 0.446 | 1.20 |
| BOLA2 | 131.86 | 4.68 | 110.09 | 5.91 | 9.31 | 0.011 | 0.462 | 1.20 |
| MLEC | 514.75 | 117.00 | 429.83 | 115.71 | 8.70 | 0.013 | 0.453 | 1.20 |
| PCBD1 | 255.94 | 26.49 | 213.84 | 29.10 | 5.61 | 0.030 | 0.445 | 1.20 |
| NUP98 | 251.78 | 33.09 | 210.54 | 28.91 | 8.56 | 0.013 | 0.451 | 1.20 |
| MRPL4 | 175.65 | 10.62 | 147.02 | 12.46 | 8.31 | 0.014 | 0.452 | 1.19 |
| MORF4L1 | 3624.18 | 1304.91 | 3033.48 | 1293.51 | 16.42 | 0.004 | 0.453 | 1.19 |
| HS.359754 | 124.95 | 15.31 | 104.58 | 19.06 | 5.19 | 0.035 | 0.447 | 1.19 |
| LPIN1 | 1037.65 | 472.67 | 870.05 | 441.93 | 8.19 | 0.015 | 0.453 | 1.19 |
| LOC729466 | 7534.01 | 2708.61 | 6317.74 | 2257.45 | 4.67 | 0.043 | 0.447 | 1.19 |
| SDHAF1 | 407.74 | 91.99 | 341.98 | 89.35 | 5.06 | 0.037 | 0.446 | 1.19 |
| LOC728275 | 146.58 | 11.44 | 122.99 | 2.49 | 4.50 | 0.046 | 0.449 | 1.19 |
| UBE3C | 520.58 | 127.09 | 436.81 | 155.13 | 4.54 | 0.045 | 0.449 | 1.19 |
| HHLA3 | 136.03 | 8.07 | 114.16 | 9.28 | 12.50 | 0.006 | 0.444 | 1.19 |
| LOC729433 | 130.24 | 20.23 | 109.31 | 18.27 | 17.93 | 0.003 | 0.449 | 1.19 |
| UBE2MP1 | 127.03 | 5.08 | 106.64 | 3.42 | 4.91 | 0.039 | 0.445 | 1.19 |
| ZCCHC16 | 104.29 | 3.80 | 87.61 | 2.78 | 19.46 | 0.003 | 0.455 | 1.19 |
| LOC731511 | 128.54 | 3.32 | 108.05 | 5.25 | 16.45 | 0.004 | 0.456 | 1.19 |
| PTPN2 | 229.47 | 55.35 | 192.87 | 43.57 | 4.34 | 0.049 | 0.452 | 1.19 |
| FARSB | 123.45 | 1.20 | 103.85 | 6.58 | 4.60 | 0.044 | 0.448 | 1.19 |
| AHI1 | 201.25 | 49.81 | 169.33 | 51.50 | 5.59 | 0.030 | 0.445 | 1.19 |
| LOC723805 | 140.21 | 4.58 | 117.99 | 1.29 | 6.90 | 0.020 | 0.446 | 1.19 |
| COPZ1 | 498.36 | 103.37 | 419.37 | 99.59 | 4.35 | 0.049 | 0.452 | 1.19 |
| OGDH | 119.26 | 3.49 | 100.38 | 6.50 | 10.87 | 0.008 | 0.456 | 1.19 |
| SDR42E1 | 134.71 | 2.64 | 113.56 | 7.06 | 6.05 | 0.026 | 0.448 | 1.19 |
| LOC729313 | 130.97 | 3.23 | 110.45 | 2.02 | 8.14 | 0.015 | 0.451 | 1.19 |
| SMARCD1 | 118.47 | 3.86 | 99.91 | 4.38 | 13.26 | 0.006 | 0.445 | 1.19 |
| DOCK10 | 2678.16 | 1034.02 | 2259.07 | 1038.55 | 8.41 | 0.014 | 0.451 | 1.19 |
| CAMLG | 1630.41 | 566.44 | 1375.58 | 529.80 | 4.31 | 0.050 | 0.452 | 1.19 |
| RAD51L3 | 154.87 | 6.44 | 130.88 | 8.10 | 6.06 | 0.026 | 0.448 | 1.18 |
| PEX26 | 124.93 | 14.61 | 105.63 | 11.47 | 6.93 | 0.020 | 0.446 | 1.18 |
| LYRM4 | 245.67 | 61.10 | 208.00 | 51.48 | 4.88 | 0.039 | 0.444 | 1.18 |
| SLC9A4 | 147.86 | 13.58 | 125.32 | 9.56 | 4.38 | 0.048 | 0.451 | 1.18 |
| WNT10B | 118.78 | 3.25 | 100.69 | 6.99 | 7.70 | 0.016 | 0.449 | 1.18 |
| PRAMEF7 | 134.22 | 3.21 | 113.79 | 7.09 | 8.81 | 0.013 | 0.457 | 1.18 |
| LOC649445 | 165.99 | 13.24 | 140.75 | 16.05 | 9.38 | 0.011 | 0.462 | 1.18 |
| PFN1 | 19754.98 | 5674.68 | 16753.49 | 6196.98 | 8.78 | 0.013 | 0.457 | 1.18 |
| SPANXA1 | 107.80 | 9.67 | 91.52 | 6.89 | 10.00 | 0.010 | 0.453 | 1.18 |
| LOC284821 | 2579.57 | 811.18 | 2190.27 | 932.68 | 4.86 | 0.040 | 0.445 | 1.18 |
| OGFOD1 | 117.59 | 3.10 | 99.87 | 3.54 | 5.68 | 0.030 | 0.446 | 1.18 |
| ALKBH8 | 196.02 | 42.85 | 166.48 | 38.02 | 5.11 | 0.036 | 0.447 | 1.18 |
| STMN1 | 125.80 | 12.59 | 106.90 | 11.70 | 4.84 | 0.040 | 0.444 | 1.18 |
| HIST1H4K | 169.90 | 8.33 | 144.42 | 2.31 | 4.49 | 0.046 | 0.449 | 1.18 |
| ARHGAP30 | 1134.28 | 400.23 | 964.31 | 357.59 | 6.87 | 0.021 | 0.446 | 1.18 |
| SHMT1 | 115.81 | 8.40 | 98.48 | 4.03 | 6.64 | 0.022 | 0.453 | 1.18 |
| LOC654123 | 155.32 | 8.50 | 132.13 | 9.71 | 8.51 | 0.014 | 0.451 | 1.18 |
| NBN | 939.73 | 363.99 | 799.51 | 323.18 | 5.72 | 0.029 | 0.446 | 1.18 |
| CREB3L4 | 123.44 | 1.75 | 105.11 | 5.10 | 7.65 | 0.017 | 0.447 | 1.17 |
| THYN1 | 135.46 | 20.23 | 115.42 | 13.28 | 4.77 | 0.041 | 0.444 | 1.17 |
| POMGNT1 | 571.43 | 167.29 | 487.07 | 141.87 | 5.41 | 0.033 | 0.445 | 1.17 |
| ATP5D | 260.69 | 45.71 | 222.32 | 39.93 | 9.12 | 0.012 | 0.457 | 1.17 |
| ZNF286A | 133.17 | 7.62 | 113.57 | 9.30 | 4.85 | 0.040 | 0.444 | 1.17 |
| NACA2 | 219.65 | 26.00 | 187.35 | 21.25 | 11.77 | 0.007 | 0.448 | 1.17 |
| MCTP2 | 126.42 | 11.33 | 107.93 | 7.34 | 7.11 | 0.019 | 0.449 | 1.17 |
| LOC100133435 | 147.27 | 8.51 | 125.84 | 2.06 | 4.89 | 0.039 | 0.444 | 1.17 |
| HS.71947 | 116.43 | 9.74 | 99.53 | 8.96 | 8.90 | 0.012 | 0.456 | 1.17 |
| LOC652495 | 152.43 | 16.52 | 130.30 | 17.67 | 16.99 | 0.003 | 0.454 | 1.17 |
| MAT2B | 125.25 | 2.09 | 107.10 | 3.28 | 13.84 | 0.005 | 0.451 | 1.17 |
| DENND1A | 133.58 | 10.89 | 114.23 | 8.03 | 5.36 | 0.033 | 0.446 | 1.17 |
| DHODH | 122.91 | 2.64 | 105.19 | 5.00 | 4.94 | 0.039 | 0.446 | 1.17 |
| DHRS11 | 167.22 | 4.70 | 143.25 | 7.54 | 5.96 | 0.027 | 0.448 | 1.17 |
| ADK | 141.58 | 14.16 | 121.30 | 10.34 | 8.87 | 0.012 | 0.456 | 1.17 |
| FOXK2 | 117.24 | 9.27 | 100.47 | 13.25 | 6.97 | 0.020 | 0.448 | 1.17 |
| CAPZA3 | 118.55 | 8.96 | 101.62 | 10.24 | 10.68 | 0.009 | 0.453 | 1.17 |
| WT1 | 124.90 | 6.33 | 107.09 | 0.33 | 5.14 | 0.036 | 0.446 | 1.17 |
| EIF4E | 114.55 | 8.08 | 98.25 | 11.19 | 7.38 | 0.018 | 0.449 | 1.17 |
| HS.556082 | 204.26 | 45.47 | 175.23 | 37.11 | 6.00 | 0.027 | 0.448 | 1.17 |
| ALG10B | 135.83 | 18.83 | 116.54 | 18.12 | 9.37 | 0.011 | 0.462 | 1.17 |
| LOC644511 | 2454.73 | 140.35 | 2107.23 | 250.72 | 5.30 | 0.034 | 0.447 | 1.16 |
| ARF3 | 407.70 | 114.97 | 350.04 | 107.36 | 8.09 | 0.015 | 0.452 | 1.16 |
| CHP | 647.08 | 135.31 | 555.59 | 159.66 | 5.88 | 0.028 | 0.446 | 1.16 |
| NDN | 189.42 | 11.80 | 162.68 | 15.50 | 10.56 | 0.009 | 0.454 | 1.16 |
| FKBP7 | 107.31 | 8.17 | 92.18 | 4.89 | 6.06 | 0.026 | 0.448 | 1.16 |
| LOC728693 | 10057.65 | 2461.49 | 8641.16 | 2234.01 | 5.11 | 0.036 | 0.447 | 1.16 |
| LOC652826 | 215.88 | 2.90 | 185.50 | 7.84 | 6.21 | 0.025 | 0.449 | 1.16 |
| FBXL10 | 2370.71 | 689.30 | 2037.69 | 690.44 | 7.19 | 0.019 | 0.450 | 1.16 |
| ALG3 | 342.93 | 56.15 | 294.79 | 45.25 | 6.76 | 0.021 | 0.448 | 1.16 |
| LOC100128221 | 126.34 | 7.55 | 108.65 | 12.75 | 5.89 | 0.028 | 0.446 | 1.16 |
| RHD | 106.96 | 8.79 | 92.02 | 6.20 | 8.72 | 0.013 | 0.455 | 1.16 |
| FLJ32810 | 132.80 | 7.06 | 114.29 | 7.66 | 19.34 | 0.003 | 0.456 | 1.16 |
| ASB13 | 155.41 | 25.26 | 133.83 | 20.33 | 6.05 | 0.026 | 0.449 | 1.16 |
| MATN4 | 105.38 | 1.48 | 90.84 | 2.46 | 8.24 | 0.014 | 0.452 | 1.16 |
| ADARB1 | 158.92 | 15.39 | 137.01 | 8.98 | 4.47 | 0.047 | 0.449 | 1.16 |
| RPL18A | 19953.10 | 3404.26 | 17204.26 | 3883.16 | 8.62 | 0.013 | 0.451 | 1.16 |
| NFATC3 | 152.16 | 18.31 | 131.37 | 11.01 | 4.85 | 0.040 | 0.444 | 1.16 |
| ELA3A | 111.08 | 0.59 | 95.99 | 4.85 | 5.90 | 0.028 | 0.447 | 1.16 |
| C9ORF93 | 130.65 | 2.58 | 112.94 | 4.49 | 4.36 | 0.049 | 0.452 | 1.16 |
| LOC100129269 | 139.56 | 11.70 | 120.71 | 8.04 | 7.30 | 0.018 | 0.450 | 1.16 |
| ASNA1 | 134.34 | 12.77 | 116.20 | 7.68 | 4.88 | 0.040 | 0.444 | 1.16 |
| MIR106B | 113.01 | 2.63 | 97.77 | 6.21 | 6.67 | 0.022 | 0.452 | 1.16 |
| MRO | 110.19 | 8.11 | 95.33 | 4.92 | 6.75 | 0.021 | 0.449 | 1.16 |
| LOC100129206 | 111.54 | 6.27 | 96.57 | 10.00 | 4.77 | 0.041 | 0.444 | 1.16 |
| ZNF519 | 117.57 | 7.55 | 101.88 | 4.53 | 5.26 | 0.034 | 0.448 | 1.15 |
| CUGBP1 | 191.34 | 44.75 | 165.80 | 40.43 | 8.49 | 0.014 | 0.453 | 1.15 |
| LOC199800 | 149.96 | 9.02 | 129.97 | 10.02 | 10.54 | 0.009 | 0.455 | 1.15 |
| DPYSL4 | 118.85 | 12.57 | 103.00 | 9.67 | 5.21 | 0.035 | 0.447 | 1.15 |
| SERP2 | 120.56 | 11.85 | 104.53 | 6.27 | 4.93 | 0.039 | 0.445 | 1.15 |
| LOC388556 | 9120.00 | 2253.09 | 7910.19 | 2302.49 | 36.71 | 0.001 | 0.515 | 1.15 |
| DEFB124 | 115.59 | 4.50 | 100.26 | 0.47 | 6.55 | 0.023 | 0.454 | 1.15 |
| ELSPBP1 | 106.27 | 5.19 | 92.23 | 4.58 | 5.56 | 0.031 | 0.445 | 1.15 |
| SCAMP3 | 163.52 | 31.25 | 141.94 | 24.18 | 4.80 | 0.041 | 0.444 | 1.15 |
| PPP2R5D | 138.43 | 7.75 | 120.18 | 4.76 | 4.49 | 0.046 | 0.449 | 1.15 |
| SART3 | 170.96 | 12.11 | 148.44 | 19.69 | 4.31 | 0.050 | 0.452 | 1.15 |
| C11ORF73 | 210.05 | 41.47 | 182.42 | 43.79 | 4.43 | 0.047 | 0.450 | 1.15 |
| GEN1 | 124.42 | 4.44 | 108.07 | 10.89 | 4.39 | 0.048 | 0.451 | 1.15 |
| GTDC1 | 260.35 | 44.84 | 226.17 | 53.34 | 5.69 | 0.030 | 0.446 | 1.15 |
| DJ222E13.2 | 126.88 | 11.96 | 110.25 | 11.99 | 8.77 | 0.013 | 0.457 | 1.15 |
| LOC653557 | 175.93 | 10.30 | 152.88 | 3.26 | 5.31 | 0.034 | 0.447 | 1.15 |
| LOC652344 | 120.32 | 4.85 | 104.61 | 2.42 | 8.80 | 0.013 | 0.457 | 1.15 |
| GRAP | 128.29 | 10.68 | 111.55 | 5.07 | 5.07 | 0.037 | 0.446 | 1.15 |
| FLJ25715 | 129.89 | 22.69 | 112.94 | 16.07 | 4.44 | 0.047 | 0.450 | 1.15 |
| AGAP1 | 111.85 | 4.38 | 97.26 | 1.01 | 6.53 | 0.023 | 0.453 | 1.15 |
| KIF9 | 114.53 | 9.27 | 99.60 | 6.60 | 5.34 | 0.033 | 0.446 | 1.15 |
| KIAA0895 | 125.90 | 9.09 | 109.55 | 11.77 | 10.38 | 0.009 | 0.454 | 1.15 |
| TRAF7 | 109.42 | 7.04 | 95.22 | 6.11 | 5.20 | 0.035 | 0.447 | 1.15 |
| LOC391655 | 185.52 | 22.22 | 161.80 | 17.03 | 7.08 | 0.019 | 0.450 | 1.15 |
| LOC650739 | 255.94 | 22.14 | 223.23 | 30.82 | 4.96 | 0.038 | 0.446 | 1.15 |
| CLUL1 | 110.62 | 4.83 | 96.50 | 2.14 | 5.11 | 0.036 | 0.447 | 1.15 |
| SLC7A3 | 178.06 | 28.37 | 155.34 | 29.32 | 31.46 | 0.001 | 0.487 | 1.15 |
| AFAP1L2 | 131.54 | 21.28 | 114.79 | 16.44 | 5.98 | 0.027 | 0.448 | 1.15 |
| LOC100133017 | 152.63 | 12.38 | 133.33 | 9.29 | 10.78 | 0.008 | 0.454 | 1.14 |
| GTF3C4 | 125.79 | 2.03 | 109.97 | 4.94 | 8.98 | 0.012 | 0.454 | 1.14 |
| STK16 | 118.97 | 10.53 | 104.04 | 6.98 | 6.00 | 0.027 | 0.447 | 1.14 |
| SEMA7A | 165.73 | 3.99 | 144.94 | 9.57 | 5.43 | 0.032 | 0.445 | 1.14 |
| CDC2L1 | 113.31 | 9.02 | 99.09 | 4.99 | 4.37 | 0.049 | 0.451 | 1.14 |
| MASP1 | 127.32 | 8.39 | 111.41 | 8.90 | 22.40 | 0.002 | 0.449 | 1.14 |
| PLCG2 | 1796.31 | 703.11 | 1572.28 | 783.35 | 4.80 | 0.041 | 0.444 | 1.14 |
| TMEM136 | 112.40 | 8.86 | 98.39 | 8.32 | 30.75 | 0.001 | 0.462 | 1.14 |
| COMMD5 | 484.21 | 169.66 | 424.17 | 169.16 | 6.50 | 0.023 | 0.452 | 1.14 |
| LOC641746 | 161.58 | 34.29 | 141.57 | 32.79 | 4.62 | 0.044 | 0.447 | 1.14 |
| PTPN22 | 478.70 | 193.03 | 420.05 | 201.52 | 10.79 | 0.008 | 0.456 | 1.14 |
| LASS2 | 746.90 | 206.13 | 655.41 | 210.58 | 22.47 | 0.002 | 0.451 | 1.14 |
| CDC42SE1 | 132.53 | 8.97 | 116.31 | 10.89 | 14.67 | 0.005 | 0.444 | 1.14 |
| THRA | 114.37 | 6.84 | 100.42 | 2.93 | 4.77 | 0.041 | 0.444 | 1.14 |
| ME3 | 134.83 | 8.44 | 118.41 | 12.51 | 6.74 | 0.021 | 0.448 | 1.14 |
| LOC100132503 | 160.52 | 3.62 | 140.98 | 8.74 | 5.68 | 0.030 | 0.446 | 1.14 |
| TAC1 | 122.47 | 5.40 | 107.60 | 10.12 | 4.57 | 0.045 | 0.448 | 1.14 |
| WDR78 | 110.41 | 8.31 | 97.05 | 4.00 | 4.63 | 0.044 | 0.447 | 1.14 |
| COX11 | 126.20 | 12.41 | 110.93 | 8.83 | 6.80 | 0.021 | 0.448 | 1.14 |
| PRMT7 | 141.71 | 21.32 | 124.68 | 17.75 | 6.60 | 0.022 | 0.453 | 1.14 |
| TMCC2 | 111.94 | 7.20 | 98.54 | 5.94 | 6.19 | 0.025 | 0.449 | 1.14 |
| FLJ40244 | 103.13 | 6.15 | 90.85 | 7.67 | 7.78 | 0.016 | 0.448 | 1.14 |
| ACSM3 | 120.03 | 11.64 | 105.76 | 6.88 | 4.94 | 0.039 | 0.446 | 1.13 |
| LOC387841 | 858.04 | 251.55 | 756.25 | 258.26 | 7.67 | 0.017 | 0.448 | 1.13 |
| HDLBP | 108.85 | 2.82 | 95.95 | 1.97 | 15.80 | 0.004 | 0.449 | 1.13 |
| IPPK | 136.50 | 13.67 | 120.32 | 12.44 | 7.72 | 0.016 | 0.448 | 1.13 |
| CST11 | 105.50 | 3.90 | 93.07 | 2.88 | 19.11 | 0.003 | 0.449 | 1.13 |
| ZNF681 | 200.75 | 33.15 | 177.13 | 37.57 | 5.16 | 0.036 | 0.447 | 1.13 |
| SDHC | 810.25 | 227.25 | 715.25 | 198.83 | 5.79 | 0.029 | 0.444 | 1.13 |
| HIST1H2AH | 112.70 | 9.72 | 99.52 | 6.98 | 6.13 | 0.026 | 0.449 | 1.13 |
| MAPBPIP | 609.70 | 185.68 | 538.64 | 168.20 | 7.01 | 0.020 | 0.449 | 1.13 |
| ATXN8OS | 107.30 | 7.69 | 94.81 | 7.94 | 6.55 | 0.023 | 0.454 | 1.13 |
| BCAS4 | 733.76 | 222.09 | 648.85 | 197.19 | 4.80 | 0.041 | 0.444 | 1.13 |
| C10ORF59 | 138.69 | 13.76 | 122.66 | 9.47 | 5.39 | 0.033 | 0.445 | 1.13 |
| CHPF | 165.15 | 1.93 | 146.08 | 5.73 | 4.64 | 0.044 | 0.447 | 1.13 |
| ANAPC1 | 207.12 | 24.64 | 183.21 | 29.00 | 9.49 | 0.011 | 0.464 | 1.13 |
| CABP5 | 120.09 | 9.17 | 106.26 | 8.66 | 15.67 | 0.004 | 0.453 | 1.13 |
| SETD1A | 431.43 | 71.54 | 381.76 | 65.01 | 8.76 | 0.013 | 0.457 | 1.13 |
| COPS7A | 1340.95 | 454.68 | 1186.93 | 417.03 | 5.82 | 0.028 | 0.444 | 1.13 |
| SLC35B2 | 444.37 | 105.21 | 393.77 | 94.86 | 8.33 | 0.014 | 0.452 | 1.13 |
| FLJ46347 | 104.02 | 6.38 | 92.20 | 2.67 | 4.78 | 0.041 | 0.444 | 1.13 |
| PIGR | 119.89 | 2.68 | 106.27 | 7.37 | 4.82 | 0.040 | 0.445 | 1.13 |
| LHFPL1 | 134.07 | 12.47 | 118.90 | 14.07 | 7.29 | 0.018 | 0.450 | 1.13 |
| CLSTN1 | 108.34 | 3.43 | 96.12 | 3.66 | 5.70 | 0.029 | 0.445 | 1.13 |
| C1ORF93 | 186.71 | 20.57 | 165.69 | 24.27 | 6.70 | 0.022 | 0.450 | 1.13 |
| CXORF52 | 113.55 | 6.65 | 100.76 | 7.93 | 7.61 | 0.017 | 0.446 | 1.13 |
| SULT1A1 | 109.68 | 14.25 | 97.34 | 10.40 | 5.41 | 0.033 | 0.445 | 1.13 |
| MAGEA9B | 132.42 | 10.83 | 117.54 | 14.49 | 7.05 | 0.020 | 0.449 | 1.13 |
| LOC100134235 | 116.02 | 6.74 | 103.08 | 6.07 | 10.22 | 0.009 | 0.452 | 1.13 |
| TBX21 | 148.29 | 17.84 | 131.78 | 17.90 | 4.78 | 0.041 | 0.444 | 1.13 |
| ADAM29 | 127.84 | 9.82 | 113.67 | 6.35 | 6.58 | 0.022 | 0.454 | 1.12 |
| DYNC2H1 | 143.98 | 14.72 | 128.03 | 8.60 | 4.33 | 0.049 | 0.452 | 1.12 |
| LOC649431 | 122.90 | 6.85 | 109.30 | 9.79 | 7.13 | 0.019 | 0.449 | 1.12 |
| MID1 | 122.44 | 10.36 | 108.90 | 5.66 | 4.90 | 0.039 | 0.444 | 1.12 |
| CEP250 | 130.37 | 6.67 | 115.99 | 6.94 | 27.17 | 0.001 | 0.450 | 1.12 |
| CDKAL1 | 157.89 | 7.66 | 140.52 | 4.66 | 9.14 | 0.012 | 0.457 | 1.12 |
| CHORDC1 | 140.97 | 3.47 | 125.52 | 0.42 | 6.92 | 0.020 | 0.446 | 1.12 |
| KIAA1967 | 777.42 | 293.39 | 692.39 | 279.29 | 10.00 | 0.010 | 0.454 | 1.12 |
| RSPO4 | 107.77 | 5.99 | 95.98 | 3.52 | 8.20 | 0.015 | 0.453 | 1.12 |
| RHOXF2B | 115.51 | 6.34 | 102.89 | 6.12 | 9.37 | 0.011 | 0.462 | 1.12 |
| C20ORF201 | 106.76 | 6.61 | 95.15 | 4.62 | 9.07 | 0.012 | 0.457 | 1.12 |
| PRND | 105.98 | 0.95 | 94.48 | 1.16 | 9.81 | 0.010 | 0.457 | 1.12 |
| RAD51L1 | 122.45 | 10.65 | 109.24 | 14.30 | 5.33 | 0.033 | 0.446 | 1.12 |
| C15ORF24 | 1978.68 | 550.97 | 1766.28 | 568.81 | 10.21 | 0.009 | 0.452 | 1.12 |
| ZNF330 | 340.23 | 110.85 | 303.74 | 107.14 | 16.01 | 0.004 | 0.455 | 1.12 |
| DAPL1 | 111.60 | 2.35 | 99.67 | 1.98 | 5.06 | 0.037 | 0.446 | 1.12 |
| CACNB2 | 119.26 | 2.39 | 106.52 | 4.04 | 5.51 | 0.031 | 0.446 | 1.12 |
| RFESD | 143.16 | 10.97 | 127.96 | 8.68 | 8.11 | 0.015 | 0.452 | 1.12 |
| LRRFIP2 | 107.41 | 3.00 | 96.00 | 3.33 | 11.90 | 0.007 | 0.444 | 1.12 |
| TBCCD1 | 111.48 | 9.04 | 99.69 | 6.00 | 4.93 | 0.039 | 0.446 | 1.12 |
| LOC100128302 | 114.50 | 2.53 | 102.40 | 5.48 | 5.17 | 0.035 | 0.447 | 1.12 |
| TMUB1 | 586.20 | 164.18 | 524.34 | 182.59 | 5.77 | 0.029 | 0.444 | 1.12 |
| FBXO27 | 108.69 | 4.39 | 97.26 | 4.33 | 4.49 | 0.046 | 0.449 | 1.12 |
| SGPP2 | 124.85 | 3.93 | 111.74 | 6.11 | 6.01 | 0.027 | 0.448 | 1.12 |
| LCE1D | 108.07 | 3.85 | 96.73 | 2.90 | 13.78 | 0.005 | 0.449 | 1.12 |
| NOVA2 | 114.13 | 10.35 | 102.16 | 8.35 | 4.97 | 0.038 | 0.446 | 1.12 |
| CHRND | 103.02 | 2.77 | 92.22 | 1.94 | 5.51 | 0.031 | 0.446 | 1.12 |
| CCDC110 | 109.31 | 9.83 | 97.89 | 7.77 | 4.40 | 0.048 | 0.450 | 1.12 |
| FAM83H | 128.00 | 9.31 | 114.65 | 9.96 | 32.77 | 0.001 | 0.494 | 1.12 |
| AQP12A | 116.63 | 4.33 | 104.47 | 8.34 | 5.18 | 0.035 | 0.447 | 1.12 |
| ANK2 | 112.30 | 3.49 | 100.60 | 4.16 | 11.86 | 0.007 | 0.445 | 1.12 |
| LOC348926 | 115.21 | 7.97 | 103.21 | 5.97 | 6.10 | 0.026 | 0.450 | 1.12 |
| SYNE1 | 97.43 | 2.41 | 87.31 | 3.19 | 18.54 | 0.003 | 0.452 | 1.12 |
| PCDHB16 | 118.81 | 3.20 | 106.49 | 4.66 | 5.01 | 0.038 | 0.446 | 1.12 |
| CLCA3 | 103.70 | 7.20 | 92.95 | 4.96 | 5.09 | 0.036 | 0.446 | 1.12 |
| EGFL6 | 111.49 | 4.38 | 99.96 | 5.33 | 8.38 | 0.014 | 0.452 | 1.12 |
| LOC285804 | 123.21 | 7.06 | 110.56 | 5.73 | 4.55 | 0.045 | 0.448 | 1.11 |
| GUK1 | 1407.43 | 495.23 | 1262.89 | 445.61 | 4.55 | 0.045 | 0.448 | 1.11 |
| LOC646050 | 111.24 | 9.65 | 99.87 | 7.10 | 5.00 | 0.038 | 0.447 | 1.11 |
| CNTROB | 136.86 | 9.09 | 122.89 | 5.72 | 5.99 | 0.027 | 0.448 | 1.11 |
| DIDO1 | 112.45 | 6.78 | 100.99 | 3.29 | 5.56 | 0.031 | 0.445 | 1.11 |
| ZNF300 | 106.99 | 7.49 | 96.08 | 3.75 | 4.51 | 0.046 | 0.449 | 1.11 |
| AKAP11 | 823.13 | 282.87 | 739.36 | 266.84 | 5.35 | 0.033 | 0.446 | 1.11 |
| STAR | 98.82 | 3.71 | 88.79 | 4.17 | 10.76 | 0.009 | 0.454 | 1.11 |
| LOC100131071 | 121.77 | 11.07 | 109.42 | 11.73 | 17.48 | 0.003 | 0.451 | 1.11 |
| KIAA1797 | 191.30 | 37.98 | 171.92 | 40.94 | 6.40 | 0.024 | 0.453 | 1.11 |
| LOC389493 | 114.40 | 1.30 | 102.85 | 4.36 | 6.51 | 0.023 | 0.452 | 1.11 |
| LOC158376 | 124.14 | 12.28 | 111.63 | 12.15 | 14.39 | 0.005 | 0.445 | 1.11 |
| PTGES2 | 213.61 | 6.63 | 192.10 | 8.58 | 6.30 | 0.024 | 0.449 | 1.11 |
| CPT2 | 372.47 | 114.52 | 335.10 | 104.49 | 6.08 | 0.026 | 0.449 | 1.11 |
| ALDH4A1 | 139.42 | 6.81 | 125.57 | 3.63 | 7.05 | 0.020 | 0.449 | 1.11 |
| LOC401286 | 119.45 | 10.98 | 107.63 | 7.69 | 5.58 | 0.031 | 0.445 | 1.11 |
| AAK1 | 111.53 | 3.55 | 100.49 | 5.29 | 7.67 | 0.017 | 0.448 | 1.11 |
| PPIL4 | 110.53 | 4.50 | 99.62 | 2.69 | 5.70 | 0.029 | 0.445 | 1.11 |
| CHN1 | 106.03 | 2.87 | 95.57 | 4.99 | 4.52 | 0.046 | 0.449 | 1.11 |
| MAPK1 | 305.33 | 50.09 | 275.22 | 50.64 | 5.78 | 0.029 | 0.444 | 1.11 |
| LOC644779 | 113.20 | 1.37 | 102.05 | 5.10 | 4.73 | 0.042 | 0.445 | 1.11 |
| CDK5RAP1 | 114.85 | 9.19 | 103.56 | 7.39 | 6.20 | 0.025 | 0.449 | 1.11 |
| ARSI | 99.69 | 2.73 | 89.95 | 4.02 | 7.93 | 0.016 | 0.452 | 1.11 |
| CDC25A | 143.08 | 9.96 | 129.10 | 5.81 | 4.48 | 0.046 | 0.449 | 1.11 |
| AKR1A1 | 2637.78 | 718.84 | 2380.76 | 820.56 | 4.32 | 0.050 | 0.452 | 1.11 |
| RIPK4 | 132.63 | 2.76 | 119.77 | 5.11 | 8.69 | 0.013 | 0.452 | 1.11 |
| LOC100129552 | 206.89 | 12.96 | 186.83 | 18.34 | 4.49 | 0.046 | 0.449 | 1.11 |
| SEC11B | 111.69 | 7.55 | 100.86 | 10.52 | 6.28 | 0.024 | 0.449 | 1.11 |
| TRIM35 | 138.71 | 10.20 | 125.35 | 6.43 | 5.30 | 0.034 | 0.447 | 1.11 |
| MPDU1 | 265.04 | 27.72 | 239.52 | 32.39 | 8.76 | 0.013 | 0.456 | 1.11 |
| MNS1 | 119.03 | 5.40 | 107.58 | 5.22 | 7.03 | 0.020 | 0.449 | 1.11 |
| C8ORF80 | 122.61 | 11.92 | 110.86 | 9.58 | 7.75 | 0.016 | 0.449 | 1.11 |
| LOC651007 | 113.86 | 10.06 | 102.98 | 6.56 | 5.26 | 0.034 | 0.448 | 1.11 |
| KRT16 | 108.93 | 4.08 | 98.57 | 5.91 | 4.77 | 0.041 | 0.444 | 1.11 |
| HOXB6 | 130.56 | 3.97 | 118.14 | 3.34 | 10.07 | 0.010 | 0.455 | 1.11 |
| RAB9B | 129.01 | 4.75 | 116.75 | 6.24 | 6.19 | 0.025 | 0.449 | 1.10 |
| LOC441179 | 109.65 | 5.06 | 99.24 | 4.76 | 4.61 | 0.044 | 0.448 | 1.10 |
| LOC100130581 | 114.45 | 5.89 | 103.59 | 2.42 | 4.92 | 0.039 | 0.445 | 1.10 |
| C1ORF53 | 137.15 | 18.02 | 124.15 | 15.61 | 4.89 | 0.039 | 0.444 | 1.10 |
| HS.242717 | 122.24 | 10.32 | 110.66 | 8.95 | 5.67 | 0.030 | 0.446 | 1.10 |
| C14ORF56 | 105.93 | 4.54 | 95.91 | 4.62 | 6.33 | 0.024 | 0.452 | 1.10 |
| LOC647060 | 125.15 | 5.80 | 113.33 | 8.40 | 7.55 | 0.017 | 0.446 | 1.10 |
| MPG | 132.42 | 5.53 | 119.93 | 7.73 | 6.26 | 0.025 | 0.449 | 1.10 |
| HS.541315 | 138.80 | 7.51 | 125.71 | 2.96 | 4.86 | 0.040 | 0.445 | 1.10 |
| RAB11FIP3 | 332.71 | 98.70 | 301.45 | 99.11 | 10.42 | 0.009 | 0.454 | 1.10 |
| LOC100128689 | 127.50 | 6.14 | 115.53 | 4.49 | 6.40 | 0.024 | 0.453 | 1.10 |
| ZSCAN5A | 202.76 | 41.51 | 183.80 | 47.95 | 4.37 | 0.049 | 0.451 | 1.10 |
| LOC652523 | 115.84 | 8.89 | 105.06 | 9.71 | 22.64 | 0.002 | 0.453 | 1.10 |
| SNX20 | 117.34 | 6.94 | 106.42 | 7.29 | 9.87 | 0.010 | 0.458 | 1.10 |
| LOC100133459 | 150.72 | 19.40 | 136.73 | 19.02 | 6.37 | 0.024 | 0.452 | 1.10 |
| BBS7 | 104.74 | 6.72 | 95.02 | 3.70 | 4.81 | 0.041 | 0.445 | 1.10 |
| LY6H | 111.92 | 2.06 | 101.56 | 5.52 | 4.62 | 0.044 | 0.447 | 1.10 |
| EMID1 | 106.57 | 7.56 | 96.73 | 3.86 | 4.33 | 0.049 | 0.452 | 1.10 |
| LRRC14B | 105.44 | 6.30 | 95.73 | 3.20 | 5.22 | 0.035 | 0.447 | 1.10 |
| MYBL2 | 125.90 | 12.30 | 114.32 | 9.22 | 6.10 | 0.026 | 0.450 | 1.10 |
| DMRTB1 | 111.73 | 5.26 | 101.46 | 6.84 | 4.43 | 0.047 | 0.450 | 1.10 |
| CSNK2A1P | 171.16 | 21.98 | 155.47 | 23.78 | 4.34 | 0.049 | 0.452 | 1.10 |
| FASLG | 134.18 | 14.93 | 121.90 | 14.43 | 12.29 | 0.007 | 0.440 | 1.10 |
| LOC650851 | 118.59 | 12.11 | 107.75 | 10.87 | 6.88 | 0.020 | 0.446 | 1.10 |
| LOC647910 | 124.27 | 10.59 | 112.93 | 8.31 | 8.50 | 0.014 | 0.452 | 1.10 |
| HPS4 | 99.50 | 4.20 | 90.42 | 2.20 | 7.83 | 0.016 | 0.449 | 1.10 |
| MAP2K1 | 1042.41 | 437.12 | 947.44 | 400.72 | 4.46 | 0.047 | 0.449 | 1.10 |
| LOC729467 | 112.54 | 3.06 | 102.29 | 1.97 | 16.02 | 0.004 | 0.456 | 1.10 |
| CEP290 | 178.88 | 43.61 | 162.60 | 42.55 | 26.47 | 0.001 | 0.452 | 1.10 |
| SPINK4 | 101.09 | 4.98 | 91.91 | 2.05 | 4.49 | 0.046 | 0.448 | 1.10 |
| LOC100129222 | 106.56 | 3.64 | 96.89 | 4.79 | 4.84 | 0.040 | 0.445 | 1.10 |
| LOC729397 | 119.13 | 9.64 | 108.35 | 6.86 | 6.64 | 0.022 | 0.453 | 1.10 |
| LOC653489 | 118.23 | 10.40 | 107.55 | 14.24 | 4.69 | 0.043 | 0.446 | 1.10 |
| LOC650846 | 111.55 | 4.08 | 101.47 | 1.46 | 4.53 | 0.045 | 0.449 | 1.10 |
| WRN | 123.62 | 13.02 | 112.52 | 11.13 | 6.34 | 0.024 | 0.451 | 1.10 |
| LOC647881 | 110.75 | 4.19 | 100.82 | 7.64 | 4.97 | 0.038 | 0.446 | 1.10 |
| LOC644725 | 116.02 | 9.60 | 105.65 | 10.75 | 9.07 | 0.012 | 0.456 | 1.10 |
| PCDHAC1 | 115.83 | 12.99 | 105.50 | 13.34 | 43.49 | 0.001 | 0.640 | 1.10 |
| TRAF2 | 177.17 | 18.27 | 161.40 | 15.38 | 8.01 | 0.015 | 0.451 | 1.10 |
| LRRC34 | 118.52 | 4.00 | 108.01 | 1.28 | 4.74 | 0.042 | 0.445 | 1.10 |
| LOC100129876 | 106.06 | 4.36 | 96.69 | 4.64 | 8.42 | 0.014 | 0.451 | 1.10 |
| C6ORF25 | 115.21 | 9.72 | 105.04 | 7.80 | 5.50 | 0.032 | 0.446 | 1.10 |
| ARL14 | 105.21 | 4.45 | 95.94 | 5.36 | 7.73 | 0.016 | 0.449 | 1.10 |
| AGPAT1 | 191.53 | 10.32 | 174.69 | 6.83 | 4.56 | 0.045 | 0.448 | 1.10 |
| REPS2 | 106.15 | 2.51 | 96.85 | 1.00 | 5.63 | 0.030 | 0.446 | 1.10 |
| LOC645262 | 117.41 | 6.39 | 107.14 | 6.78 | 8.59 | 0.013 | 0.450 | 1.10 |
| BAI1 | 106.05 | 8.61 | 96.77 | 8.87 | 7.59 | 0.017 | 0.445 | 1.10 |
| POTEB | 103.84 | 4.83 | 94.82 | 7.99 | 4.94 | 0.039 | 0.445 | 1.10 |
| VEGFB | 304.53 | 44.34 | 278.07 | 44.45 | 14.71 | 0.005 | 0.448 | 1.10 |
| OR4K2 | 101.42 | 5.61 | 92.63 | 6.14 | 5.67 | 0.030 | 0.446 | 1.09 |
| LOC643121 | 124.31 | 10.81 | 113.59 | 12.85 | 6.97 | 0.020 | 0.447 | 1.09 |
| PAX5 | 106.18 | 2.48 | 97.03 | 2.06 | 12.95 | 0.006 | 0.441 | 1.09 |
| ALKBH4 | 136.76 | 13.54 | 124.98 | 11.89 | 4.74 | 0.042 | 0.445 | 1.09 |
| MFSD2B | 109.68 | 2.11 | 100.25 | 1.21 | 4.94 | 0.039 | 0.446 | 1.09 |
| SLCO1A2 | 99.51 | 5.03 | 90.96 | 5.74 | 11.13 | 0.008 | 0.456 | 1.09 |
| C16ORF45 | 125.94 | 9.24 | 115.15 | 8.71 | 15.61 | 0.004 | 0.452 | 1.09 |
| HISPPD1 | 105.77 | 3.85 | 96.72 | 4.55 | 5.72 | 0.029 | 0.445 | 1.09 |
| OGFOD2 | 108.93 | 5.64 | 99.61 | 4.14 | 4.38 | 0.048 | 0.451 | 1.09 |
| QSOX1 | 113.69 | 6.74 | 103.97 | 6.53 | 7.33 | 0.018 | 0.450 | 1.09 |
| EXOG | 128.96 | 9.33 | 117.95 | 7.77 | 6.94 | 0.020 | 0.447 | 1.09 |
| LPPR5 | 102.47 | 0.51 | 93.74 | 3.23 | 4.65 | 0.043 | 0.447 | 1.09 |
| DCLK2 | 103.04 | 2.52 | 94.26 | 5.62 | 4.40 | 0.048 | 0.450 | 1.09 |
| LOC647055 | 135.04 | 6.02 | 123.54 | 2.19 | 5.19 | 0.035 | 0.447 | 1.09 |
| NRF1 | 105.92 | 2.48 | 96.93 | 1.74 | 15.05 | 0.004 | 0.450 | 1.09 |
| C1ORF182 | 121.98 | 10.64 | 111.66 | 9.81 | 5.02 | 0.037 | 0.447 | 1.09 |
| RER1 | 234.73 | 55.96 | 214.88 | 53.83 | 4.44 | 0.047 | 0.450 | 1.09 |
| LOC100128516 | 126.30 | 6.71 | 115.63 | 7.76 | 7.01 | 0.020 | 0.449 | 1.09 |
| LOC646786 | 291.06 | 94.21 | 266.52 | 101.23 | 4.38 | 0.048 | 0.451 | 1.09 |
| PDE6D | 404.51 | 124.54 | 370.48 | 119.94 | 12.75 | 0.006 | 0.440 | 1.09 |
| STXBP1 | 100.33 | 4.56 | 91.90 | 2.32 | 5.31 | 0.034 | 0.447 | 1.09 |
| JMJD2B | 125.45 | 9.48 | 114.92 | 11.31 | 9.80 | 0.010 | 0.456 | 1.09 |
| PCSK1 | 99.74 | 3.51 | 91.37 | 5.01 | 8.54 | 0.013 | 0.452 | 1.09 |
| FAM90A20 | 115.58 | 12.20 | 105.89 | 8.79 | 4.61 | 0.044 | 0.447 | 1.09 |
| PSMD9 | 147.64 | 6.87 | 135.30 | 9.85 | 5.04 | 0.037 | 0.447 | 1.09 |
| LOC647513 | 114.42 | 6.19 | 104.88 | 7.94 | 9.41 | 0.011 | 0.463 | 1.09 |
| RFESD | 123.53 | 4.25 | 113.24 | 3.34 | 5.63 | 0.030 | 0.446 | 1.09 |
| PRIM2 | 134.73 | 4.03 | 123.52 | 4.58 | 6.67 | 0.022 | 0.452 | 1.09 |
| GPR63 | 109.30 | 1.32 | 100.22 | 0.46 | 14.91 | 0.004 | 0.451 | 1.09 |
| NRGN | 154.37 | 16.19 | 141.55 | 13.11 | 6.71 | 0.021 | 0.450 | 1.09 |
| MIR761 | 121.76 | 4.89 | 111.68 | 3.66 | 10.04 | 0.010 | 0.453 | 1.09 |
| KIF27 | 114.23 | 8.27 | 104.85 | 5.49 | 4.33 | 0.049 | 0.452 | 1.09 |
| LOC643558 | 118.69 | 8.46 | 108.96 | 4.84 | 4.50 | 0.046 | 0.449 | 1.09 |
| C20ORF12 | 122.11 | 9.82 | 112.11 | 7.00 | 4.65 | 0.043 | 0.447 | 1.09 |
| APBA2BP | 113.98 | 6.92 | 104.65 | 5.48 | 4.38 | 0.048 | 0.451 | 1.09 |
| NR1I3 | 105.45 | 4.66 | 96.82 | 7.30 | 4.39 | 0.048 | 0.451 | 1.09 |
| DGKK | 107.25 | 5.74 | 98.48 | 5.67 | 6.92 | 0.020 | 0.446 | 1.09 |
| GABRG3 | 109.68 | 4.58 | 100.71 | 3.36 | 11.49 | 0.007 | 0.451 | 1.09 |
| NEK10 | 105.85 | 8.64 | 97.20 | 8.27 | 11.56 | 0.007 | 0.448 | 1.09 |
| LEPR | 129.88 | 7.38 | 119.28 | 8.19 | 7.21 | 0.019 | 0.450 | 1.09 |
| LOC100130141 | 110.91 | 2.49 | 101.88 | 1.91 | 14.97 | 0.004 | 0.452 | 1.09 |
| PABPC5 | 102.37 | 1.34 | 94.05 | 3.30 | 7.14 | 0.019 | 0.450 | 1.09 |
| KCTD6 | 230.17 | 39.49 | 211.53 | 41.64 | 9.05 | 0.012 | 0.455 | 1.09 |
| ATP1B3 | 112.67 | 2.91 | 103.56 | 4.34 | 11.05 | 0.008 | 0.458 | 1.09 |
| SLC22A2 | 105.54 | 1.61 | 97.06 | 0.87 | 5.91 | 0.027 | 0.448 | 1.09 |
| LOC647407 | 115.50 | 8.88 | 106.24 | 7.70 | 4.31 | 0.050 | 0.452 | 1.09 |
| HIST1H1T | 101.15 | 2.06 | 93.04 | 0.94 | 4.73 | 0.042 | 0.445 | 1.09 |
| XK | 106.28 | 2.80 | 97.77 | 1.21 | 8.87 | 0.012 | 0.456 | 1.09 |
| LOC100132209 | 111.83 | 5.26 | 102.88 | 6.86 | 5.91 | 0.027 | 0.448 | 1.09 |
| KCNJ11 | 102.43 | 3.88 | 94.25 | 1.73 | 5.63 | 0.030 | 0.446 | 1.09 |
| PHKG1 | 110.53 | 3.27 | 101.74 | 3.34 | 7.48 | 0.017 | 0.448 | 1.09 |
| LOC100132944 | 112.81 | 0.68 | 103.88 | 1.91 | 8.89 | 0.012 | 0.456 | 1.09 |
| LOC644621 | 115.43 | 6.37 | 106.29 | 7.66 | 7.40 | 0.018 | 0.448 | 1.09 |
| MCF2 | 111.95 | 9.74 | 103.11 | 8.91 | 8.98 | 0.012 | 0.453 | 1.09 |
| LOC727879 | 108.45 | 4.50 | 99.95 | 5.85 | 4.32 | 0.050 | 0.452 | 1.09 |
| CHTF8 | 108.96 | 5.28 | 100.42 | 5.90 | 4.85 | 0.040 | 0.444 | 1.09 |
| IFNB1 | 103.12 | 2.60 | 95.05 | 4.55 | 4.75 | 0.042 | 0.444 | 1.08 |
| HS.102428 | 119.17 | 4.33 | 109.85 | 5.39 | 14.06 | 0.005 | 0.451 | 1.08 |
| ECE1 | 128.15 | 11.46 | 118.19 | 13.25 | 7.98 | 0.015 | 0.452 | 1.08 |
| ELK4 | 164.04 | 31.86 | 151.30 | 32.61 | 4.77 | 0.041 | 0.444 | 1.08 |
| LOC100134436 | 129.37 | 4.64 | 119.32 | 5.74 | 4.35 | 0.049 | 0.452 | 1.08 |
| C4ORF21 | 103.61 | 3.32 | 95.61 | 5.12 | 5.21 | 0.035 | 0.447 | 1.08 |
| RBMY2FP | 141.06 | 8.35 | 130.21 | 7.18 | 5.21 | 0.035 | 0.447 | 1.08 |
| LOC728635 | 290.16 | 28.24 | 267.88 | 23.67 | 7.16 | 0.019 | 0.451 | 1.08 |
| TJP2 | 130.31 | 5.80 | 120.32 | 7.63 | 7.37 | 0.018 | 0.449 | 1.08 |
| LOC260340 | 111.18 | 2.22 | 102.66 | 1.16 | 5.45 | 0.032 | 0.445 | 1.08 |
| PCDHB5 | 107.34 | 5.80 | 99.13 | 4.45 | 7.01 | 0.020 | 0.449 | 1.08 |
| LOC100130079 | 121.90 | 4.53 | 112.62 | 4.23 | 6.41 | 0.023 | 0.453 | 1.08 |
| CLDN1 | 112.07 | 1.00 | 103.54 | 2.46 | 5.05 | 0.037 | 0.447 | 1.08 |
| DPY19L3 | 117.57 | 3.09 | 108.68 | 4.15 | 14.25 | 0.005 | 0.449 | 1.08 |
| CRYBB2 | 133.99 | 13.02 | 123.90 | 12.03 | 4.53 | 0.045 | 0.449 | 1.08 |
| SF4 | 545.09 | 123.26 | 504.10 | 126.43 | 21.29 | 0.002 | 0.450 | 1.08 |
| LOC653616 | 123.07 | 15.22 | 113.84 | 14.25 | 10.06 | 0.010 | 0.454 | 1.08 |
| LOC100129652 | 108.55 | 4.06 | 100.45 | 3.88 | 7.33 | 0.018 | 0.450 | 1.08 |
| CALM2 | 7957.28 | 2697.41 | 7363.72 | 2642.46 | 18.18 | 0.003 | 0.452 | 1.08 |
| UMOD | 113.79 | 5.60 | 105.31 | 5.27 | 5.37 | 0.033 | 0.445 | 1.08 |
| LOC100133101 | 115.10 | 7.80 | 106.55 | 8.56 | 4.63 | 0.044 | 0.447 | 1.08 |
| HS.536770 | 115.22 | 8.80 | 106.66 | 11.72 | 4.34 | 0.049 | 0.452 | 1.08 |
| NDUFA4L2 | 105.89 | 4.28 | 98.04 | 1.40 | 4.62 | 0.044 | 0.447 | 1.08 |
| DGKZ | 100.26 | 9.62 | 92.86 | 8.67 | 5.82 | 0.028 | 0.445 | 1.08 |
| GDF6 | 103.01 | 3.27 | 95.41 | 6.12 | 4.55 | 0.045 | 0.448 | 1.08 |
| CR1 | 117.81 | 7.20 | 109.13 | 8.90 | 8.63 | 0.013 | 0.451 | 1.08 |
| ZNF16 | 122.91 | 12.41 | 113.88 | 9.92 | 5.52 | 0.031 | 0.445 | 1.08 |
| LOC100127953 | 117.01 | 5.65 | 108.41 | 5.12 | 26.19 | 0.001 | 0.444 | 1.08 |
| LOC100129527 | 112.40 | 6.15 | 104.19 | 7.99 | 5.57 | 0.031 | 0.445 | 1.08 |
| IL28A | 107.00 | 3.57 | 99.18 | 4.01 | 10.43 | 0.009 | 0.455 | 1.08 |
| FLJ44477 | 118.25 | 4.27 | 109.64 | 5.72 | 5.46 | 0.032 | 0.445 | 1.08 |
| LOC645947 | 114.45 | 7.40 | 106.12 | 8.85 | 7.54 | 0.017 | 0.446 | 1.08 |
| ACOX1 | 100.51 | 4.20 | 93.23 | 5.95 | 6.80 | 0.021 | 0.448 | 1.08 |
| MGC57359 | 120.75 | 8.32 | 112.01 | 9.66 | 8.03 | 0.015 | 0.453 | 1.08 |
| FRMPD2 | 101.59 | 0.92 | 94.31 | 3.13 | 5.03 | 0.037 | 0.447 | 1.08 |
| XYLB | 132.89 | 5.06 | 123.39 | 7.27 | 7.42 | 0.018 | 0.449 | 1.08 |
| DGAT2L6 | 103.92 | 2.48 | 96.56 | 4.60 | 4.82 | 0.040 | 0.445 | 1.08 |
| LARP1B | 118.15 | 3.01 | 109.85 | 3.00 | 9.03 | 0.012 | 0.455 | 1.08 |
| HEPH | 113.33 | 10.13 | 105.36 | 9.69 | 16.20 | 0.004 | 0.458 | 1.08 |
| FGF11 | 101.77 | 1.30 | 94.66 | 2.36 | 4.37 | 0.049 | 0.451 | 1.08 |
| BEAN | 111.09 | 11.78 | 103.33 | 10.08 | 7.56 | 0.017 | 0.446 | 1.08 |
| LOC100132255 | 109.67 | 5.28 | 102.04 | 6.17 | 7.03 | 0.020 | 0.449 | 1.07 |
| DDI2 | 108.72 | 2.57 | 101.19 | 3.28 | 5.22 | 0.035 | 0.447 | 1.07 |
| PHF17 | 227.66 | 12.25 | 211.95 | 16.36 | 4.68 | 0.043 | 0.447 | 1.07 |
| LOC644669 | 116.98 | 11.82 | 108.93 | 12.72 | 5.40 | 0.033 | 0.445 | 1.07 |
| MIR1258 | 110.12 | 8.43 | 102.55 | 10.38 | 4.48 | 0.046 | 0.448 | 1.07 |
| LOC642164 | 116.11 | 5.95 | 108.12 | 5.12 | 9.73 | 0.010 | 0.459 | 1.07 |
| NHLH1 | 106.63 | 6.92 | 99.32 | 6.74 | 6.48 | 0.023 | 0.452 | 1.07 |
| LOC100128901 | 109.64 | 7.97 | 102.12 | 7.48 | 5.04 | 0.037 | 0.447 | 1.07 |
| CAPN3 | 99.14 | 1.99 | 92.35 | 2.16 | 7.61 | 0.017 | 0.445 | 1.07 |
| WWTR1 | 106.76 | 1.11 | 99.46 | 0.59 | 8.49 | 0.014 | 0.452 | 1.07 |
| GTF2A1L | 103.29 | 4.83 | 96.27 | 4.92 | 8.59 | 0.013 | 0.451 | 1.07 |
| KRTAP26-1 | 100.90 | 3.01 | 94.06 | 1.61 | 5.75 | 0.029 | 0.445 | 1.07 |
| GGA1 | 117.74 | 5.30 | 109.88 | 5.34 | 16.73 | 0.004 | 0.455 | 1.07 |
| OR5M1 | 108.22 | 0.55 | 101.03 | 2.13 | 5.04 | 0.037 | 0.447 | 1.07 |
| LOC100133091 | 110.30 | 4.44 | 103.04 | 1.95 | 5.05 | 0.037 | 0.447 | 1.07 |
| KIAA1530 | 105.33 | 4.44 | 98.42 | 4.05 | 21.13 | 0.002 | 0.445 | 1.07 |
| ZNF81 | 107.92 | 6.17 | 100.85 | 4.01 | 5.28 | 0.034 | 0.447 | 1.07 |
| ZNF233 | 106.35 | 4.30 | 99.39 | 3.19 | 7.64 | 0.017 | 0.446 | 1.07 |
| LOC645899 | 20732.06 | 5944.49 | 19378.69 | 5856.79 | 26.72 | 0.001 | 0.452 | 1.07 |
| LOC100130236 | 109.24 | 1.92 | 102.12 | 1.35 | 16.05 | 0.004 | 0.456 | 1.07 |
| PCP4 | 100.04 | 6.72 | 93.53 | 4.73 | 5.40 | 0.033 | 0.445 | 1.07 |
| PRPS1L1 | 103.99 | 2.67 | 97.24 | 3.18 | 4.60 | 0.044 | 0.448 | 1.07 |
| LOC100129291 | 110.20 | 1.89 | 103.06 | 2.95 | 4.73 | 0.042 | 0.445 | 1.07 |
| PNPLA4 | 108.87 | 9.44 | 101.82 | 7.25 | 4.65 | 0.043 | 0.448 | 1.07 |
| P4HA1 | 109.96 | 5.01 | 102.84 | 7.28 | 4.98 | 0.038 | 0.447 | 1.07 |
| MYNN | 119.00 | 5.54 | 111.30 | 3.74 | 5.93 | 0.027 | 0.448 | 1.07 |
| BPIL3 | 110.53 | 5.65 | 103.46 | 7.16 | 5.02 | 0.038 | 0.446 | 1.07 |
| SCN3A | 102.57 | 5.77 | 96.02 | 4.26 | 6.88 | 0.020 | 0.446 | 1.07 |
| HS.192506 | 120.93 | 6.16 | 113.22 | 5.19 | 13.02 | 0.006 | 0.444 | 1.07 |
| C8ORFK32 | 106.73 | 2.57 | 99.95 | 3.42 | 4.69 | 0.043 | 0.447 | 1.07 |
| ASCL4 | 98.93 | 2.60 | 92.67 | 1.67 | 10.78 | 0.008 | 0.455 | 1.07 |
| GLIPR1L2 | 117.23 | 11.51 | 109.82 | 13.33 | 6.91 | 0.020 | 0.447 | 1.07 |
| ACSL6 | 103.67 | 5.00 | 97.12 | 4.61 | 15.48 | 0.004 | 0.452 | 1.07 |
| SLC6A10P | 98.43 | 2.63 | 92.22 | 2.39 | 5.65 | 0.030 | 0.446 | 1.07 |
| TMEM208 | 325.22 | 67.04 | 304.76 | 61.21 | 5.94 | 0.027 | 0.447 | 1.07 |
| LOC100129036 | 109.90 | 6.36 | 102.99 | 5.98 | 25.54 | 0.002 | 0.436 | 1.07 |
| LOC100130054 | 108.27 | 9.64 | 101.49 | 8.11 | 5.92 | 0.027 | 0.447 | 1.07 |
| LOC727935 | 127.37 | 10.05 | 119.39 | 11.06 | 13.49 | 0.005 | 0.445 | 1.07 |
| SNTA1 | 190.92 | 16.23 | 178.99 | 14.47 | 10.99 | 0.008 | 0.456 | 1.07 |
| RPL10 | 115.44 | 13.34 | 108.28 | 13.57 | 8.62 | 0.013 | 0.451 | 1.07 |
| LOC100131894 | 116.01 | 9.85 | 108.84 | 9.75 | 5.00 | 0.038 | 0.447 | 1.07 |
| DST | 114.77 | 5.76 | 107.70 | 6.06 | 7.97 | 0.015 | 0.452 | 1.07 |
| OR8B3 | 102.39 | 2.87 | 96.09 | 2.86 | 6.29 | 0.024 | 0.450 | 1.07 |
| LOC100133766 | 111.36 | 3.18 | 104.53 | 2.05 | 8.30 | 0.014 | 0.452 | 1.07 |
| FLJ42102 | 97.32 | 1.35 | 91.38 | 1.29 | 57.12 | 0.000 | 0.762 | 1.07 |
| HS.543052 | 116.28 | 6.14 | 109.19 | 4.70 | 4.70 | 0.042 | 0.447 | 1.06 |
| HEATR2 | 104.43 | 1.41 | 98.07 | 1.71 | 4.89 | 0.039 | 0.444 | 1.06 |
| LOC440992 | 115.91 | 9.01 | 108.85 | 10.17 | 5.14 | 0.036 | 0.447 | 1.06 |
| DPP10 | 117.05 | 13.45 | 109.93 | 14.97 | 7.98 | 0.015 | 0.452 | 1.06 |
| C19ORF63 | 120.26 | 13.97 | 112.95 | 13.64 | 6.24 | 0.025 | 0.449 | 1.06 |
| LOC100134674 | 117.13 | 2.38 | 110.01 | 4.35 | 6.26 | 0.025 | 0.450 | 1.06 |
| LPHN3 | 106.67 | 2.79 | 100.19 | 3.64 | 4.88 | 0.039 | 0.444 | 1.06 |
| NR5A2 | 102.22 | 3.27 | 96.02 | 3.80 | 16.51 | 0.004 | 0.459 | 1.06 |
| SLC26A1 | 101.27 | 9.90 | 95.16 | 9.40 | 5.84 | 0.028 | 0.446 | 1.06 |
| LOC652441 | 114.62 | 13.64 | 107.76 | 12.22 | 8.07 | 0.015 | 0.453 | 1.06 |
| XYLT1 | 105.05 | 1.44 | 98.78 | 2.88 | 6.05 | 0.026 | 0.448 | 1.06 |
| MIR877 | 128.38 | 11.27 | 120.73 | 13.16 | 5.21 | 0.035 | 0.447 | 1.06 |
| LOC100133944 | 107.47 | 0.97 | 101.10 | 3.13 | 4.95 | 0.038 | 0.445 | 1.06 |
| LOC646079 | 115.13 | 3.96 | 108.33 | 4.35 | 10.85 | 0.008 | 0.456 | 1.06 |
| LOC100131003 | 109.93 | 4.52 | 103.44 | 5.28 | 13.99 | 0.005 | 0.448 | 1.06 |
| FLJ44451 | 116.33 | 1.22 | 109.54 | 3.64 | 4.85 | 0.040 | 0.444 | 1.06 |
| NAT5 | 102.01 | 2.59 | 96.18 | 2.34 | 5.64 | 0.030 | 0.447 | 1.06 |
| LOC648987 | 125.69 | 6.20 | 118.50 | 7.85 | 6.35 | 0.024 | 0.452 | 1.06 |
| SNIP | 100.03 | 3.14 | 94.37 | 4.77 | 5.83 | 0.028 | 0.445 | 1.06 |
| OTOP1 | 106.13 | 4.60 | 100.23 | 6.32 | 5.17 | 0.035 | 0.447 | 1.06 |
| STYX | 114.21 | 9.42 | 107.88 | 10.68 | 8.43 | 0.014 | 0.451 | 1.06 |
| HS.578887 | 114.27 | 1.79 | 107.94 | 1.58 | 30.71 | 0.001 | 0.459 | 1.06 |
| FLJ26850 | 108.59 | 3.78 | 102.58 | 2.34 | 5.42 | 0.032 | 0.445 | 1.06 |
| PRSS38 | 97.40 | 5.22 | 92.04 | 4.43 | 5.47 | 0.032 | 0.445 | 1.06 |
| TPD52L3 | 101.31 | 4.70 | 95.75 | 3.63 | 5.22 | 0.035 | 0.447 | 1.06 |
| LOC283767 | 118.46 | 3.66 | 111.96 | 4.37 | 15.24 | 0.004 | 0.448 | 1.06 |
| LOC653539 | 111.42 | 0.81 | 105.31 | 2.69 | 5.62 | 0.030 | 0.445 | 1.06 |
| NEDD4 | 117.14 | 1.93 | 110.76 | 1.14 | 5.22 | 0.035 | 0.448 | 1.06 |
| LOC648390 | 1745.02 | 512.70 | 1650.03 | 506.41 | 22.20 | 0.002 | 0.453 | 1.06 |
| SLC30A5 | 121.67 | 6.42 | 115.06 | 8.43 | 5.38 | 0.033 | 0.445 | 1.06 |
| LOC643640 | 118.71 | 12.75 | 112.30 | 11.22 | 4.51 | 0.046 | 0.448 | 1.06 |
| RASSF5 | 127.05 | 5.22 | 120.24 | 5.69 | 22.06 | 0.002 | 0.450 | 1.06 |
| LOC644006 | 113.48 | 1.75 | 107.43 | 0.65 | 4.36 | 0.049 | 0.451 | 1.06 |
| B3GNT9 | 113.92 | 3.40 | 107.84 | 3.06 | 6.51 | 0.023 | 0.453 | 1.06 |
| RHBDD3 | 155.48 | 19.20 | 147.20 | 17.98 | 6.94 | 0.020 | 0.447 | 1.06 |
| ZNF473 | 105.20 | 7.29 | 99.62 | 8.85 | 5.91 | 0.027 | 0.448 | 1.06 |
| LOC643570 | 122.28 | 7.13 | 115.84 | 7.06 | 30.46 | 0.001 | 0.446 | 1.06 |
| LOC653127 | 114.95 | 7.47 | 108.91 | 7.68 | 13.57 | 0.005 | 0.447 | 1.06 |
| RNU86 | 133.19 | 6.62 | 126.19 | 5.23 | 4.81 | 0.041 | 0.445 | 1.06 |
| ACSL4 | 100.46 | 3.23 | 95.19 | 4.12 | 4.43 | 0.047 | 0.450 | 1.06 |
| C2ORF58 | 112.02 | 6.97 | 106.17 | 5.82 | 6.98 | 0.020 | 0.448 | 1.06 |
| LOC136143 | 269.72 | 36.56 | 255.68 | 39.23 | 8.39 | 0.014 | 0.452 | 1.05 |
| LOC644727 | 106.15 | 5.69 | 100.63 | 6.05 | 15.61 | 0.004 | 0.453 | 1.05 |
| SNAR-I | 113.95 | 2.23 | 108.02 | 0.96 | 7.79 | 0.016 | 0.449 | 1.05 |
| ZNF251 | 136.64 | 10.99 | 129.55 | 9.98 | 4.66 | 0.043 | 0.448 | 1.05 |
| SH3BGRL2 | 125.66 | 16.72 | 119.15 | 14.43 | 4.85 | 0.040 | 0.444 | 1.05 |
| CP | 103.07 | 4.34 | 97.73 | 3.15 | 6.00 | 0.027 | 0.448 | 1.05 |
| LOC652655 | 105.47 | 3.29 | 100.03 | 3.62 | 4.73 | 0.042 | 0.445 | 1.05 |
| SYN1 | 101.12 | 6.94 | 96.01 | 5.41 | 4.34 | 0.049 | 0.452 | 1.05 |
| C11ORF31 | 134.20 | 17.04 | 127.44 | 17.30 | 38.84 | 0.001 | 0.531 | 1.05 |
| LOC100131672 | 128.92 | 4.99 | 122.44 | 6.71 | 4.77 | 0.041 | 0.444 | 1.05 |
| MTMR8 | 100.89 | 5.28 | 95.84 | 4.54 | 5.67 | 0.030 | 0.446 | 1.05 |
| HS.163813 | 124.81 | 7.88 | 118.59 | 9.46 | 4.53 | 0.045 | 0.449 | 1.05 |
| DEFA1 | 105.44 | 1.76 | 100.25 | 1.50 | 8.56 | 0.013 | 0.451 | 1.05 |
| HS.304046 | 120.96 | 6.00 | 115.01 | 4.09 | 4.40 | 0.048 | 0.450 | 1.05 |
| HIST1H3G | 111.13 | 1.66 | 105.72 | 2.94 | 7.14 | 0.019 | 0.450 | 1.05 |
| SLC4A9 | 101.37 | 4.84 | 96.44 | 4.02 | 10.18 | 0.010 | 0.452 | 1.05 |
| CDH19 | 113.99 | 8.58 | 108.49 | 6.64 | 4.91 | 0.039 | 0.444 | 1.05 |
| LOC730272 | 110.97 | 11.64 | 105.65 | 10.33 | 5.32 | 0.034 | 0.447 | 1.05 |
| PA2G4P4 | 104.31 | 4.15 | 99.30 | 4.35 | 11.43 | 0.008 | 0.453 | 1.05 |
| CLGN | 95.72 | 2.73 | 91.16 | 2.54 | 5.73 | 0.029 | 0.445 | 1.05 |
| TMEM90A | 113.08 | 1.22 | 107.69 | 1.34 | 18.34 | 0.003 | 0.456 | 1.05 |
| C7ORF60 | 99.90 | 3.52 | 95.16 | 3.13 | 4.65 | 0.043 | 0.447 | 1.05 |
| HS.582338 | 119.12 | 5.26 | 113.62 | 6.06 | 9.38 | 0.011 | 0.462 | 1.05 |
| PFTK2 | 114.03 | 4.91 | 108.78 | 3.54 | 4.38 | 0.048 | 0.451 | 1.05 |
| DEDD | 415.82 | 116.31 | 396.69 | 114.45 | 5.99 | 0.027 | 0.448 | 1.05 |
| LOC441956 | 117.94 | 6.45 | 112.60 | 6.55 | 7.09 | 0.019 | 0.450 | 1.05 |
| KIAA1524 | 110.65 | 7.61 | 105.66 | 7.09 | 8.76 | 0.013 | 0.457 | 1.05 |
| LOC100131076 | 106.79 | 0.61 | 101.98 | 1.36 | 9.03 | 0.012 | 0.455 | 1.05 |
| MAGEA5 | 119.91 | 5.65 | 114.57 | 7.45 | 4.69 | 0.043 | 0.447 | 1.05 |
| LOC100133205 | 105.96 | 6.15 | 101.27 | 5.11 | 4.73 | 0.042 | 0.445 | 1.05 |
| HS.565838 | 120.33 | 3.29 | 115.02 | 4.00 | 4.94 | 0.039 | 0.445 | 1.05 |
| C20ORF144 | 105.71 | 10.13 | 101.14 | 8.43 | 4.41 | 0.048 | 0.450 | 1.05 |
| MEIS2 | 97.68 | 1.83 | 93.46 | 1.22 | 7.23 | 0.019 | 0.450 | 1.05 |
| LOC728518 | 204.34 | 41.49 | 195.53 | 43.09 | 6.52 | 0.023 | 0.453 | 1.05 |
| RASGEF1A | 103.82 | 1.57 | 99.35 | 1.73 | 10.36 | 0.009 | 0.452 | 1.04 |
| LOC643325 | 117.83 | 7.11 | 112.81 | 7.62 | 12.26 | 0.007 | 0.440 | 1.04 |
| LOC392100 | 111.85 | 4.93 | 107.11 | 6.72 | 4.52 | 0.046 | 0.449 | 1.04 |
| SLCO1A2 | 107.27 | 5.56 | 102.74 | 4.85 | 10.82 | 0.008 | 0.455 | 1.04 |
| MIR302A | 110.14 | 2.02 | 105.50 | 1.21 | 6.18 | 0.025 | 0.449 | 1.04 |
| C12ORF39 | 106.85 | 1.67 | 102.35 | 0.84 | 8.90 | 0.012 | 0.456 | 1.04 |
| CRELD1 | 115.20 | 2.96 | 110.36 | 1.21 | 4.77 | 0.041 | 0.444 | 1.04 |
| GOLGA7B | 100.68 | 4.57 | 96.51 | 5.77 | 5.96 | 0.027 | 0.448 | 1.04 |
| SS18 | 132.33 | 8.89 | 126.94 | 8.02 | 4.48 | 0.046 | 0.448 | 1.04 |
| TBC1D29 | 98.22 | 5.15 | 94.30 | 5.15 | 5.38 | 0.033 | 0.445 | 1.04 |
| MRI1 | 96.54 | 4.72 | 92.71 | 5.08 | 12.58 | 0.006 | 0.441 | 1.04 |
| RGS12 | 113.49 | 6.04 | 109.01 | 5.95 | 4.89 | 0.039 | 0.444 | 1.04 |
| OR52E2 | 112.41 | 6.96 | 107.98 | 8.50 | 4.73 | 0.042 | 0.445 | 1.04 |
| ERCC-00079 | 97.02 | 1.71 | 93.25 | 2.11 | 7.60 | 0.017 | 0.445 | 1.04 |
| PLA2G3 | 106.24 | 4.91 | 102.16 | 4.49 | 8.80 | 0.013 | 0.457 | 1.04 |
| TSC22D4 | 207.31 | 24.84 | 199.36 | 23.53 | 9.52 | 0.011 | 0.463 | 1.04 |
| IQSEC3 | 108.74 | 9.78 | 104.60 | 8.72 | 6.74 | 0.021 | 0.449 | 1.04 |
| GNMT | 101.57 | 6.21 | 97.70 | 6.43 | 25.16 | 0.002 | 0.433 | 1.04 |
| CCDC147 | 125.83 | 2.80 | 121.08 | 4.06 | 4.44 | 0.047 | 0.450 | 1.04 |
| LOC646070 | 106.57 | 8.48 | 102.72 | 9.52 | 6.09 | 0.026 | 0.450 | 1.04 |
| LOC100134322 | 109.80 | 7.43 | 105.97 | 6.53 | 7.26 | 0.018 | 0.451 | 1.04 |
| MYH11 | 95.81 | 0.17 | 92.47 | 0.80 | 5.95 | 0.027 | 0.447 | 1.04 |
| LOC641808 | 111.95 | 2.26 | 108.08 | 1.01 | 4.70 | 0.042 | 0.447 | 1.04 |
| LOC651864 | 106.16 | 12.43 | 102.54 | 12.44 | 18.08 | 0.003 | 0.453 | 1.04 |
| LOC642548 | 120.04 | 4.91 | 115.96 | 4.12 | 5.16 | 0.036 | 0.447 | 1.04 |
| DCK | 119.08 | 8.44 | 115.08 | 7.74 | 6.87 | 0.021 | 0.446 | 1.03 |
| ERCC-00067 | 99.30 | 4.46 | 95.98 | 3.16 | 4.37 | 0.049 | 0.451 | 1.03 |
| CYP27C1 | 122.92 | 7.69 | 118.82 | 7.80 | 15.18 | 0.004 | 0.447 | 1.03 |
| FBXO40 | 94.69 | 4.33 | 91.55 | 4.80 | 4.42 | 0.048 | 0.450 | 1.03 |
| CCRL1 | 105.74 | 5.13 | 102.31 | 5.10 | 5.79 | 0.029 | 0.444 | 1.03 |
| ZNF682 | 126.59 | 4.94 | 122.55 | 3.42 | 4.60 | 0.044 | 0.448 | 1.03 |
| ABCB8 | 109.33 | 8.82 | 105.85 | 8.63 | 4.97 | 0.038 | 0.446 | 1.03 |
| LOC100133957 | 104.69 | 4.30 | 101.42 | 4.13 | 5.74 | 0.029 | 0.445 | 1.03 |
| FAM187B | 118.84 | 10.02 | 115.13 | 10.72 | 8.25 | 0.014 | 0.452 | 1.03 |
| C1ORF218 | 684.57 | 305.65 | 663.27 | 307.84 | 6.10 | 0.026 | 0.450 | 1.03 |
| LOC643018 | 116.86 | 7.39 | 113.25 | 6.97 | 14.71 | 0.005 | 0.450 | 1.03 |
| OR2A9P | 110.76 | 9.47 | 107.36 | 8.65 | 6.23 | 0.025 | 0.449 | 1.03 |
| LOC441073 | 152.47 | 18.10 | 147.79 | 18.60 | 11.93 | 0.007 | 0.445 | 1.03 |
| HS.285193 | 112.91 | 1.16 | 109.60 | 1.81 | 7.80 | 0.016 | 0.449 | 1.03 |
| LOC650037 | 115.73 | 8.98 | 112.36 | 7.82 | 5.03 | 0.037 | 0.446 | 1.03 |
| FEZ1 | 101.39 | 4.34 | 98.46 | 4.32 | 4.90 | 0.039 | 0.444 | 1.03 |
| LOC645084 | 129.13 | 9.25 | 125.40 | 8.00 | 4.86 | 0.040 | 0.445 | 1.03 |
| KIRREL2 | 95.81 | 5.11 | 93.07 | 5.03 | 4.31 | 0.050 | 0.452 | 1.03 |
| HS.542369 | 106.57 | 3.91 | 103.51 | 3.99 | 7.08 | 0.019 | 0.450 | 1.03 |
| RFTN2 | 105.46 | 2.19 | 102.49 | 2.01 | 17.80 | 0.003 | 0.445 | 1.03 |
| SGPP1 | 120.89 | 5.40 | 117.53 | 6.16 | 4.32 | 0.050 | 0.452 | 1.03 |
| MIR646 | 111.32 | 3.22 | 108.27 | 4.27 | 4.88 | 0.039 | 0.444 | 1.03 |
| LOC728212 | 100.18 | 3.68 | 97.46 | 3.61 | 4.60 | 0.044 | 0.448 | 1.03 |
| NHLRC3 | 119.57 | 7.16 | 116.39 | 6.97 | 7.32 | 0.018 | 0.450 | 1.03 |
| LOC286239 | 119.78 | 12.98 | 116.65 | 13.57 | 8.37 | 0.014 | 0.452 | 1.03 |
| C17ORF101 | 173.91 | 2.79 | 169.48 | 2.67 | 4.61 | 0.044 | 0.447 | 1.03 |
| FAM90A10 | 116.04 | 10.64 | 113.79 | 10.93 | 7.67 | 0.017 | 0.448 | 1.02 |
| LOC100130221 | 102.02 | 10.75 | 100.11 | 10.92 | 7.89 | 0.016 | 0.451 | 1.02 |
| VNN1 | 108.19 | 5.37 | 106.43 | 5.87 | 5.61 | 0.030 | 0.445 | 1.02 |
| WNT8B | 100.09 | 3.44 | 100.63 | 3.53 | 4.93 | 0.039 | 0.446 | 0.99 |
| HS.156892 | 126.17 | 12.34 | 127.50 | 12.39 | 25.26 | 0.002 | 0.432 | 0.99 |
| LOC100129335 | 108.02 | 8.92 | 109.41 | 9.47 | 4.32 | 0.050 | 0.452 | 0.99 |
| IDI2 | 112.16 | 11.15 | 113.77 | 10.84 | 6.88 | 0.020 | 0.446 | 0.99 |
| ANXA8 | 98.48 | 3.40 | 100.10 | 3.00 | 7.08 | 0.019 | 0.449 | 0.98 |
| HS.311208 | 109.83 | 6.43 | 111.73 | 6.71 | 6.57 | 0.022 | 0.454 | 0.98 |
| LOC653352 | 122.55 | 10.56 | 124.67 | 10.87 | 5.32 | 0.034 | 0.447 | 0.98 |
| LOC402116 | 109.39 | 2.62 | 111.57 | 2.03 | 5.87 | 0.028 | 0.445 | 0.98 |
| ASB18 | 107.15 | 5.28 | 109.41 | 6.06 | 4.79 | 0.041 | 0.444 | 0.98 |
| IL31 | 97.86 | 5.43 | 99.96 | 5.24 | 16.89 | 0.003 | 0.453 | 0.98 |
| OR7C1 | 95.86 | 4.07 | 97.94 | 3.58 | 4.89 | 0.039 | 0.444 | 0.98 |
| FA2H | 97.63 | 4.13 | 99.80 | 3.94 | 17.07 | 0.003 | 0.454 | 0.98 |
| SLC44A3 | 97.79 | 1.87 | 99.97 | 2.29 | 7.14 | 0.019 | 0.450 | 0.98 |
| TNFSF12-TNFSF13 | 115.70 | 10.16 | 118.31 | 10.69 | 7.42 | 0.018 | 0.449 | 0.98 |
| ACER1 | 102.31 | 4.42 | 104.73 | 4.14 | 10.88 | 0.008 | 0.456 | 0.98 |
| MIR588 | 108.03 | 7.32 | 110.60 | 8.11 | 5.10 | 0.036 | 0.447 | 0.98 |
| TRNP1 | 115.62 | 8.76 | 118.41 | 9.68 | 4.98 | 0.038 | 0.447 | 0.98 |
| HS.542544 | 113.01 | 8.49 | 115.73 | 8.28 | 6.09 | 0.026 | 0.450 | 0.98 |
| ZNF619 | 110.81 | 6.91 | 113.54 | 6.41 | 5.79 | 0.029 | 0.444 | 0.98 |
| RASGEF1C | 100.15 | 5.46 | 102.67 | 5.27 | 13.17 | 0.006 | 0.444 | 0.98 |
| LOC401433 | 108.85 | 6.62 | 111.67 | 7.45 | 4.48 | 0.046 | 0.448 | 0.97 |
| COL4A5 | 93.00 | 3.76 | 95.45 | 4.13 | 6.28 | 0.024 | 0.449 | 0.97 |
| C3ORF24 | 113.79 | 10.30 | 116.81 | 10.00 | 5.57 | 0.031 | 0.444 | 0.97 |
| TMEFF2 | 99.87 | 7.86 | 102.65 | 6.85 | 4.63 | 0.044 | 0.447 | 0.97 |
| LOC100129376 | 103.39 | 7.34 | 106.41 | 6.63 | 4.39 | 0.048 | 0.451 | 0.97 |
| SNX33 | 121.16 | 6.02 | 124.75 | 6.62 | 7.36 | 0.018 | 0.449 | 0.97 |
| C21ORF126 | 145.71 | 9.50 | 150.13 | 11.19 | 4.53 | 0.045 | 0.449 | 0.97 |
| ZYG11A | 110.01 | 8.44 | 113.36 | 9.19 | 5.77 | 0.029 | 0.445 | 0.97 |
| HS.352549 | 132.43 | 12.67 | 136.48 | 13.51 | 8.29 | 0.014 | 0.452 | 0.97 |
| IFT81 | 98.38 | 4.68 | 101.40 | 3.99 | 4.42 | 0.048 | 0.450 | 0.97 |
| ELMO2 | 136.51 | 15.08 | 140.72 | 14.32 | 7.29 | 0.018 | 0.450 | 0.97 |
| SOX30 | 113.83 | 2.23 | 117.34 | 0.93 | 4.37 | 0.049 | 0.451 | 0.97 |
| DFNB59 | 126.02 | 11.62 | 129.94 | 12.87 | 4.68 | 0.043 | 0.447 | 0.97 |
| LOC441070 | 103.56 | 4.46 | 106.81 | 3.81 | 5.89 | 0.028 | 0.446 | 0.97 |
| LOC728667 | 106.61 | 4.98 | 110.08 | 4.96 | 9.44 | 0.011 | 0.464 | 0.97 |
| SCNN1A | 103.39 | 2.29 | 106.81 | 2.71 | 6.40 | 0.024 | 0.454 | 0.97 |
| HS.526831 | 107.56 | 2.17 | 111.13 | 2.72 | 11.21 | 0.008 | 0.456 | 0.97 |
| SIK1 | 140.63 | 17.27 | 145.35 | 16.94 | 6.15 | 0.025 | 0.448 | 0.97 |
| PTCHD3 | 105.19 | 5.06 | 108.77 | 4.25 | 5.67 | 0.030 | 0.446 | 0.97 |
| LDOC1L | 258.82 | 30.58 | 267.79 | 30.89 | 18.01 | 0.003 | 0.448 | 0.97 |
| HS.255242 | 113.82 | 8.77 | 117.77 | 8.00 | 8.76 | 0.013 | 0.457 | 0.97 |
| LOC100132469 | 110.32 | 1.55 | 114.19 | 0.88 | 6.43 | 0.023 | 0.452 | 0.97 |
| LOC731366 | 111.68 | 2.14 | 115.61 | 3.23 | 5.84 | 0.028 | 0.446 | 0.97 |
| LPAL2 | 107.23 | 4.00 | 111.00 | 2.79 | 4.53 | 0.046 | 0.449 | 0.97 |
| UBE2QP2 | 112.07 | 8.80 | 116.05 | 8.98 | 12.95 | 0.006 | 0.441 | 0.97 |
| UGT1A4 | 110.67 | 2.36 | 114.61 | 2.24 | 5.28 | 0.034 | 0.447 | 0.97 |
| LOC652887 | 121.33 | 10.67 | 125.66 | 9.29 | 4.40 | 0.048 | 0.450 | 0.97 |
| OR4C46 | 96.09 | 4.18 | 99.54 | 3.32 | 6.22 | 0.025 | 0.449 | 0.97 |
| RDH16 | 100.29 | 2.29 | 103.92 | 2.96 | 9.30 | 0.011 | 0.460 | 0.97 |
| CCDC144A | 96.87 | 0.97 | 100.41 | 2.17 | 5.04 | 0.037 | 0.447 | 0.96 |
| C11ORF41 | 111.90 | 11.98 | 116.02 | 10.62 | 5.16 | 0.036 | 0.447 | 0.96 |
| UTS2 | 121.49 | 5.11 | 125.99 | 6.67 | 4.34 | 0.049 | 0.452 | 0.96 |
| SNORD114-27 | 105.13 | 4.77 | 109.05 | 4.69 | 4.64 | 0.043 | 0.447 | 0.96 |
| RUFY1 | 242.72 | 42.97 | 251.95 | 46.41 | 4.46 | 0.047 | 0.449 | 0.96 |
| PTER | 98.12 | 4.54 | 101.85 | 3.90 | 4.59 | 0.044 | 0.448 | 0.96 |
| HIPK1 | 96.27 | 3.71 | 99.95 | 4.50 | 5.54 | 0.031 | 0.445 | 0.96 |
| DSC3 | 135.98 | 18.36 | 141.22 | 17.20 | 7.66 | 0.017 | 0.447 | 0.96 |
| LOC100133567 | 110.51 | 2.64 | 114.79 | 1.09 | 4.43 | 0.047 | 0.449 | 0.96 |
| LOC651315 | 106.25 | 13.17 | 110.37 | 12.43 | 7.89 | 0.016 | 0.451 | 0.96 |
| RHCE | 96.10 | 0.69 | 99.83 | 1.99 | 4.87 | 0.040 | 0.445 | 0.96 |
| KDR | 104.39 | 5.35 | 108.48 | 4.37 | 4.38 | 0.048 | 0.451 | 0.96 |
| LOC641515 | 105.30 | 2.82 | 109.44 | 2.57 | 15.20 | 0.004 | 0.447 | 0.96 |
| FLJ20518 | 106.99 | 8.25 | 111.21 | 8.03 | 12.91 | 0.006 | 0.441 | 0.96 |
| RGPD3 | 108.31 | 5.09 | 112.61 | 5.22 | 8.77 | 0.013 | 0.457 | 0.96 |
| MAP3K12 | 135.55 | 8.85 | 140.98 | 8.79 | 9.07 | 0.012 | 0.456 | 0.96 |
| RHOBTB1 | 109.49 | 4.08 | 113.90 | 3.86 | 11.17 | 0.008 | 0.455 | 0.96 |
| KRTAP10-1 | 110.91 | 4.08 | 115.45 | 2.97 | 7.06 | 0.019 | 0.449 | 0.96 |
| MIR296 | 100.48 | 6.37 | 104.61 | 6.00 | 5.17 | 0.035 | 0.447 | 0.96 |
| LOC100131594 | 109.85 | 3.69 | 114.36 | 3.80 | 12.37 | 0.006 | 0.444 | 0.96 |
| AQP2 | 109.95 | 3.28 | 114.48 | 3.76 | 7.95 | 0.015 | 0.453 | 0.96 |
| LOC643750 | 103.80 | 2.07 | 108.13 | 2.78 | 4.91 | 0.039 | 0.445 | 0.96 |
| C8ORF42 | 110.29 | 3.74 | 114.94 | 2.94 | 7.62 | 0.017 | 0.445 | 0.96 |
| LOC729177 | 108.91 | 4.05 | 113.55 | 4.76 | 10.76 | 0.009 | 0.453 | 0.96 |
| C10ORF53 | 107.42 | 7.82 | 112.04 | 8.10 | 4.38 | 0.048 | 0.451 | 0.96 |
| TCF7 | 97.02 | 0.34 | 101.24 | 0.94 | 10.00 | 0.010 | 0.454 | 0.96 |
| SUMO4 | 130.11 | 10.51 | 135.79 | 11.70 | 4.89 | 0.039 | 0.444 | 0.96 |
| SERTAD3 | 108.93 | 0.97 | 113.71 | 1.48 | 5.82 | 0.028 | 0.444 | 0.96 |
| OR6S1 | 106.39 | 8.45 | 111.06 | 9.00 | 5.38 | 0.033 | 0.445 | 0.96 |
| FAM75A3 | 114.23 | 4.24 | 119.26 | 4.40 | 5.61 | 0.030 | 0.445 | 0.96 |
| ARHGEF4 | 93.90 | 3.85 | 98.04 | 2.44 | 4.82 | 0.040 | 0.445 | 0.96 |
| FXYD7 | 103.86 | 3.00 | 108.48 | 3.30 | 26.02 | 0.001 | 0.435 | 0.96 |
| NUPL1 | 100.85 | 5.61 | 105.34 | 4.19 | 5.50 | 0.032 | 0.446 | 0.96 |
| LOC390998 | 109.97 | 0.94 | 114.92 | 2.67 | 4.87 | 0.040 | 0.445 | 0.96 |
| ARG99 | 115.84 | 7.30 | 121.06 | 6.87 | 5.43 | 0.032 | 0.444 | 0.96 |
| LOC728278 | 104.98 | 4.99 | 109.72 | 4.15 | 4.70 | 0.042 | 0.447 | 0.96 |
| LOC642969 | 129.76 | 3.65 | 135.67 | 4.50 | 11.84 | 0.007 | 0.446 | 0.96 |
| OR4K1 | 93.84 | 4.37 | 98.12 | 4.71 | 16.44 | 0.004 | 0.455 | 0.96 |
| HS.570799 | 101.11 | 4.41 | 105.73 | 4.90 | 8.44 | 0.014 | 0.452 | 0.96 |
| LOC642449 | 122.88 | 4.53 | 128.50 | 5.84 | 7.24 | 0.019 | 0.450 | 0.96 |
| DCDC2 | 102.78 | 7.89 | 107.51 | 7.66 | 7.01 | 0.020 | 0.449 | 0.96 |
| DGKD | 107.80 | 9.40 | 112.79 | 9.16 | 13.83 | 0.005 | 0.451 | 0.96 |
| HS.584115 | 110.49 | 9.66 | 115.63 | 8.51 | 6.52 | 0.023 | 0.453 | 0.96 |
| LOC100128374 | 112.65 | 7.24 | 117.89 | 9.15 | 4.35 | 0.049 | 0.452 | 0.96 |
| OR2A2 | 114.48 | 2.41 | 119.81 | 3.41 | 9.21 | 0.012 | 0.456 | 0.96 |
| HSPA12A | 105.33 | 2.81 | 110.28 | 3.55 | 10.96 | 0.008 | 0.454 | 0.96 |
| LOC728119 | 107.62 | 8.60 | 112.71 | 9.43 | 4.74 | 0.042 | 0.445 | 0.95 |
| HS.563767 | 110.18 | 2.82 | 115.41 | 3.21 | 6.67 | 0.022 | 0.452 | 0.95 |
| SLC17A5 | 104.89 | 1.91 | 109.88 | 2.02 | 7.19 | 0.019 | 0.450 | 0.95 |
| LUZP6 | 111.15 | 6.33 | 116.44 | 6.31 | 39.05 | 0.001 | 0.534 | 0.95 |
| MBNL3 | 170.21 | 42.97 | 178.33 | 41.05 | 7.31 | 0.018 | 0.450 | 0.95 |
| DRD3 | 112.59 | 1.06 | 117.97 | 0.73 | 11.70 | 0.007 | 0.450 | 0.95 |
| LOC390378 | 108.98 | 5.73 | 114.19 | 4.21 | 5.18 | 0.035 | 0.447 | 0.95 |
| BPGM | 110.06 | 11.94 | 115.34 | 11.24 | 6.14 | 0.025 | 0.448 | 0.95 |
| HS.529590 | 105.78 | 4.75 | 110.90 | 6.37 | 5.22 | 0.035 | 0.446 | 0.95 |
| ZNF780B | 110.62 | 10.00 | 115.98 | 9.98 | 4.31 | 0.050 | 0.452 | 0.95 |
| CHP2 | 107.87 | 1.86 | 113.15 | 2.54 | 4.65 | 0.043 | 0.447 | 0.95 |
| FRMPD2 | 98.58 | 2.85 | 103.42 | 1.98 | 5.22 | 0.035 | 0.447 | 0.95 |
| LOC732393 | 106.45 | 10.39 | 111.73 | 8.86 | 4.65 | 0.043 | 0.447 | 0.95 |
| WDR87 | 107.17 | 6.55 | 112.54 | 4.67 | 4.79 | 0.041 | 0.444 | 0.95 |
| KLK5 | 96.91 | 3.37 | 101.76 | 2.05 | 6.32 | 0.024 | 0.451 | 0.95 |
| LPPR4 | 94.45 | 5.46 | 99.18 | 4.57 | 5.10 | 0.036 | 0.446 | 0.95 |
| LOC645232 | 109.54 | 9.16 | 115.05 | 7.44 | 5.54 | 0.031 | 0.445 | 0.95 |
| PTK9 | 111.16 | 12.93 | 116.79 | 14.26 | 5.23 | 0.035 | 0.447 | 0.95 |
| PRX | 114.14 | 3.85 | 119.93 | 2.61 | 7.91 | 0.016 | 0.451 | 0.95 |
| LOC653807 | 107.88 | 7.68 | 113.41 | 8.49 | 4.53 | 0.045 | 0.449 | 0.95 |
| KLRC3 | 96.03 | 7.61 | 100.96 | 7.32 | 5.86 | 0.028 | 0.445 | 0.95 |
| SGCZ | 102.33 | 4.20 | 107.59 | 2.53 | 5.01 | 0.038 | 0.447 | 0.95 |
| MMP23B | 120.64 | 3.32 | 126.84 | 4.99 | 4.78 | 0.041 | 0.444 | 0.95 |
| HS.577347 | 110.00 | 8.70 | 115.68 | 9.46 | 10.72 | 0.009 | 0.453 | 0.95 |
| SLC6A14 | 99.86 | 4.68 | 105.06 | 6.11 | 4.81 | 0.041 | 0.444 | 0.95 |
| CART1 | 102.64 | 7.16 | 108.02 | 8.60 | 5.07 | 0.037 | 0.446 | 0.95 |
| MIR125B2 | 134.63 | 8.69 | 141.68 | 10.26 | 4.97 | 0.038 | 0.446 | 0.95 |
| LOC643831 | 116.07 | 4.16 | 122.16 | 5.47 | 6.89 | 0.020 | 0.446 | 0.95 |
| KLK3 | 106.98 | 12.79 | 112.60 | 11.61 | 8.24 | 0.014 | 0.451 | 0.95 |
| USP20 | 108.25 | 6.38 | 113.95 | 7.17 | 10.57 | 0.009 | 0.455 | 0.95 |
| MIRLET7A1 | 103.21 | 8.96 | 108.64 | 7.80 | 7.89 | 0.016 | 0.451 | 0.95 |
| DPYS | 97.75 | 5.88 | 102.90 | 6.50 | 14.18 | 0.005 | 0.448 | 0.95 |
| OSTALPHA | 111.33 | 4.00 | 117.22 | 2.90 | 9.11 | 0.012 | 0.456 | 0.95 |
| HS.577948 | 110.04 | 12.55 | 115.87 | 12.67 | 13.49 | 0.005 | 0.444 | 0.95 |
| LOC645167 | 112.75 | 1.63 | 118.73 | 1.72 | 10.71 | 0.009 | 0.453 | 0.95 |
| HS.129500 | 110.50 | 6.59 | 116.38 | 4.60 | 5.10 | 0.036 | 0.447 | 0.95 |
| TNRC6C | 119.87 | 17.24 | 126.26 | 15.91 | 8.38 | 0.014 | 0.453 | 0.95 |
| UBXN11 | 112.40 | 7.49 | 118.41 | 9.58 | 4.76 | 0.041 | 0.444 | 0.95 |
| ZPLD1 | 108.66 | 5.65 | 114.48 | 7.56 | 5.27 | 0.034 | 0.447 | 0.95 |
| TTLL9 | 98.04 | 1.39 | 103.32 | 3.24 | 4.91 | 0.039 | 0.444 | 0.95 |
| CDKN2C | 98.36 | 5.81 | 103.70 | 5.83 | 16.94 | 0.003 | 0.454 | 0.95 |
| LOC646360 | 116.31 | 6.44 | 122.63 | 8.17 | 4.62 | 0.044 | 0.448 | 0.95 |
| TMPO | 115.63 | 8.17 | 121.91 | 9.38 | 8.63 | 0.013 | 0.451 | 0.95 |
| HS.548635 | 111.20 | 10.01 | 117.27 | 11.16 | 7.25 | 0.018 | 0.450 | 0.95 |
| LOC645914 | 102.02 | 5.80 | 107.60 | 7.00 | 7.92 | 0.016 | 0.451 | 0.95 |
| IFNK | 107.86 | 1.92 | 113.77 | 2.78 | 4.65 | 0.043 | 0.447 | 0.95 |
| LOC652834 | 105.32 | 9.57 | 111.13 | 10.67 | 7.99 | 0.015 | 0.452 | 0.95 |
| LOC390427 | 108.44 | 12.77 | 114.43 | 11.36 | 5.42 | 0.032 | 0.445 | 0.95 |
| PRDM13 | 112.08 | 7.09 | 118.32 | 7.48 | 5.63 | 0.030 | 0.446 | 0.95 |
| TPM1 | 98.59 | 6.72 | 104.08 | 6.74 | 117.15 | 0.000 | 0.689 | 0.95 |
| ZNF138 | 110.82 | 1.59 | 117.00 | 3.54 | 5.49 | 0.032 | 0.446 | 0.95 |
| IL1F5 | 95.75 | 6.45 | 101.10 | 5.89 | 8.17 | 0.015 | 0.451 | 0.95 |
| TNFRSF25 | 106.85 | 5.40 | 112.84 | 4.06 | 7.68 | 0.017 | 0.448 | 0.95 |
| LOC654780 | 100.82 | 4.31 | 106.48 | 3.88 | 5.13 | 0.036 | 0.446 | 0.95 |
| LOC652222 | 110.07 | 9.40 | 116.30 | 9.38 | 7.65 | 0.017 | 0.447 | 0.95 |
| LOC100132652 | 123.48 | 14.18 | 130.50 | 15.89 | 6.02 | 0.026 | 0.448 | 0.95 |
| FAIM | 112.15 | 1.39 | 118.53 | 2.64 | 7.58 | 0.017 | 0.444 | 0.95 |
| UBTD2 | 111.16 | 4.20 | 117.48 | 6.28 | 4.32 | 0.050 | 0.452 | 0.95 |
| DNASE1L1 | 97.92 | 6.02 | 103.50 | 5.82 | 4.37 | 0.049 | 0.451 | 0.95 |
| KIR3DX1 | 110.50 | 1.80 | 116.85 | 4.14 | 4.45 | 0.047 | 0.450 | 0.95 |
| IFLTD1 | 108.07 | 5.40 | 114.28 | 6.61 | 8.14 | 0.015 | 0.451 | 0.95 |
| LOC650781 | 107.87 | 9.75 | 114.07 | 9.36 | 5.97 | 0.027 | 0.447 | 0.95 |
| ERMN | 103.94 | 5.81 | 109.93 | 6.07 | 4.92 | 0.039 | 0.445 | 0.95 |
| TSPAN12 | 113.99 | 16.63 | 120.56 | 17.50 | 8.16 | 0.015 | 0.451 | 0.95 |
| LOC100133469 | 113.01 | 4.69 | 119.53 | 4.97 | 5.53 | 0.031 | 0.444 | 0.95 |
| LOC728493 | 105.03 | 3.52 | 111.11 | 5.25 | 6.07 | 0.026 | 0.448 | 0.95 |
| RNU5D | 112.26 | 6.90 | 118.80 | 4.98 | 5.88 | 0.028 | 0.446 | 0.94 |
| IGFBP1 | 97.85 | 6.91 | 103.56 | 8.19 | 5.66 | 0.030 | 0.446 | 0.94 |
| LOC653652 | 116.84 | 8.05 | 123.66 | 10.19 | 5.11 | 0.036 | 0.447 | 0.94 |
| ZNF331 | 115.68 | 3.44 | 122.44 | 2.49 | 5.41 | 0.033 | 0.445 | 0.94 |
| GJA3 | 110.71 | 6.45 | 117.18 | 7.33 | 11.91 | 0.007 | 0.444 | 0.94 |
| SORCS1 | 100.83 | 3.35 | 106.73 | 3.10 | 9.69 | 0.010 | 0.460 | 0.94 |
| LOC100130814 | 109.93 | 3.33 | 116.37 | 3.26 | 7.88 | 0.016 | 0.450 | 0.94 |
| ANKRD13A | 1795.39 | 918.06 | 1900.91 | 944.03 | 6.46 | 0.023 | 0.453 | 0.94 |
| ZFP57 | 115.67 | 9.32 | 122.50 | 11.83 | 4.44 | 0.047 | 0.450 | 0.94 |
| MARS2 | 121.95 | 3.69 | 129.14 | 5.19 | 4.36 | 0.049 | 0.451 | 0.94 |
| THEM5 | 110.61 | 9.19 | 117.14 | 9.07 | 17.88 | 0.003 | 0.445 | 0.94 |
| ARHGAP6 | 103.54 | 8.47 | 109.66 | 6.92 | 5.76 | 0.029 | 0.444 | 0.94 |
| OR10G4 | 93.61 | 5.17 | 99.15 | 5.37 | 7.47 | 0.017 | 0.448 | 0.94 |
| LOC360030 | 106.75 | 2.91 | 113.08 | 2.99 | 25.01 | 0.002 | 0.431 | 0.94 |
| LOC440550 | 101.16 | 10.09 | 107.16 | 10.48 | 13.05 | 0.006 | 0.445 | 0.94 |
| AGTPBP1 | 117.72 | 15.32 | 124.71 | 15.83 | 9.32 | 0.011 | 0.462 | 0.94 |
| LOC647989 | 107.11 | 1.85 | 113.47 | 2.11 | 14.63 | 0.005 | 0.443 | 0.94 |
| GATA6 | 109.52 | 10.22 | 116.05 | 10.59 | 11.94 | 0.007 | 0.444 | 0.94 |
| LOC100128168 | 102.16 | 8.27 | 108.26 | 5.95 | 4.38 | 0.048 | 0.451 | 0.94 |
| FER1L5 | 113.56 | 7.26 | 120.36 | 6.54 | 6.09 | 0.026 | 0.450 | 0.94 |
| UMODL1 | 103.30 | 3.82 | 109.49 | 4.28 | 5.54 | 0.031 | 0.445 | 0.94 |
| HS.569566 | 113.00 | 12.10 | 119.79 | 14.18 | 5.54 | 0.031 | 0.445 | 0.94 |
| LOC642248 | 107.91 | 5.52 | 114.39 | 4.90 | 7.48 | 0.017 | 0.448 | 0.94 |
| LOC100129122 | 111.55 | 4.40 | 118.29 | 5.13 | 10.68 | 0.009 | 0.454 | 0.94 |
| ZCCHC5 | 109.65 | 5.68 | 116.28 | 4.59 | 8.72 | 0.013 | 0.455 | 0.94 |
| LOC646155 | 109.92 | 12.83 | 116.57 | 13.74 | 6.98 | 0.020 | 0.448 | 0.94 |
| LOC441120 | 118.72 | 5.07 | 125.92 | 5.74 | 5.74 | 0.029 | 0.445 | 0.94 |
| MIR1289-1 | 111.16 | 4.14 | 117.92 | 3.46 | 7.64 | 0.017 | 0.446 | 0.94 |
| LOC651381 | 103.09 | 7.97 | 109.36 | 8.86 | 8.28 | 0.014 | 0.452 | 0.94 |
| LOC100129555 | 117.27 | 1.04 | 124.42 | 2.33 | 4.79 | 0.041 | 0.444 | 0.94 |
| HS.191602 | 123.21 | 2.95 | 130.76 | 2.83 | 7.22 | 0.019 | 0.450 | 0.94 |
| TFF3 | 113.75 | 13.62 | 120.74 | 13.66 | 6.18 | 0.025 | 0.448 | 0.94 |
| RORB | 95.15 | 2.23 | 101.03 | 1.12 | 9.02 | 0.012 | 0.455 | 0.94 |
| RARA | 107.00 | 2.20 | 113.63 | 3.02 | 5.07 | 0.037 | 0.446 | 0.94 |
| LOC100130108 | 106.08 | 1.50 | 112.68 | 2.84 | 6.60 | 0.022 | 0.453 | 0.94 |
| LOC648905 | 110.49 | 6.89 | 117.39 | 6.82 | 27.87 | 0.001 | 0.460 | 0.94 |
| OR52M1 | 106.80 | 9.66 | 113.47 | 10.08 | 4.53 | 0.045 | 0.449 | 0.94 |
| FUT2 | 99.64 | 2.09 | 105.87 | 0.67 | 5.67 | 0.030 | 0.447 | 0.94 |
| ABCA4 | 104.79 | 8.33 | 111.38 | 6.78 | 7.24 | 0.019 | 0.450 | 0.94 |
| C21ORF87 | 98.81 | 5.44 | 105.02 | 7.25 | 5.79 | 0.029 | 0.444 | 0.94 |
| LOC644433 | 110.32 | 10.63 | 117.26 | 9.91 | 4.77 | 0.041 | 0.444 | 0.94 |
| HS.529542 | 112.74 | 10.26 | 119.85 | 8.21 | 5.03 | 0.037 | 0.447 | 0.94 |
| LOC729944 | 104.33 | 3.74 | 110.92 | 5.83 | 5.22 | 0.035 | 0.448 | 0.94 |
| MGC5457 | 121.73 | 5.79 | 129.42 | 6.80 | 6.70 | 0.022 | 0.450 | 0.94 |
| LOC644787 | 106.44 | 1.43 | 113.16 | 3.76 | 4.41 | 0.048 | 0.450 | 0.94 |
| LOC100131500 | 108.23 | 1.52 | 115.09 | 3.29 | 5.65 | 0.030 | 0.446 | 0.94 |
| ZNF396 | 107.68 | 6.17 | 114.50 | 5.01 | 5.56 | 0.031 | 0.445 | 0.94 |
| WFDC9 | 101.31 | 4.75 | 107.76 | 5.92 | 4.52 | 0.046 | 0.449 | 0.94 |
| LOC642980 | 114.61 | 7.33 | 121.93 | 8.10 | 7.07 | 0.019 | 0.449 | 0.94 |
| LOC643981 | 104.84 | 5.37 | 111.54 | 6.08 | 6.96 | 0.020 | 0.448 | 0.94 |
| LOC388946 | 115.01 | 10.12 | 122.36 | 10.92 | 8.35 | 0.014 | 0.452 | 0.94 |
| HS.575479 | 111.06 | 3.78 | 118.19 | 5.14 | 5.90 | 0.028 | 0.447 | 0.94 |
| HS.545586 | 109.53 | 4.56 | 116.57 | 4.44 | 15.90 | 0.004 | 0.453 | 0.94 |
| TTC28 | 108.88 | 12.79 | 115.90 | 12.77 | 27.07 | 0.001 | 0.447 | 0.94 |
| PIAS2 | 160.79 | 19.16 | 171.18 | 18.08 | 16.08 | 0.004 | 0.455 | 0.94 |
| TSC22D1 | 110.96 | 11.94 | 118.14 | 9.61 | 4.93 | 0.039 | 0.445 | 0.94 |
| CACHD1 | 97.00 | 4.86 | 103.29 | 7.28 | 4.50 | 0.046 | 0.449 | 0.94 |
| C16ORF50 | 108.62 | 11.72 | 115.67 | 10.74 | 8.34 | 0.014 | 0.452 | 0.94 |
| MIR450B | 103.54 | 9.24 | 110.29 | 7.10 | 4.66 | 0.043 | 0.448 | 0.94 |
| NAP1L1 | 142.10 | 2.94 | 151.38 | 3.68 | 9.08 | 0.012 | 0.456 | 0.94 |
| C1QTNF8 | 94.25 | 6.07 | 100.42 | 4.25 | 5.44 | 0.032 | 0.445 | 0.94 |
| FLJ40672 | 104.36 | 4.27 | 111.26 | 5.84 | 6.94 | 0.020 | 0.447 | 0.94 |
| WIT-1 | 101.69 | 1.00 | 108.43 | 3.31 | 4.96 | 0.038 | 0.445 | 0.94 |
| LOC653537 | 110.10 | 6.98 | 117.42 | 6.78 | 5.01 | 0.038 | 0.447 | 0.94 |
| LOC100130844 | 104.98 | 2.65 | 111.97 | 1.95 | 17.26 | 0.003 | 0.455 | 0.94 |
| LOC400011 | 107.08 | 8.25 | 114.20 | 5.53 | 4.53 | 0.045 | 0.449 | 0.94 |
| AADAT | 102.19 | 6.59 | 109.00 | 5.68 | 5.59 | 0.031 | 0.445 | 0.94 |
| HS.493016 | 107.28 | 3.66 | 114.44 | 6.18 | 4.35 | 0.049 | 0.452 | 0.94 |
| SNX12 | 122.67 | 11.44 | 130.88 | 13.59 | 6.52 | 0.023 | 0.453 | 0.94 |
| LOC647370 | 117.29 | 7.95 | 125.15 | 9.04 | 4.60 | 0.044 | 0.447 | 0.94 |
| HS.539450 | 112.35 | 11.76 | 119.89 | 12.57 | 13.77 | 0.005 | 0.448 | 0.94 |
| PRAMEF5 | 108.12 | 11.76 | 115.39 | 11.75 | 7.94 | 0.015 | 0.452 | 0.94 |
| LOC442245 | 105.07 | 9.30 | 112.14 | 7.71 | 6.16 | 0.025 | 0.448 | 0.94 |
| LOC649563 | 107.96 | 11.99 | 115.28 | 14.11 | 5.66 | 0.030 | 0.447 | 0.94 |
| LOC389963 | 102.45 | 6.15 | 109.38 | 5.77 | 5.03 | 0.037 | 0.446 | 0.94 |
| LOC389906 | 102.58 | 1.19 | 109.54 | 0.74 | 8.99 | 0.012 | 0.454 | 0.94 |
| KGFLP1 | 93.85 | 4.17 | 100.23 | 4.81 | 4.91 | 0.039 | 0.445 | 0.94 |
| IFFO1 | 101.74 | 5.34 | 108.66 | 6.79 | 6.71 | 0.022 | 0.450 | 0.94 |
| HS.281931 | 111.53 | 8.91 | 119.13 | 7.79 | 6.07 | 0.026 | 0.449 | 0.94 |
| HS.299279 | 116.19 | 5.81 | 124.10 | 3.69 | 6.22 | 0.025 | 0.449 | 0.94 |
| LOC100134560 | 106.53 | 7.29 | 113.79 | 4.60 | 4.66 | 0.043 | 0.448 | 0.94 |
| ACVR1C | 108.09 | 8.32 | 115.47 | 7.34 | 5.43 | 0.032 | 0.445 | 0.94 |
| FGL1 | 103.04 | 7.93 | 110.08 | 7.43 | 7.02 | 0.020 | 0.449 | 0.94 |
| LOC644288 | 100.95 | 5.62 | 107.89 | 6.66 | 6.24 | 0.025 | 0.449 | 0.94 |
| LOC728341 | 101.59 | 7.12 | 108.63 | 7.94 | 4.53 | 0.046 | 0.449 | 0.94 |
| ENAH | 92.89 | 4.13 | 99.33 | 3.59 | 19.36 | 0.003 | 0.458 | 0.94 |
| FGF6 | 98.51 | 4.94 | 105.35 | 3.94 | 4.50 | 0.046 | 0.449 | 0.94 |
| HS.560457 | 121.94 | 7.09 | 130.42 | 6.75 | 5.04 | 0.037 | 0.447 | 0.93 |
| HS.560266 | 119.52 | 12.98 | 127.85 | 12.91 | 4.45 | 0.047 | 0.450 | 0.93 |
| ZKSCAN5 | 113.35 | 6.35 | 121.29 | 5.62 | 9.35 | 0.011 | 0.462 | 0.93 |
| LOC440456 | 114.37 | 17.65 | 122.39 | 18.66 | 4.60 | 0.044 | 0.447 | 0.93 |
| KEL | 103.74 | 5.09 | 111.04 | 7.19 | 4.52 | 0.046 | 0.449 | 0.93 |
| KLC2 | 110.32 | 7.84 | 118.11 | 9.65 | 6.01 | 0.027 | 0.448 | 0.93 |
| MEF2A | 107.98 | 4.96 | 115.60 | 2.30 | 4.95 | 0.038 | 0.445 | 0.93 |
| DEFB114 | 106.29 | 11.67 | 113.80 | 9.42 | 5.46 | 0.032 | 0.445 | 0.93 |
| LOC440080 | 108.65 | 4.02 | 116.34 | 6.33 | 5.72 | 0.029 | 0.446 | 0.93 |
| LOC652022 | 112.66 | 12.42 | 120.64 | 11.87 | 16.92 | 0.003 | 0.453 | 0.93 |
| ST20 | 111.14 | 2.51 | 119.03 | 5.03 | 5.35 | 0.033 | 0.446 | 0.93 |
| SFT2D3 | 111.95 | 0.64 | 119.92 | 2.03 | 5.18 | 0.035 | 0.447 | 0.93 |
| MARVELD2 | 111.72 | 7.71 | 119.67 | 6.10 | 4.64 | 0.043 | 0.447 | 0.93 |
| FAM123A | 111.84 | 7.99 | 119.82 | 9.40 | 9.72 | 0.010 | 0.459 | 0.93 |
| LOC651102 | 106.54 | 2.10 | 114.16 | 4.51 | 5.35 | 0.033 | 0.446 | 0.93 |
| CYP19A1 | 105.75 | 2.86 | 113.31 | 1.66 | 8.02 | 0.015 | 0.452 | 0.93 |
| LOC643824 | 103.63 | 3.80 | 111.04 | 4.53 | 17.70 | 0.003 | 0.447 | 0.93 |
| ZNF580 | 111.43 | 7.40 | 119.42 | 4.56 | 4.40 | 0.048 | 0.450 | 0.93 |
| ACAA1 | 344.35 | 96.03 | 369.06 | 96.37 | 11.41 | 0.008 | 0.453 | 0.93 |
| HS.555595 | 113.72 | 3.96 | 121.88 | 3.44 | 11.61 | 0.007 | 0.450 | 0.93 |
| LOC730351 | 100.82 | 6.23 | 108.07 | 5.18 | 4.80 | 0.041 | 0.444 | 0.93 |
| FBF1 | 103.75 | 6.56 | 111.21 | 7.70 | 7.46 | 0.017 | 0.448 | 0.93 |
| OR6C6 | 105.86 | 6.52 | 113.51 | 8.09 | 5.54 | 0.031 | 0.445 | 0.93 |
| LOC100129700 | 102.83 | 1.12 | 110.27 | 2.07 | 12.39 | 0.006 | 0.443 | 0.93 |
| LOC100130241 | 100.22 | 1.83 | 107.48 | 3.16 | 5.84 | 0.028 | 0.446 | 0.93 |
| OPN1MW | 101.24 | 8.30 | 108.58 | 8.06 | 7.98 | 0.015 | 0.452 | 0.93 |
| SMPDL3A | 117.15 | 4.38 | 125.65 | 2.64 | 4.51 | 0.046 | 0.449 | 0.93 |
| HS.287137 | 110.93 | 8.89 | 118.99 | 5.92 | 4.44 | 0.047 | 0.449 | 0.93 |
| SUOX | 93.61 | 3.62 | 100.44 | 4.53 | 4.57 | 0.045 | 0.448 | 0.93 |
| VCX2 | 117.58 | 8.07 | 126.20 | 7.73 | 7.86 | 0.016 | 0.451 | 0.93 |
| LOC645739 | 109.70 | 7.71 | 117.74 | 9.60 | 4.47 | 0.047 | 0.449 | 0.93 |
| TMEM107 | 147.39 | 5.88 | 158.20 | 8.86 | 6.07 | 0.026 | 0.449 | 0.93 |
| LOC441617 | 104.02 | 5.07 | 111.67 | 6.86 | 7.30 | 0.018 | 0.450 | 0.93 |
| HS.573763 | 117.99 | 7.29 | 126.66 | 9.92 | 4.54 | 0.045 | 0.449 | 0.93 |
| SNORD126 | 100.65 | 4.63 | 108.06 | 3.22 | 5.60 | 0.030 | 0.445 | 0.93 |
| LOC158301 | 108.23 | 0.79 | 116.20 | 1.16 | 7.82 | 0.016 | 0.449 | 0.93 |
| TTTY9B | 90.99 | 4.78 | 97.70 | 4.02 | 6.10 | 0.026 | 0.450 | 0.93 |
| LOC100129203 | 104.47 | 2.94 | 112.18 | 5.46 | 4.82 | 0.041 | 0.445 | 0.93 |
| TBX19 | 129.24 | 14.14 | 138.79 | 14.04 | 5.86 | 0.028 | 0.446 | 0.93 |
| EIF4E1B | 116.35 | 10.96 | 124.95 | 8.92 | 4.89 | 0.039 | 0.444 | 0.93 |
| TTTY13 | 107.11 | 15.30 | 115.03 | 14.84 | 4.69 | 0.043 | 0.447 | 0.93 |
| MGC40069 | 102.32 | 2.99 | 109.90 | 2.63 | 4.45 | 0.047 | 0.449 | 0.93 |
| HS.541600 | 116.90 | 14.63 | 125.57 | 16.68 | 5.32 | 0.034 | 0.447 | 0.93 |
| OVOL2 | 95.54 | 7.21 | 102.62 | 6.33 | 4.53 | 0.045 | 0.449 | 0.93 |
| MIR506 | 103.18 | 5.07 | 110.83 | 5.44 | 7.88 | 0.016 | 0.451 | 0.93 |
| LOC730110 | 116.64 | 4.37 | 125.30 | 4.90 | 6.54 | 0.023 | 0.454 | 0.93 |
| LOC728716 | 102.47 | 5.85 | 110.09 | 6.67 | 14.45 | 0.005 | 0.443 | 0.93 |
| LOC645851 | 110.79 | 1.46 | 119.05 | 4.49 | 4.68 | 0.043 | 0.447 | 0.93 |
| HS.571506 | 112.99 | 10.44 | 121.43 | 13.27 | 5.11 | 0.036 | 0.447 | 0.93 |
| KHDC1L | 109.25 | 3.59 | 117.43 | 6.05 | 5.67 | 0.030 | 0.446 | 0.93 |
| DSCR6 | 102.92 | 7.09 | 110.63 | 7.87 | 11.66 | 0.007 | 0.449 | 0.93 |
| LOC648480 | 113.24 | 3.51 | 121.76 | 4.37 | 7.72 | 0.016 | 0.448 | 0.93 |
| LOC652594 | 114.10 | 7.38 | 122.69 | 5.24 | 6.95 | 0.020 | 0.447 | 0.93 |
| HS.538491 | 112.01 | 2.43 | 120.45 | 2.91 | 6.30 | 0.024 | 0.449 | 0.93 |
| DGAT1 | 181.98 | 25.86 | 195.70 | 30.06 | 5.62 | 0.030 | 0.445 | 0.93 |
| UPK1B | 114.27 | 8.69 | 122.91 | 8.25 | 6.44 | 0.023 | 0.453 | 0.93 |
| SLC13A1 | 118.12 | 0.74 | 127.05 | 0.77 | 11.26 | 0.008 | 0.456 | 0.93 |
| LOC100134816 | 113.09 | 4.66 | 121.64 | 4.82 | 22.42 | 0.002 | 0.451 | 0.93 |
| LOC653479 | 105.27 | 5.10 | 113.23 | 5.38 | 5.05 | 0.037 | 0.447 | 0.93 |
| LOC100128942 | 99.08 | 3.07 | 106.60 | 2.17 | 12.83 | 0.006 | 0.438 | 0.93 |
| LOC730387 | 107.11 | 3.62 | 115.24 | 3.32 | 6.52 | 0.023 | 0.453 | 0.93 |
| LOC651452 | 112.42 | 6.01 | 120.96 | 5.78 | 9.62 | 0.011 | 0.463 | 0.93 |
| TRIM40 | 110.64 | 8.21 | 119.06 | 10.48 | 4.54 | 0.045 | 0.449 | 0.93 |
| DSCR8 | 98.01 | 3.43 | 105.47 | 2.89 | 7.46 | 0.017 | 0.448 | 0.93 |
| GBA3 | 110.86 | 12.57 | 119.31 | 9.98 | 4.91 | 0.039 | 0.444 | 0.93 |
| LOC652905 | 109.54 | 5.83 | 117.89 | 8.37 | 5.00 | 0.038 | 0.447 | 0.93 |
| HS.564574 | 107.15 | 13.69 | 115.34 | 10.95 | 5.09 | 0.037 | 0.447 | 0.93 |
| LOC442711 | 102.27 | 5.83 | 110.09 | 8.56 | 4.33 | 0.049 | 0.452 | 0.93 |
| ESX1 | 95.19 | 1.54 | 102.47 | 1.21 | 4.65 | 0.043 | 0.447 | 0.93 |
| LOC728951 | 102.84 | 4.26 | 110.72 | 4.81 | 8.52 | 0.013 | 0.451 | 0.93 |
| LOC285697 | 109.51 | 2.04 | 117.91 | 1.63 | 11.64 | 0.007 | 0.449 | 0.93 |
| LOC729266 | 106.41 | 10.19 | 114.57 | 11.52 | 5.99 | 0.027 | 0.448 | 0.93 |
| LOC646663 | 107.12 | 6.43 | 115.34 | 7.57 | 7.06 | 0.019 | 0.449 | 0.93 |
| MYH1 | 95.30 | 0.83 | 102.63 | 1.79 | 8.63 | 0.013 | 0.451 | 0.93 |
| HS.561575 | 113.67 | 2.92 | 122.41 | 2.97 | 4.61 | 0.044 | 0.448 | 0.93 |
| LOC645335 | 110.22 | 2.06 | 118.70 | 2.34 | 5.99 | 0.027 | 0.448 | 0.93 |
| LOC285407 | 119.13 | 6.69 | 128.32 | 9.32 | 6.04 | 0.026 | 0.449 | 0.93 |
| LOC100132272 | 102.60 | 5.55 | 110.52 | 6.02 | 27.42 | 0.001 | 0.448 | 0.93 |
| AGBL5 | 128.40 | 10.14 | 138.31 | 9.79 | 9.50 | 0.011 | 0.464 | 0.93 |
| IL1R2 | 103.70 | 4.17 | 111.72 | 6.16 | 5.68 | 0.030 | 0.446 | 0.93 |
| CD79B | 909.50 | 224.37 | 980.02 | 243.13 | 4.83 | 0.040 | 0.445 | 0.93 |
| NT5C1B | 99.92 | 0.36 | 107.68 | 2.62 | 5.67 | 0.030 | 0.446 | 0.93 |
| DMKN | 111.53 | 7.75 | 120.19 | 7.29 | 23.99 | 0.002 | 0.436 | 0.93 |
| TOMM40L | 109.55 | 10.24 | 118.08 | 8.64 | 7.14 | 0.019 | 0.450 | 0.93 |
| ZNF509 | 129.88 | 12.81 | 140.01 | 16.66 | 4.39 | 0.048 | 0.451 | 0.93 |
| LOC645099 | 107.68 | 3.35 | 116.08 | 2.17 | 7.84 | 0.016 | 0.450 | 0.93 |
| HS.126473 | 110.27 | 2.07 | 118.88 | 3.50 | 5.26 | 0.034 | 0.448 | 0.93 |
| BRD4 | 111.46 | 8.13 | 120.18 | 7.10 | 14.68 | 0.005 | 0.445 | 0.93 |
| SPNS2 | 112.67 | 7.86 | 121.50 | 6.60 | 5.44 | 0.032 | 0.445 | 0.93 |
| ZXDC | 129.47 | 17.43 | 139.62 | 16.12 | 5.19 | 0.035 | 0.447 | 0.93 |
| HS.131670 | 113.93 | 8.46 | 122.87 | 7.07 | 6.34 | 0.024 | 0.451 | 0.93 |
| LOC100131514 | 106.73 | 6.11 | 115.16 | 5.01 | 12.62 | 0.006 | 0.440 | 0.93 |
| ZFP2 | 108.07 | 5.08 | 116.63 | 6.77 | 7.78 | 0.016 | 0.448 | 0.93 |
| LOC441481 | 119.96 | 2.44 | 129.47 | 1.20 | 9.12 | 0.012 | 0.457 | 0.93 |
| HS.128848 | 111.75 | 8.62 | 120.62 | 10.71 | 5.41 | 0.033 | 0.445 | 0.93 |
| TXNDC2 | 116.52 | 1.47 | 125.78 | 3.00 | 7.41 | 0.018 | 0.449 | 0.93 |
| FGF2 | 104.39 | 6.76 | 112.69 | 7.97 | 5.53 | 0.031 | 0.445 | 0.93 |
| HS.436323 | 107.58 | 1.47 | 116.13 | 1.59 | 118.19 | 0.000 | 1.128 | 0.93 |
| HFE | 113.68 | 11.22 | 122.73 | 12.66 | 5.34 | 0.033 | 0.446 | 0.93 |
| HS.577453 | 113.57 | 11.61 | 122.61 | 14.98 | 4.63 | 0.044 | 0.447 | 0.93 |
| LOC648998 | 108.01 | 4.34 | 116.62 | 1.07 | 4.56 | 0.045 | 0.448 | 0.93 |
| LOC388279 | 99.38 | 0.69 | 107.31 | 2.73 | 5.25 | 0.034 | 0.447 | 0.93 |
| TGFB1I1 | 110.23 | 8.85 | 119.07 | 11.10 | 4.80 | 0.041 | 0.444 | 0.93 |
| C7ORF4 | 100.03 | 1.71 | 108.07 | 0.86 | 5.87 | 0.028 | 0.446 | 0.93 |
| HS.561418 | 117.69 | 12.42 | 127.14 | 14.41 | 8.20 | 0.015 | 0.453 | 0.93 |
| LOC643070 | 105.73 | 5.44 | 114.23 | 6.44 | 14.29 | 0.005 | 0.447 | 0.93 |
| CLDN8 | 99.92 | 10.56 | 107.95 | 7.84 | 4.32 | 0.050 | 0.452 | 0.93 |
| RCE1 | 118.92 | 2.65 | 128.48 | 5.04 | 6.79 | 0.021 | 0.448 | 0.93 |
| PILRB | 101.83 | 2.60 | 110.03 | 3.70 | 7.30 | 0.018 | 0.450 | 0.93 |
| LOC651777 | 128.38 | 7.28 | 138.72 | 5.90 | 8.86 | 0.013 | 0.456 | 0.93 |
| CRAT | 97.08 | 5.68 | 104.91 | 5.40 | 31.02 | 0.001 | 0.471 | 0.93 |
| BCL2L15 | 100.03 | 6.96 | 108.10 | 6.93 | 14.88 | 0.004 | 0.451 | 0.93 |
| LOC344328 | 124.61 | 5.49 | 134.67 | 5.57 | 5.11 | 0.036 | 0.447 | 0.93 |
| FAM90A8 | 107.55 | 2.58 | 116.25 | 4.07 | 4.40 | 0.048 | 0.450 | 0.93 |
| LOC100133829 | 109.96 | 2.71 | 118.87 | 3.35 | 5.71 | 0.029 | 0.446 | 0.93 |
| LOC100132402 | 105.88 | 2.85 | 114.46 | 2.23 | 11.98 | 0.007 | 0.444 | 0.92 |
| TRIM39 | 97.87 | 7.62 | 105.81 | 7.76 | 4.58 | 0.045 | 0.448 | 0.92 |
| KIN | 99.98 | 3.19 | 108.12 | 0.82 | 5.46 | 0.032 | 0.445 | 0.92 |
| LOC729154 | 112.01 | 11.71 | 121.14 | 10.67 | 11.95 | 0.007 | 0.445 | 0.92 |
| HS.258083 | 103.40 | 1.91 | 111.84 | 2.91 | 4.83 | 0.040 | 0.444 | 0.92 |
| PAIP2 | 129.00 | 11.52 | 139.52 | 11.23 | 28.63 | 0.001 | 0.450 | 0.92 |
| CHRM2 | 112.13 | 0.31 | 121.28 | 3.74 | 4.52 | 0.046 | 0.448 | 0.92 |
| SNORD72 | 101.30 | 4.25 | 109.57 | 5.59 | 5.30 | 0.034 | 0.447 | 0.92 |
| LOC729080 | 101.52 | 4.40 | 109.82 | 2.08 | 5.85 | 0.028 | 0.446 | 0.92 |
| KRTAP4-8 | 111.60 | 3.52 | 120.72 | 6.23 | 5.17 | 0.035 | 0.447 | 0.92 |
| LOC651974 | 106.61 | 8.41 | 115.33 | 10.55 | 4.57 | 0.045 | 0.448 | 0.92 |
| LOC652102 | 108.60 | 5.25 | 117.49 | 5.10 | 22.55 | 0.002 | 0.452 | 0.92 |
| CDKL2 | 113.55 | 6.27 | 122.86 | 7.91 | 9.25 | 0.011 | 0.456 | 0.92 |
| LOC100128322 | 107.22 | 9.99 | 116.01 | 13.15 | 4.81 | 0.041 | 0.444 | 0.92 |
| LOC442406 | 110.43 | 6.58 | 119.49 | 7.88 | 4.76 | 0.041 | 0.444 | 0.92 |
| KLF10 | 115.50 | 4.23 | 124.98 | 3.55 | 21.94 | 0.002 | 0.451 | 0.92 |
| HS.548213 | 131.15 | 1.69 | 141.92 | 4.96 | 5.70 | 0.029 | 0.445 | 0.92 |
| GABPAP | 103.42 | 3.63 | 111.91 | 6.62 | 4.82 | 0.040 | 0.445 | 0.92 |
| KRTAP20-3 | 123.37 | 9.51 | 133.50 | 9.54 | 10.31 | 0.009 | 0.452 | 0.92 |
| LOC645013 | 111.82 | 6.99 | 121.05 | 7.49 | 15.30 | 0.004 | 0.449 | 0.92 |
| TMEM207 | 122.85 | 4.43 | 132.99 | 5.25 | 17.35 | 0.003 | 0.455 | 0.92 |
| HS.552896 | 112.27 | 6.07 | 121.54 | 4.67 | 7.33 | 0.018 | 0.450 | 0.92 |
| LOC100131426 | 108.63 | 2.65 | 117.61 | 5.01 | 5.28 | 0.034 | 0.447 | 0.92 |
| HP | 110.55 | 7.73 | 119.70 | 8.42 | 8.05 | 0.015 | 0.453 | 0.92 |
| LOC652757 | 108.45 | 12.60 | 117.42 | 12.87 | 7.81 | 0.016 | 0.449 | 0.92 |
| DYDC1 | 103.30 | 6.81 | 111.85 | 6.35 | 19.17 | 0.003 | 0.451 | 0.92 |
| LOC641798 | 142.83 | 1.59 | 154.66 | 2.72 | 15.57 | 0.004 | 0.451 | 0.92 |
| HS.567088 | 109.08 | 7.68 | 118.12 | 11.03 | 4.66 | 0.043 | 0.448 | 0.92 |
| POGZ | 107.87 | 1.09 | 116.82 | 1.41 | 6.34 | 0.024 | 0.451 | 0.92 |
| GDF10 | 108.57 | 10.82 | 117.58 | 9.59 | 5.53 | 0.031 | 0.445 | 0.92 |
| IFNW1 | 102.39 | 6.93 | 110.89 | 5.65 | 7.49 | 0.017 | 0.448 | 0.92 |
| MAPK10 | 107.09 | 5.45 | 116.00 | 7.18 | 4.84 | 0.040 | 0.444 | 0.92 |
| CDH24 | 115.29 | 5.39 | 124.88 | 7.47 | 7.61 | 0.017 | 0.445 | 0.92 |
| SAMD4B | 142.73 | 13.71 | 154.63 | 12.96 | 22.67 | 0.002 | 0.454 | 0.92 |
| ZDHHC19 | 118.35 | 13.87 | 128.22 | 16.05 | 7.63 | 0.017 | 0.446 | 0.92 |
| GAFA1 | 106.48 | 6.89 | 115.37 | 5.58 | 5.67 | 0.030 | 0.446 | 0.92 |
| HS.286666 | 110.94 | 5.18 | 120.23 | 2.82 | 4.56 | 0.045 | 0.448 | 0.92 |
| LOC642617 | 104.10 | 3.01 | 112.84 | 5.04 | 4.52 | 0.046 | 0.449 | 0.92 |
| HS.554608 | 116.63 | 23.37 | 126.42 | 21.87 | 6.25 | 0.025 | 0.449 | 0.92 |
| C17ORF86 | 100.92 | 1.39 | 109.42 | 3.00 | 6.98 | 0.020 | 0.447 | 0.92 |
| LOC23117 | 105.95 | 5.18 | 114.87 | 5.52 | 4.93 | 0.039 | 0.445 | 0.92 |
| HS.564658 | 111.92 | 14.82 | 121.37 | 14.76 | 8.40 | 0.014 | 0.452 | 0.92 |
| HS.582055 | 110.79 | 8.14 | 120.15 | 7.42 | 5.82 | 0.028 | 0.444 | 0.92 |
| MIR578 | 106.34 | 7.79 | 115.34 | 5.78 | 6.16 | 0.025 | 0.448 | 0.92 |
| PCDH11X | 107.20 | 9.76 | 116.28 | 10.43 | 4.60 | 0.044 | 0.447 | 0.92 |
| LOC654010 | 98.95 | 6.13 | 107.36 | 7.89 | 8.26 | 0.014 | 0.451 | 0.92 |
| SELK | 106.47 | 5.38 | 115.55 | 4.20 | 5.25 | 0.034 | 0.448 | 0.92 |
| LOC100129053 | 104.17 | 7.96 | 113.07 | 7.71 | 5.06 | 0.037 | 0.447 | 0.92 |
| SPOCK3 | 101.29 | 2.84 | 109.95 | 5.11 | 5.30 | 0.034 | 0.447 | 0.92 |
| FLJ45513 | 115.87 | 11.18 | 125.78 | 10.22 | 7.85 | 0.016 | 0.450 | 0.92 |
| LOC100129159 | 110.10 | 3.24 | 119.54 | 2.36 | 9.48 | 0.011 | 0.465 | 0.92 |
| UGT2A1 | 108.70 | 7.41 | 118.04 | 4.48 | 5.23 | 0.035 | 0.448 | 0.92 |
| LOC100128994 | 137.97 | 11.40 | 149.83 | 8.09 | 4.40 | 0.048 | 0.450 | 0.92 |
| HS.562488 | 112.66 | 12.82 | 122.34 | 13.77 | 14.64 | 0.005 | 0.443 | 0.92 |
| LOC648189 | 118.87 | 11.58 | 129.09 | 15.07 | 4.45 | 0.047 | 0.449 | 0.92 |
| HS.574082 | 108.26 | 14.95 | 117.57 | 12.71 | 7.12 | 0.019 | 0.450 | 0.92 |
| DPP6 | 114.46 | 14.19 | 124.31 | 13.43 | 5.43 | 0.032 | 0.444 | 0.92 |
| MIR487A | 103.42 | 3.43 | 112.33 | 6.41 | 5.13 | 0.036 | 0.447 | 0.92 |
| LOC100131984 | 102.60 | 8.08 | 111.44 | 8.01 | 7.83 | 0.016 | 0.449 | 0.92 |
| LOC442041 | 115.72 | 3.62 | 125.71 | 4.96 | 6.52 | 0.023 | 0.453 | 0.92 |
| LOC727833 | 108.27 | 8.30 | 117.62 | 9.50 | 5.68 | 0.030 | 0.447 | 0.92 |
| LOC641733 | 102.14 | 7.14 | 110.97 | 7.99 | 9.72 | 0.010 | 0.458 | 0.92 |
| LOC641700 | 107.61 | 5.36 | 116.92 | 3.85 | 5.42 | 0.032 | 0.445 | 0.92 |
| LOC100130734 | 101.34 | 1.81 | 110.12 | 4.45 | 4.56 | 0.045 | 0.448 | 0.92 |
| RPS11 | 14951.40 | 5581.17 | 16249.21 | 5305.52 | 4.73 | 0.042 | 0.445 | 0.92 |
| HS.62645 | 117.83 | 12.36 | 128.11 | 9.11 | 5.41 | 0.033 | 0.445 | 0.92 |
| SPTBN2 | 116.12 | 9.68 | 126.26 | 10.31 | 25.16 | 0.002 | 0.431 | 0.92 |
| OSBPL9 | 113.04 | 5.00 | 122.91 | 7.60 | 6.49 | 0.023 | 0.452 | 0.92 |
| C1ORF9 | 112.94 | 9.23 | 122.80 | 10.65 | 9.35 | 0.011 | 0.463 | 0.92 |
| LOC442249 | 102.50 | 3.10 | 111.47 | 0.64 | 4.36 | 0.049 | 0.451 | 0.92 |
| LOC642413 | 109.55 | 4.32 | 119.14 | 4.55 | 4.50 | 0.046 | 0.449 | 0.92 |
| C20ORF91 | 109.41 | 6.82 | 118.99 | 8.14 | 5.03 | 0.037 | 0.447 | 0.92 |
| LOC729897 | 100.22 | 4.21 | 109.00 | 3.02 | 6.61 | 0.022 | 0.454 | 0.92 |
| STX6 | 479.47 | 222.36 | 521.54 | 208.38 | 4.98 | 0.038 | 0.446 | 0.92 |
| PABPC1L2A | 103.50 | 7.76 | 112.59 | 8.52 | 11.59 | 0.007 | 0.448 | 0.92 |
| OR1K1 | 105.88 | 0.90 | 115.18 | 4.50 | 4.37 | 0.049 | 0.451 | 0.92 |
| HS.573792 | 98.65 | 6.67 | 107.35 | 5.30 | 6.69 | 0.022 | 0.451 | 0.92 |
| GABRG2 | 99.87 | 4.95 | 108.69 | 5.81 | 5.79 | 0.029 | 0.444 | 0.92 |
| FLJ42562 | 111.91 | 4.34 | 121.80 | 2.69 | 6.28 | 0.024 | 0.449 | 0.92 |
| LOC123855 | 116.85 | 2.32 | 127.19 | 2.64 | 21.15 | 0.002 | 0.446 | 0.92 |
| C20ORF95 | 106.15 | 2.25 | 115.54 | 0.87 | 5.22 | 0.035 | 0.448 | 0.92 |
| MIR380 | 102.98 | 13.16 | 112.10 | 13.50 | 4.62 | 0.044 | 0.447 | 0.92 |
| HS.555247 | 108.97 | 1.47 | 118.62 | 0.23 | 13.38 | 0.006 | 0.444 | 0.92 |
| NEBL | 98.56 | 3.93 | 107.30 | 2.23 | 6.34 | 0.024 | 0.451 | 0.92 |
| PCDHGA12 | 109.90 | 9.56 | 119.65 | 7.46 | 8.04 | 0.015 | 0.453 | 0.92 |
| CACNA2D2 | 112.54 | 11.76 | 122.52 | 10.75 | 12.42 | 0.006 | 0.444 | 0.92 |
| FLJ34969 | 110.91 | 7.12 | 120.76 | 7.81 | 4.53 | 0.046 | 0.449 | 0.92 |
| C3ORF70 | 103.63 | 4.07 | 112.84 | 3.28 | 18.03 | 0.003 | 0.452 | 0.92 |
| ATP5I | 108.93 | 6.36 | 118.64 | 7.75 | 10.05 | 0.010 | 0.453 | 0.92 |
| LOC400509 | 111.51 | 6.15 | 121.45 | 8.87 | 4.90 | 0.039 | 0.444 | 0.92 |
| LOC648876 | 112.28 | 4.29 | 122.30 | 3.99 | 8.67 | 0.013 | 0.452 | 0.92 |
| ATP5EP2 | 4861.92 | 1817.36 | 5295.95 | 1695.95 | 5.50 | 0.031 | 0.446 | 0.92 |
| LOC642383 | 102.30 | 7.79 | 111.43 | 10.18 | 6.62 | 0.022 | 0.454 | 0.92 |
| WDFY4 | 257.93 | 65.74 | 280.97 | 69.13 | 4.38 | 0.048 | 0.451 | 0.92 |
| LOC650678 | 108.79 | 6.18 | 118.51 | 9.29 | 5.25 | 0.034 | 0.448 | 0.92 |
| PPAP2C | 112.24 | 3.19 | 122.32 | 2.40 | 15.28 | 0.004 | 0.449 | 0.92 |
| LOC100133178 | 111.37 | 1.97 | 121.37 | 4.47 | 6.91 | 0.020 | 0.447 | 0.92 |
| HS.579501 | 107.39 | 7.71 | 117.05 | 6.54 | 13.23 | 0.006 | 0.446 | 0.92 |
| LOC646310 | 106.65 | 4.75 | 116.24 | 5.34 | 12.26 | 0.007 | 0.440 | 0.92 |
| HS.574636 | 114.72 | 1.11 | 125.06 | 0.71 | 12.00 | 0.007 | 0.444 | 0.92 |
| HS.33032 | 121.94 | 6.55 | 132.94 | 7.35 | 14.95 | 0.004 | 0.452 | 0.92 |
| SLC5A1 | 104.85 | 12.59 | 114.32 | 11.05 | 9.41 | 0.011 | 0.463 | 0.92 |
| LOC100133802 | 109.59 | 8.93 | 119.49 | 11.24 | 6.78 | 0.021 | 0.448 | 0.92 |
| MIR7-3 | 101.47 | 3.69 | 110.65 | 3.33 | 11.42 | 0.008 | 0.453 | 0.92 |
| DKFZP686J0529 | 108.73 | 2.37 | 118.58 | 4.77 | 4.97 | 0.038 | 0.446 | 0.92 |
| FAM59B | 116.58 | 11.37 | 127.15 | 8.75 | 4.44 | 0.047 | 0.450 | 0.92 |
| LOC646934 | 99.84 | 5.60 | 108.90 | 6.13 | 8.70 | 0.013 | 0.453 | 0.92 |
| LOC731042 | 101.49 | 5.80 | 110.72 | 9.47 | 4.33 | 0.049 | 0.452 | 0.92 |
| LOC100130616 | 104.36 | 2.66 | 113.85 | 4.86 | 7.21 | 0.019 | 0.450 | 0.92 |
| VPS13B | 104.51 | 6.41 | 114.02 | 3.40 | 4.41 | 0.048 | 0.450 | 0.92 |
| LOC641908 | 110.20 | 11.47 | 120.24 | 10.33 | 14.79 | 0.005 | 0.454 | 0.92 |
| HS.537944 | 108.03 | 6.54 | 117.87 | 7.95 | 11.05 | 0.008 | 0.458 | 0.92 |
| LOC650363 | 112.76 | 5.20 | 123.03 | 7.76 | 5.67 | 0.030 | 0.446 | 0.92 |
| SDCCAG3 | 107.56 | 4.17 | 117.36 | 6.03 | 5.66 | 0.030 | 0.446 | 0.92 |
| LOC100128201 | 96.25 | 3.51 | 105.03 | 2.71 | 9.59 | 0.011 | 0.463 | 0.92 |
| LOC389827 | 107.49 | 2.02 | 117.29 | 3.85 | 9.05 | 0.012 | 0.455 | 0.92 |
| LOC653061 | 112.36 | 11.67 | 122.61 | 14.08 | 6.10 | 0.026 | 0.450 | 0.92 |
| OR3A2 | 107.96 | 3.98 | 117.81 | 4.27 | 8.19 | 0.015 | 0.454 | 0.92 |
| LOC392843 | 108.27 | 13.56 | 118.15 | 16.36 | 5.26 | 0.034 | 0.448 | 0.92 |
| LOC645689 | 103.48 | 2.60 | 112.93 | 1.04 | 5.23 | 0.035 | 0.447 | 0.92 |
| MGC52282 | 112.40 | 8.84 | 122.68 | 9.14 | 4.86 | 0.040 | 0.444 | 0.92 |
| TSHR | 106.07 | 13.19 | 115.77 | 11.16 | 8.02 | 0.015 | 0.452 | 0.92 |
| LOC646044 | 308.46 | 49.37 | 336.70 | 55.58 | 7.67 | 0.017 | 0.448 | 0.92 |
| C21ORF122 | 115.51 | 3.49 | 126.10 | 6.76 | 4.75 | 0.041 | 0.444 | 0.92 |
| OR52J3 | 100.79 | 11.29 | 110.04 | 12.89 | 6.89 | 0.020 | 0.446 | 0.92 |
| LOC647080 | 114.27 | 5.64 | 124.76 | 6.42 | 6.50 | 0.023 | 0.452 | 0.92 |
| LOC647269 | 113.25 | 10.81 | 123.65 | 8.91 | 4.44 | 0.047 | 0.449 | 0.92 |
| NOXA1 | 124.36 | 13.63 | 135.79 | 11.05 | 7.14 | 0.019 | 0.450 | 0.92 |
| SLC16A14 | 93.35 | 4.76 | 101.94 | 6.13 | 7.60 | 0.017 | 0.445 | 0.92 |
| CREB5 | 97.17 | 4.02 | 106.12 | 4.30 | 6.24 | 0.025 | 0.449 | 0.92 |
| KCNIP2 | 114.90 | 8.76 | 125.49 | 10.58 | 5.30 | 0.034 | 0.447 | 0.92 |
| RELT | 111.61 | 5.15 | 121.91 | 5.98 | 8.94 | 0.012 | 0.455 | 0.92 |
| C10ORF55 | 96.87 | 3.34 | 105.81 | 3.70 | 5.71 | 0.029 | 0.445 | 0.92 |
| HS.577935 | 114.61 | 7.97 | 125.19 | 10.70 | 6.06 | 0.026 | 0.448 | 0.92 |
| LOC643145 | 107.83 | 11.55 | 117.79 | 12.76 | 5.07 | 0.037 | 0.446 | 0.92 |
| CTTNBP2NL | 110.76 | 7.11 | 120.99 | 9.68 | 4.53 | 0.045 | 0.449 | 0.92 |
| KIAA1210 | 109.47 | 4.64 | 119.58 | 6.33 | 4.36 | 0.049 | 0.452 | 0.92 |
| LOC401911 | 111.70 | 11.55 | 122.04 | 12.70 | 4.65 | 0.043 | 0.447 | 0.92 |
| LOC374973 | 109.04 | 7.73 | 119.14 | 9.32 | 4.80 | 0.041 | 0.444 | 0.92 |
| HS.560344 | 119.39 | 7.91 | 130.49 | 5.78 | 5.17 | 0.036 | 0.447 | 0.91 |
| FRMPD2L1 | 101.12 | 8.40 | 110.53 | 7.75 | 4.57 | 0.045 | 0.448 | 0.91 |
| PDLIM2 | 91.12 | 1.68 | 99.60 | 1.38 | 28.24 | 0.001 | 0.459 | 0.91 |
| GFRA4 | 101.02 | 2.77 | 110.45 | 2.93 | 10.79 | 0.008 | 0.455 | 0.91 |
| LOC650410 | 107.19 | 9.76 | 117.19 | 8.37 | 12.36 | 0.006 | 0.444 | 0.91 |
| SLCO3A1 | 110.19 | 11.48 | 120.48 | 12.36 | 10.45 | 0.009 | 0.455 | 0.91 |
| LOC641983 | 112.86 | 10.03 | 123.40 | 10.66 | 29.27 | 0.001 | 0.448 | 0.91 |
| BEX1 | 109.05 | 6.17 | 119.25 | 5.41 | 7.50 | 0.017 | 0.447 | 0.91 |
| LOC648674 | 102.45 | 3.74 | 112.03 | 6.54 | 5.64 | 0.030 | 0.446 | 0.91 |
| LOC651610 | 110.11 | 7.89 | 120.40 | 9.86 | 5.25 | 0.034 | 0.447 | 0.91 |
| LOC642244 | 104.32 | 7.26 | 114.08 | 4.30 | 5.64 | 0.030 | 0.446 | 0.91 |
| LOC729581 | 111.30 | 4.39 | 121.71 | 2.99 | 9.28 | 0.011 | 0.460 | 0.91 |
| LOC649425 | 95.98 | 2.44 | 104.97 | 3.16 | 4.53 | 0.045 | 0.449 | 0.91 |
| LOC152578 | 105.84 | 6.90 | 115.76 | 4.34 | 6.70 | 0.022 | 0.450 | 0.91 |
| LOC390806 | 100.76 | 2.95 | 110.23 | 5.47 | 6.40 | 0.024 | 0.453 | 0.91 |
| LOC728969 | 104.22 | 5.23 | 114.02 | 6.06 | 8.93 | 0.012 | 0.455 | 0.91 |
| HS.583362 | 109.16 | 18.01 | 119.42 | 19.51 | 4.73 | 0.042 | 0.445 | 0.91 |
| HS.543242 | 110.97 | 16.79 | 121.41 | 14.53 | 5.34 | 0.033 | 0.446 | 0.91 |
| SYCP2L | 101.50 | 4.77 | 111.06 | 5.94 | 12.87 | 0.006 | 0.437 | 0.91 |
| MS4A4A | 109.02 | 5.97 | 119.30 | 8.41 | 4.52 | 0.046 | 0.448 | 0.91 |
| KCNG2 | 103.91 | 4.01 | 113.73 | 4.78 | 5.71 | 0.029 | 0.445 | 0.91 |
| C9ORF117 | 104.30 | 4.37 | 114.21 | 6.45 | 8.15 | 0.015 | 0.451 | 0.91 |
| LOC283523 | 96.60 | 3.60 | 105.79 | 4.20 | 4.39 | 0.048 | 0.451 | 0.91 |
| TPM1 | 109.69 | 3.17 | 120.13 | 5.78 | 5.15 | 0.036 | 0.447 | 0.91 |
| COG5 | 229.04 | 74.91 | 250.85 | 76.08 | 6.20 | 0.025 | 0.449 | 0.91 |
| MOXD1 | 127.94 | 16.11 | 140.13 | 13.30 | 5.98 | 0.027 | 0.448 | 0.91 |
| LOC652585 | 103.72 | 10.06 | 113.60 | 6.94 | 5.40 | 0.033 | 0.445 | 0.91 |
| LOC642456 | 119.58 | 6.32 | 130.99 | 7.93 | 4.60 | 0.044 | 0.447 | 0.91 |
| MIR340 | 105.90 | 8.64 | 116.02 | 8.10 | 10.76 | 0.009 | 0.454 | 0.91 |
| LOC646041 | 108.58 | 6.62 | 118.97 | 6.83 | 16.56 | 0.004 | 0.459 | 0.91 |
| ZDHHC9 | 113.53 | 2.46 | 124.40 | 4.50 | 6.51 | 0.023 | 0.452 | 0.91 |
| TMPRSS11F | 98.35 | 3.51 | 107.77 | 3.89 | 4.98 | 0.038 | 0.447 | 0.91 |
| AKNAD1 | 111.89 | 2.96 | 122.62 | 4.76 | 9.43 | 0.011 | 0.463 | 0.91 |
| LOC652894 | 111.78 | 0.98 | 122.50 | 4.48 | 5.27 | 0.034 | 0.448 | 0.91 |
| TMEM37 | 94.36 | 1.57 | 103.42 | 3.75 | 6.83 | 0.021 | 0.448 | 0.91 |
| LENG1 | 134.97 | 1.56 | 147.93 | 3.06 | 6.77 | 0.021 | 0.448 | 0.91 |
| TEP1 | 110.38 | 7.68 | 120.99 | 5.96 | 5.62 | 0.030 | 0.445 | 0.91 |
| SULT1C4 | 105.85 | 4.72 | 116.03 | 6.00 | 6.21 | 0.025 | 0.449 | 0.91 |
| UBE2Z | 102.27 | 13.92 | 112.11 | 15.27 | 6.31 | 0.024 | 0.451 | 0.91 |
| LOC402096 | 98.41 | 4.98 | 107.88 | 2.62 | 4.86 | 0.040 | 0.445 | 0.91 |
| USP22 | 101.11 | 6.15 | 110.85 | 4.10 | 8.10 | 0.015 | 0.452 | 0.91 |
| SCUBE2 | 98.24 | 3.14 | 107.72 | 3.49 | 10.10 | 0.010 | 0.453 | 0.91 |
| RGS8 | 109.91 | 7.72 | 120.54 | 7.50 | 4.88 | 0.039 | 0.444 | 0.91 |
| LOC730841 | 127.71 | 5.90 | 140.07 | 6.51 | 6.02 | 0.027 | 0.448 | 0.91 |
| HS.565244 | 119.94 | 14.56 | 131.56 | 19.20 | 4.34 | 0.049 | 0.452 | 0.91 |
| SLC30A8 | 96.33 | 5.96 | 105.67 | 4.15 | 4.61 | 0.044 | 0.448 | 0.91 |
| KRTAP10-5 | 103.52 | 5.84 | 113.56 | 5.69 | 12.98 | 0.006 | 0.443 | 0.91 |
| LOC100128883 | 126.99 | 10.36 | 139.31 | 13.32 | 6.18 | 0.025 | 0.449 | 0.91 |
| LOC100133329 | 144.28 | 17.68 | 158.29 | 14.00 | 5.20 | 0.035 | 0.447 | 0.91 |
| NKIRAS2 | 271.24 | 45.79 | 297.61 | 45.15 | 6.26 | 0.025 | 0.450 | 0.91 |
| SST | 99.25 | 8.92 | 108.91 | 6.26 | 6.26 | 0.025 | 0.450 | 0.91 |
| LOC729626 | 99.86 | 7.40 | 109.60 | 6.17 | 6.35 | 0.024 | 0.452 | 0.91 |
| AMBN | 109.38 | 5.17 | 120.07 | 6.63 | 7.44 | 0.018 | 0.449 | 0.91 |
| LOC646144 | 135.86 | 10.99 | 149.15 | 11.40 | 27.67 | 0.001 | 0.456 | 0.91 |
| LOC728003 | 104.10 | 7.03 | 114.29 | 8.28 | 4.85 | 0.040 | 0.444 | 0.91 |
| HS.171169 | 116.91 | 4.98 | 128.36 | 7.57 | 7.63 | 0.017 | 0.446 | 0.91 |
| HS.542629 | 108.69 | 4.65 | 119.34 | 5.42 | 14.69 | 0.005 | 0.447 | 0.91 |
| HS.573486 | 106.67 | 8.31 | 117.13 | 9.06 | 11.68 | 0.007 | 0.450 | 0.91 |
| CYTSB | 124.38 | 2.38 | 136.60 | 2.18 | 30.59 | 0.001 | 0.450 | 0.91 |
| HS.98737 | 117.32 | 6.28 | 128.85 | 8.13 | 5.65 | 0.030 | 0.446 | 0.91 |
| FLJ30851 | 110.86 | 8.89 | 121.77 | 7.93 | 6.52 | 0.023 | 0.453 | 0.91 |
| LOC100130849 | 106.28 | 6.56 | 116.75 | 2.91 | 4.92 | 0.039 | 0.445 | 0.91 |
| SH3BGRL | 106.42 | 9.21 | 116.91 | 7.86 | 12.97 | 0.006 | 0.441 | 0.91 |
| LOC644122 | 115.40 | 12.10 | 126.78 | 14.02 | 9.33 | 0.011 | 0.463 | 0.91 |
| LOC729501 | 106.87 | 6.74 | 117.41 | 4.64 | 6.97 | 0.020 | 0.447 | 0.91 |
| FAM70A | 126.30 | 10.78 | 138.78 | 9.73 | 5.02 | 0.037 | 0.446 | 0.91 |
| FARP1 | 104.97 | 2.73 | 115.34 | 4.70 | 6.83 | 0.021 | 0.448 | 0.91 |
| GPR120 | 97.61 | 4.76 | 107.26 | 6.97 | 5.41 | 0.033 | 0.445 | 0.91 |
| HS.570535 | 107.25 | 4.26 | 117.85 | 7.47 | 5.43 | 0.032 | 0.444 | 0.91 |
| HS.60556 | 114.01 | 10.82 | 125.31 | 11.62 | 21.23 | 0.002 | 0.448 | 0.91 |
| HS.18849 | 115.44 | 3.08 | 126.88 | 3.60 | 6.13 | 0.026 | 0.449 | 0.91 |
| LOC647529 | 108.43 | 10.18 | 119.19 | 11.84 | 10.93 | 0.008 | 0.455 | 0.91 |
| CALML3 | 98.03 | 5.51 | 107.76 | 3.41 | 4.72 | 0.042 | 0.445 | 0.91 |
| OR1F1 | 106.84 | 10.04 | 117.45 | 8.45 | 7.61 | 0.017 | 0.446 | 0.91 |
| LOC643912 | 123.77 | 14.30 | 136.08 | 14.07 | 6.58 | 0.022 | 0.454 | 0.91 |
| UNC13A | 113.12 | 10.19 | 124.40 | 6.16 | 4.42 | 0.048 | 0.450 | 0.91 |
| TRIM23 | 108.91 | 4.89 | 119.79 | 2.61 | 8.17 | 0.015 | 0.451 | 0.91 |
| LOC653082 | 111.83 | 13.48 | 123.01 | 14.74 | 15.26 | 0.004 | 0.449 | 0.91 |
| LOC401876 | 101.61 | 3.75 | 111.79 | 4.92 | 7.43 | 0.018 | 0.449 | 0.91 |
| ZBTB1 | 112.40 | 3.29 | 123.67 | 3.31 | 14.76 | 0.005 | 0.452 | 0.91 |
| SSH1 | 121.17 | 16.19 | 133.33 | 14.68 | 13.07 | 0.006 | 0.445 | 0.91 |
| LOC100130008 | 109.44 | 5.21 | 120.42 | 5.50 | 8.16 | 0.015 | 0.451 | 0.91 |
| KIAA1751 | 104.80 | 3.09 | 115.31 | 3.98 | 9.65 | 0.011 | 0.462 | 0.91 |
| GGTL3 | 123.42 | 5.47 | 135.81 | 2.52 | 6.31 | 0.024 | 0.451 | 0.91 |
| HS.162734 | 120.42 | 7.97 | 132.52 | 12.78 | 4.34 | 0.049 | 0.452 | 0.91 |
| KRT18P42 | 98.61 | 2.24 | 108.52 | 2.85 | 26.04 | 0.001 | 0.438 | 0.91 |
| OR6K2 | 103.49 | 1.63 | 113.89 | 2.02 | 9.22 | 0.012 | 0.457 | 0.91 |
| THRA | 100.51 | 3.33 | 110.62 | 3.72 | 4.97 | 0.038 | 0.445 | 0.91 |
| ACRC | 128.55 | 12.67 | 141.49 | 13.37 | 32.04 | 0.001 | 0.489 | 0.91 |
| BMP1 | 92.97 | 2.43 | 102.35 | 3.21 | 18.07 | 0.003 | 0.452 | 0.91 |
| ATXN2L | 113.35 | 12.45 | 124.79 | 13.58 | 10.47 | 0.009 | 0.456 | 0.91 |
| TCL6 | 100.59 | 4.96 | 110.74 | 4.81 | 9.75 | 0.010 | 0.459 | 0.91 |
| LOC643090 | 99.50 | 9.07 | 109.56 | 11.52 | 4.68 | 0.043 | 0.447 | 0.91 |
| LOC642566 | 101.08 | 2.84 | 111.31 | 5.29 | 6.97 | 0.020 | 0.447 | 0.91 |
| ERCC-00112 | 94.23 | 1.50 | 103.76 | 4.42 | 5.07 | 0.037 | 0.446 | 0.91 |
| HS.23217 | 108.39 | 6.46 | 119.38 | 5.75 | 24.40 | 0.002 | 0.438 | 0.91 |
| HS.568659 | 117.58 | 1.87 | 129.51 | 5.74 | 4.92 | 0.039 | 0.445 | 0.91 |
| LOC402617 | 110.36 | 3.75 | 121.57 | 7.77 | 4.52 | 0.046 | 0.448 | 0.91 |
| LOC649186 | 116.21 | 6.19 | 128.04 | 10.77 | 4.47 | 0.047 | 0.449 | 0.91 |
| CLDN19 | 115.34 | 5.03 | 127.09 | 9.21 | 4.85 | 0.040 | 0.444 | 0.91 |
| CCR2 | 105.97 | 7.40 | 116.76 | 9.43 | 4.66 | 0.043 | 0.448 | 0.91 |
| TSPAN15 | 113.91 | 13.38 | 125.51 | 14.29 | 4.33 | 0.049 | 0.452 | 0.91 |
| HS.542413 | 120.91 | 2.17 | 133.23 | 3.05 | 14.23 | 0.005 | 0.448 | 0.91 |
| PTPRE | 103.41 | 7.06 | 113.96 | 9.86 | 4.97 | 0.038 | 0.446 | 0.91 |
| LOC649255 | 107.19 | 10.31 | 118.13 | 10.89 | 10.61 | 0.009 | 0.453 | 0.91 |
| SFRS11 | 295.91 | 139.61 | 326.11 | 141.62 | 4.33 | 0.050 | 0.452 | 0.91 |
| LOC728875 | 110.13 | 8.88 | 121.37 | 9.02 | 5.87 | 0.028 | 0.446 | 0.91 |
| NKIRAS2 | 106.39 | 3.20 | 117.26 | 4.94 | 5.70 | 0.029 | 0.445 | 0.91 |
| NCR1 | 98.46 | 3.40 | 108.52 | 3.75 | 7.06 | 0.019 | 0.449 | 0.91 |
| LOC644624 | 99.63 | 6.35 | 109.81 | 7.05 | 13.72 | 0.005 | 0.446 | 0.91 |
| LOC388210 | 103.60 | 1.65 | 114.19 | 1.71 | 5.85 | 0.028 | 0.446 | 0.91 |
| AFAP1L1 | 113.03 | 2.09 | 124.59 | 1.49 | 5.59 | 0.031 | 0.445 | 0.91 |
| MIR668 | 98.81 | 12.69 | 108.93 | 13.37 | 6.90 | 0.020 | 0.446 | 0.91 |
| C19ORF71 | 109.77 | 4.13 | 121.03 | 6.48 | 6.30 | 0.024 | 0.449 | 0.91 |
| TNFRSF11A | 110.08 | 6.06 | 121.38 | 3.82 | 5.34 | 0.033 | 0.446 | 0.91 |
| SDC3 | 108.94 | 5.85 | 120.14 | 9.16 | 5.35 | 0.033 | 0.446 | 0.91 |
| LOC127602 | 107.40 | 2.99 | 118.44 | 3.38 | 4.70 | 0.042 | 0.447 | 0.91 |
| CARKD | 399.28 | 127.27 | 440.44 | 127.88 | 34.43 | 0.001 | 0.504 | 0.91 |
| FABP7 | 100.39 | 3.57 | 110.76 | 4.76 | 4.87 | 0.040 | 0.445 | 0.91 |
| KCTD8 | 117.31 | 6.61 | 129.43 | 9.94 | 6.05 | 0.026 | 0.448 | 0.91 |
| RAD50 | 95.63 | 5.59 | 105.51 | 3.73 | 9.20 | 0.012 | 0.456 | 0.91 |
| STEAP1 | 120.79 | 1.27 | 133.29 | 0.81 | 16.12 | 0.004 | 0.456 | 0.91 |
| LOC100131811 | 96.57 | 1.54 | 106.56 | 2.53 | 4.87 | 0.040 | 0.445 | 0.91 |
| LOC644348 | 101.44 | 12.60 | 111.95 | 11.35 | 5.31 | 0.034 | 0.447 | 0.91 |
| LOC653184 | 116.48 | 5.96 | 128.57 | 8.68 | 5.28 | 0.034 | 0.447 | 0.91 |
| FBXL15 | 216.71 | 37.73 | 239.23 | 44.65 | 5.62 | 0.030 | 0.445 | 0.91 |
| HS.524256 | 107.21 | 5.10 | 118.35 | 3.88 | 8.74 | 0.013 | 0.456 | 0.91 |
| LOC391727 | 114.14 | 12.66 | 126.01 | 12.95 | 29.26 | 0.001 | 0.444 | 0.91 |
| LOC644229 | 109.80 | 4.72 | 121.23 | 7.35 | 7.01 | 0.020 | 0.449 | 0.91 |
| LOC642573 | 103.47 | 8.38 | 114.26 | 9.61 | 6.47 | 0.023 | 0.453 | 0.91 |
| HS.193951 | 116.85 | 5.02 | 129.05 | 4.53 | 29.35 | 0.001 | 0.453 | 0.91 |
| LOC646316 | 170.04 | 25.36 | 187.80 | 30.90 | 4.81 | 0.041 | 0.445 | 0.91 |
| HS.572896 | 106.24 | 4.26 | 117.42 | 7.51 | 4.80 | 0.041 | 0.444 | 0.90 |
| DHRS4 | 775.31 | 210.33 | 856.94 | 220.29 | 7.72 | 0.016 | 0.448 | 0.90 |
| HS.552427 | 110.48 | 7.00 | 122.12 | 5.77 | 4.67 | 0.043 | 0.447 | 0.90 |
| LOC643493 | 107.45 | 6.44 | 118.79 | 4.60 | 10.26 | 0.009 | 0.453 | 0.90 |
| ERC1 | 133.38 | 16.39 | 147.48 | 20.99 | 4.58 | 0.044 | 0.448 | 0.90 |
| AADAT | 113.66 | 9.80 | 125.67 | 8.25 | 8.78 | 0.013 | 0.457 | 0.90 |
| LIMCH1 | 93.75 | 1.30 | 103.67 | 1.42 | 6.87 | 0.021 | 0.446 | 0.90 |
| LOC728806 | 107.86 | 8.34 | 119.29 | 8.42 | 39.23 | 0.001 | 0.548 | 0.90 |
| KCNQ4 | 111.88 | 12.59 | 123.74 | 15.55 | 5.11 | 0.036 | 0.447 | 0.90 |
| LOC644257 | 115.70 | 6.17 | 127.99 | 9.45 | 5.83 | 0.028 | 0.445 | 0.90 |
| OR10J1 | 111.07 | 6.33 | 122.86 | 8.73 | 5.01 | 0.038 | 0.446 | 0.90 |
| CTAGE5 | 110.33 | 8.26 | 122.06 | 11.31 | 5.68 | 0.030 | 0.446 | 0.90 |
| SCARNA3 | 108.24 | 2.49 | 119.75 | 4.12 | 7.79 | 0.016 | 0.449 | 0.90 |
| CRTAP | 106.95 | 14.14 | 118.32 | 11.35 | 5.44 | 0.032 | 0.445 | 0.90 |
| LOC731823 | 104.86 | 10.86 | 116.02 | 10.90 | 71.90 | 0.000 | 0.703 | 0.90 |
| LOC643697 | 110.04 | 8.15 | 121.76 | 6.14 | 10.10 | 0.010 | 0.454 | 0.90 |
| HS.570308 | 108.56 | 12.91 | 120.13 | 11.93 | 6.59 | 0.022 | 0.454 | 0.90 |
| LOC642816 | 114.43 | 10.46 | 126.64 | 10.83 | 7.74 | 0.016 | 0.450 | 0.90 |
| HS.570034 | 115.65 | 6.51 | 128.00 | 5.40 | 9.48 | 0.011 | 0.465 | 0.90 |
| LOC389852 | 104.96 | 7.63 | 116.18 | 7.10 | 4.95 | 0.038 | 0.445 | 0.90 |
| HSDL1 | 120.03 | 13.41 | 132.86 | 10.60 | 4.93 | 0.039 | 0.446 | 0.90 |
| LOC643358 | 14163.76 | 4891.26 | 15678.28 | 5431.80 | 4.77 | 0.041 | 0.444 | 0.90 |
| LOC642076 | 197.64 | 4.77 | 218.79 | 10.95 | 4.47 | 0.047 | 0.449 | 0.90 |
| AKR1E2 | 108.33 | 5.50 | 119.92 | 7.27 | 8.96 | 0.012 | 0.453 | 0.90 |
| PAK3 | 110.75 | 4.38 | 122.63 | 2.07 | 5.29 | 0.034 | 0.447 | 0.90 |
| HS.129800 | 112.60 | 3.86 | 124.68 | 3.82 | 8.67 | 0.013 | 0.451 | 0.90 |
| LOC399937 | 110.98 | 5.01 | 122.89 | 6.33 | 9.25 | 0.011 | 0.457 | 0.90 |
| LOC642245 | 116.83 | 4.72 | 129.37 | 5.64 | 9.44 | 0.011 | 0.464 | 0.90 |
| DCUN1D1 | 101.43 | 2.27 | 112.33 | 4.70 | 4.98 | 0.038 | 0.447 | 0.90 |
| BOC | 92.97 | 1.23 | 102.97 | 1.67 | 6.98 | 0.020 | 0.448 | 0.90 |
| LOC440005 | 104.24 | 7.39 | 115.47 | 5.79 | 4.54 | 0.045 | 0.449 | 0.90 |
| TMEM1 | 222.46 | 65.83 | 246.42 | 71.27 | 7.15 | 0.019 | 0.451 | 0.90 |
| RAB15 | 102.51 | 4.19 | 113.55 | 6.18 | 4.61 | 0.044 | 0.447 | 0.90 |
| VAPA | 100.60 | 9.11 | 111.44 | 9.71 | 6.44 | 0.023 | 0.453 | 0.90 |
| MYL1 | 112.98 | 8.65 | 125.17 | 12.22 | 5.24 | 0.035 | 0.447 | 0.90 |
| OR10X1 | 108.08 | 9.29 | 119.74 | 12.28 | 6.04 | 0.026 | 0.448 | 0.90 |
| DNAI2 | 101.25 | 9.00 | 112.18 | 6.20 | 5.54 | 0.031 | 0.444 | 0.90 |
| CHGA | 110.21 | 4.53 | 122.12 | 7.29 | 6.53 | 0.023 | 0.453 | 0.90 |
| CRYBA1 | 106.71 | 5.65 | 118.26 | 9.63 | 4.78 | 0.041 | 0.444 | 0.90 |
| PPP1R1B | 104.46 | 10.03 | 115.76 | 7.58 | 6.05 | 0.026 | 0.448 | 0.90 |
| HS.568058 | 127.07 | 9.82 | 140.83 | 6.06 | 5.84 | 0.028 | 0.446 | 0.90 |
| LOC100130539 | 102.75 | 6.23 | 113.88 | 5.96 | 13.21 | 0.006 | 0.445 | 0.90 |
| HS.581691 | 103.05 | 6.39 | 114.23 | 9.15 | 5.52 | 0.031 | 0.445 | 0.90 |
| LOC100131960 | 109.97 | 8.23 | 121.92 | 5.78 | 6.05 | 0.026 | 0.449 | 0.90 |
| SPEG | 117.48 | 8.90 | 130.24 | 10.58 | 13.00 | 0.006 | 0.443 | 0.90 |
| C2ORF80 | 110.78 | 1.50 | 122.82 | 1.73 | 7.45 | 0.018 | 0.448 | 0.90 |
| LOC650339 | 118.02 | 4.97 | 130.86 | 5.03 | 6.01 | 0.027 | 0.448 | 0.90 |
| WWOX | 100.35 | 1.77 | 111.28 | 4.77 | 4.96 | 0.038 | 0.446 | 0.90 |
| LOC100132308 | 120.80 | 1.27 | 133.95 | 4.51 | 4.91 | 0.039 | 0.445 | 0.90 |
| C9ORF84 | 106.73 | 8.96 | 118.36 | 9.12 | 26.02 | 0.001 | 0.433 | 0.90 |
| LOC645993 | 107.89 | 0.98 | 119.65 | 3.78 | 4.69 | 0.043 | 0.446 | 0.90 |
| LSM14B | 121.27 | 16.25 | 134.52 | 17.02 | 4.64 | 0.043 | 0.447 | 0.90 |
| HS.562585 | 99.47 | 5.82 | 110.33 | 2.88 | 4.65 | 0.043 | 0.447 | 0.90 |
| LOC100128857 | 107.67 | 6.36 | 119.44 | 7.86 | 4.50 | 0.046 | 0.448 | 0.90 |
| ABHD15 | 258.44 | 83.53 | 286.72 | 77.77 | 5.37 | 0.033 | 0.446 | 0.90 |
| CAPSL | 110.97 | 8.98 | 123.11 | 9.08 | 4.85 | 0.040 | 0.444 | 0.90 |
| FAM44A | 117.14 | 12.08 | 129.98 | 10.70 | 11.91 | 0.007 | 0.444 | 0.90 |
| SPATC1 | 122.82 | 4.20 | 136.29 | 7.11 | 6.30 | 0.024 | 0.450 | 0.90 |
| LOC653051 | 104.88 | 10.45 | 116.38 | 9.06 | 11.81 | 0.007 | 0.446 | 0.90 |
| C6ORF35 | 101.09 | 7.39 | 112.19 | 7.19 | 4.49 | 0.046 | 0.448 | 0.90 |
| FLJ45202 | 108.34 | 1.26 | 120.27 | 2.57 | 12.12 | 0.007 | 0.443 | 0.90 |
| LOC729737 | 110.55 | 2.53 | 122.73 | 3.35 | 9.91 | 0.010 | 0.458 | 0.90 |
| LOC442057 | 135.30 | 1.13 | 150.23 | 3.93 | 5.56 | 0.031 | 0.445 | 0.90 |
| KCNE4 | 98.37 | 5.75 | 109.24 | 7.43 | 5.01 | 0.038 | 0.447 | 0.90 |
| LOC401357 | 105.94 | 9.50 | 117.64 | 8.71 | 6.61 | 0.022 | 0.454 | 0.90 |
| MMP23B | 109.39 | 8.03 | 121.47 | 8.85 | 12.98 | 0.006 | 0.442 | 0.90 |
| LOC642345 | 104.92 | 6.82 | 116.52 | 5.85 | 13.89 | 0.005 | 0.450 | 0.90 |
| LOC402217 | 104.75 | 10.80 | 116.35 | 8.00 | 5.58 | 0.031 | 0.445 | 0.90 |
| LOC644411 | 108.20 | 3.55 | 120.20 | 4.33 | 6.29 | 0.024 | 0.449 | 0.90 |
| PSME1 | 151.45 | 18.43 | 168.27 | 23.16 | 5.43 | 0.032 | 0.445 | 0.90 |
| LOC646422 | 108.47 | 12.79 | 120.54 | 8.67 | 4.52 | 0.046 | 0.449 | 0.90 |
| LOC100133403 | 98.85 | 3.48 | 109.87 | 5.31 | 7.51 | 0.017 | 0.447 | 0.90 |
| TREX1 | 108.34 | 2.24 | 120.42 | 3.30 | 5.30 | 0.034 | 0.447 | 0.90 |
| PRELP | 107.38 | 5.58 | 119.36 | 2.04 | 4.58 | 0.044 | 0.448 | 0.90 |
| POM121L1P | 105.00 | 4.33 | 116.72 | 5.55 | 12.79 | 0.006 | 0.438 | 0.90 |
| BZW1 | 97.75 | 5.89 | 108.67 | 4.13 | 4.97 | 0.038 | 0.446 | 0.90 |
| SLC35C2 | 105.25 | 5.95 | 117.02 | 6.72 | 4.98 | 0.038 | 0.447 | 0.90 |
| MLL3 | 147.67 | 4.40 | 164.19 | 2.34 | 4.82 | 0.040 | 0.445 | 0.90 |
| LOC649979 | 104.23 | 3.81 | 115.90 | 5.86 | 8.09 | 0.015 | 0.452 | 0.90 |
| LOC650406 | 112.74 | 3.61 | 125.38 | 4.72 | 7.29 | 0.018 | 0.450 | 0.90 |
| LOC442442 | 135.87 | 3.16 | 151.11 | 8.57 | 4.85 | 0.040 | 0.444 | 0.90 |
| CXORF40A | 113.37 | 11.29 | 126.10 | 7.15 | 5.28 | 0.034 | 0.447 | 0.90 |
| HS.541092 | 116.84 | 8.93 | 129.95 | 10.14 | 5.52 | 0.031 | 0.445 | 0.90 |
| P2RY2 | 114.05 | 0.45 | 126.86 | 3.36 | 5.82 | 0.028 | 0.444 | 0.90 |
| MMPL1 | 108.39 | 6.69 | 120.57 | 10.92 | 4.67 | 0.043 | 0.447 | 0.90 |
| HSFY2 | 102.90 | 7.67 | 114.46 | 4.98 | 6.08 | 0.026 | 0.449 | 0.90 |
| LOC100129674 | 111.39 | 7.74 | 123.91 | 8.15 | 7.04 | 0.020 | 0.449 | 0.90 |
| PHF7 | 132.01 | 11.06 | 146.86 | 6.73 | 4.95 | 0.039 | 0.446 | 0.90 |
| LOC727732 | 106.83 | 7.88 | 118.85 | 9.32 | 13.27 | 0.006 | 0.445 | 0.90 |
| HOXA3 | 107.95 | 5.39 | 120.12 | 6.29 | 4.86 | 0.040 | 0.445 | 0.90 |
| LOC654172 | 108.07 | 11.80 | 120.26 | 10.40 | 4.50 | 0.046 | 0.449 | 0.90 |
| C10ORF39 | 119.35 | 7.99 | 132.82 | 11.27 | 6.13 | 0.026 | 0.449 | 0.90 |
| DEPDC7 | 109.81 | 9.60 | 122.22 | 14.49 | 4.33 | 0.049 | 0.452 | 0.90 |
| SYNPO2L | 98.69 | 4.00 | 109.84 | 6.12 | 9.02 | 0.012 | 0.454 | 0.90 |
| PLEC1 | 96.14 | 2.29 | 107.03 | 1.22 | 10.57 | 0.009 | 0.455 | 0.90 |
| SNRPN | 205.31 | 43.96 | 228.56 | 41.24 | 11.03 | 0.008 | 0.457 | 0.90 |
| LDHC | 113.87 | 3.98 | 126.79 | 8.35 | 4.50 | 0.046 | 0.449 | 0.90 |
| MEPE | 101.20 | 9.59 | 112.69 | 7.55 | 7.05 | 0.020 | 0.450 | 0.90 |
| LOC440335 | 104.55 | 6.46 | 116.42 | 2.54 | 5.18 | 0.035 | 0.447 | 0.90 |
| LOC648196 | 117.07 | 6.99 | 130.37 | 6.76 | 92.74 | 0.000 | 0.916 | 0.90 |
| LOC651029 | 115.96 | 8.20 | 129.12 | 9.56 | 10.23 | 0.009 | 0.452 | 0.90 |
| GJD3 | 95.51 | 3.81 | 106.35 | 1.36 | 5.88 | 0.028 | 0.446 | 0.90 |
| RFX4 | 105.64 | 6.39 | 117.63 | 8.50 | 4.61 | 0.044 | 0.447 | 0.90 |
| HS.460114 | 110.46 | 3.76 | 123.01 | 5.12 | 5.95 | 0.027 | 0.447 | 0.90 |
| FAM55C | 116.37 | 8.21 | 129.61 | 11.93 | 6.16 | 0.025 | 0.448 | 0.90 |
| APM-1 | 109.20 | 8.19 | 121.62 | 7.86 | 11.22 | 0.008 | 0.456 | 0.90 |
| NAALADL1 | 163.50 | 24.58 | 182.11 | 18.62 | 4.34 | 0.049 | 0.452 | 0.90 |
| OR1L4 | 106.14 | 5.24 | 118.24 | 4.63 | 4.44 | 0.047 | 0.450 | 0.90 |
| ZNF728 | 136.13 | 17.35 | 151.66 | 15.31 | 9.87 | 0.010 | 0.458 | 0.90 |
| CARD17 | 107.84 | 5.51 | 120.15 | 2.08 | 6.17 | 0.025 | 0.448 | 0.90 |
| LOC647521 | 111.63 | 1.90 | 124.39 | 0.76 | 14.77 | 0.005 | 0.452 | 0.90 |
| ARSA | 122.10 | 10.18 | 136.05 | 5.94 | 5.62 | 0.030 | 0.446 | 0.90 |
| ACSS3 | 106.90 | 5.52 | 119.13 | 6.79 | 4.36 | 0.049 | 0.452 | 0.90 |
| LOC646429 | 102.97 | 5.55 | 114.78 | 9.98 | 4.60 | 0.044 | 0.448 | 0.90 |
| SPRR2E | 104.34 | 9.21 | 116.31 | 7.12 | 4.96 | 0.038 | 0.445 | 0.90 |
| LOC440261 | 106.25 | 8.41 | 118.45 | 8.66 | 10.79 | 0.008 | 0.455 | 0.90 |
| LOC728081 | 116.68 | 9.39 | 130.14 | 13.89 | 5.10 | 0.036 | 0.447 | 0.90 |
| ICAM4 | 116.79 | 2.84 | 130.26 | 1.62 | 6.98 | 0.020 | 0.448 | 0.90 |
| LOC399753 | 98.13 | 6.77 | 109.46 | 3.08 | 4.58 | 0.044 | 0.448 | 0.90 |
| GNAS | 108.47 | 13.22 | 121.00 | 14.16 | 9.18 | 0.012 | 0.455 | 0.90 |
| MIR193A | 98.82 | 6.24 | 110.25 | 8.86 | 6.17 | 0.025 | 0.448 | 0.90 |
| LOC652438 | 97.94 | 7.77 | 109.28 | 11.11 | 5.31 | 0.034 | 0.447 | 0.90 |
| CPM | 98.37 | 2.75 | 109.76 | 3.21 | 5.56 | 0.031 | 0.445 | 0.90 |
| JMJD2A | 121.41 | 10.61 | 135.48 | 7.23 | 5.57 | 0.031 | 0.445 | 0.90 |
| PCDHB17 | 108.50 | 4.47 | 121.07 | 5.49 | 14.85 | 0.005 | 0.452 | 0.90 |
| LOC283116 | 105.75 | 8.81 | 118.01 | 5.73 | 5.22 | 0.035 | 0.448 | 0.90 |
| MRPL55 | 110.16 | 3.27 | 122.93 | 4.12 | 20.01 | 0.002 | 0.449 | 0.90 |
| CDK3 | 109.58 | 4.59 | 122.29 | 5.11 | 8.50 | 0.014 | 0.452 | 0.90 |
| GOLGA8F | 114.03 | 7.76 | 127.26 | 3.35 | 4.93 | 0.039 | 0.445 | 0.90 |
| C18ORF51 | 107.88 | 7.34 | 120.41 | 11.06 | 4.55 | 0.045 | 0.448 | 0.90 |
| HS.543163 | 124.94 | 8.82 | 139.45 | 11.24 | 9.27 | 0.011 | 0.459 | 0.90 |
| NTNG1 | 105.75 | 12.09 | 118.03 | 10.71 | 4.45 | 0.047 | 0.450 | 0.90 |
| LOC727858 | 100.80 | 6.04 | 112.51 | 8.99 | 6.79 | 0.021 | 0.449 | 0.90 |
| LOC728003 | 105.97 | 13.37 | 118.28 | 14.46 | 12.69 | 0.006 | 0.440 | 0.90 |
| GGT8P | 102.15 | 5.53 | 114.03 | 5.80 | 39.64 | 0.001 | 0.567 | 0.90 |
| HS.48729 | 116.77 | 6.52 | 130.35 | 5.39 | 5.38 | 0.033 | 0.445 | 0.90 |
| KCNT2 | 109.42 | 10.08 | 122.16 | 9.02 | 20.69 | 0.002 | 0.438 | 0.90 |
| PRR20B | 101.93 | 9.06 | 113.81 | 6.83 | 8.30 | 0.014 | 0.451 | 0.90 |
| ADAMTSL1 | 104.36 | 7.53 | 116.56 | 3.08 | 4.74 | 0.042 | 0.445 | 0.90 |
| BTBD3 | 148.73 | 17.67 | 166.12 | 17.92 | 8.44 | 0.014 | 0.452 | 0.90 |
| DYRK1B | 116.60 | 6.82 | 130.25 | 6.72 | 14.19 | 0.005 | 0.449 | 0.90 |
| RGPD5 | 99.93 | 0.38 | 111.63 | 4.23 | 4.41 | 0.048 | 0.450 | 0.90 |
| PACRG | 118.34 | 11.00 | 132.19 | 5.62 | 4.38 | 0.048 | 0.451 | 0.90 |
| WDR17 | 109.72 | 12.24 | 122.63 | 12.28 | 4.75 | 0.042 | 0.444 | 0.89 |
| MIR876 | 99.91 | 7.43 | 111.67 | 6.49 | 6.07 | 0.026 | 0.449 | 0.89 |
| FAM21C | 132.44 | 15.12 | 148.04 | 12.42 | 9.27 | 0.011 | 0.458 | 0.89 |
| LOC344887 | 115.26 | 14.47 | 128.84 | 10.95 | 4.78 | 0.041 | 0.444 | 0.89 |
| CCR9 | 117.00 | 8.38 | 130.84 | 13.03 | 4.88 | 0.040 | 0.444 | 0.89 |
| VWA3A | 112.17 | 5.40 | 125.45 | 2.17 | 6.47 | 0.023 | 0.452 | 0.89 |
| PPL | 113.13 | 4.17 | 126.53 | 8.43 | 5.45 | 0.032 | 0.445 | 0.89 |
| LOC100132310 | 98.90 | 7.50 | 110.62 | 5.79 | 5.30 | 0.034 | 0.447 | 0.89 |
| LOC388327 | 102.52 | 8.67 | 114.70 | 10.69 | 10.34 | 0.009 | 0.452 | 0.89 |
| NR6A1 | 108.58 | 2.93 | 121.49 | 7.38 | 4.80 | 0.041 | 0.444 | 0.89 |
| MIRLET7C | 103.29 | 4.49 | 115.58 | 2.77 | 10.53 | 0.009 | 0.454 | 0.89 |
| KIF5A | 115.39 | 5.98 | 129.14 | 8.19 | 6.85 | 0.021 | 0.447 | 0.89 |
| LOC645164 | 109.29 | 8.05 | 122.32 | 4.69 | 4.46 | 0.047 | 0.449 | 0.89 |
| LOC651131 | 102.74 | 10.63 | 114.99 | 11.73 | 19.26 | 0.003 | 0.453 | 0.89 |
| HS.85989 | 106.67 | 9.57 | 119.40 | 13.68 | 4.49 | 0.046 | 0.449 | 0.89 |
| LOC647212 | 105.50 | 14.26 | 118.08 | 10.03 | 4.33 | 0.049 | 0.452 | 0.89 |
| PCSK9 | 95.93 | 1.99 | 107.39 | 2.58 | 12.58 | 0.006 | 0.441 | 0.89 |
| TLL1 | 105.13 | 5.87 | 117.69 | 9.78 | 4.61 | 0.044 | 0.447 | 0.89 |
| LOC650580 | 117.82 | 5.30 | 131.93 | 6.04 | 14.45 | 0.005 | 0.442 | 0.89 |
| RAB2B | 916.35 | 313.94 | 1026.10 | 314.99 | 20.81 | 0.002 | 0.439 | 0.89 |
| LOC131055 | 112.01 | 4.47 | 125.45 | 4.01 | 4.44 | 0.047 | 0.450 | 0.89 |
| LOC728210 | 103.59 | 7.31 | 116.04 | 5.30 | 7.93 | 0.016 | 0.451 | 0.89 |
| LOC100131658 | 107.76 | 2.40 | 120.74 | 2.31 | 5.84 | 0.028 | 0.446 | 0.89 |
| ECAT1 | 113.81 | 14.33 | 127.53 | 10.05 | 5.50 | 0.032 | 0.446 | 0.89 |
| HS.22305 | 128.38 | 12.60 | 143.87 | 13.92 | 5.49 | 0.032 | 0.446 | 0.89 |
| CLEC11A | 122.74 | 0.68 | 137.56 | 1.65 | 11.24 | 0.008 | 0.456 | 0.89 |
| LOC400713 | 112.64 | 6.30 | 126.25 | 8.90 | 7.13 | 0.019 | 0.449 | 0.89 |
| TMEM217 | 109.63 | 6.50 | 122.90 | 4.22 | 7.44 | 0.018 | 0.448 | 0.89 |
| HS.567096 | 106.42 | 9.03 | 119.32 | 9.37 | 5.63 | 0.030 | 0.446 | 0.89 |
| LOC651834 | 98.73 | 9.04 | 110.69 | 4.54 | 4.34 | 0.049 | 0.452 | 0.89 |
| CECR1 | 101.41 | 0.75 | 113.70 | 3.73 | 5.40 | 0.033 | 0.445 | 0.89 |
| LOC389365 | 111.39 | 3.44 | 124.89 | 6.88 | 6.07 | 0.026 | 0.448 | 0.89 |
| LOC649546 | 108.40 | 10.50 | 121.54 | 13.74 | 4.32 | 0.050 | 0.452 | 0.89 |
| HS.118769 | 108.05 | 5.47 | 121.18 | 2.03 | 6.51 | 0.023 | 0.452 | 0.89 |
| LOC729549 | 101.09 | 8.59 | 113.37 | 7.90 | 5.16 | 0.036 | 0.447 | 0.89 |
| EPHA2 | 105.13 | 0.95 | 117.91 | 3.39 | 5.10 | 0.036 | 0.447 | 0.89 |
| GUCY2F | 113.69 | 8.93 | 127.52 | 7.28 | 12.94 | 0.006 | 0.441 | 0.89 |
| CYB5RL | 112.71 | 0.76 | 126.43 | 4.13 | 6.94 | 0.020 | 0.447 | 0.89 |
| SIKE | 137.73 | 17.88 | 154.50 | 21.30 | 5.60 | 0.030 | 0.445 | 0.89 |
| UBE2B | 122.52 | 2.33 | 137.48 | 4.59 | 4.42 | 0.048 | 0.450 | 0.89 |
| HS.266249 | 104.95 | 13.32 | 117.78 | 11.27 | 4.99 | 0.038 | 0.447 | 0.89 |
| PSMA1 | 113.30 | 5.46 | 127.16 | 2.67 | 5.61 | 0.030 | 0.445 | 0.89 |
| RHOA | 4628.63 | 2020.81 | 5195.03 | 1941.70 | 7.85 | 0.016 | 0.450 | 0.89 |
| LOC644366 | 111.29 | 6.49 | 124.92 | 6.43 | 10.47 | 0.009 | 0.455 | 0.89 |
| MAGEB5 | 108.07 | 4.12 | 121.33 | 5.49 | 8.46 | 0.014 | 0.452 | 0.89 |
| RPS26P11 | 104.20 | 9.77 | 116.98 | 14.69 | 4.49 | 0.046 | 0.449 | 0.89 |
| YTHDF3 | 273.05 | 90.01 | 306.57 | 85.10 | 4.31 | 0.050 | 0.452 | 0.89 |
| DPPA3 | 101.42 | 5.02 | 113.89 | 6.38 | 10.07 | 0.010 | 0.455 | 0.89 |
| FZD5 | 122.66 | 5.03 | 137.78 | 4.86 | 5.78 | 0.029 | 0.443 | 0.89 |
| HS.552957 | 128.86 | 8.81 | 144.77 | 5.33 | 7.73 | 0.016 | 0.449 | 0.89 |
| CNTNAP3B | 107.75 | 8.26 | 121.06 | 11.85 | 4.32 | 0.050 | 0.452 | 0.89 |
| LOC643486 | 98.67 | 2.81 | 110.90 | 7.37 | 4.51 | 0.046 | 0.449 | 0.89 |
| DCST1 | 105.19 | 5.61 | 118.22 | 9.02 | 6.50 | 0.023 | 0.452 | 0.89 |
| MIR1262 | 98.93 | 3.32 | 111.20 | 5.32 | 9.91 | 0.010 | 0.458 | 0.89 |
| PROCR | 187.44 | 21.41 | 210.72 | 26.15 | 7.14 | 0.019 | 0.450 | 0.89 |
| IQCJ | 100.74 | 8.91 | 113.28 | 5.20 | 4.48 | 0.046 | 0.449 | 0.89 |
| CALN1 | 104.61 | 3.52 | 117.64 | 1.64 | 4.68 | 0.043 | 0.447 | 0.89 |
| FLJ23356 | 109.30 | 13.83 | 122.91 | 12.63 | 6.51 | 0.023 | 0.452 | 0.89 |
| LOC645033 | 111.47 | 13.61 | 125.36 | 14.71 | 4.59 | 0.044 | 0.447 | 0.89 |
| LOC729837 | 110.36 | 6.11 | 124.11 | 3.77 | 7.55 | 0.017 | 0.446 | 0.89 |
| HS.423734 | 108.87 | 6.18 | 122.45 | 3.98 | 8.74 | 0.013 | 0.456 | 0.89 |
| TMOD2 | 136.03 | 9.51 | 153.00 | 13.38 | 4.93 | 0.039 | 0.445 | 0.89 |
| PRLR | 103.20 | 7.97 | 116.10 | 9.61 | 13.63 | 0.005 | 0.447 | 0.89 |
| ABCC6P1 | 106.45 | 6.34 | 119.78 | 7.99 | 5.49 | 0.032 | 0.446 | 0.89 |
| CAPS | 107.73 | 9.12 | 121.21 | 7.56 | 6.90 | 0.020 | 0.446 | 0.89 |
| LOC442448 | 118.79 | 3.92 | 133.68 | 4.04 | 9.73 | 0.010 | 0.459 | 0.89 |
| LOC729438 | 106.81 | 5.64 | 120.20 | 7.28 | 8.42 | 0.014 | 0.451 | 0.89 |
| DNAJB4 | 120.63 | 1.94 | 135.76 | 2.91 | 5.69 | 0.030 | 0.446 | 0.89 |
| GRIN3B | 102.74 | 6.53 | 115.62 | 8.52 | 4.86 | 0.040 | 0.445 | 0.89 |
| CR1L | 118.02 | 5.40 | 132.85 | 6.81 | 16.16 | 0.004 | 0.457 | 0.89 |
| LOC340239 | 103.87 | 7.24 | 116.93 | 6.91 | 7.83 | 0.016 | 0.449 | 0.89 |
| LOC644153 | 107.48 | 6.48 | 121.00 | 7.13 | 6.23 | 0.025 | 0.449 | 0.89 |
| LRRC52 | 101.29 | 9.56 | 114.03 | 12.43 | 7.66 | 0.017 | 0.447 | 0.89 |
| PKIG | 112.47 | 5.00 | 126.63 | 10.19 | 4.70 | 0.042 | 0.446 | 0.89 |
| HS.146614 | 109.27 | 4.75 | 123.02 | 4.42 | 5.06 | 0.037 | 0.447 | 0.89 |
| SLC28A2 | 103.35 | 6.79 | 116.37 | 5.89 | 12.22 | 0.007 | 0.440 | 0.89 |
| ERBB3 | 106.32 | 10.03 | 119.72 | 13.43 | 5.25 | 0.034 | 0.447 | 0.89 |
| HS.334066 | 107.11 | 10.18 | 120.65 | 10.17 | 8.72 | 0.013 | 0.454 | 0.89 |
| C6ORF145 | 131.66 | 10.12 | 148.32 | 8.39 | 10.60 | 0.009 | 0.453 | 0.89 |
| IHPK3 | 117.29 | 6.21 | 132.14 | 4.81 | 17.19 | 0.003 | 0.456 | 0.89 |
| HS.128689 | 106.20 | 7.69 | 119.66 | 7.65 | 15.77 | 0.004 | 0.450 | 0.89 |
| SNORD90 | 95.82 | 2.32 | 107.97 | 1.10 | 17.33 | 0.003 | 0.455 | 0.89 |
| LOC100133599 | 112.27 | 9.43 | 126.51 | 8.48 | 4.49 | 0.046 | 0.448 | 0.89 |
| HS.576530 | 109.73 | 2.79 | 123.67 | 6.19 | 5.49 | 0.032 | 0.445 | 0.89 |
| PLGLA1 | 107.14 | 6.26 | 120.77 | 1.70 | 4.36 | 0.049 | 0.451 | 0.89 |
| ZNF649 | 114.85 | 4.54 | 129.48 | 8.80 | 5.37 | 0.033 | 0.445 | 0.89 |
| C9ORF50 | 110.11 | 7.68 | 124.14 | 10.07 | 5.20 | 0.035 | 0.447 | 0.89 |
| RECQL5 | 115.93 | 4.11 | 130.71 | 7.63 | 7.25 | 0.018 | 0.450 | 0.89 |
| CHRNA3 | 109.61 | 8.78 | 123.59 | 10.98 | 4.57 | 0.045 | 0.448 | 0.89 |
| OVCH2 | 99.77 | 6.01 | 112.50 | 6.92 | 5.05 | 0.037 | 0.447 | 0.89 |
| CLDN22 | 108.84 | 5.03 | 122.72 | 5.27 | 6.00 | 0.027 | 0.448 | 0.89 |
| DNAJC5 | 184.23 | 43.02 | 207.76 | 47.90 | 6.12 | 0.026 | 0.450 | 0.89 |
| MR1 | 251.51 | 42.80 | 283.67 | 51.10 | 5.51 | 0.031 | 0.446 | 0.89 |
| ARSB | 112.39 | 8.34 | 126.77 | 9.68 | 6.41 | 0.024 | 0.454 | 0.89 |
| GSG1 | 116.56 | 12.35 | 131.47 | 11.93 | 60.46 | 0.000 | 0.718 | 0.89 |
| LOC728637 | 112.81 | 1.25 | 127.25 | 5.38 | 5.46 | 0.032 | 0.445 | 0.89 |
| LOC646491 | 121.33 | 6.76 | 136.86 | 8.51 | 5.84 | 0.028 | 0.445 | 0.89 |
| FOXA2 | 117.83 | 12.07 | 132.94 | 6.35 | 4.54 | 0.045 | 0.449 | 0.89 |
| LOC644005 | 103.63 | 6.94 | 116.93 | 11.64 | 4.47 | 0.047 | 0.449 | 0.89 |
| EMR4 | 95.92 | 3.81 | 108.26 | 7.05 | 6.57 | 0.022 | 0.454 | 0.89 |
| KCNK7 | 112.30 | 6.89 | 126.75 | 5.73 | 5.05 | 0.037 | 0.447 | 0.89 |
| AFF1 | 112.01 | 6.66 | 126.43 | 12.26 | 4.45 | 0.047 | 0.450 | 0.89 |
| LAIR1 | 110.66 | 2.10 | 124.91 | 5.22 | 4.98 | 0.038 | 0.446 | 0.89 |
| ZNF33A | 119.26 | 13.14 | 134.63 | 11.64 | 9.01 | 0.012 | 0.455 | 0.89 |
| JMJD2C | 133.94 | 6.84 | 151.21 | 5.21 | 16.16 | 0.004 | 0.458 | 0.89 |
| LOC650739 | 98.30 | 2.41 | 110.97 | 3.90 | 6.00 | 0.027 | 0.447 | 0.89 |
| LOC650118 | 102.05 | 5.85 | 115.21 | 9.08 | 5.95 | 0.027 | 0.448 | 0.89 |
| LOC729141 | 99.06 | 2.31 | 111.85 | 6.21 | 5.61 | 0.030 | 0.445 | 0.89 |
| UCN | 108.66 | 7.91 | 122.68 | 7.47 | 18.47 | 0.003 | 0.454 | 0.89 |
| BCL2L14 | 104.18 | 15.06 | 117.68 | 18.73 | 6.29 | 0.024 | 0.449 | 0.89 |
| LOC642644 | 106.66 | 6.06 | 120.51 | 4.40 | 9.85 | 0.010 | 0.458 | 0.89 |
| FAM48B1 | 107.04 | 4.68 | 120.95 | 4.55 | 22.12 | 0.002 | 0.452 | 0.89 |
| HS.544500 | 100.08 | 8.17 | 113.09 | 11.18 | 6.75 | 0.021 | 0.449 | 0.88 |
| MIR374B | 96.66 | 1.95 | 109.25 | 1.74 | 7.58 | 0.017 | 0.444 | 0.88 |
| DRD4 | 105.21 | 2.36 | 118.94 | 4.64 | 10.25 | 0.009 | 0.452 | 0.88 |
| XAGE2 | 106.07 | 11.08 | 119.93 | 15.11 | 5.88 | 0.028 | 0.446 | 0.88 |
| LOC646890 | 113.47 | 8.87 | 128.29 | 9.69 | 5.55 | 0.031 | 0.445 | 0.88 |
| TCP10 | 113.62 | 13.58 | 128.47 | 13.02 | 12.14 | 0.007 | 0.443 | 0.88 |
| MMP11 | 117.10 | 6.30 | 132.43 | 1.51 | 5.39 | 0.033 | 0.445 | 0.88 |
| HS.302742 | 110.33 | 5.74 | 124.79 | 10.00 | 4.92 | 0.039 | 0.445 | 0.88 |
| HS.581938 | 111.66 | 10.34 | 126.30 | 5.63 | 5.14 | 0.036 | 0.446 | 0.88 |
| SGPP2 | 102.02 | 4.19 | 115.44 | 5.64 | 6.99 | 0.020 | 0.449 | 0.88 |
| HS.584357 | 106.55 | 1.29 | 120.56 | 4.42 | 7.75 | 0.016 | 0.449 | 0.88 |
| HS.543097 | 112.38 | 4.25 | 127.17 | 1.58 | 9.60 | 0.011 | 0.464 | 0.88 |
| ATG2A | 231.67 | 43.27 | 262.18 | 45.32 | 9.84 | 0.010 | 0.458 | 0.88 |
| LOC645876 | 101.12 | 10.70 | 114.44 | 8.78 | 7.51 | 0.017 | 0.447 | 0.88 |
| FOXP1 | 103.39 | 7.54 | 117.02 | 5.91 | 10.97 | 0.008 | 0.454 | 0.88 |
| TRIM13 | 143.12 | 20.14 | 162.01 | 22.62 | 5.82 | 0.028 | 0.444 | 0.88 |
| LOC442501 | 113.30 | 13.01 | 128.28 | 12.05 | 15.13 | 0.004 | 0.449 | 0.88 |
| HS.549823 | 122.62 | 9.70 | 138.84 | 3.26 | 4.34 | 0.049 | 0.452 | 0.88 |
| WDR13 | 328.03 | 91.39 | 371.47 | 87.01 | 5.81 | 0.028 | 0.444 | 0.88 |
| LOC391771 | 111.61 | 4.83 | 126.42 | 7.58 | 5.22 | 0.035 | 0.447 | 0.88 |
| OTOF | 116.77 | 5.41 | 132.28 | 5.95 | 16.54 | 0.004 | 0.458 | 0.88 |
| HEATR7B2 | 105.16 | 9.97 | 119.13 | 13.48 | 6.29 | 0.024 | 0.449 | 0.88 |
| KCNA4 | 101.28 | 4.96 | 114.74 | 9.07 | 4.36 | 0.049 | 0.451 | 0.88 |
| LOC120376 | 103.39 | 7.63 | 117.14 | 10.20 | 5.64 | 0.030 | 0.447 | 0.88 |
| MGC13005 | 115.14 | 7.52 | 130.51 | 6.78 | 13.02 | 0.006 | 0.443 | 0.88 |
| HS.199565 | 111.89 | 7.57 | 126.84 | 5.84 | 7.63 | 0.017 | 0.446 | 0.88 |
| MXI1 | 106.99 | 3.06 | 121.29 | 1.77 | 5.13 | 0.036 | 0.447 | 0.88 |
| PLGLB2 | 112.02 | 5.62 | 127.01 | 6.70 | 5.96 | 0.027 | 0.448 | 0.88 |
| HS.121413 | 109.68 | 12.13 | 124.37 | 7.77 | 5.83 | 0.028 | 0.445 | 0.88 |
| RAD54L2 | 247.27 | 58.59 | 280.38 | 52.57 | 4.53 | 0.045 | 0.449 | 0.88 |
| OR7E24 | 108.59 | 5.84 | 123.15 | 2.27 | 6.85 | 0.021 | 0.447 | 0.88 |
| CRYAA | 114.87 | 4.96 | 130.26 | 3.28 | 13.30 | 0.006 | 0.445 | 0.88 |
| HS.131087 | 109.04 | 8.56 | 123.67 | 6.08 | 8.36 | 0.014 | 0.452 | 0.88 |
| DLX4 | 102.66 | 5.46 | 116.46 | 7.20 | 10.49 | 0.009 | 0.456 | 0.88 |
| LOC92154 | 102.17 | 7.71 | 115.93 | 3.66 | 4.88 | 0.039 | 0.444 | 0.88 |
| TUBB1 | 111.54 | 3.14 | 126.57 | 7.73 | 5.16 | 0.036 | 0.446 | 0.88 |
| CORO1B | 134.62 | 4.64 | 152.78 | 8.22 | 8.28 | 0.014 | 0.452 | 0.88 |
| C22ORF40 | 115.14 | 14.22 | 130.69 | 9.23 | 5.34 | 0.033 | 0.446 | 0.88 |
| FLJ40330 | 106.30 | 6.33 | 120.66 | 3.71 | 5.84 | 0.028 | 0.446 | 0.88 |
| GPR25 | 97.59 | 6.43 | 110.78 | 3.73 | 7.69 | 0.016 | 0.448 | 0.88 |
| DOPEY2 | 147.10 | 14.91 | 167.01 | 12.91 | 13.69 | 0.005 | 0.445 | 0.88 |
| MYLK | 111.90 | 13.27 | 127.05 | 10.89 | 9.81 | 0.010 | 0.457 | 0.88 |
| LOC729597 | 99.00 | 4.76 | 112.42 | 3.02 | 7.20 | 0.019 | 0.450 | 0.88 |
| CNPY1 | 113.47 | 11.42 | 128.86 | 10.62 | 6.26 | 0.025 | 0.450 | 0.88 |
| MIB2 | 168.47 | 9.49 | 191.32 | 16.32 | 5.28 | 0.034 | 0.447 | 0.88 |
| ERVWE1 | 97.70 | 6.74 | 110.96 | 7.69 | 4.92 | 0.039 | 0.445 | 0.88 |
| HS.540709 | 110.84 | 10.51 | 125.90 | 10.65 | 7.70 | 0.016 | 0.448 | 0.88 |
| CMPK2 | 115.14 | 4.40 | 130.79 | 4.72 | 6.60 | 0.022 | 0.453 | 0.88 |
| LOC100128904 | 100.58 | 1.55 | 114.27 | 4.35 | 7.85 | 0.016 | 0.450 | 0.88 |
| CEP63 | 99.96 | 6.77 | 113.56 | 5.49 | 12.08 | 0.007 | 0.444 | 0.88 |
| RAB1A | 271.67 | 57.24 | 308.73 | 49.63 | 4.51 | 0.046 | 0.449 | 0.88 |
| SLC34A2 | 106.98 | 12.08 | 121.59 | 13.54 | 12.24 | 0.007 | 0.440 | 0.88 |
| FLJ35848 | 113.71 | 9.38 | 129.24 | 8.75 | 6.35 | 0.024 | 0.451 | 0.88 |
| TSPYL2 | 150.46 | 20.75 | 171.01 | 24.01 | 6.02 | 0.026 | 0.449 | 0.88 |
| LOC552889 | 312.30 | 52.87 | 354.96 | 62.39 | 5.56 | 0.031 | 0.445 | 0.88 |
| LOC100130743 | 109.62 | 6.74 | 124.61 | 2.22 | 4.79 | 0.041 | 0.444 | 0.88 |
| LOC645113 | 111.00 | 2.49 | 126.20 | 2.18 | 81.88 | 0.000 | 1.007 | 0.88 |
| HS.444839 | 115.93 | 10.32 | 131.80 | 11.52 | 5.30 | 0.034 | 0.447 | 0.88 |
| KCNJ14 | 99.56 | 7.60 | 113.21 | 6.34 | 7.53 | 0.017 | 0.446 | 0.88 |
| LOC100130351 | 110.68 | 2.40 | 125.88 | 5.88 | 4.46 | 0.047 | 0.449 | 0.88 |
| LOC644496 | 110.42 | 4.68 | 125.59 | 5.08 | 15.09 | 0.004 | 0.449 | 0.88 |
| FLJ42258 | 121.97 | 5.82 | 138.73 | 4.70 | 11.20 | 0.008 | 0.456 | 0.88 |
| LOC645961 | 110.52 | 6.35 | 125.72 | 9.67 | 5.36 | 0.033 | 0.445 | 0.88 |
| CGA | 111.71 | 6.47 | 127.08 | 7.44 | 6.44 | 0.023 | 0.453 | 0.88 |
| FLJ41941 | 109.14 | 5.93 | 124.21 | 6.64 | 5.25 | 0.034 | 0.447 | 0.88 |
| CASZ1 | 258.70 | 64.07 | 294.46 | 52.23 | 4.86 | 0.040 | 0.445 | 0.88 |
| LOC729356 | 99.05 | 3.03 | 112.75 | 5.67 | 8.52 | 0.014 | 0.451 | 0.88 |
| LOC100133422 | 108.73 | 6.02 | 123.78 | 7.32 | 16.41 | 0.004 | 0.452 | 0.88 |
| ABLIM2 | 102.89 | 1.86 | 117.14 | 2.73 | 14.16 | 0.005 | 0.449 | 0.88 |
| LOC650517 | 106.86 | 3.65 | 121.68 | 9.21 | 4.55 | 0.045 | 0.449 | 0.88 |
| LOC647219 | 108.85 | 2.77 | 123.96 | 6.88 | 6.37 | 0.024 | 0.452 | 0.88 |
| SLITRK4 | 102.03 | 2.72 | 116.21 | 1.92 | 7.47 | 0.017 | 0.448 | 0.88 |
| LOC401730 | 97.19 | 7.15 | 110.70 | 7.20 | 5.15 | 0.036 | 0.447 | 0.88 |
| C6ORF89 | 149.70 | 28.51 | 170.56 | 34.21 | 4.82 | 0.040 | 0.445 | 0.88 |
| MGC39584 | 112.76 | 9.36 | 128.50 | 7.33 | 7.90 | 0.016 | 0.451 | 0.88 |
| C5ORF28 | 150.00 | 26.24 | 170.93 | 25.20 | 5.97 | 0.027 | 0.448 | 0.88 |
| CPT1A | 126.24 | 10.49 | 143.86 | 14.88 | 4.88 | 0.040 | 0.444 | 0.88 |
| C8ORF44 | 102.77 | 3.23 | 117.13 | 5.12 | 5.50 | 0.031 | 0.446 | 0.88 |
| LOC641802 | 121.62 | 1.73 | 138.64 | 5.17 | 7.00 | 0.020 | 0.449 | 0.88 |
| IKBKG | 715.53 | 300.23 | 815.83 | 296.37 | 10.22 | 0.009 | 0.452 | 0.88 |
| LOC728160 | 104.16 | 5.61 | 118.76 | 6.95 | 4.37 | 0.049 | 0.451 | 0.88 |
| HS.149495 | 107.64 | 3.41 | 122.74 | 4.66 | 9.18 | 0.012 | 0.456 | 0.88 |
| LOC654135 | 107.21 | 2.72 | 122.25 | 7.74 | 4.84 | 0.040 | 0.444 | 0.88 |
| LZTFL1 | 117.09 | 16.27 | 133.52 | 13.47 | 9.83 | 0.010 | 0.458 | 0.88 |
| HS.571292 | 113.79 | 3.02 | 129.76 | 2.53 | 26.28 | 0.001 | 0.446 | 0.88 |
| HS.574465 | 105.74 | 4.96 | 120.58 | 3.06 | 13.14 | 0.006 | 0.445 | 0.88 |
| HS.571967 | 106.43 | 8.61 | 121.42 | 11.87 | 5.24 | 0.035 | 0.447 | 0.88 |
| PADI3 | 105.38 | 4.28 | 120.23 | 5.36 | 10.19 | 0.009 | 0.452 | 0.88 |
| DUSP21 | 107.04 | 9.38 | 122.14 | 7.95 | 9.32 | 0.011 | 0.462 | 0.88 |
| HIST1H4F | 104.80 | 9.13 | 119.60 | 10.51 | 5.74 | 0.029 | 0.445 | 0.88 |
| PLCL4 | 111.63 | 19.53 | 127.41 | 23.62 | 6.52 | 0.023 | 0.453 | 0.88 |
| SLFN11 | 497.08 | 262.30 | 567.36 | 257.91 | 15.57 | 0.004 | 0.452 | 0.88 |
| LRRC3B | 101.70 | 0.23 | 116.09 | 5.60 | 4.30 | 0.050 | 0.452 | 0.88 |
| AHSA2 | 601.72 | 379.45 | 686.95 | 381.84 | 50.49 | 0.000 | 0.618 | 0.88 |
| LOC644345 | 110.82 | 8.89 | 126.52 | 12.77 | 4.33 | 0.049 | 0.452 | 0.88 |
| BST2 | 1332.14 | 504.70 | 1521.21 | 484.62 | 10.62 | 0.009 | 0.454 | 0.88 |
| EPHB4 | 100.75 | 6.85 | 115.05 | 8.15 | 7.48 | 0.017 | 0.447 | 0.88 |
| KCND3 | 112.72 | 9.54 | 128.74 | 4.85 | 4.81 | 0.041 | 0.444 | 0.88 |
| UNC13A | 99.00 | 7.04 | 113.08 | 8.22 | 6.29 | 0.024 | 0.449 | 0.88 |
| PARP10 | 158.81 | 17.57 | 181.42 | 22.08 | 5.22 | 0.035 | 0.447 | 0.88 |
| LOC728734 | 112.76 | 5.92 | 128.83 | 5.89 | 7.09 | 0.019 | 0.450 | 0.88 |
| TP53AP1 | 141.73 | 8.59 | 161.96 | 7.65 | 10.28 | 0.009 | 0.452 | 0.88 |
| MAP7 | 124.96 | 4.32 | 142.81 | 10.48 | 4.85 | 0.040 | 0.444 | 0.88 |
| LOC652837 | 147.28 | 7.18 | 168.32 | 4.85 | 6.28 | 0.024 | 0.449 | 0.87 |
| LOC643342 | 123.38 | 3.53 | 141.01 | 5.87 | 4.64 | 0.043 | 0.447 | 0.87 |
| C11ORF35 | 103.24 | 5.86 | 117.99 | 1.69 | 4.58 | 0.044 | 0.448 | 0.87 |
| PILRB | 116.87 | 6.30 | 133.57 | 3.37 | 7.12 | 0.019 | 0.450 | 0.87 |
| AMPD3 | 113.56 | 5.02 | 129.79 | 6.64 | 7.43 | 0.018 | 0.449 | 0.87 |
| HTR3B | 101.89 | 5.80 | 116.46 | 8.10 | 10.73 | 0.009 | 0.454 | 0.87 |
| CFC1 | 107.72 | 6.21 | 123.17 | 4.47 | 9.44 | 0.011 | 0.465 | 0.87 |
| LOC390660 | 128.20 | 11.27 | 146.58 | 11.44 | 10.44 | 0.009 | 0.455 | 0.87 |
| FARP1 | 110.91 | 6.71 | 126.86 | 6.03 | 10.23 | 0.009 | 0.452 | 0.87 |
| TP53I13 | 533.47 | 182.18 | 610.22 | 185.52 | 6.36 | 0.024 | 0.452 | 0.87 |
| HS.545716 | 102.59 | 6.58 | 117.35 | 11.36 | 5.34 | 0.033 | 0.446 | 0.87 |
| RASSF10 | 106.08 | 5.40 | 121.34 | 8.61 | 4.76 | 0.041 | 0.444 | 0.87 |
| LRCH4 | 529.92 | 208.40 | 606.19 | 226.89 | 6.36 | 0.024 | 0.452 | 0.87 |
| CNR1 | 128.60 | 8.22 | 147.12 | 11.72 | 8.36 | 0.014 | 0.452 | 0.87 |
| LOC644624 | 109.87 | 11.18 | 125.73 | 9.36 | 8.03 | 0.015 | 0.453 | 0.87 |
| LOC646226 | 105.09 | 7.91 | 120.27 | 10.44 | 5.44 | 0.032 | 0.445 | 0.87 |
| H2AFB1 | 93.29 | 0.91 | 106.77 | 4.36 | 5.50 | 0.031 | 0.446 | 0.87 |
| RNF150 | 93.20 | 2.70 | 106.68 | 2.52 | 6.98 | 0.020 | 0.448 | 0.87 |
| CES4 | 100.62 | 4.97 | 115.18 | 7.79 | 4.39 | 0.048 | 0.451 | 0.87 |
| LOC643495 | 106.08 | 5.63 | 121.49 | 5.11 | 4.88 | 0.039 | 0.444 | 0.87 |
| TIRAP | 126.88 | 1.79 | 145.32 | 6.34 | 4.83 | 0.040 | 0.444 | 0.87 |
| HS.122007 | 98.60 | 8.35 | 112.95 | 4.08 | 5.79 | 0.029 | 0.444 | 0.87 |
| LOC100132891 | 103.64 | 4.04 | 118.73 | 3.18 | 5.17 | 0.035 | 0.447 | 0.87 |
| CTBS | 159.58 | 8.69 | 182.82 | 7.87 | 13.48 | 0.005 | 0.443 | 0.87 |
| HOPX | 97.57 | 0.58 | 111.79 | 5.80 | 4.48 | 0.046 | 0.449 | 0.87 |
| COL11A2 | 110.53 | 0.79 | 126.65 | 4.46 | 6.17 | 0.025 | 0.448 | 0.87 |
| LOC727735 | 109.61 | 5.77 | 125.61 | 6.49 | 6.63 | 0.022 | 0.453 | 0.87 |
| HS.276796 | 104.56 | 6.96 | 119.81 | 3.69 | 7.83 | 0.016 | 0.449 | 0.87 |
| LOC652882 | 97.60 | 3.58 | 111.85 | 7.93 | 5.35 | 0.033 | 0.446 | 0.87 |
| LOC653468 | 100.56 | 9.11 | 115.26 | 8.65 | 7.26 | 0.018 | 0.451 | 0.87 |
| HS.570958 | 97.42 | 4.40 | 111.66 | 2.66 | 4.35 | 0.049 | 0.452 | 0.87 |
| LOC649975 | 102.93 | 8.01 | 117.98 | 10.28 | 5.29 | 0.034 | 0.447 | 0.87 |
| SLC27A6 | 112.06 | 9.80 | 128.46 | 6.03 | 5.96 | 0.027 | 0.448 | 0.87 |
| NFE2 | 101.30 | 13.07 | 116.15 | 16.97 | 4.67 | 0.043 | 0.447 | 0.87 |
| LOC641699 | 108.55 | 2.02 | 124.47 | 4.65 | 8.96 | 0.012 | 0.454 | 0.87 |
| AP2M1 | 410.85 | 61.56 | 471.14 | 76.99 | 6.74 | 0.021 | 0.449 | 0.87 |
| IFNE1 | 103.48 | 6.74 | 118.71 | 7.63 | 6.11 | 0.026 | 0.450 | 0.87 |
| SLC22A16 | 97.35 | 3.45 | 111.68 | 6.46 | 5.27 | 0.034 | 0.448 | 0.87 |
| ADAMTSL4 | 119.92 | 0.93 | 137.59 | 5.04 | 6.01 | 0.027 | 0.447 | 0.87 |
| LOC643936 | 102.86 | 2.87 | 118.03 | 4.18 | 4.83 | 0.040 | 0.444 | 0.87 |
| LOC100134147 | 112.57 | 2.04 | 129.18 | 6.23 | 5.08 | 0.037 | 0.447 | 0.87 |
| MMP23A | 106.76 | 4.95 | 122.53 | 4.01 | 29.07 | 0.001 | 0.447 | 0.87 |
| C9ORF102 | 183.33 | 47.64 | 210.41 | 48.68 | 6.77 | 0.021 | 0.448 | 0.87 |
| LMAN2 | 146.47 | 8.33 | 168.13 | 9.10 | 5.88 | 0.028 | 0.446 | 0.87 |
| STMN1 | 116.92 | 2.54 | 134.25 | 3.42 | 7.31 | 0.018 | 0.450 | 0.87 |
| FAM149A | 105.78 | 5.67 | 121.46 | 4.66 | 25.06 | 0.002 | 0.432 | 0.87 |
| AFAP1 | 128.52 | 6.12 | 147.61 | 6.37 | 24.86 | 0.002 | 0.431 | 0.87 |
| LOC100130987 | 105.72 | 2.50 | 121.46 | 3.59 | 5.81 | 0.028 | 0.444 | 0.87 |
| LOC345537 | 103.52 | 5.43 | 118.93 | 1.93 | 7.59 | 0.017 | 0.445 | 0.87 |
| CXORF50 | 103.93 | 2.59 | 119.40 | 4.29 | 13.21 | 0.006 | 0.446 | 0.87 |
| NFAT5 | 94.51 | 5.00 | 108.60 | 4.35 | 5.35 | 0.033 | 0.446 | 0.87 |
| LOC100131315 | 102.90 | 2.28 | 118.25 | 3.90 | 14.62 | 0.005 | 0.442 | 0.87 |
| HS.317051 | 109.00 | 3.60 | 125.29 | 1.64 | 14.36 | 0.005 | 0.445 | 0.87 |
| LOC401076 | 101.64 | 5.38 | 116.84 | 7.96 | 8.69 | 0.013 | 0.453 | 0.87 |
| LOC645447 | 107.70 | 7.68 | 123.84 | 13.52 | 4.65 | 0.043 | 0.447 | 0.87 |
| ALDH1A3 | 111.55 | 6.47 | 128.29 | 7.61 | 10.62 | 0.009 | 0.453 | 0.87 |
| OR52E5 | 106.33 | 12.10 | 122.30 | 15.85 | 6.32 | 0.024 | 0.452 | 0.87 |
| IGFALS | 106.11 | 10.59 | 122.05 | 7.27 | 5.90 | 0.028 | 0.447 | 0.87 |
| OR1S1 | 100.26 | 6.76 | 115.34 | 5.55 | 10.93 | 0.008 | 0.456 | 0.87 |
| FUBP3 | 99.61 | 3.21 | 114.62 | 7.25 | 4.42 | 0.048 | 0.450 | 0.87 |
| LOC727874 | 94.42 | 4.79 | 108.66 | 7.66 | 5.10 | 0.036 | 0.446 | 0.87 |
| MIR579 | 108.49 | 12.35 | 124.85 | 17.72 | 5.24 | 0.035 | 0.447 | 0.87 |
| LOC650257 | 109.39 | 5.27 | 125.89 | 6.96 | 13.00 | 0.006 | 0.442 | 0.87 |
| HS.545364 | 120.65 | 4.55 | 138.87 | 4.19 | 37.33 | 0.001 | 0.513 | 0.87 |
| ZNF540 | 101.67 | 5.05 | 117.05 | 2.61 | 4.77 | 0.041 | 0.444 | 0.87 |
| MBTPS1 | 163.84 | 15.90 | 188.72 | 14.40 | 7.91 | 0.016 | 0.451 | 0.87 |
| RIN1 | 128.82 | 9.59 | 148.39 | 6.90 | 4.45 | 0.047 | 0.450 | 0.87 |
| FLJ44635 | 102.87 | 3.73 | 118.50 | 3.11 | 13.75 | 0.005 | 0.445 | 0.87 |
| RAB43 | 111.85 | 11.68 | 128.85 | 10.80 | 6.37 | 0.024 | 0.453 | 0.87 |
| HS.581341 | 103.44 | 3.07 | 119.16 | 4.56 | 5.22 | 0.035 | 0.447 | 0.87 |
| GAPDHS | 102.28 | 2.97 | 117.83 | 4.93 | 10.28 | 0.009 | 0.453 | 0.87 |
| LOC653748 | 101.81 | 4.05 | 117.30 | 8.79 | 4.75 | 0.042 | 0.444 | 0.87 |
| KLF11 | 141.53 | 6.70 | 163.09 | 1.57 | 5.91 | 0.027 | 0.448 | 0.87 |
| IFNA16 | 98.51 | 9.90 | 113.52 | 6.97 | 6.72 | 0.021 | 0.450 | 0.87 |
| KIR2DS1 | 98.65 | 3.19 | 113.69 | 1.20 | 7.14 | 0.019 | 0.450 | 0.87 |
| HS.540572 | 101.31 | 4.81 | 116.77 | 2.22 | 6.31 | 0.024 | 0.451 | 0.87 |
| LOC652633 | 103.11 | 0.81 | 118.85 | 6.67 | 4.55 | 0.045 | 0.448 | 0.87 |
| HS.310040 | 111.88 | 7.95 | 128.97 | 9.82 | 4.98 | 0.038 | 0.447 | 0.87 |
| ZNF397 | 113.54 | 10.73 | 130.93 | 11.99 | 10.43 | 0.009 | 0.454 | 0.87 |
| LOC389517 | 109.66 | 1.55 | 126.47 | 3.23 | 16.68 | 0.004 | 0.453 | 0.87 |
| FAM150B | 108.53 | 5.49 | 125.17 | 2.11 | 4.69 | 0.043 | 0.447 | 0.87 |
| LOC391241 | 109.09 | 3.39 | 125.84 | 8.74 | 4.78 | 0.041 | 0.444 | 0.87 |
| FNDC5 | 103.24 | 4.81 | 119.10 | 4.95 | 10.38 | 0.009 | 0.454 | 0.87 |
| LY6G6E | 105.81 | 3.36 | 122.08 | 3.08 | 15.92 | 0.004 | 0.453 | 0.87 |
| ZNF483 | 109.16 | 1.95 | 125.94 | 2.65 | 6.54 | 0.023 | 0.454 | 0.87 |
| CACNB2 | 102.99 | 6.57 | 118.82 | 8.12 | 17.67 | 0.003 | 0.446 | 0.87 |
| LOC100133144 | 114.06 | 3.98 | 131.61 | 6.00 | 10.23 | 0.009 | 0.453 | 0.87 |
| GLT25D1 | 1056.83 | 257.84 | 1219.56 | 274.42 | 5.86 | 0.028 | 0.446 | 0.87 |
| HS.539244 | 108.40 | 12.10 | 125.10 | 9.85 | 4.44 | 0.047 | 0.450 | 0.87 |
| HS.193406 | 122.39 | 4.67 | 141.30 | 8.36 | 5.32 | 0.034 | 0.447 | 0.87 |
| PJA2 | 2174.11 | 827.80 | 2510.14 | 858.28 | 9.50 | 0.011 | 0.464 | 0.87 |
| LOC100128096 | 109.10 | 4.57 | 125.96 | 7.45 | 5.19 | 0.035 | 0.447 | 0.87 |
| HS.72488 | 114.90 | 12.94 | 132.67 | 16.44 | 4.32 | 0.050 | 0.452 | 0.87 |
| LMO4 | 1001.81 | 510.33 | 1156.83 | 505.38 | 12.33 | 0.007 | 0.440 | 0.87 |
| LOC440292 | 103.80 | 6.17 | 119.86 | 10.19 | 5.05 | 0.037 | 0.447 | 0.87 |
| OAS2 | 1461.93 | 342.91 | 1688.31 | 347.40 | 5.92 | 0.027 | 0.448 | 0.87 |
| LOC100134396 | 118.28 | 5.72 | 136.60 | 6.82 | 4.67 | 0.043 | 0.447 | 0.87 |
| LOC441666 | 107.20 | 10.57 | 123.80 | 7.84 | 8.27 | 0.014 | 0.452 | 0.87 |
| MAML1 | 104.26 | 5.67 | 120.43 | 4.72 | 13.89 | 0.005 | 0.449 | 0.87 |
| HS.283139 | 114.88 | 7.82 | 132.70 | 6.58 | 4.94 | 0.039 | 0.445 | 0.87 |
| CHI3L1 | 108.67 | 2.00 | 125.53 | 6.35 | 5.15 | 0.036 | 0.446 | 0.87 |
| SLCO1C1 | 111.66 | 10.16 | 128.99 | 4.23 | 4.90 | 0.039 | 0.444 | 0.87 |
| NFE2L2 | 1476.14 | 660.26 | 1705.80 | 579.26 | 4.90 | 0.039 | 0.444 | 0.87 |
| PYHIN1 | 123.62 | 16.02 | 142.86 | 15.36 | 9.31 | 0.011 | 0.462 | 0.87 |
| HS.541025 | 107.48 | 8.40 | 124.20 | 10.57 | 8.10 | 0.015 | 0.452 | 0.87 |
| HMGB3L1 | 115.06 | 9.88 | 132.97 | 8.52 | 10.05 | 0.010 | 0.453 | 0.87 |
| HS.562118 | 130.40 | 14.01 | 150.70 | 7.05 | 4.64 | 0.044 | 0.447 | 0.87 |
| FBP2 | 103.76 | 7.84 | 119.93 | 2.40 | 4.75 | 0.042 | 0.444 | 0.87 |
| C1QTNF7 | 109.80 | 10.07 | 126.96 | 9.42 | 35.10 | 0.001 | 0.525 | 0.86 |
| GALNT9 | 104.05 | 10.01 | 120.32 | 11.91 | 6.59 | 0.022 | 0.453 | 0.86 |
| HS.583921 | 117.25 | 8.94 | 135.61 | 6.02 | 4.36 | 0.049 | 0.452 | 0.86 |
| BPTF | 122.34 | 13.04 | 141.50 | 17.21 | 7.71 | 0.016 | 0.449 | 0.86 |
| LOC652637 | 106.37 | 0.71 | 123.03 | 4.79 | 6.83 | 0.021 | 0.448 | 0.86 |
| HRG | 106.78 | 6.51 | 123.51 | 6.33 | 4.40 | 0.048 | 0.451 | 0.86 |
| HS.377257 | 117.95 | 2.76 | 136.44 | 3.03 | 5.54 | 0.031 | 0.445 | 0.86 |
| LOC653610 | 140.18 | 8.25 | 162.18 | 11.74 | 6.01 | 0.027 | 0.447 | 0.86 |
| FLJ42986 | 101.71 | 7.61 | 117.70 | 12.71 | 4.35 | 0.049 | 0.452 | 0.86 |
| HS.544149 | 105.49 | 4.02 | 122.09 | 7.29 | 5.30 | 0.034 | 0.447 | 0.86 |
| LOC100129186 | 128.75 | 15.07 | 149.02 | 18.52 | 9.58 | 0.011 | 0.463 | 0.86 |
| C3ORF71 | 106.80 | 4.24 | 123.62 | 5.52 | 4.49 | 0.046 | 0.449 | 0.86 |
| LOC152663 | 107.29 | 12.60 | 124.20 | 10.55 | 7.48 | 0.017 | 0.448 | 0.86 |
| LOC440040 | 105.24 | 3.40 | 121.84 | 4.51 | 25.46 | 0.002 | 0.436 | 0.86 |
| ERG | 91.69 | 0.40 | 106.16 | 4.86 | 4.85 | 0.040 | 0.444 | 0.86 |
| INSRR | 116.45 | 3.03 | 134.82 | 2.62 | 6.61 | 0.022 | 0.453 | 0.86 |
| HS.44650 | 105.80 | 1.94 | 122.53 | 8.08 | 4.59 | 0.044 | 0.447 | 0.86 |
| TMEM18 | 123.00 | 21.14 | 142.47 | 17.53 | 5.23 | 0.035 | 0.447 | 0.86 |
| FRAT1 | 113.26 | 11.04 | 131.19 | 7.60 | 7.37 | 0.018 | 0.449 | 0.86 |
| LOC100128747 | 110.91 | 5.84 | 128.54 | 9.51 | 7.78 | 0.016 | 0.448 | 0.86 |
| LOC653496 | 176.91 | 25.34 | 205.07 | 25.72 | 61.10 | 0.000 | 0.744 | 0.86 |
| LOC644421 | 116.27 | 5.30 | 134.78 | 9.03 | 6.44 | 0.023 | 0.453 | 0.86 |
| HS.537922 | 106.65 | 6.54 | 123.66 | 7.41 | 5.14 | 0.036 | 0.446 | 0.86 |
| FLJ13197 | 105.39 | 6.42 | 122.21 | 2.98 | 4.84 | 0.040 | 0.444 | 0.86 |
| WHDC1L1 | 103.59 | 3.06 | 120.12 | 3.01 | 6.78 | 0.021 | 0.449 | 0.86 |
| HS.551145 | 108.97 | 4.98 | 126.36 | 3.91 | 7.16 | 0.019 | 0.451 | 0.86 |
| LOC200030 | 115.00 | 8.03 | 133.39 | 8.22 | 17.02 | 0.003 | 0.453 | 0.86 |
| FAM71E2 | 106.56 | 3.58 | 123.59 | 2.93 | 5.26 | 0.034 | 0.448 | 0.86 |
| MKL2 | 134.70 | 24.08 | 156.25 | 19.79 | 4.56 | 0.045 | 0.448 | 0.86 |
| VENTXP7 | 107.97 | 5.74 | 125.28 | 7.81 | 10.74 | 0.009 | 0.453 | 0.86 |
| C19ORF38 | 113.92 | 10.40 | 132.21 | 14.17 | 6.52 | 0.023 | 0.453 | 0.86 |
| LOC651198 | 147.73 | 23.63 | 171.47 | 16.94 | 6.15 | 0.025 | 0.448 | 0.86 |
| LOC730883 | 106.27 | 4.76 | 123.35 | 6.97 | 9.63 | 0.011 | 0.463 | 0.86 |
| HS.545587 | 115.85 | 10.58 | 134.48 | 7.85 | 4.62 | 0.044 | 0.447 | 0.86 |
| PSPHL | 109.17 | 3.22 | 126.73 | 3.54 | 5.60 | 0.030 | 0.445 | 0.86 |
| LOC643246 | 111.96 | 7.20 | 130.07 | 9.55 | 6.38 | 0.024 | 0.453 | 0.86 |
| OR11H12 | 103.55 | 7.27 | 120.32 | 1.65 | 5.16 | 0.036 | 0.446 | 0.86 |
| LOC391761 | 102.52 | 3.40 | 119.15 | 6.83 | 5.44 | 0.032 | 0.444 | 0.86 |
| HS.9123 | 117.74 | 12.05 | 136.88 | 14.96 | 4.40 | 0.048 | 0.450 | 0.86 |
| LOC100132112 | 124.03 | 4.73 | 144.19 | 5.42 | 4.51 | 0.046 | 0.449 | 0.86 |
| GGTLC1 | 111.99 | 9.08 | 130.20 | 4.05 | 6.18 | 0.025 | 0.449 | 0.86 |
| APOC3 | 103.22 | 0.78 | 120.00 | 0.63 | 28.18 | 0.001 | 0.454 | 0.86 |
| LOC728162 | 102.55 | 5.17 | 119.24 | 9.18 | 6.98 | 0.020 | 0.448 | 0.86 |
| LOC651537 | 102.06 | 6.21 | 118.69 | 6.30 | 27.71 | 0.001 | 0.462 | 0.86 |
| VSTM1 | 102.23 | 10.36 | 118.91 | 6.22 | 6.94 | 0.020 | 0.447 | 0.86 |
| UGT2B7 | 118.85 | 17.42 | 138.25 | 14.53 | 5.43 | 0.032 | 0.445 | 0.86 |
| HS.563019 | 109.93 | 7.16 | 127.88 | 13.58 | 4.33 | 0.049 | 0.452 | 0.86 |
| PKLR | 112.09 | 6.99 | 130.40 | 2.90 | 6.23 | 0.025 | 0.448 | 0.86 |
| HS.527657 | 124.64 | 10.56 | 145.00 | 12.69 | 8.60 | 0.013 | 0.451 | 0.86 |
| VN1R5 | 109.01 | 3.47 | 126.86 | 4.65 | 6.85 | 0.021 | 0.447 | 0.86 |
| HS.477876 | 103.61 | 3.09 | 120.57 | 6.59 | 8.31 | 0.014 | 0.452 | 0.86 |
| LOC652715 | 102.76 | 19.73 | 119.59 | 16.34 | 8.19 | 0.015 | 0.452 | 0.86 |
| SNORD89 | 127.03 | 3.57 | 147.87 | 5.81 | 6.38 | 0.024 | 0.452 | 0.86 |
| LOC646537 | 97.65 | 5.13 | 113.69 | 5.71 | 8.19 | 0.015 | 0.453 | 0.86 |
| ZNF204 | 94.36 | 3.53 | 109.87 | 0.43 | 7.71 | 0.016 | 0.448 | 0.86 |
| LOC100134584 | 139.95 | 4.87 | 162.95 | 3.30 | 24.02 | 0.002 | 0.437 | 0.86 |
| HS.164401 | 103.88 | 6.11 | 120.97 | 12.34 | 4.41 | 0.048 | 0.450 | 0.86 |
| PRB1 | 106.65 | 12.24 | 124.20 | 14.90 | 11.02 | 0.008 | 0.456 | 0.86 |
| SPAG9 | 408.15 | 125.37 | 475.34 | 125.59 | 20.93 | 0.002 | 0.444 | 0.86 |
| STX12 | 236.64 | 53.90 | 275.61 | 63.55 | 5.83 | 0.028 | 0.445 | 0.86 |
| TP73L | 103.02 | 6.59 | 119.99 | 8.19 | 15.35 | 0.004 | 0.451 | 0.86 |
| ZNF766 | 234.92 | 39.68 | 273.63 | 45.24 | 11.70 | 0.007 | 0.450 | 0.86 |
| HS.569199 | 107.21 | 6.29 | 124.90 | 12.85 | 4.58 | 0.045 | 0.448 | 0.86 |
| AADACL3 | 98.14 | 3.20 | 114.34 | 6.63 | 8.17 | 0.015 | 0.452 | 0.86 |
| LOC650454 | 106.32 | 5.85 | 123.88 | 11.28 | 5.60 | 0.030 | 0.445 | 0.86 |
| FLJ44005 | 106.12 | 6.39 | 123.66 | 3.36 | 8.74 | 0.013 | 0.455 | 0.86 |
| LOC730259 | 105.06 | 6.15 | 122.43 | 10.43 | 6.95 | 0.020 | 0.447 | 0.86 |
| HS.566751 | 115.07 | 3.63 | 134.11 | 3.26 | 6.38 | 0.024 | 0.453 | 0.86 |
| LOC554223 | 111.15 | 2.68 | 129.55 | 5.90 | 6.49 | 0.023 | 0.452 | 0.86 |
| DSC1 | 108.02 | 7.13 | 125.91 | 1.78 | 4.35 | 0.049 | 0.452 | 0.86 |
| LOC387647 | 123.02 | 1.54 | 143.41 | 4.06 | 6.35 | 0.024 | 0.452 | 0.86 |
| KCNH1 | 102.92 | 3.26 | 119.98 | 8.18 | 5.08 | 0.037 | 0.446 | 0.86 |
| TBX1 | 111.47 | 6.78 | 129.95 | 7.24 | 8.83 | 0.013 | 0.456 | 0.86 |
| TTTY23 | 96.95 | 5.97 | 113.04 | 7.69 | 10.24 | 0.009 | 0.453 | 0.86 |
| MITF | 107.02 | 4.84 | 124.80 | 6.34 | 10.73 | 0.009 | 0.454 | 0.86 |
| LOC651311 | 96.24 | 3.80 | 112.24 | 2.74 | 19.65 | 0.003 | 0.459 | 0.86 |
| C20ORF24 | 5618.41 | 1871.91 | 6554.99 | 2099.79 | 4.48 | 0.046 | 0.448 | 0.86 |
| TXNDC11 | 250.65 | 42.31 | 292.46 | 52.03 | 5.90 | 0.028 | 0.447 | 0.86 |
| LAX1 | 178.36 | 33.10 | 208.13 | 29.61 | 11.62 | 0.007 | 0.450 | 0.86 |
| TRIM21 | 836.46 | 222.38 | 976.29 | 199.11 | 5.41 | 0.033 | 0.445 | 0.86 |
| DAO | 115.28 | 8.71 | 134.56 | 1.96 | 4.51 | 0.046 | 0.448 | 0.86 |
| LOC100133403 | 103.19 | 0.50 | 120.46 | 2.02 | 13.86 | 0.005 | 0.450 | 0.86 |
| ATP10D | 103.41 | 7.86 | 120.73 | 3.23 | 6.36 | 0.024 | 0.452 | 0.86 |
| HS.526528 | 111.39 | 9.52 | 130.06 | 9.67 | 17.16 | 0.003 | 0.455 | 0.86 |
| LOC728217 | 96.38 | 2.42 | 112.54 | 4.95 | 5.76 | 0.029 | 0.445 | 0.86 |
| SLC35A5 | 357.20 | 87.77 | 417.14 | 104.43 | 5.22 | 0.035 | 0.446 | 0.86 |
| HAAO | 119.09 | 1.99 | 139.08 | 9.60 | 4.51 | 0.046 | 0.449 | 0.86 |
| PKD1 | 103.90 | 5.45 | 121.41 | 3.10 | 4.49 | 0.046 | 0.449 | 0.86 |
| KIF1B | 668.40 | 312.96 | 781.19 | 347.77 | 4.86 | 0.040 | 0.445 | 0.86 |
| LOC649212 | 98.12 | 3.54 | 114.67 | 4.75 | 11.36 | 0.008 | 0.451 | 0.86 |
| FRMPD2L1 | 97.80 | 10.14 | 114.31 | 10.38 | 4.65 | 0.043 | 0.447 | 0.86 |
| LOC732393 | 102.87 | 9.11 | 120.28 | 13.44 | 4.57 | 0.045 | 0.448 | 0.86 |
| SCARNA5 | 103.88 | 7.52 | 121.47 | 10.38 | 6.12 | 0.026 | 0.450 | 0.86 |
| RHBDD1 | 218.09 | 25.42 | 255.10 | 33.85 | 6.00 | 0.027 | 0.448 | 0.85 |
| LOC731542 | 133.64 | 12.85 | 156.32 | 14.69 | 4.80 | 0.041 | 0.444 | 0.85 |
| FLJ46284 | 109.82 | 2.24 | 128.46 | 7.04 | 5.79 | 0.029 | 0.444 | 0.85 |
| KIAA1908 | 116.98 | 5.97 | 136.89 | 6.45 | 40.69 | 0.001 | 0.559 | 0.85 |
| OR52H1 | 92.30 | 4.39 | 108.02 | 10.10 | 4.49 | 0.046 | 0.448 | 0.85 |
| LOC642236 | 110.31 | 5.66 | 129.10 | 3.17 | 5.32 | 0.034 | 0.447 | 0.85 |
| XKR5 | 98.02 | 10.19 | 114.72 | 12.17 | 7.85 | 0.016 | 0.450 | 0.85 |
| HS.543489 | 117.09 | 9.98 | 137.04 | 7.55 | 4.65 | 0.043 | 0.447 | 0.85 |
| LOC649680 | 109.62 | 10.85 | 128.31 | 16.68 | 5.04 | 0.037 | 0.447 | 0.85 |
| OR5J2 | 98.89 | 5.73 | 115.77 | 3.82 | 12.89 | 0.006 | 0.439 | 0.85 |
| LOC100134108 | 204.92 | 44.18 | 239.95 | 38.38 | 7.38 | 0.018 | 0.449 | 0.85 |
| FBXL3 | 101.88 | 8.26 | 119.31 | 3.63 | 4.36 | 0.049 | 0.451 | 0.85 |
| IER5L | 109.84 | 10.66 | 128.64 | 6.62 | 7.15 | 0.019 | 0.450 | 0.85 |
| DYDC1 | 102.03 | 12.71 | 119.51 | 7.07 | 4.71 | 0.042 | 0.446 | 0.85 |
| HS.513455 | 108.15 | 15.23 | 126.68 | 17.91 | 11.93 | 0.007 | 0.444 | 0.85 |
| LOC654116 | 117.90 | 12.57 | 138.12 | 19.07 | 5.39 | 0.033 | 0.445 | 0.85 |
| FAM63A | 114.27 | 1.79 | 133.91 | 4.13 | 6.44 | 0.023 | 0.453 | 0.85 |
| LOC647135 | 105.95 | 7.23 | 124.16 | 13.60 | 4.51 | 0.046 | 0.449 | 0.85 |
| LOC100128028 | 98.33 | 4.30 | 115.26 | 10.12 | 4.84 | 0.040 | 0.444 | 0.85 |
| LOC440132 | 96.12 | 5.55 | 112.68 | 10.74 | 4.92 | 0.039 | 0.445 | 0.85 |
| LOC647681 | 108.34 | 7.53 | 127.01 | 2.76 | 4.32 | 0.050 | 0.452 | 0.85 |
| LOC651991 | 100.87 | 3.39 | 118.26 | 8.86 | 5.38 | 0.033 | 0.445 | 0.85 |
| LOC645676 | 114.54 | 3.21 | 134.31 | 7.29 | 5.10 | 0.036 | 0.446 | 0.85 |
| KLHDC1 | 103.03 | 3.73 | 120.85 | 2.97 | 39.60 | 0.001 | 0.558 | 0.85 |
| LOC727832 | 116.33 | 14.55 | 136.45 | 19.78 | 6.64 | 0.022 | 0.452 | 0.85 |
| PHF14 | 119.21 | 3.33 | 139.84 | 8.51 | 6.19 | 0.025 | 0.449 | 0.85 |
| LOC100129907 | 133.25 | 4.61 | 156.31 | 11.06 | 4.65 | 0.043 | 0.447 | 0.85 |
| OR4F16 | 104.22 | 5.50 | 122.26 | 4.29 | 13.45 | 0.005 | 0.443 | 0.85 |
| LOC647346 | 102.64 | 10.45 | 120.42 | 10.26 | 11.09 | 0.008 | 0.458 | 0.85 |
| SAV1 | 171.84 | 20.09 | 201.65 | 23.93 | 4.33 | 0.049 | 0.452 | 0.85 |
| LOC100131767 | 128.81 | 7.03 | 151.19 | 13.64 | 4.56 | 0.045 | 0.448 | 0.85 |
| DHRS4L1 | 112.11 | 9.95 | 131.58 | 16.30 | 4.99 | 0.038 | 0.447 | 0.85 |
| LOC339879 | 103.57 | 8.31 | 121.57 | 15.19 | 4.31 | 0.050 | 0.452 | 0.85 |
| LOC441711 | 103.89 | 1.65 | 121.95 | 6.44 | 6.53 | 0.023 | 0.453 | 0.85 |
| CASS4 | 95.74 | 5.32 | 112.40 | 9.67 | 5.86 | 0.028 | 0.445 | 0.85 |
| ADAM3A | 100.25 | 9.56 | 117.69 | 14.31 | 6.02 | 0.026 | 0.448 | 0.85 |
| TAAR9 | 104.46 | 6.23 | 122.65 | 11.77 | 5.12 | 0.036 | 0.447 | 0.85 |
| LOC644962 | 100.52 | 4.49 | 118.03 | 5.74 | 9.73 | 0.010 | 0.459 | 0.85 |
| FTSJD2 | 370.88 | 119.72 | 435.52 | 117.36 | 10.64 | 0.009 | 0.453 | 0.85 |
| TMEM164 | 129.51 | 5.08 | 152.11 | 4.17 | 10.50 | 0.009 | 0.455 | 0.85 |
| VGLL2 | 109.78 | 3.70 | 128.94 | 6.83 | 4.35 | 0.049 | 0.452 | 0.85 |
| BCHE | 105.19 | 11.21 | 123.55 | 13.84 | 5.83 | 0.028 | 0.445 | 0.85 |
| MYO18A | 194.60 | 49.29 | 228.64 | 38.19 | 5.27 | 0.034 | 0.448 | 0.85 |
| LOC100133572 | 103.10 | 7.65 | 121.15 | 6.98 | 14.66 | 0.005 | 0.444 | 0.85 |
| DAZ4 | 89.78 | 6.43 | 105.51 | 6.36 | 5.94 | 0.027 | 0.447 | 0.85 |
| HS.574595 | 112.62 | 5.23 | 132.35 | 4.45 | 14.59 | 0.005 | 0.443 | 0.85 |
| FAS | 134.07 | 8.50 | 157.58 | 8.54 | 7.41 | 0.018 | 0.448 | 0.85 |
| RNF114 | 530.60 | 130.62 | 623.67 | 142.79 | 12.89 | 0.006 | 0.438 | 0.85 |
| LOC648927 | 118.69 | 9.46 | 139.51 | 13.59 | 8.66 | 0.013 | 0.450 | 0.85 |
| ZNF238 | 112.84 | 1.72 | 132.64 | 7.31 | 4.49 | 0.046 | 0.448 | 0.85 |
| SOX13 | 121.40 | 5.14 | 142.71 | 3.99 | 7.88 | 0.016 | 0.450 | 0.85 |
| DNAH11 | 101.14 | 8.25 | 118.95 | 11.45 | 5.25 | 0.034 | 0.447 | 0.85 |
| SLC18A1 | 106.89 | 4.27 | 125.72 | 6.59 | 7.40 | 0.018 | 0.449 | 0.85 |
| LOC649952 | 106.31 | 9.82 | 125.04 | 4.98 | 5.92 | 0.027 | 0.448 | 0.85 |
| SCRN1 | 281.71 | 84.92 | 331.38 | 79.83 | 5.49 | 0.032 | 0.446 | 0.85 |
| ADCY4 | 99.26 | 13.28 | 116.78 | 17.99 | 4.54 | 0.045 | 0.449 | 0.85 |
| LOC654252 | 113.08 | 7.00 | 133.05 | 11.71 | 5.06 | 0.037 | 0.447 | 0.85 |
| FLJ38773 | 105.53 | 4.20 | 124.18 | 7.61 | 5.13 | 0.036 | 0.447 | 0.85 |
| SIRT2 | 174.24 | 23.34 | 205.04 | 34.10 | 4.80 | 0.041 | 0.444 | 0.85 |
| CORO2A | 165.89 | 29.09 | 195.25 | 28.05 | 6.14 | 0.026 | 0.449 | 0.85 |
| FBXW10 | 111.72 | 6.68 | 131.51 | 7.54 | 4.63 | 0.044 | 0.447 | 0.85 |
| ADCK5 | 138.70 | 17.74 | 163.27 | 26.18 | 4.68 | 0.043 | 0.447 | 0.85 |
| GBA2 | 183.44 | 26.76 | 216.00 | 34.01 | 6.36 | 0.024 | 0.452 | 0.85 |
| HS.171274 | 111.08 | 4.60 | 130.80 | 7.86 | 5.26 | 0.034 | 0.448 | 0.85 |
| C8ORF79 | 110.94 | 7.73 | 130.66 | 3.97 | 8.05 | 0.015 | 0.453 | 0.85 |
| CCDC42 | 109.74 | 7.04 | 129.24 | 11.14 | 8.08 | 0.015 | 0.453 | 0.85 |
| LOC653155 | 103.95 | 3.05 | 122.43 | 3.76 | 9.84 | 0.010 | 0.457 | 0.85 |
| LOC646720 | 103.81 | 8.77 | 122.27 | 9.67 | 17.25 | 0.003 | 0.454 | 0.85 |
| TFEC | 115.68 | 8.44 | 136.26 | 9.14 | 49.36 | 0.000 | 0.587 | 0.85 |
| IRX2 | 106.93 | 4.50 | 125.96 | 6.16 | 16.17 | 0.004 | 0.458 | 0.85 |
| TSHZ1 | 175.07 | 15.50 | 206.24 | 8.12 | 4.78 | 0.041 | 0.444 | 0.85 |
| FLJ37786 | 113.81 | 3.83 | 134.08 | 9.73 | 5.95 | 0.027 | 0.448 | 0.85 |
| HS.554056 | 102.31 | 8.06 | 120.52 | 8.53 | 17.66 | 0.003 | 0.445 | 0.85 |
| CPPED1 | 117.96 | 11.25 | 138.98 | 14.75 | 4.93 | 0.039 | 0.445 | 0.85 |
| BLOC1S2 | 108.94 | 9.63 | 128.36 | 13.44 | 5.70 | 0.029 | 0.445 | 0.85 |
| CDCP1 | 113.37 | 7.98 | 133.59 | 4.57 | 5.94 | 0.027 | 0.447 | 0.85 |
| HIST2H2BF | 114.56 | 5.27 | 135.01 | 1.87 | 9.66 | 0.011 | 0.462 | 0.85 |
| PQLC1 | 1638.67 | 609.61 | 1931.39 | 717.40 | 4.65 | 0.043 | 0.448 | 0.85 |
| HS.544576 | 117.15 | 10.95 | 138.08 | 4.36 | 5.38 | 0.033 | 0.445 | 0.85 |
| LOC646845 | 104.62 | 9.68 | 123.33 | 11.59 | 6.78 | 0.021 | 0.449 | 0.85 |
| IGBP1 | 480.16 | 165.80 | 566.06 | 166.04 | 7.16 | 0.019 | 0.450 | 0.85 |
| LOC100130291 | 208.98 | 72.76 | 246.44 | 79.91 | 6.25 | 0.025 | 0.449 | 0.85 |
| MIR135B | 107.24 | 3.53 | 126.47 | 7.34 | 8.67 | 0.013 | 0.451 | 0.85 |
| TIAM2 | 111.31 | 7.48 | 131.30 | 11.64 | 5.21 | 0.035 | 0.447 | 0.85 |
| PTPRE | 93.49 | 3.29 | 110.28 | 7.95 | 6.24 | 0.025 | 0.449 | 0.85 |
| HS.546710 | 103.96 | 6.64 | 122.64 | 6.82 | 7.15 | 0.019 | 0.451 | 0.85 |
| IFRD1 | 114.26 | 10.53 | 134.81 | 11.17 | 5.53 | 0.031 | 0.444 | 0.85 |
| LOC100128691 | 110.71 | 9.98 | 130.64 | 8.09 | 7.40 | 0.018 | 0.449 | 0.85 |
| PRKD2 | 234.56 | 36.30 | 276.85 | 31.77 | 10.38 | 0.009 | 0.454 | 0.85 |
| ZNF611 | 124.53 | 7.94 | 146.99 | 4.16 | 4.53 | 0.045 | 0.449 | 0.85 |
| SPATA13 | 171.41 | 62.82 | 202.37 | 74.97 | 4.37 | 0.049 | 0.451 | 0.85 |
| LOC100129571 | 118.06 | 2.37 | 139.41 | 3.66 | 7.41 | 0.018 | 0.449 | 0.85 |
| CBX3 | 142.75 | 25.28 | 168.57 | 28.80 | 8.82 | 0.013 | 0.456 | 0.85 |
| ZNF785 | 120.29 | 2.58 | 142.07 | 2.23 | 8.71 | 0.013 | 0.453 | 0.85 |
| LOC100134654 | 98.85 | 6.75 | 116.75 | 9.02 | 7.79 | 0.016 | 0.449 | 0.85 |
| H6PD | 126.37 | 2.48 | 149.25 | 2.18 | 14.03 | 0.005 | 0.450 | 0.85 |
| ARSB | 130.35 | 17.00 | 153.96 | 11.31 | 4.78 | 0.041 | 0.444 | 0.85 |
| HS.539114 | 109.36 | 3.50 | 129.20 | 6.09 | 5.83 | 0.028 | 0.445 | 0.85 |
| LOC391746 | 107.49 | 0.61 | 127.02 | 4.96 | 6.26 | 0.025 | 0.450 | 0.85 |
| LOC649923 | 133.75 | 19.84 | 158.05 | 17.60 | 5.68 | 0.030 | 0.447 | 0.85 |
| MUL1 | 605.11 | 229.00 | 715.14 | 256.17 | 5.38 | 0.033 | 0.445 | 0.85 |
| LOC728773 | 105.07 | 5.32 | 124.19 | 11.64 | 4.98 | 0.038 | 0.447 | 0.85 |
| FLJ20021 | 166.50 | 24.71 | 196.87 | 24.25 | 29.37 | 0.001 | 0.456 | 0.85 |
| LOC729291 | 96.66 | 0.68 | 114.32 | 4.23 | 6.89 | 0.020 | 0.446 | 0.85 |
| C9ORF14 | 100.53 | 7.48 | 118.91 | 9.51 | 14.52 | 0.005 | 0.444 | 0.85 |
| HAS2 | 102.48 | 5.22 | 121.24 | 3.89 | 7.04 | 0.020 | 0.449 | 0.85 |
| C2ORF67 | 105.88 | 9.71 | 125.32 | 11.38 | 13.44 | 0.005 | 0.443 | 0.84 |
| HS.574475 | 110.35 | 6.50 | 130.62 | 11.50 | 4.79 | 0.041 | 0.444 | 0.84 |
| GAS2L1 | 104.37 | 7.27 | 123.56 | 2.19 | 4.80 | 0.041 | 0.444 | 0.84 |
| LOC642730 | 98.94 | 9.43 | 117.13 | 7.71 | 13.59 | 0.005 | 0.446 | 0.84 |
| A26C3 | 107.20 | 6.63 | 126.92 | 13.15 | 4.75 | 0.042 | 0.444 | 0.84 |
| EAPP | 437.81 | 215.77 | 518.35 | 205.62 | 13.12 | 0.006 | 0.445 | 0.84 |
| LOC340888 | 100.74 | 8.49 | 119.31 | 12.01 | 9.14 | 0.012 | 0.457 | 0.84 |
| HS.400256 | 130.42 | 11.33 | 154.50 | 8.23 | 13.11 | 0.006 | 0.445 | 0.84 |
| LOC643424 | 107.11 | 8.21 | 126.89 | 11.52 | 9.86 | 0.010 | 0.457 | 0.84 |
| USP11 | 190.28 | 17.52 | 225.50 | 13.84 | 4.46 | 0.047 | 0.449 | 0.84 |
| TCTA | 187.06 | 2.99 | 221.72 | 8.02 | 8.18 | 0.015 | 0.453 | 0.84 |
| TJP1 | 118.10 | 8.86 | 140.00 | 7.87 | 11.57 | 0.007 | 0.447 | 0.84 |
| LOC100129343 | 108.60 | 4.34 | 128.75 | 3.65 | 4.37 | 0.049 | 0.451 | 0.84 |
| SLC22A18AS | 106.27 | 12.74 | 126.00 | 9.99 | 4.87 | 0.040 | 0.445 | 0.84 |
| LOC727815 | 107.53 | 11.20 | 127.52 | 11.96 | 8.07 | 0.015 | 0.453 | 0.84 |
| CCS | 167.08 | 17.92 | 198.19 | 12.92 | 8.73 | 0.013 | 0.455 | 0.84 |
| BCOR | 128.98 | 28.09 | 153.01 | 33.89 | 4.87 | 0.040 | 0.445 | 0.84 |
| MTMR3 | 111.94 | 4.31 | 132.80 | 8.67 | 6.04 | 0.026 | 0.448 | 0.84 |
| LOC100134435 | 100.32 | 4.49 | 119.05 | 0.72 | 7.91 | 0.016 | 0.452 | 0.84 |
| GPR125 | 111.12 | 7.07 | 131.90 | 13.96 | 5.06 | 0.037 | 0.447 | 0.84 |
| HS.100261 | 98.19 | 4.05 | 116.57 | 1.79 | 5.59 | 0.031 | 0.445 | 0.84 |
| NEURL1B | 159.63 | 22.10 | 189.51 | 23.75 | 5.03 | 0.037 | 0.447 | 0.84 |
| APLP2 | 132.18 | 12.40 | 156.94 | 7.86 | 7.41 | 0.018 | 0.449 | 0.84 |
| MIR486 | 101.89 | 7.86 | 120.98 | 2.62 | 5.75 | 0.029 | 0.445 | 0.84 |
| HS.582706 | 109.92 | 1.54 | 130.54 | 6.12 | 4.66 | 0.043 | 0.448 | 0.84 |
| LOC642083 | 104.43 | 11.73 | 124.02 | 12.47 | 5.14 | 0.036 | 0.446 | 0.84 |
| APOL1 | 111.00 | 3.00 | 131.86 | 5.11 | 15.42 | 0.004 | 0.452 | 0.84 |
| ATAD3C | 101.53 | 6.59 | 120.61 | 13.04 | 5.09 | 0.037 | 0.446 | 0.84 |
| CCL23 | 90.58 | 4.76 | 107.61 | 3.99 | 18.21 | 0.003 | 0.453 | 0.84 |
| NDUFS2 | 148.61 | 33.57 | 176.57 | 29.54 | 5.68 | 0.030 | 0.447 | 0.84 |
| LOC644677 | 101.80 | 5.03 | 121.00 | 4.38 | 8.70 | 0.013 | 0.452 | 0.84 |
| SRRM2 | 1591.02 | 381.55 | 1891.20 | 402.78 | 23.50 | 0.002 | 0.449 | 0.84 |
| LILRA5 | 116.24 | 6.07 | 138.17 | 10.35 | 8.83 | 0.013 | 0.456 | 0.84 |
| TMEM195 | 100.56 | 2.22 | 119.55 | 8.71 | 4.45 | 0.047 | 0.450 | 0.84 |
| ATRN | 113.85 | 18.04 | 135.40 | 16.56 | 4.32 | 0.050 | 0.452 | 0.84 |
| LIN7B | 114.27 | 9.01 | 135.90 | 8.34 | 5.50 | 0.031 | 0.446 | 0.84 |
| LOC285359 | 106.62 | 7.52 | 126.89 | 2.65 | 6.70 | 0.022 | 0.450 | 0.84 |
| TMEM19 | 124.88 | 11.38 | 148.65 | 12.28 | 9.95 | 0.010 | 0.456 | 0.84 |
| PDZRN3 | 101.69 | 2.17 | 121.07 | 7.13 | 4.85 | 0.040 | 0.444 | 0.84 |
| C1ORF152 | 96.67 | 1.70 | 115.10 | 4.04 | 8.10 | 0.015 | 0.452 | 0.84 |
| ZGPAT | 592.13 | 211.89 | 705.01 | 217.19 | 4.92 | 0.039 | 0.445 | 0.84 |
| RRAD | 105.11 | 2.45 | 125.17 | 0.64 | 12.68 | 0.006 | 0.439 | 0.84 |
| LOC100129979 | 103.63 | 5.02 | 123.42 | 3.15 | 11.69 | 0.007 | 0.449 | 0.84 |
| SAMD4A | 130.53 | 2.38 | 155.53 | 3.59 | 9.24 | 0.012 | 0.456 | 0.84 |
| HSPBL2 | 163.44 | 18.80 | 194.79 | 8.59 | 5.32 | 0.034 | 0.447 | 0.84 |
| AHNAK | 101.98 | 9.40 | 121.55 | 11.01 | 18.11 | 0.003 | 0.454 | 0.84 |
| BRUNOL4 | 102.28 | 3.97 | 121.95 | 3.10 | 8.97 | 0.012 | 0.453 | 0.84 |
| LOC100131132 | 108.90 | 7.97 | 129.86 | 3.75 | 5.15 | 0.036 | 0.446 | 0.84 |
| MUC12 | 103.07 | 10.53 | 122.92 | 17.36 | 5.02 | 0.037 | 0.447 | 0.84 |
| HS.505855 | 116.24 | 6.42 | 138.63 | 8.09 | 5.17 | 0.035 | 0.447 | 0.84 |
| LOC144776 | 108.70 | 8.88 | 129.67 | 13.68 | 7.32 | 0.018 | 0.450 | 0.84 |
| LPAR2 | 167.69 | 23.66 | 200.06 | 32.65 | 4.45 | 0.047 | 0.450 | 0.84 |
| LOC284757 | 96.78 | 8.58 | 115.46 | 6.28 | 7.44 | 0.018 | 0.449 | 0.84 |
| CD207 | 104.45 | 12.17 | 124.64 | 8.21 | 6.82 | 0.021 | 0.448 | 0.84 |
| PJA1 | 196.42 | 37.07 | 234.44 | 26.98 | 5.58 | 0.031 | 0.445 | 0.84 |
| LOC650898 | 126.40 | 28.09 | 150.87 | 21.93 | 6.76 | 0.021 | 0.448 | 0.84 |
| HS.581740 | 111.92 | 3.23 | 133.62 | 4.41 | 14.51 | 0.005 | 0.441 | 0.84 |
| LOC653264 | 122.34 | 7.03 | 146.12 | 1.52 | 4.82 | 0.040 | 0.445 | 0.84 |
| HS.539516 | 104.40 | 7.67 | 124.71 | 2.48 | 5.44 | 0.032 | 0.445 | 0.84 |
| NRBP1 | 168.65 | 7.46 | 201.51 | 4.56 | 11.39 | 0.008 | 0.452 | 0.84 |
| BTN2A2 | 144.55 | 17.36 | 172.74 | 17.78 | 11.81 | 0.007 | 0.446 | 0.84 |
| MAP3K3 | 163.72 | 21.02 | 195.69 | 29.34 | 6.02 | 0.027 | 0.448 | 0.84 |
| PLXND1 | 118.64 | 6.39 | 141.84 | 11.61 | 5.60 | 0.030 | 0.445 | 0.84 |
| ATRN | 103.07 | 8.29 | 123.26 | 9.10 | 23.35 | 0.002 | 0.453 | 0.84 |
| RGS17 | 122.66 | 7.27 | 146.71 | 3.06 | 9.34 | 0.011 | 0.463 | 0.84 |
| ANTXR1 | 98.88 | 5.43 | 118.28 | 1.22 | 6.39 | 0.024 | 0.453 | 0.84 |
| SOX6 | 100.65 | 5.65 | 120.41 | 7.71 | 5.37 | 0.033 | 0.445 | 0.84 |
| LOC100129213 | 106.17 | 6.48 | 127.02 | 2.96 | 9.44 | 0.011 | 0.465 | 0.84 |
| ZNF34 | 125.79 | 14.58 | 150.53 | 16.73 | 10.13 | 0.010 | 0.454 | 0.84 |
| HS.545338 | 105.72 | 9.69 | 126.53 | 13.77 | 6.76 | 0.021 | 0.448 | 0.84 |
| UBASH3A | 129.69 | 18.96 | 155.27 | 24.96 | 7.13 | 0.019 | 0.449 | 0.84 |
| LOC100130000 | 118.92 | 13.59 | 142.38 | 8.27 | 4.70 | 0.042 | 0.446 | 0.84 |
| UNC13D | 165.75 | 6.92 | 198.56 | 10.44 | 9.54 | 0.011 | 0.463 | 0.83 |
| LOC100132713 | 97.41 | 6.61 | 116.72 | 7.72 | 14.00 | 0.005 | 0.451 | 0.83 |
| LOC100130298 | 177.57 | 35.11 | 212.86 | 33.31 | 33.31 | 0.001 | 0.495 | 0.83 |
| ZNF285B | 109.06 | 8.18 | 130.76 | 5.13 | 12.35 | 0.006 | 0.443 | 0.83 |
| OR4K15 | 107.57 | 15.41 | 128.98 | 16.72 | 6.05 | 0.026 | 0.448 | 0.83 |
| ACSL5 | 670.57 | 213.25 | 804.04 | 176.23 | 4.43 | 0.047 | 0.450 | 0.83 |
| LOC644853 | 104.90 | 7.38 | 125.80 | 13.98 | 5.38 | 0.033 | 0.445 | 0.83 |
| TMEM38B | 119.10 | 0.52 | 142.84 | 4.79 | 7.88 | 0.016 | 0.450 | 0.83 |
| HS.566573 | 119.95 | 3.38 | 143.87 | 8.25 | 6.61 | 0.022 | 0.454 | 0.83 |
| PCF11 | 356.58 | 120.79 | 427.71 | 103.88 | 5.27 | 0.034 | 0.448 | 0.83 |
| LOC653641 | 113.96 | 10.76 | 136.73 | 7.99 | 5.91 | 0.027 | 0.447 | 0.83 |
| HPS5 | 270.85 | 58.86 | 325.04 | 58.45 | 192.36 | 0.000 | 0.639 | 0.83 |
| JOSD2 | 140.24 | 23.49 | 168.31 | 17.78 | 4.65 | 0.043 | 0.447 | 0.83 |
| GPX4 | 2435.10 | 720.02 | 2922.99 | 830.05 | 4.83 | 0.040 | 0.445 | 0.83 |
| LOC136157 | 105.36 | 11.23 | 126.48 | 10.05 | 4.52 | 0.046 | 0.448 | 0.83 |
| PIWIL4 | 106.95 | 7.43 | 128.40 | 8.97 | 20.36 | 0.002 | 0.447 | 0.83 |
| LOC643453 | 113.22 | 5.63 | 135.92 | 11.20 | 5.32 | 0.034 | 0.447 | 0.83 |
| USP17L5 | 96.52 | 2.87 | 115.90 | 5.76 | 4.83 | 0.040 | 0.444 | 0.83 |
| ZNF189 | 262.26 | 112.50 | 315.01 | 101.00 | 4.99 | 0.038 | 0.447 | 0.83 |
| HS.397465 | 181.20 | 46.73 | 217.68 | 33.63 | 4.81 | 0.041 | 0.444 | 0.83 |
| HS.554298 | 111.73 | 4.64 | 134.25 | 1.38 | 11.92 | 0.007 | 0.445 | 0.83 |
| LOC647839 | 98.13 | 7.45 | 117.91 | 3.71 | 5.26 | 0.034 | 0.448 | 0.83 |
| PSEN2 | 162.61 | 31.30 | 195.39 | 24.54 | 8.18 | 0.015 | 0.452 | 0.83 |
| TULP4 | 139.98 | 8.78 | 168.23 | 8.67 | 20.87 | 0.002 | 0.441 | 0.83 |
| ZNF571 | 115.40 | 4.82 | 138.70 | 6.56 | 7.30 | 0.018 | 0.450 | 0.83 |
| ZFYVE9 | 105.61 | 5.03 | 126.95 | 6.43 | 18.77 | 0.003 | 0.459 | 0.83 |
| C3ORF35 | 98.27 | 4.60 | 118.16 | 11.41 | 5.03 | 0.037 | 0.446 | 0.83 |
| LOC728590 | 9671.70 | 4367.78 | 11629.25 | 4759.10 | 4.96 | 0.038 | 0.446 | 0.83 |
| LOC647570 | 122.77 | 6.36 | 147.62 | 11.89 | 5.36 | 0.033 | 0.446 | 0.83 |
| FAM104A | 855.00 | 375.84 | 1028.27 | 338.36 | 7.94 | 0.015 | 0.452 | 0.83 |
| ACVR2A | 112.81 | 14.24 | 135.70 | 11.17 | 12.91 | 0.006 | 0.441 | 0.83 |
| LOC390551 | 102.76 | 5.72 | 123.70 | 9.37 | 5.65 | 0.030 | 0.446 | 0.83 |
| FCRL2 | 109.89 | 6.58 | 132.29 | 3.60 | 12.57 | 0.006 | 0.441 | 0.83 |
| LOC646064 | 116.86 | 7.09 | 140.72 | 11.66 | 5.80 | 0.029 | 0.444 | 0.83 |
| VPS13B | 122.33 | 14.11 | 147.31 | 20.75 | 5.20 | 0.035 | 0.447 | 0.83 |
| PTPRA | 128.71 | 10.24 | 155.02 | 7.35 | 9.94 | 0.010 | 0.456 | 0.83 |
| HS.582097 | 106.60 | 4.28 | 128.40 | 3.19 | 31.49 | 0.001 | 0.491 | 0.83 |
| HS.558282 | 104.26 | 4.61 | 125.61 | 7.79 | 5.08 | 0.037 | 0.447 | 0.83 |
| NOTCH2NL | 124.81 | 7.97 | 150.39 | 3.85 | 6.72 | 0.021 | 0.449 | 0.83 |
| LOC440261 | 104.94 | 3.16 | 126.46 | 8.59 | 5.90 | 0.028 | 0.447 | 0.83 |
| CCNI2 | 116.08 | 2.81 | 139.87 | 1.99 | 10.82 | 0.008 | 0.456 | 0.83 |
| LOC644472 | 93.24 | 6.52 | 112.42 | 4.66 | 17.06 | 0.003 | 0.454 | 0.83 |
| GPR177 | 101.94 | 5.43 | 122.94 | 12.24 | 4.73 | 0.042 | 0.445 | 0.83 |
| LYSMD3 | 137.21 | 46.12 | 165.48 | 41.59 | 6.96 | 0.020 | 0.447 | 0.83 |
| BST1 | 105.45 | 2.17 | 127.22 | 7.01 | 6.39 | 0.024 | 0.453 | 0.83 |
| KIAA1875 | 133.93 | 9.28 | 161.58 | 13.67 | 7.56 | 0.017 | 0.446 | 0.83 |
| HIVEP2 | 158.86 | 28.56 | 191.68 | 18.75 | 5.53 | 0.031 | 0.444 | 0.83 |
| LCT | 106.79 | 8.36 | 128.85 | 12.51 | 5.45 | 0.032 | 0.445 | 0.83 |
| C17ORF93 | 101.69 | 7.74 | 122.71 | 4.83 | 8.37 | 0.014 | 0.452 | 0.83 |
| LOC642342 | 116.93 | 5.20 | 141.09 | 8.81 | 6.15 | 0.025 | 0.448 | 0.83 |
| MEFV | 106.67 | 11.27 | 128.77 | 9.10 | 11.75 | 0.007 | 0.448 | 0.83 |
| RTN2 | 133.43 | 6.97 | 161.10 | 12.03 | 4.83 | 0.040 | 0.444 | 0.83 |
| PMEPA1 | 116.12 | 4.64 | 140.22 | 8.93 | 7.06 | 0.020 | 0.449 | 0.83 |
| SNRNP35 | 235.46 | 53.62 | 284.39 | 66.11 | 6.08 | 0.026 | 0.449 | 0.83 |
| FAM30A | 181.04 | 15.10 | 218.75 | 19.36 | 4.81 | 0.041 | 0.444 | 0.83 |
| IFT88 | 127.84 | 18.02 | 154.52 | 16.14 | 8.22 | 0.014 | 0.452 | 0.83 |
| HS.175465 | 109.08 | 2.49 | 131.87 | 2.55 | 8.74 | 0.013 | 0.455 | 0.83 |
| KIR3DL1 | 114.31 | 7.33 | 138.21 | 5.50 | 11.18 | 0.008 | 0.456 | 0.83 |
| CYP2A13 | 110.86 | 6.80 | 134.08 | 12.61 | 4.98 | 0.038 | 0.446 | 0.83 |
| HS.568329 | 128.13 | 7.31 | 154.99 | 9.62 | 7.29 | 0.018 | 0.450 | 0.83 |
| HS.90221 | 112.19 | 5.11 | 135.71 | 8.90 | 6.93 | 0.020 | 0.446 | 0.83 |
| PTGDR | 106.60 | 10.96 | 129.01 | 8.06 | 9.44 | 0.011 | 0.464 | 0.83 |
| LOC649587 | 103.30 | 4.37 | 125.03 | 10.54 | 4.89 | 0.039 | 0.444 | 0.83 |
| STX16 | 903.72 | 466.00 | 1093.92 | 428.98 | 6.51 | 0.023 | 0.452 | 0.83 |
| LOC728142 | 124.00 | 7.49 | 150.11 | 10.18 | 5.31 | 0.034 | 0.447 | 0.83 |
| HS.120208 | 97.31 | 6.09 | 117.80 | 8.49 | 13.76 | 0.005 | 0.448 | 0.83 |
| LOC729659 | 102.05 | 8.49 | 123.57 | 9.27 | 12.04 | 0.007 | 0.445 | 0.83 |
| LOC644243 | 115.51 | 16.09 | 139.89 | 15.70 | 4.96 | 0.038 | 0.446 | 0.83 |
| PPCS | 909.27 | 434.93 | 1101.59 | 458.10 | 4.65 | 0.043 | 0.447 | 0.83 |
| HS.14555 | 195.16 | 33.79 | 236.45 | 20.90 | 5.01 | 0.038 | 0.446 | 0.83 |
| HS.539482 | 117.63 | 13.99 | 142.54 | 12.52 | 17.70 | 0.003 | 0.446 | 0.83 |
| C20ORF24 | 5079.59 | 1955.89 | 6156.95 | 1936.02 | 27.67 | 0.001 | 0.460 | 0.83 |
| RALGAPA1 | 294.77 | 110.90 | 357.39 | 110.39 | 6.51 | 0.023 | 0.452 | 0.82 |
| WNT10A | 110.29 | 11.73 | 133.82 | 15.22 | 7.04 | 0.020 | 0.449 | 0.82 |
| HIC2 | 222.58 | 60.02 | 270.10 | 69.07 | 8.69 | 0.013 | 0.452 | 0.82 |
| C3ORF10 | 577.65 | 180.61 | 701.06 | 217.26 | 5.02 | 0.038 | 0.446 | 0.82 |
| LOC554223 | 110.49 | 7.53 | 134.10 | 2.82 | 5.12 | 0.036 | 0.447 | 0.82 |
| BRWD1 | 112.71 | 15.85 | 136.82 | 13.64 | 17.85 | 0.003 | 0.445 | 0.82 |
| PGAP3 | 209.67 | 44.74 | 254.54 | 44.40 | 12.35 | 0.006 | 0.443 | 0.82 |
| HS.545298 | 105.82 | 8.67 | 128.50 | 11.67 | 4.32 | 0.050 | 0.452 | 0.82 |
| LOC338586 | 99.46 | 4.25 | 120.86 | 7.76 | 7.25 | 0.018 | 0.450 | 0.82 |
| FICD | 190.75 | 36.95 | 231.79 | 47.50 | 6.23 | 0.025 | 0.448 | 0.82 |
| OR1S2 | 109.48 | 9.60 | 133.06 | 9.09 | 16.36 | 0.004 | 0.453 | 0.82 |
| HS.325015 | 125.47 | 6.04 | 152.52 | 2.06 | 10.38 | 0.009 | 0.453 | 0.82 |
| TAZ | 118.17 | 8.83 | 143.66 | 5.84 | 8.53 | 0.013 | 0.452 | 0.82 |
| HS.576825 | 113.66 | 4.06 | 138.18 | 6.17 | 4.63 | 0.044 | 0.447 | 0.82 |
| HS.583920 | 106.31 | 10.25 | 129.26 | 16.19 | 6.33 | 0.024 | 0.452 | 0.82 |
| HS.561656 | 110.27 | 5.91 | 134.11 | 14.38 | 4.87 | 0.040 | 0.445 | 0.82 |
| PVRL2 | 109.54 | 6.02 | 133.24 | 4.19 | 8.99 | 0.012 | 0.454 | 0.82 |
| PHF3 | 204.02 | 73.03 | 248.29 | 77.24 | 4.37 | 0.049 | 0.451 | 0.82 |
| SLC10A6 | 99.14 | 3.85 | 120.68 | 2.98 | 8.04 | 0.015 | 0.453 | 0.82 |
| CFLAR | 1464.39 | 557.37 | 1782.93 | 661.44 | 5.14 | 0.036 | 0.446 | 0.82 |
| P4HTM | 184.87 | 61.06 | 225.12 | 51.55 | 7.32 | 0.018 | 0.450 | 0.82 |
| LOC644580 | 104.13 | 7.14 | 126.82 | 6.29 | 7.46 | 0.017 | 0.448 | 0.82 |
| FBXW5 | 232.70 | 39.15 | 283.43 | 51.08 | 5.48 | 0.032 | 0.445 | 0.82 |
| BBS10 | 120.62 | 30.10 | 146.94 | 27.35 | 13.80 | 0.005 | 0.449 | 0.82 |
| EPHB2 | 113.67 | 8.02 | 138.49 | 8.75 | 15.98 | 0.004 | 0.453 | 0.82 |
| LOC654092 | 105.60 | 13.52 | 128.68 | 10.76 | 10.82 | 0.008 | 0.455 | 0.82 |
| MGC70857 | 156.24 | 5.17 | 190.39 | 7.86 | 12.28 | 0.007 | 0.439 | 0.82 |
| TREML3 | 107.48 | 7.70 | 131.01 | 12.39 | 5.92 | 0.027 | 0.448 | 0.82 |
| LOC100131349 | 126.48 | 12.52 | 154.21 | 6.59 | 4.75 | 0.042 | 0.444 | 0.82 |
| LOC100132428 | 115.99 | 3.47 | 141.47 | 8.78 | 5.23 | 0.035 | 0.448 | 0.82 |
| EREG | 126.34 | 4.25 | 154.10 | 4.38 | 24.36 | 0.002 | 0.436 | 0.82 |
| WDR33 | 130.39 | 18.27 | 159.07 | 15.62 | 10.47 | 0.009 | 0.456 | 0.82 |
| SOCS5 | 121.05 | 12.12 | 147.77 | 5.24 | 6.27 | 0.025 | 0.449 | 0.82 |
| GLCE | 164.97 | 40.69 | 201.40 | 46.99 | 6.07 | 0.026 | 0.448 | 0.82 |
| DLG4 | 114.60 | 11.93 | 139.93 | 12.52 | 54.57 | 0.000 | 0.635 | 0.82 |
| DNHL1 | 112.58 | 9.80 | 137.50 | 5.61 | 4.99 | 0.038 | 0.447 | 0.82 |
| ARHGEF11 | 105.41 | 9.88 | 128.78 | 15.70 | 6.83 | 0.021 | 0.448 | 0.82 |
| INO80D | 251.24 | 58.90 | 307.00 | 67.78 | 7.70 | 0.016 | 0.448 | 0.82 |
| LOC646043 | 112.35 | 9.08 | 137.29 | 5.38 | 11.59 | 0.007 | 0.448 | 0.82 |
| PHF12 | 207.33 | 23.76 | 253.36 | 17.50 | 7.18 | 0.019 | 0.450 | 0.82 |
| GGT3 | 103.97 | 6.95 | 127.10 | 4.88 | 5.11 | 0.036 | 0.447 | 0.82 |
| RAB24 | 129.09 | 12.90 | 157.82 | 7.42 | 6.04 | 0.026 | 0.449 | 0.82 |
| ADH4 | 105.75 | 7.93 | 129.30 | 12.02 | 9.98 | 0.010 | 0.454 | 0.82 |
| LOC402509 | 104.36 | 6.25 | 127.66 | 5.75 | 4.76 | 0.041 | 0.444 | 0.82 |
| LOC728244 | 7082.03 | 3893.35 | 8664.03 | 3692.90 | 10.36 | 0.009 | 0.452 | 0.82 |
| PLAG1 | 130.98 | 18.40 | 160.25 | 7.60 | 4.53 | 0.045 | 0.449 | 0.82 |
| PPDPF | 133.27 | 18.52 | 163.05 | 19.30 | 54.67 | 0.000 | 0.659 | 0.82 |
| APCDD1L | 101.86 | 3.99 | 124.63 | 8.64 | 5.47 | 0.032 | 0.445 | 0.82 |
| HS.126889 | 131.42 | 22.26 | 160.86 | 19.68 | 4.50 | 0.046 | 0.449 | 0.82 |
| CATSPER1 | 109.91 | 8.20 | 134.54 | 4.46 | 9.18 | 0.012 | 0.455 | 0.82 |
| ST6GALNAC2 | 113.44 | 7.20 | 138.87 | 8.54 | 28.75 | 0.001 | 0.453 | 0.82 |
| SCARF2 | 102.92 | 7.92 | 126.00 | 6.86 | 4.77 | 0.041 | 0.444 | 0.82 |
| MGST1 | 109.48 | 10.19 | 134.11 | 8.93 | 7.82 | 0.016 | 0.449 | 0.82 |
| KIAA1632 | 128.51 | 4.24 | 157.49 | 3.20 | 39.24 | 0.001 | 0.558 | 0.82 |
| POR | 176.87 | 4.36 | 216.83 | 14.25 | 6.45 | 0.023 | 0.453 | 0.82 |
| TTLL3 | 141.11 | 30.32 | 173.00 | 27.19 | 6.10 | 0.026 | 0.450 | 0.82 |
| MEI1 | 156.61 | 24.24 | 192.01 | 28.62 | 13.11 | 0.006 | 0.444 | 0.82 |
| LOC100129495 | 104.91 | 10.37 | 128.63 | 11.15 | 6.45 | 0.023 | 0.453 | 0.82 |
| LOC729580 | 145.32 | 9.90 | 178.22 | 13.32 | 6.07 | 0.026 | 0.449 | 0.82 |
| C5ORF53 | 133.59 | 29.90 | 164.01 | 23.07 | 4.57 | 0.045 | 0.448 | 0.81 |
| SSBP3 | 144.04 | 21.41 | 176.86 | 29.75 | 5.87 | 0.028 | 0.446 | 0.81 |
| DNAJB14 | 442.39 | 136.09 | 543.23 | 150.70 | 6.18 | 0.025 | 0.448 | 0.81 |
| FAM160B2 | 145.68 | 12.42 | 178.99 | 12.90 | 5.45 | 0.032 | 0.445 | 0.81 |
| ITGA6 | 102.29 | 6.61 | 125.69 | 9.44 | 4.63 | 0.044 | 0.447 | 0.81 |
| MGC15634 | 111.68 | 5.32 | 137.24 | 9.74 | 10.01 | 0.010 | 0.454 | 0.81 |
| TC2N | 129.16 | 32.06 | 158.75 | 29.31 | 10.85 | 0.008 | 0.455 | 0.81 |
| HS.543803 | 111.97 | 3.58 | 137.62 | 11.93 | 4.75 | 0.042 | 0.445 | 0.81 |
| TNC | 141.87 | 14.51 | 174.40 | 9.53 | 6.62 | 0.022 | 0.453 | 0.81 |
| C16ORF52 | 134.04 | 8.92 | 164.78 | 2.87 | 7.10 | 0.019 | 0.449 | 0.81 |
| CLEC4E | 120.47 | 9.76 | 148.11 | 16.48 | 4.79 | 0.041 | 0.444 | 0.81 |
| NLRP5 | 107.58 | 3.84 | 132.28 | 8.62 | 7.69 | 0.016 | 0.448 | 0.81 |
| CHD6 | 180.22 | 36.25 | 221.64 | 39.73 | 4.79 | 0.041 | 0.444 | 0.81 |
| C1ORF26 | 127.27 | 18.79 | 156.53 | 16.05 | 4.89 | 0.039 | 0.444 | 0.81 |
| LOC643811 | 108.86 | 7.32 | 133.90 | 14.53 | 5.93 | 0.027 | 0.447 | 0.81 |
| LOC100134634 | 137.56 | 9.04 | 169.25 | 5.05 | 9.48 | 0.011 | 0.464 | 0.81 |
| LOC440896 | 104.75 | 6.09 | 128.90 | 5.98 | 32.24 | 0.001 | 0.488 | 0.81 |
| VPS39 | 166.09 | 8.87 | 204.41 | 6.67 | 27.61 | 0.001 | 0.448 | 0.81 |
| SLC22A17 | 123.32 | 15.72 | 151.78 | 19.11 | 9.91 | 0.010 | 0.458 | 0.81 |
| SCYL3 | 365.20 | 123.20 | 449.55 | 150.56 | 5.34 | 0.033 | 0.446 | 0.81 |
| FPR2 | 101.98 | 6.85 | 125.56 | 6.80 | 401.21 | 0.000 | 0.294 | 0.81 |
| FAM160B1 | 408.21 | 108.86 | 502.87 | 123.02 | 4.55 | 0.045 | 0.448 | 0.81 |
| HS.55246 | 127.89 | 4.59 | 157.55 | 6.25 | 5.94 | 0.027 | 0.447 | 0.81 |
| LOC650737 | 174.18 | 13.20 | 214.67 | 4.73 | 8.24 | 0.014 | 0.451 | 0.81 |
| MAF | 119.48 | 14.79 | 147.33 | 12.72 | 5.57 | 0.031 | 0.444 | 0.81 |
| JMJD1C | 520.27 | 226.48 | 641.72 | 214.66 | 5.08 | 0.037 | 0.446 | 0.81 |
| SURF1 | 361.43 | 119.58 | 445.81 | 94.05 | 5.50 | 0.032 | 0.446 | 0.81 |
| NPIP | 258.97 | 49.17 | 319.45 | 53.24 | 9.09 | 0.012 | 0.456 | 0.81 |
| GRIK1 | 104.26 | 7.44 | 128.63 | 13.97 | 5.76 | 0.029 | 0.445 | 0.81 |
| RTN2 | 102.92 | 4.53 | 127.01 | 9.39 | 4.97 | 0.038 | 0.446 | 0.81 |
| LOC653188 | 104.47 | 5.20 | 128.92 | 11.41 | 5.03 | 0.037 | 0.447 | 0.81 |
| HOM-TES-103 | 105.11 | 8.73 | 129.72 | 10.35 | 9.42 | 0.011 | 0.464 | 0.81 |
| CHST7 | 184.70 | 34.40 | 227.96 | 32.41 | 4.85 | 0.040 | 0.444 | 0.81 |
| HS.436572 | 116.79 | 10.13 | 144.16 | 11.02 | 5.04 | 0.037 | 0.447 | 0.81 |
| ZFAND3 | 141.50 | 6.61 | 174.67 | 1.20 | 7.87 | 0.016 | 0.451 | 0.81 |
| LOC399746 | 109.19 | 8.57 | 134.79 | 13.85 | 5.87 | 0.028 | 0.445 | 0.81 |
| MEGF9 | 128.03 | 7.61 | 158.14 | 12.62 | 7.20 | 0.019 | 0.450 | 0.81 |
| PRDM16 | 98.26 | 4.88 | 121.39 | 11.70 | 4.57 | 0.045 | 0.448 | 0.81 |
| RBPJ | 332.95 | 160.74 | 411.34 | 136.88 | 5.63 | 0.030 | 0.446 | 0.81 |
| TP53I3 | 155.35 | 28.84 | 192.12 | 32.37 | 8.14 | 0.015 | 0.452 | 0.81 |
| GPER | 100.90 | 9.49 | 124.80 | 12.85 | 9.59 | 0.011 | 0.464 | 0.81 |
| LOC649095 | 111.67 | 6.91 | 138.20 | 9.88 | 4.64 | 0.044 | 0.447 | 0.81 |
| GPR56 | 182.29 | 47.75 | 225.78 | 49.30 | 6.37 | 0.024 | 0.453 | 0.81 |
| ZHX2 | 303.42 | 90.47 | 375.81 | 82.92 | 5.70 | 0.029 | 0.445 | 0.81 |
| ETV2 | 117.52 | 16.61 | 145.58 | 18.36 | 9.24 | 0.012 | 0.456 | 0.81 |
| OSBPL2 | 132.51 | 11.97 | 164.28 | 12.22 | 4.98 | 0.038 | 0.446 | 0.81 |
| SP140L | 155.37 | 8.82 | 192.86 | 12.63 | 4.72 | 0.042 | 0.445 | 0.81 |
| IL18BP | 113.91 | 7.58 | 141.49 | 14.37 | 4.62 | 0.044 | 0.447 | 0.81 |
| NOL3 | 111.11 | 6.94 | 138.03 | 2.66 | 10.02 | 0.010 | 0.454 | 0.81 |
| HCG27 | 112.16 | 4.13 | 139.35 | 12.92 | 5.19 | 0.035 | 0.447 | 0.80 |
| LOC651195 | 102.70 | 8.90 | 127.63 | 14.46 | 6.99 | 0.020 | 0.448 | 0.80 |
| ABL2 | 108.38 | 7.12 | 134.76 | 13.84 | 5.10 | 0.036 | 0.446 | 0.80 |
| HS.320051 | 113.61 | 4.36 | 141.28 | 6.24 | 15.40 | 0.004 | 0.450 | 0.80 |
| NCF1B | 123.78 | 4.85 | 153.95 | 7.56 | 17.06 | 0.003 | 0.453 | 0.80 |
| PMEPA1 | 143.00 | 23.06 | 177.92 | 10.54 | 4.70 | 0.042 | 0.447 | 0.80 |
| SNORA29 | 110.64 | 11.16 | 137.67 | 3.96 | 5.22 | 0.035 | 0.448 | 0.80 |
| HS.133324 | 133.31 | 9.37 | 165.91 | 11.04 | 7.83 | 0.016 | 0.450 | 0.80 |
| TMEM120A | 200.61 | 39.59 | 249.67 | 32.43 | 5.92 | 0.027 | 0.448 | 0.80 |
| LOC649500 | 109.86 | 6.92 | 136.76 | 6.56 | 12.08 | 0.007 | 0.444 | 0.80 |
| HS.309308 | 102.72 | 2.56 | 127.91 | 3.99 | 18.72 | 0.003 | 0.454 | 0.80 |
| LOC440345 | 133.60 | 5.02 | 166.38 | 10.26 | 4.35 | 0.049 | 0.452 | 0.80 |
| C20ORF117 | 201.17 | 17.77 | 250.56 | 23.67 | 5.57 | 0.031 | 0.445 | 0.80 |
| FAM104B | 129.35 | 17.41 | 161.12 | 20.02 | 17.15 | 0.003 | 0.453 | 0.80 |
| LOC730286 | 115.75 | 15.29 | 144.21 | 6.32 | 4.56 | 0.045 | 0.448 | 0.80 |
| HS.536748 | 182.66 | 51.12 | 227.61 | 50.79 | 9.78 | 0.010 | 0.457 | 0.80 |
| SRC | 455.69 | 32.97 | 567.87 | 18.13 | 4.42 | 0.047 | 0.449 | 0.80 |
| IDH3G | 325.20 | 26.40 | 405.30 | 49.35 | 5.37 | 0.033 | 0.445 | 0.80 |
| LOC100134241 | 104.72 | 14.94 | 130.53 | 18.03 | 9.39 | 0.011 | 0.464 | 0.80 |
| JUP | 160.25 | 33.51 | 199.82 | 42.35 | 4.82 | 0.041 | 0.445 | 0.80 |
| HS.197670 | 101.60 | 6.93 | 126.71 | 6.84 | 12.65 | 0.006 | 0.440 | 0.80 |
| TNFSF8 | 119.38 | 2.97 | 148.89 | 3.68 | 8.71 | 0.013 | 0.453 | 0.80 |
| APOL1 | 111.84 | 5.85 | 139.51 | 13.32 | 5.57 | 0.031 | 0.445 | 0.80 |
| C14ORF100 | 422.16 | 159.00 | 526.84 | 147.47 | 5.99 | 0.027 | 0.448 | 0.80 |
| TFPI2 | 109.17 | 1.34 | 136.25 | 9.07 | 4.90 | 0.039 | 0.444 | 0.80 |
| PDLIM4 | 95.96 | 4.63 | 119.83 | 12.80 | 4.63 | 0.044 | 0.447 | 0.80 |
| ODF3B | 131.53 | 3.35 | 164.31 | 6.38 | 10.93 | 0.008 | 0.455 | 0.80 |
| HS.578161 | 109.56 | 1.10 | 136.94 | 2.05 | 42.51 | 0.001 | 0.622 | 0.80 |
| PSTPIP1 | 226.09 | 81.07 | 282.61 | 90.70 | 7.86 | 0.016 | 0.450 | 0.80 |
| C19ORF29 | 174.81 | 24.28 | 218.57 | 29.31 | 12.87 | 0.006 | 0.437 | 0.80 |
| HS.131041 | 105.94 | 8.99 | 132.47 | 12.00 | 10.37 | 0.009 | 0.453 | 0.80 |
| ANKRD10 | 333.48 | 176.20 | 417.08 | 144.19 | 4.52 | 0.046 | 0.449 | 0.80 |
| UTY | 135.77 | 20.08 | 169.87 | 31.07 | 5.37 | 0.033 | 0.445 | 0.80 |
| CABLES2 | 136.43 | 15.91 | 170.71 | 23.90 | 6.50 | 0.023 | 0.452 | 0.80 |
| HS.516314 | 111.92 | 11.80 | 140.05 | 5.00 | 4.31 | 0.050 | 0.452 | 0.80 |
| RASSF7 | 461.10 | 100.70 | 577.01 | 104.30 | 55.70 | 0.000 | 0.692 | 0.80 |
| LOC653545 | 116.80 | 3.75 | 146.24 | 5.25 | 31.50 | 0.001 | 0.496 | 0.80 |
| CLIP2 | 168.47 | 9.34 | 211.01 | 19.91 | 6.77 | 0.021 | 0.448 | 0.80 |
| TAPBP | 153.23 | 33.82 | 192.05 | 25.46 | 4.45 | 0.047 | 0.450 | 0.80 |
| RAPH1 | 108.49 | 6.31 | 136.00 | 5.10 | 10.97 | 0.008 | 0.455 | 0.80 |
| PRAMEF13 | 125.07 | 9.58 | 156.90 | 7.42 | 8.61 | 0.013 | 0.451 | 0.80 |
| LOC100134006 | 95.56 | 12.29 | 119.91 | 4.39 | 4.47 | 0.047 | 0.449 | 0.80 |
| LOC729609 | 106.54 | 15.08 | 133.71 | 6.55 | 4.50 | 0.046 | 0.449 | 0.80 |
| TM9SF2 | 1440.72 | 689.39 | 1808.15 | 645.81 | 13.94 | 0.005 | 0.449 | 0.80 |
| ARAP1 | 215.33 | 26.78 | 270.30 | 33.09 | 9.20 | 0.012 | 0.457 | 0.80 |
| PXMP4 | 115.39 | 4.54 | 144.87 | 10.61 | 6.06 | 0.026 | 0.448 | 0.80 |
| FAM84B | 136.41 | 18.76 | 171.27 | 20.29 | 34.42 | 0.001 | 0.498 | 0.80 |
| GRIP2 | 95.22 | 3.19 | 119.55 | 6.36 | 4.57 | 0.045 | 0.448 | 0.80 |
| LOC727752 | 97.25 | 2.11 | 122.12 | 5.41 | 6.36 | 0.024 | 0.452 | 0.80 |
| CLEC7A | 100.64 | 3.80 | 126.38 | 9.08 | 5.07 | 0.037 | 0.446 | 0.80 |
| IL12A | 109.87 | 8.28 | 138.07 | 6.00 | 8.45 | 0.014 | 0.452 | 0.80 |
| ERP27 | 127.25 | 13.96 | 159.95 | 22.73 | 5.75 | 0.029 | 0.445 | 0.80 |
| VPS28 | 278.67 | 50.09 | 350.32 | 30.35 | 5.08 | 0.037 | 0.447 | 0.80 |
| RYR1 | 117.83 | 8.33 | 148.14 | 17.81 | 5.13 | 0.036 | 0.447 | 0.80 |
| MAN2C1 | 182.79 | 37.32 | 230.06 | 32.95 | 10.02 | 0.010 | 0.454 | 0.79 |
| LAD1 | 107.28 | 7.99 | 135.05 | 10.44 | 8.44 | 0.014 | 0.452 | 0.79 |
| LCP2 | 148.37 | 3.07 | 186.79 | 11.19 | 6.24 | 0.025 | 0.449 | 0.79 |
| CCDC135 | 116.92 | 10.16 | 147.28 | 11.65 | 6.61 | 0.022 | 0.454 | 0.79 |
| FOXP1 | 116.14 | 11.74 | 146.31 | 8.07 | 6.00 | 0.027 | 0.448 | 0.79 |
| LOC100130900 | 102.45 | 1.36 | 129.10 | 4.01 | 8.86 | 0.012 | 0.457 | 0.79 |
| AXL | 113.88 | 9.62 | 143.57 | 10.08 | 51.22 | 0.000 | 0.643 | 0.79 |
| PLEKHM2 | 1257.00 | 497.64 | 1584.93 | 556.28 | 6.98 | 0.020 | 0.447 | 0.79 |
| ATP2A3 | 155.14 | 10.48 | 195.64 | 8.18 | 18.65 | 0.003 | 0.450 | 0.79 |
| SGMS1 | 129.79 | 8.43 | 163.68 | 17.46 | 6.42 | 0.023 | 0.453 | 0.79 |
| LOC100130623 | 155.85 | 14.14 | 196.57 | 7.12 | 5.02 | 0.037 | 0.447 | 0.79 |
| ZNF76 | 157.78 | 10.26 | 199.11 | 16.38 | 4.86 | 0.040 | 0.445 | 0.79 |
| DLGAP1 | 97.80 | 4.71 | 123.47 | 5.21 | 11.60 | 0.007 | 0.450 | 0.79 |
| C1ORF147 | 101.40 | 8.00 | 128.02 | 8.21 | 5.72 | 0.029 | 0.445 | 0.79 |
| AP3S1 | 427.22 | 151.13 | 539.73 | 159.59 | 6.83 | 0.021 | 0.448 | 0.79 |
| HS.440533 | 104.89 | 8.95 | 132.53 | 11.51 | 5.87 | 0.028 | 0.446 | 0.79 |
| AIM1 | 158.76 | 17.28 | 200.62 | 12.92 | 7.76 | 0.016 | 0.449 | 0.79 |
| LOC202134 | 118.03 | 8.74 | 149.28 | 10.29 | 6.73 | 0.021 | 0.450 | 0.79 |
| FAM43A | 185.53 | 51.00 | 234.70 | 46.48 | 5.56 | 0.031 | 0.445 | 0.79 |
| HIST2H2AA3 | 117.65 | 9.60 | 148.85 | 10.96 | 4.60 | 0.044 | 0.448 | 0.79 |
| USP2 | 107.56 | 2.88 | 136.14 | 10.51 | 6.49 | 0.023 | 0.452 | 0.79 |
| LOC729348 | 139.83 | 14.72 | 177.00 | 8.98 | 6.43 | 0.023 | 0.453 | 0.79 |
| RAB4B | 193.74 | 18.85 | 245.25 | 13.99 | 18.31 | 0.003 | 0.456 | 0.79 |
| PAOX | 361.04 | 145.87 | 457.14 | 173.53 | 5.41 | 0.033 | 0.445 | 0.79 |
| C2ORF21 | 117.91 | 6.63 | 149.31 | 11.54 | 5.63 | 0.030 | 0.446 | 0.79 |
| LOC729008 | 114.84 | 9.21 | 145.52 | 7.29 | 26.39 | 0.001 | 0.449 | 0.79 |
| MEIS3P1 | 122.80 | 16.97 | 155.65 | 5.94 | 5.13 | 0.036 | 0.446 | 0.79 |
| DEF6 | 434.80 | 189.82 | 551.16 | 186.24 | 12.74 | 0.006 | 0.438 | 0.79 |
| LOC391370 | 8147.54 | 4427.56 | 10328.85 | 4525.68 | 6.65 | 0.022 | 0.453 | 0.79 |
| TESK2 | 151.45 | 25.67 | 192.01 | 21.29 | 14.63 | 0.005 | 0.442 | 0.79 |
| LOC649088 | 108.34 | 9.73 | 137.35 | 7.56 | 7.02 | 0.020 | 0.449 | 0.79 |
| ITM2B | 110.44 | 11.73 | 140.07 | 19.58 | 6.53 | 0.023 | 0.453 | 0.79 |
| SPG3A | 133.07 | 15.54 | 168.91 | 13.92 | 6.17 | 0.025 | 0.448 | 0.79 |
| TLR6 | 127.29 | 8.31 | 161.58 | 8.95 | 11.09 | 0.008 | 0.458 | 0.79 |
| TAP1 | 6344.37 | 1775.76 | 8055.84 | 2342.54 | 5.11 | 0.036 | 0.447 | 0.79 |
| ZNF28 | 131.93 | 7.85 | 167.56 | 17.81 | 5.92 | 0.027 | 0.447 | 0.79 |
| C10ORF26 | 139.65 | 18.84 | 177.36 | 19.65 | 5.67 | 0.030 | 0.446 | 0.79 |
| NBPF14 | 180.69 | 46.75 | 229.63 | 36.50 | 4.96 | 0.038 | 0.446 | 0.79 |
| TIAF1 | 636.08 | 190.29 | 808.46 | 219.18 | 6.27 | 0.025 | 0.450 | 0.79 |
| WHAMM | 158.71 | 43.52 | 201.75 | 48.48 | 8.01 | 0.015 | 0.452 | 0.79 |
| NECAP1 | 455.54 | 166.18 | 579.50 | 178.08 | 4.43 | 0.047 | 0.449 | 0.79 |
| ZNF333 | 125.05 | 6.67 | 159.28 | 15.43 | 6.50 | 0.023 | 0.452 | 0.79 |
| ADAM28 | 107.93 | 9.14 | 137.51 | 19.42 | 4.99 | 0.038 | 0.447 | 0.78 |
| NLRP12 | 120.91 | 13.66 | 154.11 | 6.28 | 7.65 | 0.017 | 0.446 | 0.78 |
| GDE1 | 140.62 | 20.74 | 179.26 | 20.39 | 6.51 | 0.023 | 0.452 | 0.78 |
| PHF21A | 592.54 | 256.64 | 755.68 | 260.92 | 5.10 | 0.036 | 0.447 | 0.78 |
| ANK3 | 119.64 | 7.51 | 152.61 | 4.83 | 19.56 | 0.003 | 0.456 | 0.78 |
| KIAA1683 | 123.90 | 6.56 | 158.09 | 14.60 | 4.98 | 0.038 | 0.447 | 0.78 |
| CLOCK | 129.08 | 6.08 | 164.75 | 8.07 | 4.47 | 0.047 | 0.449 | 0.78 |
| RPS6KA1 | 773.52 | 234.96 | 987.26 | 292.06 | 5.29 | 0.034 | 0.447 | 0.78 |
| COMMD10 | 346.09 | 127.90 | 441.79 | 128.20 | 8.85 | 0.013 | 0.456 | 0.78 |
| HS.543093 | 107.02 | 5.14 | 136.65 | 7.66 | 4.37 | 0.049 | 0.451 | 0.78 |
| CBFA2T2 | 145.19 | 10.43 | 185.38 | 14.99 | 5.33 | 0.033 | 0.447 | 0.78 |
| DNM2 | 203.34 | 5.61 | 259.68 | 12.72 | 7.70 | 0.016 | 0.449 | 0.78 |
| ZMYM1 | 181.53 | 36.66 | 231.87 | 31.27 | 7.25 | 0.018 | 0.450 | 0.78 |
| UGP2 | 128.74 | 16.44 | 164.46 | 16.37 | 4.63 | 0.044 | 0.447 | 0.78 |
| SLC48A1 | 152.97 | 6.09 | 195.43 | 20.10 | 5.15 | 0.036 | 0.446 | 0.78 |
| C17ORF60 | 114.75 | 9.53 | 146.61 | 5.67 | 4.55 | 0.045 | 0.448 | 0.78 |
| INADL | 126.18 | 12.97 | 161.25 | 17.02 | 13.27 | 0.006 | 0.446 | 0.78 |
| KLRD1 | 119.31 | 6.71 | 152.52 | 12.28 | 4.57 | 0.045 | 0.448 | 0.78 |
| HS.186848 | 109.29 | 3.41 | 139.75 | 4.93 | 6.76 | 0.021 | 0.448 | 0.78 |
| HS.475334 | 203.37 | 49.03 | 260.18 | 41.87 | 5.22 | 0.035 | 0.446 | 0.78 |
| KRT75 | 107.75 | 19.61 | 137.87 | 15.33 | 9.56 | 0.011 | 0.462 | 0.78 |
| TRIM8 | 512.53 | 196.17 | 655.85 | 222.95 | 6.20 | 0.025 | 0.449 | 0.78 |
| MTIF3 | 513.05 | 195.76 | 656.60 | 203.39 | 16.82 | 0.004 | 0.452 | 0.78 |
| NBEA | 107.48 | 2.42 | 137.57 | 8.35 | 6.82 | 0.021 | 0.448 | 0.78 |
| CPEB4 | 180.84 | 52.24 | 231.50 | 36.45 | 4.86 | 0.040 | 0.445 | 0.78 |
| CLN3 | 161.40 | 22.81 | 206.80 | 30.28 | 9.09 | 0.012 | 0.457 | 0.78 |
| BMF | 190.66 | 32.18 | 244.31 | 24.38 | 4.84 | 0.040 | 0.444 | 0.78 |
| MED12 | 246.02 | 33.66 | 315.47 | 36.28 | 14.57 | 0.005 | 0.442 | 0.78 |
| KLF6 | 616.88 | 190.35 | 791.06 | 157.30 | 4.41 | 0.048 | 0.450 | 0.78 |
| FBLN5 | 114.46 | 2.73 | 146.83 | 12.41 | 5.51 | 0.031 | 0.446 | 0.78 |
| CNPY3 | 206.52 | 23.99 | 264.94 | 27.73 | 11.38 | 0.008 | 0.452 | 0.78 |
| ZNF564 | 157.20 | 35.41 | 201.69 | 36.63 | 17.27 | 0.003 | 0.456 | 0.78 |
| DOK1 | 108.79 | 5.93 | 139.58 | 12.26 | 7.79 | 0.016 | 0.449 | 0.78 |
| RPL9 | 5910.57 | 1973.94 | 7584.21 | 2603.19 | 4.31 | 0.050 | 0.452 | 0.78 |
| CD44 | 110.98 | 12.58 | 142.42 | 5.97 | 5.08 | 0.037 | 0.447 | 0.78 |
| BTG2 | 811.65 | 126.60 | 1041.88 | 112.46 | 17.77 | 0.003 | 0.445 | 0.78 |
| ZNF362 | 165.35 | 42.44 | 212.36 | 48.66 | 4.33 | 0.049 | 0.452 | 0.78 |
| MGC10997 | 104.36 | 12.92 | 134.06 | 15.06 | 20.22 | 0.002 | 0.452 | 0.78 |
| LOC100128908 | 118.66 | 7.67 | 152.46 | 3.09 | 9.67 | 0.011 | 0.462 | 0.78 |
| PHF11 | 1794.32 | 695.51 | 2305.67 | 888.37 | 4.41 | 0.048 | 0.450 | 0.78 |
| CYB5R4 | 639.63 | 303.18 | 822.08 | 311.10 | 38.79 | 0.001 | 0.514 | 0.78 |
| ACACB | 116.69 | 15.20 | 149.98 | 17.53 | 15.41 | 0.004 | 0.452 | 0.78 |
| RNH1 | 477.49 | 67.62 | 613.97 | 111.79 | 5.35 | 0.033 | 0.446 | 0.78 |
| HS.126768 | 160.10 | 20.25 | 205.91 | 30.05 | 5.08 | 0.037 | 0.446 | 0.78 |
| PI4K2A | 176.06 | 37.60 | 226.44 | 53.97 | 5.32 | 0.034 | 0.447 | 0.78 |
| S1PR3 | 102.54 | 6.64 | 131.98 | 17.40 | 4.39 | 0.048 | 0.451 | 0.78 |
| MKRN1 | 2171.69 | 803.89 | 2799.40 | 890.14 | 5.21 | 0.035 | 0.446 | 0.78 |
| WHAMM | 186.33 | 54.78 | 240.33 | 54.48 | 6.32 | 0.024 | 0.451 | 0.78 |
| WASL | 195.70 | 39.29 | 252.61 | 36.53 | 5.95 | 0.027 | 0.448 | 0.77 |
| FOXO3 | 190.20 | 44.19 | 245.57 | 30.01 | 5.09 | 0.036 | 0.446 | 0.77 |
| FGF9 | 117.34 | 15.96 | 151.51 | 19.21 | 17.85 | 0.003 | 0.443 | 0.77 |
| MLL4 | 242.18 | 26.88 | 312.78 | 36.30 | 9.39 | 0.011 | 0.463 | 0.77 |
| DNAJB2 | 661.16 | 162.93 | 853.93 | 220.17 | 5.47 | 0.032 | 0.445 | 0.77 |
| CCS | 137.44 | 21.10 | 177.61 | 11.28 | 7.08 | 0.019 | 0.450 | 0.77 |
| ARHGAP23 | 127.48 | 6.85 | 164.79 | 8.07 | 5.79 | 0.029 | 0.444 | 0.77 |
| FRAT1 | 105.94 | 1.86 | 136.98 | 2.66 | 24.76 | 0.002 | 0.432 | 0.77 |
| LOC440348 | 286.05 | 43.81 | 369.94 | 42.14 | 56.46 | 0.000 | 0.706 | 0.77 |
| C1ORF166 | 229.90 | 78.42 | 297.38 | 81.06 | 19.33 | 0.003 | 0.453 | 0.77 |
| AGTRAP | 133.16 | 14.54 | 172.26 | 15.52 | 5.13 | 0.036 | 0.446 | 0.77 |
| C19ORF22 | 486.59 | 150.79 | 629.45 | 106.51 | 5.25 | 0.034 | 0.447 | 0.77 |
| TNFRSF6B | 109.90 | 6.51 | 142.18 | 6.97 | 25.92 | 0.001 | 0.433 | 0.77 |
| GRIPAP1 | 433.01 | 107.03 | 560.78 | 93.75 | 13.12 | 0.006 | 0.445 | 0.77 |
| CXORF12 | 132.39 | 10.73 | 171.51 | 24.60 | 4.60 | 0.044 | 0.448 | 0.77 |
| NRCAM | 115.45 | 19.89 | 149.59 | 16.77 | 7.63 | 0.017 | 0.446 | 0.77 |
| COL1A2 | 119.93 | 3.43 | 155.40 | 5.38 | 17.46 | 0.003 | 0.451 | 0.77 |
| LOC730092 | 135.41 | 9.87 | 175.48 | 12.73 | 19.10 | 0.003 | 0.448 | 0.77 |
| OSGIN1 | 116.53 | 12.20 | 151.06 | 14.72 | 6.98 | 0.020 | 0.448 | 0.77 |
| TRPC4AP | 322.20 | 71.25 | 417.77 | 79.50 | 6.30 | 0.024 | 0.450 | 0.77 |
| UBC | 12447.74 | 4123.52 | 16150.85 | 3809.65 | 5.72 | 0.029 | 0.445 | 0.77 |
| KDM5B | 199.41 | 60.94 | 258.77 | 52.93 | 12.49 | 0.006 | 0.444 | 0.77 |
| HS.571151 | 118.78 | 5.14 | 154.24 | 3.34 | 19.20 | 0.003 | 0.453 | 0.77 |
| HS.527535 | 124.79 | 22.14 | 162.09 | 19.99 | 4.34 | 0.049 | 0.452 | 0.77 |
| VAPA | 112.58 | 11.75 | 146.23 | 4.98 | 4.46 | 0.047 | 0.449 | 0.77 |
| LOC642441 | 109.77 | 10.00 | 142.64 | 11.60 | 5.45 | 0.032 | 0.445 | 0.77 |
| CPT1B | 201.49 | 30.62 | 261.82 | 40.59 | 10.48 | 0.009 | 0.456 | 0.77 |
| RSU1 | 478.44 | 234.32 | 622.44 | 195.62 | 6.44 | 0.023 | 0.453 | 0.77 |
| TRIO | 133.82 | 9.77 | 174.10 | 4.26 | 9.48 | 0.011 | 0.464 | 0.77 |
| OVGP1 | 140.52 | 15.98 | 182.91 | 24.86 | 5.46 | 0.032 | 0.445 | 0.77 |
| LOC401252 | 106.83 | 9.39 | 139.21 | 17.61 | 6.79 | 0.021 | 0.448 | 0.77 |
| LOC100131541 | 134.49 | 10.00 | 175.26 | 6.61 | 5.70 | 0.029 | 0.445 | 0.77 |
| LOC653157 | 108.66 | 8.33 | 141.61 | 9.69 | 6.29 | 0.024 | 0.449 | 0.77 |
| ATG9A | 219.50 | 27.69 | 286.11 | 44.44 | 5.77 | 0.029 | 0.444 | 0.77 |
| PPAP2B | 98.23 | 3.38 | 128.05 | 11.86 | 4.79 | 0.041 | 0.444 | 0.77 |
| ZNF836 | 108.21 | 2.09 | 141.06 | 1.30 | 18.71 | 0.003 | 0.453 | 0.77 |
| HS.576963 | 105.79 | 11.79 | 137.92 | 23.55 | 4.59 | 0.044 | 0.448 | 0.77 |
| LIPT1 | 166.84 | 19.67 | 217.66 | 28.48 | 7.13 | 0.019 | 0.449 | 0.77 |
| SH2B1 | 160.01 | 31.18 | 208.76 | 15.66 | 5.44 | 0.032 | 0.445 | 0.77 |
| ILK | 2173.98 | 767.31 | 2836.82 | 945.40 | 6.41 | 0.024 | 0.454 | 0.77 |
| ELF4 | 420.19 | 117.56 | 548.38 | 139.60 | 5.71 | 0.029 | 0.445 | 0.77 |
| SULT1A3 | 148.83 | 10.51 | 194.26 | 7.68 | 12.09 | 0.007 | 0.444 | 0.77 |
| IFIT3 | 122.25 | 12.05 | 159.59 | 10.72 | 5.66 | 0.030 | 0.446 | 0.77 |
| OXR1 | 376.80 | 118.42 | 491.89 | 113.79 | 23.19 | 0.002 | 0.454 | 0.77 |
| SNAI3 | 121.89 | 12.59 | 159.21 | 12.93 | 41.30 | 0.001 | 0.589 | 0.77 |
| CARD16 | 135.16 | 13.54 | 176.58 | 13.55 | 22.38 | 0.002 | 0.448 | 0.77 |
| SLC22A15 | 102.98 | 15.48 | 134.54 | 17.87 | 8.82 | 0.013 | 0.456 | 0.77 |
| IFIH1 | 698.41 | 278.82 | 912.57 | 253.90 | 7.39 | 0.018 | 0.449 | 0.77 |
| WSB1 | 371.35 | 111.66 | 485.32 | 148.38 | 4.65 | 0.043 | 0.447 | 0.77 |
| PACRGL | 108.70 | 10.87 | 142.08 | 17.71 | 6.66 | 0.022 | 0.452 | 0.77 |
| ZRANB1 | 192.93 | 43.59 | 252.25 | 52.41 | 10.15 | 0.010 | 0.454 | 0.76 |
| HS.572642 | 186.90 | 42.47 | 244.44 | 52.53 | 4.53 | 0.046 | 0.449 | 0.76 |
| RGPD1 | 109.18 | 6.54 | 142.79 | 6.86 | 45.18 | 0.000 | 0.609 | 0.76 |
| APOF | 99.40 | 1.77 | 130.02 | 13.58 | 4.49 | 0.046 | 0.448 | 0.76 |
| LOC647691 | 147.94 | 24.35 | 193.52 | 33.90 | 7.95 | 0.015 | 0.453 | 0.76 |
| SCAMP2 | 150.68 | 10.41 | 197.13 | 4.40 | 8.04 | 0.015 | 0.453 | 0.76 |
| LGALS8 | 351.25 | 125.34 | 460.00 | 158.91 | 5.59 | 0.031 | 0.445 | 0.76 |
| VPS26B | 149.68 | 13.16 | 196.03 | 13.22 | 6.24 | 0.025 | 0.449 | 0.76 |
| LLPH | 781.96 | 321.74 | 1024.45 | 277.29 | 6.90 | 0.020 | 0.446 | 0.76 |
| C19ORF64 | 114.74 | 8.60 | 150.38 | 20.04 | 5.07 | 0.037 | 0.446 | 0.76 |
| GSDMD | 819.18 | 352.90 | 1073.81 | 343.24 | 16.83 | 0.004 | 0.454 | 0.76 |
| AFF1 | 182.03 | 25.99 | 238.65 | 26.73 | 21.13 | 0.002 | 0.443 | 0.76 |
| TGM2 | 104.50 | 15.34 | 137.01 | 9.47 | 9.24 | 0.011 | 0.456 | 0.76 |
| HS.334831 | 168.66 | 24.66 | 221.19 | 4.62 | 4.31 | 0.050 | 0.452 | 0.76 |
| RSU1 | 129.90 | 11.74 | 170.40 | 10.09 | 5.00 | 0.038 | 0.447 | 0.76 |
| TPSAB1 | 106.86 | 6.85 | 140.20 | 18.84 | 4.81 | 0.041 | 0.444 | 0.76 |
| DERL1 | 734.08 | 267.51 | 963.19 | 275.37 | 38.50 | 0.001 | 0.514 | 0.76 |
| VAMP4 | 189.60 | 43.47 | 248.88 | 63.88 | 4.87 | 0.040 | 0.445 | 0.76 |
| ARHGAP22 | 135.33 | 16.09 | 177.67 | 30.11 | 4.98 | 0.038 | 0.447 | 0.76 |
| FLRT2 | 122.33 | 6.98 | 160.63 | 10.10 | 5.11 | 0.036 | 0.447 | 0.76 |
| TMEM9B | 596.16 | 175.24 | 783.07 | 238.82 | 4.49 | 0.046 | 0.449 | 0.76 |
| ARL15 | 123.92 | 15.29 | 162.81 | 6.10 | 6.82 | 0.021 | 0.448 | 0.76 |
| RYR1 | 117.55 | 5.30 | 154.44 | 13.47 | 7.70 | 0.016 | 0.449 | 0.76 |
| SLCO3A1 | 113.07 | 8.13 | 148.57 | 13.90 | 4.94 | 0.039 | 0.445 | 0.76 |
| RFWD2 | 801.51 | 380.16 | 1053.56 | 376.87 | 40.85 | 0.001 | 0.578 | 0.76 |
| PPP2R2B | 117.62 | 20.18 | 154.62 | 33.26 | 4.86 | 0.040 | 0.445 | 0.76 |
| LOC100132444 | 179.38 | 49.57 | 235.82 | 47.62 | 19.14 | 0.003 | 0.451 | 0.76 |
| ATP6V0B | 443.60 | 101.77 | 583.59 | 78.20 | 6.30 | 0.024 | 0.450 | 0.76 |
| LOC728650 | 212.60 | 39.58 | 279.71 | 24.97 | 4.85 | 0.040 | 0.444 | 0.76 |
| ZNF573 | 160.70 | 14.60 | 211.44 | 30.60 | 4.76 | 0.041 | 0.444 | 0.76 |
| H2AFJ | 445.85 | 182.57 | 586.96 | 197.69 | 4.84 | 0.040 | 0.445 | 0.76 |
| ARHGAP27 | 163.21 | 23.01 | 214.91 | 34.54 | 5.53 | 0.031 | 0.445 | 0.76 |
| ADHFE1 | 109.53 | 7.16 | 144.30 | 17.79 | 4.45 | 0.047 | 0.450 | 0.76 |
| RBM6 | 717.43 | 335.50 | 946.06 | 383.96 | 7.26 | 0.018 | 0.451 | 0.76 |
| TDRD9 | 114.01 | 5.25 | 150.40 | 17.54 | 4.35 | 0.049 | 0.452 | 0.76 |
| FAM13A | 111.70 | 6.08 | 147.39 | 2.02 | 7.70 | 0.016 | 0.448 | 0.76 |
| ZNF137 | 143.14 | 19.22 | 188.88 | 4.92 | 5.11 | 0.036 | 0.447 | 0.76 |
| IL6R | 111.73 | 8.23 | 147.44 | 19.31 | 5.55 | 0.031 | 0.444 | 0.76 |
| RNF217 | 97.46 | 1.77 | 128.65 | 11.44 | 5.55 | 0.031 | 0.445 | 0.76 |
| RP2 | 199.70 | 53.44 | 263.70 | 57.25 | 4.99 | 0.038 | 0.447 | 0.76 |
| MSR1 | 111.45 | 9.43 | 147.18 | 7.71 | 8.03 | 0.015 | 0.453 | 0.76 |
| ALDH8A1 | 132.31 | 4.80 | 174.75 | 3.49 | 8.88 | 0.012 | 0.456 | 0.76 |
| ATP11C | 167.37 | 16.72 | 221.09 | 24.50 | 8.14 | 0.015 | 0.451 | 0.76 |
| HSPBAP1 | 194.92 | 51.44 | 257.52 | 56.24 | 6.26 | 0.025 | 0.449 | 0.76 |
| GABBR1 | 138.47 | 15.66 | 182.99 | 11.60 | 9.39 | 0.011 | 0.462 | 0.76 |
| ARL6IP5 | 3485.65 | 1322.53 | 4606.34 | 1696.10 | 5.07 | 0.037 | 0.446 | 0.76 |
| SP110 | 185.75 | 19.96 | 245.48 | 9.42 | 4.81 | 0.041 | 0.444 | 0.76 |
| GRIN3A | 119.77 | 5.09 | 158.30 | 10.86 | 7.49 | 0.017 | 0.448 | 0.76 |
| C19ORF10 | 608.02 | 222.62 | 804.31 | 242.30 | 4.48 | 0.046 | 0.448 | 0.76 |
| RP5-1022P6.2 | 199.95 | 66.06 | 264.56 | 86.87 | 4.76 | 0.041 | 0.444 | 0.76 |
| TNS1 | 120.25 | 5.20 | 159.13 | 14.83 | 6.57 | 0.022 | 0.454 | 0.76 |
| SLC14A1 | 109.87 | 10.72 | 145.55 | 15.96 | 10.31 | 0.009 | 0.453 | 0.75 |
| PHF11 | 1648.02 | 758.37 | 2184.47 | 695.64 | 5.80 | 0.028 | 0.444 | 0.75 |
| TMEM19 | 249.56 | 55.96 | 331.06 | 60.95 | 13.50 | 0.005 | 0.445 | 0.75 |
| FBXL16 | 114.19 | 14.11 | 151.50 | 15.31 | 53.00 | 0.000 | 0.623 | 0.75 |
| PRKAG2 | 180.72 | 42.42 | 240.07 | 46.55 | 13.90 | 0.005 | 0.451 | 0.75 |
| LYSMD2 | 1468.26 | 747.84 | 1950.51 | 765.11 | 5.15 | 0.036 | 0.447 | 0.75 |
| LRRC25 | 113.45 | 12.72 | 150.74 | 18.52 | 5.63 | 0.030 | 0.446 | 0.75 |
| RREB1 | 122.21 | 10.25 | 162.40 | 1.68 | 5.85 | 0.028 | 0.446 | 0.75 |
| DUSP8 | 124.96 | 5.94 | 166.09 | 14.83 | 7.69 | 0.017 | 0.448 | 0.75 |
| LOC441426 | 100.07 | 3.50 | 133.03 | 8.12 | 9.82 | 0.010 | 0.458 | 0.75 |
| OSBPL5 | 116.08 | 7.84 | 154.33 | 16.63 | 6.57 | 0.022 | 0.453 | 0.75 |
| SAPS2 | 256.80 | 63.22 | 341.44 | 49.99 | 8.01 | 0.015 | 0.452 | 0.75 |
| SLFN13 | 121.14 | 17.10 | 161.10 | 21.54 | 4.81 | 0.041 | 0.444 | 0.75 |
| RHBDF2 | 204.45 | 14.09 | 271.96 | 29.01 | 4.38 | 0.048 | 0.451 | 0.75 |
| ZNF91 | 336.49 | 135.28 | 447.62 | 102.77 | 5.16 | 0.036 | 0.446 | 0.75 |
| SERTAD3 | 216.01 | 60.25 | 287.40 | 77.53 | 6.69 | 0.022 | 0.451 | 0.75 |
| UCRC | 285.31 | 56.84 | 379.60 | 71.86 | 5.14 | 0.036 | 0.446 | 0.75 |
| DAG1 | 183.58 | 35.50 | 244.29 | 41.47 | 14.71 | 0.005 | 0.449 | 0.75 |
| C1ORF66 | 220.87 | 23.42 | 293.95 | 42.25 | 4.94 | 0.039 | 0.446 | 0.75 |
| P4HTM | 287.25 | 93.99 | 382.41 | 120.26 | 5.60 | 0.030 | 0.445 | 0.75 |
| SIGIRR | 162.21 | 16.78 | 216.01 | 7.81 | 6.12 | 0.026 | 0.450 | 0.75 |
| LOC728944 | 122.26 | 9.29 | 162.94 | 13.90 | 4.59 | 0.044 | 0.447 | 0.75 |
| IKBKB | 126.86 | 13.82 | 169.16 | 22.62 | 6.02 | 0.027 | 0.448 | 0.75 |
| ZNF398 | 205.13 | 49.94 | 273.61 | 64.01 | 5.81 | 0.028 | 0.444 | 0.75 |
| DNAJC4 | 198.12 | 20.33 | 264.34 | 18.23 | 16.12 | 0.004 | 0.457 | 0.75 |
| MTMR6 | 388.45 | 165.26 | 518.54 | 206.23 | 5.48 | 0.032 | 0.445 | 0.75 |
| IL1RAP | 126.72 | 12.58 | 169.16 | 10.37 | 16.04 | 0.004 | 0.456 | 0.75 |
| CD46 | 164.32 | 41.07 | 219.62 | 39.97 | 17.92 | 0.003 | 0.445 | 0.75 |
| KLF13 | 1051.09 | 536.00 | 1404.93 | 490.68 | 12.21 | 0.007 | 0.439 | 0.75 |
| PYCARD | 124.46 | 8.09 | 166.36 | 14.92 | 5.64 | 0.030 | 0.447 | 0.75 |
| ZNF701 | 130.53 | 16.38 | 174.51 | 24.68 | 8.64 | 0.013 | 0.451 | 0.75 |
| RTP4 | 144.63 | 23.98 | 193.54 | 18.35 | 6.55 | 0.023 | 0.454 | 0.75 |
| SLC31A1 | 272.43 | 102.96 | 364.63 | 78.48 | 6.46 | 0.023 | 0.453 | 0.75 |
| ZNF615 | 207.61 | 54.00 | 277.96 | 50.29 | 5.57 | 0.031 | 0.445 | 0.75 |
| HS.575696 | 105.84 | 4.20 | 141.73 | 5.40 | 8.48 | 0.014 | 0.452 | 0.75 |
| FAM39DP | 701.45 | 275.96 | 939.39 | 339.46 | 6.16 | 0.025 | 0.448 | 0.75 |
| RGL2 | 141.38 | 17.95 | 189.37 | 19.47 | 7.81 | 0.016 | 0.449 | 0.75 |
| FGFRL1 | 205.98 | 3.36 | 276.00 | 21.41 | 5.04 | 0.037 | 0.447 | 0.75 |
| SIGLEC7 | 116.22 | 5.35 | 155.90 | 6.71 | 39.70 | 0.001 | 0.576 | 0.75 |
| SLFN5 | 125.21 | 9.47 | 167.98 | 19.98 | 7.05 | 0.020 | 0.449 | 0.75 |
| IL10RB | 226.64 | 48.80 | 304.09 | 29.45 | 5.47 | 0.032 | 0.445 | 0.75 |
| ZADH2 | 138.46 | 29.46 | 185.83 | 33.93 | 4.84 | 0.040 | 0.445 | 0.75 |
| C6ORF47 | 276.03 | 82.29 | 370.49 | 87.65 | 29.99 | 0.001 | 0.452 | 0.75 |
| C2CD2 | 178.45 | 20.18 | 239.72 | 40.59 | 5.00 | 0.038 | 0.447 | 0.74 |
| DIDO1 | 175.08 | 68.62 | 235.32 | 57.01 | 4.95 | 0.039 | 0.446 | 0.74 |
| SECTM1 | 112.86 | 6.49 | 151.76 | 9.65 | 5.45 | 0.032 | 0.445 | 0.74 |
| LOC644284 | 126.07 | 23.14 | 169.53 | 37.42 | 5.25 | 0.034 | 0.447 | 0.74 |
| AFF4 | 233.81 | 55.72 | 314.47 | 74.63 | 4.43 | 0.047 | 0.450 | 0.74 |
| RAB4B | 277.46 | 57.10 | 373.25 | 58.78 | 26.20 | 0.001 | 0.446 | 0.74 |
| AGTRAP | 130.62 | 5.11 | 175.84 | 17.12 | 5.14 | 0.036 | 0.446 | 0.74 |
| FAM179B | 157.67 | 36.35 | 212.25 | 53.55 | 4.64 | 0.043 | 0.447 | 0.74 |
| FAM89B | 358.68 | 38.23 | 483.02 | 38.70 | 15.84 | 0.004 | 0.452 | 0.74 |
| SOX8 | 118.44 | 7.75 | 159.51 | 9.51 | 5.22 | 0.035 | 0.447 | 0.74 |
| LOC651524 | 107.98 | 3.29 | 145.63 | 12.94 | 4.97 | 0.038 | 0.446 | 0.74 |
| IL15 | 111.89 | 4.70 | 150.97 | 11.99 | 7.19 | 0.019 | 0.450 | 0.74 |
| GAPT | 159.03 | 35.89 | 214.80 | 27.38 | 8.14 | 0.015 | 0.451 | 0.74 |
| SLC37A1 | 231.15 | 65.61 | 312.28 | 81.09 | 8.05 | 0.015 | 0.454 | 0.74 |
| PITPNC1 | 178.29 | 28.04 | 240.92 | 39.72 | 8.62 | 0.013 | 0.451 | 0.74 |
| LOC645963 | 112.24 | 8.30 | 151.68 | 3.28 | 13.61 | 0.005 | 0.447 | 0.74 |
| MIR2116 | 117.42 | 11.14 | 158.71 | 17.06 | 10.06 | 0.010 | 0.454 | 0.74 |
| ZNF181 | 127.44 | 27.59 | 172.47 | 37.30 | 8.01 | 0.015 | 0.451 | 0.74 |
| CSRNP2 | 196.32 | 51.20 | 265.71 | 65.18 | 8.38 | 0.014 | 0.453 | 0.74 |
| ATP6V1D | 893.53 | 296.01 | 1209.94 | 395.42 | 4.39 | 0.048 | 0.451 | 0.74 |
| CLEC4D | 113.43 | 8.73 | 153.65 | 14.53 | 7.89 | 0.016 | 0.451 | 0.74 |
| DHRS1 | 343.00 | 147.58 | 464.67 | 123.02 | 8.40 | 0.014 | 0.452 | 0.74 |
| CTGLF7 | 716.45 | 156.88 | 970.95 | 221.18 | 6.21 | 0.025 | 0.449 | 0.74 |
| KIF1B | 140.24 | 32.29 | 190.09 | 46.11 | 6.23 | 0.025 | 0.448 | 0.74 |
| ZSWIM4 | 133.23 | 5.62 | 180.67 | 3.73 | 22.54 | 0.002 | 0.450 | 0.74 |
| LOC100128591 | 108.17 | 5.49 | 146.69 | 9.02 | 11.55 | 0.007 | 0.448 | 0.74 |
| CLYBL | 207.82 | 60.75 | 281.93 | 65.58 | 21.35 | 0.002 | 0.449 | 0.74 |
| LGALS9 | 128.89 | 26.21 | 174.91 | 43.51 | 4.58 | 0.044 | 0.448 | 0.74 |
| STX3 | 266.22 | 49.10 | 361.33 | 51.82 | 14.92 | 0.004 | 0.452 | 0.74 |
| HS.440088 | 181.49 | 28.87 | 246.55 | 50.42 | 4.50 | 0.046 | 0.449 | 0.74 |
| PRNP | 1379.44 | 662.19 | 1874.05 | 647.03 | 4.34 | 0.049 | 0.452 | 0.74 |
| HS.130036 | 222.18 | 67.05 | 302.03 | 66.88 | 5.42 | 0.032 | 0.445 | 0.74 |
| CRISPLD2 | 116.09 | 12.85 | 157.81 | 20.42 | 4.76 | 0.041 | 0.444 | 0.74 |
| DEM1 | 131.14 | 14.17 | 178.62 | 28.12 | 5.65 | 0.030 | 0.446 | 0.73 |
| YIPF6 | 327.82 | 132.01 | 446.57 | 142.62 | 13.05 | 0.006 | 0.445 | 0.73 |
| STX5 | 174.70 | 5.64 | 237.99 | 23.03 | 4.82 | 0.040 | 0.445 | 0.73 |
| MAPKAPK2 | 292.12 | 52.39 | 398.06 | 65.05 | 11.90 | 0.007 | 0.444 | 0.73 |
| CSRNP2 | 191.60 | 37.13 | 261.16 | 36.38 | 8.70 | 0.013 | 0.453 | 0.73 |
| LOC100132499 | 507.23 | 199.68 | 691.49 | 217.68 | 15.49 | 0.004 | 0.452 | 0.73 |
| MLL | 133.56 | 6.23 | 182.37 | 20.42 | 5.48 | 0.032 | 0.445 | 0.73 |
| BSDC1 | 524.97 | 237.36 | 716.91 | 220.68 | 10.81 | 0.008 | 0.455 | 0.73 |
| TSPO | 672.30 | 185.04 | 919.14 | 194.42 | 13.49 | 0.005 | 0.443 | 0.73 |
| LOC100133899 | 112.36 | 6.08 | 153.63 | 11.82 | 6.93 | 0.020 | 0.446 | 0.73 |
| HS.125087 | 140.57 | 4.94 | 192.35 | 15.08 | 5.41 | 0.032 | 0.445 | 0.73 |
| KIR2DL4 | 141.28 | 15.81 | 193.48 | 30.93 | 5.19 | 0.035 | 0.447 | 0.73 |
| LOC649864 | 126.09 | 17.44 | 172.73 | 22.31 | 14.68 | 0.005 | 0.444 | 0.73 |
| NOTCH3 | 124.24 | 16.28 | 170.28 | 6.06 | 4.52 | 0.046 | 0.448 | 0.73 |
| OLFML2A | 105.02 | 2.04 | 144.00 | 6.39 | 14.74 | 0.005 | 0.450 | 0.73 |
| SERF2 | 1696.19 | 547.68 | 2326.49 | 660.66 | 9.40 | 0.011 | 0.464 | 0.73 |
| HSPB1 | 2229.19 | 742.73 | 3058.38 | 653.37 | 8.83 | 0.013 | 0.457 | 0.73 |
| C5 | 131.63 | 25.87 | 180.76 | 17.71 | 8.67 | 0.013 | 0.450 | 0.73 |
| VWA5A | 135.95 | 13.96 | 186.70 | 15.76 | 6.50 | 0.023 | 0.452 | 0.73 |
| LOC440776 | 134.33 | 11.43 | 184.48 | 14.60 | 10.39 | 0.009 | 0.454 | 0.73 |
| HIST1H2BK | 153.42 | 18.41 | 210.78 | 30.03 | 7.90 | 0.016 | 0.451 | 0.73 |
| SUCNR1 | 136.04 | 19.63 | 186.93 | 39.00 | 4.48 | 0.046 | 0.448 | 0.73 |
| APOL2 | 141.12 | 18.17 | 193.96 | 32.07 | 4.49 | 0.046 | 0.449 | 0.73 |
| PLCH2 | 116.26 | 5.23 | 159.85 | 9.99 | 5.95 | 0.027 | 0.447 | 0.73 |
| ZNF524 | 188.45 | 35.12 | 259.12 | 31.04 | 6.13 | 0.026 | 0.449 | 0.73 |
| SETX | 287.94 | 59.61 | 395.96 | 89.14 | 4.72 | 0.042 | 0.445 | 0.73 |
| LOC729739 | 115.79 | 15.24 | 159.27 | 4.15 | 5.49 | 0.032 | 0.446 | 0.73 |
| CALHM2 | 159.95 | 7.60 | 220.10 | 10.98 | 7.13 | 0.019 | 0.449 | 0.73 |
| SMAD7 | 228.69 | 46.84 | 314.83 | 21.70 | 5.92 | 0.027 | 0.448 | 0.73 |
| PXN | 121.26 | 8.76 | 167.00 | 9.47 | 14.50 | 0.005 | 0.441 | 0.73 |
| FLJ31306 | 118.32 | 6.17 | 162.95 | 11.41 | 5.03 | 0.037 | 0.447 | 0.73 |
| SLC44A2 | 914.15 | 349.02 | 1259.45 | 315.75 | 5.33 | 0.033 | 0.447 | 0.73 |
| HS.254477 | 120.64 | 13.61 | 166.22 | 29.08 | 5.08 | 0.037 | 0.447 | 0.73 |
| PIK4CA | 1263.42 | 464.62 | 1741.09 | 625.66 | 4.97 | 0.038 | 0.446 | 0.73 |
| KIAA1012 | 762.24 | 424.88 | 1050.43 | 358.42 | 7.48 | 0.017 | 0.448 | 0.73 |
| HIST2H2AC | 352.91 | 145.02 | 486.43 | 168.74 | 6.02 | 0.027 | 0.448 | 0.73 |
| AMT | 131.09 | 6.38 | 180.71 | 23.72 | 4.82 | 0.040 | 0.445 | 0.73 |
| ZNF32 | 195.39 | 28.37 | 269.38 | 43.50 | 8.46 | 0.014 | 0.453 | 0.73 |
| LOC401623 | 114.70 | 5.20 | 158.15 | 9.31 | 12.01 | 0.007 | 0.446 | 0.73 |
| MNT | 299.12 | 102.01 | 412.44 | 117.74 | 7.29 | 0.018 | 0.450 | 0.73 |
| P2RY14 | 111.32 | 7.12 | 153.55 | 12.04 | 11.26 | 0.008 | 0.456 | 0.73 |
| ANKRA2 | 307.62 | 110.76 | 424.92 | 105.19 | 7.00 | 0.020 | 0.449 | 0.72 |
| SMAD5 | 217.78 | 68.28 | 300.92 | 87.33 | 6.03 | 0.026 | 0.449 | 0.72 |
| LOC124512 | 860.35 | 414.11 | 1188.85 | 372.31 | 4.49 | 0.046 | 0.449 | 0.72 |
| LOC338799 | 109.14 | 6.45 | 150.82 | 12.86 | 8.11 | 0.015 | 0.453 | 0.72 |
| BTBD11 | 116.40 | 4.39 | 160.90 | 19.18 | 4.53 | 0.045 | 0.449 | 0.72 |
| HNRNPH2 | 161.66 | 23.17 | 223.55 | 40.91 | 5.22 | 0.035 | 0.446 | 0.72 |
| BCL9L | 123.77 | 8.76 | 171.22 | 11.98 | 4.57 | 0.045 | 0.448 | 0.72 |
| NBPF3 | 303.58 | 100.91 | 420.17 | 135.40 | 5.84 | 0.028 | 0.446 | 0.72 |
| COBLL1 | 666.33 | 298.01 | 922.85 | 331.29 | 5.08 | 0.037 | 0.446 | 0.72 |
| MAP3K7IP2 | 157.91 | 17.99 | 218.78 | 34.65 | 4.94 | 0.039 | 0.446 | 0.72 |
| LOC653853 | 125.57 | 2.92 | 174.04 | 7.38 | 16.00 | 0.004 | 0.454 | 0.72 |
| ETV7 | 150.58 | 34.29 | 208.74 | 27.20 | 5.86 | 0.028 | 0.445 | 0.72 |
| BRWD2 | 335.22 | 118.55 | 464.71 | 100.77 | 7.20 | 0.019 | 0.450 | 0.72 |
| IGF2R | 2199.77 | 783.93 | 3050.69 | 1002.33 | 6.66 | 0.022 | 0.452 | 0.72 |
| FBXO11 | 478.40 | 172.12 | 663.46 | 200.74 | 4.57 | 0.045 | 0.448 | 0.72 |
| TMEM111 | 925.46 | 504.07 | 1284.62 | 459.96 | 7.38 | 0.018 | 0.449 | 0.72 |
| ALDOA | 294.10 | 48.67 | 408.26 | 66.76 | 4.50 | 0.046 | 0.449 | 0.72 |
| LOC389386 | 336.46 | 118.47 | 467.39 | 130.52 | 15.22 | 0.004 | 0.447 | 0.72 |
| AHNAK | 121.49 | 19.84 | 168.81 | 28.81 | 7.41 | 0.018 | 0.449 | 0.72 |
| PRKCD | 1321.42 | 503.22 | 1837.01 | 545.18 | 4.97 | 0.038 | 0.446 | 0.72 |
| AP2S1 | 4913.15 | 1285.26 | 6831.53 | 1356.06 | 14.00 | 0.005 | 0.449 | 0.72 |
| LOC650128 | 184.55 | 48.78 | 256.68 | 53.67 | 16.89 | 0.003 | 0.452 | 0.72 |
| LOC92017 | 109.94 | 11.50 | 152.92 | 3.37 | 7.72 | 0.016 | 0.448 | 0.72 |
| LOC100134083 | 129.66 | 7.51 | 180.38 | 24.32 | 5.01 | 0.038 | 0.446 | 0.72 |
| TSC1 | 444.12 | 192.10 | 618.44 | 191.50 | 4.58 | 0.044 | 0.448 | 0.72 |
| KIAA0174 | 1095.77 | 504.03 | 1526.24 | 551.60 | 14.25 | 0.005 | 0.448 | 0.72 |
| SERINC2 | 110.68 | 9.49 | 154.22 | 14.25 | 15.82 | 0.004 | 0.450 | 0.72 |
| CNOT8 | 189.38 | 43.19 | 263.98 | 54.37 | 4.65 | 0.043 | 0.447 | 0.72 |
| CAPN12 | 137.37 | 21.34 | 191.48 | 39.44 | 4.59 | 0.044 | 0.447 | 0.72 |
| DRAP1 | 2114.78 | 728.65 | 2947.85 | 873.49 | 7.65 | 0.017 | 0.447 | 0.72 |
| CCDC93 | 304.22 | 105.95 | 424.08 | 102.46 | 15.24 | 0.004 | 0.447 | 0.72 |
| DUSP18 | 159.87 | 29.24 | 222.87 | 33.23 | 6.20 | 0.025 | 0.449 | 0.72 |
| PDCD6IP | 263.35 | 65.01 | 367.15 | 78.88 | 9.38 | 0.011 | 0.462 | 0.72 |
| RNF135 | 253.34 | 37.79 | 353.26 | 59.71 | 6.30 | 0.024 | 0.450 | 0.72 |
| PHF1 | 142.41 | 5.79 | 198.61 | 15.61 | 9.87 | 0.010 | 0.458 | 0.72 |
| TNK2 | 658.74 | 172.27 | 918.81 | 184.02 | 5.73 | 0.029 | 0.445 | 0.72 |
| LOC100129539 | 218.30 | 51.80 | 304.67 | 24.42 | 5.45 | 0.032 | 0.445 | 0.72 |
| TAX1BP1 | 826.24 | 343.49 | 1153.22 | 370.83 | 19.59 | 0.003 | 0.456 | 0.72 |
| FLJ21865 | 156.60 | 29.27 | 218.68 | 49.39 | 4.68 | 0.043 | 0.447 | 0.72 |
| LOC641989 | 111.38 | 5.24 | 155.54 | 3.99 | 18.28 | 0.003 | 0.454 | 0.72 |
| LOC654191 | 167.41 | 14.57 | 233.82 | 28.61 | 7.61 | 0.017 | 0.446 | 0.72 |
| OGFR | 369.36 | 11.40 | 515.98 | 50.45 | 4.35 | 0.049 | 0.452 | 0.72 |
| GARNL4 | 106.20 | 12.43 | 148.39 | 19.49 | 10.29 | 0.009 | 0.452 | 0.72 |
| LOC88523 | 214.58 | 63.70 | 299.92 | 53.02 | 6.22 | 0.025 | 0.449 | 0.72 |
| HS.580229 | 112.02 | 6.52 | 156.59 | 12.90 | 5.34 | 0.033 | 0.446 | 0.72 |
| LOC146517 | 257.19 | 72.27 | 359.83 | 62.79 | 4.36 | 0.049 | 0.451 | 0.71 |
| LOC282997 | 116.32 | 1.99 | 162.76 | 14.83 | 5.59 | 0.031 | 0.445 | 0.71 |
| SLC12A6 | 213.10 | 61.36 | 298.32 | 90.62 | 4.73 | 0.042 | 0.445 | 0.71 |
| TMEM134 | 265.36 | 27.85 | 371.50 | 34.68 | 26.11 | 0.001 | 0.438 | 0.71 |
| CR2 | 145.27 | 6.49 | 203.52 | 13.92 | 12.37 | 0.006 | 0.444 | 0.71 |
| HS.5724 | 198.62 | 37.01 | 278.28 | 45.11 | 4.69 | 0.043 | 0.446 | 0.71 |
| SYS1 | 235.37 | 61.38 | 329.88 | 49.71 | 12.23 | 0.007 | 0.440 | 0.71 |
| USPL1 | 411.37 | 174.56 | 576.65 | 169.78 | 42.43 | 0.001 | 0.596 | 0.71 |
| RGL3 | 98.88 | 6.70 | 138.67 | 7.80 | 6.09 | 0.026 | 0.450 | 0.71 |
| CHMP7 | 151.15 | 25.78 | 212.04 | 42.69 | 4.66 | 0.043 | 0.448 | 0.71 |
| KIAA1267 | 341.25 | 95.07 | 478.75 | 100.80 | 32.98 | 0.001 | 0.493 | 0.71 |
| SLC41A2 | 145.96 | 24.87 | 204.77 | 22.65 | 12.34 | 0.006 | 0.442 | 0.71 |
| KIAA1545 | 128.93 | 13.16 | 180.89 | 11.97 | 7.19 | 0.019 | 0.450 | 0.71 |
| FAS | 306.93 | 141.32 | 430.94 | 148.25 | 9.10 | 0.012 | 0.457 | 0.71 |
| PIP3-E | 160.58 | 37.30 | 225.52 | 58.75 | 4.62 | 0.044 | 0.447 | 0.71 |
| AKAP13 | 153.19 | 27.85 | 215.26 | 30.61 | 38.15 | 0.001 | 0.515 | 0.71 |
| PVRL2 | 104.28 | 2.23 | 146.54 | 4.49 | 14.04 | 0.005 | 0.451 | 0.71 |
| C9ORF45 | 182.79 | 38.88 | 256.91 | 16.69 | 5.63 | 0.030 | 0.446 | 0.71 |
| TDRD7 | 405.53 | 143.63 | 570.36 | 189.97 | 5.89 | 0.028 | 0.446 | 0.71 |
| B2M | 16776.95 | 4329.28 | 23600.54 | 5665.43 | 5.83 | 0.028 | 0.445 | 0.71 |
| HS.4988 | 307.06 | 88.61 | 431.97 | 114.94 | 7.46 | 0.018 | 0.448 | 0.71 |
| NLRC4 | 111.98 | 8.14 | 157.58 | 17.70 | 6.49 | 0.023 | 0.452 | 0.71 |
| TTC21A | 101.21 | 6.41 | 142.47 | 2.16 | 10.28 | 0.009 | 0.452 | 0.71 |
| TADA2B | 195.07 | 61.50 | 274.72 | 59.87 | 7.15 | 0.019 | 0.450 | 0.71 |
| ZNF185 | 123.47 | 7.12 | 173.95 | 20.50 | 4.74 | 0.042 | 0.445 | 0.71 |
| HLA-C | 137.43 | 3.94 | 193.63 | 22.58 | 4.78 | 0.041 | 0.444 | 0.71 |
| FZD1 | 123.27 | 11.95 | 173.73 | 8.32 | 20.11 | 0.002 | 0.450 | 0.71 |
| PRKCA | 225.25 | 77.78 | 317.76 | 92.50 | 8.09 | 0.015 | 0.452 | 0.71 |
| DYNLT3 | 385.13 | 171.49 | 543.36 | 150.49 | 13.03 | 0.006 | 0.444 | 0.71 |
| HS.527071 | 121.95 | 14.58 | 172.09 | 17.99 | 4.81 | 0.041 | 0.444 | 0.71 |
| RALGPS1 | 117.60 | 11.82 | 165.97 | 11.71 | 26.14 | 0.001 | 0.442 | 0.71 |
| SBF2 | 126.51 | 9.83 | 178.59 | 14.63 | 16.69 | 0.004 | 0.454 | 0.71 |
| ATXN1 | 201.48 | 35.24 | 284.72 | 53.45 | 7.05 | 0.020 | 0.449 | 0.71 |
| WDFY1 | 1268.63 | 466.39 | 1793.62 | 612.10 | 5.79 | 0.029 | 0.444 | 0.71 |
| SIGLEC7 | 110.11 | 5.11 | 155.78 | 9.32 | 7.95 | 0.015 | 0.453 | 0.71 |
| MAP3K2 | 240.70 | 61.05 | 340.72 | 81.65 | 5.91 | 0.027 | 0.448 | 0.71 |
| FLJ45337 | 122.16 | 7.25 | 173.01 | 10.75 | 18.76 | 0.003 | 0.455 | 0.71 |
| YPEL4 | 100.72 | 7.85 | 142.69 | 18.98 | 4.35 | 0.049 | 0.452 | 0.71 |
| TAOK3 | 202.50 | 43.03 | 286.91 | 65.81 | 4.83 | 0.040 | 0.445 | 0.71 |
| OSTM1 | 184.23 | 41.05 | 261.04 | 45.16 | 7.17 | 0.019 | 0.450 | 0.71 |
| RANBP6 | 208.08 | 51.08 | 294.90 | 53.41 | 12.45 | 0.006 | 0.445 | 0.71 |
| LOC441773 | 111.50 | 7.51 | 158.04 | 23.99 | 4.86 | 0.040 | 0.444 | 0.71 |
| UBXN11 | 142.98 | 12.86 | 202.69 | 2.05 | 6.94 | 0.020 | 0.447 | 0.71 |
| INPP5K | 161.18 | 22.99 | 228.51 | 24.57 | 10.31 | 0.009 | 0.453 | 0.71 |
| EPB41L5 | 135.63 | 22.19 | 192.31 | 16.01 | 13.17 | 0.006 | 0.444 | 0.71 |
| ROBO3 | 129.69 | 2.79 | 183.92 | 21.88 | 4.91 | 0.039 | 0.445 | 0.71 |
| SYVN1 | 693.13 | 312.25 | 983.41 | 407.44 | 5.10 | 0.036 | 0.446 | 0.70 |
| CLN3 | 173.74 | 25.88 | 246.57 | 54.04 | 4.45 | 0.047 | 0.450 | 0.70 |
| GDI1 | 333.02 | 72.38 | 472.87 | 96.88 | 4.90 | 0.039 | 0.444 | 0.70 |
| TMEM8 | 168.20 | 31.57 | 239.00 | 47.82 | 6.35 | 0.024 | 0.452 | 0.70 |
| TAX1BP1 | 948.09 | 420.62 | 1347.58 | 453.38 | 20.76 | 0.002 | 0.439 | 0.70 |
| LRRC25 | 106.07 | 7.52 | 150.78 | 20.27 | 4.42 | 0.048 | 0.450 | 0.70 |
| TRPV2 | 412.22 | 146.97 | 585.97 | 185.46 | 7.00 | 0.020 | 0.448 | 0.70 |
| MAN1B1 | 254.87 | 114.81 | 362.33 | 88.15 | 4.90 | 0.039 | 0.444 | 0.70 |
| JARID1D | 258.70 | 97.58 | 367.78 | 104.80 | 11.00 | 0.008 | 0.456 | 0.70 |
| BAIAP2 | 116.68 | 15.03 | 166.02 | 6.45 | 4.92 | 0.039 | 0.445 | 0.70 |
| NUCB1 | 4259.69 | 1406.01 | 6066.24 | 1332.98 | 40.97 | 0.001 | 0.586 | 0.70 |
| ZNF493 | 130.19 | 14.38 | 185.72 | 26.88 | 6.32 | 0.024 | 0.451 | 0.70 |
| EDEM3 | 202.73 | 45.16 | 289.43 | 78.55 | 4.49 | 0.046 | 0.448 | 0.70 |
| CENTD2 | 165.72 | 23.73 | 236.64 | 26.34 | 8.67 | 0.013 | 0.450 | 0.70 |
| KIAA0494 | 702.23 | 300.00 | 1002.75 | 250.25 | 4.40 | 0.048 | 0.450 | 0.70 |
| SH3PXD2A | 166.16 | 39.54 | 237.32 | 48.67 | 12.42 | 0.006 | 0.443 | 0.70 |
| LATS2 | 115.24 | 8.22 | 164.74 | 16.10 | 6.40 | 0.024 | 0.453 | 0.70 |
| SLC6A16 | 118.47 | 6.40 | 169.36 | 18.74 | 5.60 | 0.030 | 0.445 | 0.70 |
| KRCC1 | 252.70 | 89.55 | 361.39 | 110.56 | 8.52 | 0.013 | 0.452 | 0.70 |
| MGC52000 | 360.88 | 100.45 | 516.44 | 139.10 | 5.75 | 0.029 | 0.445 | 0.70 |
| TMEM63A | 135.73 | 9.20 | 194.25 | 12.89 | 4.97 | 0.038 | 0.446 | 0.70 |
| SIL1 | 189.61 | 44.53 | 271.65 | 48.86 | 5.12 | 0.036 | 0.447 | 0.70 |
| AKAP13 | 196.51 | 38.41 | 281.80 | 38.06 | 6.03 | 0.026 | 0.448 | 0.70 |
| RTN3 | 314.24 | 62.04 | 450.67 | 71.40 | 12.71 | 0.006 | 0.440 | 0.70 |
| C17ORF91 | 156.97 | 42.27 | 225.23 | 62.89 | 5.47 | 0.032 | 0.445 | 0.70 |
| DNAH1 | 137.68 | 18.94 | 197.64 | 28.98 | 8.58 | 0.013 | 0.450 | 0.70 |
| LOC648984 | 132.44 | 13.76 | 190.28 | 22.05 | 10.93 | 0.008 | 0.454 | 0.70 |
| BLCAP | 246.88 | 34.23 | 354.73 | 31.26 | 8.66 | 0.013 | 0.450 | 0.70 |
| TSPAN32 | 119.38 | 12.48 | 171.72 | 8.96 | 21.71 | 0.002 | 0.446 | 0.70 |
| CENTD2 | 114.31 | 18.98 | 164.50 | 36.06 | 4.49 | 0.046 | 0.448 | 0.69 |
| FAM39DP | 200.79 | 37.99 | 289.12 | 36.38 | 9.34 | 0.011 | 0.462 | 0.69 |
| APOB48R | 124.14 | 10.38 | 178.77 | 6.63 | 13.31 | 0.006 | 0.446 | 0.69 |
| GABBR1 | 127.18 | 11.14 | 183.21 | 18.46 | 6.39 | 0.024 | 0.453 | 0.69 |
| RASSF4 | 141.07 | 32.32 | 203.27 | 31.96 | 8.64 | 0.013 | 0.451 | 0.69 |
| TRIM56 | 260.44 | 91.27 | 375.45 | 76.10 | 12.34 | 0.007 | 0.442 | 0.69 |
| KCNMB1 | 109.29 | 9.02 | 157.54 | 11.68 | 31.46 | 0.001 | 0.482 | 0.69 |
| RRBP1 | 695.48 | 349.26 | 1002.90 | 372.13 | 4.43 | 0.047 | 0.450 | 0.69 |
| LOC388681 | 126.12 | 13.58 | 181.90 | 8.77 | 5.51 | 0.031 | 0.446 | 0.69 |
| MAT2B | 3573.20 | 2113.68 | 5155.30 | 1741.92 | 5.61 | 0.030 | 0.445 | 0.69 |
| NEK8 | 149.15 | 20.53 | 215.19 | 15.38 | 11.57 | 0.007 | 0.448 | 0.69 |
| LOC100131850 | 126.05 | 11.10 | 181.89 | 10.40 | 4.57 | 0.045 | 0.448 | 0.69 |
| ZBTB48 | 279.99 | 100.01 | 404.03 | 99.07 | 11.66 | 0.007 | 0.450 | 0.69 |
| C11ORF67 | 156.46 | 33.25 | 225.84 | 40.64 | 7.60 | 0.017 | 0.446 | 0.69 |
| LOC728744 | 107.88 | 12.22 | 155.94 | 1.69 | 7.90 | 0.016 | 0.451 | 0.69 |
| INPP5A | 160.32 | 13.51 | 231.89 | 36.20 | 5.19 | 0.035 | 0.447 | 0.69 |
| TNIK | 114.23 | 9.71 | 165.35 | 6.84 | 11.83 | 0.007 | 0.446 | 0.69 |
| LCOR | 214.10 | 70.66 | 309.94 | 64.49 | 24.15 | 0.002 | 0.437 | 0.69 |
| DLGAP4 | 192.80 | 40.12 | 279.10 | 37.86 | 8.96 | 0.012 | 0.454 | 0.69 |
| SLC30A1 | 185.06 | 28.35 | 267.91 | 37.86 | 14.51 | 0.005 | 0.442 | 0.69 |
| PPP2R5B | 118.69 | 6.92 | 171.83 | 19.11 | 4.65 | 0.043 | 0.448 | 0.69 |
| HS.443185 | 112.65 | 9.06 | 163.31 | 24.37 | 5.24 | 0.035 | 0.447 | 0.69 |
| ADORA2B | 114.02 | 12.81 | 165.31 | 28.28 | 5.11 | 0.036 | 0.447 | 0.69 |
| ISCU | 1259.05 | 496.14 | 1827.01 | 516.26 | 9.53 | 0.011 | 0.464 | 0.69 |
| LOC221442 | 132.13 | 4.19 | 191.97 | 8.63 | 8.33 | 0.014 | 0.452 | 0.69 |
| MBNL2 | 315.88 | 171.86 | 458.98 | 180.57 | 24.98 | 0.002 | 0.429 | 0.69 |
| SAT2 | 335.39 | 107.64 | 487.65 | 134.04 | 7.78 | 0.016 | 0.448 | 0.69 |
| C9ORF103 | 168.73 | 17.60 | 245.35 | 10.66 | 13.80 | 0.005 | 0.450 | 0.69 |
| ARRDC5 | 114.40 | 10.65 | 166.37 | 21.94 | 5.88 | 0.028 | 0.446 | 0.69 |
| PYHIN1 | 139.76 | 12.11 | 203.29 | 35.35 | 4.71 | 0.042 | 0.446 | 0.69 |
| GPR108 | 188.56 | 19.69 | 274.28 | 27.93 | 7.03 | 0.020 | 0.449 | 0.69 |
| FAM131A | 194.80 | 24.20 | 283.45 | 53.30 | 4.46 | 0.047 | 0.450 | 0.69 |
| TMEM38B | 320.40 | 139.53 | 466.25 | 103.34 | 4.68 | 0.043 | 0.447 | 0.69 |
| MFSD1 | 1351.14 | 616.49 | 1966.86 | 647.06 | 9.08 | 0.012 | 0.456 | 0.69 |
| POFUT2 | 231.17 | 56.33 | 336.65 | 65.05 | 12.88 | 0.006 | 0.438 | 0.69 |
| KLF6 | 1751.48 | 836.78 | 2552.42 | 846.05 | 41.34 | 0.001 | 0.601 | 0.69 |
| ZC3H11B | 982.62 | 429.00 | 1432.08 | 577.51 | 4.75 | 0.042 | 0.445 | 0.69 |
| DGCR2 | 203.17 | 16.12 | 296.37 | 34.22 | 6.52 | 0.023 | 0.453 | 0.69 |
| NR3C2 | 119.57 | 12.32 | 174.44 | 24.15 | 5.12 | 0.036 | 0.447 | 0.69 |
| SPATS2L | 252.03 | 62.73 | 367.69 | 71.17 | 11.78 | 0.007 | 0.448 | 0.69 |
| EVI2B | 118.62 | 2.19 | 173.09 | 18.87 | 4.54 | 0.045 | 0.449 | 0.69 |
| RNASEL | 243.49 | 70.29 | 355.31 | 100.53 | 6.40 | 0.024 | 0.454 | 0.69 |
| HLA-A | 14408.31 | 3914.15 | 21030.19 | 4687.53 | 13.96 | 0.005 | 0.449 | 0.69 |
| HAMP | 114.08 | 8.01 | 166.56 | 3.64 | 9.89 | 0.010 | 0.458 | 0.68 |
| HS.445414 | 134.50 | 12.31 | 196.64 | 31.72 | 5.26 | 0.034 | 0.448 | 0.68 |
| GOLGB1 | 209.66 | 85.53 | 306.61 | 102.64 | 9.78 | 0.010 | 0.457 | 0.68 |
| PNPLA6 | 673.75 | 191.53 | 986.11 | 241.52 | 4.52 | 0.046 | 0.448 | 0.68 |
| VAC14 | 260.44 | 38.20 | 381.65 | 51.42 | 5.96 | 0.027 | 0.448 | 0.68 |
| LOC100132593 | 23711.13 | 8135.11 | 34754.51 | 6902.09 | 13.65 | 0.005 | 0.446 | 0.68 |
| BMPR2 | 143.54 | 17.46 | 210.40 | 32.99 | 6.55 | 0.023 | 0.454 | 0.68 |
| LILRB1 | 158.07 | 26.76 | 231.71 | 35.07 | 9.19 | 0.012 | 0.456 | 0.68 |
| SIL1 | 460.32 | 183.45 | 675.12 | 255.59 | 4.92 | 0.039 | 0.445 | 0.68 |
| PCDHB19P | 144.43 | 11.82 | 211.94 | 19.06 | 5.88 | 0.028 | 0.446 | 0.68 |
| PLCL1 | 117.06 | 2.14 | 171.85 | 16.59 | 5.89 | 0.028 | 0.446 | 0.68 |
| GAPT | 181.74 | 36.64 | 267.01 | 60.07 | 6.08 | 0.026 | 0.449 | 0.68 |
| AES | 1117.07 | 81.05 | 1641.48 | 141.91 | 7.00 | 0.020 | 0.449 | 0.68 |
| MST1 | 133.15 | 14.83 | 195.75 | 21.81 | 12.00 | 0.007 | 0.445 | 0.68 |
| TAP2 | 781.69 | 293.79 | 1149.27 | 320.29 | 18.34 | 0.003 | 0.457 | 0.68 |
| ABHD2 | 153.48 | 17.54 | 225.92 | 33.68 | 5.98 | 0.027 | 0.447 | 0.68 |
| TMEM179B | 292.76 | 19.81 | 431.00 | 53.89 | 6.84 | 0.021 | 0.448 | 0.68 |
| SLC30A1 | 177.25 | 49.43 | 261.00 | 30.43 | 7.44 | 0.018 | 0.449 | 0.68 |
| S100A16 | 108.93 | 14.20 | 160.42 | 26.25 | 6.74 | 0.021 | 0.448 | 0.68 |
| LOC644353 | 134.90 | 8.43 | 198.69 | 13.63 | 13.66 | 0.005 | 0.446 | 0.68 |
| DSC2 | 118.00 | 8.86 | 173.84 | 27.35 | 5.18 | 0.035 | 0.447 | 0.68 |
| PTTG1IP | 395.80 | 134.74 | 583.18 | 128.72 | 4.43 | 0.047 | 0.450 | 0.68 |
| METRNL | 161.56 | 4.88 | 238.13 | 17.20 | 9.03 | 0.012 | 0.455 | 0.68 |
| LOC642787 | 129.78 | 12.93 | 191.42 | 36.82 | 4.47 | 0.047 | 0.449 | 0.68 |
| TOM1 | 526.99 | 265.66 | 777.37 | 253.02 | 12.97 | 0.006 | 0.441 | 0.68 |
| LOC100131905 | 371.95 | 159.38 | 549.12 | 194.79 | 8.44 | 0.014 | 0.452 | 0.68 |
| PFDN5 | 2997.61 | 1529.20 | 4425.76 | 1314.63 | 11.40 | 0.008 | 0.452 | 0.68 |
| CORO1B | 401.64 | 204.62 | 593.28 | 197.26 | 9.58 | 0.011 | 0.462 | 0.68 |
| TXNRD1 | 712.42 | 336.98 | 1052.86 | 315.60 | 10.65 | 0.009 | 0.453 | 0.68 |
| MGC72104 | 296.83 | 87.46 | 438.70 | 107.07 | 6.14 | 0.025 | 0.448 | 0.68 |
| MCL1 | 845.63 | 412.22 | 1251.11 | 525.43 | 5.96 | 0.027 | 0.448 | 0.68 |
| ACADVL | 410.17 | 111.20 | 607.40 | 113.83 | 32.72 | 0.001 | 0.484 | 0.68 |
| C4ORF34 | 877.35 | 437.62 | 1299.36 | 340.74 | 6.77 | 0.021 | 0.449 | 0.68 |
| PLEKHB2 | 266.72 | 130.22 | 395.44 | 115.74 | 13.51 | 0.005 | 0.446 | 0.67 |
| FBXO3 | 133.72 | 19.99 | 198.42 | 11.71 | 8.06 | 0.015 | 0.453 | 0.67 |
| NPTN | 2203.79 | 1046.71 | 3272.33 | 1264.88 | 4.84 | 0.040 | 0.444 | 0.67 |
| ANXA2 | 405.41 | 149.20 | 602.32 | 126.98 | 13.21 | 0.006 | 0.445 | 0.67 |
| LOC154761 | 149.63 | 18.88 | 222.32 | 37.64 | 6.54 | 0.023 | 0.454 | 0.67 |
| LRRC37B | 286.49 | 116.21 | 425.77 | 113.45 | 5.90 | 0.028 | 0.447 | 0.67 |
| UBQLN2 | 776.34 | 475.30 | 1154.00 | 463.35 | 26.44 | 0.001 | 0.450 | 0.67 |
| NCOA4 | 4367.58 | 2068.40 | 6495.63 | 2327.92 | 6.11 | 0.026 | 0.450 | 0.67 |
| MYD88 | 308.80 | 62.75 | 459.51 | 58.21 | 34.63 | 0.001 | 0.525 | 0.67 |
| APOBEC3F | 270.91 | 61.83 | 403.16 | 52.08 | 6.08 | 0.026 | 0.449 | 0.67 |
| ATG12 | 487.76 | 183.72 | 725.95 | 192.25 | 6.19 | 0.025 | 0.449 | 0.67 |
| LOC648622 | 3261.04 | 1315.80 | 4856.99 | 1394.34 | 12.66 | 0.006 | 0.440 | 0.67 |
| RCSD1 | 385.66 | 94.81 | 574.91 | 98.59 | 6.32 | 0.024 | 0.451 | 0.67 |
| DYNLT1 | 810.64 | 478.24 | 1210.53 | 385.53 | 5.30 | 0.034 | 0.447 | 0.67 |
| KIAA1267 | 764.27 | 302.39 | 1141.35 | 392.17 | 6.44 | 0.023 | 0.453 | 0.67 |
| RAX2 | 144.10 | 16.40 | 215.27 | 24.42 | 6.04 | 0.026 | 0.448 | 0.67 |
| LOC200030 | 723.26 | 247.56 | 1080.70 | 352.51 | 5.39 | 0.033 | 0.445 | 0.67 |
| CDC2L6 | 278.27 | 51.83 | 415.86 | 69.95 | 12.74 | 0.006 | 0.439 | 0.67 |
| CIAO1 | 622.74 | 261.68 | 931.07 | 292.05 | 6.99 | 0.020 | 0.448 | 0.67 |
| ADPGK | 579.20 | 227.04 | 867.03 | 286.42 | 8.40 | 0.014 | 0.452 | 0.67 |
| HS.531457 | 130.38 | 22.06 | 195.22 | 25.82 | 5.84 | 0.028 | 0.445 | 0.67 |
| LOC100132526 | 138.09 | 11.43 | 206.83 | 22.32 | 10.89 | 0.008 | 0.456 | 0.67 |
| ZNF302 | 633.06 | 286.65 | 949.03 | 355.64 | 6.91 | 0.020 | 0.447 | 0.67 |
| LOC150223 | 127.88 | 3.32 | 191.90 | 14.23 | 9.35 | 0.011 | 0.463 | 0.67 |
| FUCA2 | 212.46 | 38.10 | 318.84 | 68.05 | 6.14 | 0.026 | 0.449 | 0.67 |
| SLC2A9 | 113.79 | 2.68 | 170.82 | 16.95 | 6.10 | 0.026 | 0.450 | 0.67 |
| SHC1 | 552.29 | 208.92 | 829.30 | 226.52 | 4.83 | 0.040 | 0.444 | 0.67 |
| SBNO2 | 272.90 | 10.35 | 410.14 | 15.73 | 9.22 | 0.012 | 0.457 | 0.67 |
| HS.409512 | 117.65 | 9.93 | 176.87 | 16.56 | 15.37 | 0.004 | 0.451 | 0.67 |
| C9ORF109 | 117.66 | 4.82 | 176.93 | 11.55 | 8.90 | 0.012 | 0.456 | 0.66 |
| POLD4 | 198.18 | 27.49 | 298.08 | 61.02 | 5.03 | 0.037 | 0.447 | 0.66 |
| MBP | 187.11 | 28.66 | 281.54 | 34.69 | 5.38 | 0.033 | 0.445 | 0.66 |
| LOC100131967 | 126.14 | 4.27 | 190.02 | 22.09 | 5.79 | 0.029 | 0.444 | 0.66 |
| CYB561D1 | 134.38 | 15.84 | 202.46 | 16.53 | 5.02 | 0.037 | 0.447 | 0.66 |
| RERE | 211.73 | 31.74 | 319.28 | 37.41 | 5.24 | 0.035 | 0.447 | 0.66 |
| PATL2 | 230.37 | 82.77 | 347.68 | 59.46 | 5.47 | 0.032 | 0.445 | 0.66 |
| SAMD3 | 124.47 | 15.54 | 188.08 | 25.67 | 6.79 | 0.021 | 0.448 | 0.66 |
| C14ORF159 | 467.17 | 102.19 | 706.03 | 143.11 | 8.70 | 0.013 | 0.453 | 0.66 |
| NAPRT1 | 245.33 | 72.89 | 371.18 | 73.61 | 31.06 | 0.001 | 0.475 | 0.66 |
| SENP7 | 221.15 | 76.83 | 334.97 | 85.51 | 5.82 | 0.028 | 0.445 | 0.66 |
| NCRNA00085 | 125.76 | 21.00 | 190.53 | 29.19 | 13.43 | 0.006 | 0.442 | 0.66 |
| CMIP | 1057.45 | 256.82 | 1602.86 | 278.35 | 15.00 | 0.004 | 0.450 | 0.66 |
| TCL1B | 113.32 | 5.69 | 171.79 | 18.89 | 6.99 | 0.020 | 0.448 | 0.66 |
| LOC645489 | 149.57 | 24.83 | 226.78 | 40.88 | 4.32 | 0.050 | 0.452 | 0.66 |
| LOC100132395 | 131.57 | 14.79 | 199.50 | 22.09 | 6.60 | 0.022 | 0.453 | 0.66 |
| SIK3 | 637.58 | 294.63 | 966.88 | 276.23 | 14.81 | 0.005 | 0.454 | 0.66 |
| MZF1 | 253.67 | 116.23 | 384.94 | 111.18 | 7.09 | 0.019 | 0.450 | 0.66 |
| USP6NL | 212.40 | 42.02 | 322.38 | 85.34 | 4.39 | 0.048 | 0.451 | 0.66 |
| IDH1 | 669.79 | 349.36 | 1017.40 | 482.11 | 4.44 | 0.047 | 0.450 | 0.66 |
| MBP | 217.25 | 38.17 | 330.43 | 45.47 | 8.44 | 0.014 | 0.451 | 0.66 |
| GLG1 | 747.74 | 356.09 | 1137.93 | 344.99 | 5.32 | 0.034 | 0.447 | 0.66 |
| LOC642333 | 829.62 | 372.32 | 1263.73 | 390.09 | 19.08 | 0.003 | 0.446 | 0.66 |
| JAK2 | 364.93 | 155.68 | 556.03 | 138.47 | 7.02 | 0.020 | 0.449 | 0.66 |
| POLR3GL | 552.51 | 258.28 | 842.29 | 324.95 | 5.28 | 0.034 | 0.447 | 0.66 |
| LOC727948 | 123.88 | 5.05 | 188.86 | 28.69 | 4.70 | 0.042 | 0.447 | 0.66 |
| APP | 195.88 | 24.52 | 298.65 | 19.45 | 34.61 | 0.001 | 0.518 | 0.66 |
| IKBKE | 211.81 | 61.57 | 323.09 | 69.07 | 5.39 | 0.033 | 0.445 | 0.66 |
| CD47 | 587.65 | 300.83 | 896.61 | 313.47 | 21.80 | 0.002 | 0.453 | 0.66 |
| FAM113A | 229.61 | 59.85 | 350.34 | 96.04 | 4.57 | 0.045 | 0.448 | 0.66 |
| PPP2R2B | 241.82 | 56.10 | 369.18 | 92.50 | 5.08 | 0.037 | 0.447 | 0.66 |
| LOC388237 | 118.31 | 10.97 | 180.64 | 21.31 | 6.47 | 0.023 | 0.453 | 0.65 |
| GLB1 | 657.08 | 215.84 | 1003.53 | 263.89 | 8.27 | 0.014 | 0.452 | 0.65 |
| TGIF1 | 270.10 | 55.60 | 412.55 | 70.53 | 6.70 | 0.022 | 0.450 | 0.65 |
| LAMP2 | 1160.46 | 533.53 | 1772.54 | 713.32 | 5.87 | 0.028 | 0.445 | 0.65 |
| PRCP | 1820.59 | 642.66 | 2781.39 | 630.36 | 10.98 | 0.008 | 0.456 | 0.65 |
| GPR109A | 138.70 | 22.60 | 211.95 | 8.18 | 8.72 | 0.013 | 0.455 | 0.65 |
| PARP6 | 209.98 | 32.50 | 320.99 | 66.65 | 5.61 | 0.030 | 0.445 | 0.65 |
| AGAP3 | 165.10 | 14.14 | 252.75 | 35.17 | 5.04 | 0.037 | 0.447 | 0.65 |
| ZSWIM6 | 261.56 | 95.39 | 400.70 | 115.78 | 7.99 | 0.015 | 0.452 | 0.65 |
| ETV6 | 489.81 | 209.61 | 750.93 | 185.08 | 11.60 | 0.007 | 0.448 | 0.65 |
| ZCWPW1 | 178.50 | 27.17 | 273.68 | 42.52 | 6.87 | 0.021 | 0.446 | 0.65 |
| PHKA2 | 257.17 | 90.76 | 394.51 | 66.81 | 6.56 | 0.022 | 0.454 | 0.65 |
| PICALM | 1007.64 | 474.17 | 1546.31 | 442.96 | 6.66 | 0.022 | 0.452 | 0.65 |
| DPM3 | 235.17 | 73.49 | 361.01 | 94.16 | 6.53 | 0.023 | 0.453 | 0.65 |
| ABCB1 | 110.49 | 11.90 | 169.64 | 22.47 | 6.71 | 0.021 | 0.450 | 0.65 |
| LGALS3BP | 171.03 | 20.73 | 262.62 | 8.92 | 5.39 | 0.033 | 0.445 | 0.65 |
| FYCO1 | 158.35 | 18.73 | 243.21 | 52.02 | 4.38 | 0.048 | 0.451 | 0.65 |
| MGAT4A | 170.15 | 18.30 | 261.34 | 50.47 | 4.65 | 0.043 | 0.447 | 0.65 |
| LOC642975 | 1217.96 | 604.44 | 1871.89 | 736.21 | 7.94 | 0.016 | 0.452 | 0.65 |
| TRAFD1 | 483.67 | 69.41 | 744.71 | 166.92 | 4.57 | 0.045 | 0.448 | 0.65 |
| RPS6KA5 | 180.27 | 19.08 | 277.62 | 41.07 | 5.75 | 0.029 | 0.445 | 0.65 |
| NCSTN | 758.39 | 312.64 | 1168.13 | 377.87 | 5.89 | 0.028 | 0.446 | 0.65 |
| PFDN5 | 3596.13 | 1244.77 | 5541.54 | 1801.42 | 5.83 | 0.028 | 0.445 | 0.65 |
| TMBIM6 | 4293.38 | 2191.76 | 6617.03 | 1912.81 | 12.33 | 0.007 | 0.439 | 0.65 |
| FAM129C | 152.25 | 30.65 | 234.82 | 36.68 | 18.66 | 0.003 | 0.451 | 0.65 |
| MTMR3 | 435.87 | 114.39 | 672.25 | 148.66 | 4.83 | 0.040 | 0.444 | 0.65 |
| PLEKHM1 | 174.50 | 29.85 | 269.27 | 64.54 | 4.42 | 0.048 | 0.450 | 0.65 |
| C6ORF225 | 157.63 | 22.84 | 243.26 | 37.74 | 9.43 | 0.011 | 0.463 | 0.65 |
| KIAA0182 | 172.20 | 20.22 | 265.82 | 51.42 | 4.68 | 0.043 | 0.447 | 0.65 |
| P2RY14 | 108.88 | 5.37 | 168.33 | 14.00 | 11.67 | 0.007 | 0.450 | 0.65 |
| FAR2 | 164.19 | 43.36 | 253.88 | 69.94 | 5.08 | 0.037 | 0.446 | 0.65 |
| HLA-DMA | 3213.82 | 195.83 | 4969.49 | 570.04 | 7.46 | 0.018 | 0.448 | 0.65 |
| H1FX | 226.09 | 62.01 | 349.62 | 66.77 | 7.69 | 0.016 | 0.448 | 0.65 |
| TAPBPL | 208.89 | 42.74 | 323.05 | 52.27 | 5.03 | 0.037 | 0.447 | 0.65 |
| SNTB2 | 232.12 | 98.67 | 359.34 | 110.72 | 13.08 | 0.006 | 0.444 | 0.65 |
| MSRA | 170.29 | 20.29 | 263.74 | 29.77 | 10.67 | 0.009 | 0.453 | 0.65 |
| RNF149 | 152.83 | 22.33 | 236.70 | 44.66 | 5.62 | 0.030 | 0.445 | 0.65 |
| ACADVL | 2430.22 | 1126.61 | 3764.70 | 1082.43 | 5.77 | 0.029 | 0.444 | 0.65 |
| REEP5 | 865.69 | 393.68 | 1341.37 | 398.74 | 8.12 | 0.015 | 0.452 | 0.65 |
| LOC151162 | 1213.39 | 290.88 | 1881.96 | 450.89 | 7.23 | 0.019 | 0.450 | 0.64 |
| DDX58 | 122.66 | 10.10 | 190.51 | 21.68 | 5.61 | 0.030 | 0.445 | 0.64 |
| ANGEL1 | 180.38 | 19.05 | 280.29 | 37.37 | 8.74 | 0.013 | 0.455 | 0.64 |
| CDC42BPB | 156.99 | 19.16 | 244.25 | 8.84 | 8.46 | 0.014 | 0.452 | 0.64 |
| TMSB10 | 12648.69 | 2944.15 | 19701.96 | 4975.40 | 5.05 | 0.037 | 0.447 | 0.64 |
| MSL3L1 | 327.36 | 146.56 | 510.26 | 161.60 | 20.99 | 0.002 | 0.446 | 0.64 |
| LAPTM4A | 233.04 | 69.10 | 363.67 | 109.60 | 5.49 | 0.032 | 0.445 | 0.64 |
| WBP2 | 3297.79 | 867.91 | 5146.55 | 1080.14 | 7.93 | 0.016 | 0.451 | 0.64 |
| HS.571741 | 135.82 | 17.08 | 212.03 | 37.41 | 4.85 | 0.040 | 0.444 | 0.64 |
| TNFSF10 | 422.53 | 136.04 | 660.16 | 185.28 | 7.84 | 0.016 | 0.450 | 0.64 |
| ZBED5 | 668.47 | 342.48 | 1045.44 | 312.43 | 7.33 | 0.018 | 0.450 | 0.64 |
| HERC6 | 182.19 | 4.17 | 284.96 | 40.79 | 4.63 | 0.044 | 0.447 | 0.64 |
| TMEM127 | 151.62 | 19.35 | 237.51 | 45.81 | 5.03 | 0.037 | 0.447 | 0.64 |
| ACVR1 | 524.32 | 229.64 | 821.98 | 282.14 | 9.39 | 0.011 | 0.463 | 0.64 |
| CTDSP1 | 296.77 | 84.47 | 465.70 | 127.30 | 5.15 | 0.036 | 0.447 | 0.64 |
| FLJ45256 | 148.37 | 19.73 | 232.82 | 35.23 | 7.22 | 0.019 | 0.450 | 0.64 |
| RNASEL | 239.43 | 43.56 | 375.99 | 75.39 | 5.34 | 0.033 | 0.446 | 0.64 |
| DDX17 | 473.88 | 207.85 | 745.05 | 296.22 | 4.34 | 0.049 | 0.452 | 0.64 |
| SPG11 | 613.47 | 255.07 | 964.61 | 209.95 | 9.25 | 0.011 | 0.457 | 0.64 |
| SLC25A28 | 972.35 | 452.93 | 1529.41 | 384.66 | 11.58 | 0.007 | 0.448 | 0.64 |
| LAPTM5 | 1323.82 | 300.82 | 2082.39 | 421.42 | 6.44 | 0.023 | 0.453 | 0.64 |
| KMO | 142.86 | 14.71 | 224.90 | 21.10 | 12.98 | 0.006 | 0.441 | 0.64 |
| FLJ35776 | 122.68 | 10.12 | 193.13 | 13.93 | 6.34 | 0.024 | 0.451 | 0.64 |
| MLLT6 | 904.45 | 439.38 | 1424.34 | 610.70 | 5.20 | 0.035 | 0.447 | 0.63 |
| MGC10997 | 1207.58 | 484.60 | 1901.83 | 575.41 | 13.20 | 0.006 | 0.444 | 0.63 |
| UBL3 | 298.00 | 89.17 | 469.78 | 81.78 | 26.14 | 0.001 | 0.440 | 0.63 |
| FAM13B | 499.69 | 218.50 | 787.81 | 223.25 | 50.30 | 0.000 | 0.602 | 0.63 |
| SPIRE1 | 236.60 | 89.73 | 373.31 | 87.09 | 9.05 | 0.012 | 0.455 | 0.63 |
| CR2 | 158.23 | 30.07 | 249.67 | 42.69 | 5.68 | 0.030 | 0.447 | 0.63 |
| SNX30 | 174.59 | 24.55 | 275.53 | 40.85 | 7.23 | 0.019 | 0.450 | 0.63 |
| SQSTM1 | 3524.20 | 854.87 | 5567.98 | 1652.68 | 4.44 | 0.047 | 0.449 | 0.63 |
| PDCD4 | 190.26 | 36.77 | 300.68 | 69.94 | 5.30 | 0.034 | 0.447 | 0.63 |
| ZNF320 | 247.94 | 51.20 | 391.85 | 71.09 | 4.44 | 0.047 | 0.450 | 0.63 |
| PHF1 | 175.67 | 8.95 | 277.79 | 28.25 | 5.97 | 0.027 | 0.447 | 0.63 |
| LRP10 | 934.00 | 379.92 | 1477.66 | 545.29 | 5.18 | 0.035 | 0.447 | 0.63 |
| SNX29 | 177.15 | 23.37 | 280.30 | 38.19 | 9.63 | 0.011 | 0.463 | 0.63 |
| LOC643733 | 131.35 | 11.35 | 208.38 | 24.25 | 9.35 | 0.011 | 0.463 | 0.63 |
| ITGAV | 173.67 | 60.42 | 275.59 | 69.88 | 6.30 | 0.024 | 0.449 | 0.63 |
| PBXIP1 | 109.57 | 5.77 | 173.95 | 19.38 | 6.25 | 0.025 | 0.449 | 0.63 |
| ICAM2 | 336.46 | 100.54 | 534.30 | 127.27 | 8.53 | 0.013 | 0.452 | 0.63 |
| RNASE6 | 130.17 | 18.20 | 206.88 | 12.93 | 4.31 | 0.050 | 0.452 | 0.63 |
| HS.559602 | 119.10 | 7.45 | 189.47 | 11.13 | 6.58 | 0.022 | 0.454 | 0.63 |
| LOC54103 | 130.76 | 8.42 | 208.05 | 16.82 | 15.74 | 0.004 | 0.451 | 0.63 |
| ZNF467 | 104.32 | 3.90 | 165.99 | 27.21 | 4.38 | 0.048 | 0.451 | 0.63 |
| ST3GAL5 | 183.71 | 28.77 | 293.05 | 44.33 | 5.82 | 0.028 | 0.444 | 0.63 |
| LOC728650 | 128.88 | 11.23 | 205.73 | 24.29 | 9.09 | 0.012 | 0.456 | 0.63 |
| MTF1 | 409.25 | 135.96 | 653.83 | 204.57 | 4.42 | 0.048 | 0.450 | 0.63 |
| C1S | 128.62 | 1.19 | 205.62 | 26.61 | 5.23 | 0.035 | 0.448 | 0.63 |
| IDUA | 115.48 | 15.84 | 184.86 | 24.49 | 12.14 | 0.007 | 0.442 | 0.62 |
| ATP6V0A1 | 641.19 | 231.08 | 1026.46 | 297.87 | 9.31 | 0.011 | 0.461 | 0.62 |
| CCR6 | 245.91 | 91.69 | 393.78 | 112.21 | 9.00 | 0.012 | 0.455 | 0.62 |
| PPP2R3A | 108.36 | 8.86 | 173.57 | 11.57 | 7.21 | 0.019 | 0.450 | 0.62 |
| PARP10 | 561.98 | 242.42 | 900.21 | 272.59 | 9.28 | 0.011 | 0.459 | 0.62 |
| SLC2A3 | 2431.19 | 920.13 | 3894.60 | 1238.53 | 5.86 | 0.028 | 0.445 | 0.62 |
| HS.436134 | 247.54 | 84.28 | 396.92 | 41.98 | 5.42 | 0.032 | 0.445 | 0.62 |
| MS4A7 | 122.02 | 5.50 | 195.69 | 20.85 | 8.30 | 0.014 | 0.452 | 0.62 |
| IRS2 | 131.33 | 10.12 | 210.76 | 17.72 | 13.20 | 0.006 | 0.444 | 0.62 |
| ZNF264 | 177.35 | 42.41 | 284.63 | 46.78 | 41.37 | 0.001 | 0.613 | 0.62 |
| LOC401622 | 130.95 | 6.14 | 210.35 | 26.70 | 6.31 | 0.024 | 0.450 | 0.62 |
| FAM102A | 155.76 | 22.25 | 250.20 | 56.93 | 4.71 | 0.042 | 0.446 | 0.62 |
| FPR2 | 114.16 | 3.91 | 183.50 | 17.41 | 8.84 | 0.013 | 0.457 | 0.62 |
| B3GALT4 | 112.56 | 13.48 | 181.42 | 13.89 | 19.30 | 0.003 | 0.453 | 0.62 |
| TRIB1 | 479.20 | 210.32 | 772.49 | 263.54 | 7.04 | 0.020 | 0.449 | 0.62 |
| CHST12 | 153.42 | 32.08 | 247.53 | 22.10 | 5.89 | 0.028 | 0.446 | 0.62 |
| HSCB | 407.69 | 112.33 | 657.99 | 146.39 | 4.88 | 0.040 | 0.444 | 0.62 |
| ZNF559 | 207.56 | 48.57 | 335.00 | 61.59 | 11.40 | 0.008 | 0.452 | 0.62 |
| DCUN1D3 | 176.65 | 29.27 | 285.27 | 59.39 | 4.31 | 0.050 | 0.452 | 0.62 |
| THBS3 | 151.15 | 24.05 | 244.09 | 46.03 | 6.88 | 0.020 | 0.446 | 0.62 |
| DSE | 613.89 | 371.92 | 991.50 | 327.62 | 4.41 | 0.048 | 0.450 | 0.62 |
| SERINC1 | 511.83 | 157.45 | 827.09 | 257.77 | 4.97 | 0.038 | 0.446 | 0.62 |
| FCGR1B | 108.20 | 7.98 | 175.03 | 24.80 | 4.76 | 0.041 | 0.444 | 0.62 |
| LOC100133019 | 226.52 | 54.13 | 366.58 | 74.86 | 6.32 | 0.024 | 0.451 | 0.62 |
| EPAS1 | 158.24 | 62.09 | 256.53 | 65.85 | 4.56 | 0.045 | 0.449 | 0.62 |
| HS.407903 | 163.84 | 22.54 | 265.95 | 42.72 | 5.78 | 0.029 | 0.444 | 0.62 |
| CD27 | 349.20 | 136.25 | 567.01 | 170.01 | 6.11 | 0.026 | 0.450 | 0.62 |
| MVP | 298.00 | 56.36 | 484.02 | 50.09 | 24.35 | 0.002 | 0.434 | 0.62 |
| TMEM87A | 1434.27 | 589.46 | 2331.77 | 494.17 | 8.29 | 0.014 | 0.452 | 0.62 |
| NAGPA | 361.39 | 85.64 | 588.10 | 84.64 | 21.74 | 0.002 | 0.447 | 0.61 |
| ITGB1 | 331.32 | 124.17 | 539.38 | 88.31 | 8.99 | 0.012 | 0.455 | 0.61 |
| RBM33 | 262.64 | 96.81 | 427.67 | 124.58 | 5.32 | 0.034 | 0.447 | 0.61 |
| PDE7A | 196.41 | 59.29 | 320.21 | 25.49 | 5.48 | 0.032 | 0.445 | 0.61 |
| CLK4 | 179.67 | 47.79 | 293.10 | 80.30 | 5.98 | 0.027 | 0.448 | 0.61 |
| LTB | 900.61 | 390.88 | 1470.56 | 521.90 | 7.38 | 0.018 | 0.449 | 0.61 |
| DNASE2 | 203.85 | 60.97 | 332.90 | 70.58 | 6.94 | 0.020 | 0.447 | 0.61 |
| KIR2DL3 | 134.54 | 18.40 | 219.92 | 49.34 | 4.75 | 0.042 | 0.445 | 0.61 |
| ZNF131 | 166.79 | 38.75 | 272.69 | 48.97 | 4.53 | 0.046 | 0.449 | 0.61 |
| CYB561D1 | 266.98 | 98.22 | 436.58 | 133.62 | 8.27 | 0.014 | 0.452 | 0.61 |
| MBP | 1027.25 | 471.28 | 1679.87 | 560.17 | 6.45 | 0.023 | 0.453 | 0.61 |
| LOC100132323 | 141.27 | 22.35 | 231.02 | 42.64 | 4.43 | 0.047 | 0.450 | 0.61 |
| KLHL28 | 150.76 | 25.59 | 246.56 | 52.84 | 5.99 | 0.027 | 0.448 | 0.61 |
| PDE4B | 712.21 | 293.02 | 1164.81 | 292.91 | 5.16 | 0.036 | 0.447 | 0.61 |
| HS.541829 | 143.89 | 20.82 | 235.36 | 45.92 | 4.81 | 0.041 | 0.444 | 0.61 |
| DUSP6 | 125.67 | 12.87 | 205.60 | 40.24 | 4.97 | 0.038 | 0.446 | 0.61 |
| SUMF1 | 176.34 | 37.34 | 288.84 | 80.02 | 4.56 | 0.045 | 0.448 | 0.61 |
| LEMD3 | 542.67 | 219.34 | 888.99 | 288.54 | 5.58 | 0.031 | 0.445 | 0.61 |
| IFI35 | 961.37 | 377.91 | 1576.11 | 228.64 | 5.72 | 0.029 | 0.446 | 0.61 |
| ATXN1 | 206.35 | 73.05 | 338.33 | 111.55 | 5.80 | 0.028 | 0.444 | 0.61 |
| ZNF83 | 192.64 | 68.21 | 315.89 | 96.73 | 7.35 | 0.018 | 0.450 | 0.61 |
| ZBTB20 | 125.11 | 18.75 | 205.29 | 35.44 | 7.74 | 0.016 | 0.449 | 0.61 |
| ALPK1 | 301.57 | 79.16 | 495.18 | 110.14 | 8.61 | 0.013 | 0.451 | 0.61 |
| PTTG1IP | 1216.89 | 624.45 | 2001.19 | 701.55 | 5.18 | 0.035 | 0.447 | 0.61 |
| CIITA | 251.13 | 81.62 | 413.05 | 104.39 | 8.94 | 0.012 | 0.455 | 0.61 |
| OPLAH | 127.29 | 21.39 | 209.41 | 33.00 | 12.07 | 0.007 | 0.443 | 0.61 |
| HLA-E | 3857.52 | 975.28 | 6353.88 | 1205.22 | 15.13 | 0.004 | 0.448 | 0.61 |
| VPS36 | 334.57 | 138.53 | 551.49 | 186.03 | 5.48 | 0.032 | 0.445 | 0.61 |
| TUBGCP6 | 289.37 | 84.55 | 477.23 | 87.30 | 7.19 | 0.019 | 0.450 | 0.61 |
| ATP6V0C | 200.90 | 42.03 | 331.53 | 64.09 | 6.02 | 0.026 | 0.448 | 0.61 |
| GORASP1 | 237.43 | 38.77 | 392.28 | 53.36 | 12.56 | 0.006 | 0.441 | 0.61 |
| GLTSCR2 | 5742.81 | 2248.61 | 9498.83 | 3234.93 | 4.83 | 0.040 | 0.444 | 0.60 |
| LAMA5 | 170.82 | 33.94 | 282.77 | 56.87 | 4.79 | 0.041 | 0.444 | 0.60 |
| GSTK1 | 1553.94 | 637.89 | 2574.76 | 538.68 | 4.32 | 0.050 | 0.452 | 0.60 |
| NUAK2 | 143.86 | 11.18 | 238.62 | 13.58 | 16.44 | 0.004 | 0.454 | 0.60 |
| GAA | 133.31 | 12.43 | 221.36 | 6.61 | 8.16 | 0.015 | 0.451 | 0.60 |
| TYMP | 141.81 | 32.33 | 235.48 | 35.33 | 7.62 | 0.017 | 0.445 | 0.60 |
| AUTS2 | 177.13 | 20.72 | 294.18 | 38.64 | 9.45 | 0.011 | 0.465 | 0.60 |
| LOC100132510 | 144.58 | 22.65 | 240.14 | 58.62 | 4.51 | 0.046 | 0.449 | 0.60 |
| ARRB1 | 153.99 | 13.19 | 256.03 | 26.96 | 9.32 | 0.011 | 0.462 | 0.60 |
| SGSH | 231.01 | 32.21 | 384.11 | 22.85 | 8.37 | 0.014 | 0.452 | 0.60 |
| PRCP | 623.46 | 213.03 | 1037.68 | 356.80 | 4.98 | 0.038 | 0.447 | 0.60 |
| N4BP2L1 | 190.44 | 53.42 | 316.98 | 96.39 | 4.66 | 0.043 | 0.448 | 0.60 |
| FCGR2B | 133.07 | 18.03 | 221.73 | 23.91 | 9.29 | 0.011 | 0.460 | 0.60 |
| CALU | 330.06 | 77.93 | 549.98 | 163.50 | 4.45 | 0.047 | 0.450 | 0.60 |
| GCA | 599.42 | 350.07 | 999.13 | 402.10 | 7.44 | 0.018 | 0.448 | 0.60 |
| RIPK3 | 141.37 | 23.73 | 235.74 | 50.97 | 6.00 | 0.027 | 0.448 | 0.60 |
| CHD9 | 514.02 | 69.85 | 857.26 | 113.39 | 13.60 | 0.005 | 0.446 | 0.60 |
| FLJ20489 | 136.26 | 5.64 | 227.33 | 10.30 | 16.23 | 0.004 | 0.458 | 0.60 |
| PPARD | 162.71 | 9.14 | 271.53 | 44.19 | 5.30 | 0.034 | 0.447 | 0.60 |
| GPX8 | 137.17 | 20.57 | 229.17 | 53.20 | 4.70 | 0.042 | 0.447 | 0.60 |
| FAM177A1 | 19238.67 | 6612.06 | 32143.32 | 5401.29 | 7.48 | 0.017 | 0.448 | 0.60 |
| TSPAN31 | 288.97 | 99.38 | 483.02 | 163.67 | 5.10 | 0.036 | 0.447 | 0.60 |
| FAIM3 | 1501.05 | 260.84 | 2510.34 | 329.06 | 23.19 | 0.002 | 0.457 | 0.60 |
| TAPT1 | 200.06 | 24.82 | 334.83 | 30.08 | 14.59 | 0.005 | 0.442 | 0.60 |
| OSBPL2 | 214.25 | 51.34 | 359.28 | 76.30 | 9.72 | 0.010 | 0.459 | 0.60 |
| IQSEC1 | 218.96 | 46.52 | 367.75 | 73.38 | 7.97 | 0.015 | 0.452 | 0.60 |
| IFITM1 | 866.89 | 219.64 | 1456.21 | 304.69 | 12.00 | 0.007 | 0.445 | 0.60 |
| TMEM156 | 470.06 | 163.47 | 790.51 | 120.17 | 4.42 | 0.048 | 0.450 | 0.59 |
| LOC728953 | 198.66 | 37.53 | 334.25 | 51.49 | 7.76 | 0.016 | 0.449 | 0.59 |
| VPS28 | 519.96 | 167.53 | 875.95 | 229.57 | 9.94 | 0.010 | 0.456 | 0.59 |
| C20ORF108 | 126.97 | 11.03 | 214.40 | 44.50 | 4.52 | 0.046 | 0.449 | 0.59 |
| C3AR1 | 128.84 | 10.38 | 217.83 | 17.10 | 6.21 | 0.025 | 0.449 | 0.59 |
| HS.137971 | 578.55 | 220.98 | 978.77 | 312.38 | 5.11 | 0.036 | 0.447 | 0.59 |
| CKAP4 | 212.79 | 36.44 | 360.11 | 35.58 | 12.80 | 0.006 | 0.439 | 0.59 |
| FCGR3A | 126.49 | 20.93 | 214.34 | 22.83 | 7.96 | 0.015 | 0.452 | 0.59 |
| DCLRE1C | 146.55 | 38.12 | 248.43 | 39.84 | 4.66 | 0.043 | 0.448 | 0.59 |
| TRIM52 | 172.42 | 65.13 | 292.43 | 73.51 | 4.68 | 0.043 | 0.447 | 0.59 |
| LOC100131787 | 481.18 | 130.01 | 816.19 | 242.65 | 5.10 | 0.036 | 0.446 | 0.59 |
| TRIM13 | 180.37 | 29.93 | 306.56 | 48.84 | 7.76 | 0.016 | 0.450 | 0.59 |
| ANXA2 | 170.74 | 37.43 | 290.33 | 59.81 | 9.08 | 0.012 | 0.456 | 0.59 |
| LOC374395 | 1355.12 | 614.59 | 2304.66 | 543.20 | 7.54 | 0.017 | 0.446 | 0.59 |
| ANXA2P2 | 20779.51 | 6049.43 | 35414.79 | 3984.27 | 6.21 | 0.025 | 0.449 | 0.59 |
| HEXDC | 202.74 | 56.77 | 345.84 | 76.49 | 12.35 | 0.006 | 0.443 | 0.59 |
| GAA | 117.22 | 10.56 | 200.03 | 15.36 | 14.34 | 0.005 | 0.445 | 0.59 |
| PPP1R11 | 1252.39 | 558.75 | 2138.09 | 532.78 | 6.69 | 0.022 | 0.451 | 0.59 |
| VIM | 6173.66 | 1868.14 | 10540.94 | 1589.98 | 5.28 | 0.034 | 0.447 | 0.59 |
| SESN1 | 148.46 | 23.25 | 254.13 | 41.10 | 5.45 | 0.032 | 0.445 | 0.58 |
| DENND4C | 235.22 | 40.82 | 402.70 | 74.12 | 8.46 | 0.014 | 0.453 | 0.58 |
| TGIF1 | 451.91 | 139.70 | 774.14 | 165.26 | 7.66 | 0.017 | 0.447 | 0.58 |
| TREM2 | 116.00 | 5.10 | 199.02 | 9.14 | 12.15 | 0.007 | 0.443 | 0.58 |
| FCRL2 | 142.28 | 20.89 | 244.51 | 40.02 | 5.30 | 0.034 | 0.447 | 0.58 |
| CUTL1 | 168.41 | 36.49 | 289.55 | 57.11 | 7.98 | 0.015 | 0.452 | 0.58 |
| C21ORF2 | 160.43 | 30.04 | 276.10 | 51.53 | 4.33 | 0.049 | 0.452 | 0.58 |
| RERE | 445.10 | 133.53 | 768.61 | 147.75 | 7.27 | 0.018 | 0.450 | 0.58 |
| ZNF438 | 159.62 | 13.68 | 275.68 | 45.17 | 5.92 | 0.027 | 0.448 | 0.58 |
| IQGAP2 | 202.78 | 49.54 | 350.99 | 53.80 | 9.42 | 0.011 | 0.464 | 0.58 |
| LOC100133465 | 19857.16 | 6422.13 | 34407.29 | 6133.11 | 7.93 | 0.016 | 0.452 | 0.58 |
| PCNX | 368.23 | 70.45 | 638.07 | 52.46 | 12.31 | 0.007 | 0.440 | 0.58 |
| TAGLN | 143.47 | 30.81 | 248.65 | 43.38 | 13.82 | 0.005 | 0.450 | 0.58 |
| MANBA | 244.77 | 92.28 | 424.23 | 104.08 | 5.62 | 0.030 | 0.446 | 0.58 |
| ABHD3 | 218.26 | 51.42 | 378.39 | 65.86 | 5.45 | 0.032 | 0.445 | 0.58 |
| IRAK3 | 159.13 | 11.52 | 276.23 | 50.03 | 5.24 | 0.035 | 0.447 | 0.58 |
| NT5C2 | 616.66 | 283.71 | 1071.34 | 358.60 | 5.46 | 0.032 | 0.445 | 0.58 |
| ARRDC2 | 172.08 | 35.47 | 298.97 | 63.63 | 6.81 | 0.021 | 0.448 | 0.58 |
| ABCB1 | 158.76 | 20.75 | 276.16 | 21.91 | 32.74 | 0.001 | 0.489 | 0.57 |
| ETS1 | 1599.62 | 963.29 | 2783.23 | 1196.74 | 8.71 | 0.013 | 0.454 | 0.57 |
| PLCB2 | 212.08 | 35.71 | 369.17 | 92.57 | 4.74 | 0.042 | 0.445 | 0.57 |
| HS.576106 | 247.22 | 43.47 | 430.65 | 64.47 | 6.35 | 0.024 | 0.452 | 0.57 |
| FLJ38717 | 203.55 | 45.19 | 355.19 | 37.75 | 6.34 | 0.024 | 0.451 | 0.57 |
| RAB20 | 125.40 | 6.43 | 218.84 | 28.51 | 5.91 | 0.027 | 0.448 | 0.57 |
| MGAT1 | 948.12 | 177.29 | 1654.65 | 211.60 | 11.18 | 0.008 | 0.456 | 0.57 |
| ABR | 815.49 | 388.65 | 1426.08 | 489.13 | 7.92 | 0.016 | 0.452 | 0.57 |
| ZBTB42 | 176.01 | 41.48 | 307.79 | 51.38 | 21.77 | 0.002 | 0.450 | 0.57 |
| NUDT14 | 196.56 | 26.12 | 343.84 | 60.76 | 6.57 | 0.022 | 0.454 | 0.57 |
| TNFAIP2 | 124.16 | 16.82 | 217.29 | 26.93 | 6.55 | 0.023 | 0.455 | 0.57 |
| TCN2 | 120.33 | 9.86 | 210.59 | 22.45 | 5.50 | 0.031 | 0.446 | 0.57 |
| NUB1 | 720.00 | 194.68 | 1260.13 | 275.77 | 5.99 | 0.027 | 0.448 | 0.57 |
| HNMT | 153.03 | 11.93 | 267.99 | 47.61 | 5.58 | 0.031 | 0.445 | 0.57 |
| ZNF219 | 127.32 | 4.82 | 223.71 | 33.65 | 5.41 | 0.033 | 0.445 | 0.57 |
| TAX1BP3 | 301.11 | 99.69 | 529.19 | 139.48 | 9.71 | 0.010 | 0.459 | 0.57 |
| UBA7 | 568.37 | 120.40 | 998.92 | 203.90 | 5.72 | 0.029 | 0.445 | 0.57 |
| TMEM59 | 1345.35 | 510.56 | 2364.53 | 673.57 | 8.60 | 0.013 | 0.451 | 0.57 |
| LOC729439 | 19751.13 | 8267.77 | 34723.93 | 3713.74 | 5.15 | 0.036 | 0.446 | 0.57 |
| SLC25A20 | 143.54 | 17.28 | 252.52 | 43.33 | 6.48 | 0.023 | 0.452 | 0.57 |
| SSH3 | 114.75 | 14.06 | 201.90 | 36.52 | 6.30 | 0.024 | 0.450 | 0.57 |
| ZBP1 | 133.83 | 3.07 | 235.52 | 11.60 | 14.52 | 0.005 | 0.443 | 0.57 |
| SLAMF6 | 736.70 | 334.99 | 1297.83 | 353.80 | 32.36 | 0.001 | 0.490 | 0.57 |
| DMXL2 | 136.24 | 9.03 | 240.17 | 40.78 | 5.37 | 0.033 | 0.446 | 0.57 |
| GDPD5 | 136.41 | 20.74 | 240.69 | 44.13 | 7.36 | 0.018 | 0.449 | 0.57 |
| LRPAP1 | 343.41 | 81.08 | 605.98 | 118.93 | 6.52 | 0.023 | 0.453 | 0.57 |
| PTGS2 | 118.23 | 13.50 | 208.75 | 41.94 | 4.52 | 0.046 | 0.449 | 0.57 |
| APLP2 | 160.39 | 16.58 | 283.40 | 36.13 | 7.67 | 0.017 | 0.447 | 0.57 |
| MSH3 | 18570.66 | 6949.33 | 32839.74 | 6000.83 | 5.02 | 0.037 | 0.446 | 0.57 |
| LOC728661 | 1207.45 | 675.24 | 2137.42 | 776.33 | 8.32 | 0.014 | 0.452 | 0.56 |
| GK | 482.57 | 142.16 | 855.09 | 208.34 | 7.20 | 0.019 | 0.450 | 0.56 |
| ITGB1 | 669.51 | 389.38 | 1189.49 | 464.18 | 7.94 | 0.015 | 0.452 | 0.56 |
| LOC391169 | 349.30 | 139.89 | 620.71 | 158.91 | 4.82 | 0.041 | 0.445 | 0.56 |
| GPR109B | 124.43 | 30.94 | 221.23 | 33.83 | 5.00 | 0.038 | 0.447 | 0.56 |
| RHEB | 984.09 | 335.67 | 1750.85 | 534.36 | 4.60 | 0.044 | 0.448 | 0.56 |
| SLC11A2 | 285.61 | 104.82 | 508.16 | 152.59 | 6.09 | 0.026 | 0.449 | 0.56 |
| HPCAL1 | 1352.50 | 350.88 | 2407.17 | 483.68 | 4.73 | 0.042 | 0.445 | 0.56 |
| FCHO2 | 116.48 | 11.29 | 207.66 | 44.73 | 4.71 | 0.042 | 0.446 | 0.56 |
| ANO6 | 251.79 | 61.96 | 448.96 | 109.74 | 5.63 | 0.030 | 0.446 | 0.56 |
| TMEM30A | 198.08 | 47.96 | 353.23 | 102.13 | 4.94 | 0.039 | 0.446 | 0.56 |
| TGFBR2 | 963.53 | 448.45 | 1719.96 | 486.93 | 8.75 | 0.013 | 0.456 | 0.56 |
| RHBDL2 | 132.10 | 12.83 | 235.81 | 35.71 | 7.84 | 0.016 | 0.450 | 0.56 |
| BAZ2B | 182.87 | 32.46 | 326.54 | 70.74 | 6.24 | 0.025 | 0.449 | 0.56 |
| HLA-DRA | 15401.23 | 4847.01 | 27515.93 | 7954.67 | 6.73 | 0.021 | 0.449 | 0.56 |
| HINT3 | 242.90 | 21.28 | 434.93 | 56.04 | 8.58 | 0.013 | 0.451 | 0.56 |
| HLA-F | 576.66 | 191.56 | 1032.94 | 198.27 | 10.89 | 0.008 | 0.455 | 0.56 |
| CECR6 | 126.13 | 10.22 | 225.97 | 6.93 | 17.93 | 0.003 | 0.448 | 0.56 |
| GBA | 219.75 | 48.46 | 393.73 | 58.19 | 9.75 | 0.010 | 0.459 | 0.56 |
| CCDC92 | 339.81 | 144.59 | 608.91 | 137.79 | 7.68 | 0.017 | 0.448 | 0.56 |
| HLA-H | 7313.05 | 1754.33 | 13108.02 | 1241.67 | 19.09 | 0.003 | 0.447 | 0.56 |
| RHOQ | 1548.31 | 770.06 | 2779.93 | 1009.18 | 8.91 | 0.012 | 0.456 | 0.56 |
| PPM1M | 669.31 | 236.02 | 1202.32 | 365.23 | 5.88 | 0.028 | 0.446 | 0.56 |
| DEF8 | 223.15 | 76.75 | 401.13 | 86.78 | 7.14 | 0.019 | 0.450 | 0.56 |
| LOC649456 | 126.50 | 24.03 | 227.79 | 44.40 | 6.20 | 0.025 | 0.449 | 0.56 |
| SPPL2A | 974.15 | 447.82 | 1755.29 | 536.99 | 7.53 | 0.017 | 0.446 | 0.55 |
| TPMT | 210.70 | 59.62 | 380.06 | 43.19 | 8.01 | 0.015 | 0.452 | 0.55 |
| VCPIP1 | 221.33 | 55.64 | 399.53 | 95.19 | 5.09 | 0.036 | 0.446 | 0.55 |
| PARP14 | 485.61 | 183.29 | 877.00 | 254.78 | 5.89 | 0.028 | 0.446 | 0.55 |
| CD37 | 326.01 | 90.26 | 589.13 | 184.08 | 4.76 | 0.041 | 0.444 | 0.55 |
| HS.202577 | 130.78 | 7.46 | 236.71 | 23.79 | 10.40 | 0.009 | 0.455 | 0.55 |
| PARP11 | 151.13 | 25.03 | 273.83 | 69.11 | 4.76 | 0.041 | 0.444 | 0.55 |
| LOC646936 | 235.21 | 50.29 | 426.35 | 65.77 | 4.89 | 0.039 | 0.444 | 0.55 |
| MX1 | 1929.83 | 1097.81 | 3500.94 | 1272.63 | 15.53 | 0.004 | 0.451 | 0.55 |
| METRNL | 471.87 | 185.34 | 856.71 | 211.65 | 15.62 | 0.004 | 0.453 | 0.55 |
| FTHL8 | 139.23 | 14.98 | 252.88 | 25.65 | 10.68 | 0.009 | 0.453 | 0.55 |
| ETS2 | 190.93 | 40.78 | 346.83 | 62.88 | 6.81 | 0.021 | 0.448 | 0.55 |
| DENND5B | 151.43 | 37.59 | 275.18 | 54.31 | 7.51 | 0.017 | 0.447 | 0.55 |
| DJ341D10.1 | 235.60 | 40.47 | 428.14 | 97.59 | 5.77 | 0.029 | 0.444 | 0.55 |
| FBXO11 | 728.93 | 365.83 | 1325.46 | 423.39 | 4.98 | 0.038 | 0.447 | 0.55 |
| PLEKHO1 | 614.89 | 266.69 | 1118.99 | 395.82 | 6.55 | 0.023 | 0.454 | 0.55 |
| SNORA12 | 181.82 | 37.26 | 330.99 | 4.16 | 7.76 | 0.016 | 0.450 | 0.55 |
| HLA-DPA1 | 5969.26 | 1707.22 | 10871.83 | 2823.36 | 5.22 | 0.035 | 0.446 | 0.55 |
| LOC648059 | 153.33 | 11.31 | 279.51 | 43.42 | 4.73 | 0.042 | 0.445 | 0.55 |
| LILRA5 | 129.93 | 6.19 | 236.88 | 44.07 | 4.47 | 0.047 | 0.449 | 0.55 |
| DNHL1 | 126.90 | 19.64 | 231.60 | 14.09 | 23.67 | 0.002 | 0.445 | 0.55 |
| RPS27 | 6646.64 | 2927.87 | 12136.96 | 3302.36 | 8.56 | 0.013 | 0.451 | 0.55 |
| B2M | 10568.12 | 3398.84 | 19302.10 | 4132.07 | 8.64 | 0.013 | 0.451 | 0.55 |
| IL10RB | 727.77 | 382.53 | 1331.19 | 377.42 | 7.04 | 0.020 | 0.449 | 0.55 |
| CDA | 113.57 | 10.22 | 207.75 | 11.04 | 21.42 | 0.002 | 0.452 | 0.55 |
| ZFYVE16 | 146.80 | 21.61 | 268.60 | 26.94 | 13.81 | 0.005 | 0.451 | 0.55 |
| PEPD | 850.72 | 180.46 | 1558.64 | 321.68 | 5.70 | 0.029 | 0.445 | 0.55 |
| MGC71993 | 884.56 | 172.71 | 1622.13 | 387.66 | 5.25 | 0.034 | 0.448 | 0.55 |
| GSDMB | 181.42 | 50.72 | 332.71 | 84.42 | 7.50 | 0.017 | 0.448 | 0.55 |
| BATF2 | 134.56 | 10.60 | 246.82 | 14.45 | 33.31 | 0.001 | 0.501 | 0.55 |
| SH3TC1 | 159.69 | 22.54 | 293.23 | 55.27 | 6.08 | 0.026 | 0.449 | 0.54 |
| PDE3B | 204.47 | 48.92 | 376.74 | 84.77 | 6.17 | 0.025 | 0.448 | 0.54 |
| HLA-B | 8311.75 | 3026.44 | 15330.13 | 3083.59 | 11.98 | 0.007 | 0.443 | 0.54 |
| SLC36A1 | 154.89 | 12.05 | 286.06 | 41.84 | 5.99 | 0.027 | 0.448 | 0.54 |
| ZNF674 | 17373.92 | 7821.41 | 32103.29 | 5681.59 | 6.31 | 0.024 | 0.451 | 0.54 |
| LOC100129034 | 205.31 | 39.46 | 379.73 | 90.75 | 4.91 | 0.039 | 0.444 | 0.54 |
| FARSLB | 14403.76 | 4686.46 | 26644.14 | 4513.27 | 7.05 | 0.020 | 0.449 | 0.54 |
| FKBP15 | 296.04 | 34.05 | 548.02 | 62.25 | 15.32 | 0.004 | 0.450 | 0.54 |
| ZNFX1 | 351.57 | 138.15 | 651.04 | 194.69 | 5.55 | 0.031 | 0.444 | 0.54 |
| CD74 | 10270.20 | 861.84 | 19044.15 | 1838.56 | 5.64 | 0.030 | 0.446 | 0.54 |
| MOBKL2C | 405.43 | 130.58 | 752.71 | 132.44 | 7.04 | 0.020 | 0.449 | 0.54 |
| VASH1 | 123.89 | 7.29 | 230.10 | 28.99 | 8.45 | 0.014 | 0.452 | 0.54 |
| EGR1 | 319.01 | 32.41 | 592.50 | 62.31 | 8.28 | 0.014 | 0.452 | 0.54 |
| C1ORF85 | 475.76 | 167.17 | 883.74 | 259.06 | 4.47 | 0.047 | 0.449 | 0.54 |
| TULP4 | 176.92 | 41.35 | 329.06 | 84.56 | 4.72 | 0.042 | 0.445 | 0.54 |
| GK | 621.31 | 213.62 | 1156.27 | 257.49 | 9.90 | 0.010 | 0.457 | 0.54 |
| C4ORF18 | 100.97 | 9.55 | 187.92 | 14.12 | 6.96 | 0.020 | 0.447 | 0.54 |
| HLA-H | 1441.39 | 524.34 | 2691.72 | 49.73 | 4.41 | 0.048 | 0.450 | 0.54 |
| LOC100130070 | 1343.90 | 718.50 | 2510.50 | 1089.72 | 4.69 | 0.043 | 0.447 | 0.54 |
| LRP3 | 114.62 | 9.06 | 214.46 | 26.46 | 6.37 | 0.024 | 0.452 | 0.53 |
| PRIC285 | 417.04 | 169.74 | 780.99 | 268.43 | 4.35 | 0.049 | 0.452 | 0.53 |
| ORC6L | 17123.28 | 7700.00 | 32083.59 | 6955.68 | 5.18 | 0.035 | 0.447 | 0.53 |
| DOPEY2 | 545.13 | 183.70 | 1022.15 | 293.85 | 6.55 | 0.023 | 0.454 | 0.53 |
| FCGR2B | 127.40 | 6.87 | 239.02 | 33.82 | 5.12 | 0.036 | 0.447 | 0.53 |
| LPAR1 | 128.73 | 9.46 | 241.70 | 21.58 | 6.31 | 0.024 | 0.450 | 0.53 |
| FOXO3 | 303.63 | 141.18 | 570.51 | 154.98 | 33.46 | 0.001 | 0.502 | 0.53 |
| KIAA1370 | 251.26 | 89.57 | 472.11 | 174.83 | 4.33 | 0.049 | 0.452 | 0.53 |
| IL15 | 177.99 | 35.38 | 334.45 | 64.22 | 7.22 | 0.019 | 0.450 | 0.53 |
| ERGIC1 | 134.88 | 26.99 | 253.54 | 72.63 | 4.34 | 0.049 | 0.452 | 0.53 |
| MVP | 566.73 | 142.04 | 1065.98 | 202.46 | 14.00 | 0.005 | 0.448 | 0.53 |
| GGA1 | 17862.75 | 7286.52 | 33598.87 | 6304.19 | 9.12 | 0.012 | 0.457 | 0.53 |
| EOMES | 179.85 | 45.04 | 338.43 | 91.71 | 5.77 | 0.029 | 0.444 | 0.53 |
| RNF13 | 309.26 | 52.80 | 581.97 | 109.86 | 8.01 | 0.015 | 0.452 | 0.53 |
| CSF3R | 123.18 | 9.16 | 232.25 | 52.04 | 4.33 | 0.049 | 0.452 | 0.53 |
| CAMSAP1L1 | 145.32 | 19.46 | 274.85 | 47.47 | 7.98 | 0.015 | 0.452 | 0.53 |
| FKBP1P1 | 133.59 | 19.08 | 252.74 | 11.16 | 11.73 | 0.007 | 0.449 | 0.53 |
| BTN3A3 | 211.58 | 16.96 | 400.93 | 23.67 | 45.62 | 0.000 | 0.630 | 0.53 |
| CHKB | 347.90 | 128.24 | 659.62 | 180.72 | 7.93 | 0.016 | 0.451 | 0.53 |
| ANKRD12 | 423.70 | 191.34 | 804.45 | 279.86 | 4.41 | 0.048 | 0.450 | 0.53 |
| LPP | 397.59 | 85.85 | 756.54 | 95.13 | 61.86 | 0.000 | 0.823 | 0.53 |
| LAMP2 | 311.11 | 106.51 | 592.25 | 122.75 | 20.49 | 0.002 | 0.443 | 0.53 |
| LRCH4 | 207.25 | 61.85 | 394.57 | 102.54 | 7.26 | 0.018 | 0.451 | 0.53 |
| TFIP11 | 164.71 | 17.53 | 313.72 | 17.08 | 27.38 | 0.001 | 0.446 | 0.53 |
| EDN1 | 124.50 | 37.40 | 237.21 | 69.05 | 6.09 | 0.026 | 0.450 | 0.52 |
| CYFIP1 | 156.88 | 13.44 | 299.24 | 56.31 | 5.70 | 0.029 | 0.445 | 0.52 |
| TM6SF1 | 125.51 | 7.58 | 239.65 | 41.75 | 4.86 | 0.040 | 0.444 | 0.52 |
| PINK1 | 151.27 | 31.18 | 289.20 | 67.29 | 6.54 | 0.023 | 0.454 | 0.52 |
| ZYX | 303.12 | 14.19 | 579.80 | 21.33 | 13.60 | 0.005 | 0.447 | 0.52 |
| EDEM2 | 372.71 | 90.30 | 714.12 | 213.15 | 4.53 | 0.045 | 0.449 | 0.52 |
| FCGR1C | 131.39 | 16.97 | 251.86 | 21.85 | 13.51 | 0.005 | 0.445 | 0.52 |
| FAM53B | 198.59 | 26.73 | 380.91 | 17.85 | 10.82 | 0.008 | 0.456 | 0.52 |
| HIST2H2BE | 131.90 | 6.39 | 253.01 | 32.20 | 7.28 | 0.018 | 0.449 | 0.52 |
| FLJ31306 | 295.36 | 128.88 | 566.91 | 150.82 | 18.20 | 0.003 | 0.453 | 0.52 |
| CLK1 | 662.20 | 328.29 | 1271.57 | 406.46 | 6.36 | 0.024 | 0.452 | 0.52 |
| SSPN | 108.75 | 4.97 | 208.91 | 39.33 | 4.92 | 0.039 | 0.445 | 0.52 |
| IRF9 | 1495.60 | 470.07 | 2891.16 | 834.50 | 6.57 | 0.022 | 0.454 | 0.52 |
| ARHGAP18 | 145.24 | 35.07 | 280.95 | 79.10 | 4.61 | 0.044 | 0.448 | 0.52 |
| DYRK2 | 313.94 | 108.58 | 608.01 | 202.14 | 4.71 | 0.042 | 0.446 | 0.52 |
| EIF4E3 | 294.32 | 134.38 | 570.57 | 139.03 | 7.63 | 0.017 | 0.446 | 0.52 |
| NUCB2 | 200.25 | 35.07 | 388.63 | 105.77 | 4.48 | 0.046 | 0.448 | 0.52 |
| LTB | 3236.82 | 1496.45 | 6288.64 | 2540.27 | 4.92 | 0.039 | 0.445 | 0.51 |
| LRRK2 | 203.33 | 50.74 | 395.05 | 105.75 | 4.43 | 0.047 | 0.450 | 0.51 |
| CNN3 | 151.78 | 43.89 | 295.55 | 93.00 | 5.06 | 0.037 | 0.446 | 0.51 |
| MYO9B | 315.75 | 68.80 | 615.22 | 179.21 | 4.44 | 0.047 | 0.450 | 0.51 |
| DUSP3 | 833.82 | 430.83 | 1627.30 | 466.82 | 5.17 | 0.035 | 0.447 | 0.51 |
| MLKL | 560.69 | 270.03 | 1095.62 | 304.06 | 13.80 | 0.005 | 0.450 | 0.51 |
| KIAA0430 | 598.36 | 300.09 | 1169.42 | 326.10 | 5.19 | 0.035 | 0.447 | 0.51 |
| C1ORF63 | 389.56 | 276.60 | 762.59 | 368.91 | 6.92 | 0.020 | 0.446 | 0.51 |
| WDR23 | 195.41 | 43.64 | 382.82 | 80.84 | 8.67 | 0.013 | 0.451 | 0.51 |
| HS.568928 | 147.21 | 22.48 | 288.71 | 77.31 | 4.39 | 0.048 | 0.451 | 0.51 |
| STXBP2 | 364.56 | 35.36 | 715.54 | 161.34 | 4.82 | 0.040 | 0.445 | 0.51 |
| MAN2B2 | 448.85 | 194.23 | 885.05 | 222.56 | 12.47 | 0.006 | 0.444 | 0.51 |
| LHPP | 177.89 | 24.75 | 351.22 | 79.12 | 4.54 | 0.045 | 0.449 | 0.51 |
| ROCK2 | 14879.99 | 7986.30 | 29380.71 | 6153.00 | 5.42 | 0.032 | 0.445 | 0.51 |
| STOM | 834.90 | 319.29 | 1652.45 | 517.13 | 5.24 | 0.034 | 0.447 | 0.51 |
| GPR84 | 133.27 | 14.76 | 263.81 | 40.44 | 6.53 | 0.023 | 0.453 | 0.51 |
| PRICKLE1 | 165.05 | 11.47 | 326.74 | 13.64 | 28.67 | 0.001 | 0.452 | 0.51 |
| CLCN7 | 1698.81 | 731.95 | 3363.84 | 1228.11 | 4.48 | 0.046 | 0.448 | 0.51 |
| ATP6V1G1 | 1660.16 | 802.95 | 3297.16 | 1164.27 | 6.20 | 0.025 | 0.449 | 0.50 |
| FGD2 | 654.41 | 378.51 | 1299.80 | 429.84 | 9.59 | 0.011 | 0.463 | 0.50 |
| PTP4A2 | 580.08 | 236.31 | 1153.96 | 205.52 | 14.00 | 0.005 | 0.450 | 0.50 |
| CCR6 | 15463.86 | 6692.82 | 30770.38 | 5987.38 | 6.88 | 0.020 | 0.445 | 0.50 |
| ATHL1 | 201.42 | 23.62 | 400.94 | 60.00 | 7.45 | 0.018 | 0.448 | 0.50 |
| N4BP2L1 | 183.46 | 42.27 | 366.10 | 79.44 | 7.62 | 0.017 | 0.445 | 0.50 |
| VPS13C | 256.41 | 64.45 | 512.36 | 157.36 | 4.65 | 0.043 | 0.447 | 0.50 |
| LOC100130886 | 267.19 | 60.02 | 534.46 | 138.87 | 5.87 | 0.028 | 0.446 | 0.50 |
| HS.576072 | 193.62 | 41.08 | 387.32 | 85.00 | 4.91 | 0.039 | 0.445 | 0.50 |
| FAM23B | 134.43 | 17.03 | 269.01 | 40.92 | 9.31 | 0.011 | 0.461 | 0.50 |
| LOC100131835 | 161.06 | 29.24 | 322.77 | 56.26 | 6.64 | 0.022 | 0.453 | 0.50 |
| MRC1L1 | 119.78 | 17.29 | 240.08 | 26.92 | 4.98 | 0.038 | 0.447 | 0.50 |
| FLVCR2 | 260.11 | 79.33 | 521.68 | 89.82 | 6.91 | 0.020 | 0.447 | 0.50 |
| ARRB1 | 145.45 | 6.67 | 292.52 | 32.08 | 8.02 | 0.015 | 0.452 | 0.50 |
| MX2 | 193.42 | 33.00 | 389.08 | 63.62 | 8.78 | 0.013 | 0.458 | 0.50 |
| ANXA2 | 3784.79 | 1743.42 | 7621.97 | 2654.68 | 6.89 | 0.020 | 0.446 | 0.50 |
| TLR6 | 145.39 | 10.66 | 292.95 | 15.55 | 26.52 | 0.001 | 0.453 | 0.50 |
| GBA | 339.49 | 67.62 | 684.63 | 127.56 | 8.37 | 0.014 | 0.452 | 0.50 |
| PPT1 | 2972.50 | 1597.07 | 5997.04 | 2136.72 | 8.72 | 0.013 | 0.455 | 0.50 |
| KIAA0513 | 121.18 | 2.76 | 245.42 | 33.21 | 6.64 | 0.022 | 0.452 | 0.49 |
| MAP3K1 | 890.49 | 341.07 | 1804.90 | 510.55 | 7.89 | 0.016 | 0.451 | 0.49 |
| CCDC88A | 128.49 | 14.26 | 260.56 | 41.79 | 7.35 | 0.018 | 0.450 | 0.49 |
| SIRPA | 1556.60 | 433.54 | 3157.43 | 997.81 | 4.90 | 0.039 | 0.444 | 0.49 |
| ITGB1 | 1738.20 | 1040.00 | 3539.02 | 1538.21 | 6.26 | 0.025 | 0.449 | 0.49 |
| CYBA | 877.94 | 139.54 | 1787.58 | 249.10 | 8.17 | 0.015 | 0.451 | 0.49 |
| ZBTB4 | 504.28 | 218.49 | 1026.83 | 306.97 | 10.11 | 0.010 | 0.454 | 0.49 |
| RAPGEF2 | 328.26 | 165.87 | 670.56 | 253.69 | 6.12 | 0.026 | 0.450 | 0.49 |
| GOLGA9P | 126.43 | 11.42 | 258.35 | 59.48 | 4.58 | 0.045 | 0.448 | 0.49 |
| SCPEP1 | 993.95 | 367.28 | 2032.49 | 753.72 | 4.52 | 0.046 | 0.448 | 0.49 |
| LOC729324 | 13996.36 | 4322.03 | 28641.08 | 5557.52 | 6.57 | 0.022 | 0.454 | 0.49 |
| PECAM1 | 179.04 | 11.84 | 367.06 | 55.99 | 4.86 | 0.040 | 0.445 | 0.49 |
| C2 | 115.83 | 8.42 | 237.50 | 22.33 | 7.01 | 0.020 | 0.449 | 0.49 |
| CDC42SE2 | 1075.29 | 572.35 | 2206.19 | 791.53 | 7.68 | 0.017 | 0.448 | 0.49 |
| LOC441268 | 141.90 | 11.01 | 291.24 | 41.59 | 8.43 | 0.014 | 0.451 | 0.49 |
| FLJ32255 | 126.98 | 13.29 | 261.03 | 64.83 | 4.42 | 0.047 | 0.449 | 0.49 |
| LOC440157 | 161.04 | 41.69 | 331.82 | 65.64 | 5.42 | 0.032 | 0.445 | 0.49 |
| NBPF10 | 2199.42 | 948.20 | 4541.57 | 1716.89 | 4.58 | 0.044 | 0.448 | 0.48 |
| LOC100131786 | 130.28 | 17.28 | 270.36 | 63.39 | 5.22 | 0.035 | 0.447 | 0.48 |
| LOC100133298 | 186.60 | 32.90 | 387.72 | 102.27 | 4.30 | 0.050 | 0.452 | 0.48 |
| EMILIN2 | 750.52 | 223.74 | 1561.87 | 490.73 | 5.26 | 0.034 | 0.448 | 0.48 |
| ZNF773 | 209.91 | 52.57 | 436.85 | 115.07 | 5.63 | 0.030 | 0.446 | 0.48 |
| FAM153B | 237.70 | 69.39 | 495.33 | 125.94 | 7.04 | 0.020 | 0.449 | 0.48 |
| RPS6KA2 | 120.92 | 11.68 | 252.50 | 63.51 | 4.31 | 0.050 | 0.452 | 0.48 |
| CTSO | 176.10 | 28.11 | 368.41 | 76.39 | 6.12 | 0.026 | 0.450 | 0.48 |
| RRAS | 199.16 | 36.84 | 416.90 | 98.70 | 5.56 | 0.031 | 0.445 | 0.48 |
| SAMD9 | 358.46 | 131.07 | 751.78 | 228.28 | 7.01 | 0.020 | 0.448 | 0.48 |
| ALOX5 | 697.84 | 457.47 | 1465.68 | 341.58 | 10.06 | 0.010 | 0.454 | 0.48 |
| SHCBP1 | 7214.42 | 3140.36 | 15167.32 | 4738.80 | 8.58 | 0.013 | 0.451 | 0.48 |
| CD74 | 5643.69 | 1251.25 | 11869.74 | 1296.95 | 4.79 | 0.041 | 0.444 | 0.48 |
| MYO1F | 153.30 | 14.19 | 322.78 | 42.54 | 9.45 | 0.011 | 0.465 | 0.47 |
| AKAP13 | 579.52 | 195.08 | 1228.38 | 431.84 | 4.56 | 0.045 | 0.448 | 0.47 |
| SH3BGRL | 1038.33 | 380.57 | 2205.11 | 764.40 | 5.16 | 0.036 | 0.447 | 0.47 |
| PGD | 1571.97 | 290.90 | 3344.40 | 618.88 | 8.55 | 0.013 | 0.451 | 0.47 |
| PCMTD1 | 317.06 | 115.93 | 677.77 | 189.12 | 8.22 | 0.014 | 0.452 | 0.47 |
| APOC1 | 198.55 | 27.59 | 424.63 | 27.76 | 10.39 | 0.009 | 0.454 | 0.47 |
| LOC646996 | 1117.54 | 480.00 | 2392.19 | 541.68 | 6.95 | 0.020 | 0.447 | 0.47 |
| LOC727808 | 13746.52 | 6226.89 | 29437.14 | 5975.01 | 9.55 | 0.011 | 0.462 | 0.47 |
| DKFZP434K191 | 258.04 | 61.85 | 552.89 | 151.76 | 4.58 | 0.045 | 0.448 | 0.47 |
| SIDT2 | 697.01 | 318.20 | 1493.58 | 324.51 | 8.62 | 0.013 | 0.450 | 0.47 |
| C9ORF130 | 383.21 | 123.88 | 822.92 | 208.28 | 8.89 | 0.012 | 0.456 | 0.47 |
| MCOLN1 | 188.16 | 19.21 | 404.82 | 59.28 | 8.18 | 0.015 | 0.452 | 0.46 |
| LST1 | 147.34 | 9.39 | 317.07 | 46.24 | 7.73 | 0.016 | 0.449 | 0.46 |
| IRF7 | 596.58 | 149.50 | 1290.38 | 279.42 | 7.49 | 0.017 | 0.448 | 0.46 |
| ZEB2 | 265.62 | 53.98 | 574.63 | 122.93 | 7.11 | 0.019 | 0.450 | 0.46 |
| LST1 | 167.38 | 3.71 | 362.11 | 71.63 | 4.61 | 0.044 | 0.448 | 0.46 |
| STAT1 | 6403.51 | 2327.99 | 13892.69 | 4370.42 | 6.24 | 0.025 | 0.449 | 0.46 |
| RBM3 | 164.39 | 26.15 | 356.69 | 27.09 | 34.61 | 0.001 | 0.512 | 0.46 |
| PTPRO | 149.67 | 28.53 | 324.83 | 74.79 | 5.90 | 0.028 | 0.447 | 0.46 |
| XRN1 | 541.69 | 250.98 | 1178.13 | 369.36 | 9.26 | 0.011 | 0.458 | 0.46 |
| RNASET2 | 1683.19 | 557.47 | 3665.47 | 634.88 | 11.66 | 0.007 | 0.449 | 0.46 |
| RAB31 | 939.44 | 523.38 | 2048.22 | 617.66 | 19.11 | 0.003 | 0.451 | 0.46 |
| PTPN12 | 692.38 | 316.49 | 1510.20 | 579.78 | 5.07 | 0.037 | 0.446 | 0.46 |
| CD96 | 201.25 | 59.43 | 439.13 | 125.16 | 5.99 | 0.027 | 0.447 | 0.46 |
| IRF7 | 232.30 | 8.73 | 507.09 | 41.93 | 11.02 | 0.008 | 0.457 | 0.46 |
| DAPK1 | 144.02 | 7.94 | 315.27 | 46.20 | 7.13 | 0.019 | 0.449 | 0.46 |
| ALDH3B1 | 120.32 | 10.17 | 263.46 | 60.05 | 4.74 | 0.042 | 0.445 | 0.46 |
| PPAP2B | 127.09 | 22.88 | 278.74 | 79.06 | 4.40 | 0.048 | 0.450 | 0.46 |
| TLR8 | 125.34 | 9.74 | 275.34 | 44.39 | 7.47 | 0.017 | 0.448 | 0.46 |
| COX19 | 240.71 | 80.14 | 529.69 | 108.61 | 6.78 | 0.021 | 0.449 | 0.45 |
| HS.99472 | 121.61 | 8.09 | 267.66 | 50.10 | 4.76 | 0.041 | 0.444 | 0.45 |
| ALDH2 | 140.15 | 12.47 | 308.53 | 24.19 | 17.93 | 0.003 | 0.446 | 0.45 |
| LOC283663 | 159.12 | 27.59 | 350.30 | 3.70 | 13.56 | 0.005 | 0.446 | 0.45 |
| PYCARD | 386.46 | 101.89 | 851.37 | 171.65 | 10.06 | 0.010 | 0.453 | 0.45 |
| LOC643977 | 139.98 | 11.02 | 308.38 | 50.54 | 6.59 | 0.022 | 0.453 | 0.45 |
| PVRL2 | 131.00 | 20.25 | 289.00 | 21.08 | 6.67 | 0.022 | 0.452 | 0.45 |
| CYFIP1 | 416.74 | 130.61 | 919.68 | 261.15 | 6.49 | 0.023 | 0.452 | 0.45 |
| UNC84B | 2307.35 | 1327.79 | 5092.04 | 1777.82 | 8.44 | 0.014 | 0.452 | 0.45 |
| LOC283953 | 201.46 | 41.08 | 445.73 | 70.37 | 5.75 | 0.029 | 0.445 | 0.45 |
| MGC16703 | 14702.41 | 7256.22 | 32559.82 | 5482.16 | 8.25 | 0.014 | 0.452 | 0.45 |
| DIRC2 | 164.23 | 26.24 | 364.87 | 82.28 | 4.89 | 0.039 | 0.444 | 0.45 |
| PRRG4 | 211.49 | 50.81 | 471.24 | 92.85 | 4.49 | 0.046 | 0.448 | 0.45 |
| EVL | 2107.80 | 777.48 | 4699.14 | 951.31 | 18.63 | 0.003 | 0.449 | 0.45 |
| SPI1 | 568.77 | 98.49 | 1271.15 | 123.07 | 30.57 | 0.001 | 0.447 | 0.45 |
| LILRB4 | 364.59 | 145.87 | 815.82 | 292.93 | 5.29 | 0.034 | 0.447 | 0.45 |
| S100A10 | 2549.32 | 973.19 | 5708.39 | 1362.24 | 13.46 | 0.005 | 0.444 | 0.45 |
| ISG20 | 1986.87 | 698.73 | 4451.68 | 1413.80 | 5.24 | 0.035 | 0.447 | 0.45 |
| MITF | 134.90 | 11.19 | 302.28 | 70.23 | 4.83 | 0.040 | 0.444 | 0.45 |
| KIAA0101 | 13483.47 | 5954.61 | 30279.84 | 4278.13 | 13.53 | 0.005 | 0.446 | 0.45 |
| ANXA5 | 1446.28 | 398.95 | 3253.21 | 373.16 | 117.89 | 0.000 | 0.850 | 0.44 |
| BOK | 143.03 | 20.70 | 321.86 | 88.34 | 4.52 | 0.046 | 0.449 | 0.44 |
| HBP1 | 186.43 | 8.50 | 420.70 | 94.29 | 4.61 | 0.044 | 0.447 | 0.44 |
| GCLC | 292.59 | 115.09 | 660.68 | 222.05 | 5.46 | 0.032 | 0.445 | 0.44 |
| LOC400721 | 8186.64 | 3035.30 | 18489.40 | 4472.64 | 5.94 | 0.027 | 0.447 | 0.44 |
| LOC100133649 | 10501.02 | 5127.03 | 23773.74 | 5281.78 | 16.92 | 0.003 | 0.453 | 0.44 |
| SGK3 | 134.68 | 18.60 | 305.21 | 63.95 | 5.04 | 0.037 | 0.447 | 0.44 |
| VAV3 | 182.20 | 24.00 | 413.89 | 86.92 | 4.70 | 0.042 | 0.447 | 0.44 |
| SMCR5 | 190.21 | 42.95 | 432.53 | 53.26 | 13.44 | 0.005 | 0.442 | 0.44 |
| C12ORF35 | 820.08 | 380.82 | 1864.94 | 722.44 | 5.28 | 0.034 | 0.447 | 0.44 |
| ARHGEF3 | 874.29 | 471.74 | 1991.36 | 573.43 | 15.88 | 0.004 | 0.453 | 0.44 |
| ANKRD44 | 147.92 | 33.61 | 337.21 | 82.13 | 5.95 | 0.027 | 0.447 | 0.44 |
| SIGLEC9 | 124.33 | 8.58 | 283.82 | 40.05 | 7.29 | 0.018 | 0.450 | 0.44 |
| ICA1 | 728.36 | 331.51 | 1663.42 | 481.00 | 5.92 | 0.027 | 0.447 | 0.44 |
| GBP2 | 3682.43 | 1418.70 | 8420.39 | 1671.16 | 29.83 | 0.001 | 0.449 | 0.44 |
| NCF1 | 266.24 | 85.97 | 609.25 | 211.78 | 4.70 | 0.042 | 0.446 | 0.44 |
| LOC644745 | 12342.89 | 7164.09 | 28268.05 | 6174.89 | 8.72 | 0.013 | 0.455 | 0.44 |
| CNTNAP2 | 92.06 | 3.48 | 211.32 | 25.38 | 7.64 | 0.017 | 0.446 | 0.44 |
| LOC100133866 | 192.93 | 40.60 | 442.96 | 103.50 | 5.49 | 0.032 | 0.446 | 0.44 |
| CD300LF | 128.72 | 22.81 | 295.60 | 81.11 | 4.95 | 0.038 | 0.445 | 0.44 |
| RBMS1 | 293.09 | 43.21 | 673.63 | 69.87 | 21.53 | 0.002 | 0.452 | 0.44 |
| HBP1 | 185.49 | 34.72 | 426.82 | 107.18 | 5.37 | 0.033 | 0.445 | 0.43 |
| LOC338758 | 232.36 | 54.46 | 534.67 | 35.18 | 9.75 | 0.010 | 0.458 | 0.43 |
| BTN3A2 | 431.90 | 167.17 | 994.71 | 195.92 | 7.18 | 0.019 | 0.450 | 0.43 |
| PRMT2 | 166.95 | 25.12 | 384.59 | 94.59 | 5.03 | 0.037 | 0.446 | 0.43 |
| RPL7L1 | 5542.08 | 2715.83 | 12773.34 | 4147.94 | 5.82 | 0.028 | 0.444 | 0.43 |
| H2AFY | 153.18 | 32.15 | 353.18 | 71.31 | 5.08 | 0.037 | 0.446 | 0.43 |
| IL1R2 | 196.29 | 45.47 | 453.98 | 96.53 | 4.49 | 0.046 | 0.448 | 0.43 |
| P2RX7 | 183.95 | 50.84 | 426.11 | 61.90 | 9.50 | 0.011 | 0.464 | 0.43 |
| P2RX4 | 184.09 | 61.14 | 427.86 | 133.07 | 5.83 | 0.028 | 0.445 | 0.43 |
| CD37 | 421.25 | 106.28 | 980.24 | 190.85 | 6.30 | 0.024 | 0.449 | 0.43 |
| KIAA0247 | 444.24 | 147.83 | 1036.07 | 332.97 | 5.48 | 0.032 | 0.445 | 0.43 |
| NPC1 | 593.89 | 234.44 | 1385.67 | 399.30 | 7.72 | 0.016 | 0.448 | 0.43 |
| TIMP2 | 111.46 | 5.61 | 260.32 | 54.18 | 4.76 | 0.041 | 0.444 | 0.43 |
| ADAM17 | 256.84 | 77.45 | 599.91 | 87.45 | 7.51 | 0.017 | 0.448 | 0.43 |
| LOC100131096 | 356.06 | 127.89 | 833.15 | 252.64 | 6.19 | 0.025 | 0.449 | 0.43 |
| HLA-C | 330.18 | 79.34 | 772.78 | 179.38 | 4.33 | 0.050 | 0.452 | 0.43 |
| LOC643509 | 9212.27 | 5154.75 | 21686.34 | 4092.08 | 6.46 | 0.023 | 0.453 | 0.42 |
| C11ORF75 | 471.57 | 206.74 | 1112.33 | 335.37 | 6.89 | 0.020 | 0.446 | 0.42 |
| CDAN1 | 5545.56 | 2083.72 | 13083.08 | 3611.88 | 7.85 | 0.016 | 0.451 | 0.42 |
| HLA-DRA | 7878.36 | 2133.66 | 18620.26 | 5764.21 | 4.55 | 0.045 | 0.448 | 0.42 |
| CASP4 | 914.22 | 427.48 | 2160.97 | 636.86 | 9.30 | 0.011 | 0.461 | 0.42 |
| CEP27 | 654.14 | 307.95 | 1547.90 | 421.43 | 7.07 | 0.019 | 0.449 | 0.42 |
| ADAMDEC1 | 134.45 | 18.38 | 318.36 | 68.09 | 6.39 | 0.024 | 0.453 | 0.42 |
| LOC90925 | 172.50 | 28.35 | 409.06 | 96.62 | 5.61 | 0.030 | 0.445 | 0.42 |
| LOC643882 | 166.19 | 42.71 | 394.91 | 81.17 | 6.73 | 0.021 | 0.450 | 0.42 |
| CD24 | 463.80 | 134.68 | 1103.50 | 238.72 | 9.30 | 0.011 | 0.461 | 0.42 |
| LOC401152 | 256.84 | 69.88 | 613.60 | 194.81 | 4.94 | 0.039 | 0.446 | 0.42 |
| IFI44 | 333.11 | 112.72 | 796.37 | 203.51 | 7.17 | 0.019 | 0.450 | 0.42 |
| PQLC3 | 401.31 | 157.60 | 959.93 | 353.95 | 4.88 | 0.039 | 0.444 | 0.42 |
| LOC100129960 | 144.99 | 26.67 | 347.18 | 85.44 | 5.91 | 0.027 | 0.448 | 0.42 |
| STAT1 | 1963.46 | 753.45 | 4712.58 | 1610.98 | 4.84 | 0.040 | 0.444 | 0.42 |
| FAM65B | 281.09 | 86.97 | 675.01 | 124.96 | 7.08 | 0.019 | 0.450 | 0.42 |
| GLRX | 2040.27 | 1001.05 | 4904.64 | 1148.14 | 30.30 | 0.001 | 0.447 | 0.42 |
| RN7SL1 | 13271.40 | 5411.38 | 31910.64 | 6731.87 | 22.09 | 0.002 | 0.451 | 0.42 |
| DAPP1 | 4189.64 | 1819.71 | 10079.75 | 2891.40 | 5.91 | 0.027 | 0.447 | 0.42 |
| PLEKHA1 | 202.95 | 46.29 | 488.35 | 143.13 | 4.91 | 0.039 | 0.445 | 0.42 |
| C16ORF7 | 165.19 | 36.41 | 397.82 | 95.30 | 6.11 | 0.026 | 0.450 | 0.42 |
| PSMD12 | 10583.71 | 4816.43 | 25489.56 | 5759.21 | 6.10 | 0.026 | 0.450 | 0.42 |
| HIST2H2AA3 | 167.00 | 23.50 | 402.80 | 99.09 | 4.41 | 0.048 | 0.450 | 0.41 |
| CAMK1 | 205.15 | 3.15 | 495.11 | 43.15 | 11.01 | 0.008 | 0.456 | 0.41 |
| CAPG | 210.04 | 34.72 | 508.44 | 103.65 | 5.24 | 0.034 | 0.447 | 0.41 |
| FOXO4 | 248.10 | 33.09 | 600.76 | 98.18 | 8.52 | 0.013 | 0.451 | 0.41 |
| PILRA | 139.11 | 23.27 | 336.90 | 74.44 | 6.56 | 0.022 | 0.454 | 0.41 |
| LOC644642 | 287.16 | 78.60 | 696.30 | 207.89 | 5.25 | 0.034 | 0.447 | 0.41 |
| ATP6V1B2 | 2830.69 | 1269.15 | 6868.44 | 2030.11 | 8.40 | 0.014 | 0.452 | 0.41 |
| SORT1 | 134.58 | 12.55 | 327.07 | 56.96 | 6.34 | 0.024 | 0.451 | 0.41 |
| PDCD7 | 12118.18 | 3999.15 | 29458.17 | 4220.47 | 35.33 | 0.001 | 0.525 | 0.41 |
| ARRDC4 | 159.14 | 6.18 | 386.89 | 55.54 | 7.60 | 0.017 | 0.445 | 0.41 |
| LOC123688 | 179.08 | 33.87 | 435.42 | 83.88 | 8.71 | 0.013 | 0.454 | 0.41 |
| LAMP1 | 1950.08 | 856.07 | 4741.85 | 1511.98 | 6.67 | 0.022 | 0.452 | 0.41 |
| LOC100133607 | 10968.68 | 5407.64 | 26673.32 | 4184.94 | 14.77 | 0.005 | 0.453 | 0.41 |
| LILRB3 | 9403.72 | 3491.73 | 22880.36 | 6356.67 | 7.59 | 0.017 | 0.444 | 0.41 |
| HS.197143 | 109.39 | 3.54 | 266.57 | 35.85 | 7.82 | 0.016 | 0.449 | 0.41 |
| LOC100132564 | 1667.26 | 1321.35 | 4071.69 | 1873.35 | 5.02 | 0.037 | 0.447 | 0.41 |
| ZNF669 | 266.64 | 116.18 | 651.80 | 155.63 | 5.72 | 0.029 | 0.445 | 0.41 |
| LOC440704 | 876.69 | 367.09 | 2144.33 | 735.18 | 5.29 | 0.034 | 0.447 | 0.41 |
| ANKRD30B | 2194.47 | 1032.37 | 5396.13 | 1402.25 | 10.30 | 0.009 | 0.453 | 0.41 |
| XPNPEP3 | 10828.02 | 6122.75 | 26640.68 | 4870.97 | 10.99 | 0.008 | 0.456 | 0.41 |
| LOC389517 | 5759.14 | 2421.05 | 14176.67 | 3396.57 | 7.16 | 0.019 | 0.451 | 0.41 |
| LOC400446 | 830.09 | 277.85 | 2047.61 | 571.25 | 7.01 | 0.020 | 0.450 | 0.41 |
| S100A6 | 1635.79 | 705.32 | 4038.59 | 1438.03 | 5.31 | 0.034 | 0.447 | 0.41 |
| MXD4 | 201.35 | 56.26 | 497.45 | 157.58 | 5.05 | 0.037 | 0.447 | 0.40 |
| LOC731486 | 173.38 | 19.51 | 428.39 | 36.05 | 19.64 | 0.003 | 0.457 | 0.40 |
| SPI1 | 538.44 | 206.74 | 1333.35 | 281.07 | 6.37 | 0.024 | 0.452 | 0.40 |
| CD44 | 349.08 | 111.44 | 864.79 | 103.49 | 10.89 | 0.008 | 0.456 | 0.40 |
| SHISA5 | 466.82 | 92.75 | 1156.69 | 194.70 | 6.43 | 0.023 | 0.453 | 0.40 |
| KYNU | 1613.39 | 533.95 | 3999.33 | 673.65 | 24.14 | 0.002 | 0.435 | 0.40 |
| IL17RA | 187.86 | 28.08 | 466.10 | 97.33 | 5.14 | 0.036 | 0.446 | 0.40 |
| C21ORF55 | 6379.85 | 3051.15 | 15866.09 | 3954.54 | 12.65 | 0.006 | 0.439 | 0.40 |
| GRN | 300.22 | 30.23 | 747.02 | 152.36 | 5.01 | 0.038 | 0.446 | 0.40 |
| HLA-DRB4 | 435.87 | 201.92 | 1085.65 | 333.96 | 4.63 | 0.044 | 0.447 | 0.40 |
| HEXB | 1488.54 | 786.18 | 3708.37 | 937.72 | 25.29 | 0.002 | 0.436 | 0.40 |
| S100A10 | 1726.73 | 830.59 | 4314.12 | 1711.85 | 4.55 | 0.045 | 0.448 | 0.40 |
| AIRE | 3955.67 | 1147.00 | 9895.04 | 2848.35 | 5.72 | 0.029 | 0.445 | 0.40 |
| ATP6AP2 | 2204.09 | 744.04 | 5515.16 | 1205.94 | 9.87 | 0.010 | 0.459 | 0.40 |
| VEZF1 | 316.39 | 110.37 | 795.45 | 253.36 | 4.60 | 0.044 | 0.448 | 0.40 |
| ITGB2 | 5615.99 | 1639.04 | 14185.52 | 3404.97 | 7.50 | 0.017 | 0.448 | 0.40 |
| FTL | 12876.51 | 2628.23 | 32569.45 | 9242.11 | 4.99 | 0.038 | 0.447 | 0.40 |
| LILRB1 | 4147.22 | 2173.58 | 10492.80 | 3205.03 | 9.27 | 0.011 | 0.459 | 0.40 |
| OKL38 | 176.05 | 56.44 | 446.51 | 139.57 | 4.32 | 0.050 | 0.452 | 0.39 |
| SLC44A4 | 5658.11 | 2803.58 | 14376.77 | 3976.84 | 9.00 | 0.012 | 0.455 | 0.39 |
| TSPAN32 | 143.17 | 32.71 | 363.91 | 78.94 | 8.14 | 0.015 | 0.452 | 0.39 |
| PTGER2 | 183.62 | 45.49 | 467.47 | 147.72 | 4.80 | 0.041 | 0.444 | 0.39 |
| CXCR4 | 546.72 | 247.80 | 1395.46 | 427.19 | 5.86 | 0.028 | 0.445 | 0.39 |
| FKTN | 5496.06 | 2950.79 | 14032.98 | 4453.68 | 7.74 | 0.016 | 0.450 | 0.39 |
| PCDHB9 | 232.07 | 55.50 | 594.90 | 155.06 | 4.74 | 0.042 | 0.445 | 0.39 |
| ITGB2 | 5645.15 | 1728.99 | 14474.20 | 3846.97 | 6.60 | 0.022 | 0.453 | 0.39 |
| PILRA | 136.88 | 3.70 | 351.06 | 19.75 | 18.66 | 0.003 | 0.452 | 0.39 |
| XAF1 | 197.81 | 34.63 | 508.01 | 61.32 | 12.76 | 0.006 | 0.439 | 0.39 |
| GRN | 328.85 | 134.24 | 845.34 | 289.97 | 5.74 | 0.029 | 0.445 | 0.39 |
| TP53INP1 | 150.59 | 28.95 | 388.01 | 109.06 | 5.13 | 0.036 | 0.446 | 0.39 |
| EPSTI1 | 1067.15 | 620.46 | 2749.65 | 874.01 | 11.43 | 0.008 | 0.453 | 0.39 |
| PARVG | 472.39 | 55.20 | 1219.55 | 234.37 | 7.07 | 0.019 | 0.449 | 0.39 |
| LY96 | 559.27 | 193.06 | 1445.13 | 362.04 | 8.86 | 0.012 | 0.457 | 0.39 |
| TBC1D9 | 243.81 | 97.92 | 630.11 | 163.93 | 6.35 | 0.024 | 0.452 | 0.39 |
| C5ORF41 | 182.23 | 31.96 | 472.09 | 140.95 | 4.59 | 0.044 | 0.447 | 0.39 |
| C4ORF34 | 639.20 | 209.91 | 1657.27 | 219.42 | 8.98 | 0.012 | 0.454 | 0.39 |
| CSF2RA | 116.78 | 9.16 | 302.90 | 75.15 | 4.61 | 0.044 | 0.448 | 0.39 |
| LOC100132727 | 1852.15 | 578.49 | 4805.16 | 1195.23 | 8.17 | 0.015 | 0.452 | 0.39 |
| LOC653489 | 1195.04 | 424.43 | 3102.40 | 901.08 | 6.64 | 0.022 | 0.452 | 0.39 |
| PLXNB2 | 202.73 | 54.93 | 526.63 | 126.27 | 7.60 | 0.017 | 0.446 | 0.38 |
| LOC645895 | 11296.92 | 5845.73 | 29372.07 | 6254.24 | 7.59 | 0.017 | 0.444 | 0.38 |
| FCGR1B | 128.35 | 8.02 | 334.07 | 36.39 | 8.13 | 0.015 | 0.451 | 0.38 |
| CCRL2 | 212.22 | 35.71 | 552.65 | 120.54 | 5.67 | 0.030 | 0.446 | 0.38 |
| PDE4C | 6805.67 | 3451.58 | 17723.89 | 4632.51 | 6.99 | 0.020 | 0.448 | 0.38 |
| FNDC3B | 219.80 | 63.81 | 573.54 | 128.60 | 9.45 | 0.011 | 0.465 | 0.38 |
| PARP12 | 391.24 | 104.06 | 1022.01 | 182.38 | 12.81 | 0.006 | 0.438 | 0.38 |
| TP53INP1 | 188.52 | 44.34 | 492.83 | 115.06 | 7.24 | 0.019 | 0.450 | 0.38 |
| LOC100128460 | 187.52 | 55.67 | 490.25 | 172.63 | 4.33 | 0.049 | 0.452 | 0.38 |
| LOC100133233 | 7311.76 | 4021.51 | 19132.44 | 5725.92 | 6.77 | 0.021 | 0.448 | 0.38 |
| CSF2RA | 1495.64 | 844.95 | 3917.74 | 1057.09 | 7.56 | 0.017 | 0.446 | 0.38 |
| GABARAPL1 | 180.78 | 28.85 | 473.71 | 92.42 | 6.03 | 0.026 | 0.449 | 0.38 |
| ARRDC4 | 126.99 | 27.53 | 333.05 | 88.28 | 5.07 | 0.037 | 0.446 | 0.38 |
| KYNU | 454.43 | 102.12 | 1192.76 | 231.33 | 7.39 | 0.018 | 0.448 | 0.38 |
| FAM115A | 8389.43 | 4432.75 | 22059.18 | 4375.91 | 36.75 | 0.001 | 0.522 | 0.38 |
| CD44 | 3625.03 | 1801.13 | 9532.43 | 2882.23 | 6.15 | 0.025 | 0.449 | 0.38 |
| GBP4 | 1467.69 | 713.64 | 3863.76 | 890.07 | 18.76 | 0.003 | 0.457 | 0.38 |
| LOC653086 | 2146.62 | 968.11 | 5655.05 | 1466.82 | 8.33 | 0.014 | 0.452 | 0.38 |
| ZNF223 | 688.17 | 276.79 | 1814.28 | 524.50 | 6.75 | 0.021 | 0.449 | 0.38 |
| ENG | 199.79 | 16.90 | 526.81 | 73.08 | 9.81 | 0.010 | 0.457 | 0.38 |
| SLC24A6 | 259.27 | 57.73 | 683.70 | 227.78 | 4.32 | 0.050 | 0.452 | 0.38 |
| LOC731789 | 188.48 | 54.90 | 498.84 | 159.27 | 4.49 | 0.046 | 0.449 | 0.38 |
| HS.560357 | 159.40 | 45.95 | 422.57 | 66.55 | 4.80 | 0.041 | 0.444 | 0.38 |
| LAMP2 | 425.44 | 111.76 | 1128.25 | 295.77 | 6.62 | 0.022 | 0.454 | 0.38 |
| BTN3A1 | 186.28 | 47.17 | 495.53 | 163.06 | 4.40 | 0.048 | 0.450 | 0.38 |
| LOC647389 | 272.84 | 96.75 | 726.42 | 123.36 | 6.39 | 0.024 | 0.453 | 0.38 |
| STAT1 | 3686.47 | 1398.85 | 9843.94 | 3238.29 | 5.65 | 0.030 | 0.447 | 0.37 |
| YPEL3 | 207.68 | 39.82 | 555.24 | 94.98 | 8.75 | 0.013 | 0.456 | 0.37 |
| C10ORF58 | 3792.18 | 1617.34 | 10142.74 | 3026.13 | 7.81 | 0.016 | 0.448 | 0.37 |
| AKR1D1 | 6258.26 | 2749.81 | 16857.39 | 4364.84 | 10.10 | 0.010 | 0.454 | 0.37 |
| PNPT1 | 4988.10 | 2040.78 | 13468.69 | 3927.16 | 5.59 | 0.031 | 0.445 | 0.37 |
| MS4A7 | 119.80 | 18.48 | 323.77 | 61.42 | 7.59 | 0.017 | 0.444 | 0.37 |
| PHAX | 194.97 | 66.17 | 527.80 | 115.24 | 6.80 | 0.021 | 0.449 | 0.37 |
| SYNE2 | 160.83 | 29.53 | 435.53 | 109.98 | 5.80 | 0.028 | 0.444 | 0.37 |
| LOC100134159 | 2059.66 | 1052.57 | 5592.31 | 1401.07 | 15.41 | 0.004 | 0.451 | 0.37 |
| CFB | 128.97 | 14.13 | 350.56 | 44.40 | 12.43 | 0.006 | 0.445 | 0.37 |
| VAT1 | 248.87 | 45.40 | 677.26 | 167.94 | 5.21 | 0.035 | 0.447 | 0.37 |
| IFI44L | 286.02 | 65.16 | 778.53 | 203.82 | 5.89 | 0.028 | 0.446 | 0.37 |
| HLA-DMB | 3361.99 | 1230.53 | 9151.71 | 3119.38 | 5.29 | 0.034 | 0.447 | 0.37 |
| ZNF557 | 260.16 | 69.06 | 709.31 | 202.50 | 4.91 | 0.039 | 0.445 | 0.37 |
| RASSF6 | 188.83 | 58.76 | 514.99 | 152.22 | 4.74 | 0.042 | 0.445 | 0.37 |
| IFI30 | 799.75 | 150.49 | 2181.45 | 533.14 | 6.24 | 0.025 | 0.449 | 0.37 |
| LOC390530 | 238.67 | 53.01 | 651.82 | 144.56 | 5.95 | 0.027 | 0.447 | 0.37 |
| GLIPR2 | 1046.50 | 484.46 | 2859.89 | 433.31 | 36.03 | 0.001 | 0.520 | 0.37 |
| VPS41 | 430.43 | 138.16 | 1178.12 | 285.69 | 8.68 | 0.013 | 0.452 | 0.37 |
| LOC100128126 | 200.93 | 52.85 | 550.68 | 130.28 | 5.26 | 0.034 | 0.448 | 0.36 |
| APOL3 | 539.37 | 175.42 | 1478.41 | 377.66 | 5.43 | 0.032 | 0.445 | 0.36 |
| TPP1 | 764.11 | 285.54 | 2097.82 | 369.62 | 27.43 | 0.001 | 0.451 | 0.36 |
| GABPB2 | 8716.89 | 4900.58 | 23939.48 | 7440.49 | 9.25 | 0.011 | 0.457 | 0.36 |
| PPM1K | 252.15 | 48.04 | 692.79 | 134.60 | 8.72 | 0.013 | 0.454 | 0.36 |
| CYTH4 | 238.17 | 48.24 | 657.90 | 125.62 | 9.39 | 0.011 | 0.463 | 0.36 |
| PDCD4 | 1009.11 | 514.42 | 2791.96 | 807.18 | 5.59 | 0.031 | 0.445 | 0.36 |
| KCNH6 | 5389.60 | 3019.59 | 14939.52 | 5343.40 | 5.62 | 0.030 | 0.445 | 0.36 |
| SPOCK2 | 620.03 | 328.69 | 1722.06 | 714.87 | 4.43 | 0.047 | 0.450 | 0.36 |
| PSCD4 | 310.12 | 57.63 | 861.91 | 185.54 | 7.36 | 0.018 | 0.449 | 0.36 |
| TMEM158 | 223.49 | 106.57 | 621.89 | 250.03 | 4.68 | 0.043 | 0.447 | 0.36 |
| LBA1 | 188.04 | 22.48 | 523.51 | 135.81 | 5.12 | 0.036 | 0.447 | 0.36 |
| LOC730060 | 258.14 | 108.45 | 721.70 | 243.35 | 4.60 | 0.044 | 0.448 | 0.36 |
| KLHL28 | 304.02 | 127.25 | 851.67 | 204.43 | 6.40 | 0.024 | 0.453 | 0.36 |
| ECGF1 | 4652.98 | 2004.99 | 13069.92 | 3606.78 | 9.10 | 0.012 | 0.457 | 0.36 |
| FTHL16 | 11250.75 | 3524.88 | 31610.25 | 5343.54 | 19.36 | 0.003 | 0.457 | 0.36 |
| STAT2 | 1283.20 | 415.07 | 3606.67 | 929.95 | 7.79 | 0.016 | 0.449 | 0.36 |
| LOC100133177 | 6036.66 | 3014.55 | 16993.50 | 4556.83 | 12.01 | 0.007 | 0.446 | 0.36 |
| CD9 | 196.52 | 31.61 | 553.96 | 127.42 | 6.46 | 0.023 | 0.453 | 0.35 |
| PLA2G2D | 218.24 | 48.93 | 615.71 | 175.88 | 5.31 | 0.034 | 0.447 | 0.35 |
| LOC100134053 | 236.83 | 59.16 | 668.43 | 154.85 | 5.52 | 0.031 | 0.446 | 0.35 |
| ARHGAP10 | 145.86 | 24.72 | 413.01 | 84.71 | 7.55 | 0.017 | 0.446 | 0.35 |
| SRXN1 | 733.74 | 246.65 | 2080.18 | 487.74 | 9.19 | 0.012 | 0.456 | 0.35 |
| MFSD11 | 300.34 | 118.84 | 854.56 | 279.87 | 4.35 | 0.049 | 0.452 | 0.35 |
| TDRD1 | 227.03 | 64.69 | 645.98 | 169.04 | 5.68 | 0.030 | 0.446 | 0.35 |
| LOC729090 | 7446.26 | 3827.47 | 21282.88 | 4769.56 | 6.19 | 0.025 | 0.449 | 0.35 |
| FTHL7 | 10128.42 | 2051.75 | 29001.10 | 4846.28 | 11.66 | 0.007 | 0.450 | 0.35 |
| C10ORF54 | 164.47 | 36.84 | 471.12 | 155.15 | 4.36 | 0.049 | 0.451 | 0.35 |
| GVIN1 | 446.08 | 189.74 | 1279.42 | 92.01 | 12.69 | 0.006 | 0.440 | 0.35 |
| GBP1 | 1658.33 | 621.62 | 4770.78 | 1228.14 | 5.96 | 0.027 | 0.448 | 0.35 |
| SOD2 | 346.41 | 81.86 | 997.88 | 177.01 | 9.04 | 0.012 | 0.455 | 0.35 |
| CD96 | 239.69 | 82.18 | 690.88 | 191.91 | 6.54 | 0.023 | 0.453 | 0.35 |
| SLC7A11 | 139.47 | 25.74 | 402.19 | 97.62 | 4.43 | 0.047 | 0.449 | 0.35 |
| GJC1 | 7998.05 | 3410.00 | 23107.63 | 5784.51 | 9.60 | 0.011 | 0.463 | 0.35 |
| HIST1H2BK | 575.72 | 245.54 | 1663.48 | 499.49 | 7.01 | 0.020 | 0.449 | 0.35 |
| BRI3 | 786.09 | 158.40 | 2275.90 | 593.02 | 5.44 | 0.032 | 0.445 | 0.35 |
| TSC22D3 | 211.44 | 62.91 | 612.86 | 158.05 | 6.33 | 0.024 | 0.452 | 0.34 |
| FCAR | 383.63 | 115.26 | 1112.72 | 179.11 | 13.10 | 0.006 | 0.444 | 0.34 |
| FNDC3B | 214.48 | 51.68 | 623.17 | 158.86 | 6.47 | 0.023 | 0.453 | 0.34 |
| LOC100128062 | 1947.44 | 813.47 | 5675.38 | 1750.05 | 5.70 | 0.029 | 0.445 | 0.34 |
| FTL | 11260.39 | 3505.81 | 32858.90 | 9098.42 | 5.97 | 0.027 | 0.448 | 0.34 |
| TMBIM1 | 274.74 | 54.08 | 803.70 | 203.92 | 6.09 | 0.026 | 0.449 | 0.34 |
| SEPN1 | 206.98 | 2.64 | 605.89 | 150.36 | 4.56 | 0.045 | 0.448 | 0.34 |
| SDHALP1 | 2034.40 | 1068.96 | 5964.56 | 2057.70 | 5.98 | 0.027 | 0.448 | 0.34 |
| XAF1 | 311.58 | 46.69 | 913.58 | 41.13 | 16.32 | 0.004 | 0.454 | 0.34 |
| AHNAK | 606.75 | 245.73 | 1782.19 | 513.48 | 7.60 | 0.017 | 0.445 | 0.34 |
| RASGRP2 | 129.89 | 19.16 | 381.58 | 93.94 | 5.74 | 0.029 | 0.445 | 0.34 |
| MPP1 | 267.66 | 64.16 | 786.48 | 214.93 | 4.74 | 0.042 | 0.445 | 0.34 |
| PLEKHO2 | 268.85 | 84.18 | 790.04 | 188.96 | 7.46 | 0.017 | 0.448 | 0.34 |
| RAB7B | 140.78 | 14.44 | 414.06 | 56.24 | 11.23 | 0.008 | 0.456 | 0.34 |
| SOD2 | 2060.38 | 402.68 | 6065.49 | 1909.34 | 4.57 | 0.045 | 0.448 | 0.34 |
| IL18 | 5563.14 | 3337.69 | 16437.00 | 4838.35 | 10.37 | 0.009 | 0.453 | 0.34 |
| FLJ35390 | 1497.23 | 696.63 | 4426.55 | 1307.32 | 7.18 | 0.019 | 0.449 | 0.34 |
| ATP6AP1 | 1694.31 | 659.75 | 5022.45 | 1646.15 | 5.54 | 0.031 | 0.444 | 0.34 |
| LOC648921 | 414.29 | 151.77 | 1231.65 | 371.14 | 6.32 | 0.024 | 0.451 | 0.34 |
| HS.580797 | 233.15 | 77.74 | 693.29 | 101.25 | 27.63 | 0.001 | 0.451 | 0.34 |
| LOC100190938 | 6791.20 | 4272.58 | 20269.47 | 6276.96 | 6.38 | 0.024 | 0.453 | 0.34 |
| LILRB2 | 168.83 | 32.91 | 507.08 | 113.74 | 6.91 | 0.020 | 0.447 | 0.33 |
| IL10 | 2406.78 | 1225.47 | 7242.00 | 1916.75 | 11.96 | 0.007 | 0.444 | 0.33 |
| ZNF430 | 4921.17 | 2318.98 | 14827.11 | 4116.70 | 9.32 | 0.011 | 0.462 | 0.33 |
| CSTB | 2330.50 | 643.70 | 7023.36 | 2206.82 | 4.67 | 0.043 | 0.447 | 0.33 |
| RAXL1 | 396.14 | 109.75 | 1197.53 | 331.32 | 5.62 | 0.030 | 0.446 | 0.33 |
| LOC100130445 | 3719.72 | 1251.86 | 11327.40 | 2441.35 | 10.00 | 0.010 | 0.453 | 0.33 |
| LOC100130835 | 332.78 | 77.94 | 1014.26 | 218.20 | 6.53 | 0.023 | 0.453 | 0.33 |
| SLC16A12 | 5151.19 | 2919.85 | 15730.67 | 4266.89 | 8.46 | 0.014 | 0.453 | 0.33 |
| PGCP | 143.76 | 24.52 | 442.24 | 124.27 | 4.97 | 0.038 | 0.446 | 0.33 |
| GBP1 | 1780.87 | 930.06 | 5512.07 | 1672.75 | 7.75 | 0.016 | 0.450 | 0.32 |
| SSTR2 | 285.16 | 101.72 | 884.80 | 253.78 | 5.98 | 0.027 | 0.448 | 0.32 |
| NPC2 | 2956.13 | 1261.80 | 9184.58 | 2858.99 | 6.40 | 0.024 | 0.453 | 0.32 |
| ZNF486 | 3973.99 | 1617.92 | 12363.54 | 3844.83 | 6.05 | 0.026 | 0.448 | 0.32 |
| HS.542993 | 395.32 | 165.96 | 1235.95 | 226.36 | 4.87 | 0.040 | 0.445 | 0.32 |
| VPS37C | 855.64 | 441.92 | 2675.29 | 1023.45 | 4.91 | 0.039 | 0.444 | 0.32 |
| TRIM22 | 347.80 | 76.32 | 1088.80 | 305.84 | 5.56 | 0.031 | 0.445 | 0.32 |
| RNF130 | 398.76 | 94.80 | 1252.91 | 300.03 | 6.74 | 0.021 | 0.449 | 0.32 |
| LOC728620 | 4832.84 | 2746.17 | 15194.18 | 4088.55 | 7.07 | 0.019 | 0.449 | 0.32 |
| PIM1 | 555.61 | 159.78 | 1747.45 | 583.05 | 4.79 | 0.041 | 0.444 | 0.32 |
| MBD4 | 687.62 | 250.13 | 2170.77 | 479.19 | 10.43 | 0.009 | 0.455 | 0.32 |
| LOC731542 | 6858.38 | 3413.23 | 21698.21 | 6259.26 | 6.35 | 0.024 | 0.452 | 0.32 |
| FAM46A | 233.74 | 56.29 | 739.89 | 94.47 | 22.20 | 0.002 | 0.451 | 0.32 |
| MCM8 | 4266.26 | 2149.80 | 13505.96 | 4100.54 | 5.52 | 0.031 | 0.445 | 0.32 |
| PATE2 | 241.76 | 78.70 | 766.05 | 160.80 | 7.96 | 0.015 | 0.452 | 0.32 |
| HLA-DRB6 | 949.86 | 233.40 | 3020.45 | 718.06 | 5.77 | 0.029 | 0.444 | 0.31 |
| CREB1 | 1201.21 | 634.18 | 3838.52 | 468.72 | 14.77 | 0.005 | 0.454 | 0.31 |
| C5ORF39 | 219.42 | 29.12 | 703.42 | 149.43 | 6.68 | 0.022 | 0.451 | 0.31 |
| PTPLAD2 | 838.21 | 419.23 | 2687.47 | 1010.77 | 4.70 | 0.042 | 0.447 | 0.31 |
| LILRA6 | 159.75 | 36.31 | 514.69 | 149.58 | 5.12 | 0.036 | 0.447 | 0.31 |
| GSTTP2 | 211.88 | 55.21 | 683.13 | 162.31 | 6.81 | 0.021 | 0.449 | 0.31 |
| MGC26356 | 5396.71 | 3158.82 | 17402.67 | 3879.71 | 19.56 | 0.003 | 0.454 | 0.31 |
| ANKRD22 | 182.11 | 42.15 | 587.78 | 151.60 | 6.38 | 0.024 | 0.453 | 0.31 |
| FAM40B | 317.89 | 115.31 | 1026.30 | 265.98 | 6.99 | 0.020 | 0.449 | 0.31 |
| BLZF1 | 2361.17 | 1220.16 | 7623.28 | 2720.51 | 6.07 | 0.026 | 0.448 | 0.31 |
| LOC100130168 | 3834.74 | 2127.90 | 12399.82 | 3301.86 | 7.90 | 0.016 | 0.450 | 0.31 |
| SAMD9L | 284.76 | 60.22 | 921.06 | 97.66 | 21.23 | 0.002 | 0.447 | 0.31 |
| LOC642947 | 5919.01 | 2906.19 | 19174.56 | 6018.96 | 5.47 | 0.032 | 0.445 | 0.31 |
| C8ORF37 | 2666.19 | 1581.79 | 8641.69 | 2396.79 | 7.91 | 0.016 | 0.451 | 0.31 |
| RTN1 | 132.65 | 24.46 | 430.29 | 124.90 | 4.92 | 0.039 | 0.445 | 0.31 |
| LOC648470 | 154.53 | 18.94 | 502.23 | 49.38 | 10.20 | 0.009 | 0.452 | 0.31 |
| CTSS | 225.82 | 34.40 | 733.96 | 219.48 | 4.75 | 0.042 | 0.445 | 0.31 |
| CREB1 | 5748.98 | 3066.78 | 18686.52 | 5638.49 | 8.03 | 0.015 | 0.452 | 0.31 |
| SYAP1 | 444.51 | 182.52 | 1445.53 | 411.92 | 6.41 | 0.024 | 0.454 | 0.31 |
| CLEC5A | 173.70 | 45.50 | 565.01 | 64.36 | 6.39 | 0.024 | 0.453 | 0.31 |
| POFUT1 | 3270.81 | 1487.95 | 10639.59 | 3071.29 | 7.06 | 0.019 | 0.449 | 0.31 |
| IFNGR2 | 697.36 | 262.80 | 2278.69 | 806.61 | 4.99 | 0.038 | 0.447 | 0.31 |
| GBP5 | 2149.57 | 926.22 | 7025.43 | 2265.00 | 6.20 | 0.025 | 0.449 | 0.31 |
| ZMAT3 | 3572.83 | 1700.71 | 11680.79 | 3157.83 | 4.89 | 0.039 | 0.444 | 0.31 |
| SLC16A3 | 885.81 | 339.61 | 2902.72 | 981.61 | 5.20 | 0.035 | 0.447 | 0.31 |
| C3ORF34 | 1935.75 | 1230.68 | 6344.68 | 1551.41 | 5.42 | 0.032 | 0.445 | 0.31 |
| SAT1 | 1964.76 | 1003.80 | 6448.79 | 2511.98 | 5.11 | 0.036 | 0.447 | 0.30 |
| LOC399900 | 3812.45 | 1985.55 | 12579.08 | 3792.07 | 5.35 | 0.033 | 0.446 | 0.30 |
| LOC729603 | 5669.03 | 3107.80 | 18710.63 | 5805.26 | 4.80 | 0.041 | 0.444 | 0.30 |
| LOC100130276 | 221.06 | 76.22 | 729.81 | 249.73 | 4.33 | 0.049 | 0.452 | 0.30 |
| LOC644250 | 853.07 | 443.41 | 2822.68 | 937.00 | 5.53 | 0.031 | 0.445 | 0.30 |
| GNG7 | 160.90 | 23.06 | 533.01 | 72.12 | 8.85 | 0.013 | 0.457 | 0.30 |
| TMEM140 | 217.75 | 54.26 | 723.85 | 130.00 | 11.28 | 0.008 | 0.456 | 0.30 |
| ANPEP | 160.22 | 22.87 | 533.03 | 51.69 | 10.72 | 0.009 | 0.453 | 0.30 |
| SNAPC1 | 269.12 | 67.46 | 898.69 | 284.70 | 4.51 | 0.046 | 0.449 | 0.30 |
| LOC100130053 | 493.23 | 162.67 | 1647.45 | 452.07 | 5.62 | 0.030 | 0.446 | 0.30 |
| CRCP | 1338.43 | 742.99 | 4499.83 | 1323.64 | 5.97 | 0.027 | 0.448 | 0.30 |
| C21ORF58 | 616.22 | 305.16 | 2079.27 | 639.71 | 4.96 | 0.038 | 0.446 | 0.30 |
| TSC22D3 | 463.31 | 184.69 | 1567.37 | 481.42 | 6.42 | 0.023 | 0.453 | 0.30 |
| TDRD1 | 229.68 | 83.94 | 777.14 | 183.02 | 6.42 | 0.023 | 0.453 | 0.30 |
| LOC100128505 | 8350.69 | 4153.27 | 28355.63 | 8286.72 | 8.24 | 0.014 | 0.452 | 0.29 |
| LRAP | 3957.80 | 2436.26 | 13465.64 | 4002.98 | 4.86 | 0.040 | 0.445 | 0.29 |
| ZNF577 | 410.90 | 192.72 | 1399.13 | 327.01 | 8.99 | 0.012 | 0.454 | 0.29 |
| FLJ46309 | 3373.49 | 2186.63 | 11527.84 | 3490.32 | 6.89 | 0.020 | 0.446 | 0.29 |
| DENR | 418.25 | 215.17 | 1433.32 | 469.72 | 4.95 | 0.038 | 0.445 | 0.29 |
| PYGL | 215.44 | 90.37 | 739.91 | 245.64 | 5.53 | 0.031 | 0.444 | 0.29 |
| LOC100128274 | 182.40 | 45.71 | 627.15 | 90.87 | 16.98 | 0.003 | 0.453 | 0.29 |
| RBM47 | 380.52 | 149.14 | 1310.72 | 473.31 | 4.63 | 0.044 | 0.447 | 0.29 |
| CTSD | 193.77 | 10.39 | 669.63 | 64.67 | 11.54 | 0.007 | 0.448 | 0.29 |
| ZNF394 | 1692.48 | 931.52 | 5874.25 | 1277.38 | 5.48 | 0.032 | 0.445 | 0.29 |
| LOC202781 | 1008.38 | 582.49 | 3505.37 | 924.53 | 8.24 | 0.014 | 0.452 | 0.29 |
| TRIM13 | 404.01 | 190.62 | 1405.63 | 446.22 | 4.38 | 0.048 | 0.451 | 0.29 |
| N4BP2 | 599.21 | 248.77 | 2095.62 | 631.07 | 5.01 | 0.038 | 0.446 | 0.29 |
| TNFSF15 | 256.67 | 57.48 | 898.74 | 237.82 | 5.72 | 0.029 | 0.445 | 0.29 |
| CDKN1A | 2758.17 | 1396.48 | 9661.90 | 2194.76 | 9.42 | 0.011 | 0.464 | 0.29 |
| ZNF786 | 325.28 | 132.88 | 1142.84 | 247.78 | 9.74 | 0.010 | 0.459 | 0.28 |
| ASCL2 | 235.66 | 71.47 | 834.26 | 241.53 | 6.09 | 0.026 | 0.450 | 0.28 |
| PLDN | 1054.88 | 523.57 | 3738.85 | 1298.83 | 5.16 | 0.036 | 0.447 | 0.28 |
| SDCBP | 1015.75 | 527.69 | 3604.89 | 1001.76 | 9.26 | 0.011 | 0.458 | 0.28 |
| HSD17B7 | 1477.95 | 655.34 | 5251.43 | 1686.43 | 5.30 | 0.034 | 0.447 | 0.28 |
| SERPING1 | 159.96 | 21.49 | 568.93 | 108.99 | 7.79 | 0.016 | 0.449 | 0.28 |
| LOC100128084 | 3324.20 | 1757.70 | 11861.77 | 3873.84 | 6.07 | 0.026 | 0.449 | 0.28 |
| LOC100133772 | 1477.30 | 728.13 | 5289.79 | 1198.85 | 9.01 | 0.012 | 0.455 | 0.28 |
| PTPRE | 738.22 | 236.40 | 2648.01 | 391.32 | 9.80 | 0.010 | 0.457 | 0.28 |
| EVI2B | 491.73 | 153.85 | 1764.91 | 277.56 | 17.15 | 0.003 | 0.454 | 0.28 |
| SDCBP | 509.47 | 243.73 | 1837.15 | 594.17 | 5.13 | 0.036 | 0.446 | 0.28 |
| C5AR1 | 210.80 | 43.76 | 760.24 | 128.31 | 5.60 | 0.030 | 0.445 | 0.28 |
| PNPT1 | 1372.30 | 676.05 | 4968.31 | 1697.77 | 5.87 | 0.028 | 0.446 | 0.28 |
| C14ORF85 | 3451.33 | 1833.23 | 12499.50 | 2840.84 | 7.94 | 0.015 | 0.452 | 0.28 |
| LOC728755 | 226.74 | 59.08 | 826.16 | 222.94 | 5.26 | 0.034 | 0.448 | 0.27 |
| RHOU | 220.22 | 51.98 | 803.09 | 221.54 | 5.95 | 0.027 | 0.447 | 0.27 |
| C5ORF28 | 965.14 | 473.19 | 3527.88 | 1040.88 | 7.34 | 0.018 | 0.450 | 0.27 |
| KIAA1598 | 123.91 | 13.88 | 453.59 | 134.61 | 4.56 | 0.045 | 0.448 | 0.27 |
| ABCC3 | 152.10 | 16.58 | 557.33 | 139.29 | 5.10 | 0.036 | 0.446 | 0.27 |
| OCIAD1 | 2486.88 | 1407.99 | 9153.42 | 3248.03 | 5.64 | 0.030 | 0.446 | 0.27 |
| FAM119A | 2268.32 | 1321.16 | 8358.00 | 2099.13 | 9.26 | 0.011 | 0.457 | 0.27 |
| LOC392437 | 4674.90 | 2357.20 | 17355.46 | 3984.61 | 10.85 | 0.008 | 0.456 | 0.27 |
| PIP5K2B | 800.47 | 251.60 | 2972.46 | 860.25 | 5.19 | 0.035 | 0.447 | 0.27 |
| ITM2B | 1416.85 | 748.06 | 5261.44 | 1977.66 | 5.02 | 0.037 | 0.446 | 0.27 |
| TNS3 | 470.17 | 132.50 | 1751.59 | 386.39 | 8.46 | 0.014 | 0.453 | 0.27 |
| NDRG1 | 287.69 | 98.11 | 1073.02 | 380.32 | 4.69 | 0.043 | 0.447 | 0.27 |
| CXCL9 | 1043.03 | 613.99 | 3891.22 | 1646.49 | 4.66 | 0.043 | 0.448 | 0.27 |
| BRSK1 | 199.90 | 20.08 | 747.40 | 104.71 | 10.65 | 0.009 | 0.453 | 0.27 |
| TDP1 | 805.64 | 508.29 | 3038.33 | 566.88 | 46.17 | 0.000 | 0.633 | 0.27 |
| C8ORF45 | 2794.02 | 1670.86 | 10573.15 | 2989.98 | 7.27 | 0.018 | 0.450 | 0.26 |
| IFIT3 | 214.92 | 51.71 | 815.23 | 135.68 | 11.60 | 0.007 | 0.449 | 0.26 |
| CCDC125 | 949.10 | 475.30 | 3607.32 | 1200.74 | 5.89 | 0.028 | 0.446 | 0.26 |
| C9ORF80 | 2584.54 | 1408.11 | 9856.12 | 3129.40 | 4.91 | 0.039 | 0.444 | 0.26 |
| SLC35E1 | 549.69 | 237.05 | 2096.34 | 737.16 | 4.94 | 0.039 | 0.445 | 0.26 |
| NUBPL | 569.64 | 242.65 | 2181.28 | 642.24 | 6.55 | 0.023 | 0.454 | 0.26 |
| DUSP19 | 1840.10 | 1150.42 | 7051.81 | 2314.85 | 5.00 | 0.038 | 0.447 | 0.26 |
| LTBR | 188.10 | 40.57 | 722.46 | 170.90 | 7.10 | 0.019 | 0.449 | 0.26 |
| SLC31A2 | 346.02 | 108.44 | 1331.51 | 372.70 | 5.58 | 0.031 | 0.445 | 0.26 |
| RBM47 | 287.48 | 100.13 | 1106.35 | 395.95 | 4.69 | 0.043 | 0.447 | 0.26 |
| GIMAP4 | 675.18 | 210.70 | 2598.88 | 749.75 | 5.79 | 0.029 | 0.444 | 0.26 |
| SHROOM4 | 765.34 | 409.45 | 2952.70 | 945.00 | 4.58 | 0.045 | 0.448 | 0.26 |
| C2ORF69 | 1978.59 | 978.25 | 7635.89 | 2917.59 | 4.56 | 0.045 | 0.448 | 0.26 |
| HSD11B1 | 130.53 | 18.20 | 505.86 | 127.85 | 5.93 | 0.027 | 0.448 | 0.26 |
| FLJ44124 | 2051.67 | 969.96 | 7959.99 | 2720.85 | 4.89 | 0.039 | 0.444 | 0.26 |
| LOC100129502 | 454.85 | 193.15 | 1774.54 | 493.78 | 6.06 | 0.026 | 0.448 | 0.26 |
| HCG2P7 | 2917.90 | 1395.09 | 11400.75 | 2781.91 | 6.96 | 0.020 | 0.448 | 0.26 |
| FLJ40722 | 620.46 | 254.57 | 2433.45 | 798.22 | 5.71 | 0.029 | 0.445 | 0.25 |
| PILRA | 221.82 | 62.31 | 871.24 | 319.09 | 4.36 | 0.049 | 0.452 | 0.25 |
| LYZ | 3321.53 | 1449.95 | 13046.95 | 3228.03 | 7.00 | 0.020 | 0.449 | 0.25 |
| RIN2 | 148.96 | 45.33 | 585.49 | 170.49 | 5.32 | 0.034 | 0.447 | 0.25 |
| FAM129B | 177.93 | 35.08 | 702.19 | 181.78 | 4.60 | 0.044 | 0.448 | 0.25 |
| MGST1 | 207.47 | 67.02 | 818.86 | 64.93 | 8.07 | 0.015 | 0.453 | 0.25 |
| GNS | 1508.71 | 668.43 | 5977.37 | 1845.97 | 6.08 | 0.026 | 0.449 | 0.25 |
| HS.544637 | 914.84 | 533.84 | 3630.75 | 1290.51 | 4.44 | 0.047 | 0.450 | 0.25 |
| FAM63A | 478.71 | 267.00 | 1906.63 | 358.32 | 9.31 | 0.011 | 0.461 | 0.25 |
| CEBPD | 196.06 | 35.68 | 781.32 | 226.99 | 5.29 | 0.034 | 0.447 | 0.25 |
| PPA2 | 1788.59 | 864.81 | 7135.52 | 2223.95 | 6.38 | 0.024 | 0.453 | 0.25 |
| LOC100129362 | 2707.29 | 1372.47 | 10808.91 | 3125.70 | 6.33 | 0.024 | 0.452 | 0.25 |
| GPR68 | 262.61 | 102.32 | 1050.72 | 265.31 | 7.65 | 0.017 | 0.447 | 0.25 |
| S100A9 | 173.29 | 74.94 | 693.51 | 245.81 | 5.00 | 0.038 | 0.447 | 0.25 |
| PTPRE | 1069.59 | 339.20 | 4284.68 | 1230.43 | 5.97 | 0.027 | 0.447 | 0.25 |
| CEBPB | 1777.04 | 758.78 | 7168.15 | 2198.55 | 6.28 | 0.024 | 0.449 | 0.25 |
| CCR1 | 378.10 | 86.55 | 1544.21 | 321.96 | 6.32 | 0.024 | 0.451 | 0.24 |
| LEP | 249.26 | 95.93 | 1019.95 | 348.99 | 5.19 | 0.035 | 0.447 | 0.24 |
| ZNF738 | 1075.89 | 586.65 | 4405.61 | 1307.31 | 7.39 | 0.018 | 0.449 | 0.24 |
| LOC255167 | 2369.03 | 1421.23 | 9710.93 | 3422.80 | 6.16 | 0.025 | 0.448 | 0.24 |
| HS.163752 | 448.04 | 219.97 | 1840.99 | 469.42 | 9.27 | 0.011 | 0.458 | 0.24 |
| CDKN2AIPNL | 4964.18 | 2812.35 | 20460.58 | 5068.39 | 6.51 | 0.023 | 0.452 | 0.24 |
| EID2B | 2333.43 | 1159.30 | 9619.60 | 3113.07 | 4.97 | 0.038 | 0.446 | 0.24 |
| C14ORF153 | 1247.66 | 617.67 | 5198.33 | 1237.66 | 8.02 | 0.015 | 0.452 | 0.24 |
| ZNF652 | 1260.95 | 520.00 | 5352.34 | 1387.91 | 6.38 | 0.024 | 0.453 | 0.24 |
| QRFPR | 1259.42 | 719.42 | 5346.30 | 1500.35 | 6.31 | 0.024 | 0.451 | 0.24 |
| TMEM51 | 168.69 | 44.66 | 720.27 | 253.44 | 4.38 | 0.048 | 0.451 | 0.23 |
| HNRNPU | 466.16 | 240.75 | 1992.58 | 446.57 | 7.65 | 0.017 | 0.447 | 0.23 |
| C1QC | 138.21 | 20.74 | 593.61 | 119.44 | 7.73 | 0.016 | 0.449 | 0.23 |
| DDX51 | 1602.11 | 805.08 | 6900.14 | 2358.60 | 4.83 | 0.040 | 0.444 | 0.23 |
| FCGR2A | 160.05 | 21.57 | 691.42 | 206.29 | 4.94 | 0.039 | 0.446 | 0.23 |
| SERPINE1 | 191.52 | 73.83 | 828.91 | 167.30 | 5.57 | 0.031 | 0.444 | 0.23 |
| FAM73A | 656.79 | 334.41 | 2853.00 | 1058.27 | 4.57 | 0.045 | 0.448 | 0.23 |
| SEMA3E | 650.49 | 313.50 | 2835.38 | 727.61 | 5.91 | 0.027 | 0.448 | 0.23 |
| ALPP | 1463.29 | 810.63 | 6388.42 | 2498.12 | 4.78 | 0.041 | 0.444 | 0.23 |
| FCN1 | 211.60 | 46.17 | 924.20 | 264.39 | 5.55 | 0.031 | 0.445 | 0.23 |
| PSAP | 2531.09 | 669.68 | 11061.06 | 3118.46 | 6.03 | 0.026 | 0.449 | 0.23 |
| LOC100128288 | 1093.41 | 683.98 | 4799.44 | 1570.86 | 4.81 | 0.041 | 0.444 | 0.23 |
| HIATL2 | 1364.48 | 761.79 | 6000.33 | 2099.50 | 5.08 | 0.037 | 0.447 | 0.23 |
| ABCA1 | 213.84 | 68.22 | 940.99 | 244.05 | 6.95 | 0.020 | 0.447 | 0.23 |
| SULT1A1 | 1000.88 | 485.02 | 4415.45 | 1364.17 | 5.49 | 0.032 | 0.446 | 0.23 |
| ZNF69 | 1440.82 | 906.65 | 6363.27 | 2116.94 | 5.17 | 0.035 | 0.447 | 0.23 |
| FLJ36131 | 1060.81 | 576.16 | 4686.61 | 1525.30 | 6.20 | 0.025 | 0.449 | 0.23 |
| LOC100130516 | 976.27 | 584.13 | 4313.69 | 1390.26 | 5.39 | 0.033 | 0.445 | 0.23 |
| SLC1A3 | 127.32 | 15.45 | 563.37 | 137.51 | 5.84 | 0.028 | 0.445 | 0.23 |
| TLR8 | 191.66 | 33.41 | 850.77 | 209.14 | 6.45 | 0.023 | 0.453 | 0.23 |
| LOC645452 | 3327.33 | 1687.33 | 14773.73 | 5050.77 | 5.88 | 0.028 | 0.446 | 0.23 |
| ZNF549 | 2960.92 | 1854.73 | 13175.47 | 4653.03 | 4.71 | 0.042 | 0.446 | 0.22 |
| DAB2 | 151.90 | 7.51 | 676.87 | 168.19 | 5.41 | 0.032 | 0.445 | 0.22 |
| LOC100133516 | 631.46 | 328.23 | 2813.89 | 875.45 | 6.40 | 0.024 | 0.454 | 0.22 |
| ZNF682 | 1782.77 | 848.23 | 7973.08 | 3048.17 | 4.31 | 0.050 | 0.452 | 0.22 |
| IL17RD | 908.96 | 501.78 | 4076.56 | 1504.76 | 5.09 | 0.036 | 0.446 | 0.22 |
| HSPC268 | 814.98 | 416.65 | 3660.32 | 1004.64 | 6.77 | 0.021 | 0.448 | 0.22 |
| DUXAP3 | 2446.36 | 1580.26 | 10989.81 | 3461.90 | 7.08 | 0.019 | 0.450 | 0.22 |
| LOC100132391 | 2084.40 | 1254.48 | 9371.01 | 3170.01 | 5.45 | 0.032 | 0.445 | 0.22 |
| MAGT1 | 705.77 | 349.69 | 3181.65 | 1084.86 | 4.57 | 0.045 | 0.448 | 0.22 |
| IFIT2 | 285.12 | 92.89 | 1287.33 | 386.16 | 5.90 | 0.028 | 0.447 | 0.22 |
| CYP1B1 | 2335.57 | 1036.79 | 10572.48 | 4017.23 | 4.50 | 0.046 | 0.449 | 0.22 |
| BMS1P5 | 2080.29 | 1297.52 | 9443.14 | 3204.31 | 5.19 | 0.035 | 0.447 | 0.22 |
| SCARB2 | 490.84 | 223.02 | 2228.89 | 397.35 | 15.97 | 0.004 | 0.453 | 0.22 |
| DUSP6 | 706.41 | 337.08 | 3209.55 | 1029.87 | 4.73 | 0.042 | 0.445 | 0.22 |
| DMC1 | 2571.36 | 1644.16 | 11729.12 | 3962.97 | 6.45 | 0.023 | 0.453 | 0.22 |
| CCBE1 | 953.86 | 491.41 | 4361.57 | 1123.09 | 9.05 | 0.012 | 0.454 | 0.22 |
| PSAP | 2373.70 | 1077.75 | 10941.40 | 2468.80 | 10.67 | 0.009 | 0.453 | 0.22 |
| CATSPER2 | 1557.62 | 977.18 | 7195.30 | 2947.82 | 4.66 | 0.043 | 0.448 | 0.22 |
| LOC730313 | 3451.72 | 2398.91 | 15973.79 | 4823.41 | 8.69 | 0.013 | 0.452 | 0.22 |
| TNFSF13B | 252.47 | 65.53 | 1169.50 | 368.29 | 4.83 | 0.040 | 0.444 | 0.22 |
| RXRA | 190.53 | 59.46 | 883.98 | 177.72 | 10.13 | 0.010 | 0.454 | 0.22 |
| FLJ22662 | 213.46 | 38.05 | 993.35 | 319.29 | 4.76 | 0.041 | 0.444 | 0.21 |
| LOC727962 | 170.73 | 16.88 | 795.23 | 259.70 | 4.45 | 0.047 | 0.450 | 0.21 |
| FTHL16 | 205.21 | 72.17 | 956.45 | 275.97 | 6.09 | 0.026 | 0.450 | 0.21 |
| CREG1 | 371.64 | 113.04 | 1743.42 | 553.47 | 5.10 | 0.036 | 0.446 | 0.21 |
| IGSF6 | 147.02 | 26.02 | 690.84 | 145.36 | 7.49 | 0.017 | 0.448 | 0.21 |
| CYBB | 680.70 | 176.59 | 3221.57 | 566.41 | 9.40 | 0.011 | 0.464 | 0.21 |
| ACP5 | 432.56 | 157.26 | 2049.34 | 579.25 | 6.30 | 0.024 | 0.449 | 0.21 |
| DEM1 | 703.83 | 359.33 | 3355.16 | 1263.73 | 4.56 | 0.045 | 0.449 | 0.21 |
| C15ORF63 | 1043.79 | 546.90 | 4977.16 | 1485.62 | 5.16 | 0.036 | 0.446 | 0.21 |
| MMP9 | 2780.28 | 750.95 | 13337.48 | 4427.36 | 4.42 | 0.048 | 0.450 | 0.21 |
| LOC100128510 | 494.81 | 251.01 | 2379.99 | 818.22 | 5.43 | 0.032 | 0.445 | 0.21 |
| LIPA | 1778.15 | 676.38 | 8563.49 | 1884.17 | 8.97 | 0.012 | 0.454 | 0.21 |
| GSN | 270.41 | 45.22 | 1306.37 | 303.19 | 5.32 | 0.034 | 0.447 | 0.21 |
| CSF1R | 650.54 | 152.19 | 3157.22 | 820.00 | 5.78 | 0.029 | 0.443 | 0.21 |
| APOE | 120.51 | 30.46 | 586.66 | 133.71 | 5.57 | 0.031 | 0.445 | 0.21 |
| ZNF483 | 472.62 | 246.27 | 2311.90 | 719.10 | 5.01 | 0.038 | 0.446 | 0.20 |
| LOC728903 | 992.61 | 548.45 | 4882.61 | 1635.58 | 5.46 | 0.032 | 0.445 | 0.20 |
| LOC728809 | 3230.57 | 2047.59 | 15893.79 | 5219.78 | 5.75 | 0.029 | 0.445 | 0.20 |
| FKBP14 | 1119.27 | 588.64 | 5525.18 | 1893.10 | 4.79 | 0.041 | 0.444 | 0.20 |
| CD68 | 1938.76 | 806.28 | 9594.18 | 2326.16 | 7.10 | 0.019 | 0.450 | 0.20 |
| TMEM17 | 1745.47 | 1026.02 | 8692.00 | 2808.18 | 5.74 | 0.029 | 0.445 | 0.20 |
| FTHL2 | 260.83 | 109.17 | 1309.00 | 397.73 | 5.84 | 0.028 | 0.446 | 0.20 |
| FTHL12 | 3306.04 | 2148.17 | 16776.08 | 5823.15 | 4.38 | 0.048 | 0.451 | 0.20 |
| SPTLC1 | 1266.60 | 507.25 | 6446.33 | 2042.12 | 5.83 | 0.028 | 0.445 | 0.20 |
| FTH1 | 683.36 | 356.75 | 3494.71 | 894.95 | 6.16 | 0.025 | 0.448 | 0.20 |
| LOC100131718 | 532.31 | 283.90 | 2750.44 | 893.64 | 6.13 | 0.026 | 0.449 | 0.19 |
| LOC645452 | 1565.51 | 985.67 | 8111.09 | 2604.80 | 6.89 | 0.020 | 0.446 | 0.19 |
| HSD11B1 | 140.84 | 12.10 | 737.00 | 181.03 | 5.90 | 0.028 | 0.447 | 0.19 |
| LRRC37B2 | 1216.06 | 621.60 | 6383.13 | 2227.71 | 4.94 | 0.039 | 0.446 | 0.19 |
| EPB41L3 | 163.62 | 40.04 | 866.23 | 263.99 | 5.01 | 0.038 | 0.446 | 0.19 |
| ZNF14 | 2565.78 | 1469.11 | 13646.12 | 4762.67 | 5.68 | 0.030 | 0.446 | 0.19 |
| FAM175A | 1085.17 | 583.42 | 5771.78 | 2054.49 | 4.97 | 0.038 | 0.446 | 0.19 |
| MCART1 | 2151.01 | 1080.38 | 11459.45 | 4081.72 | 4.88 | 0.040 | 0.444 | 0.19 |
| CD68 | 865.49 | 410.61 | 4647.83 | 1129.54 | 9.09 | 0.012 | 0.457 | 0.19 |
| USP49 | 515.11 | 220.52 | 2787.88 | 966.28 | 5.21 | 0.035 | 0.447 | 0.18 |
| CEBPA | 218.98 | 76.66 | 1189.01 | 279.67 | 7.95 | 0.015 | 0.452 | 0.18 |
| FPR3 | 372.06 | 54.90 | 2025.06 | 593.21 | 5.12 | 0.036 | 0.447 | 0.18 |
| LOC100132585 | 915.53 | 515.13 | 4999.33 | 1991.91 | 4.47 | 0.047 | 0.449 | 0.18 |
| CXCL16 | 457.39 | 205.80 | 2498.17 | 692.77 | 4.92 | 0.039 | 0.445 | 0.18 |
| PLD3 | 526.66 | 193.09 | 2909.68 | 922.30 | 5.66 | 0.030 | 0.446 | 0.18 |
| IDO1 | 728.37 | 391.58 | 4049.25 | 1695.19 | 4.41 | 0.048 | 0.450 | 0.18 |
| NLRP8 | 1089.95 | 655.09 | 6247.94 | 2233.41 | 5.62 | 0.030 | 0.445 | 0.17 |
| CTSB | 871.52 | 331.88 | 5006.29 | 1031.99 | 10.23 | 0.009 | 0.453 | 0.17 |
| TXNIP | 1331.08 | 423.73 | 7688.94 | 2157.14 | 6.35 | 0.024 | 0.451 | 0.17 |
| FGL2 | 702.84 | 346.24 | 4068.53 | 1433.78 | 5.13 | 0.036 | 0.447 | 0.17 |
| LOC642567 | 253.31 | 74.60 | 1481.71 | 321.30 | 7.88 | 0.016 | 0.450 | 0.17 |
| TIMP2 | 246.39 | 42.69 | 1455.54 | 466.34 | 4.92 | 0.039 | 0.445 | 0.17 |
| LILRB3 | 463.04 | 208.72 | 2737.14 | 798.77 | 6.42 | 0.023 | 0.453 | 0.17 |
| SLAMF8 | 352.30 | 115.20 | 2108.46 | 458.85 | 8.54 | 0.013 | 0.452 | 0.17 |
| IL18BP | 654.54 | 214.59 | 3954.30 | 1389.32 | 4.66 | 0.043 | 0.448 | 0.17 |
| KIAA1751 | 1187.77 | 731.92 | 7183.46 | 2787.70 | 4.81 | 0.041 | 0.444 | 0.17 |
| CD36 | 178.27 | 46.28 | 1079.50 | 396.55 | 4.37 | 0.049 | 0.451 | 0.17 |
| RARRES3 | 502.65 | 154.55 | 3051.02 | 609.48 | 9.62 | 0.011 | 0.463 | 0.16 |
| FCGRT | 208.94 | 45.92 | 1271.05 | 171.12 | 14.69 | 0.005 | 0.447 | 0.16 |
| ALDH1A1 | 142.47 | 19.39 | 898.46 | 225.16 | 5.82 | 0.028 | 0.444 | 0.16 |
| LHFPL2 | 293.18 | 94.12 | 1910.27 | 638.53 | 4.80 | 0.041 | 0.444 | 0.15 |
| PLA2G7 | 841.53 | 391.95 | 5702.43 | 1705.04 | 6.35 | 0.024 | 0.451 | 0.15 |
| KLF2 | 511.07 | 263.82 | 3542.05 | 1013.56 | 6.93 | 0.020 | 0.447 | 0.14 |
| FTHL3 | 749.88 | 322.83 | 5253.59 | 1666.07 | 5.06 | 0.037 | 0.447 | 0.14 |
| CTSB | 239.48 | 59.26 | 1711.06 | 404.24 | 7.27 | 0.018 | 0.451 | 0.14 |
| CECR1 | 338.24 | 66.65 | 2567.81 | 432.10 | 10.44 | 0.009 | 0.455 | 0.13 |
| TYROBP | 759.07 | 344.49 | 5790.76 | 961.28 | 10.89 | 0.008 | 0.456 | 0.13 |
| GPNMB | 134.64 | 11.19 | 1047.45 | 235.48 | 6.95 | 0.020 | 0.447 | 0.13 |
| CCL2 | 957.23 | 279.49 | 7521.02 | 2020.06 | 6.50 | 0.023 | 0.452 | 0.13 |
| CST3 | 279.03 | 60.23 | 2314.45 | 620.92 | 6.26 | 0.025 | 0.449 | 0.12 |
| C1ORF162 | 214.77 | 33.32 | 1829.41 | 392.88 | 7.54 | 0.017 | 0.446 | 0.12 |
| FCER1G | 473.98 | 143.24 | 4343.89 | 1211.75 | 6.07 | 0.026 | 0.449 | 0.11 |
| ALDH1A1 | 131.66 | 27.34 | 1419.73 | 352.10 | 6.78 | 0.021 | 0.448 | 0.09 |
| CYP27A1 | 224.79 | 43.12 | 2560.21 | 657.58 | 6.58 | 0.022 | 0.454 | 0.09 |
