# Supplementary material for: Regulation of IL-17A Production Is Distinct from IL-17F in a Primary Human Cell Co-culture Model of T Cell-Mediated B Cell Activation
Source: PLoS One. 2013 Mar 7;8(3):e58966. doi: 10.1371/journal.pone.0058966 (PMC3591360; doi:10.1371/journal.pone.0058966)
Supplement: Table S2 — Quantitative RT-PCR Th17 Array data set for purified CD4 T cells and B cells isolated from stimulated and non-stimulated BT co-cultures. Quantitative RT-PCR gene expression data from CD4 T and B cell populations purified by FACS after three days in co-culture with α-IgM and SAg stimulation. Data normalized to b-actin expression levels and presented as fold change (above) and raw Ct values (below) are shown. Gene expression data are from 3 independent donor pools of stimulated BT co-cultures compared to cells isolated from non-stimulated BT co-cultures. (DOC) [file pone.0058966.s002.doc]

**Table S2. Quantitative RT-PCR Th17 Array data set for purified CD4 T cells and B cells isolated from stimulated and non-stimulated BT co-cultures.** Quantitative RT-PCR gene expression data from CD4 T and B cell populations purified by FACS after three days in co-culture with α-IgM and SAg stimulation. Data normalized to b-actin expression levels and presented as fold change (above) and raw Ct values (below) are shown. Gene expression data are from 3 independent donor pools of stimulated BT co-cultures compared to cells isolated from non-stimulated BT co-cultures.

| *Fold Changes* |  |  |  |  |  |  |  |  |  |  |  |  |  |  |
| --- | --- | --- | --- | --- | --- | --- | --- | --- | --- | --- | --- | --- | --- | --- |
|  | CD4 T cells | CD4 T cells | CD4 T cells |  |  |  |  |  | B cells | B cells | B cells |  |  |  |
| Gene Symbol | Donor 1 | Donor 2 | Donor 3 | Average Fold Change | Std Dev | t-test |  | Gene Symbol | Donor 1 | Donor 2 | Donor 3 | Average Fold Change | Std Dev | t-test |
| CACYBP | 0.755 | 0.732 | 0.458 | 0.648 | 0.165 | 2.1E-02 |  | CACYBP | 1.172 | 1.716 | 1.872 | 1.587 | 0.368 | 5.1E-02 |
| CCL1 | 0.677 | 0.166 | 0.097 | 0.313 | 0.317 | 2.0E-02 |  | CCL1 | 55.225 | 201.528 | 59.771 | 105.508 | 83.187 | 9.5E-02 |
| CCL2 | 0.050 | 0.010 | 0.002 | 0.021 | 0.026 | 3.4E-07 |  | CCL2 | 3.507 | 56.495 | 17.070 | 25.691 | 27.526 | 2.0E-01 |
| CCL20 | 0.611 | 2.844 | 0.402 | 1.286 | 1.354 | 7.3E-01 |  | CCL20 | 1.642 | 7.532 | 4.656 | 4.610 | 2.945 | 1.0E-01 |
| CCL22 | 0.556 | 0.322 | 0.114 | 0.331 | 0.221 | 6.3E-03 |  | CCL22 | 243.724 | 421.888 | 358.176 | 341.263 | 90.278 | 2.8E-03 |
| CCL7 | 0.013 | 0.025 | 0.023 | 0.020 | 0.006 | 1.0E-09 |  | CCL7 | 0.009 | 0.004 | 0.006 | 0.006 | 0.002 | 2.5E-11 |
| CD247 | 0.257 | 0.203 | 0.210 | 0.223 | 0.029 | 1.4E-06 |  | CD247 | 0.079 | 0.119 | 0.289 | 0.162 | 0.111 | 2.0E-04 |
| CD28 | 0.423 | 0.362 | 0.533 | 0.439 | 0.087 | 3.6E-04 |  | CD28 | 0.119 | 0.053 | 0.077 | 0.083 | 0.034 | 1.2E-06 |
| CD34 | 0.013 | 0.025 | 0.023 | 0.020 | 0.006 | 1.0E-09 |  | CD34 | 0.009 | 0.228 | 0.006 | 0.081 | 0.128 | 2.4E-04 |
| CD3D | 0.338 | 0.258 | 0.183 | 0.260 | 0.077 | 7.7E-05 |  | CD3D | 0.452 | 0.885 | 1.908 | 1.082 | 0.748 | 8.6E-01 |
| CD3E | 0.284 | 0.398 | 0.169 | 0.284 | 0.114 | 4.1E-04 |  | CD3E | 0.255 | 0.744 | 1.728 | 0.909 | 0.750 | 8.4E-01 |
| CD3G | 0.304 | 0.303 | 0.191 | 0.266 | 0.065 | 4.0E-05 |  | CD3G | 0.278 | 0.504 | 1.212 | 0.665 | 0.487 | 3.0E-01 |
| CD4 | 0.569 | 0.434 | 0.694 | 0.565 | 0.130 | 4.4E-03 |  | CD4 | 0.091 | 0.456 | 0.190 | 0.246 | 0.189 | 2.3E-03 |
| CD40LG | 0.737 | 1.020 | 0.472 | 0.743 | 0.274 | 1.8E-01 |  | CD40LG | 0.202 | 0.505 | 0.770 | 0.492 | 0.284 | 3.6E-02 |
| CD8A | 0.213 | 0.190 | 0.175 | 0.193 | 0.019 | 2.0E-07 |  | CD8A | 0.248 | 1.005 | 2.563 | 1.272 | 1.180 | 7.1E-01 |
| CEBPB | 0.262 | 0.181 | 0.269 | 0.237 | 0.049 | 1.1E-05 |  | CEBPB | 0.301 | 0.303 | 0.451 | 0.352 | 0.086 | 2.0E-04 |
| CLEC7A | 0.068 | 0.183 | 0.003 | 0.085 | 0.091 | 6.5E-05 |  | CLEC7A | 0.001 | 0.000 | 0.001 | 0.001 | 0.000 | 6.3E-15 |
| CSF2 | 0.824 | 0.476 | 0.373 | 0.558 | 0.236 | 3.2E-02 |  | CSF2 | 56.105 | 136.602 | 180.550 | 124.419 | 63.111 | 2.8E-02 |
| CSF3 | 0.013 | 0.025 | 0.023 | 0.020 | 0.006 | 1.0E-09 |  | CSF3 | 0.736 | 0.024 | 0.412 | 0.391 | 0.356 | 4.2E-02 |
| CX3CL1 | 0.391 | 0.010 | 0.754 | 0.385 | 0.372 | 4.6E-02 |  | CX3CL1 | 13.563 | 20.268 | 11.860 | 15.230 | 4.445 | 5.2E-03 |
| CXCL1 | 0.032 | 0.077 | 0.106 | 0.072 | 0.037 | 1.7E-06 |  | CXCL1 | 0.007 | 0.003 | 0.004 | 0.005 | 0.002 | 8.3E-12 |
| CXCL12 | 0.000 | 0.053 | 0.001 | 0.018 | 0.031 | 6.2E-07 |  | CXCL12 | 0.005 | 0.002 | 0.192 | 0.066 | 0.109 | 1.2E-04 |
| CXCL2 | 0.013 | 0.025 | 0.023 | 0.020 | 0.006 | 1.0E-09 |  | CXCL2 | 0.010 | 0.004 | 0.006 | 0.007 | 0.003 | 4.3E-11 |
| CXCL5 | 0.000 | 0.001 | 0.003 | 0.001 | 0.001 | 1.4E-12 |  | CXCL5 | 0.111 | 0.022 | 0.102 | 0.078 | 0.049 | 5.3E-06 |
| CXCL6 | 0.013 | 1.120 | 0.023 | 0.386 | 0.636 | 1.7E-01 |  | CXCL6 | 0.004 | 0.002 | 0.003 | 0.003 | 0.001 | 1.4E-12 |
| S1PR1 | 0.196 | 0.128 | 0.156 | 0.160 | 0.034 | 1.8E-06 |  | S1PR1 | 0.119 | 0.065 | 0.127 | 0.104 | 0.034 | 1.4E-06 |
| FOXP3 | 0.273 | 0.418 | 0.226 | 0.306 | 0.100 | 2.8E-04 |  | FOXP3 | 0.479 | 0.458 | 0.623 | 0.520 | 0.090 | 7.6E-04 |
| GATA3 | 0.323 | 0.308 | 0.358 | 0.329 | 0.026 | 1.4E-06 |  | GATA3 | 0.208 | 0.160 | 0.586 | 0.318 | 0.233 | 7.1E-03 |
| ICAM1 | 0.330 | 0.227 | 0.198 | 0.251 | 0.069 | 4.8E-05 |  | ICAM1 | 4.222 | 6.326 | 6.335 | 5.628 | 1.217 | 2.8E-03 |
| ICOS | 1.274 | 0.255 | 0.928 | 0.819 | 0.518 | 5.8E-01 |  | ICOS | 1.635 | 2.037 | 5.546 | 3.073 | 2.152 | 1.7E-01 |
| IFNG | 1.019 | 1.264 | 0.584 | 0.956 | 0.344 | 8.3E-01 |  | IFNG | 0.593 | 0.970 | 1.830 | 1.131 | 0.634 | 7.4E-01 |
| IL10 | 0.172 | 0.078 | 0.168 | 0.139 | 0.053 | 9.5E-06 |  | IL10 | 0.121 | 0.589 | 0.364 | 0.358 | 0.234 | 8.9E-03 |
| IL12B | 0.013 | 0.025 | 0.173 | 0.070 | 0.089 | 5.5E-05 |  | IL12B | 0.128 | 0.001 | 0.001 | 0.043 | 0.074 | 2.3E-05 |
| IL12RB1 | 0.249 | 0.161 | 0.182 | 0.197 | 0.046 | 7.0E-06 |  | IL12RB1 | 0.986 | 1.162 | 1.492 | 1.213 | 0.257 | 2.2E-01 |
| IL12RB2 | 0.597 | 0.812 | 0.367 | 0.592 | 0.223 | 3.4E-02 |  | IL12RB2 | 7.027 | 12.359 | 19.285 | 12.890 | 6.146 | 2.9E-02 |
| IL13 | 0.763 | 0.698 | 0.410 | 0.623 | 0.188 | 2.6E-02 |  | IL13 | 1.894 | 0.806 | 7.671 | 3.457 | 3.690 | 3.1E-01 |
| IL15 | 0.149 | 0.161 | 0.039 | 0.116 | 0.068 | 2.2E-05 |  | IL15 | 0.192 | 0.223 | 0.221 | 0.212 | 0.017 | 1.5E-07 |
| IL17A | 0.241 | 1.988 | 1.050 | 1.093 | 0.874 | 8.6E-01 |  | IL17A | 2.142 | 0.040 | 0.057 | 0.746 | 1.209 | 7.3E-01 |
| IL17C | 0.053 | 0.067 | 0.010 | 0.043 | 0.029 | 6.0E-07 |  | IL17C | 0.057 | 0.046 | 0.079 | 0.061 | 0.017 | 7.2E-08 |
| IL17D | 0.143 | 0.505 | 0.765 | 0.471 | 0.313 | 4.3E-02 |  | IL17D | 0.377 | 0.104 | 0.065 | 0.182 | 0.170 | 1.1E-03 |
| IL17F | 36.815 | 52.999 | 18.932 | 36.249 | 17.041 | 2.3E-02 |  | IL17F | 0.606 | 9.092 | 5.595 | 5.098 | 4.265 | 1.7E-01 |
| IL17RB | 0.096 | 0.143 | 0.171 | 0.137 | 0.038 | 2.5E-06 |  | IL17RB | 0.398 | 0.452 | 0.544 | 0.465 | 0.074 | 2.3E-04 |
| IL17RC | 0.287 | 0.378 | 0.455 | 0.373 | 0.084 | 2.0E-04 |  | IL17RC | 1.246 | 2.528 | 4.195 | 2.656 | 1.478 | 1.2E-01 |
| IL17RD | 0.013 | 0.672 | 0.023 | 0.236 | 0.377 | 2.5E-02 |  | IL17RD | 0.005 | 0.002 | 0.429 | 0.145 | 0.245 | 3.8E-03 |
| IL17RE | 0.421 | 0.376 | 0.200 | 0.332 | 0.116 | 5.8E-04 |  | IL17RE | 0.302 | 0.388 | 0.183 | 0.291 | 0.103 | 2.9E-04 |
| IL18 | 0.435 | 0.625 | 0.218 | 0.426 | 0.204 | 8.2E-03 |  | IL18 | 0.370 | 0.430 | 0.579 | 0.460 | 0.108 | 9.7E-04 |
| IL1B | 0.097 | 0.186 | 0.093 | 0.125 | 0.053 | 8.7E-06 |  | IL1B | 0.004 | 0.485 | 0.586 | 0.358 | 0.311 | 2.3E-02 |
| IL2 | 1.015 | 1.867 | 0.497 | 1.126 | 0.692 | 7.7E-01 |  | IL2 | 0.004 | 0.155 | 0.503 | 0.221 | 0.256 | 6.2E-03 |
| IL21 | 2.388 | 2.461 | 1.562 | 2.137 | 0.499 | 1.7E-02 |  | IL21 | 3.315 | 1.098 | 3.653 | 2.689 | 1.388 | 1.0E-01 |
| IL22 | 26.978 | 31.725 | 26.523 | 28.409 | 2.881 | 7.9E-05 |  | IL22 | 0.002 | 0.001 | 0.001 | 0.001 | 0.001 | 8.1E-14 |
| IL23A | 0.193 | 0.175 | 0.143 | 0.170 | 0.025 | 5.8E-07 |  | IL23A | 0.181 | 0.138 | 0.125 | 0.148 | 0.029 | 9.4E-07 |
| IL23R | 3.836 | 4.892 | 2.072 | 3.600 | 1.425 | 3.4E-02 |  | IL23R | 0.151 | 0.145 | 0.284 | 0.193 | 0.078 | 5.8E-05 |
| IL25 | 0.001 | 0.001 | 0.001 | 0.001 | 0.000 | 2.9E-15 |  | IL25 | 0.023 | 0.199 | 0.015 | 0.079 | 0.104 | 1.0E-04 |
| IL27 | 0.924 | 1.319 | 0.747 | 0.997 | 0.293 | 9.9E-01 |  | IL27 | 0.827 | 0.721 | 0.890 | 0.813 | 0.085 | 1.9E-02 |
| IL3 | 0.111 | 0.066 | 0.166 | 0.114 | 0.050 | 6.6E-06 |  | IL3 | 0.059 | 1.324 | 0.921 | 0.768 | 0.647 | 5.7E-01 |
| IL4 | 0.692 | 0.025 | 0.023 | 0.247 | 0.386 | 2.8E-02 |  | IL4 | 0.007 | 0.003 | 0.004 | 0.005 | 0.002 | 7.9E-12 |
| IL5 | 1.863 | 0.591 | 1.886 | 1.446 | 0.741 | 3.6E-01 |  | IL5 | 2.081 | 0.261 | 5.228 | 2.523 | 2.513 | 3.5E-01 |
| IL6 | 0.514 | 0.818 | 0.023 | 0.452 | 0.401 | 7.7E-02 |  | IL6 | 0.037 | 0.066 | 0.069 | 0.057 | 0.017 | 7.4E-08 |
| IL6R | 0.058 | 0.038 | 0.042 | 0.046 | 0.011 | 1.0E-08 |  | IL6R | 0.236 | 0.145 | 0.100 | 0.160 | 0.069 | 3.1E-05 |
| IL7R | 0.034 | 0.045 | 0.051 | 0.043 | 0.009 | 4.9E-09 |  | IL7R | 0.080 | 0.173 | 0.352 | 0.202 | 0.138 | 5.6E-04 |
| IL8 | 0.218 | 0.106 | 0.135 | 0.153 | 0.058 | 1.5E-05 |  | IL8 | 0.001 | 0.061 | 0.044 | 0.035 | 0.031 | 7.4E-07 |
| ISG20 | 0.077 | 0.066 | 0.036 | 0.060 | 0.021 | 1.8E-07 |  | ISG20 | 0.243 | 0.150 | 0.216 | 0.203 | 0.048 | 8.6E-06 |
| JAK1 | 0.122 | 0.133 | 0.071 | 0.109 | 0.033 | 1.3E-06 |  | JAK1 | 0.149 | 0.166 | 0.180 | 0.165 | 0.015 | 7.3E-08 |
| JAK2 | 0.219 | 0.319 | 0.174 | 0.237 | 0.074 | 5.8E-05 |  | JAK2 | 0.522 | 0.588 | 0.635 | 0.581 | 0.057 | 2.1E-04 |
| MMP13 | 0.001 | 0.072 | 0.002 | 0.025 | 0.040 | 1.9E-06 |  | MMP13 | 0.003 | 0.001 | 0.002 | 0.002 | 0.001 | 3.8E-13 |
| MMP3 | 0.013 | 0.025 | 0.023 | 0.020 | 0.006 | 1.0E-09 |  | MMP3 | 0.017 | 0.008 | 0.011 | 0.012 | 0.005 | 3.7E-10 |
| MMP9 | 0.054 | 0.034 | 0.006 | 0.031 | 0.024 | 2.6E-07 |  | MMP9 | 0.276 | 0.258 | 0.549 | 0.361 | 0.163 | 2.5E-03 |
| NFATC2 | 0.645 | 0.413 | 0.467 | 0.508 | 0.121 | 2.2E-03 |  | NFATC2 | 0.277 | 0.277 | 0.285 | 0.280 | 0.005 | 1.4E-09 |
| NFKB1 | 0.638 | 0.729 | 0.396 | 0.588 | 0.172 | 1.4E-02 |  | NFKB1 | 0.817 | 1.127 | 1.093 | 1.012 | 0.170 | 9.1E-01 |
| RORC | 0.322 | 0.317 | 0.212 | 0.284 | 0.062 | 3.7E-05 |  | RORC | 1.829 | 1.987 | 2.584 | 2.134 | 0.398 | 7.9E-03 |
| SOCS1 | 0.177 | 0.246 | 0.078 | 0.167 | 0.085 | 7.0E-05 |  | SOCS1 | 2.349 | 2.195 | 3.017 | 2.521 | 0.437 | 3.8E-03 |
| SOCS3 | 0.146 | 0.158 | 0.147 | 0.151 | 0.006 | 2.2E-09 |  | SOCS3 | 1.197 | 1.463 | 1.569 | 1.410 | 0.191 | 2.1E-02 |
| STAT3 | 0.717 | 0.563 | 0.611 | 0.631 | 0.079 | 1.3E-03 |  | STAT3 | 1.072 | 1.520 | 1.723 | 1.438 | 0.333 | 8.5E-02 |
| STAT4 | 0.438 | 0.416 | 0.142 | 0.332 | 0.165 | 2.2E-03 |  | STAT4 | 5.866 | 10.353 | 17.082 | 11.100 | 5.645 | 3.6E-02 |
| STAT5A | 0.256 | 0.218 | 0.163 | 0.212 | 0.047 | 8.2E-06 |  | STAT5A | 0.812 | 1.051 | 1.164 | 1.009 | 0.179 | 9.4E-01 |
| STAT6 | 0.380 | 0.219 | 0.288 | 0.296 | 0.080 | 1.1E-04 |  | STAT6 | 0.330 | 0.345 | 0.292 | 0.322 | 0.027 | 1.7E-06 |
| SYK | 0.087 | 0.137 | 0.109 | 0.111 | 0.025 | 4.2E-07 |  | SYK | 0.145 | 0.407 | 0.201 | 0.251 | 0.138 | 7.1E-04 |
| TBX21 | 0.676 | 0.718 | 0.569 | 0.654 | 0.077 | 1.5E-03 |  | TBX21 | 1.241 | 2.144 | 1.885 | 1.757 | 0.465 | 4.8E-02 |
| TGFB1 | 0.266 | 0.276 | 0.134 | 0.225 | 0.079 | 7.1E-05 |  | TGFB1 | 0.336 | 0.414 | 0.452 | 0.401 | 0.059 | 6.3E-05 |
| TIRAP | 0.170 | 0.117 | 0.088 | 0.125 | 0.042 | 3.4E-06 |  | TIRAP | 0.277 | 0.269 | 0.306 | 0.284 | 0.019 | 3.6E-07 |
| TLR4 | 0.013 | 2.974 | 0.023 | 1.004 | 1.707 | 1.0E+00 |  | TLR4 | 0.143 | 0.081 | 0.179 | 0.134 | 0.050 | 7.2E-06 |
| TNF | 0.186 | 0.162 | 0.164 | 0.171 | 0.014 | 4.8E-08 |  | TNF | 0.482 | 0.974 | 0.924 | 0.793 | 0.271 | 2.6E-01 |
| TRAF6 | 0.213 | 0.209 | 0.173 | 0.198 | 0.022 | 3.7E-07 |  | TRAF6 | 0.204 | 0.336 | 0.269 | 0.269 | 0.066 | 4.4E-05 |
| YY1 | 0.260 | 0.356 | 0.172 | 0.263 | 0.092 | 1.6E-04 |  | YY1 | 0.385 | 0.472 | 0.514 | 0.457 | 0.066 | 1.4E-04 |
| B2M | 0.122 | 0.103 | 0.101 | 0.109 | 0.012 | 2.1E-08 |  | B2M | 0.281 | 0.309 | 0.336 | 0.309 | 0.027 | 1.6E-06 |
| HPRT1 | 0.675 | 0.858 | 0.417 | 0.650 | 0.221 | 5.2E-02 |  | HPRT1 | 1.042 | 1.673 | 1.844 | 1.520 | 0.422 | 1.0E-01 |
| RPL13A | 0.241 | 0.243 | 0.112 | 0.199 | 0.075 | 5.1E-05 |  | RPL13A | 0.409 | 0.392 | 0.560 | 0.453 | 0.093 | 5.2E-04 |
| ACTB | 1.000 | 1.000 | 1.000 | 1.000 | 0.000 | N/A |  | ACTB | 1.000 | 1.000 | 1.000 | 1.000 | 0.000 | N/A |

| *Raw Ct Values* |  |  |  |  |  |  |  |  |  |  |  |  |
| --- | --- | --- | --- | --- | --- | --- | --- | --- | --- | --- | --- | --- |
| Cell Type | CD4 T | CD4 T | CD4 T | CD4 T | CD4 T | CD4 T | B | B | B | B | B | B |
| Condition | Stim | Control | Stim | Control | Stim | Control | Stim | Control | Stim | Control | Stim | Control |
| Gene Symbol | 1 | 1 | 2 | 2 | 3 | 3 | 1 | 1 | 2 | 2 | 3 | 3 |
| CACYBP | 21.13 | 27.03 | 22.06 | 27.46 | 22.66 | 25.65 | 22.89 | 24.43 | 21.17 | 24.64 | 21.57 | 24.75 |
| CCL1 | 27.63 | 35.01 | 30.54 | 32.32 | 31.24 | 31.41 | 31.15 | 40.00 | 28.11 | 40.00 | 30.39 | 40.00 |
| CCL2 | 27.96 | 30.32 | 31.24 | 29.18 | 33.62 | 29.96 | 30.57 | 31.97 | 25.39 | 40.00 | 27.64 | 40.00 |
| CCL20 | 25.00 | 30.52 | 23.67 | 30.88 | 26.41 | 29.70 | 31.81 | 34.33 | 28.44 | 32.67 | 29.66 | 40.00 |
| CCL22 | 20.77 | 25.88 | 22.44 | 27.58 | 23.86 | 25.70 | 21.68 | 30.40 | 19.72 | 31.97 | 20.48 | 31.22 |
| CCL7 | 40.00 | 40.00 | 40.00 | 40.00 | 40.00 | 40.00 | 40.00 | 34.40 | 40.00 | 40.00 | 40.00 | 34.02 |
| CD247 | 23.01 | 28.42 | 24.23 | 26.75 | 24.11 | 25.16 | 29.92 | 27.08 | 28.15 | 27.93 | 27.40 | 28.24 |
| CD28 | 31.46 | 40.00 | 32.57 | 40.00 | 31.93 | 32.78 | 40.00 | 40.00 | 40.00 | 40.00 | 40.00 | 40.00 |
| CD34 | 40.00 | 40.00 | 40.00 | 40.00 | 40.00 | 40.00 | 40.00 | 40.00 | 34.11 | 40.00 | 40.00 | 33.47 |
| CD3D | 21.52 | 26.55 | 22.80 | 26.18 | 23.22 | 24.63 | 28.38 | 28.25 | 26.24 | 28.95 | 25.66 | 28.97 |
| CD3E | 22.12 | 26.72 | 22.52 | 26.79 | 23.68 | 25.14 | 30.01 | 29.09 | 27.30 | 29.72 | 26.61 | 29.79 |
| CD3G | 22.10 | 26.87 | 23.00 | 26.78 | 23.58 | 25.13 | 29.39 | 28.37 | 27.37 | 29.23 | 26.63 | 29.55 |
| CD4 | 24.15 | 30.47 | 25.43 | 29.24 | 24.67 | 27.49 | 33.29 | 30.98 | 29.80 | 31.08 | 31.58 | 31.86 |
| CD40LG | 22.49 | 28.31 | 22.90 | 28.66 | 23.94 | 27.23 | 31.42 | 30.17 | 28.93 | 30.24 | 28.85 | 31.46 |
| CD8A | 30.53 | 40.00 | 31.58 | 33.49 | 31.62 | 31.95 | 30.92 | 29.68 | 27.74 | 30.96 | 26.91 | 30.71 |
| CEBPB | 25.77 | 30.34 | 27.19 | 30.33 | 26.54 | 28.39 | 26.75 | 26.34 | 25.58 | 26.55 | 25.53 | 26.65 |
| CLEC7A | 34.76 | 40.00 | 34.22 | 35.38 | 40.00 | 40.00 | 40.00 | 30.19 | 40.00 | 32.42 | 40.00 | 34.17 |
| CSF2 | 21.83 | 27.55 | 23.51 | 29.29 | 23.79 | 26.98 | 28.08 | 35.15 | 25.63 | 40.00 | 25.75 | 34.76 |
| CSF3 | 40.00 | 40.00 | 40.00 | 40.00 | 40.00 | 40.00 | 36.25 | 35.55 | 40.00 | 40.00 | 36.44 | 40.00 |
| CX3CL1 | 33.79 | 39.96 | 40.00 | 37.35 | 33.65 | 38.98 | 33.17 | 38.15 | 31.43 | 39.21 | 32.73 | 39.49 |
| CXCL1 | 35.80 | 40.00 | 35.44 | 35.33 | 34.89 | 40.00 | 40.00 | 33.23 | 40.00 | 35.11 | 40.00 | 34.87 |
| CXCL12 | 40.00 | 34.58 | 33.92 | 40.00 | 40.00 | 34.82 | 40.00 | 34.60 | 40.00 | 37.82 | 33.96 | 32.78 |
| CXCL2 | 40.00 | 40.00 | 40.00 | 40.00 | 40.00 | 40.00 | 40.00 | 35.98 | 40.00 | 34.02 | 40.00 | 35.25 |
| CXCL5 | 38.85 | 33.30 | 37.68 | 32.98 | 36.43 | 37.96 | 36.99 | 33.88 | 38.15 | 40.00 | 36.48 | 35.98 |
| CXCL6 | 40.00 | 40.00 | 34.49 | 40.00 | 40.00 | 40.00 | 40.00 | 33.79 | 40.00 | 40.00 | 40.00 | 32.83 |
| S1PR1 | 23.43 | 27.89 | 24.93 | 27.17 | 24.57 | 25.46 | 25.66 | 24.17 | 25.38 | 23.61 | 24.93 | 24.53 |
| FOXP3 | 25.90 | 31.31 | 26.17 | 29.62 | 26.98 | 28.38 | 30.81 | 31.73 | 29.71 | 31.09 | 29.79 | 31.14 |
| GATA3 | 27.51 | 33.15 | 28.47 | 31.45 | 28.17 | 30.32 | 33.91 | 33.84 | 33.13 | 33.59 | 31.78 | 32.66 |
| ICAM1 | 23.80 | 28.67 | 25.23 | 28.81 | 25.35 | 26.76 | 23.89 | 26.53 | 22.14 | 27.80 | 22.66 | 28.25 |
| ICOS | 24.18 | 31.33 | 27.38 | 30.58 | 25.44 | 28.96 | 32.27 | 40.00 | 30.78 | 33.70 | 29.86 | 34.37 |
| IFNG | 21.67 | 27.57 | 22.25 | 29.40 | 23.29 | 28.44 | 27.71 | 28.69 | 25.83 | 27.81 | 25.44 | 28.99 |
| IL10 | 29.32 | 32.95 | 31.35 | 33.89 | 30.16 | 31.83 | 32.94 | 31.88 | 29.49 | 32.17 | 30.71 | 30.86 |
| IL12B | 40.00 | 40.00 | 40.00 | 40.00 | 37.11 | 40.00 | 33.45 | 31.71 | 40.00 | 33.84 | 40.00 | 31.54 |
| IL12RB1 | 24.72 | 29.47 | 26.23 | 28.78 | 25.98 | 27.23 | 25.10 | 26.20 | 23.70 | 26.94 | 23.87 | 26.65 |
| IL12RB2 | 24.13 | 29.45 | 24.57 | 30.65 | 25.64 | 28.80 | 26.26 | 29.74 | 24.28 | 30.66 | 24.17 | 31.43 |
| IL13 | 22.78 | 28.38 | 23.80 | 30.25 | 24.49 | 27.76 | 30.31 | 32.19 | 30.37 | 32.45 | 27.65 | 33.55 |
| IL15 | 28.83 | 32.29 | 29.61 | 33.37 | 31.59 | 30.87 | 28.29 | 26.84 | 26.91 | 27.95 | 27.45 | 27.55 |
| IL17A | 35.82 | 40.00 | 33.66 | 40.00 | 34.50 | 40.00 | 35.41 | 36.38 | 40.00 | 40.00 | 40.00 | 40.00 |
| IL17C | 31.59 | 33.37 | 32.15 | 34.51 | 34.76 | 33.27 | 31.00 | 28.69 | 30.15 | 27.68 | 29.88 | 28.94 |
| IL17D | 36.57 | 40.00 | 35.64 | 40.00 | 34.96 | 40.00 | 35.57 | 34.51 | 36.25 | 36.70 | 37.46 | 36.36 |
| IL17F | 22.15 | 40.00 | 22.51 | 33.25 | 23.92 | 30.42 | 34.31 | 40.00 | 29.24 | 40.00 | 30.47 | 33.92 |
| IL17RB | 28.81 | 32.85 | 29.12 | 32.10 | 28.78 | 28.84 | 28.46 | 28.53 | 27.11 | 28.41 | 27.36 | 28.90 |
| IL17RC | 33.40 | 40.00 | 33.89 | 36.20 | 33.55 | 40.00 | 31.67 | 32.66 | 29.48 | 34.38 | 29.28 | 33.73 |
| IL17RD | 40.00 | 40.00 | 35.23 | 40.00 | 40.00 | 40.00 | 40.00 | 33.76 | 40.00 | 40.00 | 32.96 | 33.18 |
| IL17RE | 29.36 | 40.00 | 30.41 | 32.77 | 31.24 | 32.98 | 32.66 | 32.78 | 31.13 | 31.76 | 32.74 | 32.95 |
| IL18 | 31.61 | 40.00 | 31.97 | 40.00 | 33.41 | 32.99 | 30.56 | 29.85 | 29.17 | 30.84 | 29.27 | 31.23 |
| IL1B | 32.64 | 40.00 | 32.59 | 33.66 | 33.51 | 40.00 | 40.00 | 32.52 | 31.91 | 32.92 | 32.16 | 40.00 |
| IL2 | 23.55 | 29.64 | 23.55 | 30.18 | 25.39 | 30.13 | 40.00 | 32.95 | 33.47 | 40.00 | 32.30 | 32.94 |
| IL21 | 24.02 | 31.20 | 24.87 | 40.00 | 25.44 | 30.20 | 31.80 | 33.13 | 32.22 | 40.00 | 31.01 | 40.00 |
| IL22 | 26.98 | 40.00 | 27.64 | 40.00 | 27.82 | 34.56 | 40.00 | 34.68 | 40.00 | 32.00 | 40.00 | 32.48 |
| IL23A | 27.74 | 32.48 | 28.77 | 31.33 | 28.99 | 29.48 | 28.89 | 27.63 | 28.12 | 27.78 | 28.78 | 28.28 |
| IL23R | 27.81 | 37.02 | 28.34 | 35.19 | 29.51 | 34.43 | 34.82 | 33.58 | 33.71 | 40.00 | 33.27 | 32.85 |
| IL25 | 40.00 | 34.83 | 40.00 | 40.00 | 40.00 | 40.00 | 40.00 | 34.97 | 35.74 | 35.67 | 40.00 | 40.00 |
| IL27 | 32.73 | 40.00 | 33.10 | 40.00 | 33.84 | 35.84 | 33.47 | 32.95 | 32.50 | 39.09 | 32.72 | 36.78 |
| IL3 | 29.75 | 32.47 | 31.38 | 34.93 | 29.98 | 32.33 | 38.00 | 40.00 | 32.34 | 34.98 | 33.39 | 35.00 |
| IL4 | 34.29 | 40.00 | 40.00 | 40.00 | 40.00 | 40.00 | 40.00 | 40.00 | 40.00 | 40.00 | 40.00 | 33.07 |
| IL5 | 25.69 | 40.00 | 28.23 | 32.09 | 26.48 | 29.90 | 30.07 | 31.82 | 31.90 | 33.59 | 28.10 | 32.83 |
| IL6 | 34.73 | 40.00 | 34.94 | 40.00 | 40.00 | 40.00 | 31.27 | 28.28 | 29.29 | 29.33 | 29.75 | 27.45 |
| IL6R | 26.92 | 29.95 | 28.42 | 28.68 | 28.21 | 26.99 | 27.86 | 26.57 | 27.40 | 27.48 | 28.46 | 27.81 |
| IL7R | 25.68 | 27.72 | 26.13 | 26.66 | 25.88 | 25.23 | 31.66 | 29.58 | 29.38 | 29.11 | 28.88 | 29.86 |
| IL8 | 28.14 | 32.74 | 30.07 | 32.12 | 29.64 | 30.24 | 40.00 | 30.22 | 31.95 | 32.09 | 32.95 | 30.25 |
| ISG20 | 24.76 | 27.53 | 25.88 | 27.55 | 26.68 | 25.76 | 23.68 | 23.20 | 23.21 | 23.18 | 23.21 | 23.09 |
| JAK1 | 23.75 | 27.22 | 24.51 | 26.97 | 25.33 | 25.56 | 24.30 | 22.86 | 22.99 | 23.01 | 23.40 | 23.26 |
| JAK2 | 26.38 | 30.84 | 26.72 | 30.30 | 27.51 | 28.85 | 25.42 | 25.74 | 24.08 | 25.89 | 24.50 | 26.25 |
| MMP13 | 40.00 | 40.00 | 35.17 | 34.93 | 40.00 | 40.00 | 40.00 | 33.01 | 40.00 | 36.01 | 40.00 | 32.55 |
| MMP3 | 40.00 | 40.00 | 40.00 | 40.00 | 40.00 | 40.00 | 40.00 | 33.75 | 40.00 | 40.00 | 40.00 | 40.00 |
| MMP9 | 27.99 | 30.22 | 29.58 | 29.52 | 31.90 | 30.78 | 33.50 | 32.26 | 32.43 | 33.70 | 31.86 | 33.64 |
| NFATC2 | 26.25 | 32.47 | 27.78 | 31.81 | 27.53 | 29.85 | 27.53 | 26.68 | 26.36 | 27.31 | 26.84 | 27.50 |
| NFKB1 | 22.21 | 27.86 | 22.91 | 28.42 | 23.71 | 26.37 | 23.62 | 24.45 | 21.99 | 24.73 | 22.56 | 25.24 |
| RORC | 27.26 | 31.68 | 28.16 | 33.09 | 28.67 | 30.90 | 32.28 | 33.70 | 30.99 | 34.00 | 31.14 | 40.00 |
| SOCS1 | 22.62 | 26.17 | 23.03 | 27.57 | 24.62 | 25.49 | 23.56 | 26.62 | 22.49 | 26.00 | 22.55 | 26.36 |
| SOCS3 | 26.35 | 30.59 | 27.12 | 29.52 | 27.15 | 27.82 | 26.43 | 29.50 | 24.97 | 29.08 | 25.39 | 27.44 |
| STAT3 | 22.73 | 33.66 | 23.96 | 27.74 | 23.77 | 25.61 | 22.99 | 24.53 | 21.32 | 24.61 | 21.67 | 24.65 |
| STAT4 | 23.82 | 28.75 | 24.78 | 29.39 | 26.26 | 28.44 | 27.21 | 30.71 | 25.22 | 31.28 | 25.03 | 31.74 |
| STAT5A | 22.32 | 26.71 | 23.44 | 27.22 | 23.78 | 24.97 | 23.45 | 24.54 | 21.91 | 24.50 | 22.29 | 24.85 |
| STAT6 | 23.63 | 29.31 | 25.31 | 28.32 | 24.84 | 26.27 | 23.57 | 22.98 | 22.33 | 23.23 | 23.10 | 24.14 |
| SYK | 32.37 | 35.75 | 32.60 | 34.10 | 32.86 | 35.59 | 25.77 | 24.90 | 23.11 | 23.61 | 24.65 | 25.32 |
| TBX21 | 24.73 | 31.45 | 25.53 | 30.44 | 25.79 | 27.88 | 24.37 | 25.26 | 22.42 | 27.45 | 23.13 | 26.42 |
| TGFB1 | 21.61 | 25.97 | 22.44 | 26.51 | 23.40 | 24.61 | 22.88 | 22.37 | 21.41 | 22.80 | 21.81 | 23.19 |
| TIRAP | 27.69 | 31.52 | 29.12 | 31.24 | 29.45 | 30.76 | 28.42 | 27.89 | 27.29 | 27.96 | 27.63 | 28.29 |
| TLR4 | 40.00 | 40.00 | 33.08 | 40.00 | 40.00 | 40.00 | 30.84 | 30.23 | 30.50 | 29.25 | 29.88 | 29.46 |
| TNF | 23.38 | 26.87 | 24.46 | 28.82 | 24.37 | 26.85 | 25.44 | 26.87 | 23.26 | 24.48 | 23.86 | 28.80 |
| TRAF6 | 26.78 | 30.98 | 27.69 | 31.07 | 27.89 | 29.27 | 27.36 | 26.52 | 25.47 | 26.51 | 26.31 | 26.64 |
| YY1 | 23.28 | 27.51 | 23.72 | 28.21 | 24.69 | 26.67 | 24.31 | 24.07 | 22.85 | 24.62 | 23.25 | 24.58 |
| B2M | 18.57 | 21.87 | 19.71 | 22.26 | 19.66 | 20.39 | 18.99 | 18.50 | 17.69 | 18.68 | 18.10 | 18.78 |
| HPRT1 | 23.02 | 28.59 | 23.56 | 29.59 | 24.52 | 27.63 | 24.55 | 25.86 | 22.70 | 25.83 | 23.09 | 26.58 |
| RPL13A | 19.32 | 23.47 | 20.19 | 24.22 | 21.24 | 22.33 | 19.97 | 19.85 | 18.87 | 20.36 | 18.88 | 20.32 |
| ACTB | 17.71 | 24.92 | 18.59 | 23.63 | 18.51 | 21.74 | 17.93 | 19.09 | 16.77 | 19.31 | 17.29 | 19.82 |
