# Supplementary material for: Regulation of IL-17A Production Is Distinct from IL-17F in a Primary Human Cell Co-culture Model of T Cell-Mediated B Cell Activation
Source: PLoS One. 2013 Mar 7;8(3):e58966. doi: 10.1371/journal.pone.0058966 (PMC3591360; doi:10.1371/journal.pone.0058966)
Supplement: Table S3 — Screen of agents in stimulated BT co-cultures. Agents screened are listed with the concentrations tested, supplier, and putative mechanism of action. Data for each agent screened on B cell proliferation, PBMC cytotoxicity, IgG, IL-17A, IL-17F, IL-2, IL-6 and TNFα. Data are presented as the Log10 ratio of values from agent-treated stimulated BT co-cultures to values from control stimulated BT co-cultures. (DOC) [file pone.0058966.s003.doc]

**Table S3. Screen of agents in stimulated BT co-cultures.** Agents screened are listed with the concentrations tested, supplier, and putative mechanism of action. Data for each agent screened on B cell proliferation, PBMC cytotoxicity, IgG, IL-17A, IL-17F, IL-2, IL-6 and TNFα. Data are presented as the Log_10_ ratio of values from agent-treated stimulated BT co-cultures to values from control stimulated BT co-cultures.

| Agent | Concentration | Supplier | Mechanism of Action | B cell Prolif. | PBMC Cyto-toxicity | sIgG | sIL-17A | sIL-17F | sIL-2 | sIL-6 | sTNF-alpha |
| --- | --- | --- | --- | --- | --- | --- | --- | --- | --- | --- | --- |
| (+/-)-Nicotine | 4.00E1 ng/ml | Sigma | Nicotinic Agonist | -0.161 | -0.075 | -0.345 | -0.112 | -0.347 | -0.140 | -0.091 | -0.198 |
| (+/-)-Nicotine | 2.00E1 ng/ml |  |  | -0.106 | -0.060 | -0.076 | -0.161 | -0.224 | -0.077 | -0.084 | -0.144 |
| (+/-)-Nicotine | 1.00E1 ng/ml |  |  | -0.113 | -0.063 | -0.025 | -0.111 | -0.144 | -0.094 | -0.060 | -0.157 |
| (+/-)-Nicotine | 5.00E0 ng/ml |  |  | -0.165 | -0.081 | -0.395 | -0.223 | -0.313 | -0.248 | -0.105 | -0.216 |
| 15(R)-15-methyl Prostaglandin D2 | 1.00E4 nM | Cayman Chemical | DP Agonist | -0.035 | -0.027 | -0.017 | -0.088 | -0.060 | -0.031 | 0.030 | -0.120 |
| 15(R)-15-methyl Prostaglandin D2 | 3.33E3 nM |  |  | -0.051 | -0.008 | 0.115 | 0.071 | -0.003 | 0.039 | 0.028 | -0.032 |
| 15(R)-15-methyl Prostaglandin D2 | 1.11E3 nM |  |  | -0.011 | -0.016 | 0.002 | 0.071 | 0.073 | 0.092 | 0.052 | 0.026 |
| 15(R)-15-methyl Prostaglandin D2 | 3.70E2 nM |  |  | 0.010 | 0.008 | -0.060 | -0.055 | 0.046 | 0.130 | -0.063 | 0.043 |
| 17-AAG | 3.33E3 nM | Tocris Cookson | Hsp90 Inhibitor | -0.680 | -0.201 | -1.679 | -0.475 | -0.750 | -0.678 | -0.840 | -1.315 |
| 17-AAG | 1.11E3 nM |  |  | -0.409 | -0.124 | -1.641 | -0.320 | -0.601 | -0.015 | -0.785 | -0.672 |
| 17-AAG | 3.70E2 nM |  |  | -0.055 | -0.058 | -1.226 | -0.036 | -0.144 | -0.001 | -0.052 | -0.073 |
| 17-AAG | 1.24E2 nM |  |  | -0.005 | -0.003 | -0.378 | -0.041 | -0.054 | -0.003 | 0.201 | 0.054 |
| 17-AAG | 4.12E1 nM |  |  | -0.019 | 0.011 | -0.031 | -0.031 | 0.003 | -0.001 | -0.289 | 0.051 |
| 17-AAG | 1.37E1 nM |  |  | 0.014 | 0.001 | 0.016 | 0.088 | 0.043 | -0.013 | 0.112 | 0.053 |
| 17-AAG | 4.60E0 nM |  |  | 0.011 | -0.006 | 0.005 | -0.033 | -0.014 | 0.016 | -0.050 | 0.031 |
| 17-AAG | 1.50E0 nM |  |  | -0.001 | 0.000 | -0.081 | -0.125 | -0.060 | 0.049 | -0.073 | 0.066 |
| 22(R)-Hydroxycholesterol | 1.00E4 nM | Sigma | LXRalpha Agonist | 0.021 | 0.049 | -1.681 | -0.447 | -0.611 | -0.130 | 0.052 | -0.117 |
| 22(R)-Hydroxycholesterol | 3.33E3 nM |  |  | -0.011 | 0.020 | -0.874 | -0.061 | -0.279 | -0.125 | 0.117 | -0.080 |
| 22(R)-Hydroxycholesterol | 1.11E3 nM |  |  | 0.008 | 0.011 | -0.002 | -0.079 | -0.085 | -0.007 | 0.000 | -0.031 |
| 22(R)-Hydroxycholesterol | 3.70E2 nM |  |  | -0.003 | 0.001 | 0.057 | 0.018 | 0.029 | 0.053 | 0.004 | -0.003 |
| 24(S)-Hydroxycholesterol | 1.00E3 nM | Sigma | LXRalpha Agonist | -0.079 | 0.021 | -1.517 | -0.215 | -0.296 | 0.040 | 0.074 | -0.030 |
| 24(S)-Hydroxycholesterol | 3.33E2 nM |  |  | 0.013 | 0.009 | -0.085 | 0.111 | 0.044 | -0.051 | 0.029 | -0.031 |
| 24(S)-Hydroxycholesterol | 1.11E2 nM |  |  | 0.002 | -0.009 | 0.082 | -0.085 | -0.043 | 0.028 | -0.026 | -0.002 |
| 24(S)-Hydroxycholesterol | 3.70E1 nM |  |  | 0.029 | 0.016 | 0.047 | -0.057 | 0.027 | 0.032 | -0.001 | 0.027 |
| 4-Aminobenzoic Hydrazide | 1.00E5 nM | Biomol | Myeloperoxidase Inhibitor | 0.015 | 0.012 | -0.023 | 0.127 | 0.025 | -0.020 | 0.033 | -0.004 |
| 4-Aminobenzoic Hydrazide | 3.33E4 nM |  |  | 0.006 | 0.005 | -0.008 | 0.075 | 0.066 | -0.050 | 0.021 | -0.020 |
| 4-Aminobenzoic Hydrazide | 1.11E4 nM |  |  | -0.023 | 0.015 | 0.007 | 0.090 | 0.046 | -0.016 | 0.022 | 0.010 |
| 4-Aminobenzoic Hydrazide | 3.70E3 nM |  |  | -0.019 | 0.003 | 0.000 | -0.064 | -0.056 | 0.019 | -0.047 | 0.031 |
| 8-CPT-cAMP | 5.00E4 nM | EMD | PKA Activator | -0.185 | -0.053 | -0.867 | 0.118 | -0.144 | 0.192 | 0.234 | -0.746 |
| 8-CPT-cAMP | 1.67E4 nM |  |  | -0.142 | -0.048 | -0.444 | 0.159 | 0.075 | -0.089 | 0.242 | -0.571 |
| 8-CPT-cAMP | 5.56E3 nM |  |  | -0.064 | -0.017 | -0.199 | 0.063 | 0.088 | -0.064 | 0.174 | -0.229 |
| 8-CPT-cAMP | 1.85E3 nM |  |  | -0.018 | 0.004 | -0.047 | 0.019 | -0.024 | 0.113 | 0.007 | -0.054 |
| AEB071 | 1.11E3 nM | Axon Medchem | PKCtheta | -0.355 | -0.145 | -0.779 | -0.834 | -1.059 | -0.948 | -0.799 | -1.289 |
| AEB071 | 3.70E2 nM |  |  | -0.127 | -0.074 | -0.326 | -0.547 | -0.675 | -0.860 | -0.576 | -0.468 |
| AEB071 | 1.24E2 nM |  |  | -0.044 | -0.019 | -0.042 | -0.213 | -0.271 | -0.721 | -0.266 | -0.180 |
| AEB071 | 4.12E1 nM |  |  | 0.001 | -0.008 | 0.027 | -0.138 | -0.237 | -0.428 | -0.128 | -0.116 |
| AMG548 | 1.00E3 nM | Amgen | p38 MAPK | -0.209 | -0.181 | -1.562 | -0.662 | -0.438 | -0.038 | -0.532 | -0.511 |
| AMG548 | 3.33E2 nM |  |  | -0.072 | -0.113 | -1.295 | -0.588 | -0.234 | 0.147 | -0.271 | -0.218 |
| AMG548 | 1.11E2 nM |  |  | -0.019 | -0.118 | -0.832 | -0.519 | 0.000 | 0.132 | -0.350 | -0.224 |
| AMG548 | 3.70E1 nM |  |  | -0.039 | -0.084 | -0.570 | -0.452 | -0.148 | 0.097 | -0.299 | -0.211 |
| AS601245 | 1.00E4 nM | EMD | JNK | -0.314 | -0.165 | -1.555 | -0.355 | -0.446 | -0.508 | -0.574 | -0.673 |
| AS601245 | 3.33E3 nM |  |  | 0.014 | -0.059 | -0.383 | -0.123 | -0.319 | -0.063 | -0.064 | -0.125 |
| AS601245 | 1.11E3 nM |  |  | 0.017 | -0.008 | 0.041 | -0.009 | -0.088 | 0.015 | -0.003 | 0.003 |
| AS601245 | 3.70E2 nM |  |  | 0.023 | -0.003 | 0.031 | -0.008 | 0.024 | 0.018 | -0.043 | 0.029 |
| AS601245 | 1.24E2 nM |  |  | -0.036 | -0.014 | 0.013 | -0.067 | -0.001 | 0.032 | -0.062 | 0.041 |
| AS601245 | 4.12E1 nM |  |  | -0.007 | -0.015 | -0.001 | -0.140 | -0.077 | 0.024 | -0.037 | 0.048 |
| AS601245 | 1.37E1 nM |  |  | 0.000 | -0.012 | -0.021 | 0.058 | 0.030 | 0.002 | -0.013 | 0.052 |
| AS601245 | 4.60E0 nM |  |  | -0.031 | -0.007 | -0.079 | -0.183 | -0.072 | 0.045 | -0.076 | 0.065 |
| AS605240 | 1.00E4 nM | Cayman Chemical | PI3K | -0.007 | -0.004 | -0.109 | -0.146 | -0.090 | 0.112 | -0.012 | 0.100 |
| AS605240 | 3.33E3 nM |  |  | -0.018 | 0.010 | -0.090 | -0.020 | 0.085 | 0.056 | 0.053 | 0.072 |
| AS605240 | 1.11E3 nM |  |  | 0.005 | -0.005 | -0.027 | -0.035 | -0.041 | 0.070 | 0.042 | 0.047 |
| AS605240 | 3.70E2 nM |  |  | 0.007 | 0.010 | -0.054 | -0.036 | -0.041 | 0.084 | -0.019 | 0.059 |
| AS605240 | 1.24E2 nM |  |  | -0.014 | 0.011 | -0.134 | -0.102 | 0.008 | 0.070 | 0.013 | 0.067 |
| AS605240 | 4.12E1 nM |  |  | -0.024 | 0.029 | 0.002 | -0.032 | -0.037 | 0.054 | -0.030 | 0.060 |
| AS605240 | 1.37E1 nM |  |  | 0.006 | 0.001 | -0.015 | -0.102 | -0.108 | 0.019 | -0.046 | 0.029 |
| AS605240 | 4.60E0 nM |  |  | -0.053 | 0.026 | -0.139 | -0.089 | -0.016 | 0.088 | -0.073 | 0.058 |
| AS703026 | 3.00E4 nM | Selleck Chem | MEK | -0.586 | -0.198 | -1.549 | -0.336 | -0.453 | -0.794 | -0.944 | -1.531 |
| AS703026 | 1.00E4 nM |  |  | -0.469 | -0.152 | -1.367 | -0.338 | -0.441 | -0.787 | -0.961 | -1.453 |
| AS703026 | 3.33E3 nM |  |  | -0.415 | -0.139 | -0.678 | -0.395 | -0.459 | -0.733 | -0.821 | -1.352 |
| AS703026 | 1.11E3 nM |  |  | -0.350 | -0.133 | -0.445 | -0.414 | -0.544 | -0.738 | -0.766 | -1.325 |
| AS703026 | 3.70E2 nM |  |  | -0.293 | -0.125 | -0.135 | -0.442 | -0.503 | -0.695 | -0.617 | -1.131 |
| AS703026 | 1.24E2 nM |  |  | -0.245 | -0.122 | -0.276 | -0.417 | -0.424 | -0.575 | -0.549 | -0.892 |
| AS703026 | 4.12E1 nM |  |  | -0.158 | -0.104 | -0.349 | -0.295 | -0.289 | -0.410 | -0.382 | -0.617 |
| AS703026 | 1.37E1 nM |  |  | -0.064 | -0.055 | -0.343 | -0.335 | -0.289 | -0.310 | -0.340 | -0.473 |
| AS703026 | 4.60E0 nM |  |  | -0.052 | -0.036 | -0.146 | -0.372 | -0.295 | -0.182 | -0.297 | -0.347 |
| AS703026 | 1.50E0 nM |  |  | -0.060 | -0.012 | -0.077 | -0.177 | -0.153 | -0.002 | -0.160 | -0.072 |
| AZD-4547 | 1.00E4 nM | Active Biochem | FGFR | -0.089 | -0.043 | -0.002 | -0.138 | -0.132 | 0.073 | -0.013 | -0.011 |
| AZD-4547 | 3.33E3 nM |  |  | 0.026 | 0.009 | 0.109 | -0.051 | 0.016 | -0.032 | -0.010 | 0.009 |
| AZD-4547 | 1.11E3 nM |  |  | 0.035 | 0.012 | 0.094 | 0.076 | 0.074 | -0.002 | 0.003 | 0.000 |
| AZD-4547 | 3.70E2 nM |  |  | 0.006 | -0.002 | 0.049 | 0.082 | 0.015 | -0.012 | 0.162 | -0.019 |
| AZD8055 | 1.11E3 nM | Active Biochem | mTOR | -0.606 | -0.224 | -1.638 | -0.392 | -0.439 | -0.736 | -1.109 | -1.171 |
| AZD8055 | 3.70E2 nM |  |  | -0.568 | -0.194 | -1.596 | -0.395 | -0.403 | -0.698 | -1.029 | -1.001 |
| AZD8055 | 1.24E2 nM |  |  | -0.498 | -0.186 | -1.648 | -0.301 | -0.375 | -0.504 | -0.979 | -0.676 |
| AZD8055 | 4.12E1 nM |  |  | -0.345 | -0.121 | -1.554 | -0.274 | -0.370 | -0.311 | -0.857 | -0.445 |
| AZD8055 | 1.37E1 nM |  |  | -0.234 | -0.108 | -0.675 | -0.267 | -0.214 | -0.077 | -0.664 | -0.263 |
| AZD8055 | 4.60E0 nM |  |  | -0.147 | -0.060 | -0.528 | -0.239 | -0.152 | -0.071 | -0.425 | -0.157 |
| AZD8055 | 1.50E0 nM |  |  | -0.018 | -0.041 | -0.206 | -0.186 | -0.096 | -0.048 | -0.263 | -0.071 |
| AZD8055 | 5.10E-1 nM |  |  | -0.054 | -0.019 | -0.072 | -0.095 | -0.057 | 0.039 | -0.115 | 0.013 |
| Actinomycin D | 4.12E1 nM | Sigma | RNA Polymerase | -1.098 | -0.668 | -1.744 | -0.493 | -0.624 | -0.761 | -0.325 | -1.144 |
| Actinomycin D | 1.37E1 nM |  |  | -1.047 | -0.513 | -1.857 | -0.493 | -0.793 | -0.737 | -0.466 | -1.181 |
| Actinomycin D | 4.60E0 nM |  |  | -0.648 | -0.147 | -1.864 | -0.417 | -0.678 | -0.040 | -0.492 | -0.655 |
| Actinomycin D | 1.50E0 nM |  |  | -0.402 | -0.074 | -1.797 | -0.382 | -0.544 | 0.108 | -0.440 | -0.426 |
| Actinomycin D | 5.10E-1 nM |  |  | -0.252 | -0.051 | -1.675 | -0.323 | -0.240 | 0.140 | -0.299 | -0.163 |
| Actinomycin D | 1.70E-1 nM |  |  | -0.096 | 0.020 | -0.790 | -0.104 | -0.126 | 0.101 | -0.197 | 0.012 |
| Actinomycin D | 5.60E-2 nM |  |  | -0.009 | 0.004 | 0.051 | -0.077 | -0.017 | 0.128 | -0.070 | 0.090 |
| Actinomycin D | 1.90E-2 nM |  |  | 0.018 | 0.034 | 0.112 | -0.121 | 0.017 | 0.114 | -0.066 | 0.046 |
| Adenosine | 1.50E4 nM | Sigma | A1 Agonist | -0.031 | -0.012 | 0.029 | -0.116 | -0.040 | -0.007 | -0.038 | -0.013 |
| Adenosine | 1.00E4 nM |  |  | -0.015 | 0.005 | -0.019 | -0.026 | 0.028 | 0.023 | 0.023 | 0.011 |
| Adenosine | 5.00E3 nM |  |  | -0.007 | -0.017 | -0.028 | -0.058 | -0.011 | -0.036 | -0.018 | -0.020 |
| Adenosine | 1.67E3 nM |  |  | 0.012 | 0.014 | 0.055 | -0.067 | -0.031 | -0.036 | -0.025 | -0.004 |
| Adenosine | 5.56E2 nM |  |  | 0.002 | 0.043 | -0.003 | -0.091 | -0.050 | -0.006 | -0.060 | -0.012 |
| Aldosterone | 1.00E4 nM | Toronto Research Chemicals | MR Agonist | -0.022 | -0.014 | -0.132 | -0.078 | -0.148 | 0.053 | -0.062 | 0.001 |
| Aldosterone | 3.33E3 nM |  |  | -0.051 | -0.010 | -0.021 | -0.109 | -0.154 | 0.041 | -0.009 | 0.005 |
| Aldosterone | 1.11E3 nM |  |  | -0.067 | -0.018 | 0.020 | -0.082 | -0.031 | 0.070 | -0.029 | 0.013 |
| Aldosterone | 3.70E2 nM |  |  | -0.023 | -0.015 | -0.018 | -0.054 | 0.031 | 0.048 | -0.020 | 0.000 |
| Aldosterone | 1.24E2 nM |  |  | -0.005 | -0.017 | -0.012 | -0.071 | -0.088 | 0.025 | -0.003 | 0.027 |
| Aldosterone | 4.12E1 nM |  |  | -0.118 | -0.097 | -0.083 | -0.185 | -0.313 | -0.146 | -0.132 | -0.120 |
| Aldosterone | 1.37E1 nM |  |  | -0.124 | -0.073 | -0.022 | -0.290 | -0.335 | -0.126 | -0.148 | -0.132 |
| Aldosterone | 4.60E0 nM |  |  | -0.086 | -0.066 | -0.066 | -0.200 | -0.276 | -0.125 | -0.219 | -0.141 |
| Apoptolidin | 1.00E3 nM | Sigma | F0F1-ATPase | -0.341 | -0.077 | -1.760 | -0.153 | -0.508 | -0.350 | -0.322 | -0.541 |
| Apoptolidin | 3.33E2 nM |  |  | -0.218 | -0.038 | -1.623 | -0.210 | -0.375 | -0.258 | -0.191 | -0.292 |
| Apoptolidin | 1.11E2 nM |  |  | -0.078 | -0.017 | -1.365 | -0.002 | -0.099 | -0.141 | -0.064 | -0.157 |
| Apoptolidin | 3.70E1 nM |  |  | -0.020 | -0.021 | -0.115 | 0.023 | 0.006 | -0.046 | 0.005 | -0.047 |
| Apoptolidin | 1.23E1 nM |  |  | -0.007 | -0.006 | 0.064 | 0.106 | -0.001 | -0.022 | -0.057 | 0.021 |
| Apoptolidin | 4.10E0 nM |  |  | 0.036 | -0.016 | 0.060 | 0.063 | 0.045 | 0.040 | 0.316 | 0.032 |
| Apoptolidin | 1.40E0 nM |  |  | -0.012 | 0.019 | -0.020 | -0.024 | -0.044 | -0.024 | -0.034 | 0.041 |
| Apoptolidin | 4.60E-1 nM |  |  | -0.012 | 0.002 | 0.052 | 0.075 | 0.058 | 0.031 | -0.011 | -0.102 |
| Atorvastatin | 3.33E3 nM | Cayman | HMG-CoA Reductase Inhibitor | -0.286 | -0.061 | -1.594 | -0.162 | -0.043 | 0.045 | -0.170 | -0.149 |
| Atorvastatin | 1.11E3 nM |  |  | -0.115 | -0.029 | -1.024 | -0.099 | 0.073 | 0.027 | 0.017 | 0.003 |
| Atorvastatin | 3.70E2 nM |  |  | -0.036 | -0.032 | -0.604 | -0.036 | 0.049 | -0.043 | 0.173 | 0.022 |
| Atorvastatin | 1.24E2 nM |  |  | -0.048 | -0.001 | -0.219 | -0.086 | -0.042 | 0.006 | 0.167 | 0.039 |
| Axitinib | 9.00E3 nM | Selleck Chem | Tyrosine Kinase | 0.000 | -0.004 | -1.527 | -0.003 | -0.633 | 0.379 | 0.232 | 0.144 |
| Axitinib | 3.00E3 nM |  |  | 0.039 | 0.024 | -1.537 | -0.104 | -0.586 | 0.394 | 0.214 | 0.107 |
| Axitinib | 1.00E3 nM |  |  | 0.080 | 0.039 | -0.382 | 0.022 | -0.212 | 0.313 | 0.199 | 0.084 |
| Axitinib | 3.33E2 nM |  |  | 0.079 | 0.019 | -0.231 | 0.053 | -0.075 | 0.224 | 0.166 | 0.089 |
| Azithromycin | 1.00E5 nM | Axon Medchem | bacteria 50S ribosome | -0.249 | -0.033 | -1.418 | -0.295 | -0.451 | -0.410 | -0.385 | -0.542 |
| Azithromycin | 5.00E4 nM |  |  | -0.102 | 0.003 | -0.616 | -0.199 | -0.221 | -0.151 | -0.081 | -0.101 |
| Azithromycin | 2.50E4 nM |  |  | -0.009 | 0.010 | -0.110 | -0.054 | -0.073 | -0.143 | -0.015 | -0.042 |
| Azithromycin | 1.25E4 nM |  |  | -0.007 | 0.010 | -0.025 | -0.048 | -0.052 | -0.031 | -0.028 | -0.016 |
| Azithromycin | 6.25E3 nM |  |  | 0.007 | 0.019 | -0.043 | 0.021 | 0.058 | -0.037 | 0.064 | 0.006 |
| Azithromycin | 3.12E3 nM |  |  | 0.023 | 0.015 | 0.011 | 0.059 | -0.070 | -0.099 | -0.036 | -0.013 |
| Azithromycin | 1.56E3 nM |  |  | 0.029 | 0.009 | 0.028 | 0.044 | 0.045 | -0.056 | 0.000 | -0.019 |
| Azithromycin | 7.81E2 nM |  |  | 0.001 | 0.019 | -0.018 | -0.076 | -0.098 | 0.007 | -0.031 | 0.027 |
| BEZ-235 | 1.11E2 nM | Biomol | PI3K/mTOR | -0.557 | -0.216 | -1.845 | -0.543 | -1.004 | -0.659 | -0.933 | -1.103 |
| BEZ-235 | 3.70E1 nM |  |  | -0.498 | -0.175 | -1.842 | -0.551 | -1.047 | -0.615 | -0.964 | -0.912 |
| BEZ-235 | 1.23E1 nM |  |  | -0.386 | -0.131 | -1.860 | -0.551 | -0.737 | -0.502 | -0.441 | -0.552 |
| BEZ-235 | 4.10E0 nM |  |  | -0.195 | -0.059 | -1.223 | -0.436 | -0.182 | -0.235 | -0.773 | -0.244 |
| BEZ-235 | 1.40E0 nM |  |  | -0.074 | -0.005 | -0.477 | -0.315 | -0.122 | -0.044 | -0.266 | -0.068 |
| BEZ-235 | 4.60E-1 nM |  |  | -0.048 | -0.013 | -0.140 | -0.135 | -0.003 | 0.053 | -0.206 | 0.032 |
| BEZ-235 | 1.50E-1 nM |  |  | -0.028 | -0.004 | -0.016 | -0.094 | 0.022 | 0.055 | -0.106 | 0.086 |
| BEZ-235 | 5.10E-2 nM |  |  | -0.013 | -0.015 | 0.066 | -0.091 | -0.004 | 0.121 | -0.008 | 0.100 |
| BIRB-796 | 1.00E3 nM | Cayman | p38 MAPK | 0.014 | -0.077 | -0.420 | -0.725 | -0.282 | 0.261 | -0.270 | -0.085 |
| BIRB-796 | 3.33E2 nM |  |  | 0.022 | -0.050 | -0.495 | -0.740 | -0.416 | 0.250 | -0.254 | -0.077 |
| BIRB-796 | 1.11E2 nM |  |  | -0.010 | -0.037 | -0.625 | -0.655 | -0.422 | 0.144 | -0.195 | -0.094 |
| BIRB-796 | 3.70E1 nM |  |  | 0.034 | 0.012 | -0.196 | -0.692 | -0.412 | 0.105 | -0.119 | -0.041 |
| BML-111 | 1.11E3 nM | Cayman | LXA4 Receptor Agonist | -0.026 | 0.067 | -0.140 | -0.235 | -0.218 | -0.200 | -0.243 | -0.049 |
| BML-111 | 3.70E2 nM |  |  | 0.000 | -0.006 | -0.114 | -0.017 | -0.015 | -0.118 | -0.008 | -0.040 |
| BML-111 | 1.24E2 nM |  |  | -0.003 | -0.029 | 0.012 | 0.035 | 0.007 | -0.136 | -0.004 | -0.046 |
| BML-111 | 4.12E1 nM |  |  | 0.002 | -0.021 | 0.044 | -0.006 | -0.044 | -0.039 | 0.009 | -0.051 |
| BML-111 | 1.37E1 nM |  |  | 0.000 | -0.003 | 0.012 | 0.026 | -0.035 | -0.033 | -0.011 | -0.019 |
| BML-111 | 4.60E0 nM |  |  | -0.007 | 0.007 | 0.016 | 0.006 | -0.018 | 0.008 | -0.004 | 0.002 |
| BML-111 | 1.50E0 nM |  |  | 0.003 | 0.028 | -0.134 | 0.022 | 0.054 | 0.019 | -0.001 | 0.025 |
| BML-111 | 5.10E-1 nM |  |  | -0.011 | 0.025 | 0.001 | 0.058 | -0.016 | 0.029 | -0.078 | 0.018 |
| BW 245C | 1.00E4 nM | Cayman | DP Agonist | -0.047 | 0.018 | 0.048 | 0.254 | 0.009 | -0.076 | 0.457 | -0.121 |
| BW 245C | 3.33E3 nM |  |  | -0.044 | -0.022 | 0.010 | 0.194 | 0.064 | -0.102 | 0.446 | -0.100 |
| BW 245C | 1.11E3 nM |  |  | 0.017 | -0.026 | 0.056 | 0.101 | 0.014 | -0.056 | 0.169 | -0.030 |
| BW 245C | 3.70E2 nM |  |  | -0.035 | -0.009 | 0.079 | 0.152 | 0.074 | -0.028 | 0.126 | -0.004 |
| BW A868C | 1.00E4 nM | Sigma | DP Antagonist | 0.021 | 0.023 | 0.096 | 0.033 | -0.043 | 0.056 | 0.052 | 0.028 |
| BW A868C | 3.33E3 nM |  |  | -0.044 | -0.012 | -0.056 | 0.085 | 0.069 | -0.029 | 0.049 | -0.003 |
| BW A868C | 1.11E3 nM |  |  | 0.019 | -0.023 | 0.138 | 0.044 | -0.005 | 0.012 | -0.042 | 0.035 |
| BW A868C | 3.70E2 nM |  |  | -0.019 | -0.004 | 0.082 | 0.043 | 0.000 | 0.005 | -0.051 | -0.011 |
| Berberine | 1.00E4 nM | Sigma | nucleic acid-binding isoquinolone alkaloid | -0.131 | -0.019 | -1.565 | -0.153 | -0.249 | -0.208 | -0.173 | -0.286 |
| Berberine | 3.33E3 nM |  |  | -0.043 | 0.013 | -1.266 | -0.108 | -0.126 | -0.112 | -0.109 | -0.131 |
| Berberine | 1.11E3 nM |  |  | 0.002 | 0.054 | -0.961 | -0.043 | -0.082 | -0.018 | -0.079 | -0.058 |
| Berberine | 3.70E2 nM |  |  | -0.023 | 0.038 | -0.326 | -0.083 | -0.118 | 0.003 | -0.101 | 0.008 |
| CAL-101 | 3.00E3 nM | Selleck Chem | PI3Kdelta | -0.444 | -0.255 | -2.004 | -0.490 | -0.915 | -0.634 | -0.884 | -1.193 |
| CAL-101 | 1.00E3 nM |  |  | -0.328 | -0.206 | -1.694 | -0.510 | -0.693 | -0.539 | -0.551 | -0.893 |
| CAL-101 | 3.33E2 nM |  |  | -0.234 | -0.177 | -1.386 | -0.509 | -0.487 | -0.460 | -0.790 | -0.734 |
| CAL-101 | 1.11E2 nM |  |  | -0.201 | -0.148 | -1.187 | -0.477 | -0.464 | -0.396 | -0.537 | -0.559 |
| CAY10650 | 1.00E3 nM | Cayman | cPLA2α | 0.001 | -0.011 | 0.033 | -0.024 | 0.009 | -0.050 | -0.013 | -0.006 |
| CAY10650 | 3.33E2 nM |  |  | 0.015 | -0.011 | -0.060 | 0.086 | 0.032 | -0.026 | 0.003 | -0.018 |
| CAY10650 | 1.11E2 nM |  |  | 0.003 | 0.005 | 0.017 | 0.030 | 0.033 | -0.065 | -0.003 | -0.035 |
| CAY10650 | 3.70E1 nM |  |  | 0.001 | 0.001 | -0.002 | -0.112 | -0.076 | 0.072 | -0.042 | 0.003 |
| CGP 52608 | 1.00E4 nM | Sigma | RORα | -1.251 | -0.792 | -1.823 | -0.999 | -1.173 | -0.979 | -1.293 | -1.661 |
| CGP 52608 | 3.33E3 nM |  |  | -1.248 | -0.777 | -1.417 | -0.991 | -1.182 | -0.984 | -1.259 | -1.689 |
| CGP 52608 | 1.11E3 nM |  |  | -1.212 | -0.755 | -1.484 | -0.855 | -1.307 | -0.977 | -0.876 | -1.493 |
| CGP 52608 | 3.70E2 nM |  |  | -0.587 | -0.429 | -1.659 | -0.480 | -0.536 | -0.505 | -0.396 | -0.617 |
| CGP 52608 | 1.24E2 nM |  |  | -0.014 | -0.045 | -0.148 | 0.017 | 0.076 | -0.080 | 0.053 | -0.045 |
| CGP 52608 | 4.12E1 nM |  |  | -0.003 | -0.018 | 0.029 | 0.011 | 0.029 | 0.004 | 0.011 | -0.029 |
| CP-690550 | 1.00E4 nM |  | JAK | -0.103 | 0.020 | -1.963 | -0.308 | -1.004 | 0.403 | -0.332 | -0.654 |
| CP-690550 | 3.33E3 nM | Selleck Chem |  | -0.178 | -0.018 | -1.866 | -0.221 | -0.710 | 0.287 | -0.302 | -0.731 |
| CP-690550 | 1.11E3 nM |  |  | -0.174 | -0.043 | -1.859 | -0.189 | -0.700 | 0.325 | -0.331 | -0.665 |
| CP-690550 | 3.70E2 nM |  |  | -0.132 | -0.033 | -1.702 | -0.082 | -0.281 | 0.315 | -0.278 | -0.449 |
| CP-690550 | 1.24E2 nM |  |  | -0.094 | -0.018 | -1.414 | -0.110 | 0.033 | 0.198 | -0.331 | -0.458 |
| CP-690550 | 4.12E1 nM |  |  | -0.070 | -0.005 | -0.754 | -0.041 | 0.040 | 0.113 | -0.339 | -0.302 |
| CP-690550 | 1.37E1 nM |  |  | -0.071 | -0.009 | -0.749 | -0.127 | -0.097 | 0.041 | -0.130 | -0.184 |
| CP-690550 | 4.60E0 nM |  |  | 0.001 | -0.003 | -0.172 | -0.090 | -0.020 | -0.013 | -0.085 | -0.090 |
| Calcitriol | 1.00E3 nM | Sigma | Vitamin D Receptor Agonist | -0.076 | -0.012 | 0.087 | -0.394 | -0.303 | -0.287 | 0.289 | -0.404 |
| Calcitriol | 3.33E2 nM |  |  | -0.093 | 0.007 | 0.115 | -0.497 | -0.489 | -0.183 | 0.348 | -0.438 |
| Calcitriol | 1.11E2 nM |  |  | -0.165 | -0.027 | 0.147 | -0.467 | -0.472 | -0.173 | 0.358 | -0.444 |
| Calcitriol | 3.70E1 nM |  |  | -0.079 | -0.007 | 0.161 | -0.452 | -0.493 | -0.196 | 0.345 | -0.452 |
| Calcitriol | 1.23E1 nM |  |  | -0.081 | 0.001 | 0.209 | -0.399 | -0.413 | -0.099 | 0.426 | -0.392 |
| Calcitriol | 4.10E0 nM |  |  | -0.033 | -0.016 | 0.227 | -0.382 | -0.397 | -0.230 | 0.344 | -0.428 |
| Calcitriol | 1.40E0 nM |  |  | -0.084 | -0.021 | 0.298 | -0.232 | -0.228 | -0.107 | 0.401 | -0.268 |
| Calcitriol | 4.60E-1 nM |  |  | 0.012 | -0.030 | 0.184 | -0.165 | -0.122 | -0.010 | 0.277 | -0.110 |
| Calcitriol | 1.50E-1 nM |  |  | -0.027 | 0.036 | 0.048 | -0.034 | -0.024 | 0.019 | 0.081 | -0.037 |
| Calcium Chloride | 1.00E6 nM | Sigma | membrane potential modulator | 0.012 | 0.015 | 0.030 | 0.012 | 0.005 | 0.034 | 0.061 | 0.007 |
| Calcium Chloride | 1.00E5 nM |  |  | -0.004 | 0.032 | 0.049 | -0.030 | 0.024 | 0.007 | 0.043 | -0.013 |
| Calcium Chloride | 1.00E4 nM |  |  | -0.040 | -0.006 | 0.041 | -0.038 | 0.044 | 0.025 | 0.021 | 0.031 |
| Calcium Chloride | 1.00E3 nM |  |  | -0.019 | -0.021 | -0.001 | -0.037 | -0.040 | 0.044 | -0.019 | 0.069 |
| Celebrex | 5.00E4 nM | Toronto Research Chemicals | COX2 | -0.591 | -0.294 | -1.774 | -0.852 | -0.414 | -0.458 | -0.615 | -0.808 |
| Celebrex | 1.67E4 nM |  |  | -0.065 | -0.014 | -0.224 | 0.013 | -0.061 | -0.241 | -0.018 | -0.063 |
| Celebrex | 5.56E3 nM |  |  | -0.050 | -0.007 | -0.066 | 0.078 | 0.090 | -0.137 | 0.025 | -0.012 |
| Celebrex | 1.85E3 nM |  |  | -0.012 | -0.003 | -0.031 | 0.017 | 0.018 | -0.090 | 0.015 | -0.019 |
| Celebrex | 6.17E2 nM |  |  | 0.023 | -0.007 | 0.016 | -0.041 | -0.059 | 0.031 | 0.013 | -0.007 |
| Celebrex | 2.06E2 nM |  |  | 0.015 | -0.005 | 0.052 | -0.033 | -0.020 | -0.039 | -0.025 | -0.030 |
| Celebrex | 6.86E1 nM |  |  | 0.008 | -0.011 | 0.030 | 0.028 | -0.014 | -0.062 | -0.038 | -0.043 |
| Celebrex | 2.29E1 nM |  |  | -0.005 | -0.002 | -0.038 | 0.025 | 0.037 | 0.009 | -0.042 | -0.001 |
| Cerivastatin | 3.70E2 nM | Toronto Research Chemicals | HMG-CoA Reductase Inhibitor | -0.469 | -0.062 | -1.981 | -0.756 | -0.988 | 0.200 | -0.234 | -0.190 |
| Cerivastatin | 1.24E2 nM |  |  | -0.232 | -0.034 | -1.832 | -0.482 | -0.847 | 0.301 | -0.202 | -0.030 |
| Cerivastatin | 4.12E1 nM |  |  | -0.078 | -0.021 | -0.820 | -0.243 | -0.414 | 0.249 | -0.057 | 0.050 |
| Cerivastatin | 1.37E1 nM |  |  | -0.079 | -0.011 | -0.294 | -0.155 | -0.149 | 0.144 | -0.006 | 0.052 |
| Colchicine | 3.70E2 nM | EMD | Microtubule | -0.460 | -0.095 | -2.047 | -0.461 | -1.002 | 0.491 | 0.068 | -0.095 |
| Colchicine | 1.24E2 nM |  |  | -0.431 | -0.101 | -2.088 | -0.431 | -0.722 | 0.475 | 0.045 | -0.158 |
| Colchicine | 4.12E1 nM |  |  | -0.394 | -0.071 | -2.047 | -0.253 | -0.271 | 0.538 | -0.007 | -0.151 |
| Colchicine | 1.37E1 nM |  |  | -0.292 | -0.035 | -1.975 | -0.101 | 0.031 | 0.602 | -0.050 | -0.008 |
| Copper(II) Sulfate | 3.00E4 nM | Sigma | Glucose-6-phosphate Dehydrogenase Inhibitor | -0.127 | -0.074 | -1.487 | -0.447 | -0.356 | -0.304 | -0.168 | -0.337 |
| Copper(II) Sulfate | 1.00E4 nM |  |  | -0.016 | -0.034 | -0.413 | -0.135 | -0.094 | -0.176 | -0.050 | -0.185 |
| Copper(II) Sulfate | 3.33E3 nM |  |  | 0.024 | -0.023 | -0.295 | -0.171 | -0.153 | -0.060 | -0.103 | -0.071 |
| Copper(II) Sulfate | 1.11E3 nM |  |  | 0.006 | -0.025 | 0.025 | -0.040 | 0.059 | -0.003 | -0.044 | -0.006 |
| Dasatinib | 3.33E3 nM | Selleck Chem | Src Family | -0.866 | -0.244 | -2.072 | -0.605 | -1.018 | -0.785 | -0.711 | -1.671 |
| Dasatinib | 1.11E3 nM |  |  | -0.876 | -0.249 | -2.075 | -0.609 | -1.079 | -0.802 | -0.615 | -1.658 |
| Dasatinib | 3.70E2 nM |  |  | -0.868 | -0.240 | -1.832 | -0.618 | -0.997 | -0.808 | -0.525 | -1.649 |
| Dasatinib | 1.24E2 nM |  |  | -0.754 | -0.138 | -1.840 | -0.632 | -1.153 | -0.807 | -0.479 | -1.592 |
| Dasatinib | 4.12E1 nM |  |  | -0.485 | -0.055 | -1.895 | -0.631 | -1.226 | -0.796 | -0.308 | -1.445 |
| Dasatinib | 1.37E1 nM |  |  | -0.466 | -0.062 | -1.916 | -0.488 | -0.892 | -0.758 | -0.173 | -1.244 |
| Dasatinib | 4.60E0 nM |  |  | -0.239 | -0.027 | -1.258 | -0.088 | -0.512 | -0.141 | 0.276 | -0.288 |
| Desloratadine | 3.00E4 nM | EMD | H1 Antagonist | -0.598 | -0.259 | -1.699 | -0.567 | -1.042 | -0.902 | -0.924 | -1.167 |
| Desloratadine | 1.00E4 nM |  |  | -0.170 | -0.069 | -0.469 | -0.367 | -0.341 | -0.274 | -0.335 | -0.338 |
| Desloratadine | 3.33E3 nM |  |  | -0.017 | -0.026 | -0.142 | -0.141 | -0.079 | -0.061 | -0.076 | -0.062 |
| Desloratadine | 1.11E3 nM |  |  | -0.002 | -0.009 | -0.086 | -0.071 | -0.135 | 0.010 | -0.015 | -0.032 |
| Dexamethasone | 1.11E3 nM | Prestwick | GR Agonist | -0.115 | -0.108 | 0.163 | -0.608 | -0.611 | -0.296 | -0.355 | -0.321 |
| Dexamethasone | 3.70E2 nM |  |  | -0.114 | -0.121 | 0.175 | -0.601 | -0.679 | -0.338 | -0.324 | -0.378 |
| Dexamethasone | 1.24E2 nM |  |  | -0.124 | -0.116 | 0.182 | -0.485 | -0.344 | -0.304 | -0.326 | -0.380 |
| Dexamethasone | 4.12E1 nM |  |  | -0.210 | -0.108 | 0.227 | -0.613 | -0.446 | -0.405 | -0.383 | -0.479 |
| Digoxigenin | 1.11E3 nM | Sigma | Na,K-ATPase | -1.096 | -0.493 | -1.625 | -0.770 | -1.328 | -1.051 | -0.460 | -1.230 |
| Digoxigenin | 3.70E2 nM |  |  | -0.791 | -0.329 | -1.713 | -0.750 | -1.376 | -0.655 | 0.123 | -0.807 |
| Digoxigenin | 1.24E2 nM |  |  | -0.335 | -0.074 | -1.699 | -0.413 | -0.565 | 0.013 | 0.470 | -0.384 |
| Digoxigenin | 4.12E1 nM |  |  | -0.147 | 0.026 | -1.557 | 0.068 | -0.006 | 0.064 | 0.063 | 0.000 |
| Digoxigenin | 1.37E1 nM |  |  | 0.009 | -0.014 | -0.060 | 0.119 | 0.073 | 0.051 | 0.037 | 0.021 |
| Digoxigenin | 4.60E0 nM |  |  | 0.017 | -0.016 | 0.003 | 0.048 | -0.001 | 0.032 | 0.077 | 0.010 |
| Digoxigenin | 1.50E0 nM |  |  | -0.023 | -0.024 | -0.096 | 0.002 | 0.042 | 0.054 | 0.035 | 0.014 |
| Digoxigenin | 5.10E-1 nM |  |  | 0.009 | 0.016 | -0.036 | 0.046 | 0.071 | 0.103 | 0.019 | 0.053 |
| Digoxin | 1.00E4 nM | Sigma | Na,K-ATPase | -1.244 | -0.571 | -1.757 | -1.011 | -1.109 | -0.972 | -0.827 | -1.516 |
| Digoxin | 3.33E3 nM |  |  | -1.228 | -0.552 | -1.756 | -1.018 | -1.189 | -0.984 | -0.815 | -1.434 |
| Digoxin | 1.11E3 nM |  |  | -1.155 | -0.518 | -1.769 | -0.983 | -1.193 | -0.963 | -0.572 | -1.259 |
| Digoxin | 3.70E2 nM |  |  | -0.842 | -0.335 | -1.811 | -0.947 | -1.178 | -0.549 | 0.065 | -0.762 |
| Dimethyl Fumarate | 5.00E4 nM | Sigma | NF-kappaB Inhibitor | -0.275 | -0.141 | -1.078 | -0.245 | -0.835 | -0.389 | -0.498 | -0.520 |
| Dimethyl Fumarate | 1.67E4 nM |  |  | -0.037 | -0.036 | -0.019 | -0.075 | -0.274 | -0.068 | -0.079 | -0.107 |
| Dimethyl Fumarate | 5.56E3 nM |  |  | -0.048 | -0.014 | 0.050 | -0.072 | -0.115 | -0.048 | 0.019 | -0.013 |
| Dimethyl Fumarate | 1.85E3 nM |  |  | -0.016 | -0.001 | -0.003 | -0.066 | -0.003 | -0.007 | -0.009 | 0.022 |
| EGF | 4.00E0 nM | R&D systems | EGFR | 0.005 | -0.023 | -0.012 | -0.033 | -0.011 | 0.010 | -0.005 | -0.009 |
| EGF | 1.30E0 nM |  |  | -0.010 | 0.002 | -0.033 | -0.066 | -0.024 | -0.004 | 0.058 | 0.048 |
| EGF | 4.40E-1 nM |  |  | 0.027 | 0.018 | -0.092 | 0.041 | 0.024 | 0.035 | 0.051 | 0.043 |
| EGF | 1.50E-1 nM |  |  | -0.003 | -0.024 | -0.101 | -0.081 | 0.024 | 0.062 | -0.017 | 0.035 |
| Enbrel | 1.00E4 ng/ml | Amgen | TNF-alpha Antagonist | -0.022 | -0.042 | 0.064 | -0.137 | -0.139 | -0.085 | 0.078 | -0.928 |
| Enbrel | 1.00E3 ng/ml |  |  | -0.012 | -0.048 | 0.022 | -0.078 | 0.031 | -0.005 | 0.152 | -0.743 |
| Enbrel | 1.00E2 ng/ml |  |  | 0.024 | -0.022 | 0.028 | 0.029 | 0.095 | 0.025 | 0.179 | -0.539 |
| Enbrel | 1.00E1 ng/ml |  |  | -0.007 | -0.010 | 0.032 | 0.030 | 0.053 | 0.040 | 0.062 | 0.020 |
| Epothilone B | 3.33E2 nM | Toronto Research Chemicals | Microtubule | -0.210 | 0.004 | -1.469 | -0.178 | -0.295 | -0.027 | -0.122 | -0.123 |
| Epothilone B | 1.11E2 nM |  |  | -0.222 | -0.003 | -1.451 | -0.139 | -0.263 | -0.049 | -0.079 | -0.126 |
| Epothilone B | 3.70E1 nM |  |  | -0.227 | -0.009 | -1.626 | -0.158 | -0.252 | 0.059 | -0.047 | -0.073 |
| Epothilone B | 1.23E1 nM |  |  | -0.204 | -0.016 | -1.610 | -0.164 | -0.217 | 0.043 | -0.011 | -0.073 |
| Epothilone B | 4.10E0 nM |  |  | -0.162 | 0.002 | -1.508 | -0.152 | -0.215 | 0.050 | -0.058 | -0.053 |
| Epothilone B | 1.40E0 nM |  |  | -0.041 | 0.004 | -0.604 | -0.045 | -0.095 | 0.064 | 0.016 | -0.015 |
| Epothilone B | 4.60E-1 nM |  |  | 0.002 | 0.014 | -0.316 | -0.088 | -0.056 | -0.022 | -0.053 | -0.029 |
| Epothilone B | 1.50E-1 nM |  |  | -0.025 | -0.002 | -0.019 | -0.066 | 0.029 | -0.050 | -0.046 | 0.017 |
| Erlotinib | 1.00E4 nM | Selleck Chem | EGFR | -0.089 | -0.010 | -0.187 | -0.286 | -0.190 | -0.012 | 0.156 | 0.019 |
| Erlotinib | 3.33E3 nM |  |  | -0.127 | -0.020 | -0.033 | -0.140 | -0.036 | -0.070 | 0.054 | -0.035 |
| Erlotinib | 1.11E3 nM |  |  | -0.066 | -0.023 | 0.047 | -0.024 | -0.003 | -0.085 | 0.050 | -0.010 |
| Erlotinib | 3.70E2 nM |  |  | -0.051 | -0.008 | 0.032 | -0.014 | 0.082 | -0.032 | 0.058 | -0.004 |
| Erythromycin | 9.00E4 nM | Sigma | bacteria 50S ribosome | -0.059 | -0.011 | -0.017 | -0.234 | -0.287 | -0.070 | 0.034 | -0.032 |
| Erythromycin | 3.00E4 nM |  |  | -0.054 | 0.003 | 0.017 | -0.204 | -0.215 | -0.007 | 0.006 | 0.006 |
| Erythromycin | 1.00E4 nM |  |  | -0.033 | -0.001 | -0.008 | -0.016 | 0.029 | -0.041 | 0.063 | -0.017 |
| Erythromycin | 3.33E3 nM |  |  | -0.001 | 0.006 | -0.017 | 0.014 | -0.100 | -0.044 | -0.073 | 0.029 |
| Ethacrynic Acid | 1.00E4 nM | ENZO | Glutathione S-Transferase Inhibitor | -0.185 | -0.085 | -1.063 | -0.316 | -0.318 | -0.352 | -0.244 | -0.331 |
| Ethacrynic Acid | 3.33E3 nM |  |  | -0.012 | -0.010 | -0.167 | -0.054 | -0.113 | -0.078 | 0.079 | -0.059 |
| Ethacrynic Acid | 1.11E3 nM |  |  | -0.009 | -0.003 | -0.109 | -0.058 | -0.114 | -0.037 | -0.123 | -0.057 |
| Ethacrynic Acid | 3.70E2 nM |  |  | -0.004 | 0.005 | -0.042 | -0.039 | -0.062 | -0.010 | 0.004 | -0.009 |
| Everolimus | 1.11E2 nM | AG Scientific | mTOR | -0.339 | -0.115 | -1.677 | -0.344 | -0.400 | -0.472 | -0.542 | -0.386 |
| Everolimus | 3.70E1 nM |  |  | -0.038 | -0.032 | 0.028 | 0.026 | 0.015 | -0.014 | -0.103 | -0.028 |
| Everolimus | 1.23E1 nM |  |  | -0.033 | -0.001 | 0.030 | -0.032 | 0.027 | -0.046 | -0.087 | -0.023 |
| Everolimus | 4.10E0 nM |  |  | -0.143 | -0.042 | -0.706 | -0.470 | -0.100 | -0.108 | -0.103 | -0.076 |
| FICZ | 1.00E3 nM | Toronto Research Chemicals | AHR | -0.051 | -0.024 | 0.160 | -0.074 | 0.012 | -0.151 | -0.310 | -0.002 |
| FICZ | 3.33E2 nM |  |  | -0.048 | 0.004 | 0.102 | -0.052 | -0.055 | -0.181 | -0.262 | -0.034 |
| FICZ | 1.11E2 nM |  |  | -0.045 | -0.012 | 0.047 | -0.154 | 0.037 | -0.170 | -0.260 | -0.061 |
| FICZ | 3.70E1 nM |  |  | -0.034 | -0.026 | 0.142 | -0.088 | -0.069 | -0.090 | -0.216 | -0.022 |
| FK-506 | 1.11E3 nM | Selleck Chem | Calcineurin Inhibitor | -0.427 | -0.166 | -1.714 | -0.587 | -1.110 | -0.877 | -0.682 | -1.285 |
| FK-506 | 3.70E2 nM |  |  | -0.385 | -0.151 | -1.801 | -0.532 | -1.138 | -0.873 | -0.420 | -1.107 |
| FK-506 | 1.24E2 nM |  |  | -0.406 | -0.168 | -1.600 | -0.580 | -1.237 | -0.895 | -0.630 | -1.183 |
| FK-506 | 4.12E1 nM |  |  | -0.356 | -0.129 | -1.706 | -0.565 | -1.223 | -0.856 | -0.748 | -1.216 |
| FK-506 | 1.37E1 nM |  |  | -0.343 | -0.138 | -1.714 | -0.545 | -1.143 | -0.831 | -0.668 | -1.111 |
| FK-506 | 4.60E0 nM |  |  | -0.341 | -0.137 | -1.703 | -0.557 | -1.230 | -0.822 | -0.694 | -1.037 |
| FSL-1 | 1.00E2 ng/ml | Invivogen | TLR2/6 | 0.018 | 0.031 | 0.002 | 0.129 | 0.204 | -0.117 | 1.004 | -0.121 |
| FSL-1 | 1.00E1 ng/ml |  |  | 0.013 | 0.025 | 0.030 | 0.178 | 0.158 | -0.112 | 0.863 | -0.216 |
| FSL-1 | 1.00E0 ng/ml |  |  | 0.022 | 0.038 | -0.007 | 0.035 | 0.083 | -0.001 | 0.427 | -0.035 |
| FSL-1 | 1.00E-1 ng/ml |  |  | -0.003 | -0.007 | -0.035 | -0.018 | 0.020 | 0.153 | 0.060 | 0.114 |
| Flagellin | 1.00E2 ng/ml | Invivogen | TLR5 | -0.101 | -0.049 | 0.160 | 0.309 | 0.236 | -0.326 | 1.087 | 0.099 |
| Flagellin | 1.00E1 ng/ml |  |  | 0.019 | 0.023 | 0.070 | 0.081 | 0.197 | -0.085 | 0.940 | -0.133 |
| Flagellin | 1.00E0 ng/ml |  |  | 0.020 | 0.008 | 0.070 | -0.005 | 0.024 | -0.065 | -0.001 | -0.051 |
| Flagellin | 1.00E-1 ng/ml |  |  | -0.008 | 0.000 | -0.052 | -0.049 | 0.043 | 0.053 | 0.071 | 0.073 |
| Fluticasone Propionate | 1.00E4 nM | American custom chemicals corporation | GR Agonist | -0.548 | -0.251 | -1.834 | -0.691 | -1.034 | -0.693 | -0.589 | -0.833 |
| Fluticasone Propionate | 3.33E3 nM |  |  | -0.437 | -0.245 | -1.611 | -0.709 | -0.970 | -0.683 | -0.777 | -0.892 |
| Fluticasone Propionate | 1.11E3 nM |  |  | -0.327 | -0.230 | -0.979 | -0.668 | -0.846 | -0.557 | -0.644 | -0.733 |
| Fluticasone Propionate | 3.70E2 nM |  |  | -0.078 | -0.105 | 0.057 | -0.189 | -0.181 | -0.229 | -0.205 | -0.220 |
| Fostamatinib Disodium | 1.00E4 nM | Selleck Chem | Syk | -0.922 | -0.371 | -1.904 | -0.787 | -1.025 | -1.040 | -1.439 | -1.737 |
| Fostamatinib Disodium | 3.33E3 nM |  |  | -0.901 | -0.362 | -1.927 | -0.828 | -1.238 | -0.961 | -1.481 | -1.778 |
| Fostamatinib Disodium | 1.11E3 nM |  |  | -0.638 | -0.274 | -1.952 | -0.709 | -0.965 | -0.762 | -1.417 | -1.551 |
| Fostamatinib Disodium | 3.70E2 nM |  |  | -0.296 | -0.108 | -0.544 | -0.370 | -0.599 | -0.117 | -0.488 | -0.409 |
| GDC-0941 | 3.00E4 nM | Selleck Chem | PI3K | -0.894 | -0.359 | -2.012 | -0.699 | -1.089 | -0.975 | -0.977 | -1.516 |
| GDC-0941 | 1.00E4 nM |  |  | -0.891 | -0.356 | -1.991 | -0.701 | -1.264 | -0.990 | -0.750 | -1.619 |
| GDC-0941 | 3.33E3 nM |  |  | -0.848 | -0.327 | -2.022 | -0.694 | -1.314 | -0.964 | -0.994 | -1.633 |
| GDC-0941 | 1.11E3 nM |  |  | -0.805 | -0.310 | -1.958 | -0.679 | -1.257 | -0.933 | -0.964 | -1.625 |
| GDC-0941 | 3.70E2 nM |  |  | -0.729 | -0.285 | -1.466 | -0.651 | -0.920 | -0.889 | -0.009 | -1.288 |
| GDC-0941 | 1.24E2 nM |  |  | -0.537 | -0.205 | -1.689 | -0.621 | -0.880 | -0.882 | -0.950 | -1.410 |
| GDC-0941 | 4.12E1 nM |  |  | -0.427 | -0.188 | -1.746 | -0.500 | -0.461 | -0.698 | -0.472 | -0.966 |
| GDC-0941 | 1.37E1 nM |  |  | -0.294 | -0.152 | -1.601 | -0.426 | 0.028 | -0.484 | -0.441 | -0.608 |
| GF 109203X | 1.00E4 nM | Biomol | PKC (c+n) | -0.524 | -0.220 | -1.670 | -0.363 | -0.485 | -0.858 | -0.903 | -1.569 |
| GF 109203X | 3.33E3 nM |  |  | -0.369 | -0.130 | -0.991 | -0.278 | -0.459 | -0.845 | -0.395 | -1.351 |
| GF 109203X | 1.11E3 nM |  |  | -0.258 | -0.084 | -0.561 | -0.292 | -0.450 | -0.847 | -0.105 | -1.001 |
| GF 109203X | 3.70E2 nM |  |  | -0.123 | -0.036 | -0.209 | -0.220 | -0.329 | -0.647 | -0.196 | -0.250 |
| GW3965 | 1.00E4 nM | Sigma | LXRalpha Agonist | -0.031 | -0.023 | -0.969 | -0.237 | -0.120 | 0.135 | -0.050 | 0.005 |
| GW3965 | 3.33E3 nM |  |  | 0.024 | -0.016 | -0.484 | -0.082 | 0.016 | 0.266 | -0.037 | 0.071 |
| GW3965 | 1.11E3 nM |  |  | -0.004 | -0.023 | -0.362 | -0.047 | -0.041 | 0.245 | -0.025 | 0.030 |
| GW3965 | 3.70E2 nM |  |  | 0.005 | -0.003 | -0.186 | -0.029 | -0.026 | 0.234 | -0.013 | 0.035 |
| GW3965 | 1.24E2 nM |  |  | 0.026 | -0.015 | -0.271 | -0.078 | -0.037 | 0.169 | -0.010 | 0.008 |
| GW3965 | 4.12E1 nM |  |  | 0.011 | 0.020 | -0.261 | -0.028 | 0.023 | 0.154 | -0.041 | 0.020 |
| Gleevec | 1.00E4 nM | Toronto Research Chemicals | BCR-ABL kinase inhibitor | -0.017 | -0.028 | -0.201 | -0.232 | -0.444 | 0.103 | 0.027 | 0.053 |
| Gleevec | 3.33E3 nM |  |  | 0.028 | -0.011 | -0.052 | 0.041 | -0.058 | 0.026 | 0.074 | 0.027 |
| Gleevec | 1.11E3 nM |  |  | 0.027 | -0.012 | 0.016 | 0.029 | -0.023 | 0.034 | -0.066 | 0.015 |
| Gleevec | 3.70E2 nM |  |  | 0.018 | 0.002 | 0.038 | -0.037 | 0.021 | 0.016 | 0.078 | 0.016 |
| HKLM | 1.00E7 mU/ml | Invivogen | TLR2 | -0.013 | 0.027 | 0.205 | 0.122 | 0.221 | -0.237 | 0.989 | 0.103 |
| HKLM | 1.00E6 mU/ml |  |  | 0.020 | 0.026 | 0.137 | 0.062 | 0.222 | -0.158 | 0.897 | 0.140 |
| HKLM | 1.00E5 mU/ml |  |  | 0.018 | 0.013 | 0.059 | 0.140 | 0.215 | -0.037 | 0.276 | 0.049 |
| HKLM | 1.00E4 mU/ml |  |  | 0.013 | 0.009 | 0.078 | -0.013 | 0.063 | 0.029 | 0.088 | -0.030 |
| Hydrocortisone | 9.00E4 nM | Sigma | GR Agonist | -0.141 | -0.160 | -0.047 | -0.429 | -0.515 | -0.251 | -0.297 | -0.249 |
| Hydrocortisone | 3.00E4 nM |  |  | -0.064 | -0.139 | 0.108 | -0.415 | -0.548 | -0.186 | -0.220 | -0.132 |
| Hydrocortisone | 1.00E4 nM |  |  | -0.048 | -0.112 | 0.161 | -0.409 | -0.291 | -0.272 | -0.231 | -0.158 |
| Hydrocortisone | 3.33E3 nM |  |  | -0.042 | -0.095 | 0.121 | -0.289 | -0.354 | -0.234 | -0.158 | -0.115 |
| Hydroxychloroquine Sulfate | 1.00E4 nM | Sigma | H1 Antagonist | -0.140 | -0.009 | -0.101 | -0.153 | -0.265 | -0.020 | -0.151 | -0.076 |
| Hydroxychloroquine Sulfate | 3.33E3 nM |  |  | -0.011 | 0.007 | 0.052 | -0.133 | -0.096 | -0.052 | -0.055 | -0.052 |
| Hydroxychloroquine Sulfate | 1.11E3 nM |  |  | 0.002 | -0.017 | -0.019 | -0.122 | -0.040 | 0.007 | -0.031 | -0.015 |
| Hydroxychloroquine Sulfate | 3.70E2 nM |  |  | 0.005 | 0.006 | -0.062 | 0.036 | -0.022 | 0.010 | 0.009 | 0.006 |
| Hydroxychloroquine Sulfate | 1.24E2 nM |  |  | 0.014 | -0.004 | -0.016 | -0.049 | -0.075 | -0.032 | -0.032 | -0.009 |
| Hydroxychloroquine Sulfate | 4.12E1 nM |  |  | 0.012 | 0.001 | 0.027 | -0.051 | 0.073 | -0.082 | -0.046 | -0.023 |
| Hydroxychloroquine Sulfate | 1.37E1 nM |  |  | -0.001 | 0.001 | 0.023 | 0.016 | 0.082 | 0.014 | -0.016 | -0.002 |
| Hydroxychloroquine Sulfate | 4.60E0 nM |  |  | 0.015 | 0.013 | 0.070 | -0.065 | 0.005 | 0.070 | -0.002 | 0.032 |
| IC-87114 | 3.33E3 nM | Selleck Chem | PI3Kdelta | -0.319 | -0.108 | -0.494 | -0.629 | -0.511 | -0.158 | -0.458 | -0.277 |
| IC-87114 | 1.11E3 nM |  |  | -0.244 | -0.083 | -0.368 | -0.567 | -0.732 | -0.150 | -0.044 | -0.244 |
| IC-87114 | 3.70E2 nM |  |  | -0.208 | -0.074 | -0.163 | -0.529 | -0.491 | -0.076 | -0.205 | -0.183 |
| IC-87114 | 1.24E2 nM |  |  | -0.088 | -0.064 | 0.001 | -0.471 | -0.364 | -0.104 | -0.346 | -0.182 |
| ICI 63197 | 9.00E4 nM | Tocris Cookson | PDE IV Inhibitor | -0.075 | -0.068 | -0.005 | 0.011 | -0.056 | 0.098 | 0.288 | -0.045 |
| ICI 63197 | 3.00E4 nM |  |  | -0.017 | -0.040 | -0.007 | 0.069 | -0.101 | -0.012 | 0.121 | -0.021 |
| ICI 63197 | 1.00E4 nM |  |  | 0.019 | -0.031 | 0.033 | 0.038 | -0.037 | -0.007 | 0.051 | -0.003 |
| ICI 63197 | 3.33E3 nM |  |  | 0.010 | -0.028 | 0.085 | 0.028 | 0.065 | -0.031 | 0.068 | 0.006 |
| IL-12 | 1.00E0 nM | R&D systems | IL-12R ligand | 0.040 | 0.021 | 0.126 | -0.104 | 0.046 | 0.106 | 0.133 | 0.028 |
| IL-12 | 3.30E-1 nM |  |  | 0.035 | 0.001 | 0.119 | -0.131 | 0.043 | 0.071 | 0.084 | 0.045 |
| IL-12 | 1.10E-1 nM |  |  | -0.009 | 0.018 | 0.114 | -0.113 | 0.081 | 0.072 | 0.091 | 0.029 |
| IL-12 | 3.70E-2 nM |  |  | 0.044 | 0.001 | 0.127 | -0.068 | 0.121 | 0.049 | 0.074 | 0.039 |
| IL-13 | 1.00E0 nM | R&D systems | IL-13R ligand | -0.044 | 0.002 | -0.252 | -0.201 | -0.125 | 0.179 | 0.049 | 0.067 |
| IL-13 | 3.30E-1 nM |  |  | -0.051 | -0.007 | -0.107 | -0.077 | -0.043 | 0.156 | 0.048 | 0.082 |
| IL-13 | 1.10E-1 nM |  |  | -0.003 | -0.023 | -0.068 | -0.089 | 0.030 | 0.077 | 0.029 | 0.069 |
| IL-13 | 3.70E-2 nM |  |  | -0.011 | -0.013 | -0.143 | -0.057 | 0.039 | 0.101 | 0.031 | 0.071 |
| IL-17A | 1.00E1 nM | R&D systems | IL-17R ligand | 0.023 | 0.016 | -0.062 | 0.895 | -0.091 | 0.025 | 0.108 | 0.063 |
| IL-17A | 3.30E0 nM |  |  | 0.025 | -0.015 | 0.037 | 0.891 | -0.058 | 0.012 | 0.131 | 0.035 |
| IL-17A | 1.10E0 nM |  |  | -0.001 | -0.002 | 0.124 | 0.890 | 0.011 | 0.026 | 0.149 | 0.008 |
| IL-17A | 3.70E-1 nM |  |  | -0.005 | -0.014 | -0.032 | 0.887 | -0.025 | 0.039 | 0.103 | 0.008 |
| IL-17F | 3.00E0 nM | R&D systems | IL-17R ligand | -0.005 | -0.016 | -0.016 | -0.028 | 0.852 | 0.081 | 0.013 | 0.027 |
| IL-17F | 1.00E0 nM |  |  | 0.021 | 0.021 | 0.060 | 0.026 | 0.847 | 0.009 | 0.086 | -0.013 |
| IL-17F | 3.30E-1 nM |  |  | 0.008 | -0.013 | 0.082 | 0.033 | 0.821 | 0.011 | 0.104 | 0.039 |
| IL-17F | 1.10E-1 nM |  |  | -0.015 | 0.002 | 0.086 | 0.084 | 0.777 | -0.011 | 0.082 | -0.017 |
| IL-2 | 3.00E0 nM | R&D systems | IL-2R ligand | -0.031 | 0.010 | -0.176 | 0.072 | 0.065 | 0.673 | 0.112 | 0.031 |
| IL-2 | 1.00E0 nM |  |  | -0.007 | 0.016 | -0.041 | 0.132 | 0.203 | 0.642 | 0.159 | 0.003 |
| IL-2 | 3.30E-1 nM |  |  | 0.033 | 0.007 | 0.053 | 0.220 | -0.033 | 0.568 | 0.146 | 0.016 |
| IL-2 | 1.10E-1 nM |  |  | 0.001 | 0.011 | 0.135 | 0.210 | 0.157 | 0.434 | 0.203 | 0.013 |
| IL-35 | 1.00E0 nM | Sino Biologics | IL-35R ligand | -0.074 | -0.020 | -0.244 | -0.183 | -0.089 | 0.082 | -0.131 | -0.030 |
| IL-35 | 3.30E-1 nM |  |  | 0.005 | -0.007 | -0.028 | -0.047 | 0.032 | 0.061 | 0.009 | 0.029 |
| IL-35 | 1.10E-1 nM |  |  | -0.028 | -0.010 | -0.020 | 0.003 | -0.018 | 0.030 | 0.009 | 0.029 |
| IL-35 | 3.70E-2 nM |  |  | -0.026 | -0.015 | 0.022 | 0.042 | 0.070 | 0.052 | 0.038 | 0.004 |
| IL-6 R alpha | 3.00E0 nM | R&D system | IL-6 | 0.000 | 0.020 | 0.110 | 0.208 | 0.181 | -0.032 | 0.450 | -0.095 |
| IL-6 R alpha | 1.00E0 nM |  |  | -0.012 | 0.033 | 0.005 | 0.120 | 0.049 | -0.016 | 0.123 | -0.012 |
| IL-6 R alpha | 3.30E-1 nM |  |  | 0.000 | 0.012 | -0.045 | 0.041 | -0.049 | 0.018 | 0.049 | -0.016 |
| IL-6 R alpha | 1.10E-1 nM |  |  | -0.003 | 0.010 | 0.010 | -0.026 | -0.027 | 0.073 | -0.004 | 0.048 |
| IL-6 | 3.00E0 nM | R&D system | IL-6R ligand | -0.009 | 0.000 | 0.039 | 0.129 | 0.076 | -0.021 | 0.687 | -0.012 |
| IL-6 | 1.00E0 nM |  |  | 0.026 | -0.015 | 0.052 | 0.095 | -0.038 | -0.073 | 0.681 | -0.004 |
| IL-6 | 3.30E-1 nM |  |  | -0.015 | 0.006 | 0.022 | 0.060 | -0.059 | -0.070 | 0.631 | 0.008 |
| IL-6 | 1.10E-1 nM |  |  | -0.008 | -0.020 | -0.036 | 0.083 | -0.009 | -0.028 | 0.129 | -0.024 |
| INCB-018424 | 3.70E2 nM | Active Biochem | JAK | -0.227 | -0.033 | -1.481 | -0.268 | -0.745 | -0.097 | -0.638 | -0.932 |
| INCB-018424 | 1.24E2 nM |  |  | -0.133 | -0.094 | -1.444 | -0.177 | -0.701 | 0.048 | -0.715 | -0.809 |
| INCB-018424 | 4.12E1 nM |  |  | -0.091 | -0.058 | -1.323 | 0.062 | -0.465 | 0.135 | -0.544 | -0.518 |
| INCB-018424 | 1.37E1 nM |  |  | -0.081 | -0.040 | -0.583 | 0.055 | -0.195 | 0.128 | -0.535 | -0.293 |
| Ibudilast | 9.00E4 nM | Sigma | PDE IV Inhibitor | -0.118 | -0.077 | -0.880 | -0.010 | -0.309 | 0.087 | 0.117 | -0.106 |
| Ibudilast | 3.00E4 nM |  |  | -0.062 | -0.052 | -0.354 | -0.009 | -0.099 | 0.086 | 0.088 | -0.050 |
| Ibudilast | 1.00E4 nM |  |  | -0.018 | -0.039 | -0.065 | -0.007 | -0.088 | 0.055 | 0.081 | -0.025 |
| Ibudilast | 3.33E3 nM |  |  | -0.001 | -0.025 | 0.037 | 0.006 | -0.027 | 0.033 | 0.037 | -0.008 |
| Iloprost | 3.00E4 nM | Invivogen | EP1 Agonist | -0.058 | -0.098 | 0.033 | 0.291 | 0.062 | 0.131 | 0.577 | -0.202 |
| Iloprost | 1.00E4 nM |  |  | -0.070 | -0.001 | 0.095 | 0.437 | 0.063 | 0.064 | 0.627 | -0.114 |
| Iloprost | 3.33E3 nM |  |  | -0.091 | -0.014 | 0.023 | 0.371 | 0.099 | 0.048 | 0.624 | -0.176 |
| Iloprost | 1.11E3 nM |  |  | -0.074 | -0.068 | 0.027 | 0.289 | 0.055 | -0.021 | 0.613 | -0.122 |
| Imiquimod | 1.00E2 ng/ml | EMD | TLR7 | 0.008 | 0.013 | 0.010 | 0.031 | 0.032 | -0.015 | 0.089 | -0.066 |
| Imiquimod | 1.00E1 ng/ml |  |  | 0.015 | 0.000 | -0.025 | 0.114 | 0.024 | 0.056 | 0.117 | 0.046 |
| Imiquimod | 1.00E0 ng/ml |  |  | 0.012 | 0.004 | -0.122 | -0.035 | -0.005 | 0.166 | 0.139 | 0.164 |
| Imiquimod | 1.00E-1 ng/ml |  |  | -0.009 | -0.006 | -0.212 | -0.066 | -0.007 | 0.144 | 0.051 | 0.139 |
| Indomethacin | 1.00E5 nM | EMD | COX1/2 | -0.024 | 0.054 | -1.139 | -0.317 | -0.404 | -0.132 | -0.098 | -0.109 |
| Indomethacin | 3.33E4 nM |  |  | -0.018 | 0.042 | -0.453 | -0.206 | -0.160 | -0.102 | -0.062 | -0.052 |
| Indomethacin | 1.11E4 nM |  |  | -0.042 | 0.003 | -0.132 | 0.027 | -0.018 | -0.069 | -0.055 | -0.028 |
| Indomethacin | 3.70E3 nM |  |  | 0.023 | -0.003 | -0.063 | -0.007 | -0.009 | -0.045 | -0.023 | -0.037 |
| JNK Inhibitor VIII | 3.00E4 nM | Selleck Chem | JNK | -0.069 | -0.001 | -0.305 | -0.118 | -0.182 | -0.268 | -0.098 | -0.105 |
| JNK Inhibitor VIII | 1.00E4 nM |  |  | -0.052 | -0.007 | -0.236 | -0.178 | -0.235 | -0.228 | -0.202 | -0.077 |
| JNK Inhibitor VIII | 3.33E3 nM |  |  | -0.006 | 0.001 | -0.148 | -0.108 | -0.042 | -0.193 | -0.050 | -0.064 |
| JNK Inhibitor VIII | 1.11E3 nM |  |  | -0.002 | 0.001 | 0.016 | -0.116 | -0.080 | -0.041 | -0.077 | 0.002 |
| LBH-589 | 3.00E4 nM | Selleck Chem | HDAC | -0.028 | -0.006 | -0.018 | -0.053 | 0.019 | 0.084 | 0.030 | 0.055 |
| LBH-589 | 1.00E4 nM |  |  | -0.014 | 0.029 | 0.083 | 0.005 | 0.062 | 0.017 | 0.098 | 0.013 |
| LBH-589 | 3.33E3 nM |  |  | 0.000 | -0.016 | 0.034 | 0.095 | -0.043 | -0.008 | 0.095 | 0.029 |
| LBH-589 | 1.11E3 nM |  |  | 0.029 | -0.007 | 0.012 | 0.021 | 0.020 | 0.009 | 0.093 | 0.029 |
| LBH-589 | 3.70E2 nM |  |  | -0.009 | -0.010 | 0.143 | -0.035 | 0.002 | 0.005 | 0.112 | 0.024 |
| LBH-589 | 1.24E2 nM |  |  | 0.016 | 0.024 | 0.027 | 0.124 | 0.176 | -0.011 | 0.130 | 0.034 |
| LBH-589 | 4.12E1 nM |  |  | 0.013 | 0.037 | -0.112 | -0.029 | 0.007 | 0.018 | -0.004 | 0.029 |
| LBH-589 | 1.37E1 nM |  |  | -0.041 | 0.010 | -0.079 | -0.084 | -0.044 | 0.082 | -0.008 | 0.066 |
| LPS | 1.00E2 ng/ml | Invivogen | TLR4 | -0.127 | -0.066 | 0.238 | 0.282 | 0.248 | -0.057 | 1.135 | 0.392 |
| LPS | 1.00E1 ng/ml |  |  | -0.126 | -0.056 | 0.160 | 0.328 | 0.265 | -0.046 | 0.717 | 0.093 |
| LPS | 1.00E0 ng/ml |  |  | -0.130 | -0.057 | 0.233 | 0.141 | 0.208 | -0.136 | 1.129 | 0.217 |
| LPS | 1.00E-1 ng/ml |  |  | -0.081 | -0.014 | 0.211 | 0.339 | 0.289 | -0.079 | 1.135 | 0.340 |
| LY-450139 | 1.00E4 nM | Selleck Chem | γ-secretase | -0.024 | -0.011 | -0.153 | -0.375 | -0.204 | -0.164 | -0.063 | -0.029 |
| LY-450139 | 3.33E3 nM |  |  | 0.012 | -0.014 | -0.024 | -0.351 | -0.085 | -0.125 | -0.068 | 0.011 |
| LY-450139 | 1.11E3 nM |  |  | -0.001 | -0.007 | -0.037 | -0.104 | -0.102 | -0.045 | 0.122 | -0.041 |
| LY-450139 | 3.70E2 nM |  |  | -0.003 | 0.002 | 0.028 | -0.195 | -0.236 | 0.074 | 0.033 | 0.014 |
| LY2157299 | 1.00E4 nM | Selleck Chem | TGFβRI | 0.000 | -0.013 | -0.111 | -0.008 | -0.033 | 0.081 | 0.006 | 0.048 |
| LY2157299 | 3.33E3 nM |  |  | 0.019 | 0.023 | -0.043 | 0.078 | 0.159 | 0.009 | 0.100 | 0.037 |
| LY2157299 | 1.11E3 nM |  |  | 0.021 | -0.016 | -0.085 | 0.029 | -0.063 | 0.047 | 0.096 | 0.058 |
| LY2157299 | 3.70E2 nM |  |  | 0.015 | 0.007 | 0.044 | 0.101 | 0.056 | -0.025 | 0.101 | 0.034 |
| LY2157299 | 1.24E2 nM |  |  | 0.001 | 0.011 | 0.076 | 0.111 | 0.081 | -0.005 | 0.099 | 0.037 |
| LY2157299 | 4.12E1 nM |  |  | 0.010 | -0.002 | 0.078 | 0.090 | 0.130 | -0.002 | 0.179 | 0.040 |
| LY2157299 | 1.37E1 nM |  |  | -0.008 | -0.004 | 0.059 | 0.030 | 0.069 | 0.008 | 0.041 | 0.008 |
| LY2157299 | 4.60E0 nM |  |  | 0.007 | -0.004 | -0.003 | -0.051 | -0.022 | 0.059 | -0.011 | 0.024 |
| Lapatinib | 1.00E4 nM | Selleck Chem | Tyrosine Kinase | -0.328 | -0.080 | -1.459 | -0.482 | -1.089 | -0.183 | -0.012 | -0.234 |
| Lapatinib | 3.33E3 nM |  |  | -0.125 | 0.000 | -0.466 | -0.046 | -0.291 | -0.166 | 0.056 | -0.109 |
| Lapatinib | 1.11E3 nM |  |  | -0.065 | -0.014 | 0.067 | -0.048 | -0.111 | -0.151 | 0.046 | -0.043 |
| Lapatinib | 3.70E2 nM |  |  | -0.003 | -0.012 | -0.114 | 0.001 | -0.080 | -0.065 | -0.020 | -0.031 |
| Lapatinib | 1.24E2 nM |  |  | -0.022 | -0.021 | 0.111 | 0.001 | -0.047 | -0.104 | -0.036 | -0.070 |
| Lapatinib | 4.12E1 nM |  |  | 0.003 | -0.001 | 0.073 | 0.004 | -0.039 | -0.034 | -0.027 | -0.022 |
| Lapatinib | 1.37E1 nM |  |  | -0.020 | 0.050 | -0.022 | 0.055 | 0.052 | 0.016 | -0.032 | -0.020 |
| Lapatinib | 4.60E0 nM |  |  | 0.005 | 0.060 | -0.119 | 0.000 | -0.020 | 0.058 | -0.095 | 0.033 |
| Lenalidomide | 3.00E4 nM | EMD | thalidomide analog | -0.112 | -0.002 | -1.184 | -0.140 | -0.055 | -0.025 | 0.102 | 0.002 |
| Lenalidomide | 1.00E4 nM |  |  | -0.070 | 0.004 | -0.927 | -0.092 | -0.012 | 0.092 | 0.245 | 0.122 |
| Lenalidomide | 3.33E3 nM |  |  | 0.012 | 0.022 | -0.576 | 0.039 | 0.069 | 0.111 | 0.273 | 0.160 |
| Lenalidomide | 1.11E3 nM |  |  | 0.003 | 0.021 | -0.364 | 0.020 | -0.014 | 0.117 | 0.219 | 0.095 |
| M344 | 1.00E4 nM | Sigma | HDAC6 | -1.184 | -0.456 | -1.877 | -0.807 | -1.033 | -0.924 | -1.271 | -1.351 |
| M344 | 3.33E3 nM |  |  | -1.149 | -0.372 | -1.832 | -0.796 | -1.027 | -0.920 | -1.173 | -1.285 |
| M344 | 1.11E3 nM |  |  | -0.370 | -0.151 | -1.681 | -0.572 | -0.858 | -0.534 | -0.702 | -0.591 |
| M344 | 3.70E2 nM |  |  | -0.088 | -0.065 | -0.661 | -0.088 | -0.198 | -0.022 | -0.089 | -0.143 |
| M344 | 1.24E2 nM |  |  | -0.041 | -0.032 | -0.342 | -0.093 | -0.013 | 0.053 | -0.011 | -0.072 |
| M344 | 4.12E1 nM |  |  | -0.053 | 0.023 | 0.007 | -0.034 | -0.012 | 0.101 | -0.034 | 0.022 |
| MLN8054 | 3.00E4 nM | Selleck Chem | Aurora | -0.849 | -0.476 | -2.025 | -0.428 | -0.418 | -0.843 | -0.384 | -1.220 |
| MLN8054 | 1.00E4 nM |  |  | -0.404 | -0.283 | -1.936 | -0.260 | -0.288 | -0.183 | 0.028 | -0.363 |
| MLN8054 | 3.33E3 nM |  |  | -0.222 | -0.141 | -1.658 | -0.133 | -0.203 | -0.037 | 0.029 | -0.152 |
| MLN8054 | 1.11E3 nM |  |  | -0.120 | -0.040 | -1.475 | -0.073 | -0.138 | 0.017 | 0.067 | -0.043 |
| MLN8054 | 3.70E2 nM |  |  | -0.095 | -0.006 | -0.992 | -0.091 | -0.171 | 0.064 | -0.008 | 0.013 |
| MLN8054 | 1.24E2 nM |  |  | -0.026 | -0.013 | -0.323 | -0.100 | -0.118 | -0.013 | -0.006 | -0.005 |
| MLN8054 | 4.12E1 nM |  |  | -0.017 | 0.006 | -0.082 | -0.078 | -0.028 | 0.088 | -0.002 | -0.010 |
| MLN8054 | 1.37E1 nM |  |  | 0.011 | -0.002 | -0.015 | -0.108 | -0.177 | 0.069 | -0.051 | 0.029 |
| MS-275 | 1.00E4 nM | EMD | HDAC6 | -1.082 | -0.240 | -1.718 | -0.373 | -0.434 | -0.689 | -0.706 | -0.868 |
| MS-275 | 3.33E3 nM |  |  | -0.746 | -0.160 | -1.599 | -0.343 | -0.445 | -0.336 | -0.587 | -0.500 |
| MS-275 | 1.11E3 nM |  |  | -0.329 | -0.091 | -1.607 | -0.234 | -0.367 | -0.036 | -0.217 | -0.302 |
| MS-275 | 3.70E2 nM |  |  | -0.090 | -0.022 | -1.174 | -0.031 | -0.134 | 0.050 | -0.064 | -0.160 |
| MS-275 | 1.24E2 nM |  |  | -0.011 | -0.002 | -0.600 | 0.008 | -0.037 | -0.009 | -0.006 | -0.122 |
| MS-275 | 4.12E1 nM |  |  | 0.009 | -0.001 | 0.005 | 0.057 | 0.073 | 0.006 | 0.042 | 0.014 |
| MS-275 | 1.37E1 nM |  |  | 0.013 | -0.005 | -0.042 | -0.087 | 0.015 | 0.050 | -0.008 | 0.023 |
| MS-275 | 4.60E0 nM |  |  | 0.026 | -0.006 | -0.014 | -0.085 | -0.015 | 0.043 | -0.052 | 0.050 |
| Methotrexate | 3.00E4 nM | Sigma | Dihydrofolate Reductase Inhibitor | -0.054 | -0.010 | -0.962 | -0.127 | -0.110 | -0.031 | -0.103 | -0.053 |
| Methotrexate | 1.00E4 nM |  |  | -0.026 | -0.001 | -0.988 | -0.045 | 0.046 | -0.057 | 0.016 | -0.027 |
| Methotrexate | 3.33E3 nM |  |  | -0.029 | 0.004 | -1.022 | -0.160 | -0.084 | 0.009 | -0.056 | -0.045 |
| Methotrexate | 1.11E3 nM |  |  | -0.041 | 0.004 | -1.078 | -0.054 | 0.043 | 0.016 | -0.021 | -0.026 |
| Methotrexate | 3.70E2 nM |  |  | -0.003 | 0.020 | -1.103 | 0.031 | 0.068 | -0.045 | -0.015 | -0.032 |
| Methotrexate | 1.24E2 nM |  |  | -0.012 | 0.007 | -1.087 | -0.055 | 0.035 | -0.046 | -0.008 | -0.012 |
| Methotrexate | 4.12E1 nM |  |  | -0.020 | 0.006 | -0.918 | -0.047 | 0.052 | -0.026 | 0.005 | -0.009 |
| Methotrexate | 1.37E1 nM |  |  | -0.006 | 0.005 | -0.099 | -0.003 | 0.035 | 0.062 | -0.004 | 0.046 |
| Monensin Sodium | 3.33E2 nM | Active Biochem | Sodium Ionophore | -0.681 | -0.228 | -1.678 | -0.439 | -0.693 | -0.482 | -0.305 | -0.896 |
| Monensin Sodium | 1.11E2 nM |  |  | -0.506 | -0.200 | -1.814 | -0.472 | -0.800 | -0.338 | -0.344 | -0.737 |
| Monensin Sodium | 3.70E1 nM |  |  | -0.339 | -0.109 | -1.927 | -0.509 | -0.764 | -0.075 | -0.299 | -0.275 |
| Monensin Sodium | 1.23E1 nM |  |  | -0.066 | -0.068 | -0.583 | -0.182 | -0.413 | 0.168 | 0.020 | -0.056 |
| Monensin Sodium | 4.10E0 nM |  |  | 0.012 | 0.019 | -0.072 | 0.042 | -0.041 | 0.105 | 0.059 | 0.084 |
| Monensin Sodium | 1.40E0 nM |  |  | 0.019 | 0.001 | -0.150 | -0.034 | -0.038 | 0.049 | 0.026 | 0.089 |
| Monensin Sodium | 4.60E-1 nM |  |  | 0.009 | -0.004 | 0.075 | 0.069 | 0.075 | 0.060 | 0.046 | 0.045 |
| Monensin Sodium | 1.50E-1 nM |  |  | 0.005 | 0.007 | 0.080 | -0.038 | -0.127 | 0.072 | -0.023 | 0.035 |
| Montelukast | 1.00E4 nM | Sigma | CysLT1 Receptor Antagonist | 0.013 | 0.027 | -0.053 | -0.073 | -0.003 | 0.004 | -0.150 | -0.034 |
| Montelukast | 3.33E3 nM |  |  | -0.010 | -0.001 | 0.006 | -0.020 | 0.043 | -0.002 | -0.086 | -0.015 |
| Montelukast | 1.11E3 nM |  |  | 0.034 | 0.002 | 0.001 | -0.043 | -0.047 | 0.005 | -0.121 | 0.010 |
| Montelukast | 3.70E2 nM |  |  | 0.002 | 0.000 | -0.041 | -0.018 | -0.118 | 0.013 | -0.077 | -0.007 |
| Mycophenolic Acid | 3.33E3 nM | Tocris Cookson | IMPDH Inhibitor | -0.400 | -0.106 | -1.630 | -0.603 | -0.966 | -0.150 | -0.405 | -0.748 |
| Mycophenolic Acid | 1.11E3 nM |  |  | -0.316 | -0.080 | -1.735 | -0.625 | -1.068 | -0.135 | -0.377 | -0.602 |
| Mycophenolic Acid | 3.70E2 nM |  |  | -0.222 | -0.045 | -1.668 | -0.552 | -0.966 | -0.016 | -0.316 | -0.381 |
| NVP-AUY-922 | 1.00E4 nM | Selleck Chem | HSP90 inhibitor | -0.688 | -0.216 | -1.691 | -0.328 | -0.433 | -0.797 | -0.844 | -1.341 |
| NVP-AUY-922 | 3.33E3 nM |  |  | -0.678 | -0.188 | -1.577 | -0.362 | -0.459 | -0.838 | -0.898 | -1.559 |
| NVP-AUY-922 | 1.11E3 nM |  |  | -0.671 | -0.170 | -1.648 | -0.371 | -0.480 | -0.842 | -0.870 | -1.574 |
| NVP-AUY-922 | 3.70E2 nM |  |  | -0.640 | -0.150 | -1.681 | -0.456 | -0.683 | -0.883 | -0.796 | -1.494 |
| NVP-AUY-922 | 1.24E2 nM |  |  | -0.617 | -0.116 | -1.694 | -0.574 | -0.946 | -0.933 | -0.751 | -1.455 |
| NVP-AUY-922 | 4.12E1 nM |  |  | -0.626 | -0.115 | -1.701 | -0.576 | -0.975 | -0.942 | -0.757 | -1.554 |
| NVP-AUY-922 | 1.37E1 nM |  |  | -0.646 | -0.151 | -1.698 | -0.577 | -0.934 | -0.948 | -0.776 | -1.551 |
| NVP-AUY-922 | 4.60E0 nM |  |  | -0.055 | -0.029 | -0.689 | -0.154 | -0.165 | 0.060 | -0.088 | -0.120 |
| NVP-AUY-922 | 1.50E0 nM |  |  | 0.022 | 0.027 | -0.061 | -0.052 | -0.035 | -0.072 | 0.022 | -0.004 |
| NVP-AUY-922 | 5.10E-1 nM |  |  | 0.020 | 0.011 | 0.025 | -0.105 | 0.028 | 0.034 | 0.016 | 0.021 |
| NVP-AUY-922 | 1.70E-1 nM |  |  | -0.051 | -0.007 | -0.069 | 0.012 | 0.004 | 0.058 | -0.024 | 0.046 |
| ODN2006 | 5.00E1 nM | Invivogen | TLR9 | 0.120 | 0.090 | 0.259 | 0.035 | -0.104 | -0.550 | 0.897 | -0.009 |
| ODN2006 | 5.00E0 nM |  |  | -0.002 | 0.013 | -0.092 | -0.044 | -0.019 | 0.123 | 0.170 | 0.096 |
| ODN2006 | 5.00E-1 nM |  |  | -0.008 | -0.011 | -0.049 | -0.062 | 0.017 | 0.124 | 0.084 | 0.211 |
| ODN2006 | 5.00E-2 nM |  |  | -0.012 | -0.004 | -0.160 | -0.036 | -0.035 | 0.152 | 0.114 | 0.189 |
| OSI-906 | 1.00E4 nM | Selleck Chem | IGF-1R inhibitor | -0.173 | -0.095 | -0.924 | -0.127 | -0.230 | -0.194 | 0.340 | -0.136 |
| OSI-906 | 3.33E3 nM |  |  | -0.069 | -0.041 | -0.109 | -0.001 | 0.019 | -0.040 | 0.122 | -0.030 |
| OSI-906 | 1.11E3 nM |  |  | -0.066 | -0.013 | -0.119 | -0.038 | -0.038 | -0.049 | 0.001 | -0.082 |
| OSI-906 | 3.70E2 nM |  |  | -0.053 | -0.014 | -0.071 | -0.001 | -0.020 | -0.052 | -0.044 | -0.027 |
| PCI-32765 | 1.00E4 nM |  | BTK | -0.537 | -0.271 | -1.719 | -0.701 | -0.954 | -0.921 | -0.999 | -1.767 |
| PCI-32765 | 3.33E3 nM |  |  | -0.451 | -0.257 | -1.653 | -0.599 | -0.975 | -0.846 | -0.292 | -1.135 |
| PCI-32765 | 1.11E3 nM |  |  | -0.363 | -0.184 | -1.746 | -0.478 | -0.868 | -0.761 | -0.328 | -0.739 |
| PCI-32765 | 3.70E2 nM |  |  | -0.297 | -0.149 | -1.729 | -0.260 | -0.173 | -0.666 | -0.316 | -0.817 |
| PCI-32765 | 1.24E2 nM |  |  | -0.284 | -0.132 | -1.703 | -0.237 | -0.280 | -0.489 | -0.233 | -0.562 |
| PCI-32765 | 4.12E1 nM |  |  | -0.246 | -0.127 | -1.740 | -0.019 | -0.056 | -0.413 | -0.175 | -0.387 |
| PCI-32765 | 1.37E1 nM |  |  | -0.216 | -0.111 | -1.716 | -0.122 | 0.089 | -0.371 | -0.170 | -0.386 |
| PCI-32765 | 4.60E0 nM |  |  | -0.194 | -0.096 | -1.552 | -0.008 | -0.001 | -0.270 | -0.217 | -0.257 |
| PD098059 | 3.00E4 nM | Sigma | MEK | -0.018 | -0.036 | -0.324 | -0.175 | -0.278 | -0.089 | -0.016 | -0.216 |
| PD098059 | 1.00E4 nM |  |  | 0.006 | -0.021 | -0.310 | -0.094 | -0.292 | -0.019 | 0.094 | -0.123 |
| PD098059 | 3.33E3 nM |  |  | 0.031 | -0.008 | -0.097 | -0.007 | -0.135 | -0.014 | 0.149 | -0.063 |
| PD098059 | 1.11E3 nM |  |  | 0.031 | -0.003 | -0.098 | 0.082 | -0.029 | -0.004 | 0.187 | -0.030 |
| PD184352 | 3.33E3 nM | Tocris Cookson | MEK | -0.184 | -0.064 | -0.105 | -0.558 | -0.906 | -0.315 | -0.441 | -0.740 |
| PD184352 | 1.11E3 nM |  |  | -0.095 | -0.046 | 0.041 | -0.471 | -0.706 | -0.242 | -0.307 | -0.404 |
| PD184352 | 3.70E2 nM |  |  | -0.065 | -0.025 | 0.082 | -0.251 | -0.460 | -0.174 | -0.185 | -0.215 |
| PD184352 | 1.24E2 nM |  |  | 0.007 | -0.004 | -0.075 | -0.173 | -0.276 | -0.069 | -0.125 | -0.051 |
| PDGF-BB | 4.00E0 nM |  | PDGFRbeta ligand | 0.015 | 0.027 | -0.148 | 0.037 | -0.239 | 0.071 | -0.039 | 0.063 |
| PDGF-BB | 1.30E0 nM |  |  | 0.027 | -0.011 | 0.022 | 0.048 | -0.169 | -0.011 | 0.018 | 0.013 |
| PDGF-BB | 4.40E-1 nM |  |  | 0.011 | -0.012 | -0.003 | -0.023 | -0.103 | 0.016 | 0.020 | 0.002 |
| PDGF-BB | 1.50E-1 nM |  |  | 0.008 | -0.014 | 0.048 | 0.049 | -0.088 | 0.057 | 0.032 | -0.009 |
| PF-2341066 | 1.00E4 nM | Active Biochem | ALK inhibitor | -0.003 | -0.015 | 0.060 | -0.077 | -0.016 | 0.095 | 0.050 | 0.081 |
| PF-2341066 | 3.33E3 nM |  |  | 0.010 | 0.026 | -0.065 | 0.007 | 0.063 | 0.023 | 0.100 | 0.057 |
| PF-2341066 | 1.11E3 nM |  |  | 0.008 | -0.015 | 0.005 | -0.008 | 0.036 | 0.025 | 0.105 | 0.056 |
| PF-2341066 | 3.70E2 nM |  |  | -0.029 | -0.006 | -0.023 | 0.038 | 0.105 | 0.041 | 0.080 | 0.063 |
| PF-2341066 | 1.24E2 nM |  |  | 0.007 | 0.002 | 0.050 | 0.023 | 0.081 | -0.008 | 0.049 | 0.003 |
| PF-2341066 | 4.12E1 nM |  |  | -0.021 | -0.010 | -0.094 | 0.027 | 0.055 | 0.030 | 0.059 | 0.047 |
| PF-2341066 | 1.37E1 nM |  |  | 0.018 | 0.005 | -0.092 | -0.001 | 0.089 | 0.009 | 0.042 | 0.032 |
| PF-2341066 | 4.60E0 nM |  |  | 0.012 | 0.010 | -0.127 | -0.071 | 0.046 | 0.089 | -0.020 | 0.030 |
| PP 242 | 1.00E3 nM | Selleck Chem | mTOR | -0.521 | -0.191 | -1.628 | -0.526 | -0.803 | -0.515 | -0.959 | -1.005 |
| PP 242 | 1.00E2 nM |  |  | -0.401 | -0.160 | -1.523 | -0.499 | -0.574 | -0.252 | -0.900 | -0.658 |
| PP 242 | 1.00E1 nM |  |  | -0.276 | -0.097 | -0.922 | -0.481 | -0.286 | -0.082 | -0.706 | -0.364 |
| PP 242 | 1.00E0 nM |  |  | -0.155 | -0.058 | -0.374 | -0.388 | -0.263 | 0.076 | -0.356 | -0.064 |
| PR-171 | 1.00E4 nM | Active Biochem | proteasome inhibitor | -1.212 | -0.718 | -1.712 | -0.361 | -0.454 | -0.860 | -0.794 | -1.412 |
| PR-171 | 3.33E3 nM |  |  | -1.208 | -0.696 | -1.650 | -0.387 | -0.447 | -0.879 | -0.804 | -1.465 |
| PR-171 | 1.11E3 nM |  |  | -1.187 | -0.687 | -1.748 | -0.442 | -0.681 | -0.755 | -0.810 | -1.440 |
| PR-171 | 3.70E2 nM |  |  | -1.184 | -0.678 | -1.757 | -0.466 | -0.718 | -0.746 | -0.976 | -1.451 |
| PR-171 | 1.24E2 nM |  |  | -1.161 | -0.669 | -1.805 | -0.576 | -0.952 | -0.658 | -0.922 | -1.427 |
| PR-171 | 4.12E1 nM |  |  | -1.144 | -0.619 | -1.870 | -0.585 | -0.955 | -0.656 | -0.844 | -1.423 |
| PR-171 | 1.37E1 nM |  |  | -0.928 | -0.384 | -1.857 | -0.330 | -0.527 | -0.576 | -0.747 | -1.020 |
| PR-171 | 4.60E0 nM |  |  | -0.363 | -0.113 | -1.168 | -0.086 | -0.281 | -0.212 | -0.303 | -0.358 |
| PR-171 | 1.50E0 nM |  |  | -0.009 | -0.011 | -0.004 | -0.036 | -0.089 | -0.011 | -0.115 | -0.121 |
| PR-171 | 5.10E-1 nM |  |  | -0.051 | -0.015 | 0.022 | 0.018 | 0.005 | 0.086 | -0.070 | -0.006 |
| Paclitaxel | 1.00E4 nM | EMD | Microtubule | -0.179 | 0.015 | -1.567 | -0.450 | -0.567 | 0.000 | -0.261 | -0.130 |
| Paclitaxel | 3.33E3 nM |  |  | -0.203 | -0.002 | -1.527 | -0.210 | -0.428 | 0.029 | -0.154 | -0.123 |
| Paclitaxel | 1.11E3 nM |  |  | -0.235 | -0.008 | -1.496 | -0.212 | -0.460 | -0.003 | -0.108 | -0.116 |
| Paclitaxel | 3.70E2 nM |  |  | -0.235 | -0.006 | -1.503 | -0.163 | -0.400 | 0.034 | -0.158 | -0.108 |
| Paclitaxel | 1.24E2 nM |  |  | -0.215 | 0.000 | -1.490 | -0.215 | -0.363 | 0.004 | -0.151 | -0.108 |
| Paclitaxel | 4.12E1 nM |  |  | -0.197 | 0.007 | -1.518 | -0.186 | -0.250 | 0.024 | -0.054 | -0.070 |
| Paclitaxel | 1.37E1 nM |  |  | -0.126 | 0.000 | -1.351 | -0.090 | -0.122 | -0.019 | -0.130 | -0.039 |
| Paclitaxel | 4.60E0 nM |  |  | -0.039 | 0.002 | -0.517 | -0.138 | -0.104 | 0.054 | -0.101 | -0.003 |
| Paclitaxel | 1.50E0 nM |  |  | 0.012 | -0.013 | -0.270 | -0.156 | -0.041 | 0.083 | -0.058 | 0.014 |
| Pam3CSK4 | 1.00E2 ng/ml | Invivogen | TLR2/1 | 0.006 | 0.042 | 0.144 | 0.154 | 0.238 | -0.004 | 1.017 | -0.098 |
| Pam3CSK4 | 1.00E1 ng/ml |  |  | 0.033 | 0.024 | 0.107 | 0.103 | 0.149 | -0.017 | 0.696 | -0.096 |
| Pam3CSK4 | 1.00E0 ng/ml |  |  | 0.014 | 0.007 | 0.094 | 0.044 | 0.037 | 0.037 | 0.214 | -0.038 |
| Pam3CSK4 | 1.00E-1 ng/ml |  |  | 0.017 | 0.009 | -0.049 | 0.051 | 0.019 | 0.065 | 0.119 | 0.029 |
| Paraquat Dichloride | 1.00E4 nM | Sigma | oxidative stress stimulant | -0.260 | -0.063 | -0.961 | 0.050 | -0.031 | -0.129 | 0.006 | -0.155 |
| Paraquat Dichloride | 3.33E3 nM |  |  | -0.083 | -0.039 | -0.128 | -0.005 | 0.058 | -0.081 | 0.012 | -0.047 |
| Paraquat Dichloride | 1.11E3 nM |  |  | -0.022 | -0.024 | 0.006 | -0.003 | 0.005 | -0.048 | 0.005 | 0.031 |
| Paraquat Dichloride | 3.70E2 nM |  |  | 0.002 | -0.010 | -0.021 | 0.081 | 0.032 | 0.001 | 0.008 | 0.036 |
| Pemetrexed Disodium | 1.00E4 nM | Toronto Research Chemical | Dihydrofolate Reductase Inhibitor | -0.035 | -0.007 | -1.077 | -0.138 | -0.152 | 0.006 | 0.046 | -0.009 |
| Pemetrexed Disodium | 3.33E3 nM |  |  | -0.026 | 0.001 | -1.074 | -0.107 | -0.027 | -0.008 | -0.086 | -0.036 |
| Pemetrexed Disodium | 1.11E3 nM |  |  | -0.012 | -0.004 | -1.081 | -0.126 | -0.045 | -0.005 | 0.030 | -0.009 |
| Pemetrexed Disodium | 3.70E2 nM |  |  | -0.007 | -0.008 | -1.023 | -0.027 | -0.009 | -0.036 | -0.024 | 0.001 |
| Pemetrexed Disodium | 1.24E2 nM |  |  | -0.006 | 0.009 | -0.959 | 0.032 | 0.013 | -0.031 | -0.010 | -0.012 |
| Pemetrexed Disodium | 4.12E1 nM |  |  | 0.019 | 0.010 | -0.585 | -0.026 | 0.042 | -0.014 | -0.001 | 0.007 |
| Pemetrexed Disodium | 1.37E1 nM |  |  | 0.012 | -0.002 | 0.007 | -0.070 | -0.005 | -0.027 | 0.013 | -0.001 |
| Pemetrexed Disodium | 4.60E0 nM |  |  | 0.027 | 0.012 | -0.050 | -0.106 | 0.000 | 0.065 | -0.041 | 0.062 |
| Picropodophyllin | 1.00E4 nM | Sigma | Microtubule | -0.480 | -0.123 | -1.687 | -0.166 | -0.378 | 0.251 | -0.272 | -0.217 |
| Picropodophyllin | 3.33E3 nM |  |  | -0.415 | -0.110 | -1.598 | -0.140 | -0.356 | 0.184 | -0.371 | -0.180 |
| Picropodophyllin | 1.11E3 nM |  |  | -0.302 | -0.041 | -1.540 | -0.010 | -0.242 | 0.126 | -0.077 | -0.084 |
| Picropodophyllin | 3.70E2 nM |  |  | -0.111 | 0.002 | -0.805 | -0.072 | -0.104 | 0.057 | -0.070 | 0.008 |
| Picropodophyllin | 1.24E2 nM |  |  | -0.007 | 0.003 | -0.049 | 0.057 | 0.052 | 0.112 | -0.007 | 0.080 |
| Picropodophyllin | 4.12E1 nM |  |  | -0.035 | -0.001 | -0.004 | 0.042 | -0.035 | 0.043 | -0.040 | 0.027 |
| Picropodophyllin | 1.37E1 nM |  |  | 0.005 | 0.009 | -0.041 | 0.040 | 0.036 | 0.068 | -0.038 | 0.053 |
| Picropodophyllin | 4.60E0 nM |  |  | -0.024 | 0.001 | -0.131 | 0.001 | -0.089 | 0.109 | -0.062 | 0.049 |
| Pirfenidone | 5.00E6 nM | Sigma | p38γ | -0.820 | -0.332 | -1.814 | -0.533 | -0.766 | -0.805 | -1.053 | -1.432 |
| Pirfenidone | 1.67E6 nM |  |  | -0.222 | -0.084 | -1.474 | -0.413 | -0.414 | -0.260 | -0.240 | -0.433 |
| Pirfenidone | 1.00E6 nM |  |  | -0.101 | -0.039 | -0.917 | -0.226 | -0.174 | -0.496 | 0.019 | -0.286 |
| Pirfenidone | 5.56E5 nM |  |  | -0.026 | 0.025 | -0.448 | -0.098 | -0.019 | -0.094 | 0.052 | -0.117 |
| Pirfenidone | 3.33E5 nM |  |  | 0.014 | -0.003 | -0.093 | 0.144 | 0.069 | -0.290 | 0.156 | -0.118 |
| Pirfenidone | 1.85E5 nM |  |  | -0.018 | 0.049 | -0.018 | 0.045 | -0.025 | 0.013 | 0.046 | -0.060 |
| Pirfenidone | 1.11E5 nM |  |  | 0.010 | 0.014 | -0.027 | 0.181 | 0.201 | -0.281 | 0.236 | -0.084 |
| Pirfenidone | 3.00E4 nM |  |  | 0.011 | 0.031 | 0.094 | 0.045 | 0.069 | 0.082 | 0.063 | 0.039 |
| Pirfenidone | 1.00E4 nM |  |  | -0.011 | 0.003 | -0.027 | 0.144 | 0.119 | 0.041 | 0.159 | -0.047 |
| Pirfenidone | 3.33E3 nM |  |  | 0.021 | -0.015 | 0.012 | 0.064 | 0.075 | 0.053 | -0.007 | 0.020 |
| Pirfenidone | 1.11E3 nM |  |  | -0.002 | -0.014 | -0.073 | 0.119 | 0.059 | 0.024 | 0.021 | -0.029 |
| Pirfenidone | 3.70E2 nM |  |  | -0.005 | 0.000 | 0.079 | 0.027 | 0.038 | 0.088 | -0.032 | 0.009 |
| Pirfenidone | 1.24E2 nM |  |  | -0.034 | -0.016 | 0.103 | 0.081 | 0.056 | 0.054 | -0.008 | 0.067 |
| Pirfenidone | 4.12E1 nM |  |  | -0.025 | -0.007 | 0.074 | 0.029 | -0.039 | -0.030 | 0.051 | 0.028 |
| Pirfenidone | 1.37E1 nM |  |  | -0.001 | -0.004 | 0.026 | 0.029 | 0.049 | 0.015 | 0.024 | 0.028 |
| Poly(I:C)HMW | 1.00E3 ng/ml | Invivogen | TLR3 | -0.012 | -0.016 | -0.084 | 0.061 | -0.069 | 0.116 | 0.115 | 0.053 |
| Poly(I:C)HMW | 1.00E2 ng/ml |  |  | 0.011 | -0.003 | -0.073 | 0.034 | 0.037 | 0.079 | 0.110 | 0.012 |
| Poly(I:C)HMW | 1.00E1 ng/ml |  |  | 0.011 | 0.003 | -0.091 | 0.057 | 0.005 | -0.028 | 0.065 | -0.025 |
| Poly(I:C)HMW | 1.00E0 ng/ml |  |  | -0.004 | 0.016 | -0.112 | -0.001 | 0.048 | 0.064 | 0.101 | 0.005 |
| Poly(I_C)LMW | 1.00E3 ng/ml |  | TLR3 | 0.001 | -0.014 | -0.077 | -0.032 | -0.015 | 0.193 | 0.142 | 0.195 |
| Poly(I_C)LMW | 1.00E2 ng/ml |  |  | -0.005 | -0.008 | -0.006 | -0.005 | 0.077 | 0.107 | 0.127 | 0.068 |
| Poly(I_C)LMW | 1.00E1 ng/ml |  |  | 0.003 | -0.001 | -0.019 | 0.011 | -0.005 | 0.103 | 0.116 | 0.026 |
| Poly(I_C)LMW | 1.00E0 ng/ml |  |  | 0.031 | 0.011 | -0.203 | -0.063 | 0.015 | 0.114 | 0.088 | 0.046 |
| Prednisolone | 3.33E3 nM | Sigma | GR Agonist | -0.189 | -0.117 | -0.444 | -0.487 | -0.726 | -0.593 | 0.043 | -0.556 |
| Prednisolone | 1.11E3 nM |  |  | -0.159 | -0.109 | -0.359 | -0.390 | -0.479 | -0.507 | 0.014 | -0.437 |
| Prednisolone | 3.70E2 nM |  |  | -0.178 | -0.123 | -0.930 | -0.344 | -0.674 | -0.485 | 0.086 | -0.416 |
| Prednisolone | 1.24E2 nM |  |  | -0.086 | -0.079 | -0.217 | -0.173 | -0.119 | -0.489 | 0.073 | -0.398 |
| Prednisolone | 4.12E1 nM |  |  | -0.073 | -0.053 | 0.052 | -0.175 | -0.067 | -0.384 | 0.105 | -0.271 |
| Prednisolone | 1.37E1 nM |  |  | -0.008 | -0.028 | 0.003 | -0.262 | -0.065 | -0.026 | 0.231 | -0.054 |
| Prostaglandin E1 | 1.00E4 nM | Sigma | EP Agonist | -0.058 | -0.097 | -0.053 | 0.099 | -0.220 | -0.114 | 0.547 | -0.572 |
| Prostaglandin E1 | 3.33E3 nM |  |  | -0.070 | -0.115 | 0.085 | 0.174 | -0.140 | -0.138 | 0.521 | -0.505 |
| Prostaglandin E1 | 1.11E3 nM |  |  | -0.064 | -0.096 | 0.081 | 0.143 | -0.173 | -0.107 | 0.497 | -0.487 |
| Prostaglandin E1 | 3.70E2 nM |  |  | -0.061 | -0.097 | 0.074 | 0.190 | -0.153 | -0.122 | 0.529 | -0.440 |
| Prostaglandin E2 | 1.00E4 nM | Sigma | EP Agonist | -0.141 | -0.093 | -0.565 | 0.019 | -0.248 | -0.213 | 0.356 | -0.688 |
| Prostaglandin E2 | 3.33E3 nM |  |  | -0.066 | -0.090 | -0.005 | 0.313 | -0.245 | -0.245 | 0.729 | -0.466 |
| Prostaglandin E2 | 1.11E3 nM |  |  | -0.062 | -0.052 | 0.002 | 0.190 | -0.197 | -0.174 | 0.483 | -0.453 |
| Prostaglandin E2 | 3.70E2 nM |  |  | -0.066 | -0.067 | 0.042 | 0.174 | -0.159 | -0.124 | 0.359 | -0.394 |
| R(-)rolipram | 1.00E4 nM | Biomol | PDE IV Inhibitor | -0.071 | -0.034 | -0.519 | -0.093 | -0.162 | 0.165 | 0.075 | 0.036 |
| R(-)rolipram | 3.33E3 nM |  |  | -0.041 | -0.048 | -0.335 | -0.012 | -0.055 | 0.078 | 0.154 | -0.014 |
| R(-)rolipram | 1.11E3 nM |  |  | -0.067 | -0.032 | -0.223 | 0.003 | -0.018 | 0.057 | 0.103 | 0.011 |
| R(-)rolipram | 3.70E2 nM |  |  | -0.009 | -0.010 | -0.078 | 0.017 | -0.062 | 0.053 | 0.083 | -0.009 |
| RANTES | 3.00E0 nM | R&D system | RANTES receptor ligand | 0.001 | 0.006 | -0.071 | 0.084 | 0.035 | -0.047 | 0.093 | -0.018 |
| RANTES | 1.00E0 nM |  |  | 0.004 | 0.018 | -0.010 | 0.020 | -0.055 | -0.018 | 0.118 | 0.014 |
| RANTES | 3.30E-1 nM |  |  | 0.015 | 0.029 | 0.085 | -0.049 | 0.001 | 0.030 | 0.068 | 0.024 |
| RANTES | 1.10E-1 nM |  |  | -0.025 | 0.026 | -0.079 | -0.036 | 0.029 | 0.091 | -0.045 | 0.056 |
| RO 320-1195 | 3.33E3 nM | Roche | p38 MAPK | 0.011 | -0.038 | -0.243 | -0.232 | -0.100 | 0.070 | -0.118 | 0.017 |
| RO 320-1195 | 1.11E3 nM |  |  | 0.016 | -0.022 | -0.307 | -0.103 | 0.023 | 0.034 | -0.101 | 0.012 |
| RO 320-1195 | 3.70E2 nM |  |  | 0.000 | -0.005 | -0.164 | -0.109 | -0.068 | -0.008 | -0.069 | -0.025 |
| RO 320-1195 | 1.24E2 nM |  |  | -0.002 | -0.008 | -0.091 | -0.026 | 0.008 | 0.039 | -0.036 | 0.056 |
| RO-5126766 | 1.00E4 nM | Active Biochem | MEK | -0.294 | -0.097 | -0.830 | -0.342 | -0.408 | -0.703 | -0.737 | -1.336 |
| RO-5126766 | 3.33E3 nM |  |  | -0.337 | -0.116 | -0.566 | -0.357 | -0.406 | -0.756 | -0.781 | -1.367 |
| RO-5126766 | 1.11E3 nM |  |  | -0.259 | -0.093 | -0.604 | -0.309 | -0.355 | -0.694 | -0.677 | -1.206 |
| RO-5126766 | 3.70E2 nM |  |  | -0.185 | -0.088 | -0.342 | -0.281 | -0.359 | -0.589 | -0.517 | -0.951 |
| Raltitrexed | 3.00E4 nM | Toronto Research Chemical | Thymidylate Synthase | 0.019 | 0.011 | -0.951 | 0.030 | -0.014 | 0.038 | 0.128 | 0.038 |
| Raltitrexed | 1.00E4 nM |  |  | -0.007 | 0.003 | -0.987 | 0.144 | 0.032 | -0.027 | 0.267 | 0.050 |
| Raltitrexed | 3.33E3 nM |  |  | -0.018 | 0.000 | -1.042 | 0.075 | 0.066 | -0.002 | 0.069 | 0.011 |
| Raltitrexed | 1.11E3 nM |  |  | -0.003 | -0.005 | -0.978 | -0.023 | -0.016 | 0.026 | 0.013 | 0.018 |
| Raltitrexed | 3.70E2 nM |  |  | 0.000 | 0.007 | -1.046 | 0.012 | -0.012 | -0.060 | 0.085 | -0.020 |
| Raltitrexed | 1.24E2 nM |  |  | 0.026 | 0.005 | -1.052 | 0.021 | 0.041 | -0.060 | 0.087 | -0.003 |
| Raltitrexed | 4.12E1 nM |  |  | 0.000 | -0.011 | -0.959 | -0.023 | -0.013 | -0.009 | 0.026 | 0.002 |
| Raltitrexed | 1.37E1 nM |  |  | 0.001 | 0.005 | -0.993 | -0.016 | -0.037 | 0.042 | -0.004 | 0.026 |
| Rapamycin | 1.00E3 nM | EMD | mTOR | -0.362 | -0.063 | -2.005 | -0.885 | -0.856 | -0.128 | -0.150 | -0.135 |
| Rapamycin | 3.33E2 nM |  |  | -0.385 | -0.068 | -2.064 | -0.942 | -0.939 | -0.091 | -0.251 | -0.179 |
| Rapamycin | 1.11E2 nM |  |  | -0.368 | -0.078 | -1.791 | -0.989 | -1.032 | -0.272 | -0.373 | -0.227 |
| Rapamycin | 3.70E1 nM |  |  | -0.347 | -0.099 | -1.743 | -0.929 | -0.854 | -0.342 | -0.283 | -0.232 |
| Rapamycin | 1.23E1 nM |  |  | -0.363 | -0.088 | -1.690 | -0.823 | -0.797 | -0.362 | -0.444 | -0.291 |
| Rapamycin | 4.10E0 nM |  |  | -0.362 | -0.097 | -1.639 | -0.757 | -0.712 | -0.410 | -0.473 | -0.357 |
| Rapamycin | 1.40E0 nM |  |  | -0.335 | -0.084 | -1.641 | -0.789 | -0.643 | -0.272 | -0.383 | -0.318 |
| Rapamycin | 4.60E-1 nM |  |  | -0.258 | -0.074 | -1.475 | -0.660 | -0.559 | -0.311 | -0.397 | -0.281 |
| Remicade | 1.00E4 ng/ml | VWR | TNF-alpha Antagonist | -0.040 | -0.037 | 0.116 | -0.162 | -0.192 | -0.094 | 0.188 | -0.886 |
| Remicade | 1.00E3 ng/ml |  |  | -0.049 | -0.049 | 0.000 | -0.075 | -0.131 | -0.049 | 0.220 | -0.764 |
| Remicade | 1.00E2 ng/ml |  |  | 0.022 | -0.009 | 0.049 | 0.048 | 0.092 | 0.048 | 0.101 | -0.110 |
| Remicade | 1.00E1 ng/ml |  |  | 0.032 | 0.010 | 0.037 | 0.069 | -0.024 | 0.061 | 0.026 | 0.034 |
| Ro-32-0432 | 3.00E3 nM | Selleck Chem | PKC (c+n) | -0.315 | -0.111 | -0.803 | -0.327 | -0.399 | -0.936 | -0.170 | -0.986 |
| Ro-32-0432 | 1.00E3 nM |  |  | -0.178 | -0.071 | -0.532 | -0.179 | -0.190 | -0.837 | -0.045 | -0.661 |
| Ro-32-0432 | 3.33E2 nM |  |  | -0.055 | -0.024 | -0.244 | -0.180 | -0.094 | -0.570 | -0.017 | -0.356 |
| Ro-32-0432 | 1.11E2 nM |  |  | -0.005 | -0.006 | -0.061 | -0.032 | -0.063 | -0.301 | 0.106 | -0.167 |
| Roflumilast | 3.00E4 nM | Active Biochem | PDE IV Inhibitor | -0.041 | -0.035 | -0.202 | -0.026 | 0.008 | 0.100 | 0.173 | 0.020 |
| Roflumilast | 1.00E4 nM |  |  | -0.024 | -0.035 | -0.326 | 0.127 | 0.001 | 0.064 | 0.186 | -0.030 |
| Roflumilast | 3.33E3 nM |  |  | -0.013 | -0.036 | -0.145 | -0.092 | -0.062 | 0.079 | 0.132 | -0.014 |
| Roflumilast | 1.11E3 nM |  |  | -0.027 | -0.042 | -0.204 | -0.114 | -0.031 | 0.147 | -0.036 | 0.038 |
| SB-939 | 1.00E4 nM | Selleck Chem | HDAC Inhibitor | -1.118 | -0.426 | -1.675 | -0.367 | -0.436 | -0.833 | -1.038 | -1.196 |
| SB-939 | 3.33E3 nM |  |  | -1.127 | -0.414 | -1.610 | -0.367 | -0.457 | -0.852 | -1.172 | -1.263 |
| SB-939 | 1.11E3 nM |  |  | -1.044 | -0.302 | -1.610 | -0.370 | -0.475 | -0.852 | -1.151 | -1.212 |
| SB-939 | 3.70E2 nM |  |  | -0.305 | -0.126 | -1.558 | -0.172 | -0.369 | -0.458 | -0.665 | -0.517 |
| SGI-1776 | 3.00E4 nM | Selleck Chem | Pim kinase inhibitor | -1.203 | -0.874 | -1.732 | -0.356 | -0.453 | -0.841 | -1.396 | -1.598 |
| SGI-1776 | 1.00E4 nM |  |  | -1.052 | -0.379 | -1.777 | -0.358 | -0.456 | -0.841 | -1.253 | -1.562 |
| SGI-1776 | 3.33E3 nM |  |  | -0.296 | -0.145 | -0.751 | -0.335 | -0.378 | -0.498 | -0.114 | -0.544 |
| SGI-1776 | 1.11E3 nM |  |  | -0.063 | -0.035 | -0.070 | -0.177 | -0.077 | -0.104 | -0.135 | -0.076 |
| SR1001 | 1.00E4 nM | Scripps | RORα/γ | -0.044 | 0.039 | -0.111 | -0.149 | -0.229 | 0.011 | -0.054 | 0.039 |
| SR1001 | 3.33E3 nM |  |  | -0.004 | 0.002 | -0.059 | 0.035 | 0.069 | -0.029 | 0.030 | -0.020 |
| SR1001 | 1.11E3 nM |  |  | -0.031 | -0.012 | 0.040 | -0.014 | -0.022 | -0.030 | -0.001 | -0.002 |
| SR1001 | 3.70E2 nM |  |  | 0.030 | -0.016 | 0.120 | 0.004 | -0.021 | -0.021 | -0.029 | -0.019 |
| SR2211 | 1.00E4 nM | Scripps | RORγ | -0.367 | -0.032 | -1.685 | -0.665 | -1.358 | -0.144 | -0.255 | -0.379 |
| SR2211 | 3.33E3 nM |  |  | -0.089 | 0.002 | -0.560 | -0.444 | -0.571 | 0.002 | -0.034 | -0.039 |
| SR2211 | 1.11E3 nM |  |  | -0.019 | 0.047 | -0.078 | -0.262 | -0.168 | 0.045 | -0.027 | 0.017 |
| SR2211 | 3.70E2 nM |  |  | -0.028 | 0.001 | -0.117 | -0.157 | -0.050 | 0.124 | -0.088 | 0.057 |
| Shaoguamycin B | 1.11E2 nM | Sigma | NADH Dehydrogenase Inhibitor | -0.612 | -0.131 | -1.848 | -0.441 | -0.522 | -0.959 | -0.932 | -1.133 |
| Shaoguamycin B | 3.70E1 nM |  |  | -0.503 | -0.098 | -1.801 | -0.438 | -0.472 | -0.763 | -0.502 | -0.957 |
| Shaoguamycin B | 1.23E1 nM |  |  | -0.464 | -0.073 | -1.798 | -0.463 | -0.479 | -0.637 | -0.468 | -0.836 |
| Shaoguamycin B | 4.10E0 nM |  |  | -0.453 | -0.072 | -1.749 | -0.501 | -0.511 | -0.599 | -0.475 | -0.802 |
| Shaoguamycin B | 1.40E0 nM |  |  | -0.453 | -0.074 | -1.739 | -0.454 | -0.299 | -0.506 | -0.423 | -0.733 |
| Shaoguamycin B | 4.60E-1 nM |  |  | -0.422 | -0.073 | -1.654 | -0.356 | 0.055 | -0.560 | -0.435 | -0.675 |
| Shaoguamycin B | 1.50E-1 nM |  |  | -0.360 | -0.056 | -1.487 | -0.286 | -0.061 | -0.477 | -0.391 | -0.647 |
| Shaoguamycin B | 5.10E-2 nM |  |  | -0.258 | -0.032 | -0.916 | -0.331 | -0.004 | -0.314 | -0.176 | -0.383 |
| Syk Inhibitor | 1.00E4 nM | EMD | Syk | -0.307 | -0.115 | -1.181 | -0.661 | -0.647 | -0.401 | 0.267 | -0.596 |
| Syk Inhibitor | 3.33E3 nM |  |  | -0.231 | -0.102 | -0.581 | -0.402 | -0.837 | -0.221 | -0.001 | -0.512 |
| Syk Inhibitor | 1.11E3 nM |  |  | -0.238 | -0.072 | -0.010 | -0.270 | -0.379 | -0.248 | -0.297 | -0.412 |
| Syk Inhibitor | 3.70E2 nM |  |  | -0.129 | -0.059 | -0.103 | -0.219 | -0.032 | -0.123 | 0.122 | -0.135 |
| Temsirolimus | 1.11E3 nM | Active Biochem | mTOR | -0.365 | -0.098 | -1.644 | -0.568 | -0.783 | -0.577 | -0.738 | -0.579 |
| Temsirolimus | 3.70E2 nM |  |  | -0.388 | -0.066 | -1.606 | -0.436 | -0.802 | -0.623 | -0.760 | -0.711 |
| Temsirolimus | 1.24E2 nM |  |  | -0.433 | -0.115 | -1.621 | -0.550 | -0.678 | -0.371 | -0.760 | -0.446 |
| Temsirolimus | 4.12E1 nM |  |  | -0.386 | -0.098 | -1.665 | -0.544 | -0.671 | -0.405 | -0.748 | -0.452 |
| Temsirolimus | 1.37E1 nM |  |  | -0.395 | -0.101 | -1.653 | -0.546 | -0.634 | -0.404 | -0.739 | -0.447 |
| Temsirolimus | 4.60E0 nM |  |  | -0.388 | -0.106 | -1.627 | -0.521 | -0.645 | -0.372 | -0.718 | -0.418 |
| Temsirolimus | 1.50E0 nM |  |  | -0.397 | -0.109 | -1.627 | -0.536 | -0.619 | -0.334 | -0.771 | -0.390 |
| Temsirolimus | 5.10E-1 nM |  |  | -0.379 | -0.098 | -1.619 | -0.540 | -0.630 | -0.307 | -0.712 | -0.346 |
| Tin(II) Chloride | 3.00E4 nM | Sigma | antioxidant | -0.110 | -0.017 | -0.017 | -0.059 | -0.108 | -0.040 | -0.138 | -0.160 |
| Tin(II) Chloride | 1.00E4 nM |  |  | -0.041 | -0.004 | -0.002 | 0.001 | 0.016 | -0.006 | -0.027 | -0.085 |
| Tin(II) Chloride | 3.33E3 nM |  |  | -0.008 | -0.010 | 0.057 | -0.022 | 0.016 | 0.026 | -0.015 | -0.046 |
| Tin(II) Chloride | 1.11E3 nM |  |  | 0.003 | -0.012 | 0.085 | 0.085 | 0.117 | -0.052 | 0.066 | -0.033 |
| Torcetrapib | 3.00E4 nM | Toronto Research Chemical | CETP inhibitor | -0.119 | 0.015 | -0.976 | -0.465 | -0.414 | -0.259 | -0.223 | -0.165 |
| Torcetrapib | 1.00E4 nM |  |  | -0.034 | -0.028 | -1.088 | -0.369 | -0.327 | -0.304 | -0.225 | -0.188 |
| Torcetrapib | 3.00E3 nM |  |  | -0.021 | -0.010 | -0.305 | -0.139 | -0.156 | -0.188 | -0.109 | -0.061 |
| Torcetrapib | 1.00E3 nM |  |  | 0.030 | -0.012 | -0.023 | -0.041 | 0.014 | 0.008 | 0.070 | 0.000 |
| Torin-1 | 1.00E3 nM | Tocris Cookson | mTOR | -0.591 | -0.220 | -1.609 | -0.566 | -0.896 | -0.670 | -0.797 | -1.235 |
| Torin-1 | 3.33E2 nM |  |  | -0.527 | -0.186 | -1.626 | -0.556 | -0.907 | -0.629 | -0.825 | -1.017 |
| Torin-1 | 1.11E2 nM |  |  | -0.485 | -0.182 | -1.636 | -0.506 | -0.886 | -0.523 | -0.842 | -0.832 |
| Torin-1 | 3.70E1 nM |  |  | -0.433 | -0.130 | -1.624 | -0.516 | -0.751 | -0.354 | -0.763 | -0.561 |
| Torin-1 | 1.23E1 nM |  |  | -0.248 | -0.095 | -1.257 | -0.539 | -0.453 | -0.154 | -0.672 | -0.310 |
| Torin-1 | 4.10E0 nM |  |  | -0.133 | -0.051 | -0.515 | -0.415 | -0.166 | 0.000 | -0.440 | -0.141 |
| Torin-1 | 1.40E0 nM |  |  | -0.096 | -0.035 | -0.330 | -0.257 | -0.141 | -0.002 | -0.211 | -0.037 |
| Torin-1 | 4.60E-1 nM |  |  | -0.038 | -0.016 | -0.162 | -0.232 | -0.114 | 0.065 | -0.136 | 0.023 |
| Torin-2 | 1.00E3 nM | Tocris Cookson | mTOR | -0.807 | -0.328 | -1.638 | -0.562 | -0.882 | -0.764 | -0.797 | -1.470 |
| Torin-2 | 3.33E2 nM |  |  | -0.684 | -0.290 | -1.682 | -0.580 | -0.952 | -0.630 | -0.888 | -1.298 |
| Torin-2 | 1.11E2 nM |  |  | -0.604 | -0.231 | -1.691 | -0.581 | -0.917 | -0.547 | -0.904 | -1.079 |
| Torin-2 | 3.70E1 nM |  |  | -0.497 | -0.183 | -1.651 | -0.570 | -0.849 | -0.405 | -0.825 | -0.755 |
| Torin-2 | 1.23E1 nM |  |  | -0.369 | -0.123 | -1.612 | -0.539 | -0.546 | -0.255 | -0.752 | -0.438 |
| Torin-2 | 4.10E0 nM |  |  | -0.163 | -0.071 | -0.842 | -0.470 | -0.366 | -0.039 | -0.609 | -0.213 |
| Torin-2 | 1.40E0 nM |  |  | -0.137 | -0.043 | -0.324 | -0.339 | -0.124 | 0.018 | -0.334 | -0.089 |
| Torin-2 | 4.60E-1 nM |  |  | -0.041 | -0.027 | -0.285 | -0.220 | -0.101 | 0.127 | -0.157 | 0.046 |
| UO126 | 1.00E4 nM | EMD | MEK | -0.336 | -0.161 | -0.720 | -0.471 | -0.536 | -0.643 | -0.798 | -0.712 |
| UO126 | 3.33E3 nM |  |  | -0.211 | -0.090 | -0.239 | -0.356 | -0.378 | -0.339 | -0.502 | -0.242 |
| UO126 | 1.11E3 nM |  |  | -0.143 | -0.061 | -0.090 | -0.216 | -0.113 | -0.152 | -0.338 | -0.061 |
| UO126 | 3.70E2 nM |  |  | -0.037 | -0.026 | -0.027 | -0.105 | 0.006 | -0.080 | -0.216 | -0.005 |
| UO126 | 1.24E2 nM |  |  | -0.048 | -0.015 | 0.007 | -0.044 | -0.039 | -0.003 | -0.174 | 0.009 |
| UO126 | 4.12E1 nM |  |  | -0.071 | -0.005 | 0.051 | -0.060 | 0.046 | -0.004 | -0.090 | -0.012 |
| UO126 | 1.37E1 nM |  |  | -0.077 | -0.010 | -0.008 | -0.087 | -0.023 | -0.002 | -0.046 | -0.001 |
| UO126 | 4.60E0 nM |  |  | -0.026 | -0.011 | -0.050 | -0.100 | -0.052 | 0.053 | -0.079 | 0.040 |
| Ursolic Acid | 1.00E4 nM | Biomol | STAT3 inhibitor | -0.218 | -0.052 | -1.430 | -0.362 | -0.160 | 0.008 | -0.034 | -0.030 |
| Ursolic Acid | 3.33E3 nM |  |  | -0.053 | -0.024 | 0.097 | -0.350 | -0.147 | -0.043 | -0.024 | -0.029 |
| Ursolic Acid | 1.11E3 nM |  |  | -0.027 | -0.003 | 0.053 | -0.210 | -0.008 | 0.129 | -0.005 | -0.026 |
| Ursolic Acid | 3.70E2 nM |  |  | -0.020 | -0.002 | -0.014 | -0.074 | -0.106 | -0.008 | 0.004 | -0.018 |
| Ursolic Acid | 1.24E2 nM |  |  | 0.011 | 0.000 | 0.050 | -0.114 | -0.027 | 0.032 | 0.005 | -0.021 |
| Ursolic Acid | 4.12E1 nM |  |  | 0.002 | -0.019 | 0.044 | 0.028 | -0.010 | -0.057 | 0.017 | -0.047 |
| Ursolic Acid | 1.37E1 nM |  |  | -0.018 | -0.001 | 0.038 | -0.038 | -0.033 | -0.029 | -0.070 | -0.040 |
| Ursolic Acid | 4.60E0 nM |  |  | -0.081 | -0.014 | 0.010 | -0.075 | -0.087 | -0.023 | -0.082 | -0.027 |
| Vioxx | 5.00E4 nM | Toronto Research Chemical | COX2 | 0.042 | 0.018 | -0.199 | 0.026 | 0.043 | 0.059 | -0.031 | 0.019 |
| Vioxx | 1.67E4 nM |  |  | -0.002 | 0.016 | -0.064 | -0.049 | 0.015 | 0.095 | -0.197 | 0.040 |
| Vioxx | 5.56E3 nM |  |  | 0.004 | -0.004 | 0.011 | -0.036 | -0.001 | 0.031 | 0.039 | 0.017 |
| Vioxx | 1.85E3 nM |  |  | -0.003 | -0.003 | -0.070 | -0.109 | -0.059 | 0.060 | -0.035 | 0.031 |
| Vorinostat | 3.33E3 nM | Active Biochem | HDAC6 | -0.679 | -0.225 | -1.768 | -0.787 | -0.886 | -0.774 | -0.905 | -0.902 |
| Vorinostat | 1.11E3 nM |  |  | -0.321 | -0.115 | -1.105 | -0.512 | -0.129 | -0.167 | 0.019 | -0.402 |
| Vorinostat | 3.70E2 nM |  |  | -0.121 | -0.055 | -0.848 | -0.220 | 0.029 | 0.063 | 0.111 | -0.078 |
| Vorinostat | 1.24E2 nM |  |  | 0.005 | 0.006 | 0.090 | 0.043 | 0.091 | 0.086 | 0.066 | 0.002 |
| Vorinostat | 4.12E1 nM |  |  | -0.035 | -0.004 | -0.015 | 0.059 | 0.043 | 0.072 | 0.020 | 0.013 |
| Vorinostat | 1.37E1 nM |  |  | -0.017 | -0.001 | 0.050 | 0.082 | 0.072 | 0.016 | 0.012 | 0.006 |
| Vorinostat | 4.60E0 nM |  |  | -0.035 | 0.005 | 0.003 | -0.056 | 0.007 | 0.030 | 0.028 | -0.012 |
| Vorinostat | 1.50E0 nM |  |  | -0.041 | -0.008 | 0.034 | -0.065 | 0.009 | -0.003 | 0.008 | 0.011 |
| Wortmannin | 3.70E2 nM | EMD | PI3K | -0.412 | -0.193 | -1.512 | -0.568 | -0.800 | -0.415 | -0.397 | -0.737 |
| Wortmannin | 1.24E2 nM |  |  | -0.321 | -0.167 | -1.290 | -0.533 | -0.834 | -0.449 | -0.367 | -0.621 |
| Wortmannin | 4.12E1 nM |  |  | -0.260 | -0.165 | -1.108 | -0.599 | -0.600 | -0.488 | -0.418 | -0.428 |
| Wortmannin | 1.37E1 nM |  |  | -0.158 | -0.094 | -0.177 | -0.476 | -0.589 | -0.243 | -0.123 | -0.155 |
| Wortmannin | 4.60E0 nM |  |  | -0.074 | -0.018 | -0.062 | -0.356 | -0.651 | -0.073 | -0.034 | -0.077 |
| Wortmannin | 1.50E0 nM |  |  | -0.027 | -0.014 | 0.023 | -0.440 | -0.550 | 0.017 | -0.064 | -0.036 |
| YM155 | 3.70E2 nM | Selleck Chem | Survivin inhibitor | -0.966 | -0.534 | -1.975 | -0.470 | -0.925 | -0.651 | -0.507 | -1.064 |
| YM155 | 1.24E2 nM |  |  | -0.734 | -0.349 | -1.988 | -0.455 | -0.868 | -0.548 | -0.316 | -0.934 |
| YM155 | 4.12E1 nM |  |  | -0.568 | -0.206 | -1.982 | -0.351 | -0.641 | -0.390 | -0.300 | -0.708 |
| YM155 | 1.37E1 nM |  |  | -0.476 | -0.130 | -1.958 | -0.299 | -0.453 | -0.338 | -0.414 | -0.638 |
| YM155 | 4.60E0 nM |  |  | -0.299 | -0.034 | -1.791 | -0.142 | -0.205 | -0.285 | -0.274 | -0.383 |
| YM155 | 1.50E0 nM |  |  | -0.055 | -0.002 | -0.851 | 0.070 | -0.072 | -0.136 | -0.140 | -0.134 |
| YM155 | 5.10E-1 nM |  |  | -0.004 | -0.003 | 0.037 | -0.019 | -0.016 | 0.009 | -0.019 | -0.005 |
| YM155 | 1.70E-1 nM |  |  | -0.027 | -0.010 | -0.061 | 0.091 | 0.101 | 0.028 | -0.028 | 0.036 |
| Zinc Chloride | 9.00E4 nM | Sigma | corrosive | -0.153 | -0.407 | -0.297 | -0.061 | 0.006 | 0.083 | 0.092 | 0.098 |
| Zinc Chloride | 3.00E4 nM |  |  | -0.038 | -0.030 | -0.017 | 0.071 | 0.026 | 0.059 | 0.052 | 0.082 |
| Zinc Chloride | 1.00E4 nM |  |  | -0.023 | -0.016 | -0.006 | 0.003 | 0.005 | 0.003 | 0.045 | 0.046 |
| Zinc Chloride | 3.33E3 nM |  |  | -0.079 | -0.018 | 0.041 | 0.052 | 0.036 | 0.030 | 0.046 | 0.037 |
| rhM-CSF | 1.00E0 nM | R&D Systems | CSF-1R ligand | 0.005 | -0.003 | -0.038 | -0.001 | -0.026 | 0.036 | 0.045 | -0.050 |
| rhM-CSF | 3.30E-1 nM |  |  | 0.018 | -0.009 | 0.035 | 0.019 | -0.015 | 0.015 | 0.052 | -0.052 |
| rhM-CSF | 1.10E-1 nM |  |  | -0.003 | -0.007 | -0.020 | 0.059 | -0.006 | 0.053 | 0.144 | -0.020 |
| rhM-CSF | 3.70E-2 nM |  |  | 0.000 | 0.012 | 0.041 | -0.059 | 0.028 | 0.080 | -0.024 | -0.012 |
| ssRNA40 | 1.00E2 ng/ml | Invivogen | TLR8 | 0.013 | 0.020 | 0.013 | 0.069 | 0.104 | 0.020 | 0.109 | 0.011 |
| ssRNA40 | 1.00E1 ng/ml |  |  | 0.026 | 0.003 | 0.075 | 0.015 | 0.115 | 0.163 | 0.200 | 0.113 |
| ssRNA40 | 1.00E0 ng/ml |  |  | 0.004 | 0.008 | -0.104 | 0.001 | 0.001 | 0.131 | 0.156 | 0.150 |
| ssRNA40 | 1.00E-1 ng/ml |  |  | -0.017 | -0.013 | -0.034 | -0.068 | -0.068 | 0.091 | 0.045 | 0.110 |
| trans-Retinoic Acid | 3.00E4 nM | Sigma | RAR/RXR Agonist | -0.099 | -0.072 | -1.492 | -0.651 | -0.937 | 0.072 | -0.129 | -0.300 |
| trans-Retinoic Acid | 1.00E4 nM |  |  | -0.033 | -0.051 | -0.714 | -0.260 | -0.439 | 0.260 | 0.008 | -0.054 |
| trans-Retinoic Acid | 3.33E3 nM |  |  | -0.027 | -0.009 | 0.036 | -0.050 | -0.221 | 0.315 | 0.014 | 0.052 |
| trans-Retinoic Acid | 1.11E3 nM |  |  | -0.030 | -0.002 | 0.104 | 0.054 | -0.269 | 0.287 | 0.032 | 0.008 |
| trans-Triprolidine | 2.00E5 nM | Tocris Cookson | H1 Antagonist | -0.379 | -0.127 | -1.645 | -0.335 | -0.423 | -0.603 | -0.664 | -0.633 |
| trans-Triprolidine | 6.67E4 nM |  |  | -0.122 | -0.049 | -0.431 | -0.179 | -0.142 | -0.172 | -0.144 | -0.158 |
| trans-Triprolidine | 2.22E4 nM |  |  | -0.012 | -0.030 | -0.076 | -0.061 | -0.017 | -0.104 | -0.071 | -0.035 |
| trans-Triprolidine | 7.41E3 nM |  |  | -0.010 | -0.021 | 0.059 | 0.034 | -0.016 | -0.037 | -0.008 | 0.017 |
| TWEAK | 5.00E2 ng/ml | R&D Systems | Fn14 ligand | -0.010 | -0.009 | -0.117 | -0.079 | -0.069 | 0.121 | 0.031 | 0.033 |
| TWEAK | 1.67E2 ng/ml |  |  | -0.023 | 0.023 | -0.015 | -0.075 | -0.056 | 0.053 | 0.047 | -0.027 |
| TWEAK | 5.56E1 ng/ml |  |  | 0.014 | 0.042 | 0.020 | -0.002 | -0.040 | -0.005 | 0.026 | -0.036 |
| TWEAK | 1.85E1 ng/ml |  |  | 0.008 | 0.004 | -0.019 | 0.030 | 0.001 | -0.015 | 0.066 | -0.055 |
