# Supplementary material for: Regulation of IL-17A Production Is Distinct from IL-17F in a Primary Human Cell Co-culture Model of T Cell-Mediated B Cell Activation
Source: PLoS One. 2013 Mar 7;8(3):e58966. doi: 10.1371/journal.pone.0058966 (PMC3591360; doi:10.1371/journal.pone.0058966)
Supplement: Table S4 — Lists of agents and their concentrations that regulated IL-17A and/or IL-17F in stimulated in BT co-cultures. Agents are listed that decreased both IL-17A and IL-17F, decreased IL-17A but not IL-17F, decreased IL-17F but not IL-17A, and increased either IL-17A or IL-17F. Agents were only included if they inhibited readouts greater than Log10 ratio −0.2 or increased readouts greater than Log10 ratio −0.15 at 2 or more non-cytotoxic concentrations. (DOC) [file pone.0058966.s004.doc]

**Table S4. Lists of agents and their concentrations that regulated IL-17A and/or IL-17F in stimulated in BT co-cultures.** Agents are listed that decreased both IL-17A and IL-17F, decreased IL-17A but not IL-17F, decreased IL-17F but not IL-17A, and increased either IL-17A or IL-17F. Agents were only included if they inhibited readouts greater than Log_10_ ratio -0.2 or increased readouts greater than Log_10_ ratio -0.15 at 2 or more non-cytotoxic concentrations.

| *Compounds that Decreased both IL-17A and IL-17F* | | |
| --- | --- | --- |
| Agent | Concentration | Class/Target |
| AEB071 | 1.11E3 nM | PKCtheta |
| AEB071 | 3.70E2 nM | PKCtheta |
| AEB071 | 1.24E2 nM | PKCtheta |
| AMG548 | 1.00E3 nM | p38 MAPK |
| AMG548 | 3.33E2 nM | p38 MAPK |
| AS703026 | 1.00E4 nM | MEK |
| AS703026 | 3.33E3 nM | MEK |
| AS703026 | 1.11E3 nM | MEK |
| AS703026 | 3.70E2 nM | MEK |
| AS703026 | 1.24E2 nM | MEK |
| AS703026 | 4.12E1 nM | MEK |
| AS703026 | 1.37E1 nM | MEK |
| AS703026 | 4.60E0 nM | MEK |
| BEZ-235 | 3.70E1 nM | mTOR/PI3K |
| BEZ-235 | 1.23E1 nM | mTOR/PI3K |
| BIRB-796 | 1.00E3 nM | p38 MAPK |
| BIRB-796 | 3.33E2 nM | p38 MAPK |
| BIRB-796 | 1.11E2 nM | p38 MAPK |
| BIRB-796 | 3.70E1 nM | p38 MAPK |
| BIRB-796 | 1.23E1 nM | p38 MAPK |
| BIRB-796 | 4.10E0 nM | p38 MAPK |
| CP-690550 | 1.00E4 nM | JAK |
| CP-690550 | 3.33E3 nM | JAK |
| CAL-101 | 3.33E2 nM | PI3Kdelta |
| CAL-101 | 1.11E2 nM | PI3Kdelta |
| Calcitriol | 1.00E3 nM | Vitamin D Receptor Agonist |
| Calcitriol | 3.33E2 nM | Vitamin D Receptor Agonist |
| Calcitriol | 1.11E2 nM | Vitamin D Receptor Agonist |
| Calcitriol | 3.70E1 nM | Vitamin D Receptor Agonist |
| Calcitriol | 1.23E1 nM | Vitamin D Receptor Agonist |
| Calcitriol | 4.10E0 nM | Vitamin D Receptor Agonist |
| Calcitriol | 1.40E0 nM | Vitamin D Receptor Agonist |
| Cerivastatin | 3.70E2 nM | HMG-CoA Reductase Inhibitor |
| Cerivastatin | 1.24E2 nM | HMG-CoA Reductase Inhibitor |
| Cerivastatin | 4.12E1 nM | HMG-CoA Reductase Inhibitor |
| Colchicine | 3.33E3 nM | Microtubule |
| Colchicine | 3.70E2 nM | Microtubule |
| Colchicine | 1.24E2 nM | Microtubule |
| Colchicine | 4.12E1 nM | Microtubule |
| Dasatinib | 1.24E2 nM | Src Kinase |
| Dasatinib | 4.12E1 nM | Src Kinase |
| Dasatinib | 1.37E1 nM | Src Kinase |
| Dexamethasone | 3.70E2 nM | GR Agonist |
| Dexamethasone | 1.24E2 nM | GR Agonist |
| Dexamethasone | 4.12E1 nM | GR Agonist |
| Dexamethasone | 1.37E1 nM | GR Agonist |
| Dexamethasone | 4.60E0 nM | GR Agonist |
| Erythromycin | 9.00E4 nM | bacteria 50S ribosome |
| Erythromycin | 3.00E4 nM | bacteria 50S ribosome |
| FK-506 | 1.11E3 nM | Calcineurin Inhibitor |
| FK-506 | 3.70E2 nM | Calcineurin Inhibitor |
| FK-506 | 1.24E2 nM | Calcineurin Inhibitor |
| FK-506 | 4.12E1 nM | Calcineurin Inhibitor |
| FK-506 | 1.37E1 nM | Calcineurin Inhibitor |
| FK-506 | 4.60E0 nM | Calcineurin Inhibitor |
| FK-506 | 1.50E0 nM | Calcineurin Inhibitor |
| FK-506 | 5.10E-1 nM | Calcineurin Inhibitor |
| GF 109203X | 3.33E3 nM | PKC (c+n) |
| GF 109203X | 1.11E3 nM | PKC (c+n) |
| GF 109203X | 3.70E2 nM | PKC (c+n) |
| IC-87114 | 3.33E3 nM | PI3Kdelta |
| IC-87114 | 1.11E3 nM | PI3Kdelta |
| IC-87114 | 3.70E2 nM | PI3Kdelta |
| IC-87114 | 1.24E2 nM | PI3Kdelta |
| IC-87114 | 4.12E1 nM | PI3Kdelta |
| MS-275 | 3.33E3 nM | HDAC Inhibitor |
| MS-275 | 1.11E3 nM | HDAC Inhibitor |
| PCI-32765 | 1.11E3 nM | BTK |
| PCI-32765 | 1.24E2 nM | BTK |
| PD184352 | 3.33E3 nM | MEK |
| PD184352 | 1.11E3 nM | MEK |
| PD184352 | 3.70E2 nM | MEK |
| PP 242 | 1.00E2 nM | mTOR |
| PP 242 | 1.00E1 nM | mTOR |
| PP 242 | 1.00E0 nM | mTOR |
| Paclitaxel | 1.00E4 nM | Microtubule |
| Paclitaxel | 3.33E3 nM | Microtubule |
| Paclitaxel | 1.11E3 nM | Microtubule |
| Paclitaxel | 1.24E2 nM | Microtubule |
| Prednisolone | 3.33E3 nM | GR Agonist |
| Prednisolone | 1.11E3 nM | GR Agonist |
| Prednisolone | 3.70E2 nM | GR Agonist |
| RO-5126766 | 1.00E4 nM | Raf/MEK |
| RO-5126766 | 3.33E3 nM | Raf/MEK |
| RO-5126766 | 1.11E3 nM | Raf/MEK |
| RO-5126766 | 3.70E2 nM | Raf/MEK |
| Rapamycin | 1.00E3 nM | mTOR |
| Rapamycin | 3.33E2 nM | mTOR |
| Rapamycin | 1.11E2 nM | mTOR |
| Rapamycin | 3.70E1 nM | mTOR |
| Rapamycin | 1.23E1 nM | mTOR |
| Rapamycin | 4.10E0 nM | mTOR |
| Rapamycin | 1.40E0 nM | mTOR |
| Rapamycin | 4.60E-1 nM | mTOR |
| SR2211 | 1.00E4 nM | RORγ |
| SR2211 | 3.33E3 nM | RORγ |
| Shaoguamycin B | 3.70E1 nM | NADH Dehydrogenase Inhibitor |
| Shaoguamycin B | 1.23E1 nM | NADH Dehydrogenase Inhibitor |
| Shaoguamycin B | 4.10E0 nM | NADH Dehydrogenase Inhibitor |
| Shaoguamycin B | 1.40E0 nM | NADH Dehydrogenase Inhibitor |
| Syk Inhibitor | 1.00E4 nM | Syk |
| Syk Inhibitor | 3.33E3 nM | Syk |
| Syk Inhibitor | 1.11E3 nM | Syk |
| Temsirolimus | 1.11E3 nM | mTOR |
| Temsirolimus | 3.70E2 nM | mTOR |
| Temsirolimus | 1.24E2 nM | mTOR |
| Temsirolimus | 4.12E1 nM | mTOR |
| Temsirolimus | 1.37E1 nM | mTOR |
| Temsirolimus | 4.60E0 nM | mTOR |
| Temsirolimus | 1.50E0 nM | mTOR |
| Temsirolimus | 5.10E-1 nM | mTOR |
| Torcetrapib | 3.00E4 nM | CETP Inhibitor |
| Torcetrapib | 1.00E4 nM | CETP Inhibitor |
| Torin-1 | 3.70E1 nM | mTOR |
| Torin-1 | 1.23E1 nM | mTOR |
| Torin-2 | 1.23E1 nM | mTOR |
| Torin-2 | 4.10E0 nM | mTOR |
| UO126 | 1.00E4 nM | MEK |
| UO126 | 3.33E3 nM | MEK |
| Wortmannin | 3.70E2 nM | PI3K |
| Wortmannin | 1.24E2 nM | PI3K |
| Wortmannin | 4.12E1 nM | PI3K |
| Wortmannin | 1.37E1 nM | PI3K |
| Wortmannin | 4.60E0 nM | PI3K |
| Wortmannin | 1.50E0 nM | PI3K |
| Wortmannin | 5.10E-1 nM | PI3K |
|  |  |  |
| *Compounds that Decreased IL-17A but not IL-17F* | | |
| Agent | Concentration | Class/Target |
| AMG548 | 1.11E2 nM | p38 MAPK |
| AMG548 | 3.70E1 nM | p38 MAPK |
| AMG548 | 1.23E1 nM | p38 MAPK |
| AMG548 | 4.10E0 nM | p38 MAPK |
| AMG548 | 1.40E0 nM | p38 MAPK |
| BEZ-235 | 4.10E0 nM | mTOR/PI3K |
| BEZ-235 | 1.40E0 nM | mTOR/PI3K |
| IC-87114 | 1.37E1 nM | PI3Kdelta |
| IC-87114 | 4.60E0 nM | PI3Kdelta |
| IC-87114 | 1.50E0 nM | PI3Kdelta |
| Shaoguamycin B | 4.60E-1 nM | NADH Dehydrogenase Inhibitor |
| Shaoguamycin B | 1.50E-1 nM | NADH Dehydrogenase Inhibitor |
| Shaoguamycin B | 5.10E-2 nM | NADH Dehydrogenase Inhibitor |
| Torin-1 | 4.10E0 nM | mTOR |
| Torin-1 | 1.40E0 nM | mTOR |
| Torin-1 | 4.60E-1 nM | mTOR |
| Torin-2 | 1.40E0 nM | mTOR |
| Torin-2 | 4.60E-1 nM | mTOR |
| Ursolic Acid | 3.33E3 nM | STAT3 inhibitor |
| Ursolic Acid | 1.11E3 nM | STAT3 inhibitor |
| Vorinostat | 1.11E3 nM | HDAC Inhibitor |
| Vorinostat | 3.70E2 nM | HDAC Inhibitor |
|  |  |  |
| *Compounds that Decreased IL-17F but not IL-17A* | | |
| Agent | Concentration | Class/Target |
| (+/-)-Nicotine | 4.00E1 ng/ml | Nicotinic Agonist |
| (+/-)-Nicotine | 2.00E1 ng/ml | Nicotinic Agonist |
| Aldosterone | 4.12E1 nM | MR Agonist |
| Aldosterone | 4.60E0 nM | MR Agonist |
| Axitinib | 9.00E3 nM | Tyrosine Kinase |
| Axitinib | 3.00E3 nM | Tyrosine Kinase |
| Axitinib | 1.00E3 nM | Tyrosine Kinase |
| CP-690550 | 1.11E3 nM | JAK |
| CP-690550 | 3.70E2 nM | JAK |
| Epothilone B | 3.33E2 nM | Microtubule |
| Epothilone B | 1.11E2 nM | Microtubule |
| Epothilone B | 3.70E1 nM | Microtubule |
| Epothilone B | 1.23E1 nM | Microtubule |
| Epothilone B | 4.10E0 nM | Microtubule |
| INCB-018424 | 1.24E2 nM | JAK |
| INCB-018424 | 4.12E1 nM | JAK |
| PD098059 | 3.00E4 nM | MEK |
| PD098059 | 1.00E4 nM | MEK |
| Paclitaxel | 3.70E2 nM | Microtubule |
| Paclitaxel | 4.12E1 nM | Microtubule |
| Picropodophyllin | 1.00E4 nM | Microtubule |
| Picropodophyllin | 3.33E3 nM | Microtubule |
| Picropodophyllin | 1.11E3 nM | Microtubule |
| Prostaglandin E2 | 1.00E4 nM | EP Agonist |
| Prostaglandin E2 | 3.33E3 nM | EP Agonist |
|  |  |  |
| *Compounds that Increased IL-17A or IL-17F* | | |
| Agent | Concentration | Class/Target |
| BW 245C | 1.00E4 nM | DP Agonist |
| BW 245C | 3.33E3 nM | DP Agonist |
| BW 245C | 3.70E2 nM | DP Agonist |
| FSL-1 | 1.00E2 ng/ml | TLR2/6 |
| FSL-1 | 1.00E1 ng/ml | TLR2/6 |
| Flagellin | 1.00E2 ng/ml | TLR5 |
| Flagellin | 1.00E1 ng/ml | TLR5 |
| HKLM | 1.00E7 mU/ml | TLR2 |
| HKLM | 1.00E6 mU/ml | TLR2 |
| HKLM | 1.00E5 mU/ml | TLR2 |
| Iloprost | 3.00E4 nM | EP Agonist |
| Iloprost | 1.00E4 nM | EP Agonist |
| Iloprost | 3.33E3 nM | EP Agonist |
| Iloprost | 1.11E3 nM | EP Agonist |
| IL-2 | 3.30E-1 nM | IL-2R ligand |
| IL-2 | 1.10E-1 nM | IL-2R ligand |
| LPS | 1.00E2 ng/ml | TLR4 |
| LPS | 1.00E1 ng/ml | TLR4 |
| LPS | 1.00E-1 ng/ml | TLR4 |
| Prostaglandin E1 | 3.33E3 nM | EP Agonist |
| Prostaglandin E1 | 3.70E2 nM | EP Agonist |
| Prostaglandin E2 | 3.33E3 nM | EP Agonist |
| Prostaglandin E2 | 1.11E3 nM | EP Agonist |
| Prostaglandin E2 | 3.70E2 nM | EP Agonist |
